# Supplementary material for: Iron-catalyzed aliphatic C–H functionalization to construct carbon–carbon bonds
Source: Nat Commun. 2025 May 20;16:4673. doi: 10.1038/s41467-025-60010-1 (PMC12092725; doi:10.1038/s41467-025-60010-1)
Supplement: Supplementary file 1 — Supplementary Information [file 41467_2025_60010_MOESM1_ESM.pdf]

– Supplementary Information –

Iron-catalyzed aliphatic C–H functionalization to construct carbon–carbon bonds

Lulu Zhou[1,2†], Hengrui Cai[1,2†], Dong Xie[1,2†], Kangkang Sun[1,2†], Shanmei Zhu[1,2], Mengying Guo[1,2], Wei Han[1,2]*

[1] State Key Laboratory of Microbial Technology, Jiangsu Collaborative Innovation Center of Biomedical Functional Materials, Jiangsu Key Laboratory of Biofunctional Materials, Jiangsu Key Laboratory of New Power Batteries, Nanjing Normal University, Wenyuan Road No.1, 210023 Nanjing, China

[2] School of Chemistry and Materials Science, Nanjing Normal University, Wenyuan Road No.1, 210023 Nanjing, China

[†]These authors contributed equally to this work.

[*]Corresponding author. E-mail: whhanwei@outlook.com

Table of Contents

Content	Page
**General information**	S3
**Preliminary mechanistic studies**	S4
**Optimization of reaction conditions**	S13
**Procedure for the preparation of linear bis-peptide ligands**	S15
**[Fe]/BCMOM-catalyzed alkane C-H functionalization**	S18
**Copies of NMR spectra**	S140
**References**	S457

General Information

Reagents. All reactions were carried out under air atmosphere unless otherwise noted. Commercially available reagents and materials were used without further purification. $Fe(acac)_2$ (99.95% pure) from Aldrich, $FeCl_2$ (anhydrous, 99.99%) from Aldrich, and H_2O_2 (35% w/w aq. solution) from Alfa, were used as received. MeCN (from Adamas-beta® and Energy chemical) was purified prior to use by distillation. Water is deionized and brine refers to a saturated aqueous solution of NaCl.

Analytical methods. Reactions were monitored by thin layer chromatography (TLC) carried out on 0.25 mm Yantai silica plates (GF-254), using shortwave UV light (254 nm) as the visualizing agent or iodine (mixed with silica gel) stain in case of no UV activity. The crude product was purified by silica gel column chromatography was performed using Yantai silica gel (300-400 mesh). ^1H (400 MHz) and ^{13}C NMR spectra (100 MHz) of solutions in $CDCl_3$, CD_3COCD_3 or DMSO-d_6 were recorded on a Bruker Avance 400 NMR spectrometer. Chemical shifts were expressed in parts per million (ppm) downfield from tetramethylsilane and refer to the solvent signals ($CDCl_3$: δ_H 7.26 and δ_C 77.0 ppm; CD_3COCD_3: δ_H 2.05 and δ_C 29.8, 206.3 ppm; DMSO-d_6: δ_H 2.50 and δ_C 39.50 ppm). The signals of water were observed at about 1.58 ppm in $CDCl_3$, 2.84 ppm in CD_3COCD_3 and 3.33 ppm in DMSO-d_6, respectively. Signals appearing around 3.70 ppm in ^1H NMR and ~58 ppm in ^{13}C NMR in some spectra are attributed to trace residual ethanol from purification. Abbreviations for signal couplings are: br, broad; s, singlet; d, doublet; t, triplet; m, multiplet; dd, doublet of doublets; dt, triplet of doublets; td, doublet of triplets; tt, triplet of triplets; tdd, doublet of doublet of triplets. Coupling constants, *J*, were reported in hertz unit (Hz). HRMS was performed on a Q-TOF mass spectrometer. Infrared spectra of neat substances were recorded on a Thermo Nicolet Corporation GC-FTIR NEXUS670 spectrometer. GC-MS was determined with Agilent 7890-5975C.

Definition of "Unknown Compound". In this study, "unknown compound" refers to newly discovered compounds that have not been previously reported in the literature.

Preliminary Mechanistic Studies

(1) Effect of radical scavengers

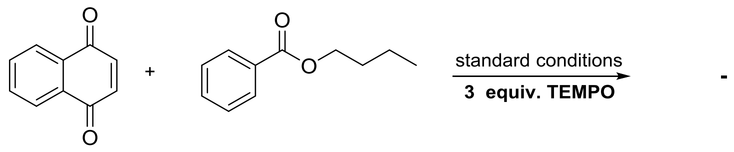

Supplementary Fig. 1 Effect of TEMPO on the reaction.

As *general procedure A*: A 25 mL flask was charged with Fe(acac)$_2$ (0.0125 mmol, 3.3 mg), BCMOM (0.025 mmol, 18.6 mg), 1,4-naphthoquinone (0.25 mmol, 40.3 mg) before standard cycles of evacuation and backfilling with dry and pure N_2, and then solvents CH_3CN (2 mL) and H_2O (2 mL) was added. The reaction mixture was stirred under N_2 atmosphere at room temperature for 10 min, and then butyl benzoate (0.5 mmol, 89 μL), H_2O_2 (1.5 mmol, 129 μL), and a radical scavenger TEMPO (0.75 mmol, 119.6 mg) was added into the stirring reaction mixture. Upon completion, the reaction mixture was stirred at 80 ℃ for 1.0 hour. Consequently, the desired product was not observed. The result suggests that a radical intermediate may involve the transformation process.

(2) Trapping of alkyl radicals by $BrCCl_3$

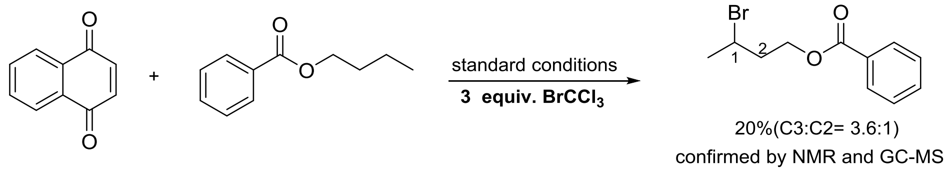

Supplementary Fig. 2 Radical trapping experiment with $BrCCl_3$.

As *general procedure A*: A 25 mL flask was charged with Fe(acac)$_2$ (0.025 mmol, 6.6 mg), BCMOM (0.05 mmol, 37.2 mg), 1,4-naphthoquinone (0.5 mmol, 80.6 mg) before standard cycles of evacuation and backfilling with dry and pure N_2, and then solvents CH_3CN (4 mL) and H_2O (4 mL) was added. The reaction mixture was stirred under N_2 atmosphere at room temperature for 10 min, and then butyl benzoate (1.0 mmol, 178 μL), H_2O_2 (1.5 mmol, 129 μL), and a radical scavenger $BrCCl_3$ (1.5 mmol,

306 μL) was added into the reaction mixture. Upon completion, the reaction mixture was stirred at 80 ℃ for 1.0 hour. After the mixture was cooled to room temperature, filtered through Celite and then washed with ethyl acetate (3 x 5 mL). The organic phases were combined, dried (Na_2SO_4) and concentrated to give the crude product. The residue was purified by column chromatography (Petroleum ether/ ethyl acetate) on silica gel to afford the bromide. This result implicates a pathway involving free alkyl radicals.

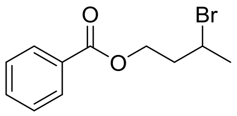

3-Bromobutyl benzoate: The NMR spectroscopic data agree with those described in ref.[S1].

^{1}H NMR (400 MHz, $CDCl_3$): δ 8.07-8.01 (m, 2 H), 7.59-7.54 (m, 1 H), 7.47-7.43 (m, 2 H), 4.54 (dt, *J* = 11.2, 5.7 Hz, 1 H), 4.45 (ddd, *J* = 11.2, 7.8, 5.7 Hz, 1 H), 4.34-4.26 (m, 1 H), 2.33-2.18 (m, 2 H), 1.80 (d, *J* = 6.7 Hz, 3 H) ppm; ^{13}C NMR (100 MHz, $CDCl_3$): δ 166.4, 133.1, 129.6, 128.4, 63.0, 47.0, 39.8, 26.6 ppm.

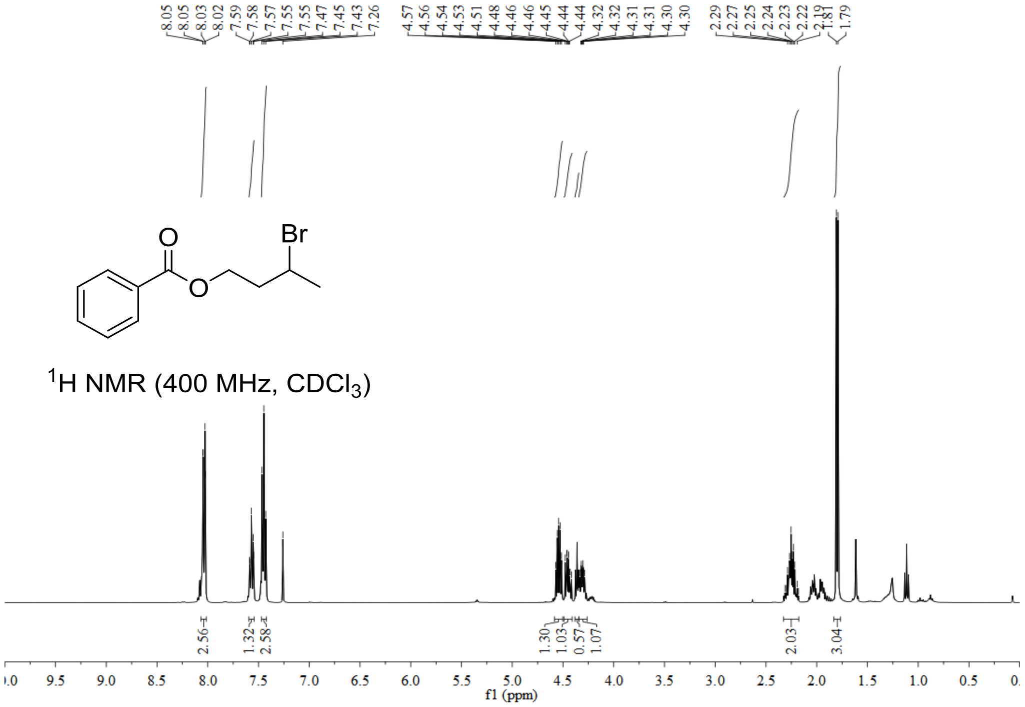

Supplementary Fig. 3 ^{1}H NMR confirmation of $BrCCl_3$ trapping product.

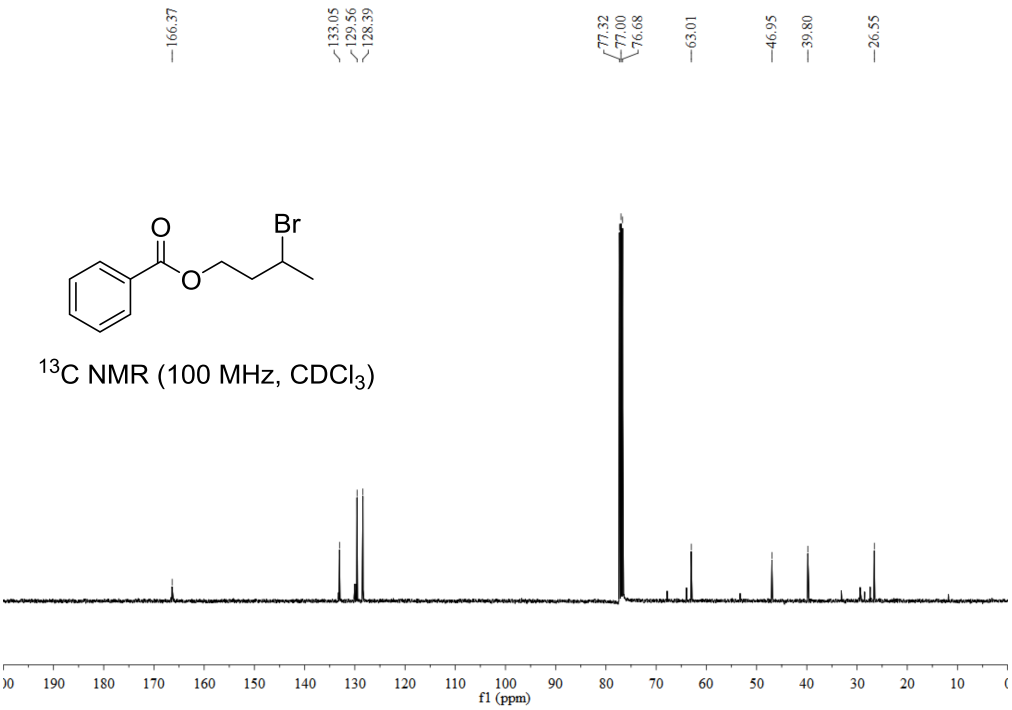

Supplementary Fig. 4 ^{13}C NMR confirmation of $BrCCl_3$ trapping product.

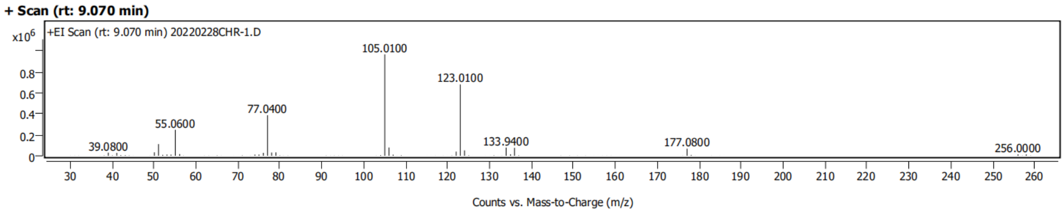

Supplementary Fig. 5 GC-MS spectrum of the $BrCCl_3$ trapping product

(3) Kinetic isotope effect

Initial rate constants: As per *general procedure A*, reaction of cyclohexane (0.5 mmol, 54 μL, 2 equiv), or cyclohexane-D_{12} (0.5 mmol, 54 μL, 2 equiv), with 1,4-naphthoquinone (0.25 mmol, 40.3 mg, 1 equiv) was conducted. The KIE was determined for the reaction time between 0 and 50 min. The resulting crude sample was analyzed by GC using mesitylene as internal standard to assess yield of **42** or **42-d**: k_H/k_D=2.05 suggests C-H cleavage is the rate-determining step.

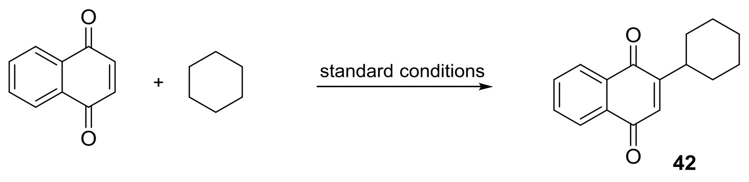

Supplementary Fig. 6 Determination of the kinetic isotope effect (KIE) using cyclohexane.

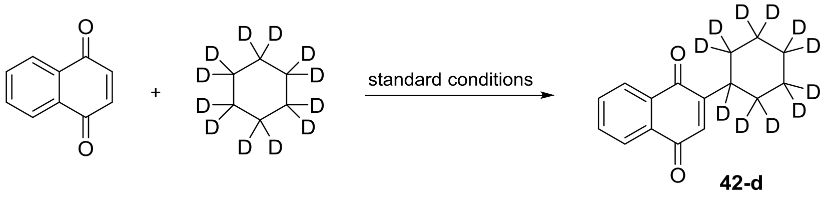

k_H/k_D = 0.9216/0.45= 2.05

Supplementary Fig. 7 Determination of the kinetic isotope effect (KIE) using cyclohexane-D_{12}.

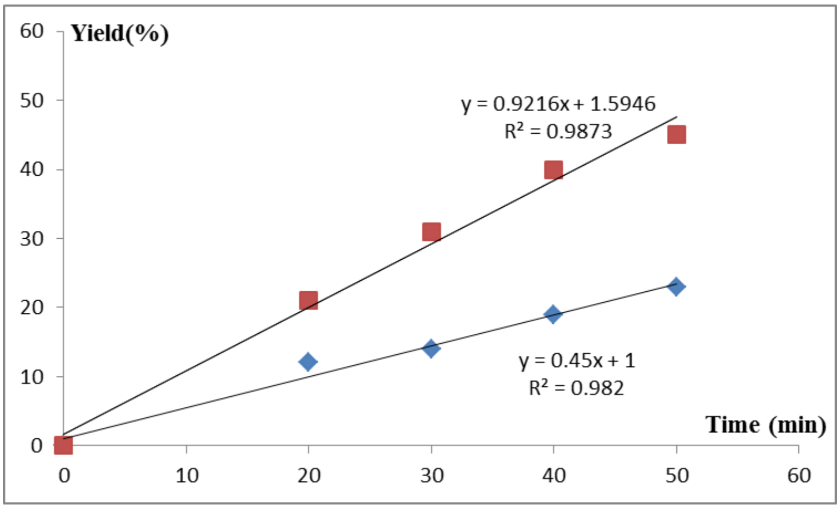

Supplementary Fig. 8 Initial rates of the reaction with cyclohexane(red) or cyclohexane-D_{12} (blue)

Intermolecular competition

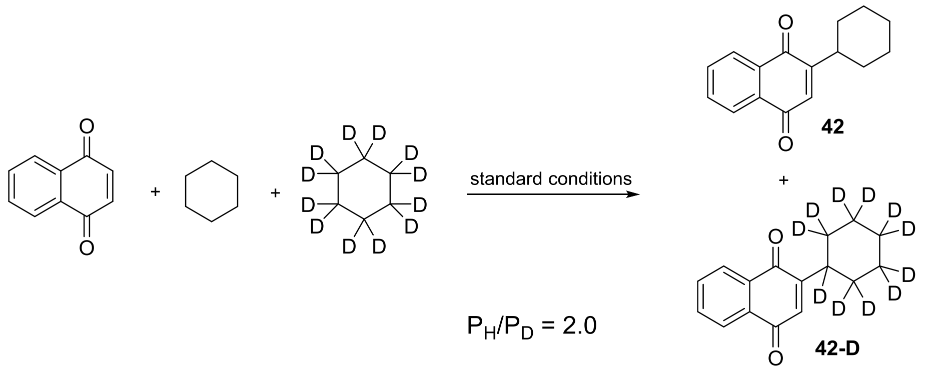

Supplementary Fig. 9 Intermolecular competition between cyclohexane and cyclohexane-D_{12}

Following *general procedure A:* reaction of cyclohexane (0.25 mmol, 27 μL, 1 equiv), and cyclohexane-D_{12} (0.25 mmol, 27 μL, 1 equiv), with 1,4-naphthoquinone (0.25 mmol, 40.3 mg, 1 equiv), was undertaken. The reaction mixture was stirred at 80 °C for 30 min. The mixture was then allowed to get to room temperature, filtered through Celite and then washed with ethyl acetate (3 x 5 mL). The organic phases were combined, dried (Na_2SO_4) and concentrated to give the crude product. Then, the volatiles were removed and the analytically pure product was obtained by flash chromatography. The P_H/P_D of 2.0 for the formation of the products was measured via ^1H NMR, indicating that C-H cleavage of alkane is involved in the rate determining step.

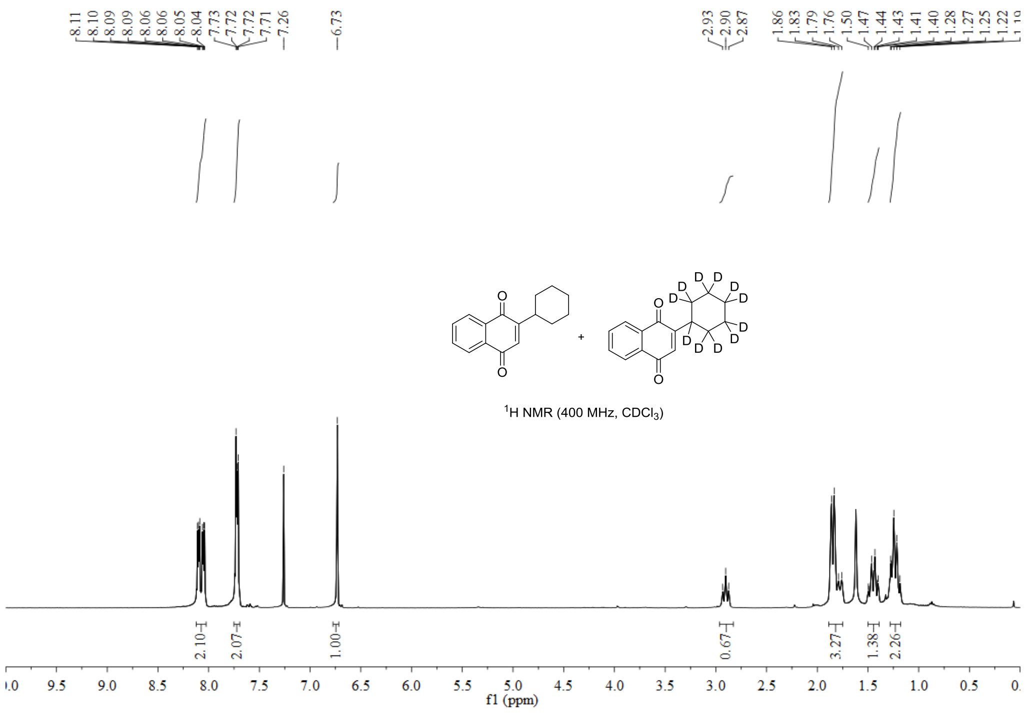

Supplementary Fig. 10 ^1H NMR spectrum of the alkylation products 42 and 42-D

(4) The effect of $H_2{}^{18}O$ investigated by substitution of the substrate with acetanilide

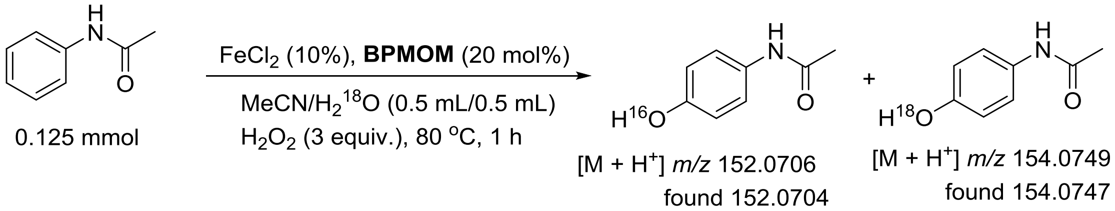

Supplementary Fig. 11 Isotope labeling experiment with $H_2{}^{18}O$ to probe the origin of the oxygen atom in the hydroxylated product.

The general procedure A was applied with acetanilide (17.1 mg, 0.125 mmol, 1 equiv.), $FeCl_2$ (1.6 mg, 0.0125 mmol, 0.1 equiv.), **BCMOM** (18.6 mg, 0.025 mmol, 0.2 equiv.), H_2O_2 (32 μL, 0.375 mmol, 3 equiv.), CH_3CN (0.5 mL) and $H_2{}^{18}O$ (0.5 mL) at 80 ℃ for 1 h. Then, the analytically pure product was obtained by flash chromatography and was measured via HRMS. The ratio of ^{16}O to ^{18}O-labelled product was 3.3:0.08, that is, $H_2{}^{18}O$ leads to a small but distinct amount (2.4%) of ^{18}O incorporation into the product. The reason for the small amount of ^{18}O incorporation is in that the rate of oxygen exchange between high-valent iron oxo intermediate and $H_2{}^{18}O$ is much slower than that of oxygen transfer from the intermediate to the arene substrate (C-H cleavage of arene was not involved in the rate determing step)[S2], as demonstrated in previous studies[S3-S4]. This result suggests the formation of oxoiron under normal reaction conditions.

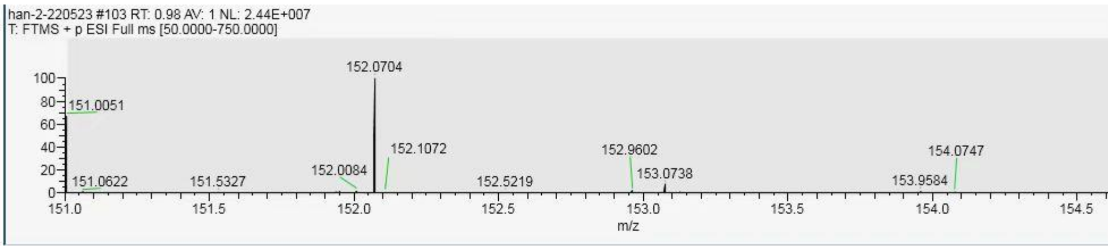

Supplementary Fig. 12 HRMS analysis of products from the $H_2{}^{18}O$-labeling experiment.

(5) HRMS spectrum showing the formation of high-valent oxoiron species in the presence of H_2O_2

The general procedure A was applied with $FeCl_2$ (1.6 mg, 0.0125 mmol, 0.1 equiv.), **BCMOM** (18.6 mg, 0.025 mmol, 0.2 equiv.), CH_3CN (1.0 mL) and H_2O (1.0 mL) at 80 ℃ for 1 h. Then, the reaction mixture was measured via HRMS, which led to found the species $[(BCMOM)_2Fe(acac)_2 + H]^+$ (m/z, 1715.6495).

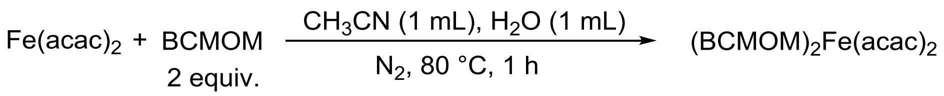

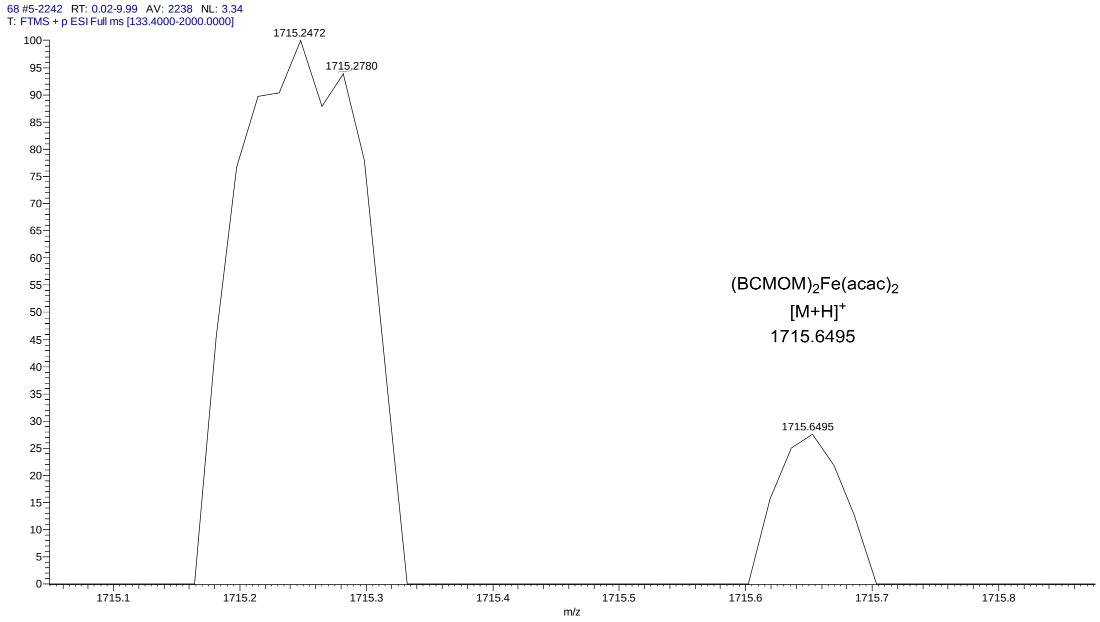

Supplementary Fig. 13 HRMS spectrum of (BCMOM)$_2$Fe(acac)$_2$.

The general procedure A was applied with FeCl$_2$ (1.6 mg, 0.0125 mmol, 0.1 equiv.), **BCMOM** (18.6 mg, 0.025 mmol, 0.2 equiv.), H$_2$O$_2$ (32 μL, 0.375 mmol, 3 equiv.), CH$_3$CN (1.0 mL) and H$_2$O (1.0 mL) at 80 ℃ for 1 h. Then, the reaction mixture was measured via HRMS, which led to found the species found the species [(acac)$_2$(BCMOM)$_2^{\bullet+}$Fe^{IV}(O) + H]$^+$ (m/z, 1731.4928).

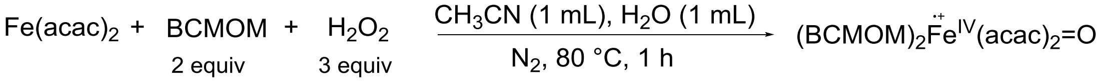

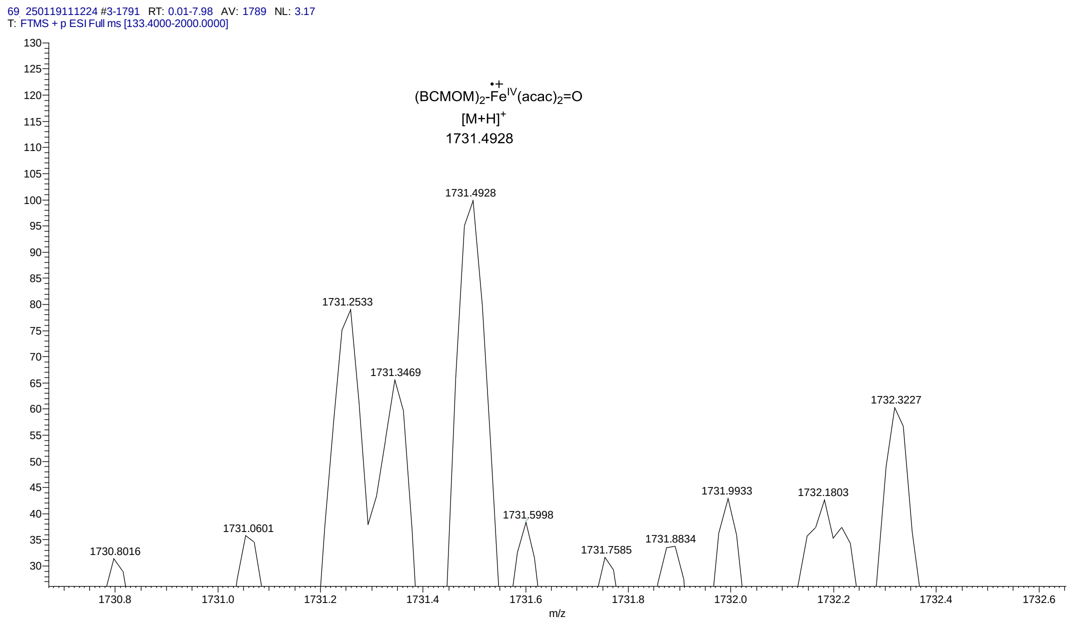

Supplementary Fig. 14 HRMS spectrum confirming the formation of oxoiron(IV) species.

(6) Aminated intermediate in cyclization of 1,4-quinones

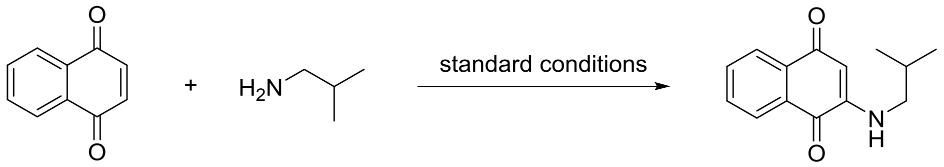

Supplementary Fig. 15 Aminated intermediate in cyclization of 1,4-quinones.

Following *general procedure C*, a reaction of 1,4-naphthoquinone (80.7 mg, 0.5 mmol, 1 equiv.), 2-methylpropan-1-amine (99.9 μL, 1.0 mmol, 2 equiv.), H_2SO_4 (67.5 μL, 2.4 equiv.), BCMOM (37.2 mg, 0.05 mmol, 0.1 equiv.), $FeCl_2$ (3.2 mg, 0.025 mmol, 0.05 equiv.), and H_2O_2 (129 μL, 1.5 mmol, 3 equiv.) at 80 ℃ for 0.5 h. Column chromatography (PE/EA/Et_3N, 20:1:0.1) afforded the aminated product as a yellow solid (45.8 mg, 40%). The result suggests that the process of cyclization of 1,4-quinones is initiated by dehydrogenative amination with 1,4-quinones followed by cyclization via internal molecular C_{sp3}-C_{sp2} coupling.

R_f = 0.40 (silica gel, PE/EA/Et_3N, 20:1:0.1).

^{1}H NMR (400 MHz, $CDCl_3$): δ 8.10 (d, J = 7.5 Hz, 1 H), 8.05 (d, J = 7.6 Hz, 1 H),

7.73 (t, J = 7.3 Hz, 1 H), 7.61 (t, J = 7.5 Hz, 1 H), 5.98 (s, 1 H), 5.73 (s, 1 H), 3.01 (t, J = 6.4 Hz, 2 H), 2.05-1.95 (m, 1 H), 1.01 (d, J = 6.7 Hz, 6 H) ppm; ^{13}C NMR (100 MHz, $CDCl_3$): δ 183.0, 182.0, 148.1, 134.8, 133.7, 131.9, 130.5, 126.3, 126.2, 100.8, 50.1, 27.6, 20.4 ppm.

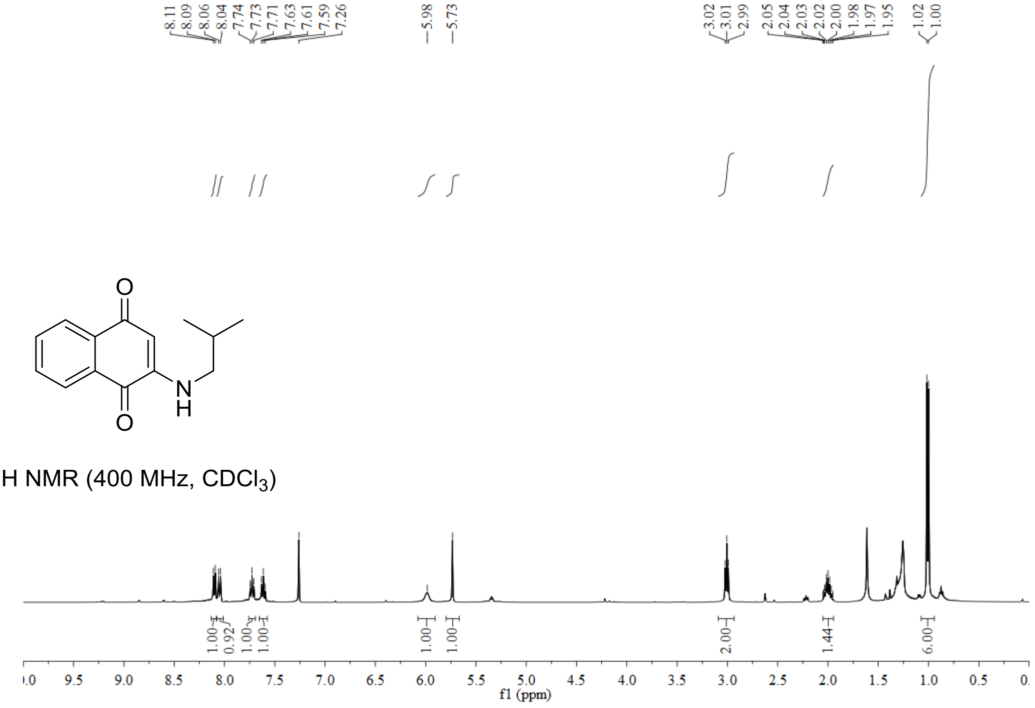

Supplementary Fig. 16 ^{1}**H NMR spectrum of the aminated intermediate.**

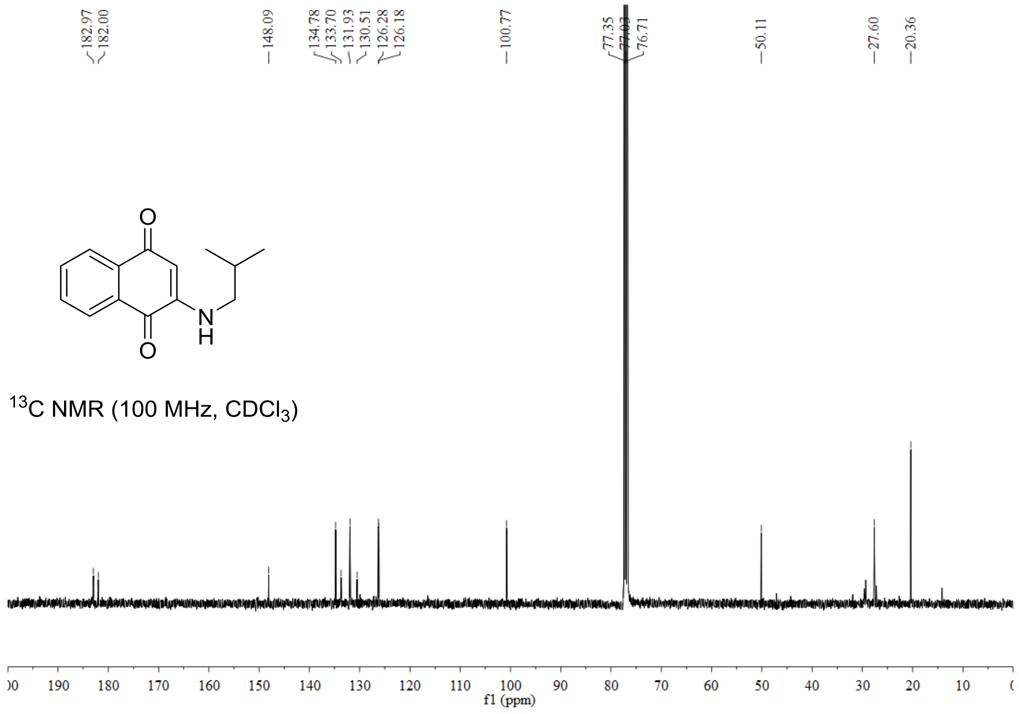

Supplementary Fig. 17 ^{13}C NMR spectrum of the aminated intermediate.

Optimization of reaction conditions

Table S1: Optimization Studies

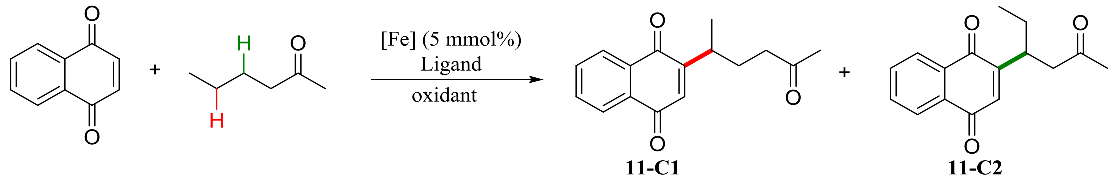

Entry	[Fe]	Ligand	[O]	Solvent	Yield of **11-C1**/%	Yield of **11-C2**/%
1	Fe(acac)$_2$	-	H_2O_2	MeCN/H_2O	-	-
2	Fe(acac)$_2$	L-Serine	H_2O_2	MeCN/H_2O	10	4
3	-	L-Serine	H_2O_2	MeCN/H_2O	-	-
4	Fe(acac)$_2$	L-Cysteine	H_2O_2	MeCN/H_2O	40	18
5	Fe(acac)$_2$	L-Cystine	H_2O_2	MeCN/H_2O	44	20
6	Fe(acac)$_2$	(BOC-CYS-OH)$_2$	H_2O_2	MeCN/H_2O	47	20
7	Fe(acac)$_2$	BCPOM	H_2O_2	MeCN/H_2O	33	15
8	Fe(acac)$_2$	BCPOH	H_2O_2	MeCN/H_2O	30	15
9	Fe(acac)$_2$	CPOM	H_2O_2	MeCN/H_2O	12	10
10	Fe(acac)$_2$	CPOH	H_2O_2	MeCN/H_2O	28	15
**11**	**Fe(acac)$_2$**	**BCMOM**	**H_2O_2**	**MeCN/H_2O**	**58**	**25**
12	Fe(acac)$_2$	BCGOM	H_2O_2	MeCN/H_2O	39	16
13	Fe(acac)$_2$	BC*t*LOM	H_2O_2	MeCN/H_2O	46	20
14	Fe(acac)$_2$	BCSOM	H_2O_2	MeCN/H_2O	49	17
15	-	BCMOM	H_2O_2	MeCN/H_2O	-	-
**16**	**$FeCl_2$**	**BCMOM**	**H_2O_2**	**MeCN/H_2O**	**54**	**25**
17	$FeSO_4$•$7H_2O$	BCMOM	H_2O_2	MeCN/H_2O	50	22
18	$Fe(NO_3)_3$•$9H_2O$	BCMOM	H_2O_2	MeCN/H_2O	50	22
19	FePc	BCMOM	H_2O_2	MeCN/H_2O	32	18
20	$FeCl_3$	BCMOM	H_2O_2	MeCN/H_2O	54	25
21	Fe(OTf)$_3$	BCMOM	H_2O_2	MeCN/H_2O	50	26

22	$Fe(ClO_4)_3$	BCMOM	H_2O_2	$MeCN/H_2O$	54	25
23	$Fe(AcO)_2$	BCMOM	H_2O_2	$MeCN/H_2O$	40	20
24	$FeC_2O_4{\bullet}2H_2O$	BCMOM	H_2O_2	$MeCN/H_2O$	45	22
25	$Fe(acac)_2$	BCMOM	-	$MeCN/H_2O$	-	-
26	$Fe(acac)_2$	BCMOM	$K_2S_2O_8$	$MeCN/H_2O$	10	3
27	$Fe(acac)_2$	BCMOM	$Na_2S_2O_8$	$MeCN/H_2O$	10	3
28	$Fe(acac)_2$	BCMOM	TBHP	$MeCN/H_2O$	-	-
29	$Fe(acac)_2$	BCMOM	DTBP	$MeCN/H_2O$	-	-
30	$Fe(acac)_2$	BCMOM	H_2O_2	MeCN	-	-
31	$Fe(acac)_2$	BCMOM	H_2O_2	DCM/H_2O	-	-
32	$Fe(acac)_2$	BCMOM	H_2O_2	$DMSO/H_2O$	-	-
33	$Fe(acac)_2$	BCMOM	H_2O_2	$EtOH/H_2O$	-	-
34	$Fe(acac)_2$	BCMOM	H_2O_2	Et_2O/H_2O	-	-
35[a]	$Fe(acac)_2$	BCMOM	H_2O_2	$MeCN/H_2O$	55	21
36[b]	$Fe(acac)_2$	BCMOM	H_2O_2	$MeCN/H_2O$	43	17
37[c]	$Fe(acac)_2$	BCMOM	H_2O_2	$MeCN/H_2O$	38	16
38[d]	$Fe(acac)_2$	BCMOM	H_2O_2	$MeCN/H_2O$	59	23
39[e]	$Fe(acac)_2$	BCMOM	H_2O_2	$MeCN/H_2O$	34	16
40[f]	$Fe(acac)_2$	BCMOM	H_2O_2	$MeCN/H_2O$	45	20
41[g]	$Fe(acac)_2$	BCMOM	H_2O_2	$MeCN/H_2O$	46	19
42[h]	$Fe(acac)_2$	BCMOM	H_2O_2	$MeCN/H_2O$	58	26

Reaction conditions: 1,4-naphthoquinone (0.25 mmol), 2-hexanone (2.0 equiv.), [Fe] (5 mol%), ligand (10 mol%), H_2O_2 (3.0 equiv.), solvent (solvent:H_2O = 2 mL:2 mL), 80 ℃, 1 h, and N_2. Isolated yields are given. [a] $Fe(acac)_2$ (7 mol%) and BCMOM (14 mol%). [b] $Fe(acac)_2$ (3 mol%) and BCMOM (6 mol%). [c] BCMOM (5 mol%). [d] BCMOM (15 mol%). [e] H_2O_2 (2.0 equiv.). [f] H_2O_2 (4.0 equiv.). [g] 70 ℃. [h] 90 ℃.

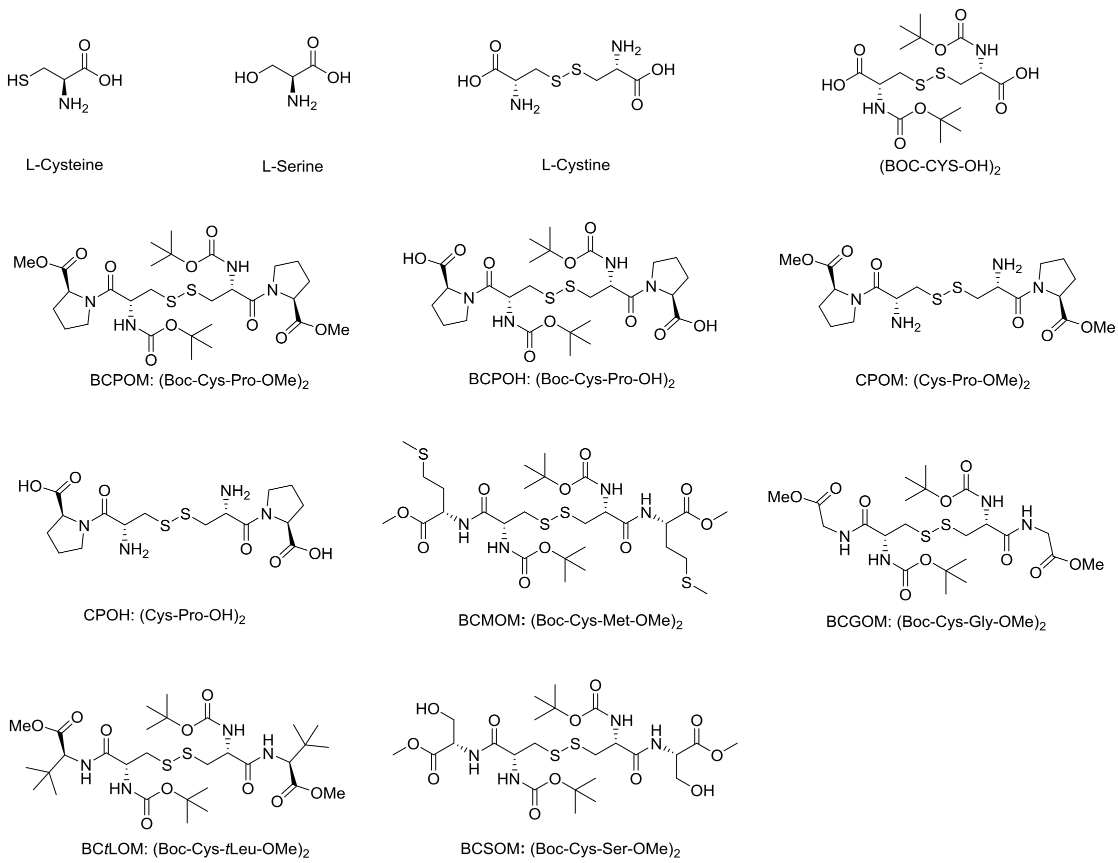

Supplementary Fig. 18 Structures of different ligands used in this study.

General procedure for screening reactions: A 25 mL flask was charged with [Fe] (0.0125 mmol), ligand (0.025 mmol), 1,4-naphthoquinone (40.3 mg, 0.25 mmol, 1 equiv.), and 2-hexanone (63 μL, 0.5 mmol) before standard cycles of evacuation and backfilling with dry and pure N_2, and then solvents CH_3CN (2 mL) and H_2O (2 mL) were added. The reaction mixture was stirred under N_2 atmosphere at room temperature for 10 min, and then H_2O_2 (64 μL, 0.75 mmol) was added dropwise into the stirring reaction mixture. Upon completion, the reaction mixture was stirred at 80 ℃ until no further changes were observed by TLC. The mixture was then allowed to get to room temperature, and extracted with EtOAc (3 x 10 mL). The organic phases were combined and concentrated to give the crude product. The residue was further purified by column chromatography (petroleum ether/ ethyl acetate) on silica gel to afford the corresponding products.

Procedures for the Preparation of Linear Bis-peptide ligands

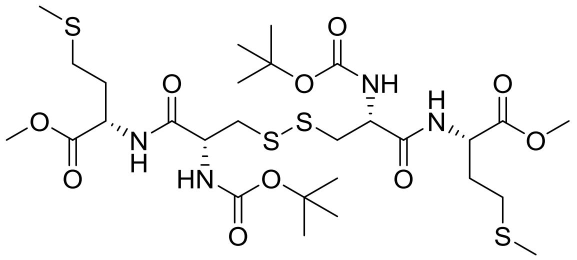

BCMOM [(Boc-Cys-Met-OMe)$_2$]**:**

A flask was charged with *N,N'*-Bis-(*tert*-butoxycarbonyl)-L-cystine (449.5 mg, 1.0 mmol, 1.0 equiv.), L-methionine methylester hydrochloride (407.6 mg, 2.0 mmol, 2 equiv.) before standard cycles of evacuation and back-filling with dry and pure N_2. Subsequently, 6 mL of anhydrous CH_2Cl_2 and *N*-methylmorphine (NMM) (266.5 uL, 2.4 mmol, 2.4 equiv.) were added and the mixture was stirred at -5 ℃ (ice-salt bath). Then, to the mixture was added a 6 mL of anhydrous CH_2Cl_2 solution of *N,N'*-dicyclohexylcarbodiimide (DCC) (467.9 mg, 2.2 mmol, 2.2 equiv.) dropwise over a period of 0.5 h. The resulting reaction mixture continued to be stirred at -5 ℃ for 2 h and thereafter was left on stirring over-night at room temperature until the reaction was complete (observed by TLC). The precipitate of the reaction was filtrated and washed with DCM (3 × 10 mL). The organic phases were combined and evaporated under reduced pressure. The residue was purified by column chromatography (Petroleum ether/ ethyl acetate from 10:5 to 10:6) on silica gel to afford the corresponding product as a white solid (534 mg, 70%), R_f = 0.30 (silica gel, PE/EA, 10:6). ^{1}H NMR (400 MHz, $CDCl_3$) δ 7.76 (d, *J* = 8.2 Hz, 2 H), 5.54 (d, *J* = 9.4 Hz, 2 H), 4.82 (s, 2 H), 4.74 (td, *J* = 8.7, 5.3 Hz, 2 H), 3.71 (s, 6 H), 3.08 (dd, *J* = 14.5, 3.7 Hz, 2 H), 2.95 – 2.85 (m, 2 H), 2.63 – 2.49 (m, 4 H), 2.21 (dt, *J* = 14.0, 7.2 Hz, 2 H), 2.08 (s, 6 H), 1.97 (dt, *J* = 14.1, 8.4 Hz, 2 H), 1.44 (s, 18 H) ppm; ^{13}C NMR (100 MHz, $CDCl_3$) δ 171.81, 170.51, 155.68, 80.10, 54.17, 52.38, 51.29, 46.61, 31.04, 30.40, 28.26, 15.33 ppm; HRMS (ESI) calcd. for $C_{28}H_{50}N_4O_{12}S_4H^+$ [M + H$^+$] *m/z* 763.2387, found: 763.2390; IR (KBr, cm^{-1}): ν_{max} 3333, 2976, 2923, 2855, 1742, 1664, 1520, 1367, 1312, 1250, 1168, 1044, 1019, 863, 642; Mp: 305.3-306.0 ℃.

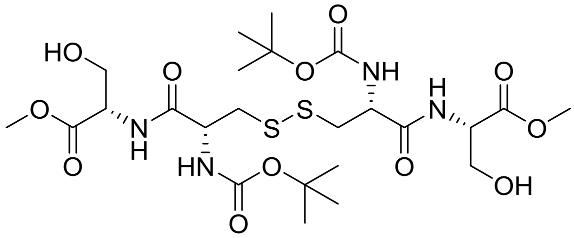

BCSOM [(Boc-Cys-Ser-OMe)$_2$]:

A flask was charged with *N,N'*-Bis-(*tert*-butoxycarbonyl)-L-cystine (449.5 mg, 1.0 mmol, 1.0 equiv.), L-serine methylester hydrochloride (396.9 mg, 2.5 mmol, 2.5 equiv.) before standard cycles of evacuation and back-filling with dry and pure N_2. Subsequently, 6 mL of anhydrous CH_2Cl_2 and *N*-methylmorphine (NMM) (333.2 uL, 3 mmol, 3 equiv.) were added and the mixture was stirred at -5 ℃ (ice-salt bath). Then, to the mixture was added a 6 mL of anhydrous CH_2Cl_2 solution of *N,N'*-dicyclohexylcarbodiimide (DCC) (579.0 mg, 2.75 mmol, 2.75 equiv.) dropwise over a period of 0.5 h. The resulting reaction mixture continued to be stirred at -5 ℃ for 2 h and thereafter was left on stirring over-night at room temperature until the reaction was complete (observed by TLC). The precipitate of the reaction was filtrated and washed with DCM (3 × 10 mL). The organic phases were combined and evaporated under reduced pressure. The residue was purified by column chromatography (Petroleum ether/ ethyl acetate from 10:8 to 10:9) on silica gel to afford the corresponding product as a white solid (482 mg, 75%), R_f = 0.38 (silica gel, PE/EA, 10:8). ^{1}H NMR (400 MHz, $CDCl_3$): δ7.74 (s, 2 H), 5.89 (s, 2 H), 4.73-4.51 (m, 4 H), 4.01-3.77 (m, 4 H), 3.68 (s, 6 H), 3.05-3.03 (m, 4 H), 1.37 (s, 18 H) ppm; ^{13}C NMR (100 MHz, $CDCl_3$): δ171.2, 170.6, 170.4, 155.7, 80.7, 62.5, 60.4, 54.8, 53.9, 52.7, 43.4, 28.3, 21.4, 14.2 ppm. HRMS (ESI) *calcd for* $C_{28}H_{46}N_4O_{10}S_2H^+$ $[M + H]^+$ *m/z* 643.2314, found: 643.2315; IR (KBr, cm^{-1}): ν_{max} 3327, 2973, 1746, 1674, 1521, 1372, 1248, 1166, 1046, 869, 735, 577; Mp: 116.2-116.6 ℃.

[Fe]/BCMOM-catalyzed alkane C-H functionalization

General Procedure A: A 25 mL flask was charged with $Fe(acac)_2$ (6.6 mg, 0.025

mmol), BCMOM (37.2 mg, 0.05 mmol), quinone (0.5 mmol), alkane (1.0 mmol) before standard cycles of evacuation and backfilling with dry and pure N_2, and then solvents CH_3CN (4 mL) and H_2O (4 mL) were added. The reaction mixture was stirred under N_2 atmosphere at room temperature for 10 min, and then H_2O_2 (129 μL, 1.5 mmol) was added dropwise into the stirring reaction mixture. Upon completion, the reaction mixture was stirred at 80 ℃ until no further changes were observed by TLC. The mixture was then allowed to get to room temperature, and extracted with ethyl acetate (3 x 10 mL). The organic phases were combined, dried (Na_2SO_4) and concentrated to give the crude product. The residue was purified by column chromatography (Petroleum ether/ ethyl acetate) on silica gel to afford the corresponding product.

***General Procedure B*:** A 25 mL flask was charged with $Fe(acac)_2$ (0.025 mmol, 6.6 mg), BCMOM (0.05 mmol, 37.2 mg), quinone (0.5 mmol), basic alkane (pre-protonation：1.0 mmol, mixed with 1.2 mmol H_2SO_4) before standard cycles of evacuation and backfilling with dry and pure N_2, and then CH_3CN (4 mL) and H_2O (4 mL) were added. The reaction mixture was stirred under N_2 atmosphere at room temperature for 10 min, and then H_2O_2 (129 μL, 1.5 mmol) was added dropwise into the stirring reaction mixture. Upon completion, the reaction mixture was stirred at 80 ℃ until no further changes were observed by TLC. After the mixture was cooled to room temperature, mixed with saturated aqueous Na_2CO_3 (10 mL), filtered through celite and then washed with ethyl acetate (3 x 10 mL). The organic phases were combined, dried (Na_2SO_4) and concentrated to give the crude product. The residue was purified by column chromatography (Petroleum ether/ ethyl acetate) on silica gel to afford the corresponding product.

General Procedure C: A 25 mL flask was charged with $FeCl_2$ (3.2 mg, 0.025 mmol), BCMOM (37.2 mg, 0.05 mmol), quinones (0.5 mmol), and alkyl amine (pre-protonation：1.0 mmol, mixed with 1.2 mmol H_2SO_4) before standard cycles of

evacuation and backfilling with dry and pure N_2, and then solvents CH_3CN (4 mL) and H_2O (4 mL) were added. The reaction mixture was stirred under N_2 atmosphere at room temperature for 10 min. Then, H_2O_2 (129 μL, 1.5 mmol) was added dropwise into the stirring reaction mixture. Upon completion, the reaction mixture was stirred at 80 ℃ until no further changes were observed by TLC. After the mixture was cooled to room temperature, 2.0 mmol of Na_2CO_3 was added and the resulting mixture was stirred overnight. The mixture was then diluted with a saturated aqueous Na_2CO_3 solution (10 mL), and extracted with ethyl acetate (3 x 10 mL). The organic phases were combined, dried (Na_2SO_4) and concentrated to give the crude product. The residue was purified by column chromatography (Petroleum ether/ ethyl acetate) on silica gel to afford the corresponding product.

General Procedure D: A 25 mL flask was charged with $FeCl_2$ (3.2 mg, 0.025 mmol), BCMOM (37.2 mg, 0.05 mmol), alkane (1.0 mmol), and azine (pre-protonation: 0.5 mmol, mixed with 1.2 mmol con. H_2SO_4) before standard cycles of evacuation and backfilling with dry and pure N_2, and then solvents CH_3CN (4 mL) and H_2O (4 mL) were added. The reaction mixture was stirred under N_2 atmosphere at room temperature for 10 min. Then, H_2O_2 (129 μL, 1.5 mmol) was added dropwise into the stirring reaction mixture. Upon completion, the reaction mixture was stirred at 80 ℃ until no further changes were observed by TLC. The mixture was then allowed to get to room temperature, diluted with a saturated aqueous Na_2CO_3 (10 mL), and extracted with ethyl acetate (3 x 10 mL). The organic phases were combined, dried (Na_2SO_4) and concentrated to give the crude product. The residue was purified by column chromatography (Petroleum ether/ ethyl acetate) on silica gel to afford the corresponding product.

Additional examples of ketones, acids, and amines tested under standard

conditions.

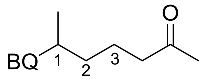

154, 70%
(C1:C2:C3=6:5.8:1)

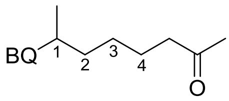

155, 42%
(C1:C2:C3:C4=4.3:3.5:1.6:0.6)

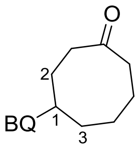

156, 45%
(C1:C2:C3=2:1:6)

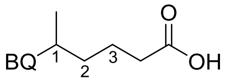

157, 70%
(C1:C2:C3=1.9:1.6:1)

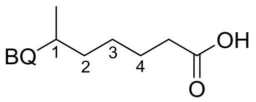

158, 72%
(C1:C2:C3:C4=1:0.8:0.7:0.5)

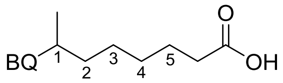

159, 75%
(C1:C2:C3:C4:C5=2.7:2.4:2.1:1.8:0.7)

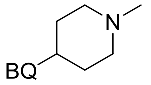

160, 80%

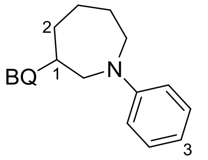

161, 85% (C1:C2:C3=3.1:6:4.3)

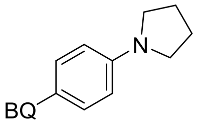

162, 76%

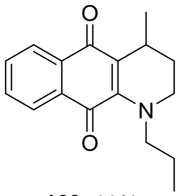

163, 69%

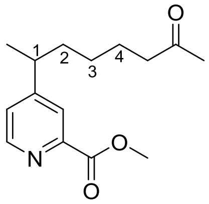

164, 47%
(C1:C2:C3:C4:C5=15:10:7:4)

Supplementary Fig. 19 Additional examples of ketones, acids, and amines tested under standard conditions.

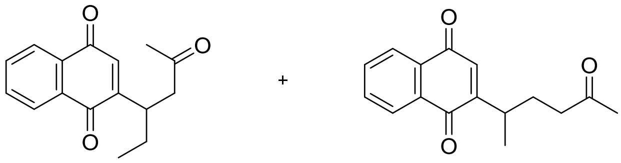

2-(5-Oxohexan-3-yl)naphthalene-1,4-dione (11-1) and 2-(5-Oxohexan-2-yl)naphthalene-1,4-dione (11-2) (11-1:11-2 = 1:2.3): *The General Procedure A* was applied with 1,4-naphthoquinone (80.6 mg, 0.5 mmol, 1 equiv.), 2-hexanone (126 μL, 1.0 mmol, 2 equiv.), $Fe(acac)_2$ (6.6 mg, 0.025 mmol, 0.05 equiv.), BCMOM (37.2 mg, 0.05 mmol, 0.1 equiv.), H_2O_2 (35%) (129 μL, 1.5 mmol, 3 equiv.), acetonitrile (4 mL) and water (4 mL) at 80 ℃ for 3 h. Column chromatography (PE/EA, 10:1) afforded the title product as a dark brown wax (106.7 mg, 83%).

TLC: R_f = 0.35 (silica gel, PE/EA, 10:1).

11-1:

^{1}H NMR (400 MHz, $CDCl_3$): δ 8.12-8.04 (m, 2 H), 7.74-7.72 (m, 2 H), 6.70 (s, 1 H), 3.44-3.37 (m, 1 H), 2.80 (d, *J* = 7.3 Hz, 2 H), 2.13 (s, 3 H), 1.64-1.58 (m, 2 H), 0.87 (t, *J* = 7.3 Hz, 3 H) ppm.

^{13}C NMR (100 MHz, $CDCl_3$): δ 206.5, 185.1, 184.7, 153.6, 134.2, 133.7, 133.7, 132.3, 131.8, 126.8, 125.9, 47.7, 35.5, 30.1, 27.1, 11.7 ppm.

HRMS (ESI) *m/z* calcd. for $C_6H_{16}O_3H^+$ [M + H^+] 257.1178, found: 257.1180.

IR (KBr, cm^{-1}): ν_{max} 2920, 2849, 1759, 1662, 1593, 1373, 1302, 1247, 1051, 926, 780, 720, 670.

11-2:

^{1}H NMR (400 MHz, $CDCl_3$): δ 8.22-7.93 (m, 2 H), 7.82-7.66 (m, 2 H), 6.76 (s, 1 H), 3.20-3.04 (m, 1 H), 2.54-2.34 (m, 2 H), 2.13 (s, 3 H), 1.89-1.74 (m, 2 H), 1.20 (d, *J* = 6.9 Hz, 3 H) ppm.

^{13}C NMR (100 MHz, $CDCl_3$): δ 208.1, 185.3, 184.7, 155.5, 133.7, 133.5, 132.3, 131.8, 126.7, 126.0, 41.3, 31.3, 30.0, 29.5, 19.5 ppm.

HRMS (ESI) *m/z* calcd. for $C_6H_{16}O_3H^+$ [M + H^+] 257.1178, found: 257.1173.

IR (KBr, cm^{-1}): ν_{max} 2920, 2849, 1759, 1662, 1593, 1373, 1302, 1247, 1051, 926, 781, 719, 668.

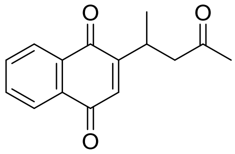

2-(4-Oxopentan-2-yl)naphthalene-1,4-dione (12): *The General Procedure A* was applied with 1,4-naphthoquinone (80.6 mg, 0.5 mmol, 1 equiv.), 2-pentanone (108 μL, 1.0 mmol, 2 equiv.), $Fe(acac)_2$ (6.6 mg, 0.025 mmol, 0.05 equiv.), BCMOM (37.2 mg, 0.05 mmol, 0.1 equiv.), H_2O_2 (35%) (129 μL, 1.5 mmol, 3 equiv.), acetonitrile (4 mL) and water (4 mL) at 80 ℃ for 1 h. Column chromatography (PE/EA, 10:1) afforded the title product as a dark brown solid (72.6 mg, 60%).

TLC: R_f = 0.30 (silica gel, PE/EA, 10:1).

^{1}H NMR (400 MHz, $CDCl_3$): δ 8.11-8.03 (m, 2 H), 7.74-7.72 (m, 2 H), 6.73 (s, 1 H), 3.62-3.54 (m, 1 H), 2.82 (dd, *J* = 17.2, 8.0 Hz, 1 H), 2.63 (dd, *J* = 17.2, 8.0 Hz, 1 H), 2.16 (s, 3 H), 1.21 (d, *J* = 6.9 Hz, 3 H) ppm.

^{13}C NMR (100 MHz, $CDCl_3$): δ 206.2, 185.2, 184.5, 154.9, 133.72, 133.70, 133.5, 132.3, 131.8, 126.7, 125.9, 49.2, 30.1, 28.6, 19.3 ppm.

HRMS (ESI) *m/z* calcd. for $C_{15}H_{14}O_3H^+$ [M + H^+] 243.1022, found: 243.1017.

IR (KBr, cm^{-1}): ν_{max} 2995, 1770, 1759, 1716, 1663, 1594, 1456, 1374, 1303, 1246, 1057, 937, 848, 780, 719, 635.

Mp: 66.6-67.5 ℃.

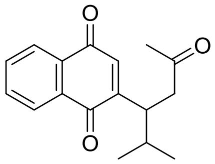

2-(2-Methyl-5-oxohexan-3-yl)naphthalene-1,4-dione (13-1)： *The General Procedure A* was applied with 1,4-naphthoquinone (80.6 mg, 0.5 mmol, 1 equiv.),

5-methyl-2-hexanone (142 μL, 1.0 mmol, 2 equiv.), $Fe(acac)_2$ (6.6 mg, 0.025 mmol, 0.05 equiv.), BCMOM (37.2 mg, 0.05 mmol, 0.1 equiv.), H_2O_2 (35%) (129 μL, 1.5 mmol, 3 equiv.), acetonitrile (4 mL) and water (4 mL) at 80 ℃ for 3 h. Column chromatography (PE/EA, 10:1) afforded the title product as a brown wax (47.3 mg, 35%).

TLC: R_f = 0.35 (silica gel, PE/EA, 10:1).

^{1}H NMR (400 MHz, $CDCl_3$): δ 8.22-7.97 (m, 2 H), 7.82-7.66 (m, 2 H), 6.65 (s, 1 H), 3.39-3.26 (m, 1 H), 2.93-2.78 (m, 2 H), 2.11 (s, 3 H), 1.91-1.83 (m, 1 H), 0.92 (d, *J* = 6.7 Hz, 3 H), 0.89 (d, *J* = 6.7 Hz, 3 H) ppm.

^{13}C NMR (100 MHz, $CDCl_3$): δ 206.7, 185.1, 184.6, 153.7, 134.1, 133.7, 133.6, 132.3, 131.8, 126.9, 125.9, 45.2, 39.6, 31.8, 30.1, 20.8, 19.9 ppm.

HRMS (ESI) *m/z* calcd. for $C_{17}H_{18}O_3H^+$ [M + H^+] 271.1335, found: 271.1334.

IR (KBr, cm^{-1}): ν_{max} 2960, 2925, 1770, 1759, 1716, 1663, 1594, 1373, 1302, 1246, 1057, 779, 719, 635.

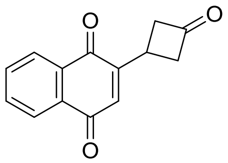

2-(3-Oxocyclobutyl)naphthalene-1,4-dione (14): *The General Procedure A* was applied with 1,4-naphthoquinone (80.6 mg, 0.5 mmol, 1 equiv.), cyclobutanone (77 μL, 1.0 mmol, 2 equiv.), $Fe(acac)_2$ (6.6 mg, 0.025 mmol, 0.05 equiv.), BCMOM (37.2 mg, 0.05 mmol, 0.1 equiv.), H_2O_2 (35%) (129 μL, 1.5 mmol, 3 equiv.), acetonitrile (4 mL) and water (4 mL) at 80 ℃ for 3 h. Column chromatography (PE/EA, 10:1) afforded the title product as a brown solid (36.2 mg, 32%).

TLC: R_f = 0.35 (silica gel, PE/EA, 10:1).

^{1}H NMR (400 MHz, $CDCl_3$): δ 8.13-8.08 (m, 2 H), 7.77 (dd, *J* = 5.6, 3.2 Hz, 2 H), 6.89 (d, *J* = 1.2 Hz, 1 H), 3.79-3.65 (m, 1 H), 3.54-3.43 (m, 2 H), 3.31-3.16 (m, 2H) ppm.

^{13}C NMR (100 MHz, $CDCl_3$) δ 204.3, 184.8, 184.7, 151.6, 134.1, 134.0, 133.7, 132.2, 131.9, 126.7, 126.3, 52.6, 24.9 ppm.

HRMS (ESI) *m/z* calcd. for $C_{14}H_{10}O_3H^+$ [M + H^+] 227.0709, found: 227.0706.

IR (KBr, cm^{-1}): ν_{max} 2995, 1770, 1759, 1662, 1374, 1302, 1246, 1057, 929, 781, 635.

Mp: 73.4-74.2 ℃.

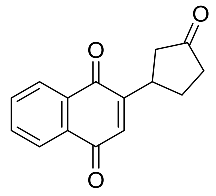

2-(3-Oxocyclopentyl)naphthalene-1,4-dione (15): *The General Procedure A* was applied with 1,4-naphthoquinone (80.6 mg, 0.5 mmol, 1 equiv.), cyclopentanone (89 μL, 1.0 mmol, 2 equiv.), $Fe(acac)_2$ (6.6 mg, 0.025 mmol, 0.05 equiv.), BCMOM (37.2 mg, 0.05 mmol, 0.1 equiv.), H_2O_2 (35%) (129 μL, 1.5 mmol, 3 equiv.), acetonitrile (4 mL) and water (4 mL) at 80 ℃ for 3 h. Column chromatography (PE/EA, 10:1) afforded the title product as a dark brown solid (84.0 mg, 70%).

TLC: R_f = 0.35 (silica gel, PE/EA, 10:1).

^{1}H NMR (400 MHz, $CDCl_3$): δ 8.12-8.06 (m, 2 H), 7.77-7.75 (m, 2 H), 6.80 (s, 1 H), 3.67-3.59 (m, 1 H), 2.68 (dd, *J* = 18.1, 7.5 Hz, 1 H), 2.51-2.33 (m, 3 H), 2.22 (dd, *J* = 18.1, 10.9 Hz, 1 H), 1.99-1.88 (m, 1 H) ppm.

^{13}C NMR (100 MHz, $CDCl_3$): δ 216.4, 184.9, 184.7, 152.0, 134.0, 133.9, 133.2, 132.2, 131.8, 126.8, 126.1, 43.5, 38.2, 36.4, 28.3 ppm.

HRMS (ESI) *m/z* calcd. for $C_{15}H_{12}O_3H^+$ [M + H^+] 241.0865, found: 241.0871.

IR (KBr, cm^{-1}): ν_{max} 2995, 1770, 1747, 1662, 1621, 1594, 1374, 1303, 1248, 1057, 782, 709, 669.

Mp: 93.5-94.7 ℃.

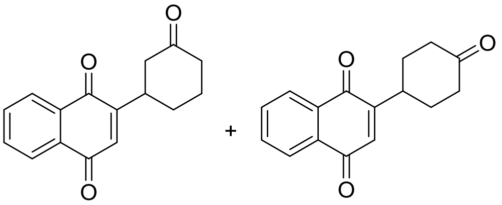

2-(3-Oxocyclohexyl)naphthalene-1,4-dione (16-1) and 2-(4-oxocyclohexyl)naphthalene-1,4-dione (16-2) (16-1:16-2 = 3:1): *The General Procedure A* was applied with 1,4-naphthoquinone (80.6 mg, 0.5 mmol, 1 equiv.), cyclohexanone (104 μL, 1.0 mmol, 2 equiv.), $Fe(acac)_2$ (6.6 mg, 0.025 mmol, 0.05 equiv.), BCMOM (37.2 mg, 0.05 mmol, 0.1 equiv.), H_2O_2 (35%) (129 μL, 1.5 mmol, 3 equiv.), acetonitrile (4 mL) and water (4 mL) at 80 ℃ for 3 h. Column chromatography (PE/EA, 10:1) afforded the title product as a brown solid (89.0 mg, 70%).

TLC: R_f = 0.35 (silica gel, PE/EA, 10:1).

^{1}H NMR (400 MHz, $CDCl_3$): δ 8.20-7.97 (m, 2H), 7.86-7.63 (m, 2H), 6.76 (s, 1H), 3.45-3.31 (m, 1H), 2.66-2.31 (m, 4H), 2.27-2.04 (m, 2H), 1.94-1.79 (m, 1H), 1.78-1.63 (m, 1H) ppm.

^{13}C NMR (100 MHz, $CDCl_3$): δ 210.0, 209.3, 185.0, 184.2, 153.5, 152.5, 133.91, 133.85, 133.7, 133.4, 132.2, 131.7, 126.81, 126.79, 126.10, 126.07, 45.7, 41.1, 40.9, 37.3, 35.1, 31.5, 30.5, 25.1 ppm.

HRMS (ESI) *m/z* calcd. for $C_{16}H_{14}O_3H^+$ [M + H^+] 255.1022, found: 255.1016.

IR (KBr, cm^{-1}): ν_{max} 2931, 2863, 1713, 1662, 1594, 1328, 1304, 1266, 941, 780 712.

Mp: 94.2-95.5 ℃.

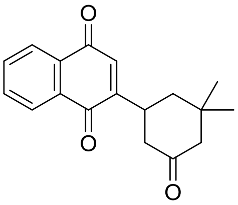

2-(3,3-Dimethyl-5-oxocyclohexyl)naphthalene-1,4-dione (17-1): *The General Procedure A* was applied with 1,4-naphthoquinone (80.6 mg, 0.5 mmol, 1 equiv.),

3,3-dimethylcyclohexanone (142 μL, 1.0 mmol, 2 equiv.), $Fe(acac)_2$ (6.6 mg, 0.025 mmol, 0.05 equiv.), BCMOM (37.2 mg, 0.05 mmol, 0.1 equiv.), H_2O_2 (35%) (129 μL, 1.5 mmol, 3 equiv.), acetonitrile (4 mL) and water (4 mL) at 80 ℃ for 3 h. Column chromatography (PE/EA, 10:1) afforded the title product as a brown solid (79.0 mg, 56%).

TLC: R_f = 0.35 (silica gel, PE/EA, 10:1).

^{1}H NMR (400 MHz, $CDCl_3$): δ 8.11-8.06 (m, 2 H), 7.78-7.74 (m, 2 H), 6.79 (s, 1 H), 3.53 (t, J = 12.8 Hz, 1 H), 2.49 (d, J = 13.1 Hz, 1 H), 2.35 (d, J = 13.1 Hz, 1 H), 2.27 (d, J = 14.2 Hz, 1 H), 1.81 (d, J = 12.5 Hz, 2 H), 1.66 (d, J = 12.5 Hz, 1 H), 1.13 (s, 3 H), 1.07 (s, 3 H) ppm.

^{13}C NMR (100 MHz, $CDCl_3$): δ 209.4, 185.0, 184.2, 152.7, 133.9, 133.6, 132.2, 131.7, 126.8, 126.1, 54.4, 44.7, 44.0, 35.4, 33.3, 32.0, 25.5 ppm.

HRMS (ESI) *m/z* calcd. for $C_{18}H_{18}O_3H^+$ [M + H^+] 283.1335, found: 283.1337.

IR (KBr, cm^{-1}): ν_{max} 2995, 2937, 1770, 1759, 1715, 1663, 1594, 1456, 1373, 1303, 1246, 1057, 778, 712, 669.

Mp: 80.5-81.9 ℃.

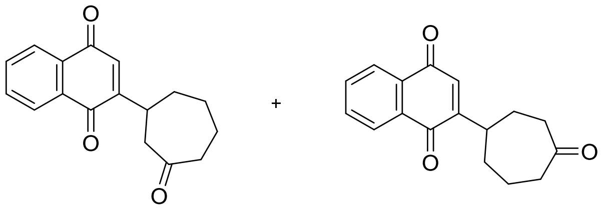

2-(3-Oxocycloheptyl)naphthalene-1,4-dione (18-1) and 2-(4-Oxocycloheptyl)naphthalene-1,4-dione (18-2) (18-1:18-2 = 1:1.3): *The General Procedure A* was applied with 1,4-naphthoquinone (80.6 mg, 0.5 mmol, 1 equiv.), cycloheptanone (120 μL, 1.0 mmol, 2 equiv.), $Fe(acac)_2$ (6.6 mg, 0.025 mmol, 0.05 equiv.), BCMOM (37.2 mg, 0.05 mmol, 0.1 equiv.), H_2O_2 (35%) (129 μL, 1.5 mmol, 3 equiv.), acetonitrile (4 mL) and water (4 mL) at 80 ℃ for 3 h. Column chromatography (PE/EA, 10:1) afforded the title product as a dark brown solid (99.2

mg, 74%).

TLC: R_f = 0.35 (silica gel, PE/EA, 10:1).

18-1:

^{1}H NMR (400 MHz, $CDCl_3$): δ 8.12-8.05 (m, 2 H), 7.74 (dd, *J* = 5.4, 3.7 Hz, 2 H), 6.73 (s, 1 H), 3.34 (t, *J* = 10.7 Hz, 1 H), 2.75 (dd, *J* = 15.0, 11.7 Hz, 1 H), 2.65 (dd, *J* = 11.2, 3.7 Hz, 1 H), 2.63-2.56 (m, 2 H), 2.11-2.00 (m, 2 H), 1.74-1.56 (m, 4 H) ppm.

^{13}C NMR (100 MHz, $CDCl_3$): δ 212.0, 185.1, 184.1, 155.0, 133.9, 133.9, 133.3, 132.2, 131.8, 126.8, 126.1, 48.4, 43.7, 36.6, 34.9, 29.3, 24.2 ppm.

HRMS (ESI) *m/z* calcd. for $C_{17}H_{16}O_3H^+$ [M + H^+] 269.1178, found: 269.1182.

IR (KBr, cm^{-1}): ν_{max} 2995, 1770, 1759, 1662, 1456, 1374, 1304, 1246, 1057, 931, 781, 626.

Mp: 54.6-55.7 ℃.

18-2:

^{1}H NMR (400 MHz, $CDCl_3$): δ 8.14-8.03 (m, 2 H), 7.76-7.73 (m, 2 H), 6.74 (s, 1 H), 3.10 (t, *J* = 11.3 Hz, 1 H), 2.77-2.69 (m, 1 H), 2.64-2.53 (m, 3 H), 2.11-2.03 (m, 2 H), 1.99-1.95 (m, 1 H), 1.85-1.71 (m, 2 H), 1.68-1.47 (m, 1 H) ppm.

^{13}C NMR (100 MHz, $CDCl_3$): δ 213.8, 185.2, 184.4, 155.3, 133.8, 133.8, 133.3, 132.2, 131.8, 126.8, 126.0, 43.6, 42.7, 40.2, 35.7, 29.7, 23.6 ppm.

HRMS (ESI) *m/z* calcd. for $C_{17}H_{16}O_3H^+$ [M + H^+] 269.1178, found: 269.1175.

IR (KBr, cm^{-1}): ν_{max} 2995, 2937, 1770, 1759, 1699, 1662, 1615, 1593, 1456, 1374, 1304, 1247, 1057, 876, 780, 715, 669.

Mp: 58.3-59.2 ℃.

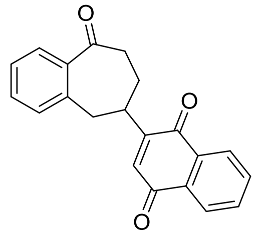

2-(9-Oxo-6,7,8,9-tetrahydro-5H-benzo[7]annulen-6-yl)naphthalene-1,4-dione

(19-1): *The General Procedure A* was applied with 1,4-naphthoquinone (80.6 mg, 0.5 mmol, 1 equiv.), 1-benzosuberone (161.8 mg, 1.0 mmol, 2 equiv.), $Fe(acac)_2$ (6.6 mg, 0.025 mmol, 0.05 equiv.), BCMOM (37.2 mg, 0.05 mmol, 0.1 equiv.), H_2O_2 (35%) (129 μL, 1.5 mmol, 3 equiv.), acetonitrile (4 mL) and water (4 mL) at 80 ℃ for 3 h. Column chromatography (PE/EA, 10:1) afforded the title product as a brown wax (98.0 mg, 62%).

TLC: R_f = 0.40 (silica gel, PE/EA, 10:1).

^{1}H NMR (400 MHz, $CDCl_3$) δ 8.17-8.05 (m, 2 H), 7.81-7.73 (m, 3 H), 7.44 (td, *J* = 7.4, 1.3 Hz, 1 H), 7.37 (t, *J* = 7.4 Hz, 1 H), 7.09 (d, *J* = 7.4 Hz, 1 H), 6.60 (s, 1 H), 3.71-3.61 (m, 1 H), 3.23-3.18 (m, 1 H), 3.02-2.94 (m, 1 H), 2.90-2.87 (m, 1H), 2.81-2.75 (m, 1 H), 2.14-2.05 (m, 1 H), 1.84-1.75 (m, 1 H) ppm.

^{13}C NMR (100 MHz, $CDCl_3$): δ 205.1, 185.0, 184.6, 153.2, 138.4, 137.6, 134.0, 133.9, 133.9, 132.6, 132.2, 131.8, 130.5, 128.8, 127.6, 126.9, 126.1, 39.5, 36.6, 34.4, 25.5 ppm.

HRMS (ESI) *m/z* calcd. for $C_{21}H_{16}O_3H^+$ [M + H^+] 317.1172, found: 317.1168.

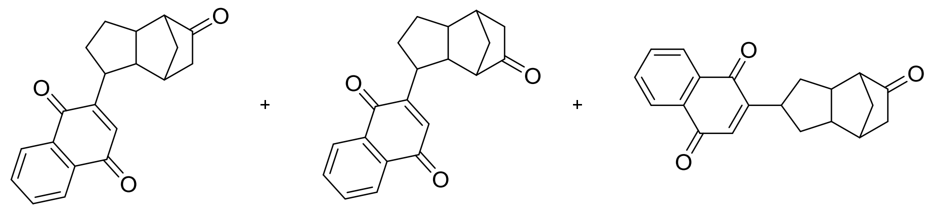

2-(5-Oxooctahydro-1H-4,7-methanoinden-1-yl)naphthalene-1,4-dione (20-3) and **2-(6-oxooctahydro-1H-4,7-methanoinden-1-yl)naphthalene-1,4-dione (20-4)** and **2-(5-oxooctahydro-1H-4,7-methanoinden-2-yl)naphthalene-1,4-dione (20-5) (20-3:20-4:20-5=1:1:3):** *The General Procedure A* was applied with 1,4-naphthoquinone (80.6 mg, 0.5 mmol, 1 equiv.), tricyclo[5.2.1.02.6]-8-decanone (154 μL, 1.0 mmol, 2 equiv.), $Fe(acac)_2$ (6.6 mg, 0.025 mmol, 0.05 equiv.), BCMOM (37.2 mg, 0.05 mmol, 0.1 equiv.), H_2O_2 (35%) (129 μL, 1.5 mmol, 3 equiv.), acetonitrile (4 mL) and water (4 mL) at 80 ℃ for 3 h. Column chromatography

(PE/EA, 10:1) afforded the title product as a light brown solid (115.3 mg, 75%).

TLC: R_f = 0.33 (silica gel, PE/EA, 10:1).

20-3 and **20-4:**

^{1}H NMR (400 MHz, $CDCl_3$): δ 8.12-8.04 (m, 4 H), 7.78-7.71 (m, 4 H), 6.88 (s, 1 H), 6.85 (s, 1 H), 3.02-2.97 (m, 2 H), 2.60 (d, *J* = 3.5 Hz, 1 H), 2.55 (s, 1 H), 2.48 (s, 1 H), 2.44 (s, 1 H), 2.43-2.38 (m, 2 H), 2.21-2.11 (m, 2 H), 2.09-1.93 (m, 8 H), 1.79-1.70 (m, 4 H), 1.69-1.62 (m, 2 H), 1.38-1.31 (m, 2 H) ppm.

^{13}C NMR (100 MHz, $CDCl_3$): δ 217.1, 217.0, 185.2, 185.2, 185.1, 185.0, 153.6, 153.0, 133.8, 133.7, 133.5, 132.3, 132.3, 131.8, 131.7, 126.7, 126.0, 54.1, 53.8, 53.3, 48.9, 47.7, 44.4, 44.1, 43.8, 42.8, 42.5, 39.5, 38.8, 34.4, 34.4, 32.2, 32.0, 31.9, 30.8 ppm.

HRMS (ESI) *m/z* calcd. for $C_{20}H_{18}O_3H^+$ [M + H^+] 307.1329, found: 307.1326.

IR (KBr, cm-1): ν_{max} 2994, 1769, 1757, 1661, 1373, 1246, 1056, 779, 713.

20-5:

^{1}H NMR (400 MHz, $CDCl_3$): δ 8.09-8.03 (m, 2 H), 7.75-7.72 (m, 2 H), 6.70 (d, *J* = 1.3 Hz, 1 H), 3.64-3.66 (m, 1 H), 2.47 (d, *J* = 3.8 Hz, 1 H), 2.42 (s, 1 H), 2.35 (t, *J* = 6.0 Hz, 2 H), 2.09-2.02 (m, 3 H), 1.90 (dd, *J* = 11.2, 3.9 Hz, 1 H), 1.82 (dd, *J* = 17.6, 4.4 Hz, 1 H), 1.80-1.70 (m, 2 H), 1.64-1.61 (m, 1 H) ppm.

^{13}C NMR (100 MHz, $CDCl_3$): δ 216.9, 185.2, 185.0, 154.1, 133.7, 132.4, 132.3, 131.8, 126.6, 126.0, 54.5, 46.2, 44.5, 41.4, 40.4, 40.2, 36.8, 36.0, 31.9 ppm.

HRMS (ESI) *m/z* calcd. for $C_{20}H_{18}O_3H^+$ [M + H^+] 307.1335, found: 307.1328.

IR (KBr, cm^{-1}): ν_{max} 2995, 1770, 1758, 1662, 1594, 1456, 1374, 1303, 1246, 1056, 931, 862, 775, 633.

Mp: 149.7-151.4 ℃.

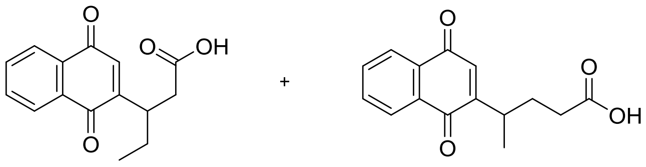

3-(1,4-Dioxo-1,4-dihydronaphthalen-2-yl)pentanoic acid (21-1) and **4-(1,4-Dioxo-1,4-dihydronaphthalen-2-yl)pentanoic acid (21-2) (21-1:21-2 = 1:2):** *The General Procedure A* was applied with 1,4-naphthoquinone (80.6 mg, 0.5 mmol, 1 equiv.), valeric acid (110 μL, 1.0 mmol, 2 equiv.), $Fe(acac)_2$ (6.6 mg, 0.025 mmol, 0.05 equiv.), BCMOM (37.2 mg, 0.05 mmol, 0.1 equiv.), H_2O_2 (35%) (129 μL, 1.5 mmol, 3 equiv.), acetonitrile (4 mL) and water (4 mL) at 80 ℃ for 1 h. Column chromatography (PE/EA/FA, 10:1.5:1) afforded the title product as a dark brown wax (97.8 mg, 75%).

TLC: R_f = 0.35 (silica gel, PE/EA/FA, 10:1.5:1).

^{1}H NMR (400 MHz, $CDCl_3$): δ 8.11-8.02 (m, 4 H), 7.74-7.70 (m, 4 H), 6.76 (s, 1 H), 6.75 (s, 1 H, minor), 3.38-3.33 (m, 1 H, minor), 3.19-3.14 (m, 1 H), 2.68 (d, *J* = 7.2 Hz, 2 H, minor), 2.35 (m, 2 H), 1.97-1.79 (m, 2 H), 1.69-1.66 (m, 2 H, minor), 1.21 (d, *J* = 6.9 Hz, 3 H), 0.89 (t, *J* = 7.4 Hz, 3 H, minor) ppm.

^{13}C NMR (100 MHz, $CDCl_3$): δ 185.2, 185.1 (minor), 184.6, 184.5 (minor), 179.0, 177.5 (minor), 155.1, 152.7 (minor), 134.6, 133.7, 133.7, 133.7, 132.3, 131.7, 126.8, 126.8, 126.0, 37.8 (minor), 36.5 (minor), 31.8, 31.5, 30.3, 26.9 (minor), 19.3, 11.6 (minor) ppm.

HRMS (ESI) *m/z* calcd. for $C_{15}H_{14}O_4H^+$ [M + H^+] 259.0971, found: 259.0969.

IR (KBr, cm^{-1}): ν_{max} 2966, 2931, 1707, 1664, 1594, 1330, 1304, 1256, 908, 780, 721, 671.

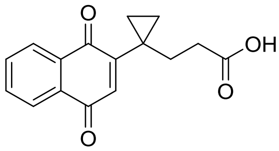

3-(1-(1,4-Dioxo-1,4-dihydronaphthalen-2-yl)cyclopropyl)propanoic acid (22): *The General Procedure A* was applied with 1,4-naphthoquinone (80.6 mg, 0.5 mmol, 1 equiv.), 3-cyclopropylpropanoic acid (104.1 μL, 1.0 mmol, 2 equiv.), $Fe(acac)_2$ (6.6 mg, 0.025 mmol, 0.05 equiv.), BCMOM (37.2 mg, 0.05 mmol, 0.1 equiv.), H_2O_2

(35%) (129 μL, 1.5 mmol, 3 equiv.), acetonitrile (4 mL) and water (4 mL) at 80 ℃ for 3 h. Column chromatography (PE/EA/FA, from 10:2:0.2 to 10:3:0.2) afforded the title product as a dark brown wax (67.5 mg, 50%).

TLC: R_f = 0.5 (silica gel, PE/EA/FA, 10:2:0.2).

^{1}H NMR (400 MHz, $CDCl_3$): δ 8.12-8.08 (m, 1 H), 8.03 (d, *J* = 6.4 Hz, 1 H), 7.72 (s, 2 H), 6.77 (s, 1 H), 2.31 (t, *J* = 6.4 Hz, 2 H), 1.98 (t, *J* = 6.4 Hz, 2 H), 0.91-0.70 (m, 4 H) ppm.

^{13}C NMR (100 MHz, $CDCl_3$): δ 185.4, 184.3, 178.3, 152.7, 137.0, 133.8, 133.6, 132.7, 131.8, 126.7, 125.9, 32.4, 32.2, 23.1, 12.6 ppm.

HRMS (ESI) *m/z* calcd. for $C_{16}H_{14}O_4H^+$ [M + H^+] 271.0965, found: 271.0966.

IR (KBr, cm^{-1}): ν_{max} 2933, 2856, 1711, 1658, 1587, 1297, 1250, 720.

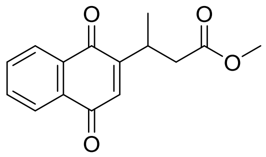

Methyl 3-(1,4-dioxo-1,4-dihydronaphthalen-2-yl)butanoate (23): *The General Procedure A* was applied with 1,4-naphthoquinone (80.6 mg, 0.5 mmol, 1 equiv.), methyl butyrate (116 μL, 1.0 mmol, 2 equiv.), $Fe(acac)_2$ (6.6 mg, 0.025 mmol, 0.05 equiv.), BCMOM (37.2 mg, 0.05 mmol, 0.1 equiv.), H_2O_2 (35%) (129 μL, 1.5 mmol, 3 equiv.), acetonitrile (4 mL) and water (4 mL) at 80 ℃ for 3 h. Column chromatography (PE/EA, 10:1.5) afforded the title product as a light brown wax (78.0 mg, 60%).

TLC: R_f = 0.5 (silica gel, PE/EA, 10:1.5).

^{1}H NMR (400 MHz, $CDCl_3$): δ 8.12-8.04 (m, 2 H), 7.74-7.72 (m, 2 H), 6.76 (d, *J* = 0.9 Hz, 1 H), 3.65 (s, 3 H), 3.58 (m, 1 H), 2.61 (m, 2 H), 1.27 (d, *J* = 7.0 Hz, 3 H) ppm.

^{13}C NMR (100 MHz, $CDCl_3$) δ 185.2, 184.4, 171.9, 154.3, 133.8, 133.7 133.6, 132.3, 131.8, 126.8, 126.0, 51.7, 39.7, 29.7, 19.4 ppm.

HRMS (ESI) *m/z* calcd. for $C_{15}H_{14}O_4H^+$ [M + H^+] 259.0965, found: 259.0971.

IR (KBr, cm^{-1}): ν_{max} 3357, 2921, 2851, 1736, 1664, 1594, 1437, 1304, 1174, 1003, 910, 780, 718.

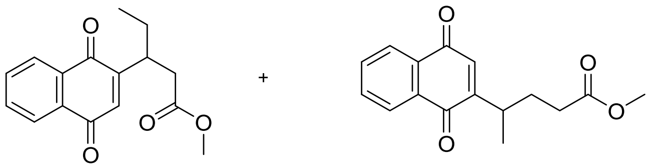

Methyl 3-(1,4-dioxo-1,4-dihydronaphthalen-2-yl)pentanoate (24-1) and **Methyl 4-(1,4-dioxo-1,4-dihydronaphthalen-2-yl)pentanoate (24-2) (24-1:24-2 = 2:3):** *The General Procedure A* was applied with 1,4-naphthoquinone (80.6 mg, 0.5 mmol, 1 equiv.), methyl valerate (134 μL, 1.0 mmol, 2 equiv.), $Fe(acac)_2$ (6.6 mg, 0.025 mmol, 0.05 equiv.), BCMOM (37.2 mg, 0.05 mmol, 0.1 equiv.), H_2O_2 (35%) (129 μL, 1.5 mmol, 3 equiv.), acetonitrile (4 mL) and water (4 mL) at 80 ℃ for 2 h. Column chromatography (PE/EA, 20:1) afforded the title product as a brown wax (95.9 mg, 70%).

TLC: R_f = 0.35 (silica gel, PE/EA, 20:1).

24-1:

^{1}H NMR (400 MHz, $CDCl_3$): δ 8.14-8.04 (m, 2 H), 7.76-7.71 (m, 2 H), 6.74 (d, *J* = 0.7 Hz, 1 H), 3.61 (s, 3 H), 3.44-3.37 (m, 1 H), 2.67-2.65 (m, 1 H), 1.71-1.64 (m, 2 H), 0.90 (t, *J* = 7.4 Hz, 3 H) ppm.

^{13}C NMR (100 MHz, $CDCl_3$): δ 185.1, 184.5, 172.1, 153.1, 134.5, 133.7, 133.7, 132.4, 131.8, 126.8, 126.0, 51.7, 38.1, 36.7, 27.0, 11.6 ppm.

HRMS (ESI) *m/z* calcd. for $C_{16}H_{16}O_4H^+$ [M + H^+] 273.1128, found: 273.1120.

IR (KBr, cm^{-1}): ν_{max} 2921, 2851, 1736, 1664, 1594, 1435, 1301, 1266, 1174, 780, 718.

24-2:

^{1}H NMR (400 MHz, $CDCl_3$): δ 8.12-8.03 (m, 2 H), 7.75-7.71 (m, 2 H), 6.76 (d, *J* = 0.8 Hz, 1 H), 3.63 (s, 3 H), 3.21-3.12 (m, 1 H), 2.36-2.30 (m, 2 H), 1.99-1.81 (m, 2 H), 1.21 (d, *J* = 6.9 Hz, 3 H) ppm.

^{13}C NMR (100 MHz, $CDCl_3$): δ 185.2, 184.6, 173.4, 155.2, 133.7, 133.7, 133.6, 132.3, 131.8, 126.7, 126.0, 51.6, 31.9, 31.6, 30.7, 19.4 ppm.

HRMS (ESI) *m/z* calcd. for $C_{16}H_{16}O_4H^+$ [M + H^+] 273.1128, found: 273.1122.

IR (KBr, cm^{-1}): ν_{max} 2952, 1736, 1664, 1594, 1437, 1330, 1304, 1256, 1174, 780, 721.

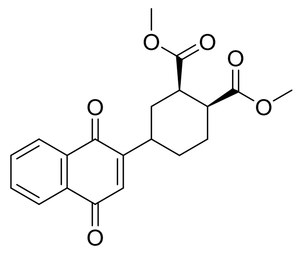

Dimethyl (1S,2R)-4-(1,4-dioxo-1,4-dihydronaphthalen-2-yl)cyclohexane-1,2-dicarboxylate (25): *The General Procedure A* was applied with 1,4-naphthoquinone (80.6 mg, 0.5 mmol, 1 equiv.), dimethyl cyclohexane-1,2-dicarboxylate (179 μL, 1.0 mmol, 2 equiv.), $Fe(acac)_2$ (6.6 mg, 0.025 mmol, 0.05 equiv.), BCMOM (37.2 mg, 0.05 mmol, 0.1 equiv.), H_2O_2 (35%) (129 μL, 1.5 mmol, 3 equiv.), acetonitrile (4 mL) and water (4 mL) at 80 ℃ for 3 h. Column chromatography (PE/EA, 10:1) afforded the title product as a yellow solid (124.6 mg, 70%).

TLC: R_f = 0.40 (silica gel, PE/EA, 10:1).

^{1}H NMR (400 MHz, $CDCl_3$): δ 8.17-7.97 (m, 1 H), 7.79-7.67 (m, 1 H), 6.73 (s, 1 H), 3.75 (s, 2 H), 3.71 (s, 1 H), 2.95 (t, *J* = 12.2 Hz, 1 H), 2.49 (dt, *J* = 12.2, 3.9 Hz, 1 H), 2.37 (d, *J* = 12.9 Hz, 1 H), 2.19 (dd, *J* = 13.9, 2.9 Hz, 1 H), 2.12-1.91 (m, 1 H), 1.64-1.59 (m, 1 H), 1.32-1.24 (m, 1 H) ppm.

^{13}C NMR (100 MHz, $CDCl_3$): δ 185.2, 184.2, 173.7, 173.2, 154.5, 133.8, 133.7, 133.3, 132.3 131.7, 126.7, 126.0, 52.0, 51.9, 43.1, 41.6, 33.1, 32.6, 30.9, 24.0 ppm.

HRMS (ESI) *m/z* calcd. for $C_{20}H_{20}O_6H^+$ [M + H^+] 357.1339, found: 357.1335.

IR (KBr, cm^{-1}): ν_{max} 2951, 1770, 1738, 1663, 1594, 1373, 1303, 1247, 1162, 1051, 940, 780, 719, 669.

Mp: 105.2-107.1 ℃.

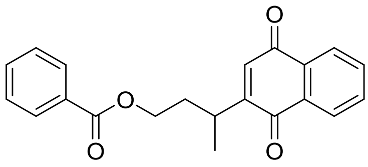

3-(1,4-Dioxo-1,4-dihydronaphthalen-2-yl)butyl benzoate (26-2): *The General Procedure A* was applied with 1,4-naphthoquinone (80.6 mg, 0.5 mmol, 1 equiv.), butyl benzoate (178 μL, 1.0 mmol, 2 equiv.), Fe(acac)$_2$ (6.6 mg, 0.025 mmol, 0.05 equiv.), BCMOM (37.2 mg, 0.05 mmol, 0.1 equiv.), H_2O_2 (35%) (129 μL, 1.5 mmol, 3 equiv.), acetonitrile (4 mL) and water (4 mL) at 80 ℃ for 3 h. Column chromatography (PE/EA, 10:2) afforded the title product as a yellow wax (93.5 mg, 56%).

TLC: R_f = 0.40 (silica gel, PE/EA, 10:1).

^{1}H NMR (400 MHz, CDCl$_3$) δ 8.09-7.99 (m, 2 H), 7.93 (dd, *J* = 8.3, 1.3 Hz, 2 H), 7.73-7.68 (m, 2 H), 7.51-7.46 (m, 1 H), 7.33 (t, *J* = 7.8 Hz, 2 H), 6.81 (d, *J* = 0.8 Hz, 1 H), 4.41-4.34 (m, 2 H), 3.40-3.34 (m, 1 H), 2.19-1.96 (m, 2 H), 1.30 (d, *J* = 6.9 Hz, 3 H) ppm.

^{13}C NMR (100 MHz, CDCl$_3$) δ 185.2, 184.6, 166.4, 155.3, 133.7, 133.7, 133.6, 132.9, 132.4, 131.8, 129.9, 129.4, 128.2, 126.7, 125.9, 62.9, 34.7, 29.9, 19.7 ppm.

HRMS (ESI) *m/z* calcd. for $C_{21}H_{18}O_4H^+$ [M + H$^+$] 335.1284, found: 335.1289.

IR (KBr, cm^{-1}): ν_{max} 2964, 2923, 1718, 1664, 1594, 1452, 1273, 1114, 1069, 778, 712.

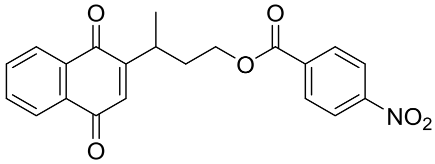

3-(1,4-Dioxo-1,4-dihydronaphthalen-2-yl)butyl 4-nitrobenzoate (27-2): *The General Procedure A* was applied with 1,4-naphthoquinone (80.6 mg, 0.5 mmol, 1 equiv.), butyl 4-nitrobenzoate (235.0 mg, 1.0 mmol, 2 equiv.), Fe(acac)$_2$ (6.6 mg,

0.025 mmol, 0.05 equiv.), BCMOM (37.2 mg, 0.05 mmol, 0.1 equiv.), H_2O_2 (35%) (129 μL, 1.5 mmol, 3 equiv.), acetonitrile (4 mL) and water (4 mL) at 80 ℃ for 3 h. Column chromatography (PE/EA, 10:2) afforded the title product as a brown wax (117.5 mg, 62%).

TLC: R_f = 0.45 (silica gel, PE/EA, 10:1).

^{1}H NMR (400 MHz, $CDCl_3$): δ 8.16 (d, *J* = 8.9 Hz, 2 H), 8.14 (d, *J* = 8.9 Hz, 2 H), 8.07-7.97 (m, 2 H), 7.73-7.66 (m, 2 H), 6.80 (s, 1 H), 4.50-4.38 (m, 2 H), 3.44-3.30 (m, 1 H), 2.21-1.97 (m, 2 H), 1.30 (d, *J* = 7.0 Hz, 3 H) ppm.

^{13}C NMR (100 MHz, $CDCl_3$): δ 185.1, 184.6, 164.5, 155.1, 150.4, 135.2, 133.8, 133.6, 132.2, 131.7, 130.5, 126.7, 125.9, 123.4, 63.9, 34.7, 29.8, 19.6 ppm.

HRMS (ESI) *m/z* calcd. for $C_{21}H_{17}NO_6H^+$ [M + H^+] 380.1129, found: 380.1126.

IR (KBr, cm^{-1}): ν_{max} 3112, 3077, 2926, 1724, 1664, 1594, 1526, 1458, 1349, 1275, 1104, 1015, 873, 782, 718, 506.

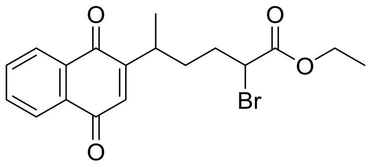

Ethyl 2-bromo-5-(1,4-dioxo-1,4-dihydronaphthalen-2-yl)hexanoate (28-3): *The General Procedure A* was applied with 1,4-naphthoquinone (80.6 mg, 0.5 mmol, 1 equiv.), ethyl 2-bromohexanoate (185 μL, 1.0 mmol, 2 equiv.), $Fe(acac)_2$ (6.6 mg, 0.025 mmol, 0.05 equiv.), BCMOM (37.2 mg, 0.05 mmol, 0.1 equiv.), H_2O_2 (35%) (129 μL, 1.5 mmol, 3 equiv.), acetonitrile (4 mL) and water (4 mL) at 80 ℃ for 3 h. Column chromatography (PE/EA, 10:1) afforded the title product as a light brown wax (134.2 mg, 71%).

TLC: R_f = 0.50 (silica gel, PE/EA, 10:1).

^{1}H NMR (400 MHz, $CDCl_3$): δ 8.12-8.04 (m, 2 H), 7.76-7.71 (m, 2 H), 6.76 (s, 1 H), 4.25-4.17 (m, 3 H), 3.18-3.15 (m, 1 H), 2.16-1.914 (m, 2 H), 1.85-1.50 (m, 1 H), 1.71-1.64 (m, 1 H), 1.28 (td, *J* = 7.1, 6.1 Hz, 3 H), 1.21 (dd, *J* = 6.9, 1.4 Hz, 3 H)

ppm.

^{13}C NMR (100 MHz, $CDCl_3$): δ 185.2, 184.6, 169.5, 155.2, 133.7, 133.64, 133.62, 132.3, 131.8, 126.7, 126.0, 62.0, 45.7, 45.5, 33.3, 32.6, 32.6, 31.6, 31.5, 19.6, 19.4, 13.9 ppm.

HRMS (ESI) *m/z* calcd. for $C_{18}H_{19}BrO_4H^+$ [M + H^+] 379.0540, found: 379.0537.

IR (KBr, cm^{-1}): ν_{max} 2966, 2933, 1736, 1662, 1594, 1458, 1330, 1301, 1256, 1153, 1025, 910, 780, 721, 671.

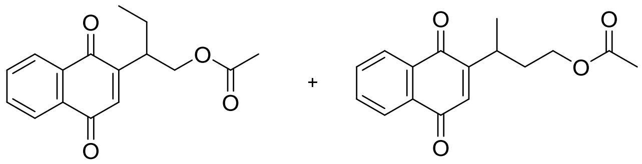

2-(1,4-Dioxo-1,4-dihydronaphthalen-2-yl)butyl acetate (29-1) and 3-(1,4-Dioxo-1,4-dihydronaphthalen-2-yl)butyl acetate (29-2) (29-1:29-2=1:4): *The General Procedure A* was applied with 1,4-naphthoquinone (80.6 mg, 0.5 mmol, 1 equiv.), butyl acetate (135 μL, 1.0 mmol, 2 equiv.), $Fe(acac)_2$ (6.6 mg, 0.025 mmol, 0.05 equiv.), BCMOM (37.2 mg, 0.05 mmol, 0.1 equiv.), H_2O_2 (35%) (129 μL, 1.5 mmol, 3 equiv.), acetonitrile (4 mL) and water (4 mL) at 80 ℃ for 3 h. Column chromatography (PE/EA, 10:1) afforded the title product as a light brown wax (99.3 mg, 73%).

TLC: R_f = 0.40 (silica gel, PE/EA, 10:1).

29-1:

^{1}H NMR (400 MHz, $CDCl_3$): δ 8.14-8.06 (m, 2 H), 7.77-7.72 (m, 2 H), 6.79 (s, 1 H), 4.31-4.19 (m, 2 H), 3.34-3.24 (m, 1 H), 1.99 (s, 3 H), 1.78-1.62 (m, 2 H), 0.94 (t, *J* = 7.4 Hz, 3 H) ppm.

^{13}C NMR (100 MHz, $CDCl_3$): δ 185.0, 184.6, 170.9, 154.1, 135.3, 133.8, 132.3, 131.8, 126.8, 126.1, 65.4, 392, 23.5, 20.8, 11.7 ppm.

HRMS (ESI) *m/z* calcd. for $C_{16}H_{16}O_4H^+$ [M + H^+] 273.1122, found: 273.1120.

IR (KBr, cm^{-1}): ν_{max} 2964, 2927, 2853, 1742, 1664, 1594, 1460, 1304, 1229, 1038,

780, 718.

29-2:

^{1}H NMR (400 MHz, $CDCl_3$): δ 8.12-8.04 (m, 2 H), 7.76-7.71 (m, 2 H), 6.77 (s, 1 H), 4.16-4.03 (m, 2 H), 3.32-3.20 (m, 1 H), 2.01-1.80 (m, 2 H), 1.95 (s, 3 H), 1.24 (d, J = 6.9 Hz, 3 H) ppm.

^{13}C NMR (100 MHz, $CDCl_3$): δ 185.2, 184.6, 170.9, 155.4, 133.7, 133.6, 132.4, 131.8, 126.7, 126.0, 62.3, 34.5, 29.6, 20.8, 19.6 ppm.

HRMS (ESI) *m/z* calcd. for $C_{16}H_{16}O_4H^+$ [M + H^+] 273.1122, found: 273.1121.

IR (KBr, cm^{-1}): ν_{max} 2966, 2929, 1738, 1664, 1594, 1458, 1301, 1242, 1046, 780, 721.

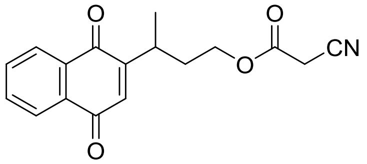

3-(1,4-Dioxo-1,4-dihydronaphthalen-2-yl)butyl 2-cyanoacetate (30-2): *The General Procedure A* was applied with 1,4-naphthoquinone (80.6 mg, 0.5 mmol, 1 equiv.), n-butyl cyanoacetate (144 mg, 1.0 mmol, 2 equiv.), $Fe(acac)_2$ (6.6 mg, 0.025 mmol, 0.05 equiv.), BCMOM (37.2 mg, 0.05 mmol, 0.1 equiv.), H_2O_2 (35%) (129 μL, 1.5 mmol, 3 equiv.), acetonitrile (4 mL) and water (4 mL) at 80 ℃ for 3 h. Column chromatography (PE/EA, 10:2) afforded the title product as a brown wax (93.6 mg, 63%).

TLC: R_f = 0.5 (silica gel, PE/EA, 10:2).

^{1}H NMR (400 MHz, $CDCl_3$): δ 8.11-8.05 (m, 2 H), 7.75 (dd, J = 5.7, 3.3 Hz, 2 H), 6.78 (s, 1 H), 4.31-4.19 (m, 2 H), 3.44 (s, 2 H), 3.31-3.22 (m, 1 H), 2.06-1.83 (m, 2 H), 1.26 (d, J = 6.9 Hz, 3 H) ppm.

^{13}C NMR (100 MHz, $CDCl_3$) δ 185.1, 184.6, 162.8, 154.7, 133.9, 133.8, 133.8, 132.2, 131.7, 126.8, 126.0, 112.8, 64.7, 34.3, 29.1, 24.6, 19.3 ppm.

HRMS (ESI) *m/z* calcd. for $C_{17}H_{15}NO_4H^+$ [M + H^+] 298.1074, found: 298.1070.

IR (KBr, cm^{-1}): ν_{max} 2966, 2925, 2851, 1748, 1662, 1594, 1460, .1390, 1256, 1118, 931, 780, 721.

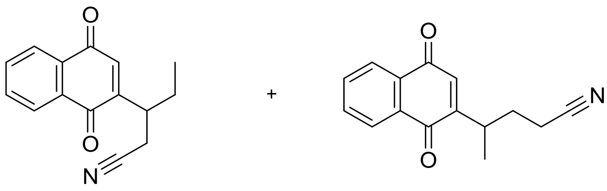

3-(1,4-Dioxo-1,4-dihydronaphthalen-2-yl)pentanenitrile (31-1) and **4-(1,4-Dioxo-1,4-dihydronaphthalen-2-yl)pentanenitrile (31-2) (31-1:31-2 = 1:2):** *The General Procedure A* was applied with 1,4-naphthoquinone (80.6 mg, 0.5 mmol, 1 equiv.), valeronitrile (107 µL, 1.0 mmol, 2 equiv.), $Fe(acac)_2$ (6.6 mg, 0.025 mmol, 0.05 equiv.), BCMOM (37.2 mg, 0.05 mmol, 0.1 equiv.), H_2O_2 (35%) (129 µL, 1.5 mmol, 3 equiv.), acetonitrile (4 mL) and water (4 mL) at 80 ℃ for 3 h. Column chromatography (PE/EA, 10:1) afforded the title product as a brown solid (84.9 mg, 70%).

TLC: R_f = 0.45 (silica gel, PE/EA, 10:1).

31-1:

^{1}H NMR (400 MHz, $CDCl_3$): δ 8.12-8.08 (m, 2 H), 7.78-7.76 (m, 2 H), 6.87 (d, *J* = 0.7 Hz, 1 H), 3.28-3.21 (m, 1 H), 2.76-2.7 (m, 2 H), 1.86-1.78 (m, 2 H), 0.97 (t, *J* = 7.4 Hz, 3 H) ppm.

^{13}C NMR (100 MHz, $CDCl_3$): δ 184.5, 184.4, 149.7, 135.7, 134.1, 134.0, 132.0, 131.7, 126.8, 126.2, 117.5, 36.8, 25.3, 21.9, 11.6 ppm.

HRMS (ESI) *m/z* calcd. for $C_{15}H_{13}NO_2H^+$ [M + H$^+$] 240.1025, found: 240.1027.

IR (KBr, cm^{-1}): ν_{max} 2922, 1770, 1664, 1594, 1457, 1424, 1374, 1330, 1303, 1247, 1051, 913, 780, 718, 669.

Mp: 65.6-67.3 ℃.

31-2:

^{1}H NMR (400 MHz, $CDCl_3$): δ 8.12-8.05 (m, 2 H), 7.77-7.73 (m, 2 H), 6.78 (s, 1 H), 3.28-3.20 (m, 1 H), 2.38 (t, *J* = 7.5 Hz, 2 H), 2.07-1.83 (m, 2 H), 1.27 (d, *J* = 7.0 Hz,

3 H) ppm.

^{13}C NMR (100 MHz, $CDCl_3$): δ 184.9, 184.5, 153.7, 134.1, 133.9, 133.9, 132.2, 131.7, 126.8, 126.1, 119.0, 31.9, 31.2, 18.9, 15.4 ppm.

HRMS (ESI) *m/z* calcd. for $C_{15}H_{13}NO_2H^+$ [M + H$^+$] 240.1025, found: 240.1024.

IR (KBr, cm^{-1}): ν_{max} 2995, 1770, 1759, 1663, 1594, 1456, 1374, 1303, 1246, 1056, 932, 781, 719, 669, 636.

Mp: 87.1-88.6 ℃.

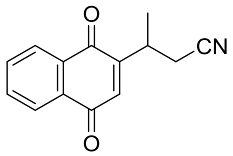

3-(1,4-Dioxo-1,4-dihydronaphthalen-2-yl)butanenitrile (32): *The General Procedure A* was applied with 1,4-naphthoquinone (80.6 mg, 0.5 mmol, 1 equiv.), butyronitrile (88 μL, 1.0 mmol, 2 equiv.), $Fe(acac)_2$ (6.6 mg, 0.025 mmol, 0.05 equiv.), BCMOM (37.2 mg, 0.05 mmol, 0.1 equiv.), H_2O_2 (35%) (129 μL, 1.5 mmol, 3 equiv.), acetonitrile (4 mL) and water (4 mL) at 80 ℃ for 3 h. Column chromatography (PE/EA, 10:1) afforded the title product as a dark brown solid (56.5 mg, 50%).

TLC: R_f = 0.45 (silica gel, PE/EA, 10:1).

^{1}H NMR (400 MHz, $CDCl_3$) δ 8.12-8.07 (m, 2 H), 7.79-7.75 (m, 2 H), 6.87 (s, 1 H), 3.53-3.45 (m, 1 H), 2.78-2.64 (m, 2 H), 1.42 (d, *J* = 7.0 Hz, 3 H) ppm.

^{13}C NMR (100 MHz, $CDCl_3$): δ 184.6, 184.3, 150.9, 134.9, 134.1, 134.0, 132.0, 131.8, 126.8, 126.2, 117.5, 29.8, 23.8, 17.9 ppm.

HRMS (ESI) *m/z* calcd. for $C_{18}H_{18}O_3H^+$ [M + H$^+$] 226.0869, found: 226.0873.

IR (KBr, cm^{-1}): ν_{max} 2929, 1770, 1665, 1621, 1593, 1507, 1330, 1304, 1252, 1060, 781, 720, 668.

Mp: 85.2-86.9 ℃.

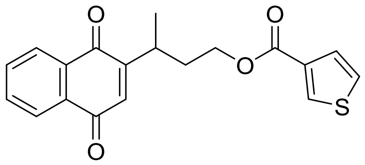

3-(1,4-Dioxo-1,4-dihydronaphthalen-2-yl)butyl thiophene-3-carboxylate (33-2): *The General Procedure A* was applied with 1,4-naphthoquinone (80.6 mg, 0.5 mmol, 1 equiv.), butyl thiophene-3-carboxylate (194.0 mg, 1.0 mmol, 2 equiv.), $Fe(acac)_2$ (6.6 mg, 0.025 mmol, 0.05 equiv.), BCMOM (37.2 mg, 0.05 mmol, 0.1 equiv.), H_2O_2 (35%) (129 μL, 1.5 mmol, 3 equiv.), acetonitrile (4 mL) and water (4 mL) at 80 ℃ for 3 h. Column chromatography (PE/EA, 10:2) afforded the title product as a brown wax (86.7 mg, 51%).

TLC: R_f = 0.45 (silica gel, PE/EA, 10:1).

^{1}H NMR (400 MHz, $CDCl_3$): δ 8.11-8.01 (m, 2 H), 7.99 (dd, *J* = 3.0, 1.1 Hz, 1 H), 7.74-7.69 (m, 2 H), 7.41 (dd, *J* = 5.1, 1.1 Hz, 1 H), 7.21 (dd, *J* = 5.1, 3.0 Hz, 1 H), 6.80 (d, *J* = 0.7 Hz, 1 H), 4.33 (t, *J* = 6.4 Hz, 2 H), 3.40-3.29 (m, 1 H), 2.16-1.90 (m, 3 H), 1.28 (d, *J* = 7.0 Hz, 3 H) ppm.

^{13}C NMR (100 MHz, $CDCl_3$): δ 185.2, 184.6, 162.5, 155.3, 133.7, 133.7, 133.6, 133.3, 132.7, 132.4, 131.8, 127.7, 126.7, 126.0, 125.9, 62.6, 34.7, 29.8, 19.6 ppm.

HRMS (ESI) *m/z* calcd. for $C_{19}H_{16}O_4SH^+$ [M + H^+] 341.0842, found: 341.0836.

IR (KBr, cm^{-1}): ν_{max} 2962, 2921, 2851, 1713, 1662, 1594, 1552, 1411, 1304, 1258, 1188, 1102, 780, 749, 718.

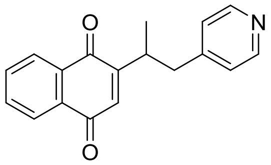

2-(1-(Pyridin-4-yl)propan-2-yl)naphthalene-1,4-dione (34): *The General Procedure B* was applied with 1,4-naphthoquinone (80.6 mg, 0.5 mmol, 1 equiv.), 4-propylpyridine (68 μL, 1.0 mmol, 2 equiv.), $Fe(acac)_2$ (6.6 mg, 0.025 mmol, 0.05 equiv.), BCMOM (37.2 mg, 0.05 mmol, 0.1 equiv.), H_2O_2 (35%) (129 μL, 1.5 mmol,

3 equiv.), acetonitrile (4 mL) and water (4 mL) at 80 ℃ for 1 h. Column chromatography (PE/EA/Et_3N, 10:3:0.1) afforded the title product as a dark brown solid (83.1 mg, 60%).

TLC: R_f = 0.55 (silica gel, PE/EA/Et_3N, 10:3:0.1).

^{1}H NMR (400 MHz, $CDCl_3$): δ 8.49 (d, *J* = 5.8 Hz, 2 H), 8.12-8.05 (m, 2 H), 7.77-7.73 (m, 2 H), 7.14 (d, *J* = 5.7 Hz, 2 H), 6.76 (s, 1 H), 3.52-3.44 (m, 1 H), 3.01-2.96 (m, 1 H), 2.66-2.61 (m, 1 H), 1.18 (d, *J* = 6.9 Hz, 3 H) ppm.

^{13}C NMR (100 MHz, $CDCl_3$): δ 185.1, 184.6, 154.5, 149.7, 148.5, 133.9, 133.9, 133.8, 132.2, 131.8, 126.8, 126.1, 124.4, 41.3, 33.3, 18.6 ppm.

HRMS (ESI) *m/z* calcd. for $C_{18}H_{15}NO_2H^+$ [M + H^+] 278.1176, found: 278.1170.

IR (KBr, cm^{-1}): ν_{max} 2921, 2851, 1662, 1594, 1415, 1330, 1304, 1252, 780, 718.

Mp: 94.1-95.9 ℃.

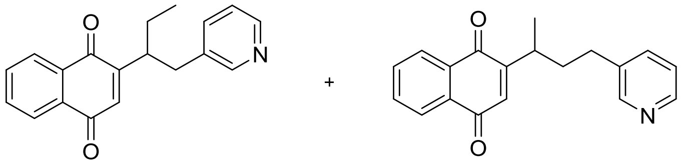

2-(1-(Pyridin-3-yl)butan-2-yl)naphthalene-1,4-dione (35-2) and 2-(4-(Pyridin-3-yl)butan-2-yl)naphthalene-1,4-dione (35-3) (35-2:35-3 = 2:1): *The General Procedure B* was applied with 1,4-naphthoquinone (80.6 mg, 0.5 mmol, 1 equiv.), 3-butylpyridine (148 μL, 1.0 mmol, 2 equiv.), $Fe(acac)_2$ (6.6 mg, 0.025 mmol, 0.05 equiv.), BCMOM (37.2 mg, 0.05 mmol, 0.1 equiv.), H_2O_2 (35%) (129 μL, 1.5 mmol, 3 equiv.), acetonitrile (4 mL) and water (4 mL) at 80 ℃ for 3 h. Column chromatography (PE/EA//Et_3N, 10:3:1) afforded the title product as a dark brown wax (94.9 mg, 65%).

TLC: R_f = 0.32 (silica gel, PE/EA/Et_3N, 10:3:0.1).

35-2:

^{1}H NMR (400 MHz, $CDCl_3$): δ 8.47-8.36 (m, 2 H), 8.08-8.02 (m, 2 H), 7.77-7.69 (m, 2 H), 7.49 (d, *J* = 7.6 Hz, 1 H), 7.23-7.14 (m, 1 H), 6.69 (s, 1 H), 3.34-3.24 (m, 1 H),

2.96-2.79 (m, 2 H), 1.75-1.59 (m, 2 H), 0.88 (t, *J* = 7.6 Hz, 3 H) ppm.

^{13}C NMR (100 MHz, $CDCl_3$): δ 184.9, 184.7, 153.0, 150.2, 147.8, 136.5, 134.9, 134.8, 133.8, 133.8, 132.2, 131.7, 126.8, 126.0, 41.2, 37.5, 26.2, 11.7 ppm.

HRMS (ESI) calcd. for $C_{19}H_{18}NO_2H^+$ $[M + H]^+$ 292.1332, found: 292.1332.

IR (KBr, cm^{-1}): ν_{max} 2968, 2933, 1664, 1599, 1333, 1310, 1250, 778, 720.

35-3:

^{1}H NMR (400 MHz, $CDCl_3$) δ 8.47-8.36 (m, 2 H), 8.13-8.08 (m, 1 H), 8.07-8.01 (m, 1 H), 7.77-7.69 (m, 2 H), 7.49 (d, *J* = 7.6 Hz, 1 H), 7.19 (dd, *J* = 7.6, 4.8 Hz, 1 H), 6.78 (s, 1 H), 3.24-3.13 (m, 1 H), 2.74-2.53 (m, 2 H), 2.02-1.90 (m, 1 H), 1.84-1.74 (m, 1 H), 1.25 (d, *J* = 6.8 Hz, 3 H) ppm.

^{13}C NMR (100 MHz, $CDCl_3$) δ 185.2, 184.7, 155.6, 149.8, 147.5, 136.8, 135.7, 133.7, 133.6, 132.3, 131.8, 126.7, 126.0, 123.3, 37.2, 31.8, 30.8, 19.4 ppm.

HRMS (ESI) *m/z* calcd for $C_{19}H_{18}NO_2H^+$ $[M + H^+]$ 292.1332, found: 292.1332.

IR (KBr, cm^{-1}): ν_{max} 2968, 2933, 1664, 1599, 1333, 1310, 1250, 778, 720.

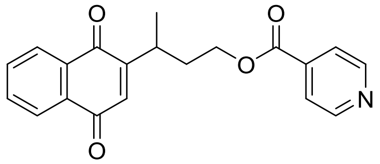

3-(1,4-Dioxo-1,4-dihydronaphthalen-2-yl)butyl thiophene-3-carboxylate (36-2): *The General Procedure B* was applied with 1,4-naphthoquinone (80.6 mg, 0.5 mmol, 1 equiv.), butyl isonicotinate (188.5 mg, 1.0 mmol, 2 equiv.), $Fe(acac)_2$ (6.6 mg, 0.025 mmol, 0.05 equiv.), BCMOM (37.2 mg, 0.05 mmol, 0.1 equiv.), H_2O_2 (35%) (129 μL, 1.5 mmol, 3 equiv.), acetonitrile (4 mL) and water (4 mL) at 80 ℃ for 1 h. Column chromatography (PE/EA/Et_3N, 10:2:0.1) afforded the title product as a dark brown solid (108.9 mg, 65%).

TLC: R_f = 0.5 (silica gel, PE/EA/Et_3N, 10:2:0.1).

¹H NMR (400 MHz, $CDCl_3$): δ 8.66 (dd, *J* = 4.4, 1.6 Hz, 2 H), 8.09-7.98 (m, 2 H), 7.75-7.67 (m, 4 H), 6.80 (d, *J* = 0.8 Hz, 1 H), 4.46-4.35 (m, 2 H), 3.41-3.30 (m, 1 H), 2.19-1.96 (m, 2 H), 1.30 (d, *J* = 7.0 Hz, 3 H) ppm.

¹³C NMR (100 MHz, $CDCl_3$): δ 185.1, 184.6, 164.9, 155.1, 150.4, 137.1, 133.8, 133.8, 133.6, 132.2, 131.7, 126.7, 125.9, 122.7, 63.8, 34.7, 29.7, 19.5 ppm.

HRMS (ESI) *m/z* calcd. for $C_{20}H_{17}NO_4H^+$ [M + H^+] 336.1231, found: 336.1229.

IR (KBr, cm^{-1}): ν_{max} 2966, 2923, 1728, 1662, 1594, 1407, 1328, 1281, 1122, 1062, 910, 780, 758, 708.

Mp: 68.9-70.2 ℃.

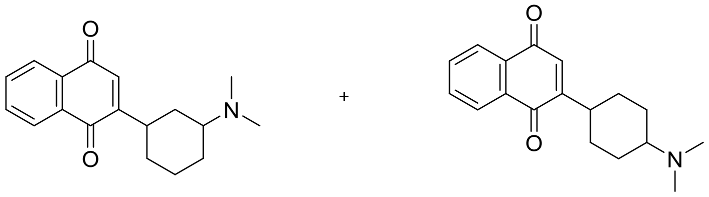

2-(3-(Dimethylamino)cyclohexyl)naphthalene-1,4-dione (37-1) and **2-(4-(Dimethylamino)cyclohexyl)naphthalene-1,4-dione (37-2) (37-1:37-2 = 3:1):** *The General Procedure B* was applied with 1,4-naphthoquinone (80.6 mg, 0.5 mmol, 1 equiv.), N,N-dimethylcyclohexylamine (153 μL, 1.0 mmol, 2 equiv.), $Fe(acac)_2$ (6.6 mg, 0.025 mmol, 0.05 equiv.), BCMOM (37.2 mg, 0.05 mmol, 0.1 equiv.), H_2O_2 (35%) (129 μL, 1.5 mmol, 3 equiv.), acetonitrile (4 mL) and water (4 mL) at 80 ℃ for 1 h. Column chromatography (PE/EA/TEA, 20:1:0.1) afforded the title product as a red brown wax (92.3 mg, 65%).

TLC: R_f = 0.50 (silica gel, PE/EA/TEA, 20:1:0.1).

37-1:

¹H NMR (400 MHz, $CDCl_3$): δ 8.10-8.03 (m, 2 H), 7.72-7.70 (m, 2 H), 6.76 (d, *J* = 1.1 Hz, 1 H), 3.43-3.35 (m, 1 H), 2.34 (s, 6 H), 2.25-2.21 (m, 1 H), 2.16-2.10 (m, 1 H), 2.02-1.96 (m, 1 H), 1.87-1.79 (m, 2 H), 1.60-1.55 (m, 1 H), 1.44-1.28 (m, 3 H) ppm.

¹³C NMR (100 MHz, $CDCl_3$): δ 185.5, 184.7, 156.3, 133.6, 133.5, 133.4, 132.6, 131.9, 126.7, 125.9, 60.9, 43.5, 35.4, 30.9, 28.9, 20.3 ppm.

HRMS (ESI) *m/z* calcd. for $C_{18}H_{21}NO_2H^+$ [M + H$^+$] 284.1645, found: 284.1640.

IR (KBr, cm^{-1}): ν_{max} 2929, 2857, 1662, 1594, 1460, 1304, 1260, 1153, 1071, 764, 721.

37-2:

^{1}H NMR (400 MHz, $CDCl_3$): δ 8.10-8.03 (m, 2 H), 7.73-7.69 (m, 2 H), 6.87 (d, *J* = 1.2 Hz, 1 H), 3.06-2.99 (m, 1 H), 2.27 (s, 6 H), 2.20-2.15 (m, 1 H), 2.05-1.97 (m, 2 H), 1.76-1.69 (m, 2 H), 1.64-1.55 (m, 4 H) ppm.

^{13}C NMR (100 MHz, $CDCl_3$): δ 185.4, 184.9, 155.4, 133.7, 133.6, 133.5, 132.5, 131.9, 126.6, 125.9, 60.6, 43.2, 35.9, 28.4, 26.0 ppm.

HRMS (ESI) *m/z* calcd. for $C_{18}H_{21}NO_2H^+$ [M + H$^+$] 284.1645, found: 284.1644.

IR (KBr, cm^{-1}): ν_{max} 2931, 2859, 1662, 1594, 1452, 1304, 1250, 1143, 1036, 941, 778, 733.

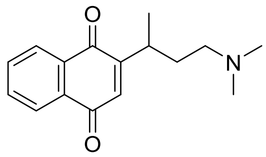

2-(4-(Dimethylamino)butan-2-yl)naphthalene-1,4-dione (38): *The General Procedure B* was applied with 1,4-naphthoquinone (80.6 mg, 0.5 mmol, 1 equiv.), N,N-dimethylaminobutane (143 μL, 1.0 mmol, 2 equiv.), $Fe(acac)_2$ (6.6 mg, 0.025 mmol, 0.05 equiv.), BCMOM (37.2 mg, 0.05 mmol, 0.1 equiv.), H_2O_2 (35%) (129 μL, 1.5 mmol, 3 equiv.), acetonitrile (4 mL) and water (4 mL) at 80 ℃ for 1 h. Column chromatography (PE/EA/Et_3N, 10:2:0.1) afforded the title product as a dark brown wax (77.6 mg, 60%).

TLC: R_f = 0.5 (silica gel, PE/EA/Et_3N, 10:2:0.1).

^{1}H NMR (400 MHz, $CDCl_3$): δ 8.09-8.05 (m, 2 H), 7.71-7.68 (m, 2 H), 6.64 (s, 1 H), 3.28-3.19 (m, 1 H), 2.36-2.15 (m, 2 H), 1.90 (s, 6 H), 1.73-1.66 (m, 2 H), 1.21 (d, *J* = 6.9 Hz, 3 H) ppm.

^{13}C NMR (100 MHz, $CDCl_3$): δ 185.5, 182.1, 157.6, 133.2, 132.9, 131.8, 129.3, 126.4, 125.7, 56.6, 43.8, 35.5, 30.7, 19.1 ppm.

HRMS (ESI) *m/z* calcd. for $C_{16}H_{19}NO_2H^+$ [M + H$^+$] 258.1489, found: 258.1482.

IR (KBr, cm^{-1}): ν_{max} 3357, 2925, 2853, 1713, 1664, 1590, 1547, 1467, 1365, 1279, 735.

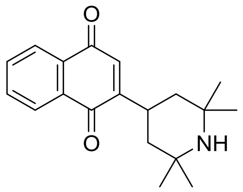

2-(2,2,6,6-Tetramethylpiperidin-4-yl)naphthalene-1,4-dione 39: *The General Procedure B* was applied with 1,4-naphthoquinone (80.6 mg, 0.5 mmol, 1 equiv.), 2,2,6,6-tetramethylpiperidine (175 μL, 1.0 mmol, 2 equiv.), $Fe(acac)_2$ (6.6 mg, 0.025 mmol, 0.05 equiv.), BCMOM (37.2 mg, 0.05 mmol, 0.1 equiv.), H_2O_2 (35%) (129 μL, 1.5 mmol, 3 equiv.), acetonitrile (4 mL) and water (4 mL) at 80 ℃ for 2 h. Column chromatography (PE/EA/TEA, 10:2:0.1) afforded the title product as a dark brown solid (111.4 mg, 75%).

TLC: R_f = 0.45 (silica gel, PE/EA/TEA, 10:2:0.1).

^{1}H NMR (400 MHz, $CDCl_3$): δ 8.11-8.05 (m, 2 H), 7.74-7.72 (m, 2 H), 6.72 (s, 1 H), 3.46 (t, *J* = 12.5 Hz, 1 H), 1.74 (dd, *J* = 12.5, 2.2 Hz, 2 H), 1.34 (s, 6 H), 1.17 (s, 6 H), 1.14-1.07 (m, 2 H) ppm.

^{13}C NMR (100 MHz, $CDCl_3$): δ 185.3, 184.7, 155.3, 133.7, 133.3, 132.4, 131.8, 126.6, 126.0, 50.9, 43.8, 34.8, 28.7, 27.9 ppm.

HRMS (ESI) *m/z* calcd. for $C_{19}H_{23}NO_2H^+$ [M + H$^+$] 298.1802, found: 298.1801.

IR (KBr, cm^{-1}): ν_{max} 2958, 2925, 1664, 1594, 1458, 1365, 1328, 1304, 1285, 1250, 1161, 974, 776, 710, 669, 580.

Mp: 67.9-69.4 ℃.

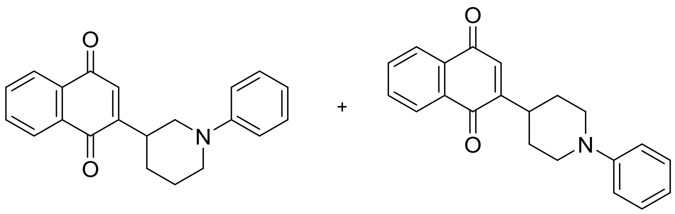

2-(1-Phenylpiperidin-3-yl)naphthalene-1,4-dione (40-1) and **2-(1-Phenylpiperidin-4-yl)naphthalene-1,4-dione (40-2) (40-1:40-2 = 1:3):** *The General Procedure B* was applied with 1,4-naphthoquinone (80.6 mg, 0.5 mmol, 1 equiv.), N-phenylpiperidine (168 μL, 1.0 mmol, 2 equiv.), $Fe(acac)_2$ (6.6 mg, 0.025 mmol, 0.05 equiv.), BCMOM (37.2 mg, 0.05 mmol, 0.1 equiv.), H_2O_2 (35%) (129 μL, 1.5 mmol, 3 equiv.), acetonitrile (4 mL) and water (4 mL) at 80 ℃ for 2 h. Column chromatography (PE/EA/TEA, 20:3:0.1) afforded the title product as a purple solid (104.3 mg, 65%).

TLC: R_f = 0.55 (silica gel, PE/EA/TEA, 20:3:0.1).

40-1:

^{1}H NMR (400 MHz, $CDCl_3$): δ 8.16-8.06 (m, 2 H), 7.77-7.72 (m, 2 H), 7.30-7.26 (m, 2 H), 7.02-6.98 (m, 2 H), 6.88 (d, *J* = 1.0 Hz, 1 H), 6.84 (t, *J* = 7.3 Hz, 1 H), 3.84-3.71 (m, 1 H), 3.75-3.71 (m, 1 H), 3.37-3.33 (m, 1 H), 2.91-2.86 (m, 1 H), 2.76 (dd, *J* = 12.1, 10.1 Hz, 1 H), 1.87-1.81 (m, 2 H), 1.62-1.52 (m, 2 H) ppm.

^{13}C NMR (100 MHz, $CDCl_3$): δ 185.2, 184.5, 152.9, 150.9, 134.3, 133.8, 132.3, 131.9, 129.2, 126.8, 126.0, 119.4, 116.6, 55.1, 50.1, 34.6, 29.4, 24.4 ppm.

HRMS (ESI) *m/z* calcd. for $C_{21}H_{19}NO_2H^+$ [M + H^+] 318.1489, found: 318.1491.

IR (KBr, cm^{-1}): ν_{max} 2923, 2851, 1662, 1596, 1497, 1330, 1304, 1250, 753, 694.

Mp: 63.1-64.2 ℃.

40-2:

^{1}H NMR (400 MHz, $CDCl_3$): δ 8.13-8.06 (m, 2 H), 7.77-7.72 (m, 2 H), 7.30-7.26 (m, 2 H), 6.98 (d, *J* = 7.9 Hz, 2 H), 6.87 (t, *J* = 7.3 Hz, 1 H), 6.79 (s, 1 H), 3.81 (d, *J* = 12.4 Hz, 2 H), 3.10-3.06 (m, 1 H), 2.88 (td, *J* = 12.3, 2.1 Hz, 2 H), 1.96 (d, *J* = 12.7 Hz, 2 H), 1.77-1.67 (m, 2 H) ppm.

^{13}C NMR (100 MHz, $CDCl_3$): δ 185.3, 184.6, 154.5, 154.5, 133.8, 133.7, 133.4, 132.3, 131.9, 129.1, 126.7, 126.0, 119.0, 116.8, 50.1, 34.9, 31.0 ppm.

HRMS (ESI) *m/z* calcd. for $C_{21}H_{19}NO_2H^+$ [M + H$^+$] 318.1489, found: 318.1489.

IR (KBr, cm^{-1}): ν_{max} 2921, 2851, 1662, 1596, 1497, 1328, 1304, 1213, 949, 760, 694.

Mp: 133.2-134.5 ℃.

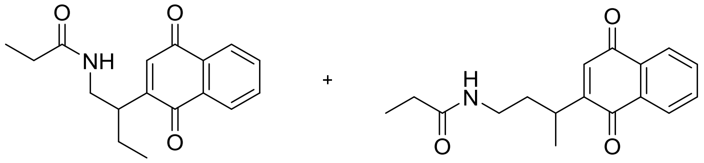

N-(2-(1,4-Dioxo-1,4-dihydronaphthalen-2-yl)butyl)propionamide (41-2) and **N-(3-(1,4-Dioxo-1,4-dihydronaphthalen-2-yl)butyl)propionamide (41-3) (41-2:41-3=2:3):** *The General Procedure A* was applied with 1,4-naphthoquinone (80.6 mg, 0.5 mmol, 1 equiv.), N-butylpropionamide (147 μL, 1.0 mmol, 2 equiv.), $Fe(acac)_2$ (6.6 mg, 0.025 mmol, 0.05 equiv.), BCMOM (37.2 mg, 0.05 mmol, 0.1 equiv.), H_2O_2 (35%) (129 μL, 1.5 mmol, 3 equiv.), acetonitrile (4 mL) and water (4 mL) at 80 ℃ for 2 h. Column chromatography (PE/EA/FA, 10:3:0.1) afforded the title product as a dark brown wax (107.3 mg, 75%).

TLC: R_f = 0.45 (silica gel, PE/EA/FA, 10:3:0.1).

41-2:

^{1}H NMR (400 MHz, $CDCl_3$): δ 8.11-8.04 (m, 2 H), 7.75-7.71 (m, 2 H), 6.75 (s, 1 H), 5.65 (s, 1 H), 3.58-3.40 (m, 2 H), 3.16-3.08 (m, 1 H), 2.11 (q, *J* = 7.6 Hz, 2 H), 1.73-1.60 (m, 2 H), 1.05 (t, *J* = 7.6 Hz, 3 H), 0.91 (t, *J* = 7.4 Hz, 3 H) ppm.

^{13}C NMR (100 MHz, $CDCl_3$): δ 185.3, 184.9, 174.0, 154.9, 135.1, 133.8, 133.8, 132.3, 131.7, 126.7, 126.0, 42.7, 40.6, 29.7, 24.2, 11.8, 9.9 ppm.

HRMS (ESI) *m/z* calcd. for $C_{17}H_{19}NO_3H^+$ [M + H$^+$] 286.1444, found: 286.1441.

IR (KBr, cm^{-1}): ν_{max} 3306, 2929, 1664, 1594, 1545, 1460, 1330, 1304, 1268, 778, 718.

41-3:

^{1}H NMR (400 MHz, $CDCl_3$): δ 8.10-8.02 (m, 2 H), 7.75-7.70 (m, 2 H), 6.78 (s, 1 H), 5.92 (s, 1 H), 3.44-3.35 (m, 2 H), 3.21-3.08 (m, 1 H), 2.22 (q, J = 7.6 Hz, 2 H), 1.73-1.70 (m, 2 H), 1.22 (d, J = 6.8 Hz, 2 H), 1.15 (t, J = 7.6 Hz, 2 H) ppm.

^{13}C NMR (100 MHz, $CDCl_3$): δ 185.2, 185.1, 174.0, 155.4, 133.8, 133.7, 132.2, 131.8, 126.7, 126.0, 37.4, 36.2, 29.7, 29.3, 19.2, 9.8 ppm.

HRMS (ESI) *m/z* calcd. for $C_{17}H_{19}NO_3H^+$ [M + H^+] 286.1444, found: 286.1442.

IR (KBr, cm^{-1}): ν_{max} 3308, 2933, 1664, 1594, 1460, 1330, 1301, 1256, 908, 778, 721.

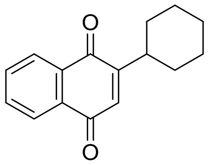

2-Cyclohexylnaphthalene-1,4-dione (42): *The General Procedure A* was applied with 1,4-naphthoquinone (80.6 mg, 0.5 mmol, 1 equiv.), cyclohexane (108 μL, 1.0 mmol, 2 equiv.), $Fe(acac)_2$ (6.6 mg, 0.025 mmol, 0.05 equiv.), BCMOM (37.2 mg, 0.05 mmol, 0.1 equiv.), H_2O_2 (35%) (129 μL, 1.5 mmol, 3 equiv.), acetonitrile (4 mL) and water (4 mL) at 80 ℃ for 3 h. Column chromatography (PE/Et_2O, 10:1) afforded the title product as a rufous solid (72.0 mg, 60%). The NMR spectroscopic data agree with those described in ref.[S5].

TLC: R_f = 0.45 (silica gel, PE/Et_2O, 10:1).

^{1}H NMR (400 MHz, $CDCl_3$) δ 8.11-8.04 (m, 2 H), 7.74-7.71 (m, 2 H), 6.73 (s, 1 H), 2.90 (t, J = 11.9 Hz, 1 H), 1.85 (d, J = 10.8 Hz, 4 H), 1.50-1.40 (m, 2 H), 1.28-1.18 (m, 4 H) ppm.

^{13}C NMR (100 MHz, $CDCl_3$) δ 185.6, 184.7, 156.2, 133.5, 133.5, 133.0, 132.4, 131.8, 126.6, 125.8, 36.6, 32.2, 26.3, 26.0 ppm.

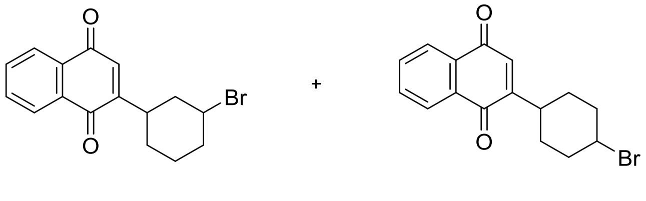

2-(3-Bromocyclohexyl)naphthalene-1,4-dione (43-1) and 2-(4-Bromocyclohexyl)naphthalene-1,4-dione (43-2) (43-1:43-2 = 5:2): *The General Procedure A* was applied with 1,4-naphthoquinone (80.6 mg, 0.5 mmol, 1 equiv.), bromocyclohexane (124 μL, 1.0 mmol, 2 equiv.), $Fe(acac)_2$ (6.6 mg, 0.025 mmol, 0.05 equiv.), BCMOM (37.2 mg, 0.05 mmol, 0.1 equiv.), H_2O_2 (35%) (129 μL, 1.5 mmol, 3 equiv.), acetonitrile (4 mL) and water (4 mL) at 80 ℃ for 3 h. Column chromatography (PE/EA, 10:1) afforded the title product as a light brown solid (63.8 mg, 40%).

TLC: R_f = 0.60 (silica gel, PE/EA, 10:1).

43-1:

^{1}H NMR (400 MHz, $CDCl_3$): δ 8.13-8.03 (m, 2 H), 7.76-7.71 (m, 2 H), 6.72 (d, *J* = 1.0 Hz, 1 H), 4.82-4.79 (m, 1 H), 3.58-3.56 (m, 1 H), 2.28-2.21 (m, 1 H), 2.14-2.01 (m, 2 H), 1.86-1.71 (m, 3 H), 1.35-1.28 (m, 2 H) ppm.

^{13}C NMR (100 MHz, $CDCl_3$): δ 185.3, 184.4, 154.7, 133.8, 133.7, 133.6, 132.4, 131.8, 126.8, 125.9, 53.0, 39.3, 34.2, 32.0, 31.4, 21.0 ppm.

HRMS (ESI) *m/z* calcd. for $C_{16}H_{15}BrO_2H^+$ [M + H^+] 319.0334, found: 319.0332.

IR (KBr, cm^{-1}): ν_{max} 2927, 2853, 1722, 1662, 1594, 1328, 1304, 1275, 1254, 943, 778, 710.

Mp: 76.2-77.5 ℃.

43-2:

^{1}H NMR (400 MHz, $CDCl_3$): δ 8.12-8.04 (m, 2 H), 7.76-7.72 (m, 2 H), 6.75 (d, *J* = 0.9 Hz, 1 H), 4.17-4.11 (m, 1 H), 3.10-3.01 (m, 1 H), 2.49-2.39 (m, 2 H), 1.96-1.90 (m, 1 H), 1.81-1.71 (m, 2 H), 1.61-1.49 (m, 1 H), 1.34-1.24 (m, 2 H) ppm.

^{13}C NMR (100 MHz, $CDCl_3$): δ 185.2, 184.3, 153.5, 133.8, 133.8, 133.4, 132.2, 131.8, 126.8, 126.0, 49.9, 43.0, 37.7, 37.4, 30.3, 26.8 ppm.

HRMS (ESI) *m/z* calcd. for $C_{16}H_{15}BrO_2H^+$ [M + H^+] 319.0334, found: 319.0333.

IR (KBr, cm^{-1}): ν_{max} 2929, 2857, 1664, 1594, 1448, 1328, 1304, 1275, 1246, 984, 937, 778, 710, 570.

Mp: 73.2-75.0 ℃.

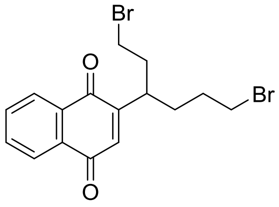

2-(1,6-Dibromohexan-3-yl)naphthalene-1,4-dione (44): *The General Procedure A* was applied with 1,4-naphthoquinone (80.6 mg, 0.5 mmol, 1 equiv.), 1,6-dibromohexane (157 μL, 1.0 mmol, 2 equiv.), $Fe(acac)_2$ (6.6 mg, 0.025 mmol, 0.05 equiv.), BCMOM (37.2 mg, 0.05 mmol, 0.1 equiv.), H_2O_2 (35%) (129 μL, 1.5 mmol, 3 equiv.), acetonitrile (4 mL) and water (4 mL) at 80 ℃ for 3 h. Column chromatography (PE/EA, 20:1) afforded the title product as a dark brown wax (60.3 mg, 30%).

TLC: R_f = 0.35 (silica gel, PE/EA, 20:1).

^{1}H NMR (400 MHz, $CDCl_3$): δ 8.13-8.05 (m, 2 H), 7.78-7.73 (m, 1 H), 6.81 (s, 1 H), 3.38 (t, *J* = 6.3 Hz, 1 H), 3.42-3.26 (m, 2 H), 3.25-3.19 (m, 1 H), 2.28-2.13 (m, 1 H), 1.87-1.76 ppm (m, 2 H).

^{13}C NMR (100 MHz, $CDCl_3$): δ 184.8, 184.7, 152.3, 135.4, 133.9, 133.9, 132.2, 131.7, 126.9, 126.1, 37.2, 37.0, 33.1, 32.6, 30.6, 30.3 ppm.

HRMS (ESI) *m/z* calcd. for $C_{16}H_{16}Br_2O_2H^+$ [M + H^+] 398.9596, found: 398.9591.

IR (KBr, cm^{-1}): ν_{max} 2930, 1663, 1614, 1594, 1456, 1329, 1302, 1253, 910, 780, 718, 669, 562.

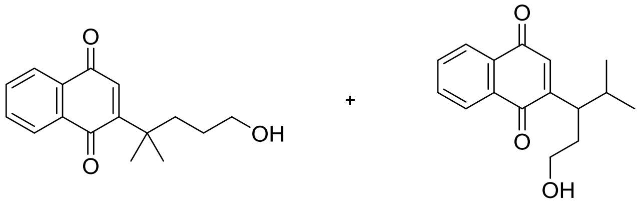

2-(5-Hydroxy-2-methylpentan-2-yl)naphthalene-1,4-dione (45-1) and 2-(1-hydroxy-4-methylpentan-3-yl)naphthalene-1,4-dione (45-2) (45-1:45-2 =

1:0.7) : *The General Procedure A* was applied with 1,4-naphthoquinone (80.6 mg, 0.5 mmol, 1 equiv.), 4-methyl-1-pentanol (127 μL, 1.0 mmol, 2 equiv.), $Fe(acac)_2$ (6.6 mg, 0.025 mmol, 0.05 equiv.), BCMOM (37.2 mg, 0.05 mmol, 0.1 equiv.), H_2O_2 (35%) (129 μL, 1.5 mmol, 3 equiv.), acetonitrile (4 mL) and water (4 mL) at 80 ℃ for 1 h. Column chromatography (PE/EA, 10:2) afforded the title product as a dark brown wax (90.7 mg, 70%), unknown compound.

TLC: R_f = 0.45 (silica gel, PE/EA, 10:2).

^{1}H NMR (400 MHz, $CDCl_3$): δ 8.02-8.11 (m, 4 H), 7.78-7.67 (m, 4 H), 6.81 (s, 1 H, minor), 6.77 (s, 1 H), 3.61-3.43 (m, 2 H), 3.57 (t, *J* = 6.8 Hz, 2 H, minor), 2.94-2.30 (m, 1 H), 2.15-2.03 (m, 1 H), 1.93-1.66 (m, 2 H), 1.90 (t, *J* = 6.8 Hz, 2 H, minor), 1.71 (t, *J* = 6.7 Hz, 2 H, minor), 1.33 (s, 6 H, minor), 0.98 (d, *J* = 6.7 Hz, 3 H), 0.87 (d, *J* = 6.7 Hz, 3 H) ppm.

^{13}C NMR (100 MHz, $CDCl_3$): δ 185.6, 185.5 (minor), 185.1, 185.0 (minor), 157.0, 154.1 (minor), 135.5, 135.0 (minor), 133.9 (minor), 133.8, 133.8 (minor), 133.5, 133.3, 132.2 (minor), 131.8 (minor), 131.5, 127.0 (minor), 127.0, 126.0 (minor), 125.7, 63.2, 60.9 (minor), 38.9 (minor), 36.9, 34.5 (minor), 32.2, 28.5 (minor), 27.9, 21.0 (minor), 20.3 ppm.

HRMS (ESI) *m/z* calcd. for $C_6H_{16}O_3H^+$ [M + H^+] 259.1335, found: 259.1335.

IR (KBr, cm^{-1}): ν_{max} 2994, 1770, 1759, 1663, 1594, 1456, 1373, 1246, 1056, 930, 779, 721, 668.

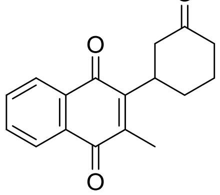

2-Methyl-3-(3-oxocyclohexyl)naphthalene-1,4-dione (46-1): *The General Procedure A* was applied with menadione (87.8 mg, 0.5 mmol, 1 equiv.), cyclohexanone (104 μL, 1.0 mmol, 2 equiv.), $Fe(acac)_2$ (6.6 mg, 0.025 mmol, 0.05

equiv.), BCMOM (37.2 mg, 0.05 mmol, 0.1 equiv.), H_2O_2 (35%) (129 μL, 1.5 mmol, 3 equiv.), acetonitrile (4 mL) and water (4 mL) at 80 ℃ for 3 h. Column chromatography (PE/EA, 10:1.5) afforded the title product as a light brown solid (76.4 mg, 57%).

TLC: R_f = 0.35 (silica gel, PE/EA, 10:1.5).

^{1}H NMR (400 MHz, $CDCl_3$): δ 8.07-8.02 (m, 2 H), 7.71-7.69 (m, 2 H), 3.29-3.18 (m, 2 H), 2.51-2.47 (m, 3 H), 2.36 (d, J = 11.0 Hz, 1 H), 2.21 (s, 3 H), 1.82-1.71 (m, 3 H) ppm.

^{13}C NMR (100 MHz, $CDCl_3$): δ 210.3, 185.1, 184.9, 146.3, 143.9, 133.6, 133.4, 132.5, 131.6, 126.2, 44.7, 41.2, 40.6, 28.6, 26.0, 12.4 ppm.

HRMS (ESI) *m/z* calcd. for $C_{17}H_{16}O_3H^+$ [M + H^+] 269.1178, found: 269.1178.

IR (KBr, cm^{-1}): ν_{max} 2994, 2950, 1770, 1759, 1714, 1661, 1594, 1373, 1293, 1246, 1054, 928, 783, 716, 668.

Mp: 105.6-107.3 ℃.

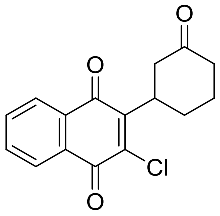

2-Chloro-3-(3-oxocyclohexyl)naphthalene-1,4-dione (47-1): *The General Procedure A* was applied with 2-chloro-1,4-naphthoquinone (99.3 mg, 0.5 mmol, 1 equiv.), cyclohexanone (104 μL, 1.0 mmol, 2 equiv.), $Fe(acac)_2$ (6.6 mg, 0.025 mmol, 0.05 equiv.), BCMOM (37.2 mg, 0.05 mmol, 0.1 equiv.), H_2O_2 (35%) (129 μL, 1.5 mmol, 3 equiv.), acetonitrile (4 mL) and water (4 mL) at 80 ℃ for 1 h. Column chromatography (PE/EA, 10:2) afforded the title product as a yellow solid (116.6 mg, 81%).

TLC: R_f = 0.35 (silica gel, PE/EA, 10:2).

^{1}H NMR (400 MHz, $CDCl_3$): δ 8.15-8.08 (m, 2 H), 7.80-7.73 (m, 2 H), 3.71-3.65 (m,

1 H), 3.25-3.19 (m, 1 H), 2.51-2.39 (m, 4 H), 2.21-2.17 (m, 1 H), 1.89-1.85 (m, 1 H), 1.83-1.73 (m, 1 H) ppm.

^{13}C NMR (100 MHz, $CDCl_3$): δ 209.3, 182.6, 177.6, 146.8, 143.7, 134.5, 134.1, 132.0, 130.8, 127.2, 127.1, 43.6, 41.1, 40.7, 30.9, 27.9, 25.6 ppm.

HRMS (ESI) *m/z* calcd. for $C_{16}H_{13}ClO_3H^+$ [M + H^+] 289.0632, found: 289.0634.

IR (KBr, cm^{-1}): ν_{max} 2928, 1770, 1714, 1679, 1661, 1593, 1282, 1245, 851, 714, 668.

Mp: 133.4-134.7 ℃.

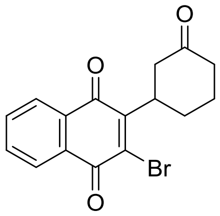

2-Bromo-3-(3-oxocyclohexyl)naphthalene-1,4-dione (48-1): *The General Procedure A* was applied with 2-bromo-1,4-naphthoquinone (122.2 mg, 0.5 mmol, 1 equiv.), cyclohexanone (104 μL, 1.0 mmol, 2 equiv.), $Fe(acac)_2$ (6.6 mg, 0.025 mmol, 0.05 equiv.), BCMOM (37.2 mg, 0.05 mmol, 0.1 equiv.), H_2O_2 (35%) (129 μL, 1.5 mmol, 3 equiv.), acetonitrile (4 mL) and water (4 mL) at 80 ℃ for 1 h. Column chromatography (PE/EA, 10:2) afforded the title product as a brown solid (131.1 mg, 79%).

TLC: R_f = 0.35 (silica gel, PE/EA, 10:2).

^{1}H NMR (400 MHz, $CDCl_3$): δ 8.14-8.07 (m, 2 H), 7.79-7.71 (m, 2 H), 3.72-3.64 (m, 1 H), 3.22 (dd, *J* = 14.0, 12.8 Hz, 1 H), 2.51-2.40 (m, 4 H), 2.20-2.16 (m, 1 H), 1.92-1.87 (m, 1 H), 1.85-1.73 (m, 1 H) ppm.

^{13}C NMR (100 MHz, $CDCl_3$): δ 209.3, 182.0, 177.6, 150.3, 140.0, 134.4, 134.0, 131.9, 130.5, 127.5, 127.1, 44.7, 43.7, 41.1, 28.0, 25.6 ppm.

HRMS (ESI) *m/z* calcd. for $C_{16}H_{13}BrO_3H^+$ [M + H^+] 333.0121, found: 333.0110.

IR (KBr, cm^{-1}): ν_{max} 2995, 1770, 1759, 1714, 1674, 1661, 1592, 1373, 1241, 1057, 790, 635.

Mp: 125.0-126.9 ℃.

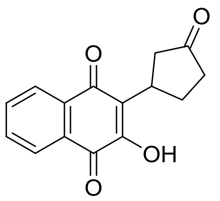

2-Hydroxy-3-(3-oxocyclopentyl)naphthalene-1,4-dione (49): *The General Procedure A* was was applied with 2-hydroxy-1,4-naphoquinone (88.9 mg, 0.5 mmol, 1 equiv.), cyclopentanone (89 μL, 1.0 mmol, 2 equiv.), Fe(acac)$_2$ (6.6 mg, 0.025 mmol, 0.05 equiv.), BCMOM (37.2 mg, 0.05 mmol, 0.1 equiv.), H_2O_2 (35%) (129 μL, 1.5 mmol, 3 equiv.), acetonitrile (4 mL) and water (4 mL) at 80 ℃ for 1 h. Column chromatography (PE/EA, 10:2) afforded the title product as a red brown solid (64.9 mg, 51%).

TLC: R_f = 0.35 (silica gel, PE/EA, 10:2).

^{1}H NMR (400 MHz, $CDCl_3$): δ 8.13 (dd, *J* = 7.7, 0.9 Hz, 1H), 8.09 (dd, *J* = 7.6, 1.0 Hz, 1H)， 7.80-7.76 (m, 1H), 7.72-7.68 (m, 1H), 3.97-3.80 (m, 1H), 2.89-2.81 (m, 1H), 2.58-2.19 (m, 5H) ppm.

^{13}C NMR (100 MHz, $CDCl_3$): δ 218.6, 184.3, 181.2, 153.21, 135.2, 133.1, 132.9, 129.1, 127.0, 126.2, 123.9, 41.6, 38.6, 32.0, 27.1 ppm.

HRMS (ESI) *m/z* calcd. for $C_{15}H_{12}O_4H^+$ [M + H$^+$] 257.0808, found: 257.0806.

IR (KBr, cm^{-1}): ν_{max} 3345, 2931, 2855, 1707, 1670, 1645, 1594, 1460, 1371, 1343, 1273, 1213, 1157, 1122, 953, 725.

Mp: 142.5-144.3 ℃.

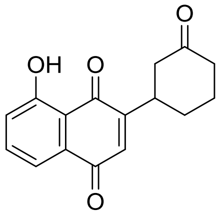

8-Hydroxy-2-(3-oxocyclohexyl)naphthalene-1,4-dione (50-1): *The General Procedure A* was applied with 5-hydroxy-1,4-naphoquinone (91.0 mg, 0.5 mmol, 1 equiv.), cyclohexanone (104 μL, 1.0 mmol, 2 equiv.), $Fe(acac)_2$ (6.6 mg, 0.025 mmol, 0.05 equiv.), BCMOM (37.2 mg, 0.05 mmol, 0.1 equiv.), H_2O_2 (35%) (129 μL, 1.5 mmol, 3 equiv.), acetonitrile (4 mL) and water (4 mL) at 80 ℃ for 1 h. Column chromatography (PE/EA, 10:2) afforded the title product as an orange solid (56.7 mg, 42%).

TLC: R_f = 0.35 (silica gel, PE/EA, 10:2).

^{1}H NMR (400 MHz, $CDCl_3$) δ 11.90 (s, 1 H), 7.67-7.60 (m, 2 H), 7.30-7.26 (m, 1 H), 6.73 (d, J = 0.7 Hz, 1 H), 3.39-3.33 (m, 1 H), 2.59-2.48 (m, 2 H), 2.44-2.32 (m, 2 H), 2.20-2.05 (m, 2 H), 1.91-1.78 (m, 1 H), 1.75-1.65 (m, 1 H) ppm.

^{13}C NMR (100 MHz, $CDCl_3$) δ 209.1, 190.1, 183.5, 161.2, 153.9, 136.4, 133.7, 132.1, 124.4, 119.6, 114.7, 45.6, 41.1, 37.4, 30.5, 25.1 ppm.

HRMS (ESI) *m/z* calcd. for $C_{16}H_{14}O_4H^+$ [M + H^+] 271.0965, found: 271.0957.

IR (KBr, cm^{-1}): ν_{max} 2923, 2853, 1701, 1643, 1452, 1318, 1248, 1201, 1170, 908, 753.

Mp: 152.0-153.3 ℃.

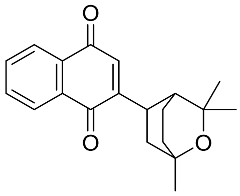

2-(1,3,3-trimethyl-2-oxabicyclo[2.2.2]octan-5-yl)naphthalene-1,4-dione (51-2): *The General Procedure A* was applied with 1,4-naphthoquinone (80.6 mg, 0.5 mmol, 1 equiv.), 1,8-cineole (169 μL, 1.0 mmol, 2 equiv.), $Fe(acac)_2$ (6.6 mg, 0.025 mmol, 0.05 equiv.), BCMOM (37.2 mg, 0.05 mmol, 0.1 equiv.), H_2O_2 (35%) (129 μL, 1.5 mmol, 3 equiv.), acetonitrile (4 mL) and water (4 mL) at 80 ℃ for 1 h. Column chromatography (PE/EA, 10:1) afforded the title product as a brown solid (94.6 mg, 61%).

TLC: R_f = 0.45 (silica gel, PE/EA/FA, 10:6:0.1).

^{1}H NMR (400 MHz, $CDCl_3$): δ 8.13-8.05 (m, 2 H), 7.78-7.71 (m, 2 H), 6.82 (d, *J* = 1.2 Hz, 1 H), 4.00-3.90 (m, 1 H), 1.96-1.90 (m, 1 H), 1.85-1.74 (m, 1 H), 1.73-1.66 (m, 1 H), 1.57-1.51 (m, 2 H), 1.49 (s, 3 H), 1.48-1.41 (m, 2 H), 1.32 (s, 3 H), 1.16 (s, 3 H) ppm.

^{13}C NMR (100 MHz, $CDCl_3$): δ 185.0, 185.0, 153.7, 134.5, 133.8, 133.7, 132.5, 131.9, 126.7, 126.0, 74.3, 70.5, 37.7, 34.8, 31.6, 31.3, 29.0, 28.6, 27.3, 16.3 ppm.

HRMS (ESI) *m/z* calcd. for $C_{20}H_{22}O_3H^+$ [M + H^+] 311.1642, found: 311.1641.

IR (KBr, cm-1): ν_{max} 2968, 2929, 1662, 1594, 1458, 1378, 1330, 1304, 1246, 984, 778, 725.

Mp: 102.3-103.5 ℃.

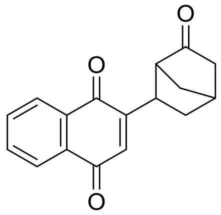

2-(6-Oxobicyclo[2.2.1]heptan-2-yl)naphthalene-1,4-dione (52-2): *The General Procedure A* was applied with 1,4-naphthoquinone (80.6 mg, 0.5 mmol, 1 equiv.), norcamphor (111.0 mg, 1.0 mmol, 2 equiv.), $Fe(acac)_2$ (6.6 mg, 0.025 mmol, 0.05 equiv.), BCMOM (37.2 mg, 0.05 mmol, 0.1 equiv.), H_2O_2 (35%) (129 μL, 1.5 mmol, 3 equiv.), acetonitrile (4 mL) and water (4 mL) at 80 ℃ for 3 h. Column chromatography (PE/EA, 10:2) afforded the title product as a yellow solid (62.5 mg, 47%).

TLC: R_f = 0.40 (silica gel, PE/EA, 10:2).

^{1}H NMR (400 MHz, $CDCl_3$): δ 8.19-8.04 (m, 2 H), 7.83-7.69 (m, 2 H), 6.79 (d, *J* = 1.4 Hz, 1 H), 3.22-3.18 (m, 1 H), 2.75-2.72 (m, 2 H), 2.27-2.20 (m, 1 H), 2.16-2.08 (m, 2 H), 1.79-1.73 (m, 3 H) ppm.

^{13}C NMR (100 MHz, $CDCl_3$): δ 216.2, 185.2, 184.9, 153.5, 133.9, 133.9, 132.3,

132.2, 131.8, 126.7, 126.1, 50.2, 45.5, 39.5, 39.0, 35.1, 30.9 ppm.

HRMS (ESI) *m/z* calcd. for $C_{17}H_{14}O_3H^+$ [M + H^+] 267.1016, found: 267.1018.

IR (KBr, cm^{-1}): ν_{max} 2962, 2921, 1746, 1662, 1594, 1330, 1304, 1250, 1124, 914, 780, 727.

Mp: 117.4-119.2 ℃.

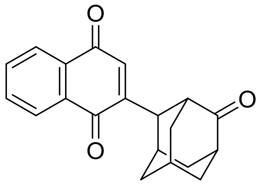

2-((1R,2R,3R,5S,7S)-4-Oxoadamantan-2-yl)naphthalene-1,4-dione (53-1): *The General Procedure A* was applied with 1,4-naphthoquinone (80.6 mg, 0.5 mmol, 1 equiv.), 2-adamantanone (154.8 mg, 1.0 mmol, 2 equiv.), $Fe(acac)_2$ (6.6 mg, 0.025 mmol, 0.05 equiv.), BCMOM (37.2 mg, 0.05 mmol, 0.1 equiv.), H_2O_2 (35%) (129 μL, 1.5 mmol, 3 equiv.), acetonitrile (4 mL) and water (4 mL) at 80 ℃ for 3 h. Column chromatography (PE/EA, 10:1) afforded the title product as a light yellow solid (58.1 mg, 38%).

TLC: R_f = 0.30 (silica gel, PE/EA, 10:1).

^{1}H NMR (400 MHz, $CDCl_3$): δ 8.07-8.03 (M, 2 H), 7.77-7.67 (m, 2 H), 6.85 (s, 1 H), 3.73 (s, 1 H), 2.73 (s, 1 H), 2.56 (s, 1 H), 2.35-2.32 (m, 1 H), 2.26 (s, 1 H), 2.24-2.22 (m, 1 H), 2.19-2.11 (m, 4 H), 2.05-2.02 (m, 1 H), 1.95-1.88 (m, 2 H) ppm.

^{13}C NMR (100 MHz, $CDCl_3$): δ 217.8, 184.8, 184.5, 152.2, 135.4, 133.8, 133.7, 132.2, 131.8, 126.6, 126.1, 49.2, 47.7, 46.5, 41.2, 40.0, 37.6, 32.9, 31.3, 27.3 ppm.

HRMS (ESI) *m/z* calcd. for $C_{20}H_{18}O_3H^+$ [M + H^+] 307.1335, found: 307.1336.

IR (KBr, cm^{-1}): ν_{max} 2923, 2856, 1769, 1718, 1662, 1615, 1594, 1455, 1374, 1330, 1304, 1252, 1054, 945, 777, 723, 669.

Mp: 143.7-145.3 ℃.

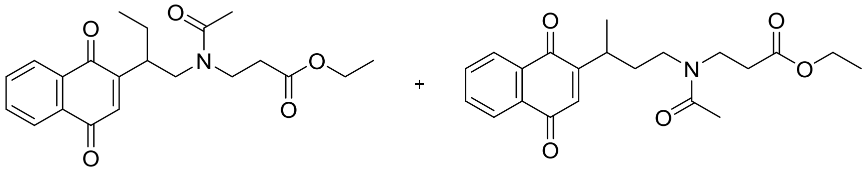

Ethyl 3-(N-(2-(1,4-dioxo-1,4-dihydronaphthalen-2-yl)butyl)acetamido)propanoate (54-1) and **Ethyl 3-(N-(3-(1,4-dioxo-1,4-dihydronaphthalen-2-yl)butyl)acetamido)propanoate (54-2) (54-1:54-2=1:1.2):** *The General Procedure A* was applied with 1,4-naphthoquinone (80.6 mg, 0.5 mmol, 1 equiv.), ethyl butylacetylaminopropionate (224 μL, 1.0 mmol, 2 equiv.), Fe(acac)$_2$ (6.6 mg, 0.025 mmol, 0.05 equiv.), BCMOM (37.2 mg, 0.05 mmol, 0.1 equiv.), H_2O_2 (35%) (129 μL, 1.5 mmol, 3 equiv.), acetonitrile (4 mL) and water (4 mL) at 80 ℃ for 3 h. Column chromatography (PE/EA/FA, 10:3:0.1) afforded the title product as a light brown wax (111.6 mg, 60%).

TLC: R_f = 0.37 (silica gel, PE/EA/FA, 10:3:0.1).

54-2:

^{1}H NMR (400 MHz, CDCl$_3$): δ 8.12-8.04 (m, 2 H), 7.77-7.71 (m, 2 H), 6.78 (d, 0.7 Hz, 1 H), 4.09 (q, *J* = 8.0 Hz, 2 H), 3.59 (t, *J* = 8.0 Hz, 2 H), 3.36 (t, *J* = 8.0 Hz, 2 H), 3.10 (m, 1 H), 2.56 (t, *J* = 8.0 Hz, 2 H), 2.06 (s, 3 H), 1.91-1.81 (m, 1 H), 1.75-1.62 (m, 1 H), 1.25 (d, *J* = 8.0 Hz, 3 H), 1.24 (t, *J* = 8.0 Hz, 3 H) ppm.

^{13}C NMR (100 MHz, CDCl$_3$): δ 185.3, 185.0, 172.1, 170.9, 154.8, 133.8, 133.7, 133.5, 132.2, 131.7, 126.7, 126.0, 60.5, 44.0, 42.2, 35.1, 32.9, 30.1, 21.3, 19.1, 14.1 ppm.

54-1:

^{1}H NMR (400 MHz, CDCl$_3$): δ 8.12-8.04 (m, 2 H), 7.77-7.71 (m, 2 H), 6.80 (d, *J* = 0.7 Hz, 1 H), 4.13 (q, *J* = 8.0 Hz, 2 H), 3.54 (t, *J* = 8.0 Hz, 2 H), 3.36 (t, *J* = 8.0 Hz, 2 H), 3.10 (m, 1 H), 2.55 (m, 2 H), 2.10 (s, 3 H), 1.92-1.81 (m, 1 H), 1.75-1.62 (m, 1 H), 1.24 (t, *J* = 8.0 Hz, 3 H), 1.22 (m, 3 H) ppm.

^{13}C NMR (100 MHz, CDCl$_3$): 184.7, 184.6, 170.5, 170.5, 155.4, 133.9, 133.6, 133.4,

132.3, 131.8, 126.6, 125.9, 60.9, 48.1, 43.4, 33.7, 33.2, 30.0, 21.4, 19.5, 14.1 ppm.

HRMS (ESI) *m/z* calcd. for $C_{21}H_{25}NO_5H^+$ [M + H^+] 372.1806, found: 372.1799.

IR (KBr, cm^{-1}): ν_{max} 2976, 2929, 1732, 1662, 1594, 1456, 1423, 1376, 1301, 1254, 1190, 1021, 782, 721.

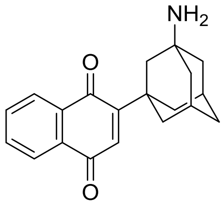

2-((1r,3s,5R,7S)-3-Aminoadamantan-1-yl)naphthalene-1,4-dione (55-1): *The General Procedure B* was applied with 1,4-naphthoquinone (80.6 mg, 0.5 mmol, 1 equiv.), 1-adamantanamine hydrochloride (189.6 mg, 1.0 mmol, 2 equiv.), $Fe(acac)_2$ (6.6 mg, 0.025 mmol, 0.05 equiv.), BCMOM (37.2 mg, 0.05 mmol, 0.1 equiv.), H_2O_2 (35%) (129 μL, 1.5 mmol, 3 equiv.), acetonitrile (4 mL) and water (4 mL) at 80 ℃ for 1 h. Column chromatography (PE/EA/TEA, 10:10:0.3) afforded the title product as a light red brown wax (86.0 mg, 56%).

TLC: R_f = 0.50 (silica gel, PE/EA/TEA, 10:10:0.3).

^{1}H NMR (400 MHz, $CDCl_3$): δ 8.07-8.01 (m, 2 H), 7.73-7.69 (m, 2 H), 6.75 (s, 1 H), 2.26 (s, 2 H), 1.97-1.89 (m, 4 H), 1.86 (s, 2 H), 1.80-1.71 (m, 2 H), 1.65 (s, 4 H) ppm.

^{13}C NMR (100 MHz, $CDCl_3$): δ 185.8, 184.7, 156.7, 134.4, 133.7, 133.6, 133.3, 131.3, 126.9, 125.6, 48.9, 48.2, 44.9, 40.4, 39.3, 35.3, 29.8 ppm.

HRMS (ESI) *m/z* calcd. for $C_{20}H_{21}NO_2H^+$ [M + H^+] 308.1645, found: 308.1633.

IR (KBr, cm^{-1}): ν_{max} 2910, 2853, 1662, 1594, 1454, 1332, 1310, 1244, 894, 776, 714, 582.

Mp: 63.5-65.7 ℃.

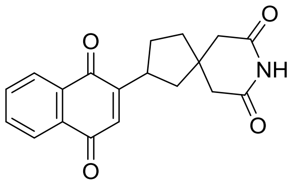

2-(1,4-Dioxo-1,4-dihydronaphthalen-2-yl)-8-azaspiro[4.5]decane-7,9-dione (56-2): *The General Procedure A* was applied with 1,4-naphthoquinone (80.6 mg, 0.5 mmol, 1 equiv.), 3,3-tetramethyleneglutarimide (172.4 mg, 1.0 mmol, 2 equiv.), Fe(acac)$_2$ (6.6 mg, 0.025 mmol, 0.05 equiv.), BCMOM (37.2 mg, 0.05 mmol, 0.1 equiv.), H_2O_2 (35%) (129 μL, 1.5 mmol, 3 equiv.), acetonitrile (4 mL) and water (4 mL) at 80 ℃ for 1 h. Column chromatography (PE/EA/FA, 10:3:0.1) afforded the title product as a light brown solid (90.4 mg, 56%).

TLC: R_f = 0.45 (silica gel, PE/EA/FA, 10:4:0.1).

^{1}H NMR (400 MHz, $CDCl_3$): δ 8.16 (br s, 1 H), 8.10-8.04 (m, 2 H), 7.77-7.72 (m, 2 H), 6.79 (s, 1 H), 3.48-3.38 (m, 1 H), 2.67 (s, 2 H), 2.64 (s, 2 H), 2.22-2.15 (m, 1 H), 2.13-2.07 (m, 1 H), 2.02-1.93 (m, 1 H), 1.86-1.78 (m, 2 H), 1.60-1.54 (m, 1 H) ppm.

^{13}C NMR (100 MHz, $CDCl_3$): δ 185.1, 184.9, 171.7, 152.9, 133.9, 133.8, 133.0, 132.3, 131.8, 126.7, 126.1, 44.7, 44.1, 43.5, 40.7, 38.5, 37.3, 30.5 ppm.

HRMS (ESI) *m/z* calcd. for $C_{19}H_{17}NO_4H^+$ [M + H$^+$] 324.1231, found: 324.1231.

IR (KBr, cm^{-1}): ν_{max} 3228, 3094, 2931, 2861, 1695, 1662, 1594, 1367, 1304, 1256, 1151, 780, 731, 587.

Mp: 174.2-175.6 ℃.

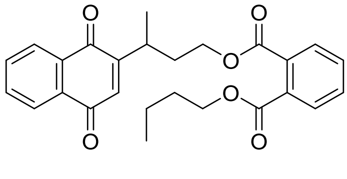

Butyl (3-(1,4-dioxo-1,4-dihydronaphthalen-2-yl)butyl) phthalate (57-2): *The General Procedure A* was applied with 1,4-naphthoquinone (80.6 mg, 0.5 mmol, 1 equiv.), dibutyl phthalate (270 μL, 1.0 mmol, 2 equiv.), Fe(acac)$_2$ (6.6 mg, 0.025 mmol, 0.05 equiv.), BCMOM (37.2 mg, 0.05 mmol, 0.1 equiv.), H_2O_2 (35%) (129 μL,

1.5 mmol, 3 equiv.), acetonitrile (4 mL) and water (4 mL) at 80 ℃ for 3 h. Column chromatography (PE/EA, 10:2) afforded the title product as a brown wax (117.2 mg, 54%).

TLC: R_f = 0.40 (silica gel, PE/EA, 10:2).

^{1}H NMR (400 MHz, $CDCl_3$): δ 8.12-7.97 (m, 2 H), 7.72-7.68 (m, 2 H), 7.68-7.59 (m, 2 H), 7.47-7.44 (m, 2 H), 6.78 (d, *J* = 0.8 Hz, 1 H), 4.42-4.31 (m, 2 H), 4.28 (t, *J* = 6.7 Hz, 2 H), 3.36-3.25 (m, 1 H), 2.14-1.91 (m, 2 H), 1.72-1.67 (m, 2 H), 1.45-1.39 (m, 2 H), 1.27 (d, *J* = 7.0 Hz, 3 H), 0.94 (t, *J* = 7.4 Hz, 3 H) ppm.

^{13}C NMR (100 MHz, $CDCl_3$): δ 185.2, 184.6, 167.6, 167.3, 155.2, 133.65, 133.6, 133.6, 132.3, 132.1, 131.8, 131.7, 131.0, 130.8, 128.9, 128.5, 126.7, 125.9, 65.6, 63.6, 34.4, 30.5, 29.8, 19.6, 19.2, 13.7 ppm.

HRMS (ESI) *m/z* calcd. for $C_{26}H_{26}O_6H^+$ [M + H^+] 435.1808, found: 435.1807.

IR (KBr, cm^{-1}): ν_{max} 2960, 2930, 2873, 1725, 1664, 1595, 1457, 1386, 1286, 1123, 1073, 939, 780, 744, 720.

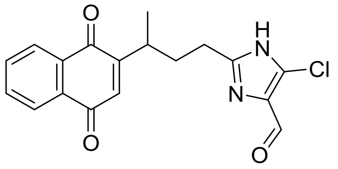

5-Chloro-2-(3-(1,4-dioxo-1,4-dihydronaphthalen-2-yl)butyl)-1H-imidazole-4-carbaldehyde (58-2): *The General Procedure B* was applied with 1,4-naphthoquinone (80.6 mg, 0.5 mmol, 1 equiv.), 2-butyl-4-chloro-5-formylimidazole (189.8 mg, 1.0 mmol, 2 equiv.), $Fe(acac)_2$ (6.6 mg, 0.025 mmol, 0.05 equiv.), BCMOM (37.2 mg, 0.05 mmol, 0.1 equiv.), H_2O_2 (35%) (129 µL, 1.5 mmol, 3 equiv.), acetonitrile (4 mL) and water (4 mL) at 80 ℃ for 1 h. Column chromatography (PE/EA, 10:3) afforded the title product as a brown wax (82.1 mg, 48%).

TLC: R_f = 0.30 (silica gel, PE/EA, 10:3).

^{1}H NMR (400 MHz, $CDCl_3$): δ 11.70 (s, 1 H), 9.50 (s, 1 H), 8.10-8.02 (m, 2 H), 7.74-7.72 (m, 2 H), 6.82 (d, *J* = 0.7 Hz, 1 H), 3.12-3.16 (m, 1 H), 2.88-2.84 (m, 2 H),

2.14-1.98 (m, 2 H), 1.25 (d, *J* = 6.9 Hz, 3 H) ppm.

^{13}C NMR (100 MHz, $CDCl_3$): δ 185.2, 185.1, 177.6, 154.7, 153.3, 141.5, 133.9, 133.9, 132.2, 131.7, 126.9, 126.0, 126.0, 33.6, 31.4, 26.6, 19.3 ppm.

HRMS (ESI) *m/z* calcd. for $C_{18}H_{15}ClN_2O_3H^+$ [M + H^+] 343.0844, found: 343.0836.

IR (KBr, cm^{-1}): ν_{max} 2927, 2853, 1664, 1594, 1507, 1388, 1330, 1304, 1256, 826, 780, 718.

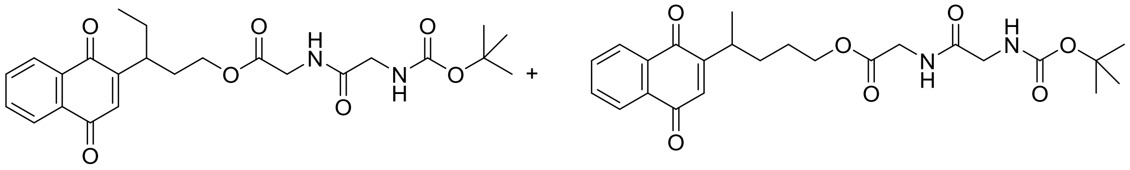

3-(1,4-Dioxo-1,4-dihydronaphthalen-2-yl)pentyl (tert-butoxycarbonyl)glycylglycinate (59-1) and 4-(1,4-dioxo-1,4-dihydronaphthalen-2-yl)pentyl (tert-butoxycarbonyl)glycylglycinate (59-2) (59-1:59-2=4:5): *The General Procedure A* was applied with 1,4-naphthoquinone (80.6 mg, 0.5 mmol, 1 equiv.), pentyl N-Boc-glycine-glycinate (305.3 mg, 1.0 mmol, 2 equiv.), $Fe(acac)_2$ (6.6 mg, 0.025 mmol, 0.05 equiv.), BCMOM (37.2 mg, 0.05 mmol, 0.1 equiv.), H_2O_2 (35%) (129 μL, 1.5 mmol, 3 equiv.), acetonitrile (4 mL) and water (4 mL) at 80 ℃ for 3 h. Column chromatography (PE/EA/FA, 10:6:0.1) afforded the title product as a yellow wax (139.8 mg, 61%).

TLC: R_f = 0.45 (silica gel, PE/EA/FA, 10:6:0.1).

59-2:

^{1}H NMR (400 MHz, $CDCl_3$): δ 8.12-8.02 (m, 2 H), 7.77-7.69 (m, 2 H), 6.75 (s, 1 H), 5.31 (s, 1 H), 4.15-4.11 (m, 2 H), 4.04 (d, *J* = 5.3 Hz, 2 H), 3.85 (s, 2 H), 3.16-3.10 (m, 1 H), 1.93-1.89 (m, 1 H), 1.71-1.58 (m, 2 H), 1.43 (s, 9 H), 1.19 (d, *J* = 6.9 Hz, 3 H) ppm.

^{13}C NMR (100 MHz, $CDCl_3$): δ185.3, 184.9, 169.7, 169.7, 169.4, 155.6, 134.6, 133.8, 133.7, 132.2, 131.8, 126.7, 125.9, 80.2, 65.1, 44.1, 41.15, 32.6, 32.0, 28.2, 26.3, 19.4

ppm.

59-1:

^{1}H NMR (400 MHz, $CDCl_3$): δ 8.12-8.02 (m, 2 H), 7.77-7.69 (m, 2 H), 6.75 (s, 1 H), 5.31 (s, 1 H), 4.15-4.11 (m, 2 H), 3.95 (d, *J* = 5.1 Hz, 2 H), 3.84 (s, 2 H), 3.10-3.03 (m, 1 H), 2.00-1.97 (m, 1 H), 1.71-1.58 (m, 2 H), 1.43 (s, 9 H), 0.86 (t, *J* = 7.4 Hz, 3 H) ppm.

^{13}C NMR (100 MHz, $CDCl_3$): δ 184.8, 184.7, 169.7, 169.7, 169.4, 153.4, 134.6, 133.8, 133.5, 132.1 131.7, 126.8, 126.0, 80.2, 63.3, 44.1, 41.1, 36.3, 31.5, 28.2, 27.2, 11.6 ppm

HRMS (ESI) *m/z* calcd. for $C_{24}H_{30}N_2O_7H^+$ [M + H^+] 459.2126, found: 459.2125.

IR (KBr, cm^{-1}): ν_{max} 3345, 2972, 2933, 1746, 1664, 1526, 1367, 1301, 1254, 1170, 1030, 943, 782, 721.

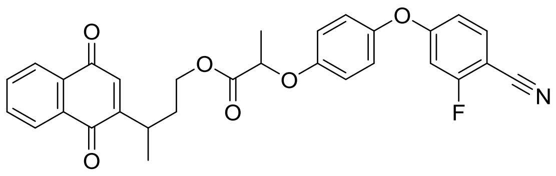

3-(1,4-Dioxo-1,4-dihydronaphthalen-2-yl)butyl 2-(4-(4-cyano-3-fluorophenoxy)phenoxy)propanoate (60-2): *The General Procedure A* was applied with 1,4-naphthoquinone (80.6 mg, 0.5 mmol, 1 equiv.), cyhalofop-butyl (372.3 mg, 1.0 mmol, 2 equiv.), $Fe(acac)_2$ (6.6 mg, 0.025 mmol, 0.05 equiv.), BCMOM (37.2 mg, 0.05 mmol, 0.1 equiv.), H_2O_2 (35%) (129 μL, 1.5 mmol, 3 equiv.), acetonitrile (4 mL) and water (4 mL) at 80 ℃ for 3 h. Column chromatography (PE/EA, 10:2) afforded the title product as a light brown wax (100.7 mg, 38%).

TLC: R_f = 0.50 (silica gel, PE/EA, 10:2).

^{1}H NMR (400 MHz, $CDCl_3$): δ 8.10-8.04 (m, 2 H), 7.76-7.72 (m, 2 H), 7.46-7.42 (m, 1 H), 7.33-7.26 (m, 1 H), 6.99 (dd, *J* = 9.0, 2.0 Hz, 2 H), 6.90-6.85 (m, 3 H), 6.75 (s, 1 H), 4.69 (m, 1 H), 4.25-4.19 (m, 2 H), 3.23-3.19 (m, 1 H), 2.01-1.93 (m, 1 H),

1.88-1.78 (m, 1 H), 1.60 (t, J = 7.2 Hz, 3 H), 1.22 (d, J = 6.9 Hz, 3 H) ppm.

^{13}C NMR (100 MHz, $CDCl_3$): δ185.1, 184.5, 171.9, 155.0, 154.9, 152.3 (d, J = 250 Hz), 150.5 (d, J = 10.5 Hz), 148.5, 148.4, 133.8, 133.7, 132.2, 131.8, 129.3 (d, J = 3.0 Hz), 126.7, 126.0, 121.1 (d, J = 3.0 Hz), 120.5 (d, J = 21 Hz), 118.5, 117.7 (d, J = 2.0 Hz), 116.5 (d, J = 4.0 Hz), 105.9 (d, J = 2.0 Hz), 72.9, 63.2, 34.4, 29.2, 19.5, 18.5 ppm.

HRMS (ESI) *m/z* calcd. for $C_{30}H_{24}FNO_6NH_4^+$ [M + NH_4^+] 531.1926, found: 531.1930.

IR (KBr, cm^{-1}): ν_{max} 3073, 2925, 2853, 2233, 1751, 1664, 1594, 1500, 1283, 1221, 1192, 1050, 1133, 945, 844, 721.

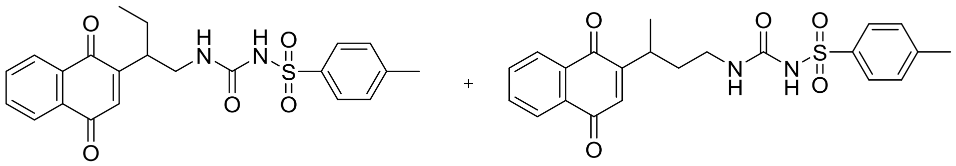

N-((2-(1,4-Dioxo-1,4-dihydronaphthalen-2-yl)butyl)carbamoyl)-4-methylbenzene sulfonamide (61-1) and **N-((3-(1,4-Dioxo-1,4-dihydronaphthalen-2-yl)butyl)carbamoyl)-4-methylbenzene sulfonamide (61-2) (61-1:61-2=1:2):** *The General Procedure A* was applied with 1,4-naphthoquinone (80.6 mg, 0.5 mmol, 1 equiv.), tolbutamide (273.1 mg, 1.0 mmol, 2 equiv.), $Fe(acac)_2$ (6.6 mg, 0.025 mmol, 0.05 equiv.), BCMOM (37.2 mg, 0.05 mmol, 0.1 equiv.), H_2O_2 (35%) (129 μL, 1.5 mmol, 3 equiv.), acetonitrile (4 mL) and water (4 mL) at 80 ℃ for 3 h. Column chromatography (PE/EA/FA, 10:3:0.1) afforded the title product as a dark brown solid (149.5 mg, 70%).

TLC: R_f = 0.35 (silica gel, PE/EA/FA, 10:3:0.1).

61-1:

^{1}H NMR (400 MHz, $CDCl_3$): δ 8.49 (br s, 1 H), 8.11-8.02 (m, 2 H), 7.76-7.72 (m, 2 H), 7.66 (d, J = 8.2 Hz, 2 H), 7.19 (d, J = 8.2 Hz, 2 H), 6.63 (d, J = 0.5 Hz, 1 H), 6.57 (t, J = 5.6 Hz, 1 H), 3.48 (t, J = 6.2 Hz, 2 H), 3.12-3.05 (m, 1 H), 2.37 (s, 3 H), 1.57

(m, 2 H), 0.86 (t, *J* = 7.4 Hz, 3 H) ppm.

^{13}C NMR (100 MHz, $CDCl_3$): δ 184.8, 184.7, 154.7, 154.2, 144.8, 136.4, 135.2, 133.8, 133.8, 132.2, 131.7, 129.9, 126.8, 126.8, 126.0, 43.0, 40.7, 24.2, 21.6, 11.6 ppm.

HRMS (ESI) *m/z* calcd. for $C_{22}H_{22}O_{25}SH^+$ [M + H^+] 427.1322, found: 427.1324.

IR (KBr, cm^{-1}): ν_{max} 3355, 2927, 1664, 1594, 1542, 1452, 1332, 1304, 1161, 1089, 885, 813, 778, 665, 587, 547.

Mp: 114.2-115.1 ℃.

61-2:

^{1}H NMR (400 MHz, $CDCl_3$): δ 8.63 (br s, 1 H), 8.12-8.09 (m, 1 H), 8.06-8.04 (m, 1 H), 7.82 (d, *J* = 8.4 Hz, 2 H), 7.76-7.71 (m, 2 H), 7.31 (d, *J* = 8.4 Hz, 2 H), 6.76 (s, 1 H), 6.70 (t, *J* = 5.6 Hz, 1 H), 3.33 (m, 1 H), 3.12 (m, 2 H), 2.41 (s, 3 H), 1.80-1.62 (m, 2 H), 1.18 (d, *J* = 6.9 Hz, 3 H) ppm.

^{13}C NMR (100 MHz, $CDCl_3$): δ 185.2, 184.9, 155.2, 154.7, 144.7, 136.6, 133.8, 133.8, 133.8, 132.2, 131.8, 129.9, 127.1, 126.8, 126.0, 38.2, 35.9, 29.4, 21.6, 19.3 ppm.

HRMS (ESI) *m/z* calcd. for $C_{22}H_{22}O_5SH^+$ [M + H^+] 427.1322, found: 427.1318.

IR (KBr, cm^{-1}): ν_{max} 3355, 2927, 1664, 1594, 1540, 1454, 1330, 1304, 1254, 1161, 1089, 894, 813, 780, 721, 665, 585, 547.

Mp: 119.6-120.7 ℃.

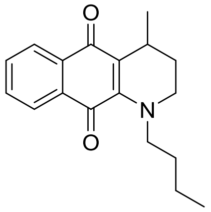

Butyl-4-methyl-1,2,3,4-tetrahydrobenzo[*g*]quinoline-5,10-dione (62): The *General Procedure C* was applied with naphthalene-1,4-dione (80.7 mg, 0.5 mmol, 1 equiv.), dibutylamine (170.1 μL, 1 mmol, 2 equiv.), H_2SO_4 (67.5 μL, 2.4 equiv.), $FeCl_2$ (3.2

mg, 0.025 mmol, 0.05 equiv.), BCMOM (37.2 mg, 0.05 mmol, 0.1 equiv.), H_2O_2 (35%) (129 μL, 1.5 mmol, 3 equiv.), acetonitrile (4 mL) and water (4 mL) at 80 ℃ for 1 h. Column chromatography (PE/EA/Et_3N, 20:1:0.1) afforded the title product as a red solid (92.4 mg, 65%).

TLC: R_f = 0.38 (silica gel, PE/EA/Et_3N, 20:1:0.1).

^{1}H NMR (400 MHz, $CDCl_3$) δ 8.01 (dd, *J* = 7.6, 0.9 Hz, 1 H), 7.89 (dd, *J* = 7.6, 0.9 Hz, 1 H), 7.62 (td, *J* = 7.5, 1.3 Hz, 1 H), 7.53 (td, *J* = 7.5, 1.3 Hz, 1 H), 3.71–3.54 (m, 2 H), 3.49–3.39 (m, 1 H), 3.33-3.27 (m, 2 H), 1.83–1.60 (m, 4 H), 1.39-1.33 (m, 2 H), 1.18 (d, *J* = 6.9 Hz, 3 H), 0.96 (t, *J* = 7.4 Hz, 3 H) ppm.

^{13}C NMR (100 MHz, $CDCl_3$) δ 183.7, 180.5, 148.1, 133.5, 133.0, 132.5, 131.4, 125.8, 125.2, 120.7, 53.9, 46.3, 31.2, 26.9, 24.5, 21.1, 20.1, 13.9 ppm.

HRMS (ESI) *m/z* calcd. for $C_{18}H_{21}NO_2H^+$ $[M + H]^+$ 284.1645, found: 284.1645.

IR (KBr, cm^{-1}): ν_{max} 3361, 2953, 2924, 2866, 1668, 1615, 1593, 1549, 1361, 1274, 1214, 723.

Mp: 89.7-90.3 ℃.

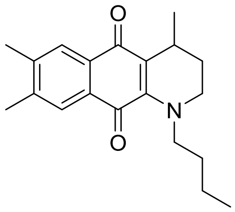

1-Butyl-4,7,8-trimethyl-1,2,3,4-tetrahydrobenzo[*g*]quinoline-5,10-dione (63): The *General Procedure C* was applied with 6,7-dimethylnaphthalene-1,4-dione (97.9 mg, 0.5 mmol, 1 equiv.), dibutylamine (170.1 μL, 1.0 mmol, 2 equiv.), H_2SO_4 (67.5 μL, 2.4 equiv.), $FeCl_2$ (3.2 mg, 0.025 mmol, 0.05 equiv.), BCMOM (37.2 mg, 0.05 mmol, 0.1 equiv.), H_2O_2 (35%) (129 μL, 1.5 mmol, 3 equiv.), acetonitrile (4 mL) and water (4 mL) at 80 ℃ for 1 h. Column chromatography (PE/EA/Et_3N, 20:1:0.1) afforded the title product as a red solid (105.8 mg, 68%).

TLC: R_f = 0.50 (silica gel, PE/EA/Et_3N, 20:1:0.1).

^{1}H NMR (400 MHz, $CDCl_3$): δ 7.74 (s, 1 H), 7.64 (s, 1 H), 3.77–3.50 (m, 2 H), 3.42-3.38 (m, 1 H), 3.37–3.15 (m, 2 H), 2.33 (d, *J* = 6.5 Hz, 6 H), 1.83–1.59 (m, 4 H), 1.35 (m, 2 H), 1.16 (d, *J* = 6.9 Hz, 3 H), 0.95 (t, *J* = 7.4 Hz, 3 H) ppm.

^{13}C NMR (100 MHz, $CDCl_3$): δ 183.8, 181.0, 148.1, 143.1, 140.6, 131.0, 130.4, 127.0, 126.4, 120.5, 53.9, 46.2, 31.2, 26.9, 24.4, 21.1, 20.14, 20.1, 19.8, 13.9 ppm.

HRMS (ESI) *m/z* calcd. for $C_{20}H_{25}NO_2H^+$ $[M + H]^+$ 312.1958, found: 312.1962.

IR (KBr, cm^{-1}): ν_{max} 2957, 2927, 2860, 1670, 1599, 1546, 1357, 1286, 1195, 746.

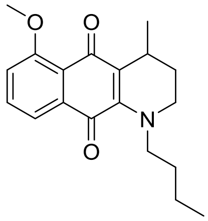

1-Butyl-6-methoxy-4-methyl-1,2,3,4-tetrahydrobenzo[*g*]quinoline-5,10-dione (64): The *General Procedure C* was applied with 5-methoxynaphthalene-1,4-dione (99.0 mg, 0.5 mmol, 1 equiv.), dibutylamine (170.1 µL, 1.0 mmol, 2 equiv.), H_2SO_4 (67.5 µL, 2.4 equiv.), $FeCl_2$ (3.2 mg, 0.025 mmol, 0.05 equiv.), BCMOM (37.2 mg, 0.05 mmol, 0.1 equiv.), H_2O_2 (35%) (129 µL, 1.5 mmol, 3 equiv.), acetonitrile (4 mL) and water (4 mL) at 80 ℃ for 1 h. Column chromatography (PE/EA/Et_3N, from 40:1:0.1 to 10:1:0.1) afforded the title product as a red solid (75.2 mg, 48%).

TLC: R_f = 0.45 (silica gel, PE/EA/Et_3N, 10:1:0.1).

^{1}H NMR (400 MHz, $CDCl_3$): δ 7.54 (d, *J* = 7.5 Hz, 1 H), 7.47 (t, *J* = 8.0 Hz, 1 H), 7.19 (d, *J* = 8.3 Hz, 1 H), 3.95 (s, 3 H), 3.68-3.43 (m, 2 H), 3.40–3.26 (m, 2 H), 3.22 (m, 1 H), 1.81–1.53 (m, 4 H), 1.32 (m, 2 H), 1.15 (d, *J* = 6.9 Hz, 3H), 0.93 (t, *J* = 7.3 Hz, 3 H) ppm.

^{13}C NMR (100 MHz, $CDCl_3$): δ 183.8, 181.2, 158.4, 146.9, 135.2, 132.4, 122.6, 120.3, 118.8, 117.8, 56.4, 53.3, 45.6, 31.1, 27.0, 24.3, 21.2, 20.1, 13.9 ppm.

HRMS (ESI) *m/z* calcd. for $C_{19}H_{23}NO_3H^+$ $[M + H]^+$ 314.1751, found: 314.1747.

IR (KBr, cm^{-1}): ν_{max} 2956, 2928, 2861, 1668, 1621, 1592, 1554, 1266, 1213, 1013,

757.

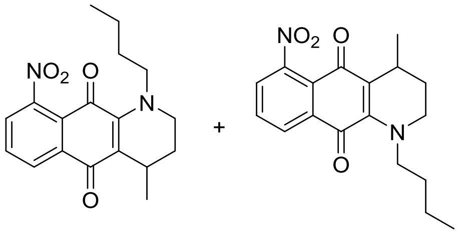

1-Butyl-4-methyl-9-nitro-1,2,3,4-tetrahydrobenzo[*g*]quinoline-5,10-dione (65-1) and **1-Butyl-4-methyl-6-nitro-1,2,3,4-tetrahydrobenzo[*g*]quinoline-5,10-dione (65-2) (65-1:65-2 = 1.6:1):** The *General Procedure C* was applied with 5-nitronaphthalene-1,4-dione (112.8 mg, 0.5 mmol, 1 equiv.), dibutylamine (170.1 μL, 1.0 mmol, 2 equiv.), H_2SO_4 (67.5 μL, 2.4 equiv.), $FeCl_2$ (3.2 mg, 0.025 mmol, 0.05 equiv.), BCMOM (37.2 mg, 0.05 mmol, 0.1 equiv.), H_2O_2 (35%) (129 μL, 1.5 mmol, 3 equiv.), acetonitrile (4 mL) and water (4 mL) at 80 ℃ for 1 h. Column chromatography (PE/EA/Et_3N, 20:1:0.1) afforded a 1.6:1 mixture of oxidation products as a purplish red solid (98.4 mg, 60%).

TLC: R_f = 0.50 (silica gel, PE/EA/Et_3N, 20:1:0.1).

^{1}H NMR (400 MHz, $CDCl_3$): δ 8.21 (dd, *J* = 5.1, 3.9 Hz, 1 H, minor), 8.05 (dd, *J* = 7.7, 1.1 Hz, 1 H), 7.83–7.45 (m, 2 H), 3.70–3.38 (m, 3 H), 3.34–3.20 (m, 2 H), 1.85–1.58 (m, 4 H), 1.35 (m, 2 H), 1.17-1.15 (m, 3 H), 0.98-0.95 (m, 3 H) ppm.

^{13}C NMR (100 MHz, $CDCl_3$): δ 181.4 (minor), 181.3, 177.4 (minor), 176.2, 150.1, 147.8 (minor), 147.5, 134.7, 133.4 (minor), 133.2, 131.9 (minor), 128.6 (minor), 128.1, 127.0, 125.8, 124.4 (minor), 121.1, 119.2 (minor), 54.0, 53.5 (minor), 46.5, 45.5 (minor), 31.3 (minor), 31.2, 26.7, 24.5, 24.3 (minor), 20.9, 20.1, 13.9, 13.8 (minor) ppm.

HRMS (ESI) *m/z* calcd. for $C_{18}H_{20}N_2O_4H^+$ $[M + H]^+$ 329.1496, found: 329.1499.

IR (KBr, cm^{-1}): ν_{max} 2959, 2929, 2868, 1678, 1544, 1365, 1269, 1214, 1118, 712.

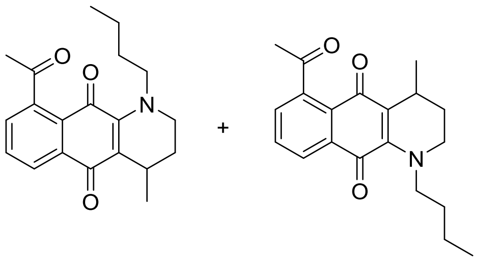

9-Acetyl-1-butyl-4-methyl-1,2,3,4-tetrahydrobenzo[*g*]quinoline-5,10-dione (66-1) and **6-Acetyl-1-butyl-4-methyl-1,2,3,4-tetrahydrobenzo[*g*]quinoline-5,10-dione (66-2) (66-1:66-2 = 1.1:1):** The *General Procedure C* was applied with 5-acetylnaphthalene-1,4-dione (111.1 mg, 0.5 mmol, 1 equiv.), dibutylamine (170.1 μL, 1.0 mmol, 2 equiv.), H_2SO_4 (67.5 μL, 2.4 equiv.), $FeCl_2$ (3.2 mg, 0.025 mmol, 0.05 equiv.), BCMOM (37.2 mg, 0.05 mmol, 0.1 equiv.), H_2O_2 (35%) (129 μL, 1.5 mmol, 3 equiv.), acetonitrile (4 mL) and water (4 mL) at 80 ℃ for 1 h. Column chromatography (PE/EA/Et_3N, 15:1:0.1) afforded a 1.1:1 mixture of products as a purplish red solid (102.4 mg, 63%).

TLC: R_f = 0.40 (silica gel, PE/EA/Et_3N, 15:1:0.1).

^{1}H NMR (400 MHz, $CDCl_3$): δ 8.10 (d, *J* = 7.7 Hz, 1 H), 7.96 (d, *J* = 7.7 Hz, 1 H, minor), 7.66 (t, *J* = 7.6 Hz, 1 H), 7.58 (t, *J* = 7.6 Hz, 1 H, minor), 7.39 (d, *J* = 1.7 Hz, 1 H), 7.37 (d, *J* = 1.3 Hz, 1 H, minor), 3.74–3.43 (m, 3 H), 3.40–3.22 (m, 2 H), 2.55 (s, 3 H), 2.53 (s, 3 H, minor), 1.84–1.62 (m, 4 H), 1.37 (m, 2 H), 1.18 (d, *J* = 6.8 Hz, 3 H), 1.00-0.95 (m, 3 H) ppm.

^{13}C NMR (100 MHz, $CDCl_3$): δ 205.3, 204.1, 183.9, 182.8 (minor), 179.7 (minor), 179.4, 148.7, 147.9 (minor), 142.3, 142.2 (minor), 133.7, 133.1, 132.6, 131.5, 130.6, 130.2, 130.1, 128.4, 126.7, 126.5, 120.2 (minor), 120.0, 53.9 (minor), 53.6, 46.4 (minor), 46.0, 31.2, 30.5, 26.8 (minor), 26.7, 24.4, 21.0, 20.1, 13.9 (minor), 13.8 ppm.

HRMS (ESI) *m/z* calcd. for $C_{20}H_{23}NO_3H^+$ $[M + H]^+$ 326.1751, found: 326.1756.

IR (KBr, cm^{-1}): ν_{max} 2957, 2929, 2870, 1703, 1669, 1613, 1547, 1361, 1256, 1210, 1110.

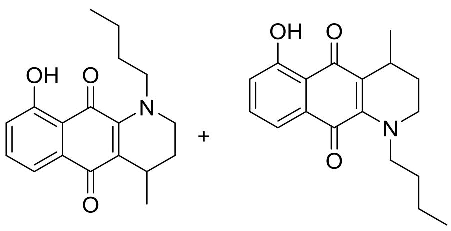

1-Butyl-9-hydroxy-4-methyl-1,2,3,4-tetrahydrobenzo[*g*]quinoline-5,10-dione (67-1) and **1-Butyl-6-hydroxy-4-methyl-1,2,3,4-tetrahydrobenzo[*g*]quinoline-5,10-dione (67-2) (67-1:67-2 = 5:3):** The *General Procedure C* was applied with 5-hydroxynaphthalene-1,4-dione (91.7 mg, 0.5 mmol, 1 equiv.), dibutylamine (170.1 μL, 1.0 mmol, 2 equiv.), H_2SO_4 (67.5 μL, 2.4 equiv.), $FeCl_2$ (3.2 mg, 0.025 mmol, 0.05 equiv.), BCMOM (37.2 mg, 0.05 mmol, 0.1 equiv.), H_2O_2 (35%) (129 μL, 1.5 mmol, 3 equiv.), acetonitrile (4 mL) and water (4 mL) at 80 ℃ for 1 h. Column chromatography (PE/EA/Et_3N, from 20:1:0.1 to 10:1:0.1) afforded the title product as a dark red solid (74.8 mg, 50%) and a red solid (46.4 mg, 31%).

TLC: R_f = 0.55 and 0.60 (silica gel, PE/EA/Et_3N, 10:1:0.1).

67-1:

^{1}H NMR (400 MHz, $CDCl_3$): δ 11.84 (s, 1 H), 7.70–7.45 (m, 2 H), 7.07 (dd, *J* = 8.1, 1.2 Hz, 1 H), 3.76–3.57 (m, 2 H), 3.48–3.38 (m, 1 H), 3.38–3.16 (m, 2 H), 1.83–1.59 (m, 4 H), 1.43–1.32 (m, 2 H), 1.16 (d, *J* = 6.9 Hz, 3 H), 0.97 (t, *J* = 7.4 Hz, 3 H) ppm.

^{13}C NMR (100 MHz, $CDCl_3$): δ 188.1, 180.0, 161.0, 146.8, 136.3, 133.2, 122.3, 121.9, 117.7, 115.1, 54.5, 46.9, 31.2, 26.8, 24.6, 21.0, 20.2, 13.9 ppm.

HRMS (ESI) *m/z* calcd. for $C_{18}H_{21}NO_3H^+$ $[M + H]^+$ 300.1594, found: 300.1596.

IR (KBr, cm^{-1}): ν_{max} 2958, 2926, 2861, 1620, 1553, 1455, 1284, 1235, 751.

67-2:

^{1}H NMR (400 MHz, $CDCl_3$): δ 13.23 (s, 1 H), 7.47–7.33 (m, 2 H), 7.14 (dd, *J* = 7.5, 2.0 Hz, 1 H), 3.76–3.57 (m, 2 H), 3.54–3.43 (m, 1 H), 3.29 (m, 2 H), 1.88–1.59 (m, 4 H), 1.44–1.34 (m, 2 H), 1.19 (d, J = 6.9 Hz, 3 H), 0.97 (t, *J* = 7.4 Hz, 3 H) ppm.

^{13}C NMR (100 MHz, $CDCl_3$): δ 185.5, 183.1, 160.2, 148.8, 133.2, 132.6, 124.1,

118.9, 118.2, 114.9, 54.1, 46.6, 31.1, 26.7, 23.8, 20.9, 20.1, 13.9 ppm.

HRMS (ESI) *m/z* calcd. for $C_{18}H_{21}NO_3H^+$ $[M + H]^+$ 300.1594, found: 300.1588.

IR (KBr, cm^{-1}): ν_{max} 3365, 2958, 2924, 2856, 1615, 1548, 1471, 1346, 1273, 1213.

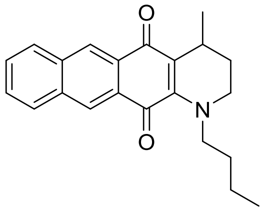

1-Butyl-4-methyl-1,2,3,4-tetrahydronaphtho[2,3-*g*]quinoline-5,12-dione (68): The *General Procedure C* was applied with anthracene-1,4-dione (109.5 mg, 0.5 mmol, 1 equiv.), dibutylamine (170.1 μL, 1.0 mmol, 2 equiv.), H_2SO_4 (67.5 μL, 2.4 equiv.), $FeCl_2$ (3.2 mg, 0.025 mmol, 0.05 equiv.), BCMOM (37.2 mg, 0.05 mmol, 0.1 equiv.), H_2O_2 (35%) (129 μL, 1.5 mmol, 3 equiv.), acetonitrile (4 mL) and water (4 mL) at 80 ℃ for 1 h. Column chromatography (PE/EA/Et_3N, from 20:1:0.1 to 10:1:0.1) afforded the title product as an orange red solid (113.3 mg, 68%).

TLC: R_f = 0.38 (silica gel, PE/EA/Et_3N, 10:1:0.1).

^{1}H NMR (400 MHz, $CDCl_3$): δ 8.49 (s, 1 H), 8.42 (s, 1 H) 7.97 (t, *J* = 7.7 Hz, 2 H), 7.68–7.51 (m, 2 H), 3.93–3.56 (m, 2 H), 3.54–3.37 (m, 2 H), 3.36–3.21 (m, 1 H), 1.88–1.61 (m, 4 H), 1.41-1.35 (m, 2 H), 1.22 (d, *J* = 6.9 Hz, 3 H), 0.97 (t, *J* = 7.4 Hz, 3 H) ppm.

^{13}C NMR (100 MHz, $CDCl_3$): δ 183.2, 180.2, 149.3, 135.2, 134.0, 129.8, 129.7, 129.6, 128.8, 128.1,128.0, 126.4, 122.9, 54.0, 46.3, 31.2, 26.8, 24.7, 21.0, 20.2, 13.9 ppm.

HRMS (ESI) *m/z* calcd. for $C_{22}H_{23}NO_2H^+$ $[M + H]^+$ 334.1802, found: 334.1804.

IR (KBr, cm^{-1}): ν_{max} 3336, 2956, 2928, 2867, 1669, 1546, 1456, 1366, 1282, 1185, 759.

Mp: 107.7–108.3 ℃.

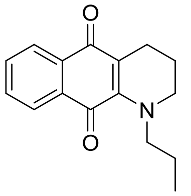

1-Propyl-1,2,3,4-tetrahydrobenzo[*g*]quinoline-5,10-dione (69): The *General Procedure C* was applied with naphthalene-1,4-dione (80.7 mg, 0.5 mmol, 1 equiv.), dipropylamine (138.5 μL, 1.0 mmol, 2 equiv.), H_2SO_4 (67.5 μL, 2.4 equiv.), $FeCl_2$ (3.2 mg, 0.025 mmol, 0.05 equiv.), BCMOM (37.2 mg, 0.05 mmol, 0.1 equiv.), H_2O_2 (35%) (129 μL, 1.5 mmol, 3 equiv.), acetonitrile (4 mL) and water (4 mL) at 80 ℃ for 1 h. Column chromatography (PE/EA/Et_3N, 10:1:0.1) afforded the title product as a rose red solid (79.4 mg, 62%).

TLC: R_f = 0.70 (silica gel, PE/EA/Et_3N, 10:1:0.1).

^{1}H NMR (400 MHz, $CDCl_3$): δ 8.01 (dd, *J* = 7.6, 0.9 Hz, 1 H), 7.90 (dd, *J* = 7.6, 0.9 Hz, 1 H), 7.63 (td, *J* = 7.5, 1.2 Hz, 1 H), 7.54 (td, *J* = 7.5, 1.2 Hz, 1 H), 3.60–3.53 (m, 2 H), 3.36–3.30 (m, 2 H), 2.65 (t, *J* = 6.4 Hz, 2 H), 1.93–1.86 (m, 2 H), 1.78–1.68 (m, 2 H), 0.94 (t, *J* = 7.4 Hz, 3 H) ppm.

^{13}C NMR (100 MHz, $CDCl_3$): δ 183.3, 181.0, 148.8, 133.5, 132.8, 132.4, 131.5, 125.8, 125.2, 116.2, 55.6, 50.9, 22.3, 21.2, 20.5, 11.2 ppm.

HRMS (ESI) *m/z* calcd. for $C_{16}H_{17}NO_2H^+$ [M + H]$^+$ 256.1332, found: 256.1328.

IR (KBr, cm^{-1}): ν_{max} 3357, 2961, 2922, 2850, 1670, 1619, 1592, 1549, 1207, 1161, 1082, 960, 723.

Mp: 88.6–89.2 ℃.

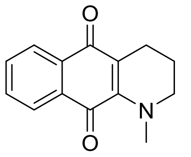

1-Methyl-1,2,3,4-tetrahydrobenzo[*g*]quinoline-5,10-dione (70): The *General Procedure C* was applied with naphthalene-1,4-dione (80.7 mg, 0.5 mmol, 1 equiv.), *N*-methylpropan-1-amine (105.7 μL, 1.0 mmol, 2 equiv.), H_2SO_4 (67.5 μL, 2.4 equiv.),

$FeCl_2$ (3.2 mg, 0.025 mmol, 0.05 equiv.), BCMOM (37.2 mg, 0.05 mmol, 0.1 equiv.), H_2O_2 (35%) (129 μL, 1.5 mmol, 3 equiv.), acetonitrile (4 mL) and water (4 mL) at 80 °C for 1 h. Column chromatography (PE/EA/Et_3N, from 20:1:0.1 to 10:1.5:0.1) afforded the title product as a red solid (54.5 mg, 48%).

TLC: R_f = 0.35 (silica gel, PE/EA/Et_3N, 10:1.5:0.1).

^{1}H NMR (400 MHz, $CDCl_3$): δ 8.04 (dd, *J* = 7.6, 0.9 Hz, 1 H), 7.93 (dd, *J* = 7.6, 0.9 Hz, 1 H), 7.66 (td, *J* = 7.5, 1.3 Hz, 1 H), 7.57 (td, *J* = 7.5, 1.3 Hz, 1 H), 3.40–3.30 (m, 5 H), 2.67 (t, *J* = 6.3 Hz, 2 H), 1.99–1.88 (m, 2 H) ppm.

^{13}C NMR (100 MHz, $CDCl_3$): δ 183.4, 180.9, 149.0, 133.6, 132.9, 132.3, 131.5, 125.9, 125.3, 116.3, 53.0, 41.9, 20.7, 20.4 ppm.

HRMS (ESI) *m/z* calcd. for $C_{14}H_{13}NO_2H^+$ $[M + H]^+$ 228.1019, found: 228.1013.

IR (KBr, cm^{-1}): ν_{max} 2929, 2850, 1670, 1593, 1552, 1383, 1279, 1210, 954, 723.

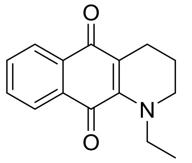

1-Ethyl-1,2,3,4-tetrahydrobenzo[*g*]quinoline-5,10-dione (71): The *General Procedure C* was applied with naphthalene-1,4-dione (80.7 mg, 0.5 mmol, 1 equiv.), *N*-ethylpropan-1-amine (123.5 μL, 1.0 mmol, 2 equiv.), H_2SO_4 (67.5 μL, 2.4 equiv.), $FeCl_2$ (3.2 mg, 0.025 mmol, 0.05 equiv.), BCMOM (37.2 mg, 0.05 mmol, 0.1 equiv.), H_2O_2 (35%) (129 μL, 1.5 mmol, 3 equiv.), acetonitrile (4 mL) and water (4 mL) at 80 °C for 1 h. Column chromatography (PE/EA/Et_3N, from 20:1:0.1 to 10:1:0.1) afforded the title product as a red solid (50.6 mg, 42%).

TLC: R_f = 0.45 (silica gel, PE/EA/Et_3N, 10:1:0.1).

^{1}H NMR (400 MHz, $CDCl_3$): δ 8.02 (dd, *J* = 7.5, 0.6 Hz, 1 H), 7.96–7.87 (m, 1 H), 7.63 (td, *J* = 7.5, 1.2 Hz, 1 H), 7.55 (td, *J* = 7.5, 1.2 Hz, 1 H), 3.64 (q, *J* = 7.0 Hz, 2 H), 3.37–3.27 (m, 2 H), 2.65 (t, *J* = 6.3 Hz, 2 H), 1.94–1.86 (m, 2 H), 1.30 (t, *J* = 7.0 Hz, 3 H) ppm.

^{13}C NMR (100 MHz, $CDCl_3$): δ 183.3, 181.0, 148.8, 133.5, 132.9, 132.4, 131.5, 125.8, 125.2, 116.3, 50.1, 48.8, 21.1, 20.5, 14.1 ppm.

HRMS (ESI) *m/z* calcd. for $C_{15}H_{15}NO_2H^+$ $[M + H]^+$ 242.1176, found: 242.1170.

IR (KBr, cm^{-1}): ν_{max} 2926, 2852, 1672, 1590, 1550, 1382, 1266, 1208, 1161, 1077, 724.

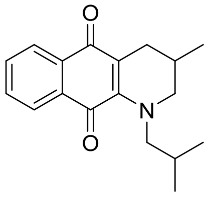

1-Isobutyl-3-methyl-1,2,3,4-tetrahydrobenzo[*g*]quinoline-5,10-dione (72): The *General Procedure C* was applied with naphthalene-1,4-dione (80.7 mg, 0.5 mmol, 1 equiv.), diisobutylamine (176.4 μL, 1.0 mmol, 2 equiv.), H_2SO_4 (67.5 μL, 2.4 equiv.), $FeCl_2$ (3.2 mg, 0.025 mmol, 0.05 equiv.), BCMOM (37.2 mg, 0.05 mmol, 0.1 equiv.), H_2O_2 (35%) (129 μL, 1.5 mmol, 3 equiv.), acetonitrile (4 mL) and water (4 mL) at 80 ℃ for 1 h. Column chromatography (PE/EA/Et_3N, from 20:1:0.1 to 10:1:0.1) afforded the title product as a red solid (84.9 mg, 60%).

TLC: R_f = 0.48 (silica gel, PE/EA/Et_3N, 10:1:0.1).

^{1}H NMR (400 MHz, $CDCl_3$): δ 8.03 (dd, *J* = 7.6, 0.6 Hz, 1 H), 7.95–7.88 (m, 1 H), 7.64 (td, *J* = 7.5, 1.2 Hz, 1 H), 7.56 (td, *J* = 7.5, 1.2 Hz, 1 H), 3.71 (m, 1 H), 3.53 (m, 1 H), 3.27 (m, 1 H), 2.96 (m, 2 H), 2.16–2.03 (m, 2 H), 1.98 (m, 1 H), 1.09 (d, *J* = 6.6 Hz, 3 H), 0.95 (d, *J* = 6.7 Hz, 3 H), 0.92 (d, *J* = 6.7 Hz, 3 H) ppm.

^{13}C NMR (100 MHz, $CDCl_3$): δ 183.5, 181.2, 148.7, 133.5, 132.8, 132.6, 131.5, 125.9, 125.1, 115.8, 60.5, 58.2, 29.3, 28.5, 25.7, 20.2, 20.0, 18.7 ppm.

HRMS (ESI) *m/z* calcd. for $C_{18}H_{21}NO_2H^+$ $[M + H]^+$ 284.1645, found: 284.1641.

IR (KBr, cm^{-1}): ν_{max} 2958, 2927, 2870, 1670, 1619, 1591, 1548, 1384, 1286, 1248, 1224, 1162, 990, 721.

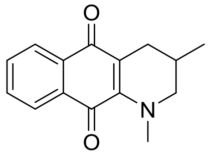

1,3-Dimethyl-1,2,3,4-tetrahydrobenzo[*g*]quinoline-5,10-dione (73): The *General Procedure C* was applied with naphthalene-1,4-dione (80.7 mg, 0.5 mmol, 1 equiv.), *N*,2-dimethylpropan-1-amine (125.7 μL, 1.0 mmol, 2 equiv.), H_2SO_4 (67.5 μL, 2.4 equiv.), $FeCl_2$ (3.2 mg, 0.025 mmol, 0.05 equiv.), BCMOM (37.2 mg, 0.05 mmol, 0.1 equiv.), H_2O_2 (35%) (129 μL, 1.5 mmol, 3 equiv.), acetonitrile (4 mL) and water (4 mL) at 80 ℃ for 1 h. Column chromatography (PE/EA/Et_3N, from 20:1:0.1 to 10:1.5:0.1) afforded the title product as a red solid (60.3 mg, 50%).

TLC: R_f = 0.50 (silica gel, PE/EA/Et_3N, 10:1.5:0.1).

^{1}H NMR (400 MHz, $CDCl_3$): δ 8.02 (dd, *J* = 7.6, 0.9 Hz, 1 H), 7.91 (dd, *J* = 7.6, 0.9 Hz, 1 H), 7.63 (td, *J* = 7.5, 1.3 Hz, 1 H), 7.55 (td, *J* = 7.5, 1.3 Hz, 1 H), 3.35 (s, 3 H), 3.25 (m, 1 H), 2.92 (m, 2 H), 2.08 (m, 1 H), 2.02–1.95 (m, 1 H), 1.08 (d, *J* = 6.5 Hz, 3 H) ppm.

^{13}C NMR (100 MHz, $CDCl_3$): δ 183.3, 181.1, 148.4, 133.6, 132.9, 132.3, 131.5, 125.9, 125.3, 115.7, 59.6, 42.0, 28.7, 25.7, 18.7 ppm.

HRMS (ESI) *m/z* calcd. for $C_{15}H_{15}NO_2H^+$ $[M + H]^+$ 242.1176, found: 242.1170.

IR (KBr, cm^{-1}): ν_{max} 2953, 2926, 2852, 1670, 1592, 1555, 1385, 1262, 1170, 722.

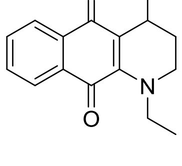

1-Ethyl-4-methyl-1,2,3,4-tetrahydrobenzo[*g*]quinoline-5,10-dione (74): The *General Procedure C* was applied with naphthalene-1,4-dione (80.7 mg, 0.5 mmol, 1 equiv.), *N*-ethylbutan-1-amine (139.5 μL, 1.0 mmol, 2 equiv.), H_2SO_4 (67.5 μL, 2.4 equiv.), $FeCl_2$ (3.2 mg, 0.025 mmol, 0.05 equiv.), BCMOM (37.2 mg, 0.05 mmol, 0.1 equiv.), H_2O_2 (35%) (129 μL, 1.5 mmol, 3 equiv.), acetonitrile (4 mL) and water (4

mL) at 80 ℃ for 1 h. Column chromatography (PE/EA/Et_3N, from 20:1:0.1 to 10:1:0.1) afforded the title product as a red solid (89.3 mg, 70%).

TLC: R_f = 0.50 (silica gel, PE/EA/Et_3N, 10:1:0.1).

^{1}H NMR (400 MHz, $CDCl_3$): δ 8.03 (d, *J* = 7.6 Hz, 1 H), 7.91 (d, *J* = 7.6 Hz, 1 H), 7.65 (t, *J* = 7.4 Hz, 1 H), 7.56 (t, *J* = 7.4 Hz, 1 H), 3.77–3.57 (m, 2 H), 3.49–3.26 (m, 3 H), 1.85–1.73 (m, 2 H), 1.34 (t, *J* = 7.0 Hz, 3 H), 1.20 (d, *J* = 6.9 Hz, 3 H) ppm.

^{13}C NMR (100 MHz, $CDCl_3$): δ 183.6, 180.5, 148.1, 133.5, 133.0, 132.4, 131.4, 125.8, 125.2, 120.8, 48.9, 45.5, 26.9, 24.4, 21.0, 14.1 ppm.

HRMS (ESI) *m/z* calcd. for $C_{16}H_{17}NO_2H^+$ $[M + H]^+$ 256.1332, found: 256.1327.

IR (KBr, cm^{-1}): ν_{max} 2958, 2931, 2869, 1672, 1595, 1548, 1356, 1300, 1270, 1212, 723.

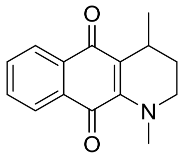

1,4-Dimethyl-1,2,3,4-tetrahydrobenzo[*g*]quinoline-5,10-dione (75): The *General Procedure C* was applied with naphthalene-1,4-dione (80.7 mg, 0.5 mmol, 1 equiv.), *N*-methylbutan-1-amine (127.4 μL, 1.0 mmol, 2 equiv.), H_2SO_4 (67.5 μL, 2.4 equiv.), $FeCl_2$ (3.2 mg, 0.025 mmol, 0.05 equiv.), BCMOM (37.2 mg, 0.05 mmol, 0.1 equiv.), H_2O_2 (35%) (129 μL, 1.5 mmol, 3 equiv.), acetonitrile (4 mL) and water (4 mL) at 80 ℃ for 1 h. Column chromatography (PE/EA/Et_3N, from 20:1:0.1 to 10:1:0.1) afforded the title product as a red solid (86.8 mg, 72%).

TLC: R_f = 0.50 (silica gel, PE/EA/Et_3N, 10:1:0.1).

^{1}H NMR (400 MHz, $CDCl_3$): δ 8.01 (d, *J* = 7.6 Hz, 1 H), 7.89 (d, *J* = 7.6 Hz, 1 H), 7.63 (td, *J* = 7.5, 1.0 Hz, 1 H), 7.54 (dd, *J* = 10.8, 4.3 Hz, 1 H), 3.50–3.21 (m, 6 H), 1.83 (m, 1 H), 1.76–1.70 (m, 1 H), 1.17 (d, *J* = 6.9 Hz, 3 H) ppm.

^{13}C NMR (100 MHz, $CDCl_3$): δ 183.8, 180.4, 148.1, 133.6, 133.1, 132.3, 131.5, 125.8, 125.3, 120.6, 48.5, 42.0, 26.8, 24.2, 20.8 ppm.

HRMS (ESI) *m/z* calcd. for $C_{15}H_{15}NO_2H^+$ $[M + H]^+$ 242.1176, found: 242.1168.

IR (KBr, cm^{-1}): ν_{max} 2952, 2922, 2861, 1681, 1606, 1550, 1386, 1364, 1295, 1217, 798, 724, 697.

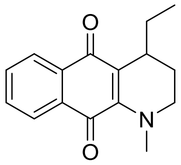

4-Ethyl-1-methyl-1,2,3,4-tetrahydrobenzo[*g*]quinoline-5,10-dione (76): The *General Procedure C* was applied with naphthalene-1,4-dione (80.7 mg, 0.5 mmol, 1 equiv.), *N*-methylpentan-1-amine (139.9 μL, 1.0 mmol, 2 equiv.), H_2SO_4 (67.5 μL, 2.4 equiv.), $FeCl_2$ (3.2 mg, 0.025 mmol, 0.05 equiv.), BCMOM (37.2 mg, 0.05 mmol, 0.1 equiv.), H_2O_2 (35%) (129 μL, 1.5 mmol, 3 equiv.), acetonitrile (4 mL) and water (4 mL) at 80 ℃ for 1 h. Column chromatography (PE/EA/Et_3N, from 20:1:0.1 to 10:1:0.1) afforded the title product as a red solid (88.0 mg, 69%).

TLC: R_f = 0.45 (silica gel, PE/EA/Et_3N, 10:1:0.1).

^{1}H NMR (400 MHz, $CDCl_3$): δ 8.01 (d, *J* = 7.6 Hz, 1 H), 7.90 (d, *J* = 7.6 Hz, 1 H), 7.63 (td, *J* = 7.5, 1.0 Hz, 1 H), 7.54 (td, *J* = 7.5, 1.0 Hz, 1 H), 3.42–3.22 (m, 5 H), 3.10 (m, 1 H), 2.01–1.90 (m, 1 H), 1.68 (m, 1 H), 1.34–1.18 (m, 2 H), 0.98 (t, *J* = 7.4 Hz, 3 H) ppm.

^{13}C NMR (100 MHz, $CDCl_3$): δ 183.7, 180.5, 147.8, 133.5, 133.0, 132.3, 131.4, 125.7, 125.3, 120.1, 48.6, 41.9, 30.7, 27.0, 22.7, 11.7 ppm.

HRMS (ESI) *m/z* calcd. for $C_{16}H_{17}NO_2H^+$ $[M + H]^+$ 256.1332, found: 256.1337.

IR (KBr, cm^{-1}): ν_{max} 2930, 1675, 1606, 1555, 1454, 1388, 1283, 1217, 957, 798, 725.

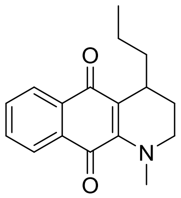

1-Methyl-4-propyl-1,2,3,4-tetrahydrobenzo[*g*]quinoline-5,10-dione (77): The

General Procedure C was applied with naphthalene-1,4-dione (80.7 mg, 0.5 mmol, 1 equiv.), *N*-methylhexan-1-amine (156.3 μL, 1.0 mmol, 2 equiv.), H_2SO_4 (67.5 μL, 2.4 equiv.), $FeCl_2$ (3.2 mg, 0.025 mmol, 0.05 equiv.), BCMOM (37.2 mg, 0.05 mmol, 0.1 equiv.), H_2O_2 (35%) (129 μL, 1.5 mmol, 3 equiv.), acetonitrile (4 mL) and water (4 mL) at 80 ℃ for 1 h. Column chromatography (PE/EA/Et_3N, from 20:1:0.1 to 10:1:0.1) afforded the title product as a red solid (78.1 mg, 58%).

TLC: R_f = 0.50 (silica gel, PE/EA/Et_3N, 10:1:0.1).

^{1}H NMR (400 MHz, $CDCl_3$): δ 8.02 (d, *J* = 7.6 Hz, 1 H), 7.90 (d, *J* = 7.6 Hz, 1 H), 7.66–7.60 (m, 1 H), 7.54 (td, *J* = 7.5, 0.9 Hz, 1 H), 3.42–3.33 (m, 4 H), 3.29–3.18 (m, 2 H), 1.96–1.89 (m, 1 H), 1.68 (m, 1 H), 1.61–1.54 (m, 1 H), 1.52–1.43 (m, 1 H), 1.41–1.33 (m, 1 H), 1.28–1.23 (m, 1 H), 0.94 (t, *J* = 7.2 Hz, 3 H) ppm.

^{13}C NMR (100 MHz, $CDCl_3$): δ 183.7, 180.5, 147.8, 133.6, 133.0, 132.4, 131.4, 125.8, 125.3, 120.3, 48.6, 41.9, 36.5, 28.8, 23.3, 20.3, 14.2 ppm.

HRMS (ESI) *m/z* calcd. for $C_{17}H_{19}NO_2H^+$ $[M + H]^+$ 270.1489, found: 270.1483.

IR (KBr, cm^{-1}): ν_{max} 2956, 2929, 2855, 1671, 1620, 1594, 1553, 1381, 1281, 1220, 723.

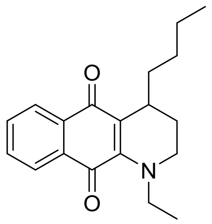

4-Butyl-1-ethyl-1,2,3,4-tetrahydrobenzo[*g*]quinoline-5,10-dione (78): The *General Procedure C* was applied with naphthalene-1,4-dione (80.7 mg, 0.5 mmol, 1 equiv.), *N*-ethylheptan-1-amine (190.0 μL, 1.0 mmol, 2 equiv.), H_2SO_4 (67.5 μL, 2.4 equiv.), $FeCl_2$ (3.2 mg, 0.025 mmol, 0.05 equiv.), BCMOM (37.2 mg, 0.05 mmol, 0.1 equiv.), H_2O_2 (35%) (129 μL, 1.5 mmol, 3 equiv.), acetonitrile (4 mL) and water (4 mL) at 80 ℃ for 1 h. Column chromatography (PE/EA/Et_3N, from 20:1:0.1 to 10:1.5:0.1) afforded the title product as a red solid (66.9 mg, 45%).

TLC: R_f = 0.50 (silica gel, PE/EA/Et_3N, 10:1.5:0.1).

^{1}H NMR (400 MHz, $CDCl_3$): δ 8.02 (dd, *J* = 7.6, 0.9 Hz, 1 H), 7.90 (dd, *J* = 7.6, 0.9 Hz, 1 H), 7.63 (td, *J* = 7.5, 1.2 Hz, 1 H), 7.54 (td, *J* = 7.5, 1.2 Hz, 1 H), 3.78–3.53 (m, 2 H), 3.44–3.24 (m, 2 H), 3.18 (m, 1 H), 2.0–1.91 (m, 1 H), 1.74–1.62 (m, 2 H), 1.45–1.30 (m, 8 H), 0.91 (t, *J* = 6.8 Hz, 3 H) ppm.

^{13}C NMR (100 MHz, $CDCl_3$): δ 183.6, 180.6, 147.8, 133.5, 133.0, 132.5, 131.4, 125.8, 125.3, 120.5, 48.8, 45.6, 34.1, 29.3, 29.3, 23.2, 22.8, 14.1, 14.1 ppm.

HRMS (ESI) *m/z* calcd. for $C_{19}H_{23}NO_2H^+$ $[M + H]^+$ 298.1802, found: 298.1796.

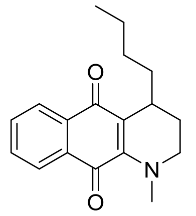

4-Butyl-1-methyl-1,2,3,4-tetrahydrobenzo[*g*]quinoline-5,10-dione (79): The *General Procedure C* was applied with naphthalene-1,4-dione (80.7 mg, 0.5 mmol, 1 equiv.), *N*-methylheptan-1-amine (173.5 μL, 1.0 mmol, 2 equiv.), H_2SO_4 (67.5 μL, 2.4 equiv.), $FeCl_2$ (3.2 mg, 0.025 mmol, 0.05 equiv.), BCMOM (37.2 mg, 0.05 mmol, 0.1 equiv.), H_2O_2 (35%) (129 μL, 1.5 mmol, 3 equiv.), acetonitrile (4 mL) and water (4 mL) at 80 ℃ for 1 h. Column chromatography (PE/EA/Et_3N, from 30:1:0.1 to 15:1:0.1) afforded the title product as a red solid (56.6 mg, 40%).

TLC: R_f = 0.48 (silica gel, PE/EA/Et_3N, 15:1:0.1).

^{1}H NMR (400 MHz, $CDCl_3$): δ 8.02 (dd, *J* = 7.6, 0.6 Hz, 1 H), 7.90 (dd, *J* = 7.6, 0.6 Hz, 1 H), 7.64 (td, *J* = 7.5, 1.1 Hz, 1 H), 7.54 (td, *J* = 7.5, 1.1 Hz, 1 H), 3.45–3.31 (m, 4 H), 3.29–3.23 (m, 1 H), 3.19 (m, 1 H), 1.93 (m, 1 H), 1.74–1.62 (m, 2 H), 1.44–1.29 (m, 5 H), 0.91 (t, *J* = 6.8 Hz, 3 H) ppm.

^{13}C NMR (100 MHz, $CDCl_3$): δ 183.8, 180.4, 147.8, 133.6, 133.0, 132.3, 131.4, 125.8, 125.3, 120.3, 48.6, 42.0, 33.9, 29.3, 29.0, 23.2, 22.8, 14.1 ppm.

HRMS (ESI) *m/z* calcd. for $C_{18}H_{21}NO_2H^+$ $[M + H]^+$ 284.1645, found: 284.1642.

IR (KBr, cm^{-1}): ν_{max} 2927, 2855, 1672, 1596, 1552, 1379, 1280, 1217, 955, 724.

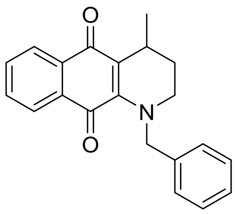

1-Benzyl-4-methyl-1,2,3,4-tetrahydrobenzo[*g*]quinoline-5,10-dione (80): The *General Procedure C* was applied with naphthalene-1,4-dione (80.7 mg, 0.5 mmol, 1 equiv.), *N*-benzylbutan-1-amine (184.8 μL, 1.0 mmol, 2 equiv.), H_2SO_4 (67.5 μL, 2.4 equiv.), $FeCl_2$ (3.2 mg, 0.025 mmol, 0.05 equiv.), BCMOM (37.2 mg, 0.05 mmol, 0.1 equiv.), H_2O_2 (35%) (129 μL, 1.5 mmol, 3 equiv.), acetonitrile (4 mL) and water (4 mL) at 80 ℃ for 1 h. Column chromatography (PE/EA/Et_3N, from 20:1:0.1 to 10:1:0.1) afforded the title product as an orange solid (79.3 mg, 50%).

TLC: R_f = 0.50 (silica gel, PE/EA/Et_3N, 10:1:0.1).

^{1}H NMR (400 MHz, $CDCl_3$): δ 8.04 (dd, *J* = 7.6, 0.6 Hz, 1 H), 7.89 (dd, *J* = 7.6, 0.6 Hz, 1 H), 7.65 (td, *J* = 7.5, 1.1 Hz, 1 H), 7.55 (td, *J* = 7.5, 1.1 Hz, 1 H), 7.42–7.27 (m, 5 H), 5.01 (d, *J* = 15.6 Hz, 1 H), 4.78 (d, *J* = 15.6 Hz, 1 H), 3.36 (m, 2 H), 3.23 (m, 1 H), 1.81 (m, 1 H), 1.70 (m, 1 H), 1.18 (d, *J* = 6.9 Hz, 3 H) ppm.

^{13}C NMR (100 MHz, $CDCl_3$): δ 183.5, 181.0, 148.1, 137.9, 133.6, 132.9, 132.5, 131.7, 128.6, 127.3, 127.2, 126.0, 125.4, 122.0, 56.9, 45.6, 26.7, 24.5, 21.0 ppm.

HRMS (ESI) *m/z* calcd. for $C_{21}H_{19}NO_2H^+$ $[M + H]^+$ 318.1489, found: 318.1492.

IR (KBr, cm^{-1}): ν_{max} 3388, 2926, 2855, 1673, 1597, 1548, 1512, 1336, 1298, 1262, 1215, 724.

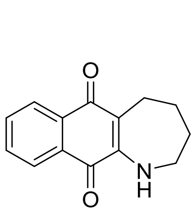

2,3,4,5-Tetrahydro-1*H*-naphtho[2,3-*b*]azepine-6,11-dione (81): The *General*

Procedure C was applied with naphthalene-1,4-dione (80.7 mg, 0.5 mmol, 1 equiv.), butan-1-amine (99.5μL, 1.0 mmol, 2 equiv.), H_2SO_4 (67.5 μL, 2.4 equiv.), $FeCl_2$ (3.2 mg, 0.025 mmol, 0.05 equiv.), BCMOM (37.2 mg, 0.05 mmol, 0.1 equiv.), H_2O_2 (35%) (129 μL, 1.5 mmol, 3 equiv.), acetonitrile (4 mL) and water (4 mL) at 80 ℃ for 1 h. Column chromatography (PE/EA/Et_3N, from 20:1:0.1 to 10:1:0.1) afforded the title product as a red solid (39.7 mg, 35%).

TLC: R_f = 0.60 (silica gel, PE/EA/Et_3N, 10:1:0.1).

^{1}H NMR (400 MHz, $CDCl_3$): δ 8.08 (d, *J* = 7.7 Hz, 1 H), 7.99 (d, *J* = 7.6 Hz, 1 H), 7.67 (t, *J* = 7.5 Hz, 1 H), 7.57 (t, *J* = 7.5 Hz, 1 H), 6.05 (s, 1 H), 3.46 (m, 2 H), 2.90 (m, 2 H), 1.88 (m, 4 H) ppm.

^{13}C NMR (100 MHz, $CDCl_3$): δ 183.0, 182.5, 148.8, 134.3, 133.5, 131.8, 130.3, 126.3, 125.8, 118.1, 45.2, 29.1, 25.4, 23.5 ppm.

HRMS (ESI) *m/z* calcd. for $C_{14}H_{13}NO_2H^+$ $[M + H]^+$ 228.1019, found: 228.1018.

IR (KBr, cm^{-1}): ν_{max} 3357, 2917, 2851, 1671, 1598, 1561, 1492, 1363, 1268, 723.

Mp: 91.7–92.3 ℃.

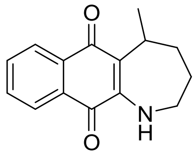

5-Methyl-2,3,4,5-tetrahydro-1*H*-naphtho[2,3-*b*]azepine-6,11-dione (82): The *General Procedure C* was applied with naphthalene-1,4-dione (80.7 mg, 0.5 mmol, 1 equiv.), pentan-1-amine (118.3 μL, 1.0 mmol, 2 equiv.), H_2SO_4 (67.5 μL, 2.4 equiv.), $FeCl_2$ (3.2 mg, 0.025 mmol, 0.05 equiv.), BCMOM (37.2 mg, 0.05 mmol, 0.1 equiv.), H_2O_2 (35%) (129 μL, 1.5 mmol, 3 equiv.), acetonitrile (4 mL) and water (4 mL) at 80 ℃ for 1 h. Column chromatography (PE/EA/Et_3N, from 20:1:0.1 to 10:1:0.1) afforded the title product as an orange solid (45.8 mg, 38%).

TLC: R_f = 0.60 (silica gel, PE/EA/Et_3N, 10:1:0.1).

^{1}H NMR (400 MHz, $CDCl_3$): δ 8.08 (dd, *J* = 7.6, 0.6 Hz, 1 H), 7.98 (dd, *J* = 7.6, 0.6

Hz, 1 H), 7.67 (td, J = 7.6, 1.1 Hz, 1 H), 7.56 (td, J = 7.6, 1.1 Hz, 1 H), 6.09 (s, 1 H), 3.78–3.58 (m, 2 H), 3.18–3.02 (m, 1 H), 1.93–1.68 (m, 4 H), 1.21 (d, J = 7.2 Hz, 3 H) ppm.

^{13}C NMR (100 MHz, $CDCl_3$): δ 183.1, 183.0, 148.3, 134.3, 133.5, 131.8, 130.2, 126.3, 125.8, 123.3, 46.8, 30.9, 29.3, 23.7, 19.7 ppm.

HRMS (ESI) *m/z* calcd. for $C_{15}H_{15}NO_2H^+$ $[M + H]^+$ 242.1176, found: 242.1169.

IR (KBr, cm^{-1}): ν_{max} 3352, 2922, 2857, 1670, 1605, 1575, 1502, 1329, 1267, 1129, 1072, 907, 726, 695, 572.

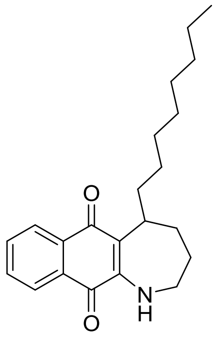

5-Octyl-2,3,4,5-tetrahydro-1*H*-naphtho[2,3-*b*]azepine-6,11-dione (83): The *General Procedure C* was applied with naphthalene-1,4-dione (80.7 mg, 0.5 mmol, 1 equiv.), dodecan-1-amine (191.1 μL, 1.0 mmol, 2 equiv.), H_2SO_4 (67.5 μL, 2.4 equiv.), $FeCl_2$ (3.2 mg, 0.025 mmol, 0.05 equiv.), BCMOM (37.2 mg, 0.05 mmol, 0.1 equiv.), H_2O_2 (35%) (129 μL, 1.5 mmol, 3 equiv.), acetonitrile (4 mL) and water (4 mL) at 80 °C for 1 h. Column chromatography (PE/EA/Et_3N, from 20:1:0.1 to 10:1:0.1) afforded the title product as an orange solid (47.5 mg, 28%).

TLC: R_f = 0.60 (silica gel, PE/EA/Et_3N, 10:1:0.1).

^{1}H NMR (400 MHz, $CDCl_3$): δ 8.08 (dd, J = 7.6, 0.9 Hz, 1 H), 7.99 (dd, J = 7.6, 0.9 Hz, 1 H), 7.68 (td, J = 7.5, 1.3 Hz, 1 H), 7.57 (td, J = 7.5, 1.3 Hz, 1 H), 6.05 (s, 1 H), 3.69–3.53 (m, 2 H), 3.06 (m, 1 H), 2.08–2.00 (m, 1 H), 1.81 (m, 2 H), 1.65–1.25 (m, 15 H), 0.86 (t, J = 6.7 Hz, 3 H) ppm.

^{13}C NMR (100 MHz, $CDCl_3$): δ 183.3, 183.1, 148.4, 134.3, 133.5, 131.8, 130.2,

126.4, 125.8, 123.8, 47.0, 34.0, 33.3, 31.9, 29.8, 29.6, 29.3, 28.0, 27.6, 23.7, 22.7, 14.1 ppm.

HRMS (ESI) *m/z* calcd. for $C_{22}H_{29}NO_2H^+$ $[M + H]^+$ 340.2271, found: 340.2264.

IR (KBr, cm^{-1}): ν_{max} 3344, 2922, 2854, 1667, 1605, 1572, 1501, 1334, 1267, 725.

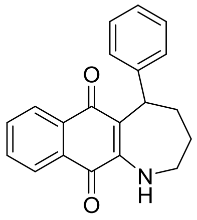

5-Phenyl-2,3,4,5-tetrahydro-1*H*-naphtho[2,3-*b*]azepine-6,11-dione (84): The *General Procedure C* was applied with naphthalene-1,4-dione (80.7 mg, 0.5 mmol, 1 equiv.), 4-phenylbutan-1-amine (161.3 μL, 1.0 mmol, 2 equiv.), H_2SO_4 (67.5 μL, 2.4 equiv.), $FeCl_2$ (3.2 mg, 0.025 mmol, 0.05 equiv.), BCMOM (37.2 mg, 0.05 mmol, 0.1 equiv.), H_2O_2 (35%) (129 μL, 1.5 mmol, 3 equiv.), acetonitrile (4 mL) and water (4 mL) at 80 ℃ for 1 h. Column chromatography (PE/EA/Et_3N, from 30:1:0.1 to 20:1:0.1) afforded the title product as a red solid (60.6 mg, 40%).

TLC: R_f = 0.50 (silica gel, PE/EA/Et_3N, 20:1:0.1).

^{1}H NMR (400 MHz, $CDCl_3$): δ 8.12–8.01 (m, 2 H), 7.69 (dd, *J* = 13.8, 6.3 Hz, 1 H), 7.59 (t, *J* = 7.5 Hz, 1 H), 7.32–7.23 (m, 4 H), 7.15 (t, *J* = 6.6 Hz, 1 H), 6.31 (s, 1 H), 4.99–4.87 (m, 1 H), 3.52 (m, 1 H), 3.11–3.01 (m, 1 H), 2.48–2.39 (m, 1 H), 2.16 (m, 1 H), 1.86–1.76 (m, 1 H), 1.67–1.57 (m, 1 H) ppm.

^{13}C NMR (100 MHz, $CDCl_3$): δ 182.7, 149.3, 144.5, 134.6, 133.5, 131.9, 130.3, 128.2, 127.8, 126.6, 125.9, 125.7, 123.2, 118.2, 45.0, 39.8, 31.0, 24.4 ppm.

HRMS (ESI) *m/z* calcd. for $C_{20}H_{17}NO_2H^+$ $[M + H]^+$ 304.1332, found: 304.1331.

IR (KBr, cm^{-1}): ν_{max} 3344, 2930, 1712, 1670, 1599, 1567, 1499, 1333, 1271, 728, 698.

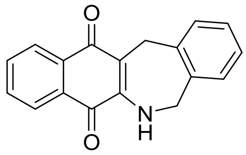

7,12-Dihydro-5*H*-benzo[*e*]naphtho[2,3-*b*]azepine-5,13(6*H*)-dione (85): The *General Procedure C* was applied with naphthalene-1,4-dione (80.7 mg, 0.5 mmol, 1 equiv.), *o*-tolylmethanamine (126.6 μL, 1.0 mmol, 2 equiv.), H_2SO_4 (67.5 μL, 2.4 equiv.), $FeCl_2$ (3.2 mg, 0.025 mmol, 0.05 equiv.), BCMOM (37.2 mg, 0.05 mmol, 0.1 equiv.), H_2O_2 (35%) (129 μL, 1.5 mmol, 3 equiv.), acetonitrile (4 mL) and water (4 mL) at 80 ℃ for 1 h. Column chromatography (PE/EA/Et_3N, from 20:1:0.1 to 10:1:0.1) afforded the title product as a red solid (52.3 mg, 38%).

TLC: R_f = 0.55 (silica gel, PE/EA/Et_3N, 10:1:0.1).

^{1}H NMR (400 MHz, $CDCl_3$): δ 8.10 (d, *J* = 7.6 Hz, 1 H), 7.94 (d, *J* = 7.6 Hz, 1 H), 7.67 (t, *J* = 7.5 Hz, 1 H), 7.54 (t, *J* = 7.5 Hz, 1 H), 7.32–7.17 (m, 4 H), 6.30 (s, 1 H), 4.69 (d, *J* = 5.3 Hz, 2 H), 4.25 (s, 2 H) ppm.

^{13}C NMR (100 MHz, $CDCl_3$): δ 181.9, 181.3, 145.0, 139.7, 135.6, 134.6, 133.3, 131.7, 130.0, 128.9, 128.8, 127.4, 127.3, 126.4, 125.8, 111.2, 46.8, 27.3 ppm.

HRMS (ESI) *m/z* calcd. for $C_{18}H_{13}NO_2H^+$ $[M + H]^+$ 276.1019, found: 276.1016.

IR (KBr, cm^{-1}): ν_{max} 3322, 2919, 1677, 1595, 1554, 1494, 1361, 1264, 722.

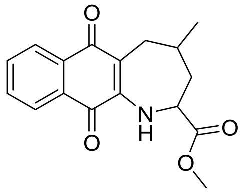

Methyl 4-methyl-6,11-dioxo-2,3,4,5,6,11-hexahydro-1*H*-naphtho[2,3-*b*]azepine-2-carboxylate (86): The *General Procedure C* was applied with naphthalene-1,4-dione (80.7 mg, 0.5 mmol, 1 equiv.), methyl L-leucinate hydrochloride (185.4 mg, 1.0 mmol, 2 equiv.), H_2SO_4 (67.5 μL, 2.4 equiv.), $FeCl_2$ (3.2 mg, 0.025 mmol, 0.05 equiv.), BCMOM (37.2 mg, 0.05 mmol, 0.1 equiv.), H_2O_2 (35%) (129 μL, 1.5 mmol, 3 equiv.), acetonitrile (4

mL) and water (4 mL) at 80 ℃ for 1 h. Column chromatography (PE/EA/Et_3N, 10:1:0.1) afforded the title product as an orange red solid (29.9 mg, 20%).

TLC: R_f = 0.40 (silica gel, PE/EA/Et_3N, 10:1:0.1).

^{1}H NMR (400 MHz, $CDCl_3$): δ 8.08 (d, *J* = 7.6 Hz, 1 H), 8.02 (d, *J* = 7.6 Hz, 1 H), 7.68 (td, *J* = 7.5, 1.0 Hz, 1 H), 7.59 (td, *J* = 7.5, 1.0 Hz, 1 H), 6.51 (s, 1 H), 4.53-4.48 (m, 1 H), 3.81 (s, 3 H), 3.13–2.93 (m, 1 H), 2.49 (m, 1 H), 2.33–2.13 (m, 2 H), 1.90 (m, 1 H), 1.11 (d, *J* = 6.5 Hz, 3 H) ppm.

^{13}C NMR (100 MHz, $CDCl_3$): δ 183.3, 181.5, 172.4, 147.1, 134.3, 133.2, 132.0, 130.3, 126.3, 126.0, 117.3, 55.1, 52.8, 40.3, 30.1, 30.0, 23.0 ppm.

HRMS (ESI) *m/z* calcd. for $C_{17}H_{17}NO_4H^+$ $[M + H]^+$ 300.1231, found: 300.1235.

IR (KBr, cm^{-1}): ν_{max} 3352, 2955, 2922, 2852, 1743, 1667, 1608, 1571, 1488, 1298, 1277, 1225, 724.

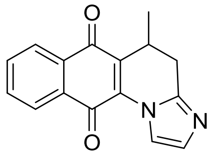

5-Methyl-4,5-dihydrobenzo[*g*]imidazo[1,2-*a*]quinoline-6,11-dione (87): The *General Procedure C* was applied with naphthalene-1,4-dione (80.7 mg, 0.5 mmol, 1 equiv.), 2-propyl-1*H*-imidazole (112.4 mg, 1.0 mmol, 2 equiv.), H_2SO_4 (67.5 μL, 2.4 equiv.), $FeCl_2$ (3.2 mg, 0.025 mmol, 0.05 equiv.), BCMOM (37.2 mg, 0.05 mmol, 0.1 equiv.), H_2O_2 (35%) (129 μL, 1.5 mmol, 3 equiv.), acetonitrile (4 mL) and water (4 mL) at 80 ℃ for 1 h. Column chromatography (PE/EA/Et_3N, 10:3:0.1) afforded the title product as an orange solid (33.0 mg, 25%).

TLC: R_f = 0.45 (silica gel, PE/EA/Et_3N, 10:3:0.1).

^{1}H NMR (400 MHz, $CDCl_3$): δ 8.18–8.13 (m, 2 H), 7.84–7.75 (m, 2 H), 7.26 (s, 1 H), 7.10 (d, *J* = 1.6 Hz, 1 H), 3.67–3.58 (m, 1 H), 3.07 (m, 2 H), 1.09 (d, *J* = 7.2 Hz, 3 H) ppm.

^{13}C NMR (100 MHz, $CDCl_3$): δ 182.9, 179.6, 145.1, 134.8, 134.5, 133.9, 131.5,

131.1, 129.6, 126.8, 126.4, 118.5, 28.7, 25.0, 18.2 ppm.

HRMS (ESI) *m/z* calcd. for $C_{16}H_{12}N_2O_2H^+$ $[M + H]^+$ 265.0972, found: 265.0961.

IR (KBr, cm^{-1}): ν_{max} 2922, 2852, 1667, 1610, 1415, 1353, 1260, 1127, 717.

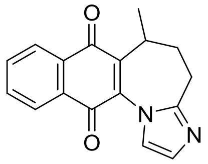

6-Methyl-5,6-dihydro-4*H*-imidazo[1,2-*a*]naphtho[2,3-*f*]azepine-7,12-dione (88): The *General Procedure C* was applied with naphthalene-1,4-dione (80.7 mg, 0.5 mmol, 1 equiv.), 2-butyl-1*H*-imidazole (128.0 mg, 1.0 mmol, 2 equiv.), H_2SO_4 (67.5 µL, 2.4 equiv.), $FeCl_2$ (3.2 mg, 0.025 mmol, 0.05 equiv.), BCMOM (37.2 mg, 0.05 mmol, 0.1 equiv.), H_2O_2 (35%) (129 µL, 1.5 mmol, 3 equiv.), acetonitrile (4 mL) and water (4 mL) at 80 ℃ for 1 h. Column chromatography (PE/EA/Et_3N, 10:5:0.2) afforded the title product as a brown wax (48.7 mg, 35%).

TLC: R_f = 0.30 (silica gel, PE/EA/Et_3N, 10:5:0.2).

^{1}H NMR (400 MHz, $CDCl_3$): δ 8.21–8.12 (m, 2 H), 7.83–7.76 (m, 2 H), 7.32 (d, *J* = 1.5 Hz, 1 H), 7.09 (d, *J* = 1.5 Hz, 1 H), 3.80–3.69 (m, 1 H), 3.01 (m, 1 H), 2.68 (m, 1 H), 2.51 (m, 1 H), 2.21 (m, 1 H), 0.70 (d, *J* = 7.4 Hz, 3 H) ppm.

^{13}C NMR (100 MHz, $CDCl_3$): δ 183.7, 179.9, 148.9, 144.6, 139.6, 134.4, 134.1, 131.9, 130.9, 128.3, 126.9, 126.7, 121.2, 37.8, 24.5, 18.7 ppm.

HRMS (ESI) *m/z* calcd. for $C_{17}H_{14}N_2O_2H^+$ $[M + H]^+$ 279.1128, found: 279.1127.

IR (KBr, cm^{-1}): ν_{max} 3365, 2923, 2851, 1672, 1614, 1419, 1275, 1147, 727.

Mp: 55.7–56.3 ℃.

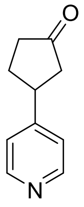

3-(Pyridin-4-yl)cyclopentan-1-one (89): *The General Procedure D* was applied with pyridine (40.0 μL, 0.5 mmol, 1 equiv.), H_2SO_4 (67.5 μL, 2.4 equiv.), $FeCl_2$ (3.2 mg, 0.025 mmol, 0.05 equiv.), BCMOM (37.2 mg, 0.05 mmol, 0.1 equiv.), cyclopentanone (88.9 μL, 2 equiv., 1.0 mmol), H_2O_2 (35%) (129 μL, 1.5 mmol, 3 equiv.), acetonitrile (4 mL) and water (4 mL) at 80 ℃ under N_2 for 3 h. Column chromatography (PE/EA/TEA, from 10:4:0.1 to 10:6:0.1) afforded the title product as a black liquid (48.3 mg, 60%), known compound (CAS: 1181456-15-4).

TLC: R_f = 0.49 (silica gel, PE/EA/TEA, 10:6:0.1).

^{1}H NMR (400 MHz, $CDCl_3$): δ 8.59 (dd, *J* = 4.8, 1.6 Hz, 2 H), 7.26 (d, *J* = 6.0, 2 H), 3.53–3.36 (m, 1 H), 2.71 (dd, *J* = 18.4, 7.2 Hz, 1 H), 2.51 (m, 2 H), 2.38–2.26 (m, 2 H), 2.07–1.91 (m, 1 H) ppm.

^{13}C NMR (100 MHz, $CDCl_3$): δ 216.4, 153.4, 149.0, 122.5, 44.6, 41.5, 38.5, 30.3 ppm.

HRMS (ESI) *m/z* calcd for $C_{10}H_{11}NOH^+$ $[M + H]^+$ 162.0914, found: 162.0908.

IR (KBr, cm^{-1}): ν_{max} 3355, 2919, 2847, 1738, 1631, 1602, 1407, 1135, 822.

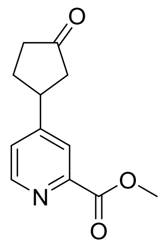

Methyl 4-(3-oxocyclopentyl)picolinate (90): *The General Procedure D* was applied with methyl picolinate (61.5 μL, 0.5 mmol, 1 equiv.), H_2SO_4 (67.5 μL, 2.4 equiv.), $FeCl_2$ (3.2 mg, 0.025 mmol, 0.05 equiv.), BCMOM (37.2 mg, 0.05 mmol, 0.1 equiv.), cyclopentanone (88.9 μL, 1.0 mmol, 2 equiv.), H_2O_2 (35%) (129 μL, 1.5 mmol, 3 equiv.), acetonitrile (4 mL) and water (4 mL) at 80 ℃ under N_2 for 3 h. Column chromatography (PE/EA/TEA, from 10:1:0.1 to 10:3:0.1) afforded the title product as a yellow liquid (52.6 mg, 48%), unknown compound.

TLC: R_f = 0.35 (silica gel, PE/EA/TEA, 10:3:0.1).

^{1}H NMR (400 MHz, $CDCl_3$): δ 8.69 (d, *J* = 4.8 Hz, 1 H), 8.05 (s, 1 H), 7.36 (dd, *J* = 5.2, 1.6 Hz, 1 H), 4.01 (s, 3 H), 3.54–3.43 (m, 1 H), 2.73 (dd, *J* = 18.0, 7.6 Hz, 1 H), 2.57–2.47 (m, 2 H), 2.41–2.29 (m, 2 H), 2.07–1.96 (m, 1 H) ppm.

^{13}C NMR (100 MHz, $CDCl_3$): δ 216.2, 165.7, 153.4, 150.1, 148.3, 125.3, 123.6, 53.0, 44.6, 41.4, 38.5, 30.3 ppm.

HRMS (ESI) *m/z* calcd for $C_{12}H_{13}NO_3H^+$ $[M + H]^+$ 220.0968, found: 220.0965.

IR (KBr, cm^{-1}): ν_{max} 2960, 2920, 2847, 1735, 1576, 1398, 1369, 1289, 1128, 1027, 980, 766.

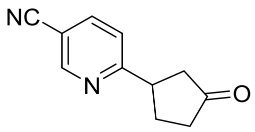

6-(3-Oxocyclopentyl)nicotinonitrile (91): *The General Procedure D* was applied with nicotinonitrile (52.6 mg, 0.5 mmol, 1 equiv.), H_2SO_4 (67.5 μL, 2.4 equiv.), $FeCl_2$ (3.2 mg, 0.025 mmol, 0.05 equiv.), BCMOM (37.2 mg, 0.05 mmol, 0.1 equiv.), cyclopentanone (88.9 μL, 1.0 mmol, 2 equiv.), H_2O_2 (35%) (129 μL, 1.5 mmol, 3 equiv.), acetonitrile (4 mL) and water (4 mL) at 80 ℃ under N_2 for 3 h. Column chromatography (PE/EA/TEA, from 10:3:0.1 to 10:5:0.1) afforded the title product as a yellow liquid (43.7 mg, 47%), known compound (CAS: 1391271-54-7).

TLC: R_f = 0.52 (silica gel, PE/EA/TEA, 10:5:0.1).

^{1}H NMR (400 MHz, $CDCl_3$): δ 8.83 (d, *J* = 1.2 Hz, 1 H), 7.91 (dd, *J* = 8.0, 2.0 Hz, 1 H), 7.34 (d, *J* = 8.0 Hz, 1 H), 3.70–3.59 (m, 1 H), 2.75–2.58 (m, 2 H), 2.55–2.39 (m, 2 H), 2.36–2.26 (m, 1 H), 2.19–2.11 (m, 1 H) ppm.

^{13}C NMR (100 MHz, $CDCl_3$): δ 217.3, 166.8, 152.3, 139.7, 122.2, 116.7, 108.0, 44.2, 44.0, 38.0, 30.0 ppm.

HRMS (ESI) *m/z* calcd for $C_{11}H_{10}N_2OH^+$ $[M + H]^+$ 187.0866, found: 187.0859.

IR (KBr, cm^{-1}): ν_{max} 3349, 2924, 2847, 2227, 1731, 1661, 1626, 1590, 1543, 1484, 1401, 1289, 1141, 1024, 852, 651, 563.

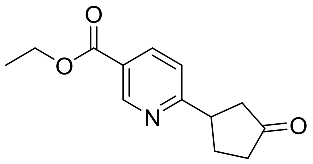

Ethyl 6-(3-oxocyclopentyl)nicotinate (92): *The General Procedure D* was applied with ethyl nicotinate (69.7 μL, 0.5 mmol, 1 equiv.), H_2SO_4 (67.5 μL, 2.4 equiv.), $FeCl_2$ (3.2 mg, 0.025 mmol, 0.05 equiv.), BCMOM (37.2 mg, 0.05 mmol, 0.1 equiv.), cyclopentanone (88.9 μL, 1.0 mmol, 2 equiv.), H_2O_2 (35%) (129 μL, 1.5 mmol, 3 equiv.), acetonitrile (4 mL) and water (4 mL) at 80 ℃ under N_2 for 3 h. Column chromatography (PE/EA/TEA, from 10:3:0.1 to 10:5:0.1) afforded the title product as a yellow liquid (57.9 mg, 50%), unknown compound.

TLC: R_f = 0.48 (silica gel, PE/EA/TEA, 10:5:0.1).

^{1}H NMR (400 MHz, $CDCl_3$): δ 9.16 (d, *J* = 2.0 Hz, 1 H), 8.24 (dd, *J* = 8.0, 2.0 Hz, 1 H), 7.28 (d, *J* = 8.0 Hz, 1 H), 4.40 (q, *J* = 10.6 Hz, 2 H), 3.67–3.56 (m, 1 H), 2.76–2.57 (m, 2 H), 2.56–2.39 (m, 2 H), 2.36–2.26 (m, 1 H), 2.22–2.11 (m, 1 H), 1.40 (t, *J* = 7.2 Hz, 3 H) ppm.

^{13}C NMR (100 MHz, $CDCl_3$): δ 218.1, 166.6, 165.2, 150.8, 137.7, 124.6, 121.6, 61.3, 44.3, 44.1, 38.2, 30.1, 14.3 ppm.

HRMS (ESI) *m/z* calcd for $C_{13}H_{15}NO_3H^+$ $[M + H]^+$ 234.1125, found: 234.1123.

IR (KBr, cm^{-1}): ν_{max} 2918, 2847, 1738, 1714, 1596, 1395, 1365, 1283, 1118, 1017.

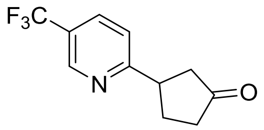

3-(5-(Trifluoromethyl)pyridin-2-yl)cyclopentan-1-one (93): *The General Procedure D* was applied with 3-(trifluoromethyl)pyridine (57.7 μL, 0.5 mmol, 1 equiv.), H_2SO_4 (67.5 μL, 2.4 equiv.), $FeCl_2$ (3.2 mg, 0.025 mmol, 0.05 equiv.), BCMOM (37.2 mg, 0.05 mmol, 0.1 equiv.), cyclopentanone (88.9 μL, 1.0 mmol, 2 equiv.), H_2O_2 (35%) (129 μL, 1.5 mmol, 3 equiv.), acetonitrile (4 mL) and water (4

mL) at 80 ℃ under N_2 for 3 h. Column chromatography (PE/EA/TEA, from 10:1:0.1 to 10:3:0.1) afforded the title product as a yellow liquid (59.6 mg, 52%), known compound (CAS: 1391222-38-0).

TLC: R_f = 0.40 (silica gel, PE/EA/TEA, 10:3:0.1).

^{1}H NMR (400 MHz, $CDCl_3$): δ 8.81 (s, 1 H), 7.87 (dd, J = 8.4, 2.4 Hz, 1 H), 7.33 (d, J = 8.4 Hz, 1 H), 3.69–3.59 (m, 1 H), 2.74–2.57 (m, 2 H), 2.56–2.40 (m, 2 H), 2.36–2.25 (m, 1 H), 2.22–2.13 (m, 1 H) ppm.

^{13}C NMR (100 MHz, $CDCl_3$): δ 217.8, 166.3, 146.5 (q, J = 4.0 Hz), 133.7 (q, J = 3.0 Hz),124.8 (q, J = 33.5 Hz), 123.5 (q, J = 270.5 Hz), 121.8, 44.2, 44.0, 38.1, 30.1 ppm.

^{19}F NMR (376 MHz, $CDCl_3$) δ -62.29 ppm.

HRMS (ESI) *m/z* calcd for $C_{11}H_{10}F_3NOH^+$ $[M + H]^+$ 230.0787, found: 230.0783.

IR (KBr, cm^{-1}): ν_{max} 2930, 2847, 1744, 1607, 1572, 1495, 1401, 1324, 1165, 1129, 1082, 1011, 852.

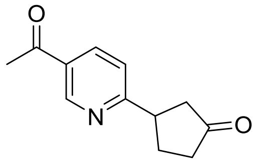

3-(5-acetylpyridin-2-yl)cyclopentan-1-one (**94**): *The General Procedure D* was applied with 1-(pyridin-3-yl)ethan-1-one (55.5 µL, 0.5 mmol, 1 equiv.), H_2SO_4 (67.5 µL, 2.4 equiv.), $FeCl_2$ (3.2 mg, 0.025 mmol, 0.05 equiv.), BCMOM (37.2 mg, 0.05 mmol, 0.1 equiv.), cyclopentanone (89.0 µL, 1.0 mmol, 2 equiv.), H_2O_2 (35%) (129 µL, 1.5 mmol, 3 equiv.), acetonitrile (4 mL) and water (4 mL) at 80 ℃ under N_2 for 3 h. Column chromatography (PE/EA/TEA, 2:1:0.1) afforded the title product as a black solid (40.6 mg, 40%), unknown compound.

TLC: R_f = 0.45 (silica gel, PE/EA/TEA, 2:1:0.1).

^{1}H NMR (400 MHz, $CDCl_3$) δ 9.10 (s, 1 H), 8.18 (d, J = 8.0 Hz, 1 H), 7.31 (d, J = 8.0 Hz, 1 H), 3.73–3.51 (m, 1 H), 2.75–2.63 (m, 2 H), 2.62 (s, 3 H), 2.55–2.39 (m, 2 H), 2.36–2.26 (m, 1 H), 2.22–2.11 (m, 1 H) ppm.

^{13}C NMR (100 MHz, $CDCl_3$) δ 217.9, 196.4, 167.0, 149.9, 136.1, 130.8, 122.0, 44.2, 44.1, 38.1, 30.1, 26.7 ppm.

HRMS (ESI) *m/z* calcd for $C_{12}H_{13}NO_2H^+$ $[M + H]^+$ 204.1019, found: 204.1012.

IR (KBr, cm^{-1}): ν_{max} 2968, 2927, 2850, 1752, 1681, 1593, 1398, 1363, 1280, 1156, 1121, 1029, 956, 855, 607.

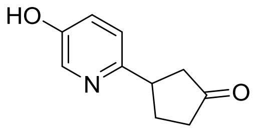

3-(5-hydroxypyridin-2-yl)cyclopentan-1-one (**95**)**:** *The General Procedure D* was applied with pyridin-3-ol (48.5 μL, 0.5 mmol, 1 equiv.), H_2SO_4 (67.5 μL, 2.4 equiv.), $FeCl_2$ (3.2 mg, 0.025 mmol, 0.05 equiv.), BCMOM (37.2 mg, 0.05 mmol, 0.1 equiv.), cyclopentanone (89.0 μL, 1.0 mmol, 2 equiv.), H_2O_2 (35%) (129 μL, 1.5 mmol, 3 equiv.), acetonitrile (4 mL) and water (4 mL) at 80 ℃ under N_2 for 3 h. Column chromatography (EA/TEA, 1:0.1) afforded the title product as a black solid (42.5 mg, 48%), known compound (CAS: 1391293-54-1).

TLC: R_f = 0.42 (silica gel, EA/TEA, 1:0.1).

^{1}H NMR (400 MHz, Acetone-d_6) δ 8.92 (br,1 H), 8.03 (d, *J* = 4.4 Hz, 1 H), 7.27 (d, *J* = 8.0 Hz, 1 H), 7.10 (dd, *J* = 8.0, 4.4 Hz, 1 H), 4.05–3.90 (m, 1 H), 2.67 (dd, *J* = 17.6, 8.4 Hz, 1 H), 2.40 (dd, *J* = 18.0, 7.6 Hz, 1 H), 2.36–2.24 (m, 2 H), 2.23–2.07 (m, 2 H). ppm

^{13}C NMR (100 MHz, Acetone-d_6) δ 218.0, 151.7, 151.0, 140.4, 123.2, 122.7, 43.2, 38.1, 37.8, 29.3 ppm.

HRMS (ESI) *m/z* calcd for $C_{10}H_{11}NO_2H^+$ $[M + H]^+$ 178.0863, found: 178.0860.

IR (KBr, cm^{-1}): ν_{max} 1734, 1569, 1457, 1333, 1297, 1256, 1221, 1179, 1138, 1091, 814, 761, 630.

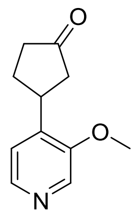

3-(3-Methoxypyridin-4-yl)cyclopentan-1-one (**96**)**:** *The General Procedure D* was applied with 3-methoxypyridine (53.5 μL, 0.5 mmol, 1 equiv.), H_2SO_4 (67.5 μL, 2.4 equiv.), $FeCl_2$ (3.2 mg, 0.025 mmol, 0.05 equiv.), BCMOM (37.2 mg, 0.05 mmol, 0.1 equiv.), cyclopentanone (88.9 μL, 1.0 mmol, 2 equiv.), H_2O_2 (35%) (129 μL, 1.5 mmol, 3 equiv.), acetonitrile (4 mL) and water (4 mL) at 80 ℃ under N_2 for 3 h. Column chromatography (PE/EA/TEA, from 10:1:0.1 to 10:3:0.1) afforded the title product as a brown liquid (40.1 mg, 42%), known compound (CAS: 1882679-81-3).

TLC: R_f = 0.48 (silica gel, PE/EA/TEA, 10:3:0.1).

^{1}H NMR (400 MHz, $CDCl_3$): δ 8.25 (s, 1 H), 8.24 (d, *J* = 4.8 Hz, 1 H), 7.12 (d, *J* = 4.8 Hz, 1 H), 3.94 (s, 3 H), 3.66 (m, 1 H), 2.66 (dd, *J* = 18.0, 7.2 Hz, 1 H), 2.46–2.25 (m, 4 H), 2.01 (m, 1 H) ppm.

^{13}C NMR (100 MHz, $CDCl_3$): δ 217.8, 153.8, 142.6, 140.1, 132.6, 121.2, 56.0, 43.7, 38.4, 36.1, 28.5 ppm.

HRMS (ESI) *m/z* calcd for $C_{11}H_{13}NO_2H^+$ $[M + H]^+$ 192.1019, found: 192.1018.

IR (KBr, cm^{-1}): ν_{max} 2919, 2847, 1744, 1596, 1502, 1461, 1412, 1307, 1266, 1141, 1017, 829.

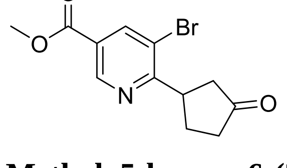

Methyl 5-bromo-6-(3-oxocyclopentyl)nicotinate (**97**)**:** *The General Procedure D* was applied with methyl 5-bromonicotinate (110.2 μL, 0.5 mmol, 1 equiv.), H_2SO_4 (67.5 μL, 2.4 equiv.), $FeCl_2$ (3.2 mg, 0.025 mmol, 0.05 equiv.), BCMOM (37.2 mg, 0.05 mmol, 0.1 equiv.), cyclopentanone (88.9 μL, 1.0 mmol, 2 equiv.), H_2O_2 (35%)

(129 μL, 1.5 mmol, 3 equiv.), acetonitrile (4 mL) and water (4 mL) at 80 ℃ under N_2 for 3 h. Column chromatography (PE/EA/TEA, from 10:1:0.1 to 10:3:0.1) afforded the title product as a yellow liquid (81.7 mg, 55%), unknown compound.

TLC: R_f = 0.47 (silica gel, PE/EA/TEA, 10:3:0.1).

^{1}H NMR (400 MHz, $CDCl_3$): δ 9.04 (d, J = 1.6 Hz, 1 H), 8.44 (d, J = 1.6 Hz, 1 H), 4.16–4.07 (m, 1 H), 3.95 (s, 3 H), 2.85–2.75 (m, 1 H), 2.60-2.53 (m, 1 H), 2.51–2.39 (m, 2 H), 2.29 (m, 1 H), 2.20–2.09 (m, 1 H) ppm.

^{13}C NMR (100 MHz, $CDCl_3$): δ 218.0, 164.6, 164.5, 148.7, 141.1, 125.5, 120.8, 52.7, 43.5, 42.2, 37.6, 28.8 ppm.

HRMS (ESI) m/z calcd for $C_{12}H_{12}BrNO_3H^+$ $[M + H]^+$ 298.0074, found: 298.0066.

IR (KBr, cm^{-1}): ν_{max} 2960, 2919, 2847, 1725, 1584, 1431, 1389, 1271, 1124, 1041, 964, 764.

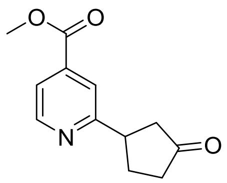

Methyl 2-(3-oxocyclopentyl)isonicotinate (**98**)**:** *The General Procedure D* was applied with methyl isonicotinate (60.3 μL, 0.5 mmol, 1 equiv.), H_2SO_4 (67.5 μL, 2.4 equiv.), $FeCl_2$ (3.2 mg, 0.025 mmol, 0.05 equiv.), BCMOM (37.2 mg, 0.05 mmol, 0.1 equiv.), cyclopentanone (88.9 μL, 1.0 mmol, 2 equiv.), H_2O_2 (35%) (129 μL, 1.5 mmol, 3 equiv.), acetonitrile (4 mL) and water (4 mL) at 80 ℃ under N_2 for 3 h. Column chromatography (PE/EA/TEA, from 10:3:0.1 to 10:5:0.1) afforded the title product as a yellow liquid (47.1 mg, 43%), known compound (CAS: 1391221-78-5).

TLC: R_f = 0.52 (silica gel, PE/EA/TEA, 10:5:0.1).

^{1}H NMR (400 MHz, $CDCl_3$): δ 8.70 (d, J = 4.8 Hz, 1 H), 7.76 (s, 1 H), 7.70 (dd, J = 4.8, 1.6 Hz, 1 H), 3.96 (s, 3 H), 3.69–3.59 (m, 1 H), 2.75–2.58 (m, 2 H), 2.55–2.40 (m, 2 H), 2.37–2.26 (m, 1 H), 2.21–2.11 (m, 1 H) ppm.

^{13}C NMR (100 MHz, $CDCl_3$): δ 218.2, 165.6, 163.5, 150.3, 137.9, 121.2, 121.0, 52.7, 44.4, 44.0, 38.2, 30.2 ppm.

HRMS (ESI) *m/z* calcd for $C_{12}H_{13}NO_3H^+$ $[M + H]^+$ 220.0968, found: 220.0968.

IR (KBr, cm^{-1}): ν_{max} 2960, 2924, 2847, 1744, 1560, 1431, 1401, 1289, 1195, 1159, 1118, 982, 758, 681.

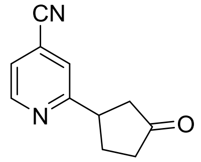

2-(3-Oxocyclopentyl)isonicotinonitrile (99): *The General Procedure D* was applied with isonicotinonitrile (52.6 mg, 1 equiv., 0.5 mmol), H_2SO_4 (67.5 μL, 2.4 equiv.), $FeCl_2$ (3.2 mg, 0.025 mmol, 0.05 equiv.), BCMOM (37.2 mg, 0.05 mmol, 0.1 equiv.), cyclopentanone (88.9 μL, 2 equiv., 1.0 mmol), H_2O_2 (35%) (129 μL, 1.5 mmol, 3 equiv.), acetonitrile (4 mL) and water (4 mL) at 80 ℃ under N_2 for 3 h. Column chromatography (PE/EA/TEA, from 10:3:0.1 to 10:6:0.1) afforded the title product as a white solid (40.0 mg, 43%), unknown compound.

TLC: R_f = 0.48 (silica gel, PE/EA/TEA, 10:6:0.1).

^{1}H NMR (400 MHz, $CDCl_3$): δ 8.73 (d, *J* = 4.8 Hz, 1 H), 7.44 (s, 1 H), 7.39 (dd, *J* = 4.8, 1.2 Hz, 1 H), 3.66–3.56 (m, 1 H), 2.71–2.56 (m, 2 H), 2.54–2.39 (m, 2 H), 2.35–2.25 (m, 1 H), 2.20–2.10 (m, 1 H) ppm.

^{13}C NMR (100 MHz, $CDCl_3$): δ 217.4, 164.1, 150.5, 123.7, 123.2, 120.8, 116.4, 44.1, 43.8, 38.0, 30.0 ppm.

HRMS (ESI) *m/z* calcd for $C_{11}H_{10}NO_2H^+$ $[M + H]^+$ 187.0866, found: 187.0863.

IR (KBr, cm^{-1}): ν_{max} 3054, 2960, 2233, 1738, 1596, 1543, 1472, 1407, 1289, 1236, 1171, 1135, 840.

Mp: 66.4-68.2 ℃.

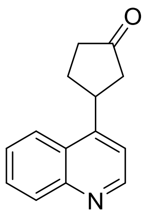

3-(Quinolin-4-yl)cyclopentan-1-one (100): *The General Procedure D* was applied with quinoline (60.0 μL, 1 equiv., 0.5 mmol), H_2SO_4 (67.5 μL, 2.4 equiv.), $FeCl_2$ (3.2 mg, 0.025 mmol, 0.05 equiv.), BCMOM (37.2 mg, 0.05 mmol, 0.1 equiv.), cyclopentanone (88.9 μL, 2 equiv., 1.0 mmol), H_2O_2 (35%) (129 μL, 1.5 mmol, 3 equiv.), acetonitrile (4 mL) and water (4 mL) at 80 ℃ under N_2 for 3 h. Column chromatography (PE/EA/TEA, from 10:2:0.1 to 10:5:0.1) afforded the title product as a brown liquid (47.5 mg, 45%), unknown compound.

TLC: R_f = 0.45 (silica gel, PE/EA/TEA, 10:5:0.1).

^{1}H NMR (400 MHz, $CDCl_3$): δ 8.87 (d, *J* = 4.4 Hz, 1 H), 8.15 (d, *J* = 8.8 Hz, 1 H), 8.08 (d, *J* = 8.4 Hz, 1 H), 7.74 (t, *J* = 8.0 Hz, 1 H), 7.61 (t, *J* = 8.4 Hz, 1 H), 7.27 (d, *J* = 5.2 Hz, 1 H), 4.25–4.16 (m, 1 H), 2.82 (dd, *J* = 18.0, 7.6 Hz, 1 H), 2.59–2.39 (m, 4 H), 2.20–2.15 (m, 1 H) ppm.

^{13}C NMR (100 MHz, $CDCl_3$): δ 217.0, 150.2, 148.6, 148.3, 130.5, 129.3, 127.0, 126.8, 122.9, 116.9, 44.8, 38.0, 37.1, 29.6 ppm.

HRMS (ESI) *m/z* calcd for $C_{14}H_{13}NOH^+$ $[M + H]^+$ 212.1070, found: 212.1072.

IR (KBr, cm^{-1}): ν_{max} 3476, 3063, 2974, 2903, 1752, 1587, 1510, 1410, 1162, 843, 767, 624, 489, 436.

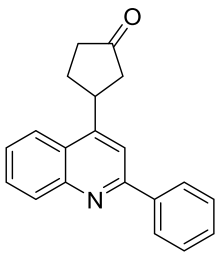

3-(2-Phenylquinolin-4-yl)cyclopentan-1-one (101): *The General Procedure D* was applied with 2-phenylquinoline (105.8 mg, 0.5 mmol, 1 equiv.), H_2SO_4 (67.5 μL, 2.4 equiv.), $FeCl_2$ (3.2 mg, 0.025 mmol, 0.05 equiv.), BCMOM (37.2 mg, 0.05 mmol, 0.1 equiv.), cyclopentanone (88.9 μL, 1.0 mmol, 2 equiv.), H_2O_2 (35%) (129 μL, 1.5 mmol, 3 equiv.), acetonitrile (4 mL) and water (4 mL) at 80 ℃ under N_2 for 3 h. Column chromatography (PE/EA/TEA, from 10:0.5:0.1 to 10:2:0.1) afforded the title product as a yellow liquid (74.7 mg, 52%), known compound (CAS: 2307617-32-7).

TLC: R_f = 0.42 (silica gel, PE/EA/TEA, 10:2:0.1).

^{1}H NMR (400 MHz, $CDCl_3$): δ 8.24 (d, *J* = 8.4 Hz, 1 H), 8.14 (d, *J* = 6.8 Hz, 2 H), 8.07 (d, *J* = 8.4 Hz, 1 H), 7.78–7.72 (m, 2 H), 7.58 (d, *J* = 7.2 Hz, 1 H), 7.53 (t, *J* = 7.6 Hz, 2 H), 7.47 (t, *J* = 7.2 Hz, 1 H), 4.27–4.18 (m, 1 H), 2.86 (dd, *J* = 18.0, 7.2 Hz, 1 H), 2.65–2.56 (m, 2 H), 2.54–2.40 (m, 2 H), 2.31–2.21 (m, 1 H) ppm.

^{13}C NMR (100 MHz, $CDCl_3$): δ 217.1, 157.3, 149.0, 148.6, 139.6, 130.8, 129.5, 129.4, 128.8, 127.5, 126.4, 125.9, 122.7, 114.9, 44.9, 38.1, 37.4, 29.7 ppm.

HRMS (ESI) *m/z* calcd for $C_{20}H_{17}NOH^+$ $[M + H]^+$ 288.1383, found: 288.1376.

IR (KBr, cm^{-1}): ν_{max} 3065, 2924, 2847, 1738, 1590, 1543, 1495, 1342, 1148, 769, 693.

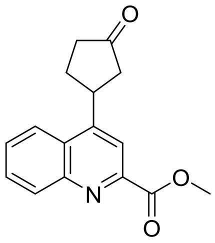

Methyl 4-(3-oxocyclopentyl)quinoline-2-carboxylate (102): *The General Procedure D* was applied with methyl quinoline-2-carboxylate (95.5 mg, 0.5 mmol, 1 equiv.), H_2SO_4 (67.5 μL, 2.4 equiv.), $FeCl_2$ (3.2 mg, 0.025 mmol, 0.05 equiv.), BCMOM (37.2 mg, 0.05 mmol, 0.1 equiv.), cyclopentanone (88.9 μL, 1.0 mmol, 2 equiv.), H_2O_2 (35%) (129 μL, 1.5 mmol, 3 equiv.), acetonitrile (4 mL) and water (4 mL) at 80 ℃

under N_2 for 3 h. Column chromatography (PE/EA/TEA, from 10:4:0.1 to 10:6:0.1) afforded the title product as a yellow liquid (94.2 mg, 70%), unknown compound.

TLC: R_f = 0.52 (silica gel, PE/EA/TEA, 10:6:0.1).

^{1}H NMR (400 MHz, $CDCl_3$): δ 8.35 (d, *J* = 8.4 Hz, 1 H), 8.13 (d, *J* = 7.2 Hz, 2 H), 7.80 (t, *J* = 7.6 Hz, 1 H), 7.72 (t, *J* = 7.2 Hz, 1 H), 4.27–4.17 (m, 1 H), 4.11–4.06 (s, 3 H), 2.85 (dd, *J* = 18.4, 7.2 Hz, 1 H), 2.59–2.41 (m, 4 H), 2.31–2.21 (m, 1 H) ppm.

^{13}C NMR (100 MHz, $CDCl_3$): δ 216.3, 166.0, 150.2, 147.7, 138.9, 131.8, 130.1, 128.9, 128.2, 122.8, 116.8, 53.3, 44.9, 38.3, 37.5, 29.6 ppm.

HRMS (ESI) *m/z* calcd for $C_{16}H_{15}NO_3H^+$ $[M + H]^+$ 270.1125, found: 270.1120.

IR (KBr, cm^{-1}): ν_{max} 2953, 2919, 2853, 1738, 1590, 1502, 1466, 1436, 1342, 1259, 1247, 1212, 1148, 1106, 994, 882, 787, 764.

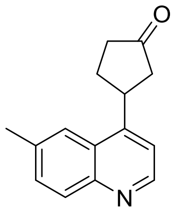

3-(6-Methylquinolin-4-yl)cyclopentan-1-one (**103**): *The General Procedure D* was applied with 6-methylquinoline (68.0 μL, 0.5 mmol, 1 equiv.), H_2SO_4 (67.5 μL, 2.4 equiv.), $FeCl_2$ (3.2 mg, 0.025 mmol, 0.05 equiv.), BCMOM (37.2 mg, 0.05 mmol, 0.1 equiv.), cyclopentanone (88.9 μL, 1.0 mmol, 2 equiv.), H_2O_2 (35%) (129 μL, 1.5 mmol, 3 equiv.), acetonitrile (4 mL) and water (4 mL) at 80 ℃ under N_2 for 3 h. Column chromatography (PE/EA/TEA, from 10:2:0.1 to 10:5:0.1) afforded the title product as a brown liquid (39.4 mg, 35%), unknown compound.

TLC: R_f = 0.45 (silica gel, PE/EA/TEA, 10:5:0.1).

^{1}H NMR (400 MHz, $CDCl_3$): δ 8.80 (d, *J* = 4.4 Hz, 1 H), 8.06 (d, *J* = 8.4 Hz, 1 H), 7.82 (s, 1 H), 7.58 (dd, *J* = 8.4, 1.2 Hz, 1 H), 7.24 (d, *J* = 4.4 Hz, 1 H), 4.23–4.14 (m, 1 H), 2.82 (dd, *J* = 18.0, 7.2 Hz, 1 H), 2.58 (s, 3 H), 2.56–2.53 (m, 1 H), 2.48 (m, 3 H), 2.21–2.12 (m, 1 H) ppm.

^{13}C NMR (100 MHz, $CDCl_3$): δ 217.1, 149.1, 148.1, 146.6, 136.8, 131.6, 129.9, 127.0, 121.8, 116.9, 44.9, 38.1, 37.1, 29.6, 22.1 ppm.

HRMS (ESI) *m/z* calcd for $C_{15}H_{13}NOH^+$ $[M + H]^+$ 226.1227, found: 226.1219.

IR (KBr, cm^{-1}): ν_{max} 2960, 2912, 1738, 1584, 1508, 1436, 1395, 1342, 1142, 858, 504.

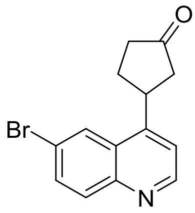

3-(6-Bromoquinolin-4-yl)cyclopentan-1-one (**104**): *The General Procedure D* was applied with 6-bromoquinoline (70.0 μL, 0.5 mmol, 1 equiv.), H_2SO_4 (67.5 μL, 2.4 equiv.), $FeCl_2$ (3.2 mg, 0.025 mmol, 0.05 equiv.), BCMOM (37.2 mg, 0.05 mmol, 0.1 equiv.), cyclopentanone (88.9 μL, 1.0 mmol, 2 equiv.), H_2O_2 (35%) (129 μL, 1.5 mmol, 3 equiv.), acetonitrile (4 mL) and water (4 mL) at 80 ℃ under N_2 for 3 h. Column chromatography (PE/EA/TEA, from 10:2:0.1 to 10:5:0.1) afforded the title product as a brown liquid (62.1 mg, 43%), unknown compound.

TLC: R_f = 0.35 (silica gel, PE/EA/TEA, 10:5:0.1).

^{1}H NMR (400 MHz, Acetone-d_6): δ 8.89 (d, J = 4.8 Hz, 1 H), 8.52 (d, J = 2.0 Hz, 1 H), 8.01 (d, J = 8.8 Hz, 1 H), 7.87 (dd, J = 9.2, 2.4 Hz, 1 H), 7.56 (d, J = 4.4 Hz, 1 H), 4.40–4.32 (m, 1 H), 2.75 (dd, J = 18.0, 8.0 Hz, 1H), 2.63–2.53 (m, 1 H), 2.52–2.40 (m, 3 H), 2.24–2.13 (m, 1 H) ppm.

^{13}C NMR (100 MHz, Acetone-d_6): δ 216.2, 151.8, 149.6, 148.0, 133.1, 133.1, 129.4, 126.8, 121.0, 119.1, 45.2, 38.7, 37.6, 30.6 ppm.

HRMS (ESI) *m/z* calcd for $C_{14}H_{12}BrNOH^+$ $[M + H]^+$ 290.0175, found: 290.0165.

IR (KBr, cm^{-1}): ν_{max} 2966, 1744, 1590, 1484, 1448, 1401, 1342, 1242, 1159, 1065, 1024, 840, 728, 604, 498, 427.

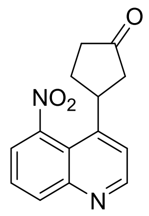

3-(5-Nitroquinolin-4-yl)cyclopentan-1-one (105): *The General Procedure D* was applied with 5-nitroquinoline (88.9 mg, 0.5 mmol, 1 equiv.), H_2SO_4 (67.5 μL, 2.4 equiv.), $FeCl_2$ (3.2 mg, 0.025 mmol, 0.05 equiv.), BCMOM (37.2 mg, 0.05 mmol, 0.1 equiv.), cyclopentanone (88.9 μL, 1.0 mmol, 2 equiv.), H_2O_2 (35%) (129 μL, 1.5 mmol, 3 equiv.), acetonitrile (4 mL) and water (4 mL) at 80 ℃ under N_2 for 3 h. Column chromatography (PE/EA/TEA, from 10:1:0.1 to 10:3:0.1) afforded the title product as a brown solid (44.8 mg, 35%), unknown compound.

TLC: R_f = 0.38 (silica gel, PE/EA/TEA, 10:3:0.1).

^{1}H NMR (400 MHz, $CDCl_3$): δ 8.96 (d, *J* = 8.8 Hz, 1 H), 8.34 (d, *J* = 8.0 Hz, 2 H), 7.78 (t, *J* = 8.0 Hz, 1 H), 7.58 (d, *J* = 8.8 Hz, 1 H), 3.88–3.76 (m, 1 H), 2.92 (dd, *J* = 18.0, 8.8 Hz, 1 H), 2.69 (dd, *J* = 18.0, 7.6 Hz, 1 H), 2.59–2.49 (m, 2 H), 2.42–2.20 (m, 2 H) ppm.

^{13}C NMR (100 MHz, $CDCl_3$): δ 218.1, 164.1, 147.9, 145.4, 136.3, 132.6, 127.6, 124.2, 123.5, 120.0, 44.2, 43.7, 38.0, 30.0 ppm.

HRMS (ESI) *m/z* calcd for $C_{14}H_{12}N_2O_3H^+$ $[M + H]^+$ 257.0921, found: 257.0913.

IR (KBr, cm^{-1}): ν_{max} 2960, 2924, 2842, 1738, 1596, 1525, 1401, 1336, 1159, 1129, 822, 788, 734.

Mp: 128.5-130.2 ℃.

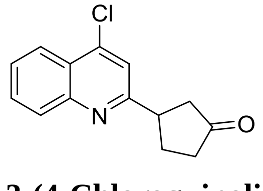

3-(4-Chloroquinolin-2-yl)cyclopentan-1-one (106): *The General Procedure D* was applied with 4-chloroquinoline (65.5 μL, 0.5 mmol, 1 equiv.), H_2SO_4 (67.5 μL, 2.4

equiv.), $FeCl_2$ (3.2 mg, 0.025 mmol, 0.05 equiv.), BCMOM (37.2 mg, 0.05 mmol, 0.1 equiv.), cyclopentanone (88.9 μL, 1.0 mmol, 2 equiv.), H_2O_2 (35%) (129 μL, 1.5 mmol, 3 equiv.), acetonitrile (4 mL) and water (4 mL) at 80 ℃ under N_2 for 3 h. Column chromatography (PE/EA/TEA, from 10:1:0.1 to 10:3:0.1) afforded the title product as brown liquid (47.8 mg, 39%), unknown compound.

TLC: R_f = 0.45 (silica gel, PE/EA/TEA, 10:3:0.1).

^{1}H NMR (400 MHz, $CDCl_3$): δ 8.19 (d, *J* = 8.4 Hz, 1 H), 8.04 (d, *J* = 8.4 Hz, 1 H), 7.75 (t, *J* = 6.8 Hz, 1 H), 7.60 (t, *J* = 7.2 Hz, 1 H), 7.44 (s, 1 H), 3.77–3.67 (m, 1 H), 2.88 (dd, *J* = 18.0, 8.8 Hz, 1 H), 2.66 (dd, *J* = 18.0, 7.6 Hz, 1 H), 2.58–2.46 (m, 2 H), 2.39–2.19 (m, 2 H) ppm.

^{13}C NMR (100 MHz, $CDCl_3$): δ 218.2, 162.5, 148.6, 142.9, 130.5, 129.4, 127.1, 125.2, 123.9, 120.4, 44.3, 43.9, 38.1, 30.0 ppm.

HRMS (ESI) *m/z* calcd for $C_{14}H_{12}ClNOH^+$ $[M + H]^+$ 246.0680, found: 246.0670.

IR (KBr, cm^{-1}): ν_{max} 3065, 2960, 2919, 2906, 1744, 1584, 1543, 1495, 1401, 1277, 1148, 1124, 953, 863, 764.

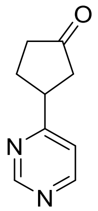

3-(Pyrimidin-4-yl)cyclopentan-1-one (**107**)**:** *The General Procedure D* was applied with pyrimidine (39.6 μL, 0.5 mmol, 1 equiv.), H_2SO_4 (67.5 μL, 2.4 equiv.), $FeCl_2$ (3.2 mg, 0.025 mmol, 0.05 equiv.), BCMOM (37.2 mg, 0.05 mmol, 0.1 equiv.), cyclopentanone (88.9 μL, 1.0 mmol, 2 equiv.), H_2O_2 (35%) (129 μL, 1.5 mmol, 3 equiv.), acetonitrile (4 mL) and water (4 mL) at 80 ℃ under N_2 for 3 h. Column chromatography (PE/EA/TEA, from 10:4:0.1 to 10:6:0.1) afforded the title product as a yellow liquid (48.3 mg, 55%), unknown compound.

TLC: R_f = 0.45 (silica gel, PE/EA/TEA, 10:6:0.1).

^{1}H NMR (400 MHz, $CDCl_3$) δ 9.16 (s, 1 H), 8.66 (d, *J* = 5.2 Hz, 1 H), 7.24 (dd, *J* = 5.2, 1.2 Hz, 1 H), 3.53 (m, 1 H), 2.72–2.57 (m, 2 H), 2.56–2.40 (m, 2 H), 2.36–2.26 (m, 1 H), 2.23–2.12 (m, 1 H) ppm.

^{13}C NMR (100 MHz, $CDCl_3$): δ 217.3, 170.8, 158.9, 157.1, 119.7, 43.6, 43.5, 38.0, 29.6 ppm.

HRMS (ESI) *m/z* calcd for $C_9H_{10}N_2OH^+$ $[M + H]^+$ 163.0866, found: 163.0868.

IR (KBr, cm^{-1}): ν_{max} 3370, 2920, 2850, 1658, 1628, 1469, 1415, 1250, 1097, 1050, 802.

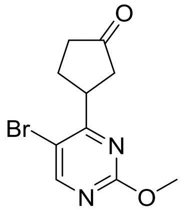

3-(5-Bromo-2-methoxypyrimidin-4-yl)cyclopentan-1-one (**108**): *The General Procedure D* was applied with 5-bromo-2-methoxypyrimidine (96.4 mg, 0.5 mmol, 1 equiv.), H_2SO_4 (67.5 μL, 2.4 equiv.), $FeCl_2$ (3.2 mg, 0.025 mmol, 0.05 equiv.), BCMOM (37.2 mg, 0.05 mmol, 0.1 equiv.), cyclopentanone (88.9 μL, 1.0 mmol, 2 equiv.), H_2O_2 (35%) (129 μL, 1.5 mmol, 3 equiv.), acetonitrile (4 mL) and water (4 mL) at 80 ℃ under N_2 for 3 h. Column chromatography (PE/EA/TEA, from 10:2:0.1 to 10:5:0.1) afforded the title product as a white liquid (64.8 mg, 48%), unknown compound.

TLC: R_f = 0.35 (silica gel, PE/EA/TEA, 10:5:0.1).

^{1}H NMR (400 MHz, Acetone-*d*$_6$): δ 8.60 (s, 1 H), 4.05–3.96 (m, 1 H), 3.92 (s, 3 H), 2.63 (dd, *J* = 18.0, 7.2 Hz, 1 H), 2.54–2.41 (m, 2 H), 2.38–2.23 (m, 2 H), 2.16–2.07 (m, 1 H) ppm.

^{13}C NMR (100 MHz, Acetone-*d*$_6$): δ 216.4, 171.9, 165.3, 161.5, 112.5, 55.4, 42.8, 42.2, 37.4, 28.7 ppm.

HRMS (ESI) *m/z* calcd for $C_{10}H_{11}BrN_2O_2H^+$ $[M + H]^+$ 271.0077, found: 271.0068.

IR (KBr, cm^{-1}): ν_{max} 3473, 2953, 1744, 1549, 1461, 1407, 1365, 1318, 1195, 1153, 1082, 1017, 958, 799, 670, 498.

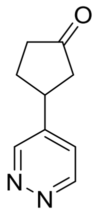

3-(Pyridazin-4-yl)cyclopentan-1-one (**109**): *The General Procedure D* was applied with pyridazine (36.6 μL, 0.5 mmol, 1 equiv.), H_2SO_4 (67.5 μL, 2.4 equiv.), $FeCl_2$ (3.2 mg, 0.025 mmol, 0.05 equiv.), BCMOM (37.2 mg, 0.05 mmol, 0.1 equiv.), cyclopentanone (88.9 μL, 1.0 mmol, 2 equiv.), H_2O_2 (35%) (129 μL, 1.5 mmol, 3 equiv.), acetonitrile (4 mL) and water (4 mL) at 80 ℃ under N_2 for 3 h. Column chromatography (PE/EA/TEA, from 10:7:0.1 to 10:10:0.1) afforded the title product as a black liquid (48.3 mg, 60%), unknown compound.

TLC: R_f = 0.43 (silica gel, PE/EA/TEA, 10:7:0.1).

^{1}H NMR (400 MHz, $CDCl_3$) δ 9.16 (s, 1 H), 9.14 (d, *J* = 5.6 Hz, 1 H), 7.35 (dd, *J* = 5.6, 2.4 Hz, 1 H), 3.45 (m, 1 H), 2.74 (dd, *J* = 18.0, 7.6 Hz, 1 H), 2.57–2.47 (m, 2 H), 2.43–2.29 (m, 2 H), 2.10–1.92 (m, 1 H) ppm.

^{13}C NMR (100 MHz, $CDCl_3$): δ 215.4, 151.4, 151.2, 142.3, 124.1, 44.0, 39.2, 38.3, 29.9 ppm.

HRMS (ESI) *m/z* calcd for $C_9H_{10}N_2OH^+$ $[M + H]^+$ 163.0866, found: 163.0862.

IR (KBr, cm^{-1}): ν_{max} 2924, 2842, 1744, 1708, 1584, 1395, 1283, 1129, 994, 858.

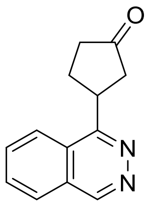

3-(Phthalazin-1-yl)cyclopentan-1-one (**110**): *The General Procedure D* was applied with phthalazine (65.2 mg, 0.5 mmol, 1 equiv.), H_2SO_4 (67.5 μL, 2.4 equiv.), $FeCl_2$

(3.2 mg, 0.025 mmol, 0.05 equiv.), BCMOM (37.2 mg, 0.05 mmol, 0.1 equiv.), cyclopentanone (88.9 μL, 1.0 mmol, 2 equiv.), H_2O_2 (35%) (129 μL, 1.5 mmol, 3 equiv.), acetonitrile (4 mL) and water (4 mL) at 80 ℃ under N_2 for 3 h. Column chromatography (PE/EA/TEA, from 1:1:0.1 to 1:2:0.1) afforded the title product as a black liquid (47.7 mg, 45%), unknown compound.

TLC: R_f = 0.52 (silica gel, PE/EA/TEA, 1:2:0.1).

^{1}H NMR (400 MHz, $CDCl_3$): δ 9.46 (s, 1 H), 8.18 (d, J = 8.4 Hz, 1 H), 8.03–7.91 (m, 3 H), 4.40 (m, 1H), 3.16 (dd, J = 18.4, 9.2 Hz, 1 H), 2.73 (dd, J = 18.4, 7.6 Hz, 1 H), 2.64–2.52 (m, 2 H), 2.51–2.35 (m, 2 H) ppm.

^{13}C NMR (100 MHz, $CDCl_3$): δ 217.6, 160.0, 150.7, 132.9, 132.2, 127.4, 126.6, 125.3, 123.1, 43.6, 38.4, 37.9, 29.3 ppm.

HRMS (ESI) *m/z* calcd for $C_{13}H_{11}N_2OH^+$ $[M + H]^+$ 213.1023, found: 213.1023.

IR (KBr, cm^{-1}): ν_{max} 2924, 2847, 1738, 1661, 1549, 1401, 1360, 1153, 758.

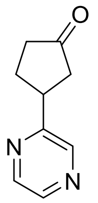

3-(Pyrazin-2-yl)cyclopentan-1-one (111): *The General Procedure D* was applied with pyrazine (40.5 mg, 0.5 mmol, 1 equiv.), H_2SO_4 (67.5 μL, 2.4 equiv.), $FeCl_2$ (3.2 mg, 0.025 mmol, 0.05 equiv.), BCMOM (37.2 mg, 0.05 mmol, 0.1 equiv.), cyclopentanone (88.9 μL, 1.0 mmol, 2 equiv.), H_2O_2 (35%) (129 μL, 1.5 mmol, 3 equiv.), acetonitrile (4 mL) and water (4 mL) at 80 ℃ under N_2 for 3 h. Column chromatography (PE/EA/TEA, from 10:1:0.1 to 10:6:0.1) afforded the title product as a brown liquid (45.4 mg, 56%), known compound (CAS: 1342003-29-5).

TLC: R_f = 0.42 (silica gel, PE/EA/TEA, 10:6:0.1).

^{1}H NMR (400 MHz, $CDCl_3$): δ 8.52 (s, 2 H), 8.45 (d, J = 1.2 Hz, 1 H), 3.67–3.57 (m, 1 H), 2.72–2.56 (m, 2 H), 2.51–2.43 (m, 2 H), 2.37–2.27 (m, 1 H), 2.21–2.13 (m, 1 H) ppm.

^{13}C NMR (100 MHz, $CDCl_3$): δ 217.7, 157.9, 144.3, 143.9, 143.1, 44.0, 41.3, 38.1, 30.0 ppm.

HRMS (ESI) *m/z* calcd for $C_9H_{10}NO_2H^+$ $[M + H]^+$ 163.0866, found: 163.0859.

IR (KBr, cm^{-1}): ν_{max} 2966, 2924, 2847, 1738, 1466, 1412, 1141, 1011, 858.

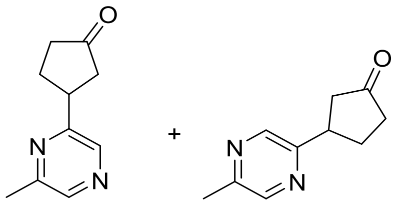

3-(6-Methylpyrazin-2-yl)cyclopentan-1-one (**112-1**) and **3-(5-Methylpyrazin-2-yl)cyclopentan-1-one** (**112-2**) (**112-1:112-2 = 3:2**): *The General Procedure D* was applied with 2-methylpyrazine (46.6 μL, 0.5 mmol, 1 equiv.), H_2SO_4 (67.5 μL, 2.4 equiv.), $FeCl_2$ (3.2 mg, 0.025 mmol, 0.05 equiv.), BCMOM (37.2 mg, 0.05 mmol, 0.1 equiv.), cyclopentanone (88.9 μL, 1.0 mmol, 2 equiv.), H_2O_2 (35%) (129 μL, 1.5 mmol, 3 equiv.), acetonitrile (4 mL) and water (4 mL) at 80 ℃ under N_2 for 3 h. Column chromatography (PE/EA/TEA, from 10:1:0.1 to 10:3:0.1) afforded the title product as a yellow liquid (43.1 mg, 49%), unknown compound.

TLC: R_f = 0.38 (silica gel, PE/EA/TEA, 10:3:0.1).

^{1}H NMR (400 MHz, $CDCl_3$): δ 8.39–8.33 (m, 2 H, minor), 8.32–8.28 (m, 2 H), 3.82–3.72 (m, 1 H), 3.61–3.53 (m, 1 H, minor), 2.77 (dd, *J* = 18.0, 8.0 Hz, 2 H), 2.71–2.66 (m, 2 H, minor), 2.63 (s, 3 H), 2.62–2.54 (m, 2 H), 2.52 (s, 3 H, minor), 2.51–2.43 (m, 2 H), 2.35–2.24 (m, 2 H), 2.19–2.10 (m, 2 H) ppm.

^{13}C NMR (100 MHz, $CDCl_3$): δ 218.2, 218.0 (minor), 156.0, 153.5 (minor), 151.7, 144.0 (minor), 142.7 (minor), 141.7, 141.5, 140.3 (minor), 44.1 (minor), 43.4, 41.3 (minor), 39.5, 38.2 (minor), 37.6, 30.0 (minor), 28.8, 21.5 (minor), 21.5 ppm.

HRMS (ESI) *m/z* calcd for $C_{10}H_{12}N_2OH^+$ $[M + H]^+$ 177.1023, found: 177.1016.

IR (KBr, cm^{-1}): ν_{max} 3048, 2966, 2924, 1738, 1536, 1461, 1412, 1236, 1148, 1030, 970, 846.

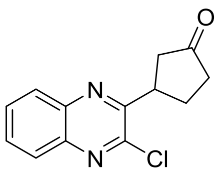

3-(3-Chloroquinoxalin-2-yl)cyclopentan-1-one (**113**): *The General Procedure D* was applied with 2-chloroquinoxaline (82.4 mg, 0.5 mmol, 1 equiv.), H_2SO_4 (67.5 μL, 2.4 equiv.), $FeCl_2$ (3.2 mg, 0.025 mmol, 0.05 equiv.), BCMOM (37.2 mg, 0.05 mmol, 0.1 equiv.), cyclopentanone (88.9 μL, 1.0 mmol, 2 equiv.), H_2O_2 (35%) (129 μL, 1.5 mmol, 3 equiv.), acetonitrile (4 mL) and water (4 mL) at 80 ℃ under N_2 for 3 h. Column chromatography (PE/EA/TEA, from 10:0.5:0.1 to 10:2:0.1) afforded the title product as a yellow liquid (39.3 mg, 32%), unknown compound.

TLC: R_f = 0.44 (silica gel, PE/EA/TEA, 10:2:0.1).

^{1}H NMR (400 MHz, $CDCl_3$): δ 8.05–7.98 (m, 2 H), 7.78–7.73 (m, 2 H), 4.28–4.20 (m, 1 H), 2.96 (dd, *J* = 18.0, 7.6 Hz, 1 H), 2.68 (dd, *J* = 18.4, 7.6 Hz, 1 H), 2.61–2.45 (m, 2 H), 2.41–2.24 (m, 2 H) ppm.

^{13}C NMR (100 MHz, $CDCl_3$): δ 217.7, 155.4, 147.0, 141.0, 140.6, 130.5, 130.3, 128.8, 128.1, 42.9, 40.4, 37.5, 28.5 ppm.

HRMS (ESI) *m/z* calcd for $C_{13}H_{11}ClN_2OH^+$ $[M + H]^+$ 247.0633, found: 247.0627.

IR (KBr, cm^{-1}): ν_{max} 2966, 2919, 2847, 1738, 1560, 1543, 1478, 1395, 1271, 1195, 1165, 1124, 1041, 923, 764, 599.

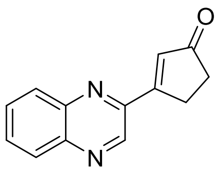

3-(Quinoxalin-2-yl)cyclopent-2-en-1-one (**114**): *The General Procedure D* was applied with quinoxaline (60.0 μL, 0.5 mmol, 1 equiv.), H_2SO_4 (67.5 μL, 2.4 equiv.), $FeCl_2$ (3.2 mg, 0.025 mmol, 0.05 equiv.), BCMOM (37.2 mg, 0.05 mmol, 0.1 equiv.), cyclopentanone (88.9 μL, 1.0 mmol, 2 equiv.), H_2O_2 (35%) (129 μL, 1.5 mmol, 3

equiv.), acetonitrile (4 mL) and water (4 mL) at 80 ℃ under N_2 for 3 h. Column chromatography (PE/EA/TEA, from 10:1:0.1 to 10:3:0.1) afforded the title product as a black solid (81.9 mg, 78%), unknown compound.

TLC: R_f = 0.35 (silica gel, PE/EA/TEA, 10:3:0.1).

^{1}H NMR (400 MHz, $CDCl_3$): δ 9.22 (s, 1 H), 8.15 (m, 2 H), 7.83 (m, 2 H), 7.01 (s, 1 H), 3.32 (dd, J = 4.8, 2.8 Hz, 2 H), 2.70–2.64 (m, 2 H) ppm.

^{13}C NMR (100 MHz, $CDCl_3$): δ 209.4, 170.9, 147.5, 143.6, 142.4, 142.2, 132.0, 131.2, 130.8, 130.1, 129.3, 35.2, 28.0 ppm.

HRMS (ESI) *m/z* calcd for $C_{13}H_{11}N_2OH^+$ $[M + H]^+$ 211.0866, found: 211.0857.

IR (KBr, cm^{-1}): ν_{max} 3048, 2924, 1714, 1674, 1590, 1495, 1436, 1365, 1253, 1176, 1129, 958, 876, 775.

Mp: 152.4-153.0 ℃.

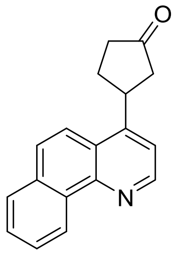

3-(Benzo[h]quinolin-4-yl)cyclopentan-1-one (115-1): *The General Procedure D* was applied with benzo[h]quinoline (91.4 mg, 0.5 mmol, 1 equiv.), H_2SO_4 (67.5 μL, 2.4 equiv.), $FeCl_2$ (3.2 mg, 0.025 mmol, 0.05 equiv.), BCMOM (37.2 mg, 0.05 mmol, 0.1 equiv.), cyclopentanone (88.9 μL, 1.0 mmol, 2 equiv.), H_2O_2 (35%) (129 μL, 1.5 mmol, 3 equiv.), acetonitrile (4 mL) and water (4 mL) at 80 ℃ under N_2 for 3 h. Column chromatography (PE/EA/TEA, from 10:0.1:0.1 to 10:1:0.1) afforded the title product as a yellow liquid (52.2 mg, 40%), unknown compound.

TLC: R_f = 0.25 (silica gel, PE/EA/TEA, 10:1:0.1).

^{1}H NMR (400 MHz, $CDCl_3$): δ 9.33 (d, *J* = 8.0 Hz, 1 H), 8.97 (d, *J* = 4.4 Hz, 1 H), 8.00–7.87 (m, 3 H), 7.79–7.69 (m, 2 H), 7.41 (d, *J* = 4.8 Hz, 1 H), 4.35–4.19 (m, 1 H), 2.85 (dd, *J* = 18.0, 7.6 Hz, 1 H), 2.68–2.38 (m, 4 H), 2.26–2.15 (m, 1 H) ppm.

^{13}C NMR (100 MHz, $CDCl_3$): δ 217.1, 148.6, 148.4, 146.6, 133.1, 131.8, 128.4, 128.1, 127.6, 127.3, 124.9, 124.8, 120.2, 117.7, 44.9, 38.1, 37.4, 29.9 ppm.

HRMS (ESI) *m/z* calcd for $C_{18}H_{15}NOH^+$ $[M + H]^+$ 262.1227, found: 262.1219.

IR (KBr, cm^{-1}): ν_{max} 3060, 2960, 2918, 2842, 1744, 1584, 1508, 1436, 1401, 1277, 1148, 829, 758.

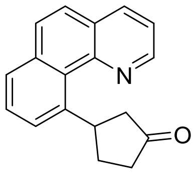

3-(Benzo[h]quinolin-10-yl)cyclopentan-1-one (115-2): *The General Procedure D* was applied with benzo[h]quinoline (91.4 mg, 0.5 mmol, 1 equiv.), H_2SO_4 (67.5 μL, 2.4 equiv.), $FeCl_2$ (3.2 mg, 0.025 mmol, 0.05 equiv.), BCMOM (37.2 mg, 0.05 mmol, 0.1 equiv.), cyclopentanone (88.9 μL, 1.0 mmol, 2 equiv.), H_2O_2 (35%) (129 μL, 1.5 mmol, 3 equiv.), acetonitrile (4 mL) and water (4 mL) at 80 ℃ under N_2 for 3 h. Column chromatography (PE/EA/TEA, from 10:0.1:0.1 to 10:1:0.1) afforded the title product as a yellow solid (32.6 mg, 25%), unknown compound.

TLC: R_f = 0.45 (silica gel, PE/EA/TEA, 10:1:0.1).

^{1}H NMR (400 MHz, $CDCl_3$): δ 9.24 (d, *J* = 7.6 Hz, 1 H), 8.13 (d, *J* = 8.0 Hz, 1 H), 7.88 (d, *J* = 6.8 Hz, 1 H), 7.78 (d, *J* = 8.8 Hz, 1 H), 7.75–7.65 (m, 3 H), 7.45 (d, *J* = 8.0 Hz, 1 H), 3.91–3.82 (m, 1 H), 3.05 (dd, *J* = 18.0, 7.2 Hz, 1 H), 2.72 (dd, *J* = 18.4, 8.0 Hz, 1 H), 2.66–2.51 (m, 2 H), 2.41–2.30 (m, 2 H) ppm.

^{13}C NMR (100 MHz, $CDCl_3$): δ 219.3, 161.4, 145.8, 136.4, 133.7, 131.3, 128.1, 127.7, 127.2, 127.0, 125.0, 124.8, 124.6, 120.8, 44.5, 44.2, 37.7, 30.5 ppm.

HRMS (ESI) *m/z* calcd for $C_{18}H_{15}NOH^+$ $[M + H]^+$ 262.1227, found: 262.1217.

IR (KBr, cm^{-1}): ν_{max} 3048, 2919, 2841, 1738, 1596, 1508, 1448, 1395, 1148, 1124, 1017, 846, 799, 752.

Mp: 59.0-60.3 ℃.

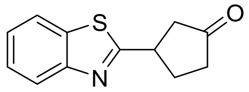

3-(Benzo[d]thiazol-2-yl)cyclopentan-1-one (**116**)**:** *The General Procedure D* was applied with benzo[d]thiazole (55.2 μL, 0.5 mmol, 1 equiv.), H_2SO_4 (67.5 μL, 2.4 equiv.), $FeCl_2$ (3.2 mg, 0.025 mmol, 0.05 equiv.), BCMOM (37.2 mg, 0.05 mmol, 0.1 equiv.), cyclopentanone (88.9 μL, 1.0 mmol, 2 equiv.), H_2O_2 (35%) (129 μL, 1.5 mmol, 3 equiv.), acetonitrile (4 mL) and water (4 mL) at 80 ℃ under N_2 for 3 h. Column chromatography (PE/EA/TEA, from 10:1:0.1 to 10:3:0.1) afforded the title product as a yellow solid (57.5 mg, 53%), known compound (CAS: 1198105-07-5).

TLC: R_f = 0.38 (silica gel, PE/EA/TEA, 10:3:0.1).

^{1}H NMR (400 MHz, $CDCl_3$): δ 7.98 (d, *J* = 8.0 Hz, 1 H), 7.86 (d, *J* = 8.0 Hz, 1 H), 7.47 (t, *J* = 7.6 Hz, 1 H), 7.37 (t, *J* = 7.6 Hz, 1 H), 3.97–3.86 (m, 1 H), 2.85–2.72 (m, 2 H), 2.64–2.49 (m, 2 H), 2.40–2.29 (m, 2 H) ppm.

^{13}C NMR (100 MHz, $CDCl_3$): δ 216.2, 172.9, 153.0, 134.7, 126.2, 125.0, 122.8, 121.6, 44.6, 41.0, 38.0, 30.3 ppm.

Mp: 81.9-82.6 ℃.

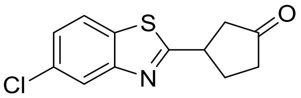

3-(5-Chlorobenzo[d]thiazol-2-yl)cyclopentan-1-one (**117**)**:** *The General Procedure D* was applied with 5-chlorobenzo[d]thiazole (78.0 mg, 0.5 mmol, 1 equiv.), H_2SO_4 (67.5 μL, 2.4 equiv.), $FeCl_2$ (3.2 mg, 0.025 mmol, 0.05 equiv.), BCMOM (37.2 mg, 0.05 mmol, 0.1 equiv.), cyclopentanone (88.9 μL, 1.0 mmol, 2 equiv.), H_2O_2 (35%) (129 μL, 1.5 mmol, 3 equiv.), acetonitrile (4 mL) and water (4 mL) at 80 ℃ under N_2

for 3 h. Column chromatography (PE/EA/TEA, from 10:0.5:0.1 to 10:2:0.1) afforded the title product as a brown solid (63.3 mg, 50%), unknown compound.

TLC: R_f = 0.30 (silica gel, PE/EA/TEA, 10:2:0.1).

^{1}H NMR (400 MHz, $CDCl_3$): δ 7.96 (d, J = 2.0 Hz, 1 H), 7.77 (d, J = 8.4 Hz, 1 H), 7.36 (dd, J = 8.4, 2.0 Hz, 1 H), 3.95–3.86 (m, 1 H), 2.84–2.69 (m, 2 H), 2.63–2.49 (m, 2 H), 2.41–2.28 (m, 2 H) ppm.

^{13}C NMR (100 MHz, $CDCl_3$): δ 216.0, 174.9, 153.9, 133.0, 132.2, 125.6, 122.8, 122.3, 44.5, 41.1, 37.9, 30.3 ppm.

HRMS (ESI) *m/z* calcd for $C_{12}H_{10}ClNOSH^+$ $[M - H]^+$ 252.0255, found: 252.0237.

IR (KBr, cm^{-1}): ν_{max} 2966, 2919, 2853, 1744, 1590, 1543, 1508, 1436, 1395, 1294, 1171, 1135, 1071, 923, 870, 799, 575.

Mp: 83.7-85.5 ℃.

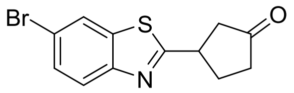

3-(6-Bromobenzo[d]thiazol-2-yl)cyclopentan-1-one (**118**): *The General Procedure D* was applied with 6-bromobenzo[d]thiazole (107.1 mg, 0.5 mmol, 1 equiv.), H_2SO_4 (67.5 μL, 2.4 equiv.), $FeCl_2$ (3.2 mg, 0.025 mmol, 0.05 equiv.), BCMOM (37.2 mg, 0.05 mmol, 0.1 equiv.), cyclopentanone (88.9 μL, 1.0 mmol, 2 equiv.), H_2O_2 (35%) (129 μL, 1.5 mmol, 3 equiv.), acetonitrile (4 mL) and water (4 mL) at 80 ℃ under N_2 for 3 h. Column chromatography (PE/EA/TEA, from 10:1:0.1 to 10:3:0.1) afforded the title product as a brown liquid (59.0 mg, 40%), unknown compound.

TLC: R_f = 0.47 (silica gel, PE/EA/TEA, 10:3:0.1).

^{1}H NMR (400 MHz, $CDCl_3$): δ 8.00 (d, J = 2.0 Hz, 1 H), 7.82 (d, J = 8.8 Hz, 1 H), 7.57 (dd, J = 8.8, 2.0 Hz, 1 H), 3.95–3.83 (m, 1 H), 2.87–2.69 (m, 2 H), 2.66–2.49 (m, 2 H), 2.41–2.29 (m, 2 H) ppm.

^{13}C NMR (100 MHz, $CDCl_3$): δ 216.0, 173.5, 151.9, 136.4, 129.7, 124.2, 124.0, 118.6, 44.4, 41.0, 37.9, 30.3 ppm.

HRMS (ESI) *m/z* calcd for $C_{12}H_{10}BrNOSH^+$ $[M - H]^+$ 295.9739, found: 295.9738.

IR (KBr, cm^{-1}): ν_{max} 2933, 2903, 1741, 1581, 1516, 1439, 1398, 1310, 1274, 1179, 1138, 814.

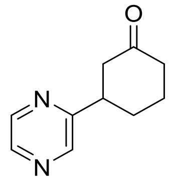

3-(Pyrazin-2-yl)cyclohexan-1-one (119): *The General Procedure D* was applied with pyrazine (39.6 mg, 0.5 mmol, 1 equiv.), H_2SO_4 (67.5 μL, 2.4 equiv.), $FeCl_2$ (3.2 mg, 0.025 mmol, 0.05 equiv.), BCMOM (37.2 mg, 0.05 mmol, 0.1 equiv.), cyclohexanone (104.7 μL, 1.0 mmol, 2 equiv.), H_2O_2 (35%) (129 μL, 1.5 mmol, 3 equiv.), acetonitrile (4 mL) and water (4 mL) at 80 ℃ under N_2 for 3 h. Column chromatography (PE/EA/TEA, from 1:1:0.1 to 1:2:0.1) afforded the title product as a yellow liquid (59.9 mg, 68%), known compound (CAS: 1340429-24-4).

TLC: R_f = 0.58 (silica gel, PE/EA/TEA, 1:2:0.1).

^{1}H NMR (400 MHz, $CDCl_3$): δ 8.53 (s, 1 H), 8.49–8.35 (m, 2 H), 3.30–3.21 (m, 1 H), 2.81 (t, *J* = 14.0 Hz, 1 H), 2.61–2.53 (m, 1 H), 2.48–2.38 (m, 2 H), 2.21–2.06 (m, 2 H), 2.01–1.82 (m, 2 H) ppm.

^{13}C NMR (100 MHz, $CDCl_3$): δ 210.4, 158.2, 144.2, 143.6, 143.1, 46.1, 43.6, 41.0, 31.3, 25.1 ppm.

HRMS (ESI) *m/z* calcd for $C_{10}H_{12}N_2OH^+$ $[M + H]^+$ 177.1023, found: 177.1019.

IR (KBr, cm^{-1}): ν_{max} 2927, 2862, 1711, 1404, 1227, 1132, 1020, 455.

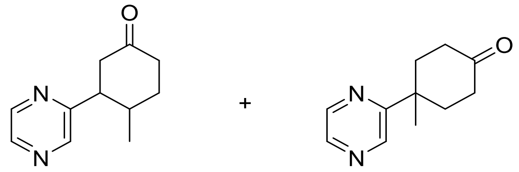

4-Methyl-3-(pyrazin-2-yl)cyclohexan-1-one (120-1) and 4-Methyl-4-(pyrazin-2-yl)cyclohexan-1-one (120-2) (120-1:120-2 = 3:1): *The General Procedure D* was applied with pyrazine (39.6 mg, 0.5 mmol, 1 equiv.), H_2SO_4 (67.5 μL, 2.4 equiv.), $FeCl_2$ (3.2 mg, 0.025 mmol, 0.05 equiv.), BCMOM (37.2 mg, 0.05 mmol, 0.1 equiv.), 4-methylcyclohexan-1-one (123.0 μL, 1.0 mmol, 2 equiv.), H_2O_2 (35%) (129 μL, 1.5 mmol, 3 equiv.), acetonitrile (4 mL) and water (4 mL) at 80 ℃ under N_2 for 3 h. Column chromatography (PE/EA/TEA, from 1:1:0.1 to 1:2:0.1) afforded the title product as a yellow liquid (47.5 mg, 50%), known compound (CAS: 2138092-33-6) and unknown compound.

TLC: R_f = 0.65 (silica gel, PE/EA/TEA, 1:2:0.1).

^{1}H NMR (400 MHz, $CDCl_3$): δ 8.76 (s, 1 H, minor), 8.58–8.54 (m, 1 H), 8.46 (d, *J* = 2.4 Hz, 1 H), 8.40 (d, *J* = 1.6 Hz, 1 H), 2.85–2.80 (m, 1 H), 2.75–2.66 (m, 1 H), 2.55–2.51 (m, 1 H), 2.46–2.38 (m, 2 H), 2.33–2.24 (m, 2 H), 2.18–2.12 (m, 1 H), 2.00–1.94 (m, 1 H), 1.89–1.80 (m, 1 H), 1.63–1.56 (m, 1 H), 1.37 (s, 3 H, minor), 0.78 (d, *J* = 6.4 Hz, 3 H) ppm.

^{13}C NMR (100 MHz, $CDCl_3$): δ 210.5, 161.0 (minor), 157.6, 144.6, 144.5, 143.8 (minor), 143.2, 142.4 (minor), 142.2 (minor), 50.3, 46.4, 41.0, 38.7 (minor), 38.2 (minor), 37.2 (minor), 36.2 (minor),35.6, 34.2, 29.9 (minor), 29.1 (minor), 19.1 ppm.

HRMS (ESI) *m/z* calcd for $C_{11}H_{14}N_2OH^+$ $[M + H]^+$ 191.1179, found: 191.1176.

IR (KBr, cm^{-1}): ν_{max} 2924, 2862, 1711, 1404, 1166, 1029, 876, 411.

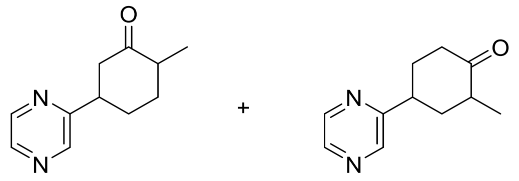

2-Methyl-5-(pyrazin-2-yl)cyclohexan-1-one (121-1) and 2-Methyl-4-(pyrazin-2-yl)cyclohexan-1-one (121-2) (121-1:121-2 = 10:9): *The General Procedure D* was applied with pyrazine (39.6 mg, 0.5 mmol, 1 equiv.), H_2SO_4 (67.5 μL, 2.4 equiv.), $FeCl_2$ (3.2 mg, 0.025 mmol, 0.05 equiv.), BCMOM (37.2

mg, 0.05 mmol, 0.1 equiv.), 2-methylcyclohexan-1-one (125.2 μL, 1.0 mmol, 2 equiv.), H_2O_2 (35%) (129 μL, 1.5 mmol, 3 equiv.), acetonitrile (4 mL) and water (4 mL) at 80 ℃ under N_2 for 3 h. Column chromatography (PE/EA/TEA, from 1:1:0.1 to 1:2:0.1) afforded the title product as a yellow liquid (47.5 mg, 50%), unknown compound.

TLC: R_f = 0.59 (silica gel, PE/EA/TEA, 1:2:0.1).

^{1}H NMR (400 MHz, $CDCl_3$): δ 8.75–8.63 (m, 1 H, minor), 8.57-8.55 (m, 1 H), 8.54–8.52 (m, 1 H, minor), 8.46 (d, *J* = 2.8 Hz, 2 H), 8.42 (d, *J* = 1.2 Hz, 1 H, minor), 3.20 (m, 1 H), 2.99–2.93 (m, 1 H), 2.86–2.76 (m, 2 H), 2.60–2.56 (m, 1 H), 2.54–2.48 (m, 2 H), 2.26–2.16 (m, 2 H), 2.12–2.03 (m, 3 H), 1.99–1.94 (m, 1 H), 1.72–1.67(m, 3 H), 1.09 (d, *J* = 6.4 Hz, 3 H, minor), 0.80 (d, *J* = 6.4 Hz, 3 H) ppm.

^{13}C NMR (100 MHz, $CDCl_3$): δ 212.0, 212.0, 158.0, 158.0, 147.8, 144.5, 144.4, 144.2, 143.6, 143.1, 51.5, 48.4, 46.5, 44.9, 44.6, 41.5, 34.8, 32.7, 31.9, 26.2, 14.4, 12.3 ppm.

HRMS (ESI) *m/z* calcd for $C_{11}H_{14}N_2OH^+$ $[M + H]^+$ 191.1179, found: 191.1172.

IR (KBr, cm^{-1}): ν_{max} 2920, 2862, 1717, 1404, 1145, 1020, 849, 400.

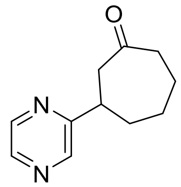

3-(Pyrazin-2-yl)cycloheptan-1-one (122): *The General Procedure D* was applied with pyrazine (39.6 mg, 0.5 mmol, 1 equiv.), H_2SO_4 (67.5 μL, 2.4 equiv.), $FeCl_2$ (3.2 mg, 0.025 mmol, 0.05 equiv.), BCMOM (37.2 mg, 0.05 mmol, 0.1 equiv.), cycloheptanone (119.1 μL, 1.0 mmol, 2 equiv.), H_2O_2 (35%) (129 μL, 1.5 mmol, 3 equiv.), acetonitrile (4 mL) and water (4 mL) at 80 ℃ under N_2 for 3 h. Column chromatography (PE/EA/TEA, from 10:3:0.1 to 10:5:0.1) afforded the title product as a yellow liquid (64.6 mg, 68%), known compound (CAS: 1343576-27-1).

TLC: R_f = 0.45 (silica gel, PE/EA/TEA, 10:5:0.1).

¹H NMR (400 MHz, $CDCl_3$): δ 8.48 (d, *J* = 2.4 Hz, 1 H), 8.47 (s, 1 H), 8.42 (d, *J* = 2.4 Hz, 1 H), 2.97–2.91 (m, 1 H), 2.73–2.64 (m, 2 H), 2.63–2.60 (m, 2 H), 2.21–2.13 (m, 1 H), 2.11–2.01 (m, 3 H), 1.85–1.76 (m, 2 H) ppm.

¹³C NMR (100 MHz, $CDCl_3$): δ 214.0, 160.9, 144.0, 143.2, 142.7, 47.5, 43.6, 42.4, 36.4, 29.7, 23.3 ppm.

HRMS (ESI) *m/z* calcd for $C_{11}H_{14}N_2OH^+$ $[M + H]^+$ 191.1179, found: 191.1174.

IR (KBr, cm^{-1}): ν_{max} 2927, 2862, 1705, 1410, 1156, 1020, 838, 401.

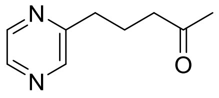

5-(pyrazin-2-yl)pentan-2-one (**123**): *The General Procedure D* was applied with pyrazine (39.6 μL, 0.5 mmol, 1 equiv.), H_2SO_4 (67.5 μL, 2.4 equiv.), $FeCl_2$ (3.2 mg, 0.025 mmol, 0.05 equiv.), BCMOM (37.2 mg, 0.05 mmol, 0.1 equiv.), pentan-2-one (107.4 μL, 1.0 mmol, 2 equiv.), H_2O_2 (35%) (129 μL, 1.5 mmol, 3 equiv.), acetonitrile (4 mL) and water (4 mL) at 80 ℃ under N_2 for 3 h. Column chromatography (EA/PE/TEA, 1:2:0.1) afforded the title product as a yellow liquid (27.1 mg, 33%), known compound (CAS: 146431-63-2).

TLC: R_f = 0.38 (silica gel, EA/PE/TEA, 1:1:0.1).

¹H NMR (400 MHz, $CDCl_3$) δ 8.49 (s, 1 H), 8.47 (s, 1 H), 8.42 (s, 1 H), 2.83 (t, *J* = 7.6 Hz, 2 H), 2.52 (t, *J* = 7.2 Hz, 2 H), 2.13 (s, 3 H), 2.09–1.96 (m, 2 H) ppm.

¹³C NMR (100 MHz, $CDCl_3$) δ 208. 2, 157.0, 144.5, 144.0, 142.3, 42.6, 34.3, 29.9, 23.0 ppm.

HRMS (ESI) *m/z* calcd for $C_9H_{12}N_2OH^+$ $[M + H]^+$ 165.1023, found: 165.1024.

IR (KBr, cm^{-1}): ν_{max} 2968, 2920, 2876, 1722, 1410, 1145, 1022, 402.

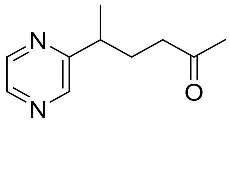

5-(Pyrazin-2-yl)hexan-2-one (124): *The General Procedure D* was applied with pyrazine (39.6 mg, 0.5 mmol, 1 equiv.), H_2SO_4 (67.5 μL, 2.4 equiv.), $FeCl_2$ (3.2 mg, 0.025 mmol, 0.05 equiv.), BCMOM (37.2 mg, 0.05 mmol, 0.1 equiv.), hexan-2-one (124.6 μL, 1.0 mmol, 2 equiv.), H_2O_2 (35%) (129 μL, 1.5 mmol, 3 equiv.), acetonitrile (4 mL) and water (4 mL) at 80 ℃ under N_2 for 3 h. Column chromatography (PE/EA/TEA, from 10:3:0.1 to 10:6:0.1) afforded the title product as a brown liquid (38.3 mg, 43%), unknown compound.

TLC: R_f = 0.48 (silica gel, PE/EA/TEA, 10:6:0.1).

^{1}H NMR (400 MHz, $CDCl_3$): δ 8.50 (s, 1 H), 8.44 (s, 1 H), 8.41 (d, *J* = 2.4 Hz, 1 H), 2.99–2.94 (m, 1 H), 2.38–2.29 (m, 2 H), 2.09 (s, 3 H), 2.03–1.91 (m, 2 H), 1.32 (d, *J* = 6.8 Hz, 3 H) ppm.

^{13}C NMR (100 MHz, $CDCl_3$): δ 208.3, 160.8, 144.1, 144.0, 142.5, 41.3, 38.6, 30.1, 29.9, 20.5 ppm.

HRMS (ESI) *m/z* calcd for $C_{10}H_{14}N_2OH^+$ $[M + H]^+$ 179.1179, found: 179.1178.

IR (KBr, cm^{-1}): ν_{max} 3370, 2968, 2920, 2856, 1711, 1658, 1410, 1363, 1162, 1014, 401.

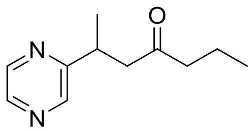

2-(Pyrazin-2-yl)heptan-4-one (125): *The General Procedure D* was applied with pyrazine (39.6 mg, 0.5 mmol, 1 equiv.), H_2SO_4 (67.5 μL, 2.4 equiv.), $FeCl_2$ (3.2 mg, 0.025 mmol, 0.05 equiv.), BCMOM (37.2 mg, 0.05 mmol, 0.1 equiv.), heptan-4-one (142.6 μL, 1.0 mmol, 2 equiv.), H_2O_2 (35%) (129 μL, 1.5 mmol, 3 equiv.), acetonitrile (4 mL) and water (4 mL) at 80 ℃ under N_2 for 3 h. Column chromatography (PE/EA/TEA, from 10:7:0.1 to 1:1:0.1) afforded the title product as a brown liquid (55.7 mg, 58%), unknown compound.

TLC: R_f = 0.48 (silica gel, PE/EA/TEA, 1:1:0.1).

¹H NMR (400 MHz, $CDCl_3$) δ 8.52 (s, 1 H), 8.44 (d, *J* = 1.2 Hz, 1 H), 8.38 (s, 1 H), 3.63–3.42 (m, 1 H), 3.07 (dd, *J* = 17.2, 7.6 Hz, 1 H), 2.69 (dd, *J* = 17.2, 5.6 Hz, 1 H), 2.35 (t, *J* = 7.6 Hz, 2 H), 1.59–1.46 (m, 2 H), 1.28 (dd, *J* = 7.0, 1.2 Hz, 3 H), 0.85 (td, *J* = 7.2, 1.2 Hz, 3 H) ppm.

¹³C NMR (100 MHz, $CDCl_3$) δ 209.4, 160.5, 144.4, 143.7, 142.3, 48.2, 45.1, 34.1, 20.8, 17.2, 13.6 ppm.

HRMS (ESI) *m/z* calcd for $C_{11}H_{16}N_2OH^+$ $[M + H]^+$ 193.1336, found: 193.1332.

IR (KBr, cm^{-1}): ν_{max} 2968, 2933, 2879, 1723, 1404, 1126, 1041, 412.

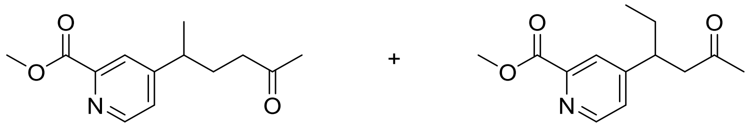

Methyl 4-(5-oxohexan-2-yl)picolinate (**126-1**) and **Methyl 4-(5-oxohexan-3-yl)picolinate** (**126-2**) **(126-1:126-2 = 2:1):** *The General Procedure D* was applied with methyl picolinate (61.5 μL, 0.5 mmol, 1 equiv.), H_2SO_4 (67.5 μL, 2.4 equiv.), $FeCl_2$ (3.2 mg, 0.025 mmol, 0.05 equiv.), BCMOM (37.2 mg, 0.05 mmol, 0.1 equiv.), hexan-2-one (124.6 μL, 1.0 mmol, 2 equiv.), H_2O_2 (35%) (129 μL, 1.5 mmol, 3 equiv.), acetonitrile (4 mL) and water (4 mL) at 80 ℃ under N_2 for 3 h. Column chromatography (PE/EA/TEA, from 10:5:0.1 to 10:9:0.1) afforded the title product as a yellow liquid (56.4 mg, 48%), unknown compound.

TLC: R_f = 0.55 (silica gel, PE/EA/TEA, 10:9:0.1).

¹H NMR (400 MHz, $CDCl_3$): δ 8.63 (d, *J* = 5.2 Hz, 1 H), 8.60 (d, *J* = 4.8 Hz, 1 H, minor), 7.95 (s, 1 H), 7.95 (s, 1 H, minor), 7.31–7.29 (m, 1 H, minor), 7.28 (dd, *J* = 4.8, 1.2 Hz, 1 H), 3.99 (s, 3 H), 3.99 (s, 3 H, minor), 2.81–2.75 (m, 2 H), 2.33–2.27 (m, 2 H), 2.07 (s, 3 H), 2.06 (s, 3 H, minor), 1.95–1.77 (m, 3 H), 1.72–1.54 (m, 2 H), 1.27 (d, *J* = 6.8 Hz, 3 H), 0.76 (t, *J* = 7.6 Hz, 3 H, minor) ppm.

¹³C NMR (100 MHz, $CDCl_3$): δ 207.9, 206.3 (minor), 165.9, 157.0, 155.2 (minor), 152.5 (minor), 149.9, 149.8 (minor), 149.7 (minor), 148.1, 126.8 (minor), 125.8,

124.0 (minor), 123.9, 52.9, 49.1 (minor), 41.7 (minor), 41.1, 38.7, 30.8, 30.5 (minor), 30.0, 29.6 (minor), 28.5 (minor), 21.4, 11.7 (minor) ppm.

HRMS (ESI) *m/z* calcd for $C_{13}H_{17}NO_3H^+$ $[M + H]^+$ 236.1281, found: 236.1278.

IR (KBr, cm^{-1}): ν_{max} 2963, 2944, 2873, 1723, 1593, 1445, 1304, 1209, 1121, 1091, 984, 796.

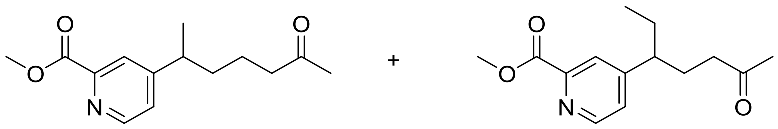

Methyl 4-(6-oxoheptan-2-yl)picolinate (**127-1**) and **Methyl 4-(6-oxoheptan-3-yl)picolinate** (**127-2**) **(127-1:127-2 = 2:1):** *The General Procedure D* was applied with methyl picolinate (61.5 μL, 0.5 mmol, 1 equiv.), H_2SO_4 (67.5 μL, 2.4 equiv.), $FeCl_2$ (3.2 mg, 0.025 mmol, 0.05 equiv.), BCMOM (37.2 mg, 0.05 mmol, 0.1 equiv.), heptan-2-one (140.7 μL, 1.0 mmol, 2 equiv.), H_2O_2 (35%) (129 μL, 1.5 mmol, 3 equiv.), acetonitrile (4 mL) and water (4 mL) at 80 ℃ under N_2 for 3 h. Column chromatography (PE/EA/TEA, from 10:4:0.1 to 10:8:0.1) afforded the title product as a yellow liquid (57.3 mg, 46%), unknown compound.

TLC: R_f = 0.48 (silica gel, PE/EA/TEA, 10:9:0.1).

^{1}H NMR (400 MHz, $CDCl_3$): δ 8.63 (d, *J* = 5.2 Hz, 1 H), 8.63 (d, *J* = 5.2 Hz, 1 H, minor), 7.96 (s, 1 H), 7.92 (s, 1 H, minor), 7.29 (d, *J* = 4.8 Hz, 1 H), 7.24 (d, *J* = 4.8 Hz, 1 H, minor), 4.00 (s, 3 H), 4.00 (s, 3 H, minor), 2.77 (dd, *J* = 13.6, 6.4 Hz, 1 H), 2.55–2.50 (m, 1 H, minor), 2.40 (t, *J* = 7.2 Hz, 2 H), 2.28–2.18 (m, 1 H), 2.09 (s, 3 H), 2.04 (s, 3 H, minor), 1.74–1.69 (m, 1 H), 1.63–1.51 (m, 4 H), 1.44–1.35 (m, 1 H), 1.26 (d, *J* = 6.8 Hz, 3 H), 0.76 (t, *J* = 7.2 Hz, 3 H, minor) ppm.

^{13}C NMR (100 MHz, $CDCl_3$): δ 208.4, 207.9 (minor), 166.0, 165.2 (minor), 157.6, 155.6 (minor), 149.9 (minor), 149.8, 148.1 (minor), 148.0, 126.5 (minor), 125.8, 124.5 (minor), 123.9, 52.9, 52.9 (minor), 46.6 (minor), 43.3, 41.2 (minor), 39.5, 36.8, 30.0 (minor), 29.9, 29.1 (minor), 29.0 (minor), 21.5, 21.3, 11.9 (minor) ppm.

HRMS (ESI) *m/z* calcd for $C_{14}H_{19}NO_3H^+$ [M + H]$^+$ 250.1438, found: 250.1435.

IR (KBr, cm^{-1}): ν_{max} 2956, 2920, 1747, 1723, 1604, 1439, 1297, 1209, 1126, 1097, 984, 791.

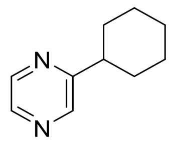

2-Cyclohexylpyrazine (**128**)**:** *The General Procedure D* was applied with pyrazine (39.6 mg, 0.5 mmol, 1 equiv.), H_2SO_4 (67.5 μL, 2.4 equiv.), $FeCl_2$ (3.2 mg, 0.025 mmol, 0.05 equiv.), BCMOM (37.2 mg, 0.05 mmol, 0.1 equiv.), cyclohexane (108.6 μL, 1.0 mmol, 2 equiv.), H_2O_2 (35%) (129 μL, 1.5 mmol, 3 equiv.), acetonitrile (4 mL) and water (4 mL) at 80 ℃ under N_2 for 3 h. Column chromatography (PE/EA/TEA, from 10:1:0.1 to 10:3:0.1) afforded the title product as a yellow liquid (60.9 mg, 58%), known compound (CAS: 53190-45-7).

TLC: R_f = 0.55 (silica gel, PE/EA/TEA, 1:3:0.1).

^{1}H NMR (400 MHz, $CDCl_3$): δ 8.49 (d, *J* = 2.4 Hz, 1 H), 8.47 (s, 1 H), 8.38 (d, *J* = 2.4 Hz, 1 H), 2.78-2.70 (m, 1 H), 1.97–1.84 (m, 4 H), 1.81–1.73 (m, 1 H), 1.62–1.52 (m, 3 H), 1.48–1.35 (m, 2 H) ppm.

^{13}C NMR (100 MHz, $CDCl_3$): δ 161.7, 143.9, 143.5, 142.2, 44.0, 32.4, 26.3, 25.8 ppm.

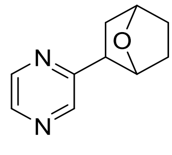

2-(7-Oxabicyclo[2.2.1]heptan-2-yl)pyrazine (**129**)**:** *The General Procedure D* was applied with pyrazine (39.6 mg, 0.5 mmol, 1 equiv.), H_2SO_4 (67.5 μL, 2.4 equiv.), $FeCl_2$ (3.2 mg, 0.025 mmol, 0.05 equiv.), BCMOM (37.2 mg, 0.05 mmol, 0.1 equiv.), 1,4-oxycyclohexane (103.5 μL, 1.0 mmol, 2 equiv.), H_2O_2 (35%) (129 μL, 1.5 mmol, 3 equiv.), acetonitrile (4 mL) and water (4 mL) at 80 ℃ under N_2 for 3 h. Column

chromatography (PE/EA/TEA, from 10:6:0.1 to 10:9:0.1) afforded the title product as a yellow liquid (51.1 mg, 58%), unknown compound.

TLC: R_f = 0.53 (silica gel, PE/EA/TEA, 10:9:0.1).

^{1}H NMR (400 MHz, $CDCl_3$): δ 8.61 (s, 1 H), 8.46 (s, 1 H), 8.39 (d, J = 2.4 Hz, 1 H), 4.79 (t, J = 4.0 Hz, 1 H), 4.64 (d, J = 4.8 Hz, 1 H), 3.17 (dd, J = 8.0, 6.0 Hz, 1 H), 2.06 (d, J = 7.2 Hz, 2 H), 1.83-1.81 (m, 2 H), 1.71–1.62 (m, 1 H), 1.61–1.55 (m, 1 H) ppm.

^{13}C NMR (100 MHz, $CDCl_3$): δ 159.9, 144.0, 143.5, 142.3, 81.1, 76.6, 49.0, 39.0, 30.2, 29.6 ppm.

HRMS (ESI) *m/z* calcd for $C_{10}H_{12}N_2OH^+$ $[M + H]^+$ 177.1023, found: 177.1019.

IR (KBr, cm^{-1}): ν_{max} 2986, 2956, 2873, 1687, 1533, 1463, 1404, 1304, 1203, 1138, 1044, 1021, 997, 926, 896, 778, 554, 412.

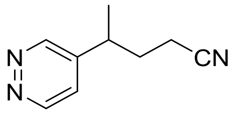

4-(Pyridazin-4-yl)pentanenitrile (**130**)**:** *The General Procedure D* was applied with pyridazine (36.6 μL, 0.5 mmol, 1 equiv.), H_2SO_4 (67.5 μL, 2.4 equiv.), $FeCl_2$ (3.2 mg, 0.025 mmol, 0.05 equiv.), BCMOM (37.2 mg, 0.05 mmol, 0.1 equiv.), hexanenitrile (106.8 μL, 1.0 mmol, 2 equiv.), H_2O_2 (35%) (129 μL, 1.5 mmol, 3 equiv.), acetonitrile (4 mL) and water (4 mL) at 80 ℃ under N_2 for 3 h. The reaction mixture is carefully acidified with hydrochloric acid 10% (pH 3-4). Column chromatography (PE/EA/FA, from 1:2:0.1 to 1:3:0.1) afforded the title product as a yellow liquid (54.8 mg, 68%), unknown compound.

TLC: R_f = 0.55 (silica gel, PE/EA/FA, 1:3:0.1).

^{1}H NMR (400 MHz, $CDCl_3$): 9.12 (d, J = 5.2, 1 H) 9.09 (s, 1 H), 7.31 (dd, J = 5.2, 2.4 Hz, 1 H), 2.99–2.85 (m, 1 H), 2.38–2.30 (m, 1 H), 2.25–2.17 (m, 1 H), 2.04–1.92 (m, 2 H), 1.35 (d, J = 6.8 Hz, 3 H) ppm.

^{13}C NMR (100 MHz, $CDCl_3$): δ 151.4, 151.2, 143.9, 124.5, 118.5, 36.0, 32.1, 20.3, 15.3 ppm.

HRMS (ESI) *m/z* calcd for $C_9H_{11}N_3H^+$ $[M + H]^+$ 162.1026, found: 162.1024.

IR (KBr, cm^{-1}): ν_{max} 3381, 2980, 2939, 2236, 1700, 1658, 1581, 1463, 1392, 1102, 1055, 984, 866, 660.

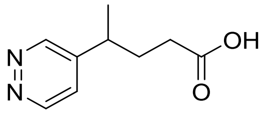

4-(Pyridazin-4-yl)pentanoic acid (131): *The General Procedure D* was applied with pyridazine (36.6 μL, 0.5 mmol, 1 equiv.), H_2SO_4 (67.5 μL, 2.4 equiv.), $FeCl_2$ (3.2 mg, 0.025 mmol, 0.05 equiv.), BCMOM (37.2 mg, 0.05 mmol, 0.1 equiv.), pentanoic acid (109.9 μL, 1.0 mmol, 2 equiv.), H_2O_2 (35%) (129 μL, 1.5 mmol, 3 equiv.), acetonitrile (4 mL) and water (4 mL) at 80 ℃ under N_2 for 3 h. The reaction mixture is carefully acidified with hydrochloric acid 10% (pH 3-4). Column chromatography (PE/EA/FA, from 1:2:0.1 to 1:3:0.1) afforded the title product as a brown liquid (61.2 mg, 68%), unknown compound.

TLC: R_f = 0.50 (silica gel, PE/EA/FA, 1:3:0.1).

^{1}H NMR (400 MHz, $CDCl_3$): δ 10.65 (s, 1 H), 9.09 (s, 2 H), 7.44 (s, 1 H), 2.88-2.84 (m, 1 H), 2.29 (t, J = 6.8 Hz, 2 H), 2.03–1.88 (m, 2 H), 1.31 (d, J = 6.4 Hz, 3 H) ppm.

^{13}C NMR (100 MHz, $CDCl_3$): δ 176.5, 151.5, 150.7, 147.1, 125.7, 36.4, 31.9, 31.8, 20.5 ppm.

HRMS (ESI) *m/z* calcd for $C_9H_{12}N_2O_2H^+$ $[M + H]^+$ 181.0972, found: 181.0965.

IR (KBr, cm^{-1}): ν_{max} 2974, 2920, 2532, 1711, 1593, 1398, 1274, 1198, 979, 855, 678.

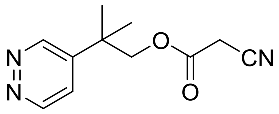

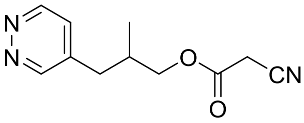

2-Methyl-2-(pyridazin-4-yl)propyl 2-cyanoacetate (132-1) and 2-methyl-3-(pyridazin-4-yl)propyl 2-cyanoacetate (132-2) (132-1:132-2 = 5:2): *The General Procedure D* was applied with pyridazine (36.6 μL, 0.5 mmol, 1 equiv.), H_2SO_4 (67.5 μL, 2.4 equiv.), $FeCl_2$ (3.2 mg, 0.025 mmol, 0.05 equiv.), BCMOM (37.2 mg, 0.05 mmol, 0.1 equiv.), butyl 2-cyanoacetate (144.0 μL, 2 equiv., 1.0 mmol), H_2O_2 (35%) (129 μL, 1.5 mmol, 3 equiv.), acetonitrile (4 mL) and water (4 mL) at 80 ℃ under N_2 for 3 h. The reaction mixture is carefully acidified with hydrochloric acid 10% (pH 3-4). Column chromatography (PE/EA/FA, from 1:2:0.1 to 1:3:0.1) afforded the title product as a yellow liquid (61.3 mg, 56%), unknown compound.

TLC: R_f = 0.48 (silica gel, PE/EA/FA, 1:3:0.1).

^{1}H NMR (400 MHz, $CDCl_3$): δ 9.09 (s, 1 H), 8.90 (t, *J* = 4.8 Hz, 2 H, minor), 8.87 (d, *J* = 5.2 Hz, 1 H), 7.44 (dd, *J* = 5.2, 2.4 Hz, 1 H), 7.32–7.28 (m, 1 H, minor), 4.63 (s, 2 H), 3.63 (s, 2 H), 3.50–3.45 (m, 1 H, minor), 3.39–3.35 (m, 1 H, minor), 2.86–2.82 (m, 1 H, minor), 2.38–2.33 (m, 1 H, minor), 2.00–1.90 (m, 1 H, minor), 1.28 (s, 6 H), 0.81 (d, *J* = 6.8 Hz, 3 H, minor).

^{13}C NMR (100 MHz, $CDCl_3$): δ 178.9, 153.2 (minor), 150.9, 150.7 (minor), 150.5, 147.5, 141.0 (minor), 127.1(minor), 124.8, 71.1, 66.3 (minor), 45.8 (minor), 39.6, 39.2, 36.6 (minor), 36.2 (minor), 24.1, 16.1 (minor) ppm.

HRMS (ESI) *m/z* calcd for $C_{11}H_{13}N_3O_2H^+$ $[M - H]^+$ 218.0935, found: 218.0930.

IR (KBr, cm^{-1}): ν_{max} 3346, 2974, 2933, 2868, 1587, 1469, 1363, 1297, 1050, 991, 855, 678, 589.

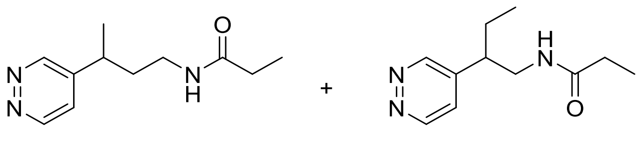

N-(3-(pyridazin-4-yl)butyl)propionamide (133-1) and N-(2-(pyridazin-4-yl)butyl)propionamide (133-2) (133-1:133-2 = 10:9): *The General Procedure D* was applied with pyridazine (36.6 μL, 0.5 mmol, 1 equiv.),

H_2SO_4 (67.5 μL, 2.4 equiv.), $FeCl_2$ (3.2 mg, 0.025 mmol, 0.05 equiv.), BCMOM (37.2 mg, 0.05 mmol, 0.1 equiv.), N-butylpropionamide (146.6 μL, 1.0 mmol, 2 equiv.), H_2O_2 (35%) (129 μL, 1.5 mmol, 3 equiv.), acetonitrile (4 mL) and water (4 mL) at 80 ℃ under N_2 for 3 h. Column chromatography (EA/TEA, 10:0.1) afforded the title product as a brown liquid (51.1 mg, 58%), unknown compound.

TLC: R_f = 0.53 (silica gel, EA/TEA, 10:0.1).

^{1}H NMR (400 MHz, $CDCl_3$): δ 9.04 (d, J = 5.2 Hz, 1 H), 9.01 (s, 1 H), 9.00 (s, 1 H), 8.95 (s, 1 H), 7.33 (dd, J = 5.2, 2.4 Hz, 1 H), 7.29 (dd, J = 5.2, 2.0 Hz, 1 H), 6.25 (s, 1 H), 5.94 (s, 1 H), 3.70–3.65 (m, 1 H), 3.35–3.25 (m, 1 H), 3.25–3.14 (m, 2 H), 2.85–2.75 (m, 2 H), 2.21–2.08 (m, 4 H), 1.84 (dd, J = 14.8, 7.6 Hz, 2 H), 1.67–1.52 (m, 2 H), 1.29 (d, J = 6.8 Hz, 3 H), 1.10 ppm (t, J = 7.6 Hz, 3 H), 1.05 (t, J = 7.4 Hz, 3 H, minor), 0.81 (t, J = 7.2 Hz, 3 H, minor) ppm.

^{13}C NMR (100 MHz, $CDCl_3$):δ 174.3, 174.0, 152.4, 151.7, 151.1, 151.0, 145.9, 142.8, 125.8, 124.6, 45.0, 43.7, 37.4, 36.7, 34.9, 29.5, 29.4, 25.7, 21.0, 11.6, 9.9, 9.8 ppm.

HRMS (ESI) m/z calcd for $C_{11}H_{17}N_3OH^+$ $[M + H]^+$ 208.1445, found: 208.1437.

IR (KBr, cm^{-1}): ν_{max} 3275, 2980, 2933, 1652, 1546, 1457, 1374, 1233, 1050.

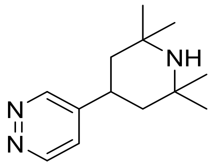

4-(2,2,6,6-Tetramethylpiperidin-4-yl)pyridazine (134): *The General Procedure C* was applied with pyridazine (36.6 μL, 0.5 mmol, 1 equiv.), 2,2,6,6-tetramethylpiperidine (174.0 μL, 1.0 mmol, 2 equiv.), H_2SO_4 (135.0 μL, 4.8 equiv.), $FeCl_2$ (3.2 mg, 0.025 mmol, 0.05 equiv.), BCMOM (37.2 mg, 0.05 mmol, 0.1 equiv.), H_2O_2 (35%) (129 μL, 1.5 mmol, 3 equiv.), acetonitrile (4 mL) and water (4 mL) at 80 ℃ under N_2 for 3 h. Column chromatography (PE/EA/TEA, from 1:2:0.2 to 1:3:0.2) afforded the title product as a yellow liquid (60.2 mg, 55%), unknown compound.

TLC: R_f = 0.45 (silica gel, PE/EA/TEA, 1:3:0.2).

^{1}H NMR (400 MHz, $CDCl_3$): δ 9.10 (s, 1 H), 9.09 (d, *J* = 5.2 Hz, 1 H), 7.30 (dd, *J* = 5.2, 2.4 Hz, 1 H), 3.10–3.00 (m, 1 H), 1.78 (dd, *J* = 12.8, 3.2 Hz, 2 H), 1.35 (d, *J* = 7.2 Hz, 1 H), 1.31 (s, 6 H), 1.26 (d, *J* = 7.2 Hz, 1 H), 1.20 (s, 6 H) ppm.

^{13}C NMR (100 MHz, $CDCl_3$): δ 152.0, 151.2, 145.4, 124.3, 50.7, 44.6, 34.8, 33.3, 28.1 ppm.

HRMS (ESI) *m/z* calcd for $C_{13}H_{21}N_3H^+$ $[M + H]^+$ 220.1808, found: 220.1801.

IR (KBr, cm^{-1}): ν_{max} 3364, 3038, 2963, 2920, 2732, 1652, 1593, 1368, 1239, 1097, 979, 791, 678.

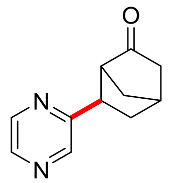

6-(Pyrazin-2-yl)bicyclo[2.2.1]heptan-2-one (135): *The General Procedure D* was applied with pyrazine (19.8 mg, 0.25 mmol, 1 equiv.), H_2SO_4 (33.0 μL, 2.4 equiv.), $FeCl_2$ (1.6 mg, 0.0125 mmol, 0.05 equiv.), BCMOM (18.6 mg, 0.025 mmol, 0.1 equiv.), 2,5-methanocyclohexanone (55.5 mg, 0.5 mmol, 2 equiv.), H_2O_2 (35%) (64.5 μL, 0.75 mmol, 3 equiv.), acetonitrile (2 mL) and water (2 mL) at 80 ℃ under N_2 for 3 h. Column chromatography (PE/EA/TEA, from 10:5:0.1 to 10:8:0.1) afforded the title product as a yellow liquid (13.2 mg, 28%), unknown compound.

TLC: R_f = 0.58 (silica gel, PE/EA/TEA, 10:8:0.1).

^{1}H NMR (400 MHz, $CDCl_3$): δ 8.50 (s, 1 H), 8.49 (s, 1 H), 8.41 (s, 1 H), 3.24 (dd, *J* = 8.4, 5.2 Hz, 1 H), 2.86 (s, 1 H), 2.77 (s, 1 H), 2.40–2.31 (m, 1 H), 2.23–2.12 (m, 2 H), 2.05–1.92 (m, 2 H), 1.71 (d, *J* = 10.0 Hz, 1 H) ppm.

^{13}C NMR (100 MHz, $CDCl_3$): δ 216.7, 158.2, 144.6, 143.8, 142.6, 56.3, 44.8, 40.5, 35.8, 34.9, 34.5 ppm.

HRMS (ESI) *m/z* calcd for $C_{11}H_{12}N_2OH^+$ $[M + H]^+$ 189.1023, found: 189.1018.

IR (KBr, cm^{-1}): ν_{max} 2956, 2915, 1747, 1404, 1150, 1020, 843, 412.

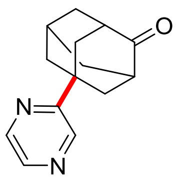

5-(Pyrazin-2-yl)adamantan-2-one (136): *The General Procedure D* was applied with pyrazine (19.8 mg, 0.25 mmol, 1 equiv.), H_2SO_4 (33.0 μL, 2.4 equiv.), $FeCl_2$ (1.6 mg, 0.0125 mmol, 0.05 equiv.), BCMOM (18.6 mg, 0.025 mmol, 0.1 equiv.), 2-adamantanone (77.5 mg, 0.5 mmol, 2 equiv.), H_2O_2 (35%) (64.5 μL, 0.75 mmol, 3 equiv.), acetonitrile (2 mL) and water (2 mL) at 80 ℃ under N_2 for 3 h. Column chromatography (PE/EA/TEA, from 10:3:0.1 to 10:5:0.1) afforded the title product as a yellow solid (17.1 mg, 30%), unknown compound.

TLC: R_f = 0.43 (silica gel, PE/EA/TEA, 10:5:0.1).

^{1}H NMR (400 MHz, $CDCl_3$): δ 8.60 (d, J = 1.2 Hz, 1 H), 8.53 (t, J = 2.4 Hz, 1 H), 8.44 (d, J = 2.4 Hz, 1 H), 2.70 (s, 2 H), 2.34–2.31 (m, 4 H), 2.25 (s, 2 H), 2.17–2.01 (m, 5 H) ppm.

^{13}C NMR (100 MHz, $CDCl_3$): δ 217.1, 161.3, 143.7, 142.5, 141.2, 46.2, 42.6, 40.5, 38.4, 38.0, 27.8 ppm.

HRMS (ESI) *m/z* calcd for $C_{14}H_{16}N_2OH^+$ $[M + H]^+$ 229.1336, found: 229.1334.

Mp: 73.3-74.3 ℃.

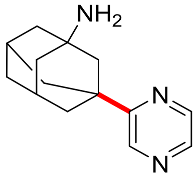

3-(Pyrazin-2-yl)adamantan-1-amine (137): *The General Procedure C* was applied with pyrazine (19.8 mg, 0.25 mmol, 1 equiv.), adamantan-2-amine (94.8 mg, 0.5 mmol, 2 equiv.), H_2SO_4 (67.5 μL, 4.8 equiv.), $FeCl_2$ (1.6 mg, 0.0125 mmol, 0.05 equiv.), BCMOM (18.6 mg, 0.025 mmol, 0.1 equiv.), H_2O_2 (35%) (64.5 μL, 0.75 mmol, 3 equiv.), acetonitrile (2 mL) and water (2 mL) at 80 ℃ under N_2 for 3 h.

Column chromatography (EA/TEA, from 1:0.1 to 1:0.2) afforded the title product as yellow liquid (31.6 mg, 55%), unknown compound.

TLC: R_f = 0.23 (silica gel, EA/TEA, 1:0.2).

^{1}H NMR (400 MHz, $CDCl_3$): δ 8.56 (s, 1 H), 8.49 (s, 1 H), 8.39 (s, 1 H), 2.30 (s, 2 H), 2.00–1.84 (m, 8 H), 1.73 (s, 4 H), 1.68 (s, 2 H) ppm.

^{13}C NMR (100 MHz, $CDCl_3$): 162.5, 143.6, 142.2, 141.5, 49.1, 48.4, 43.7, 40.5, 40.2, 35.0, 29.7 ppm.

HRMS (ESI) *m/z* calcd for $C_{14}H_{19}N_3H^+$ $[M + H]^+$ 230.1652, found: 230.1645.

IR (KBr, cm^{-1}): ν_{max} 2915, 2856, 1575, 1398, 1132, 1097, 1055, 1020.

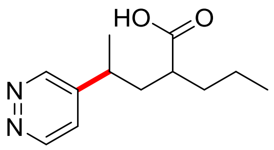

2-Propyl-4-(pyridazin-4-yl)pentanoic acid (138): *The General Procedure D* was applied with pyridazine (18.3 μL, 0.25 mmol, 1 equiv.), H_2SO_4 (33.0 μL, 2.4 equiv.), $FeCl_2$ (1.6 mg, 0.0125 mmol, 0.05 equiv.), BCMOM (18.6 mg, 0.025 mmol, 0.1 equiv.), 2-propylpentanoic acid (80.9 μL, 0.5 mmol, 2 equiv.), H_2O_2 (35%) (64.5 μL, 0.75 mmol, 3 equiv.), acetonitrile (2 mL) and water (2 mL) at 80 ℃ under N_2 for 3 h. The reaction mixture is carefully acidified with hydrochloric acid 10% (pH 3-4). Column chromatography (EA/TEA, 1:0.1) afforded the title product as a yellow liquid (28.9 mg, 52%), unknown compound.

TLC: R_f = 0.58 (silica gel, EA/TEA, 1:0.1).

^{1}H NMR (400 MHz, $CDCl_3$) δ 10.97 (s, 1 H), 9.10 (d, *J* = 5.6 Hz, 1 H), 9.06 (d, *J* = 5.2 Hz, 1 H), 7.42 (d, *J* = 3.2 Hz, 1 H), 2.89–2.83 (m, 1 H), 2.44–1.98 (m, 2 H), 1.82–1.58 (m, 2 H), 1.50–1.34 (m, 2 H), 1.32-1.30 (m, 3 H), 1.27–1.22 (m, 1 H), 0.91-0.82 (m, 3 H) ppm.

^{13}C NMR (100 MHz, $CDCl_3$) δ 179.4, 151.7, 150.3, 147.3, 125.9, 44.2, 39.5, 36.2, 35.5, 35.3, 21.1, 20.3 13.9 ppm.

HRMS (ESI) *m/z* calcd for $C_{12}H_{18}N_2O_2H^+$ $[M + H]^+$ 223.1441, found: 223.1436.

IR (KBr, cm^{-1}): ν_{max} 2956, 2933, 2868, 1717, 1581, 1463, 1387, 1239, 1186, 1091, 979, 866.

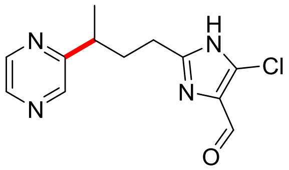

5-Chloro-2-(3-(pyrazin-2-yl)butyl)-1λ^2-imidazole-4-carbaldehyde (139): *The General Procedure D* was applied with pyrazine (19.8 mg, 0.25 mmol, 1 equiv.), H_2SO_4 (33.0 μL, 2.4 equiv.), $FeCl_2$ (1.6 mg, 0.0125 mmol, 0.05 equiv.), BCMOM (18.6 mg, 0.025 mmol, 0.1 equiv.), 2-butyl-5-chloro-1λ^2-imidazole-4-carbaldehyde (95.2 mg, 0.5 mmol, 2 equiv.), H_2O_2 (35%) (64.5 μL, 0.75 mmol, 3 equiv.), acetonitrile (2 mL) and water (2 mL) at 80 ℃ under N_2 for 3 h. Column chromatography (PE/EA/TEA, from 10:5:0.1 to 10:8:0.1) afforded the title product as a yellow liquid (34.2 mg, 52%), unknown compound.

TLC: R_f = 0.58 (silica gel, PE/EA/TEA, 10:8:0.1).

^{1}H NMR (400 MHz, $CDCl_3$): δ 9.59 (s, 1 H), 8.51 (dd, *J* = 4.8, 1.2 Hz, 2 H), 8.42 (d, *J* = 2.4 Hz, 1 H), 3.06–2.93 (m, 1 H), 2.78–2.63 (m, 2 H), 2.26–2.07 (m, 2 H), 1.36 (d, *J* = 7.2 Hz, 3 H) ppm.

^{13}C NMR (100 MHz, $CDCl_3$) δ 177.6, 160.2, 153.4, 143.9, 143.9, 142.6, 141.2, 126.0, 38.5, 34.3, 26.7, 20.3 ppm.

HRMS (ESI) *m/z* calcd for $C_{12}H_{13}ClN_4OH^+$ $[M + H]^+$ 265.0851, found: 265.0843.

IR (KBr, cm^{-1}): ν_{max} 2968, 2933, 1676, 1516, 1387, 1250, 1102, 1020, 808, 412.

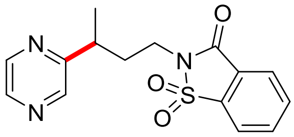

2-(3-(Pyrazin-2-yl)butyl)benzo[d]isothiazol-3(2H)-one 1,1-dioxide (140): *The General Procedure D* was applied with pyrazine (19.8 mg, 1 equiv., 0.25 mmol), H_2SO_4 (33.0 μL, 2.4 equiv.), $FeCl_2$ (1.6 mg, 0.0125 mmol, 0.05 equiv.), BCMOM (18.6 mg, 0.025 mmol, 0.1 equiv.), 2-butylbenzo[d]isothiazol-3(2H)-one 1,1-dioxide (120.9 mg, 0.5 mmol, 2 equiv.), H_2O_2 (35%) (64.5 μL, 0.75 mmol, 3 equiv.), acetonitrile (2 mL) and water (2 mL) at 80 ℃ under N_2 for 3 h. Column chromatography (PE/EA/TEA, from 10:5:0.1 to 10:8:0.1) afforded the title product as a yellow liquid (47.6 mg, 60%), unknown compound.

TLC: R_f = 0.58 (silica gel, PE/EA/TEA, 10:8:0.1).

^{1}H NMR (400 MHz, $CDCl_3$): δ 8.50 (d, J = 10.0 Hz, 2 H), 8.41 (s, 1 H), 8.02 (d, J = 7.2 Hz, 1 H), 7.90 (d, J = 7.2 Hz, 1 H), 7.87–7.77 (m, 2 H), 3.73 (t, J = 7.2 Hz, 2 H), 3.16–3.05 (m, 1 H), 2.45–2.33 (m, 1 H), 2.21–2.11 (m, 1 H), 1.36 (d, J = 6.8 Hz, 3 H) ppm.

^{13}C NMR (100 MHz, $CDCl_3$) δ 160.0, 158.9, 144.2, 142.6, 137.6, 134.7, 134.3, 127.2, 125.1, 120.9, 37.5, 36.6, 34.2, 20.6 ppm.

HRMS (ESI) *m/z* calcd for $C_{15}H_{15}N_3O_3SH^+$ $[M + H]^+$ 318.0907, found: 318.0906.

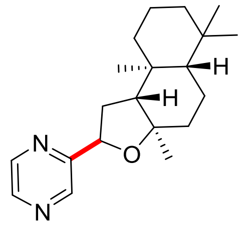

2-((3aR,5aS,9aS,9bR)-3a,6,6,9a-Tetramethyldodecahydronaphtho[2,1-b]furan-2-yl)pyrazine (141): *The General Procedure D* was applied with pyrazine (19.8 mg, 0.25 mmol, 1 equiv.), H_2SO_4 (33.0 μL, 2.4 equiv.), $FeCl_2$ (1.6 mg, 0.0125 mmol, 0.05 equiv.), BCMOM (18.6 mg, 0.025 mmol, 0.1 equiv.), (3aR,5aS,9aS,9bR)-3a,6,6,9a-tetramethyldodecahydronaphtho[2,1-b]furan (120.6 mg, 2 equiv., 0.5 mmol), H_2O_2 (35%) (64.5 μL, 0.75 mmol, 3 equiv.), acetonitrile (2 mL) and water (2 mL) at 80 ℃ under N_2 for 3 h. Column chromatography

(PE/EA/DCM/TEA, 10:1:0.5:0.1) afforded the title product as a yellow liquid (33.0 mg, 42%), unknown compound.

TLC: R_f = 0.38 (silica gel, PE/EA/DCM/TEA, 10:1:0.5:0.1).

^{1}H NMR (400 MHz, $CDCl_3$) δ 8.77 (s, 1 H), 8.51–8.48 (m, 1 H), 8.45 (d, *J* = 2.4 Hz, 1 H), 5.22 (dd, *J* = 9.6, 2.4 Hz, 1 H), 2.39–2.32 (m, 1 H), 2.11–2.06 (m, 1 H), 1.87–1.81 (m, 2 H), 1.64 (dd, *J* = 13.2, 3.6 Hz, 2 H), 1.54 (dd, *J* = 13.6, 6.8 Hz, 2 H), 1.40–1.29 (m, 4 H), 1.17–1.07 (m, 3 H), 1.02–0.94 (m, 2 H), 0.88 (s, 6 H), 0.83 (s, 3 H) ppm.

^{13}C NMR (100 MHz, $CDCl_3$) δ 159.4, 143.6, 142.8, 142.4, 82.8, 76.8, 58.4, 57.2, 42.3, 39.9, 39.8, 36.2, 33.5, 33.1, 30.4, 21.8, 21.1, 20.7, 18.3, 15.1 ppm.

HRMS (ESI) *m/z* calcd for $C_{20}H_{30}N_2OH^+$ $[M + H]^+$ 315.2431, found: 315.2421.

IR (KBr, cm^{-1}): ν_{max} 2927, 2862, 1717, 1457, 1380, 1310, 1156, 1126, 1027, 855, 407.

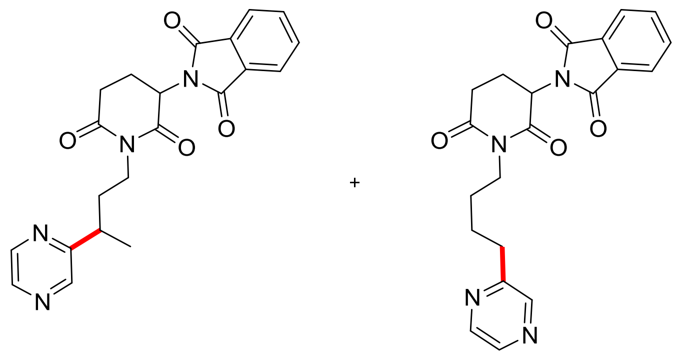

2-(2,6-Dioxo-1-(3-(pyrazin-2-yl)butyl)piperidin-3-yl)isoindoline-1,3-dione (142-1) and **2-(2,6-dioxo-1-(4-(pyrazin-2-yl)butyl)piperidin-3-yl)isoindoline-1,3-dione (142-2) (142-1:142-2 = 2:1):** *The General Procedure D* was applied with pyrazine (19.8 mg, 0.25 mmol, 1 equiv.), H_2SO_4 (33.0 μL, 2.4 equiv.), $FeCl_2$ (1.6 mg, 0.0125 mmol, 0.05 equiv.), BCMOM (18.6 mg, 0.025 mmol, 0.1 equiv.), 2-(1-butyl-2,6-dioxopiperidin-3-yl)isoindoline-1,3-dione (160.3 mg, 0.5 mmol, 2 equiv.), H_2O_2 (35%) (64.5 μL, 0.75 mmol, 3 equiv.), acetonitrile (2 mL) and water (2

mL) at 80 ℃ under N_2 for 3 h. Column chromatography (PE/EA/TEA, 10:4:0.1) afforded the title product as a yellow liquid (58.8 mg, 60%), unknown compound.

TLC: R_f = 0.38 (silica gel, PE/EA /TEA, 10:4:0.1).

^{1}H NMR (400 MHz, $CDCl_3$) δ 8.49 (s, 1 H), 8.46 (dd, *J* = 8.0, 1.2 Hz, 1 H), 8.38 (dd, *J* = 4.4, 2.4 Hz, 1 H), 7.90–7.84 (m, 3 H), 7.78–7.71 (m, 3 H), 5.04–4.97 (m, 1 H, minor), 4.96–4.86 (m, 1 H), 4.02–3.89 (m, 1 H, minor), 3.88–3.81 (m, 1 H, minor),3.80–3.64 (m, 2 H), 3.22–3.13 (m, 1 H), 3.01–2.88 (m, 2 H), 2.86–2.77 (m, 2 H, minor), 2.76–2.62 (m, 2 H), 2.18–2.06 (m, 2 H), 1.90–1.82 (m, 1 H, minor), 1.81–1.65 (m, 1 H, minor), 1.65–1.51 (m, 1 H, minor), 1.46 (t, *J* = 7.2 Hz, 4 H, minor), 1.31 (dd, *J* = 6.8, 2.8 Hz, 3 H) ppm.

^{13}C NMR (100 MHz, $CDCl_3$) δ 170.9 (minor), 170.7, 168.3, 168.0 (minor), 167.3, 167.2 (minor), 160.7, 160.6 (minor), 144.1 (minor), 144.0, 144.0 (minor), 143.9, 142.4 (minor), 142.4, 134.4, 134.4 (minor), 131.6, 131.6 (minor), 123.7, 123.7 (minor), 58.3 (minor), 50.0, 49.3 (minor), 46.3, 39.0 (minor), 37.1, 33.7 (minor), 31.9, 31.4 (minor), 22.6 (minor), 21.9, 20.7, 18.4 (minor), 8.6 ppm.

HRMS (ESI) *m/z* calcd for $C_{21}H_{20}N_4O_4H^+$ $[M + H]^+$ 393.1558, found: 393.1550.

IR (KBr, cm^{-1}): ν_{max} 2963, 2915, 1723, 1676, 1387, 1351, 1179, 1126, 1020, 725.

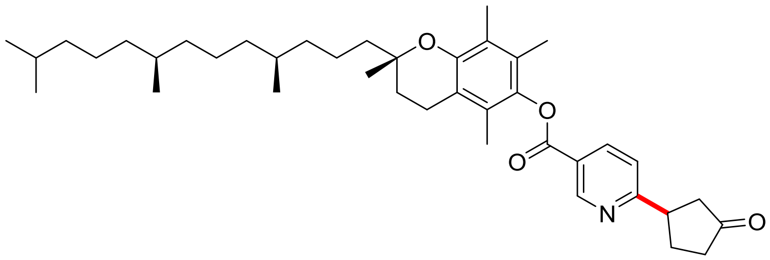

(S)-2,5,7,8-Tetramethyl-2-((4S,8S)-4,8,12-trimethyltridecyl)chroman-6-yl 6-(3-oxocyclopentyl)nicotinate (143): *The General Procedure D* was applied with (S)-2,5,7,8-tetramethyl-2-((4S,8S)-4,8,12-trimethyltridecyl)chroman-6-yl nicotinate (141.0 mg, 0.25 mmol, 1 equiv.), H_2SO_4 (33.0 μL, 2.4 equiv.), $FeCl_2$ (1.6 mg, 0.0125 mmol, 0.05 equiv.), BCMOM (18.6 mg, 0.025 mmol, 0.1 equiv.), cyclopentanone

(44.5 μL, 0.5 mmol, 2 equiv.), H_2O_2 (35%) (64.5 μL, 0.75 mmol, 3 equiv.), acetonitrile (2 mL) and water (2 mL) at 80 ℃ under N_2 for 3 h. Column chromatography (PE/EA/TEA, from 10:1:0.1 to 10:3:0.1) afforded the title product as a brown liquid (80.3 mg, 52%), unknown compound.

TLC: R_f = 0.46 (silica gel, PE/EA/TEA, 10:3:0.1).

^{1}H NMR (400 MHz, $CDCl_3$): δ 9.38 (d, *J* = 2.0 Hz, 1 H), 8.43 (dd, *J* = 8.0, 2.0 Hz, 1 H), 7.37 (d, *J* = 8.0 Hz, 1 H), 3.73–3.63 (m, 1 H), 2.76 (dd, *J* = 18.4, 9.6 Hz, 1 H), 2.67 (d, *J* = 8.0 Hz, 1 H), 2.62 (t, *J* = 6.8 Hz, 2 H), 2.58–2.43 (m, 2 H), 2.38–2.29 (m, 1 H), 2.27–2.19 (m, 1 H), 2.12 (s, 3 H), 2.05 (s, 3 H), 2.01 (s, 3 H), 1.88–1.77 (m, 2 H), 1.74–1.27 (m, 16 H), 1.19–0.98 (m, 8 H), 0.85 (t, *J* = 6.8 Hz, 12 H) ppm.

^{13}C NMR (100 MHz, $CDCl_3$): δ 217.9, 167.3, 163.8, 151.2, 149.6, 140.2, 138.3, 126.7, 125.0, 123.8, 123.3, 121.9, 117.6, 75.1, 44.3, 44.2, 39.3, 38.2, 37.53–37.23 (m), 32.81–32.62 (m), 30.1, 28.0, 24.8, 24.4, 22.7, 22.6, 21.0, 20.6, 19.78–19.53 (m), 13.1, 12.2, 11.9 ppm.

HRMS (ESI) *m/z* calcd for $C_{40}H_{59}NO_4H^+$ $[M + H]^+$ 618.4517, found: 618.4512.

IR (KBr, cm^{-1}): ν_{max} 2947, 2924, 2859, 1731, 1590, 1461, 1377, 1283, 1236, 1159, 1094, 1023, 728.

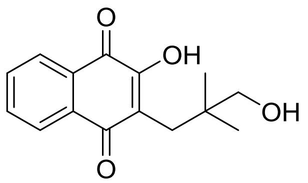

2-Hydroxy-3-(3-hydroxy-2,2-dimethylpropyl)naphthalene-1,4-dione (146): *The General Procedure* was applied with 2-hydroxynaphthalene-1,4-dione (87.4 mg, 0.5 mmol, 1 equiv.), 2,2-dimethylpropan-1-ol (136.3 mg, 1.5 mmol, 3 equiv.), $Fe(acac)_2$ (6.6 mg, 0.025 mmol, 0.05 equiv.), BCMOM (74.4 mg, 0.1 mmol, 0.2 equiv.), H_2O_2 (35%) (129 μL, 1.5 mmol, 3 equiv.), acetonitrile (4 mL) and water (4 mL) at 80 ℃ under N_2 for 3 h. Column chromatography (PE/DCM, from 1:2 to 1:5) afforded the title product as a yellow solid (61.1 mg, 47%), known compound (CAS:

171522-35-3).

TLC: R_f = 0.5 (silica gel, PE/DCM, 1:5).

^{1}H NMR (400 MHz, $CDCl_3$) δ 8.08 (d, *J* = 7.6 Hz, 1 H), 7.81 (d, *J* = 7.6 Hz, 1 H), 7.66 (t, *J* = 7.6 Hz, 1 H), 7.52 (t, *J* = 7.6 Hz, 1 H), 3.98 (s, 2 H), 2.34 (s, 2 H), 1.07 (s, 6 H) ppm.

^{13}C NMR (100 MHz, $CDCl_3$) δ 179.6, 179.0, 162.0, 134.9, 131.9, 130.8, 129.9, 128.8, 124.1, 113.3, 32.0, 29.7, 28.0, 24.8 ppm.

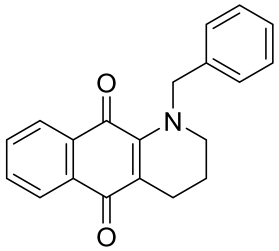

1-Benzyl-1,2,3,4-tetrahydrobenzo[*g*]quinoline-5,10-dione (149): *The General Procedure* was applied with naphthalene-1,4-dione (80.6 mg, 0.5 mmol, 1 equiv.), N-benzylpropan-1-amine (169.2 μL, 1.0 mmol, 2 equiv.), $FeCl_2$ (3.2 mg, 0.025 mmol, 0.05 equiv.), BCMOM (32.2 mg, 0.05 mmol, 0.1 equiv.), H_2O_2 (35%) (129 μL, 1.5 mmol, 3 equiv.), acetonitrile (4 mL) and water (4 mL) at 80 ℃ under N_2 for 3 h. Column chromatography (PE/EA, from 10:1 to 20:1) afforded the title product as a red liquid (106.1 mg, 70%), known compound (CAS: 135831-91-3).

TLC: R_f = 0.5 (silica gel, PE/EA, 10:1).

^{1}H NMR (400 MHz, $CDCl_3$) δ 8.04 (d, *J* = 7.6 Hz, 1 H), 7.91 (d, *J* = 7.6 Hz, 1 H), 7.65 (t, *J* = 7.6 Hz, 1 H), 7.56 (t, *J* = 7.6 Hz, 1 H), 7.37–7.33 (m, 2 H), 7.32–7.27 (m, 3 H), 4.86 (s, 2 H), 3.31–3.22 (m, 2 H), 2.68 (t, *J* = 6.4 Hz, 2 H), 1.92–1.83 (m, 2H) ppm.

^{13}C NMR (100 MHz, $CDCl_3$) δ 183.1, 181.5, 148.8, 137.9, 133.6, 132.7, 132.5, 131.8, 128.6, 127.3, 127.3, 126.1, 125.3, 117.8, 56.8, 50.1, 21.1, 20.3 ppm.

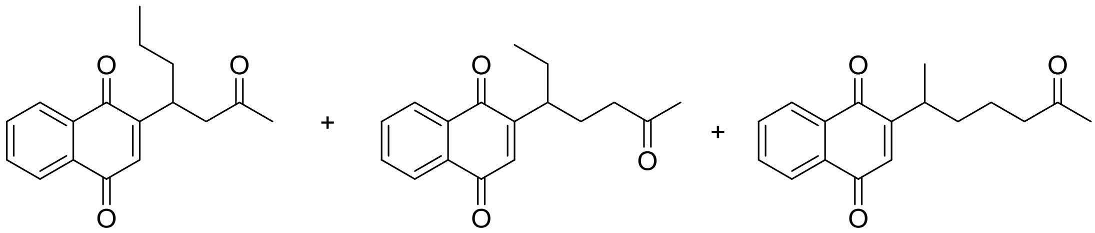

2-(2-oxoheptan-4-yl)naphthalene-1,4-dione (154-1) and 2-(6-oxoheptan-3-yl)naphthalene-1,4-dione (154-2) and 2-(6-oxoheptan-2-yl)naphthalene-1,4-dione (154-3) (154-1:154-2:154-3 = 1:5.8:6): *The General Procedure A* was applied with 1,4-naphthoquinone (80.6 mg, 0.5 mmol, 1 equiv.), 2-heptanone (140.7 μL, 1.0 mmol, 2 equiv.), Fe(acac)$_2$ (6.6 mg, 0.025 mmol, 0.05 equiv.), BCMOM (37.2 mg, 0.05 mmol, 0.1 equiv.), H_2O_2 (35%) (129 μL, 1.5 mmol, 3 equiv.), acetonitrile (4 mL) and water (4 mL) at 80 ℃ for 1 h. Column chromatography (PE/EA, 10:1) afforded the title product as a dark brown solid (94.6 mg,70%).

TLC: R_f = 0.30 (silica gel, PE/EA, 10:1).

154-1:

^{1}H NMR (400 MHz, $CDCl_3$): δ 8.17–8.09 (m, 1H), 8.08–8.00 (m, 1H), 7.83–7.62 (m, 2H), 6.72 (s, 1H), 3.49 (t, *J* = 7.3 Hz, 1H), 2.80 (d, *J* = 7.2 Hz, 2H), 2.13 (s, 5H), 1.57–1.52 (m, 2H), 1.34–1.26 (m, 2H), 0.89 (t, *J* = 7.3 Hz, 3H) ppm.

154-2 and **154-3**:

^{1}H NMR (400 MHz, $CDCl_3$): δ 8.14–8.03 (m, 2H), 7.77–7.72 (m, 2H), 6.74 (d, *J* = 7.6 Hz, 1H), 3.16-3.11 (m, 0.49H) (C2), 3.00-2.93 (m, 0.51H) (C1), 2.45 (t, *J* = 6.8 Hz, 1H), 2.39-2.35 (m, 1H), 2.13 (s, 1.5H), 2.09 (s, 1.5H), 2.04–1.91 (m, 0.5H), 1.82-1.75 (m, 0.5H), 1.69–1.53 (m, 3H), 1.19 (d, *J* = 6.9 Hz, 1.52H), 0.87 (t, *J* = 7.4 Hz, 1.48H) ppm.

^{13}C NMR (100 MHz, $CDCl_3$): δ 208.6, 208.0, 185.4, 185.2, 185.0, 184.8, 156.0, 154.2, 134.4, 133.8, 133.7, 133.5, 126.8, 126.7, 127.0, 126.0, 43.3, 41.3, 38.5, 35.3, 31.8, 30.0, 27.7, 27.3, 21.4, 19.4, 11.7 ppm.

HRMS (ESI): *calcd* for $C_{17}H_{19}O_3^+$ $[M + H]^+$ *m/z* 271.1329, *found:* 271.1329.

IR (KBr, cm^{-1}): ν_{max} 2960, 2932, 2876, 1693, 1595, 1456, 1287, 1265, 1205, 1073, 768, 713.

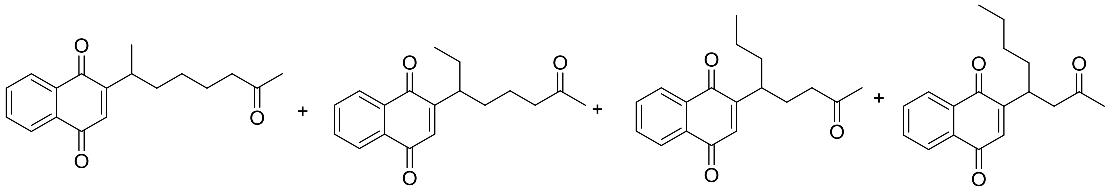

2-(7-oxooctan-2-yl)naphthalene-1,4-dione (155-1) and **2-(7-oxooctan-3-yl)naphthalene-1,4-dione (155-2)** and **2-(7-oxooctan-4-yl)naphthalene-1,4-dione (155-3)** and **2-(2-oxooctan-4-yl)naphthalene-1,4-dione (155-4)** **(155-1:155-2:155-3:155-4=4.3:3.5:1.6:0.6):** *The General Procedure A* was applied with 1,4-naphthoquinone (80.6 mg, 0.5 mmol, 1 equiv.), 2-octanone (156.6 μL, 1.0 mmol, 2 equiv.), $Fe(acac)_2$ (6.6 mg, 0.025 mmol, 0.05 equiv.), BCMOM (37.2 mg, 0.05 mmol, 0.1 equiv.), H_2O_2 (35%) (129 μL, 1.5 mmol, 3 equiv.), acetonitrile (4 mL) and water (4 mL) at 80 ℃ for 1 h. Column chromatography (PE/EA, 10:1) afforded the title product as a dark brown solid (59.7 mg, 42%).

TLC: R_f = 0.32 (silica gel, PE/EA, 10:1).

155-1 and **155-2** and **155-3** and **155-4**:

^{1}H NMR (400 MHz, $CDCl_3$): δ 8.20–7.99 (m, 2H), 7.76-7.72 (m, 2H), 6.78 (s, 0.16H) (C3), 6.74 (s, 0.78H) (C1+C2), 6.71 (s, 0.06H) (C4), 3.14-3.10 (m, 0.4H), 3.08–2.94 (m, 0.6H), 2.51–2.35 (m, 2.12H), 2.23–2.05 (m, 2.95H), 1.96 (dd, *J* = 15.6, 9.4 Hz, 0.67H), 1.86–1.70 (m, 0.6H), 1.58 (d, *J* = 6.9 Hz, 3.26H), 1.42–1.21 (m, 1.38H), 1.18 (d, *J* = 6.8 Hz, 1.2H) (C1), 0.86 (dd, *J* = 14.7, 9.8 Hz, 1.8H).

HRMS (ESI) *m/z* calcd. for $C_{18}H_{21}O_3^+$ [M + H^+] 285.1485, found: 285.1485.

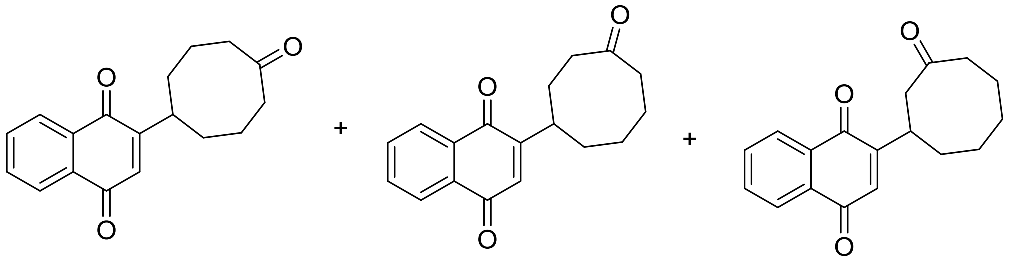

2-(5-oxocyclooctyl)naphthalene-1,4-dione (156-1) and 2-(4-oxocyclooctyl)naphthalene-1,4-dione (156-2) and 2-(3-oxocyclooctyl)naphthalene-1,4-dione (156-3) (156-1:156-2:156-3 = 6:2:1): *The General Procedure A* was applied with 1,4-naphthoquinone (80.6 mg, 0.5 mmol, 1 equiv.), cyclooctanone (132.9 μL, 1.0 mmol, 2 equiv.), Fe(acac)$_2$ (6.6 mg, 0.025 mmol, 0.05 equiv.), BCMOM (37.2 mg, 0.05 mmol, 0.1 equiv.), H_2O_2 (35%) (129 μL, 1.5 mmol, 3 equiv.), acetonitrile (4 mL) and water (4 mL) at 80 ℃ for 1 h. Column chromatography (PE/EA, 10:1) afforded the title product as a dark brown solid (63.5 mg, 45%).

TLC: R_f = 0.36 (silica gel, PE/EA, 10:1).

156-1:

^{1}H NMR (400 MHz, $CDCl_3$): δ 8.12–8.03 (m, 2H), 7.76–7.72 (m, 2H), 6.73 (s, 1H), 3.20–3.14 (m, 1H), 2.75–2.63 (m, 1H), 2.56–2.50 (m, 1H), 2.48–2.39 (m, 2H), 2.15–2.05 (m, 2H), 2.03–1.96 (m, 1H), 1.94–1.87 (m, 1H), 1.80–1.71 (m, 1H), 1.70–1.61 (m, 2H), 1.50–1.43 (m, 1H) ppm.

^{13}C NMR (100 MHz, $CDCl_3$): δ 217.0, 185.2, 184.7, 155.3, 133.8, 133.8, 133.4, 132.2, 131.8, 126.7, 126.0, 42.1, 40.2, 36.1, 30.2, 29.9, 25.9, 25.4 ppm.

HRMS (ESI) *m/z* calcd. for $C_{18}H_{19}O_3^+$ [M + H$^+$] 283.1329, found: 283.1329.

IR (KBr, cm^{-1}): ν_{max} 2925, 2855, 1700, 1662, 1592, 1468, 1329, 1306, 1257, 785, 708.

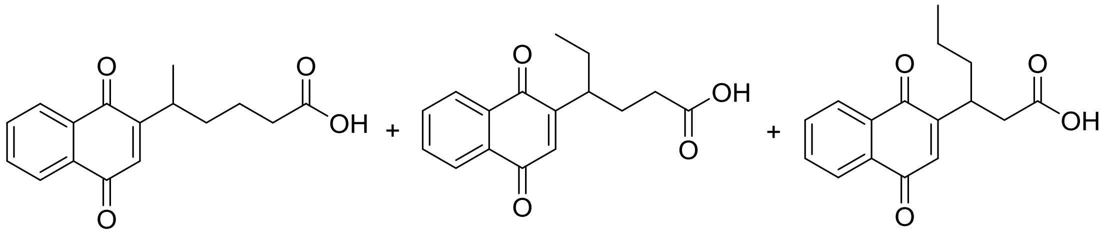

3-(1,4-Dioxo-1,4-dihydronaphthalen-2-yl)hexanoic acid (157-1) and 4-(1,4-dioxo-1,4-dihydronaphthalen-2-yl)hexanoic acid (157-2) and 5-(1,4-dioxo-1,4-dihydronaphthalen-2-yl)hexanoic acid (157-3) (157-1:157-2:157-3=1.9:1.6:1): *The General Procedure A* was applied with 1,4-naphthoquinone (80.6 mg, 0.5 mmol, 1 equiv.), hexanoic acid (127.9 μL, 1.0 mmol, 2 equiv.), $Fe(acac)_2$ (6.6 mg, 0.025 mmol, 0.05 equiv.), BCMOM (37.2 mg, 0.05 mmol, 0.1 equiv.), H_2O_2 (35%) (129 μL, 1.5 mmol, 3 equiv.), acetonitrile (4 mL) and water (4 mL) at 80 ℃ for 1 h. Column chromatography (PE/EA/FA, 10:1.5:1) afforded the title product as a dark brown wax (95.3 mg, 70%).

TLC: R_f = 0.36 (silica gel, PE/EA/FA, 10:1.5:1).

157-1 and **157-2** and **157-3:**

^{1}H NMR (400 MHz, $CDCl_3$): δ 8.20–7.97 (m, 2H), 7.76–7.71 (m, 2H), 6.75 (d, *J* = 4.1 Hz, 1H), 3.46 (dt, *J* = 12.2, 5.9 Hz, 0.22H) (C3), 3.16 (p, *J* = 7.3 Hz, 0.36H) (C2), 3.01 (p, *J* = 7.6, 6.9 Hz, 0.42H)(C1), 2.70 (d, *J* = 6.9 Hz, 0.42H), 2.42–2.33 (m, 0.74H), 2.33–2.24 (m, 0.8H), 2.04-2.00 (m, 0.57H), 1.92-1.86 (m, 0.63H), 1.75–1.58 (m, 2H), 1.19 (d, *J* = 6.5 Hz, 1.26H), 0.91-0.85 (m, 1.74H) ppm.

HRMS (ESI) *m/z* calcd. for $C_{16}H_{17}O_4^+$ [M + H$^+$] 273.1122, found: 273.1122.

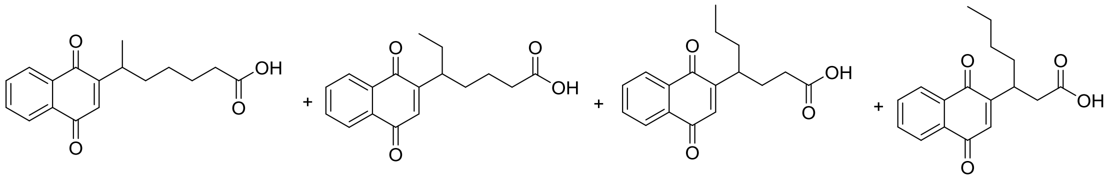

6-(1,4-dioxo-1,4-dihydronaphthalen-2-yl)heptanoic acid (158-1) and 5-(1,4-dioxo-1,4-dihydronaphthalen-2-yl)heptanoic acid (158-2) and 4-(1,4-dioxo-1,4-dihydronaphthalen-2-yl)heptanoic acid (158-3) and 3-(1,4-dioxo-1,4-dihydronaphthalen-2-yl)heptanoic acid (158-4) (158-1:158-2:158-3:158-4=1:0.8:0.7:0.5): *The General Procedure A* was applied with 1,4-naphthoquinone (80.6 mg, 0.5 mmol, 1 equiv.), heptanoic acid (144.7 μL, 1.0 mmol, 2 equiv.), $Fe(acac)_2$ (6.6 mg, 0.025 mmol, 0.05 equiv.), BCMOM (37.2 mg,

0.05 mmol, 0.1 equiv.), H_2O_2 (35%) (129 μL, 1.5 mmol, 3 equiv.), acetonitrile (4 mL) and water (4 mL) at 80 ℃ for 1 h. Column chromatography (PE/EA/FA, 10:1.5:1) afforded the title product as a dark brown wax (103.1 mg, 72%).

TLC: R_f = 0.37 (silica gel, PE/EA/FA, 10:1.5:1).

158-1 and **158-2** and **158-3** and **158-4:**

^{1}H NMR (400 MHz, $CDCl_3$): δ 8.07 (dt, *J* = 15.7, 3.6 Hz, 2H), 7.73 (dd, *J* = 5.9, 3.0 Hz, 2H), 6.75 (d, *J* = 5.3 Hz, 1H), 3.43 (p, *J* = 7.4 Hz, 0.17H) (C4), 3.17–3.11 (m, 0.24H) (C3), 3.11–3.06 (m, 0.27H) (C2), 2.99 (d, *J* = 8.9 Hz, 0.34H) (C1), 2.68 (d, *J* = 7.2 Hz, 0.34H), 2.31 (dd, *J* = 14.5, 7.2 Hz, 1.82H), 1.99 (dd, *J* = 13.7, 6.7 Hz, 0.5H), 1.86 (dt, *J* = 14.4, 7.6 Hz, 0.5H), 1.72–1.47 (m, 3.17H), 1.40–1.22 (m, 1.06H), 1.17 (d, *J* = 6.8 Hz, 1.02H), 0.86 (q, *J* = 7.8 Hz, 2.04H) ppm.

HRMS (ESI) *m/z* calcd. for $C_{17}H_{19}O_4^+$ [M + H^+] 287.1278, found: 287.1277.

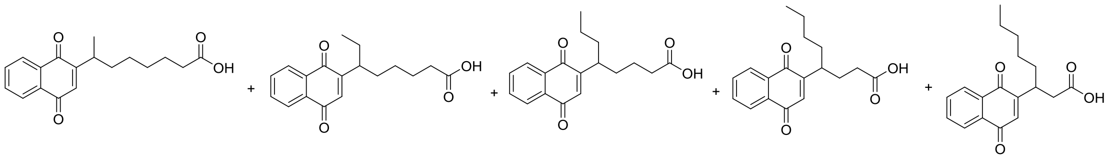

7-(1,4-dioxo-1,4-dihydronaphthalen-2-yl)octanoic acid (159-1) and **6-(1,4-dioxo-1,4-dihydronaphthalen-2-yl)octanoic acid and (159-2)** and **5-(1,4-dioxo-1,4-dihydronaphthalen-2-yl)octanoic acid (159-3)** and **4-(1,4-dioxo-1,4-dihydronaphthalen-2-yl)octanoic acid (159-4)** and **3-(1,4-dioxo-1,4-dihydronaphthalen-2-yl)octanoic acid (159-5) (159-1:159-2:159-3:159-4:159-5=2.7:2.4:2.1:1.8:0.7):** *The General Procedure A* was applied with 1,4-naphthoquinone (80.6 mg, 0.5 mmol, 1 equiv.), Octanoic acid (159.0 μL, 1.0 mmol, 2 equiv.), $Fe(acac)_2$ (6.6 mg, 0.025 mmol, 0.05 equiv.), BCMOM (37.2 mg, 0.05 mmol, 0.1 equiv.), H_2O_2 (35%) (129 μL, 1.5 mmol, 3 equiv.), acetonitrile (4 mL) and water (4 mL) at 80 ℃ for 1 h. Column chromatography (PE/EA/FA, 10:1.5:1) afforded the title product as a dark brown wax (112.6 mg, 75%).

TLC: R_f = 0.378 (silica gel, PE/EA/FA, 10:1.5:1).

159-1 and 159-2 and 159-3 and 159-4 and 159-5:

^{1}H NMR (400 MHz, $CDCl_3$): δ 8.12–8.05 (m, 2H), 7.77–7.72 (m, 2H), 6.78 (s, 0.07H) (C5), 6.76 (s, 0.21H) (C3), 6.75 (s, 0.24H) (C2), 6.74 (s, 0.27H) (C1), 6.72 (s, 0.18H) (C4), 3.43 (p, *J* = 7.3 Hz, 0.2H), 3.16–2.97 (m, 0.8H), 2.69 (d, *J* = 7.3 Hz, 0.4H), 2.57–2.51 (m, 0.25H), 2.38–2.24 (m, 1.6H), 2.04–1.95 (m, *J* = 13.4, 7.6 Hz, 0.32H), 1.91–1.81 (m, 0.46H), 1.72–1.50 (m, 4.09H), 1.41–1.24 (m, 2.20H), 1.17 (d, *J* = 6.9 Hz, 0.81H), 0.92–0.81 (m, 2.19H) ppm.

HRMS (ESI) *m/z* calcd. for $C_{18}H_{21}O_4^+$ [M + H^+] 301.1435, found: 301.1435.

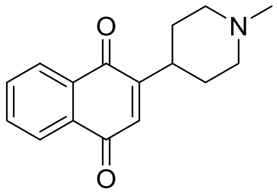

2-(1-Methylpiperidin-4-yl)naphthalene-1,4-dione (160): *The General Procedure B* was applied with 1,4-naphthoquinone (80.6 mg, 0.5 mmol, 1 equiv.), 1-methylpiperidine (125.3 μL, 1.0 mmol, 2 equiv.), H_2SO_4 (67.5 μL, 2.4 equiv.), $Fe(acac)_2$ (6.6 mg, 0.025 mmol, 0.05 equiv.), BCMOM (37.2 mg, 0.05 mmol, 0.1 equiv.), H_2O_2 (35%) (129 μL, 1.5 mmol, 3 equiv.), acetonitrile (4 mL) and water (4 mL) at 80 ℃ for 2 h. Column chromatography (PE/EA/TEA, 1:1:0.1) afforded the title product as a dark brown solid (102.1 mg, 80%).

TLC: R_f = 0.38 (silica gel, PE/EA/TEA, 4:1:0.1).

^{1}H NMR (400 MHz, $CDCl_3$) δ 8.12–8.08 (m, 1H), 8.07–8.03 (m, 1H), 7.79–7.69 (m, 2H), 6.76 (s, 1H), 2.98 (d, *J* = 11.9 Hz, 2H), 2.93–2.82 (m, 1H), 2.32 (s, 3H), 2.11 (t, *J* = 11.9 Hz, 2H), 1.83 (d, *J* = 12.8 Hz, 2H), 1.72–1.53 (m, 2H) ppm.

^{13}C NMR (100 MHz, $CDCl_3$) δ 185.3, 184.7, 154.8, 133.7, 133.7, 133.29, 132.33, 131.8, 126.7, 126.0, 55.8, 46.3, 34.3, 31.2 ppm.

HRMS (ESI) *m/z* calcd. for $C_{16}H_{18}NO_2^+$ [M + H^+] 256.1332, found: 256.1331.

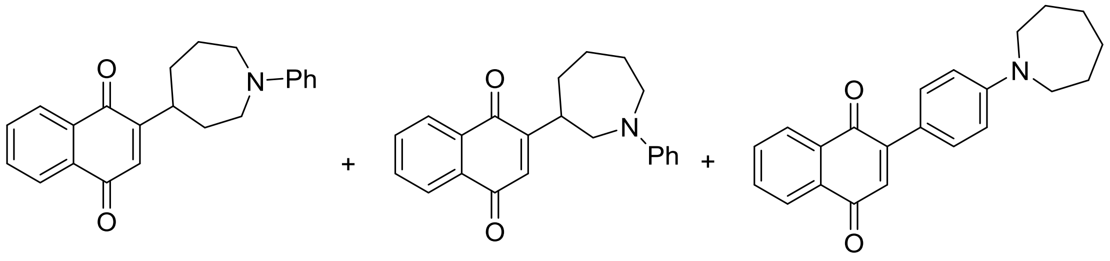

2-(1-Phenylazepan-4-yl)naphthalene-1,4-dione (161-1) and 2-(1-phenylazepan-3-yl)naphthalene-1,4-dione (161-2) and 2-(4-(azepan-1-yl)phenyl)naphthalene-1,4-dione (161-3) (161-1:161-2:161-3=6:3.1:4.3): *The General Procedure B* was applied with 1,4-naphthoquinone (80.6 mg, 0.5 mmol, 1 equiv.), 1-phenylazepane (175.9 mg, 1.0 mmol, 2 equiv.), H_2SO_4 (67.5 μL, 2.4 equiv.), $Fe(acac)_2$ (6.6 mg, 0.025 mmol, 0.05 equiv.), BCMOM (37.2 mg, 0.05 mmol, 0.1 equiv.), H_2O_2 (35%) (129 μL, 1.5 mmol, 3 equiv.), acetonitrile (4 mL) and water (4 mL) at 80 ℃ for 1 h. Column chromatography (PE/EA/TEA, 40:1:0.1) afforded the title product as a purple solid (140.9 mg, 85%).

TLC: R_f = 0.45 (silica gel, PE/EA/TEA, 20:1:0.1).

^{1}H NMR (400 MHz, $CDCl_3$): δ 8.20–8.14 (m, 0.5H), 8.11–8.08 (m, 1.3H), 8.07–8.02 (m, 0.7H), 7.78–7.67 (m, 2.5H), 7.59 (d, *J* = 9.0 Hz, 1.0H), 7.23 (d, *J* = 8.5 Hz, 1.5H), 7.02 (s, 0.43H) (C3), 6.84–6.64 (m, 3H), 3.74 (t, *J* = 4.3 Hz, 0.31H) (C2), 3.70 (t, *J* = 4.3 Hz, 0.6H) (C3), 3.60 (dt, *J* = 14.1, 5.1 Hz, 1H), 3.55–3.50 (m, 2.2H), 3.49–3.41 (m, 1H), 3.10 (t, *J* = 11.2 Hz, 0.8H), 2.16–2.09 (m, 1H), 2.05–1.99 (m, 1H), 1.88–1.79 (m, 4H), 1.61–1.53 (m, 3H) ppm.

HRMS (ESI) *m/z* calcd. for $C_{22}H_{22}NO_2^+$ [M + H^+] 332.1645, found: 332.1642.

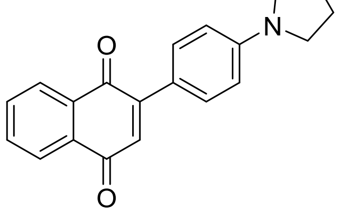

2-(4-(Pyrrolidin-1-yl)phenyl)naphthalene-1,4-dione (162): *The General Procedure*

B was applied with 1,4-naphthoquinone (80.6 mg, 0.5 mmol, 1 equiv.), 1-phenylpyrrolidine (145.4 µL, 1.0 mmol, 2 equiv.), H_2SO_4 (67.5 µL, 2.4 equiv.), $Fe(acac)_2$ (6.6 mg, 0.025 mmol, 0.05 equiv.), BCMOM (37.2 mg, 0.05 mmol, 0.1 equiv.), H_2O_2 (35%) (129 µL, 1.5 mmol, 3 equiv.), acetonitrile (4 mL) and water (4 mL) at 80 ℃ for 2 h. Column chromatography (PE/EA/TEA, 6:1:0.1) afforded the title product as a purple solid (115.3 mg, 76%).

TLC: R_f = 0.40 (silica gel, PE/EA/TEA, 4:1:0.1).

^{1}H NMR (400 MHz, $CDCl_3$): δ 8.22–8.14 (m, 1H), 8.13–8.03 (m, 1H), 7.78–7.67 (m, 2H), 7.60 (d, *J* = 8.6 Hz, 2H), 7.01 (s, 1H), 6.61 (d, *J* = 8.5 Hz, 2H), 3.36 (s, 4H), 2.03 (s, 4H) ppm.

^{13}C NMR (100 MHz, $CDCl_3$) δ 185.6, 185.2, 149.3, 147.5, 133.5, 133.3, 132.9, 132.3, 131.1, 130.5, 126.9, 125.6, 119.8, 111.6, 47.5, 25.5 ppm.

HRMS (ESI) *m/z* calcd. for $C_{20}H_{18}NO_2^+$ [M + H^+] 304.1332, found: 304.1331.

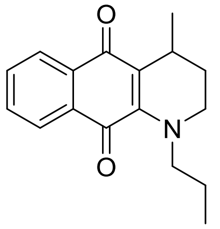

4-Methyl-1-propyl-1,2,3,4-tetrahydrobenzo[g]quinoline-5,10-dione (163): *The General Procedure C* was applied with 1,4-naphthoquinone (80.6 mg, 0.5 mmol, 1 equiv.), N-propylbutan-1-amine (115.2 mg, 1.0 mmol, 2 equiv.), H_2SO_4 (67.5 µL, 2.4 equiv.), $FeCl_2$ (3.2 mg, 0.025 mmol, 0.05 equiv.), BCMOM (37.2 mg, 0.05 mmol, 0.1 equiv.), H_2O_2 (35%) (129 µL, 1.5 mmol, 3 equiv.), acetonitrile (4 mL) and water (4 mL) at 80 ℃ for 1 h. Column chromatography (PE/EA/TEA, 20:1:0.1 to 10:1:0.1) afforded the title product as a red solid (92.9 mg, 69%).

TLC: R_f = 0.42 (silica gel, PE/EA/TEA, 10:1:0.1).

^{1}H NMR (400 MHz, $CDCl_3$): δ 8.02 (d, *J* = 7.6 Hz, 1H), 7.89 (d, *J* = 7.7 Hz, 1H), 7.63 (t, *J* = 6.9 Hz, 1H), 7.54 (t, *J* = 7.5 Hz, 1H), 3.67–3.51 (m, 2H), 3.48-3.41 (m, 4.1

Hz, 1H), 3.38–3.24 (m, 2H), 1.87–1.66 (m, 4H), 1.18 (d, J = 6.9 Hz, 3H), 0.95 (t, J = 7.4 Hz, 3H) ppm.

^{13}C NMR (100 MHz, $CDCl_3$): δ 183.7, 180.6, 148.1, 133.5, 133.0, 132.5, 131.4, 125.8, 125.2, 120.7, 55.67, 46.3, 26.9, 24.5, 22.3, 21.1, 11.3 ppm.

HRMS (ESI) *m/z* calcd. for $C_{17}H_{20}NO_2^+$ [M + H$^+$] 270.1489, found: 270.1487.

IR (KBr, cm^{-1}): ν_{max} 2958, 2925, 2868, 1667, 1615, 1592, 1462, 1423, 1376, 1277, 1215, 1164, 1084, 997, 795, 723, 694, 657.

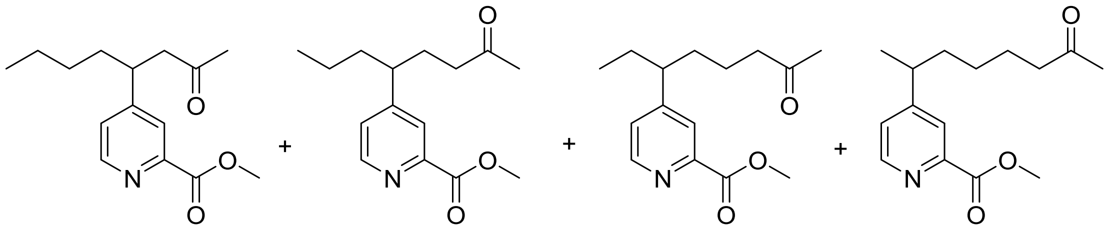

Methyl 4-(2-oxooctan-4-yl)picolinate (164-1) and **methyl 4-(7-oxooctan-4-yl)picolinate (164-2)** and **methyl methyl 4-(7-oxooctan-3-yl)picolinate (164-3)** and **methyl 4-(7-oxooctan-2-yl)picolinate (164-4) (10-1:10-2:10-3=4:7:10:15)**: *The General Procedure D* was applied with methyl picolinate (61.5 μL, 0.5 mmol, 1 equiv.), H_2SO_4 (67.5 μL, 2.4 equiv.), $FeCl_2$ (3.2 mg, 0.025 mmol, 0.05 equiv.), BCMOM (37.2 mg, 0.05 mmol, 0.1 equiv.), octan-2-one (156.6 μL, 1.0 mmol, 2 equiv.), H_2O_2 (35%) (129 μL, 1.5 mmol, 3 equiv.), acetonitrile (4 mL) and water (4 mL) at 80 ℃ under N_2 for 3 h. Column chromatography (PE/EA/TEA, from 10:4:0.1 to 10:8:0.1) afforded the title product as a yellow liquid (61.9 mg, 47%), unknown compound.

TLC: R_f = 0.49 (silica gel, PE/EA/TEA, 10:9:0.1).

^{1}H NMR (400 MHz, $CDCl_3$): δ 8.62-8.57 (m, 1H), 8.00–7.83 (m, 1H), 7.28–7.19 (m, 1H), 3.97 (s, 3H), 2.79–2.69 (m, 1H), 2.50-2.47 (m, 0.5H), 2.35 (q, J = 6.8, 6.0 Hz, 1.5H), 2.28–2.14 (m, 0.7H), 2.04 (dd, J = 13.2, 7.7 Hz, 3.3H), 1.81–1.57 (m, 2H), 1.56–1.34 (m, 2H), 1.22 (d, J = 7.0 Hz, 1.5H), 0.86–0.79 (m, 0.7H), 0.79–0.74 (m, 0.4H), 0.71 (t, J = 7.4 Hz, 1H) ppm.

HRMS (ESI) *m/z* calcd. for $C_{15}H_{22}NO_3^+$ [M + H$^+$] 264.1594, found: 264.1592.

Copies of NMR Spectra

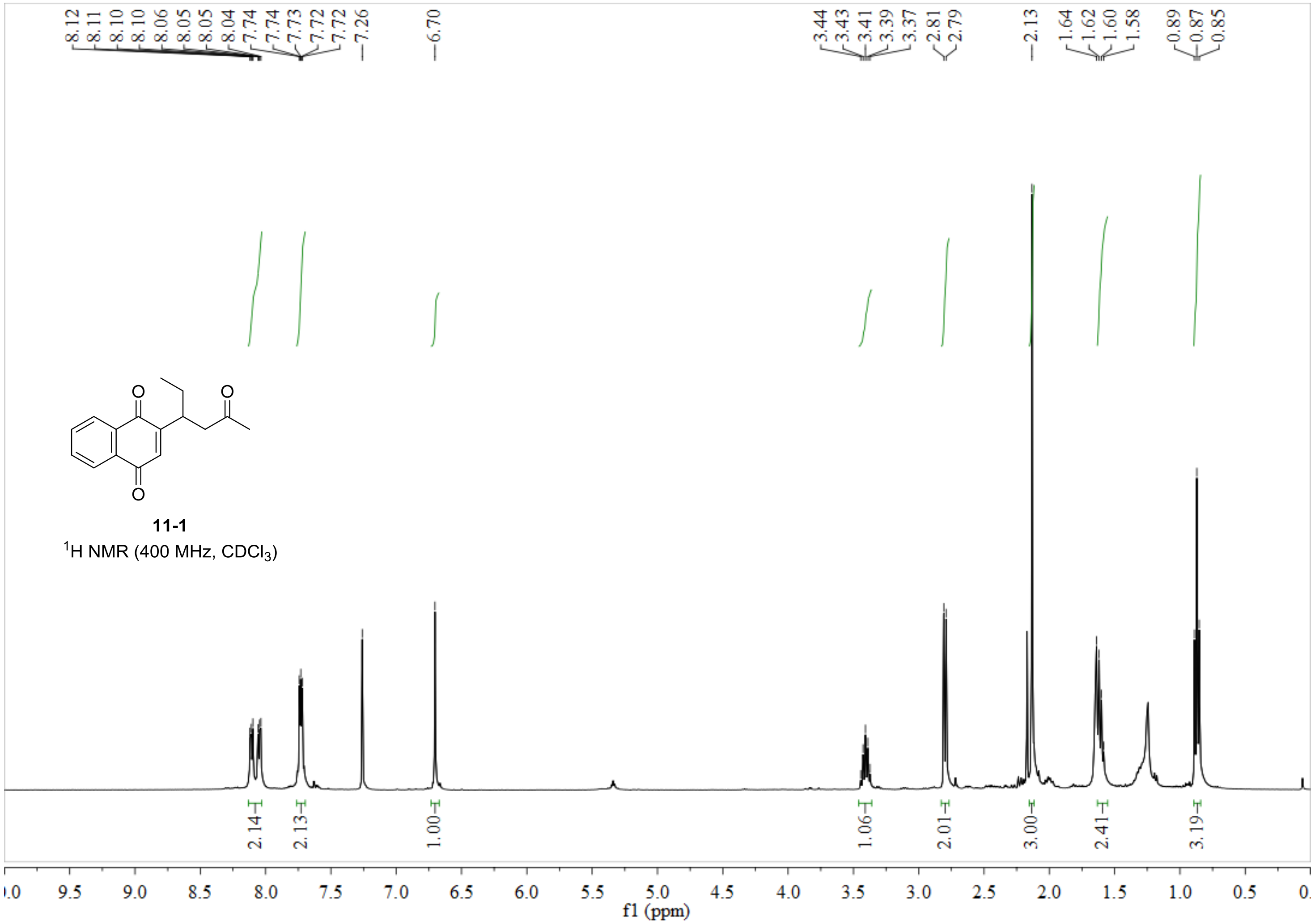

8.12
8.11
8.10
8.10
8.06
8.05
8.05
8.04
7.74
7.74
7.73
7.72
7.72
7.26
6.70
3.44
3.43
3.41
3.39
3.37
2.81
2.79
2.13
1.64
1.62
1.60
1.58
0.89
0.87
0.85
11-1
¹H NMR (400 MHz, CDCl3)
2.14
2.13
1.00
1.06
2.01
3.00
2.41
3.19
f1 (ppm)

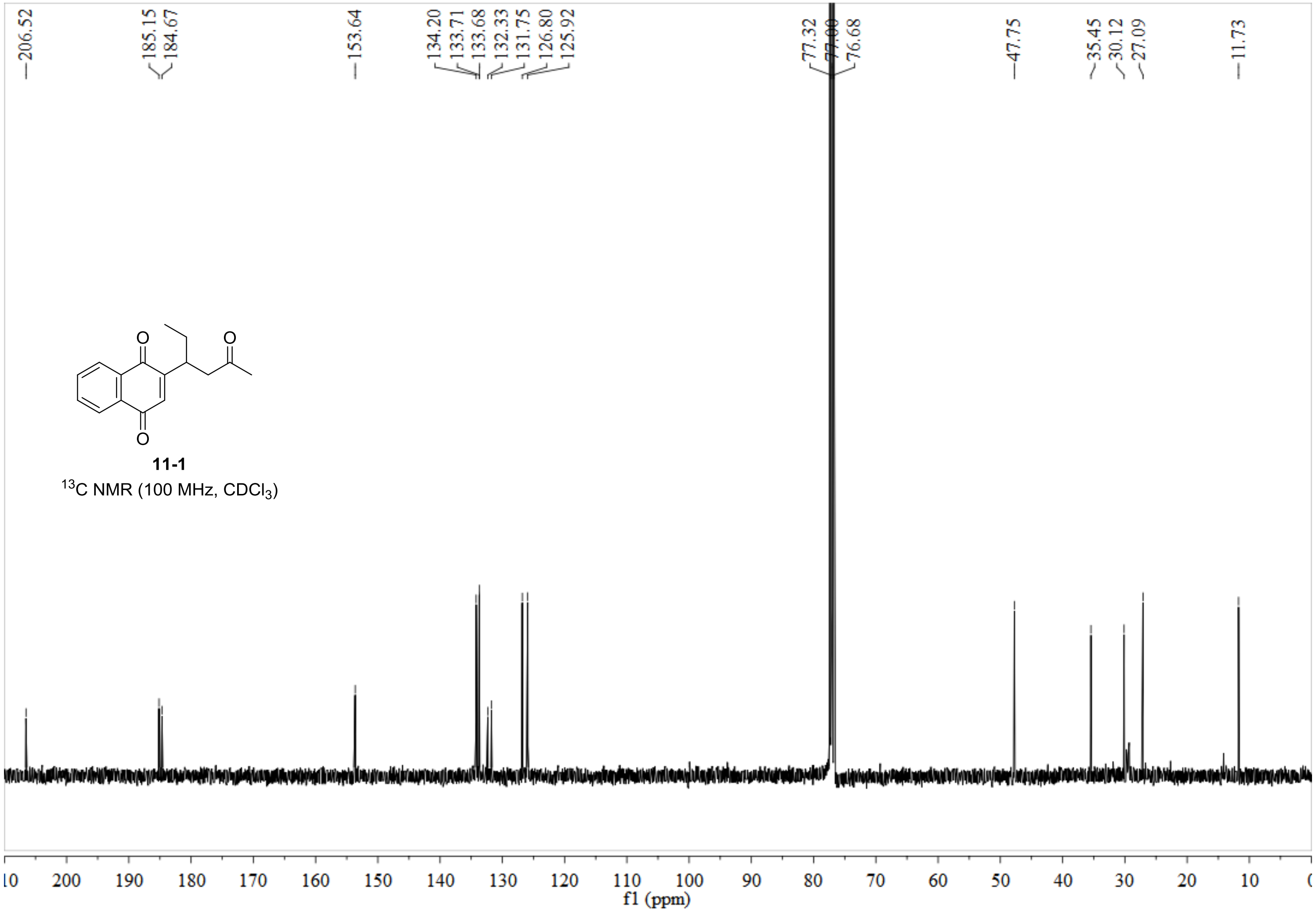
206.52
185.15
184.67
153.64
134.20
133.71
133.68
132.33
131.75
126.80
125.92
77.32
77.00
76.68
47.75
35.45
30.12
27.09
11.73
11-1
^{13}C NMR (100 MHz, $CDCl_3$)
f1 (ppm)

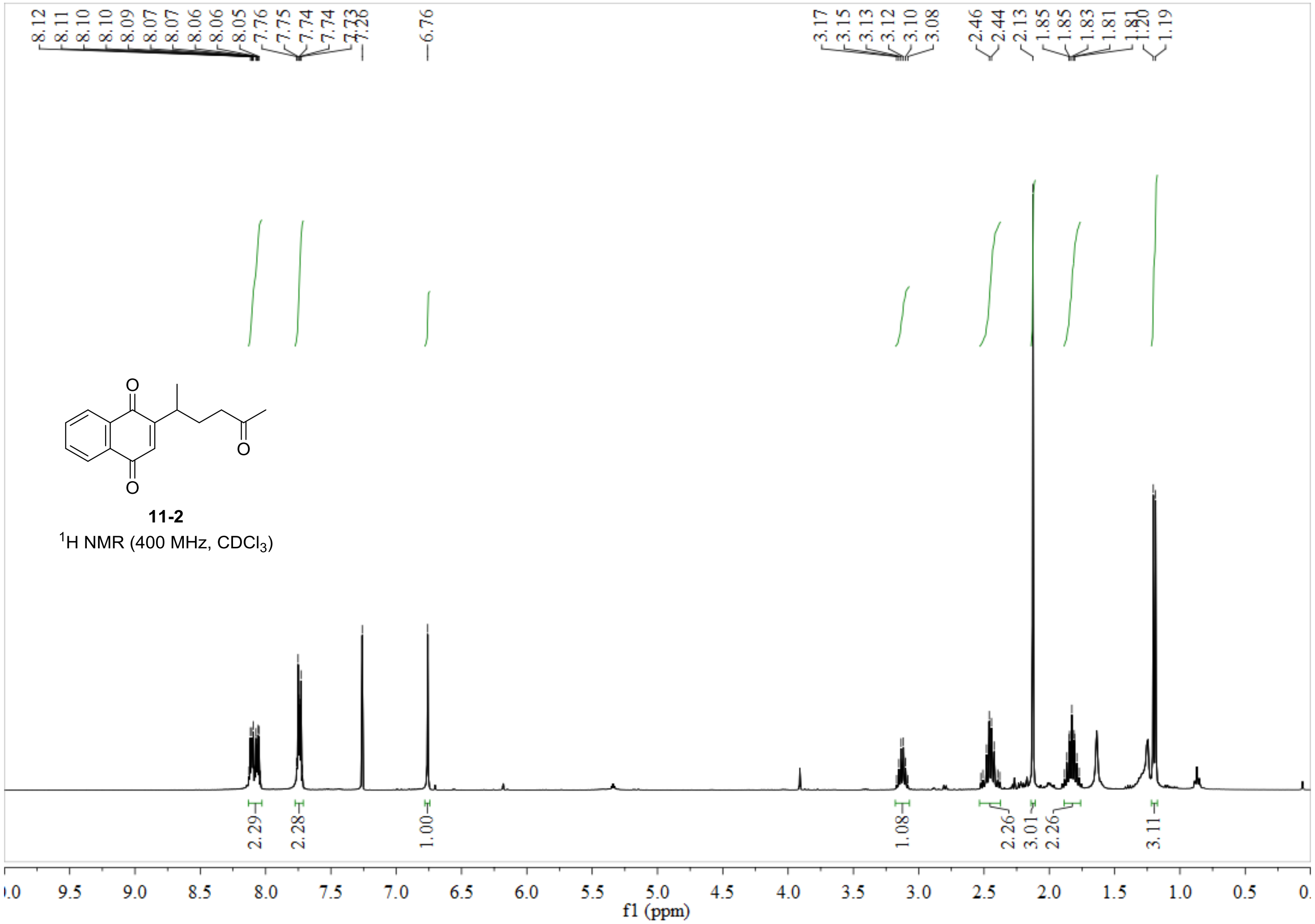

11-2

^1H NMR (400 MHz, $CDCl_3$)

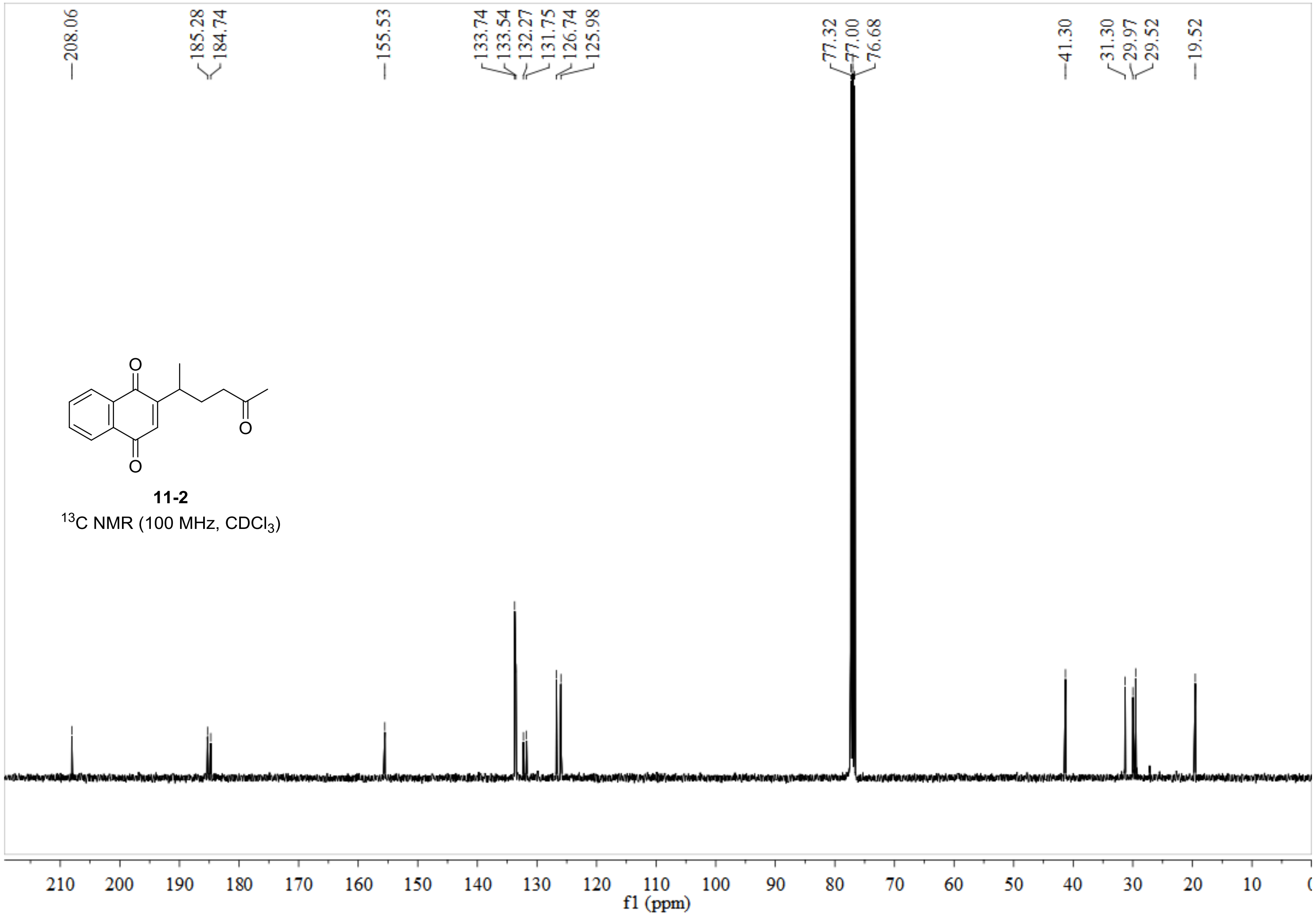

11-2

^{13}C NMR (100 MHz, $CDCl_3$)

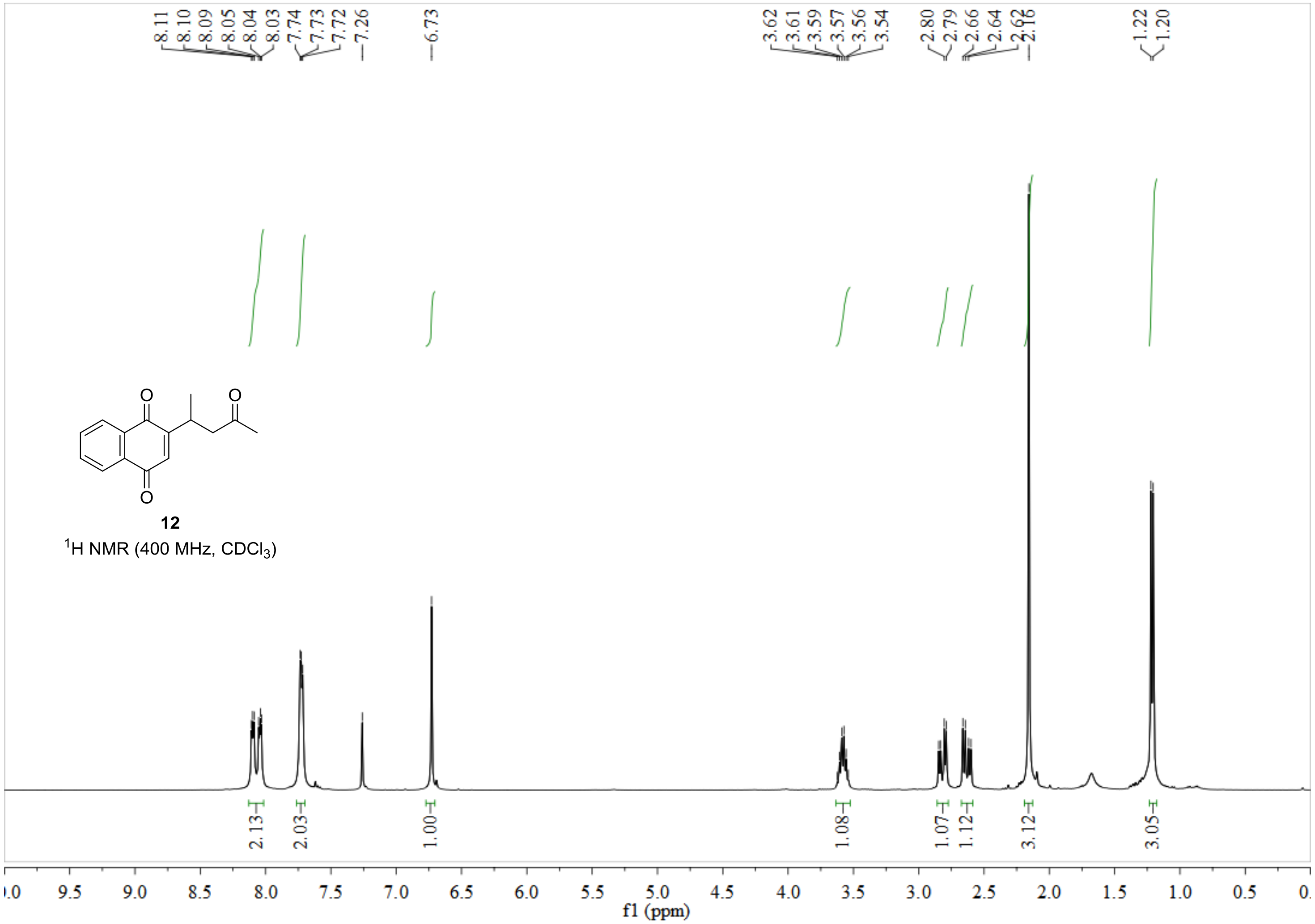
12
^{1}H NMR (400 MHz, $CDCl_3$)
f1 (ppm)

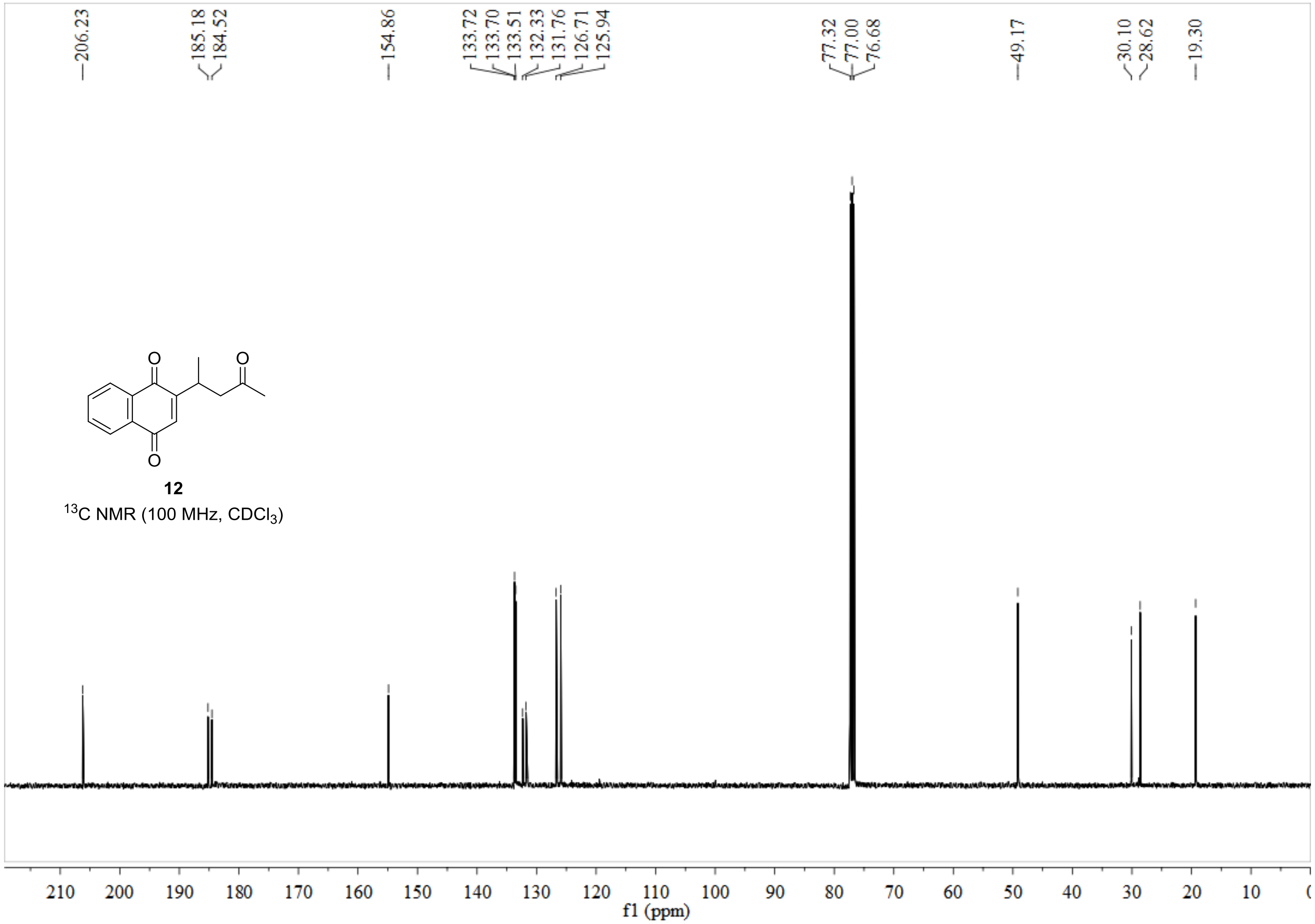

12

^{13}C NMR (100 MHz, $CDCl_3$)

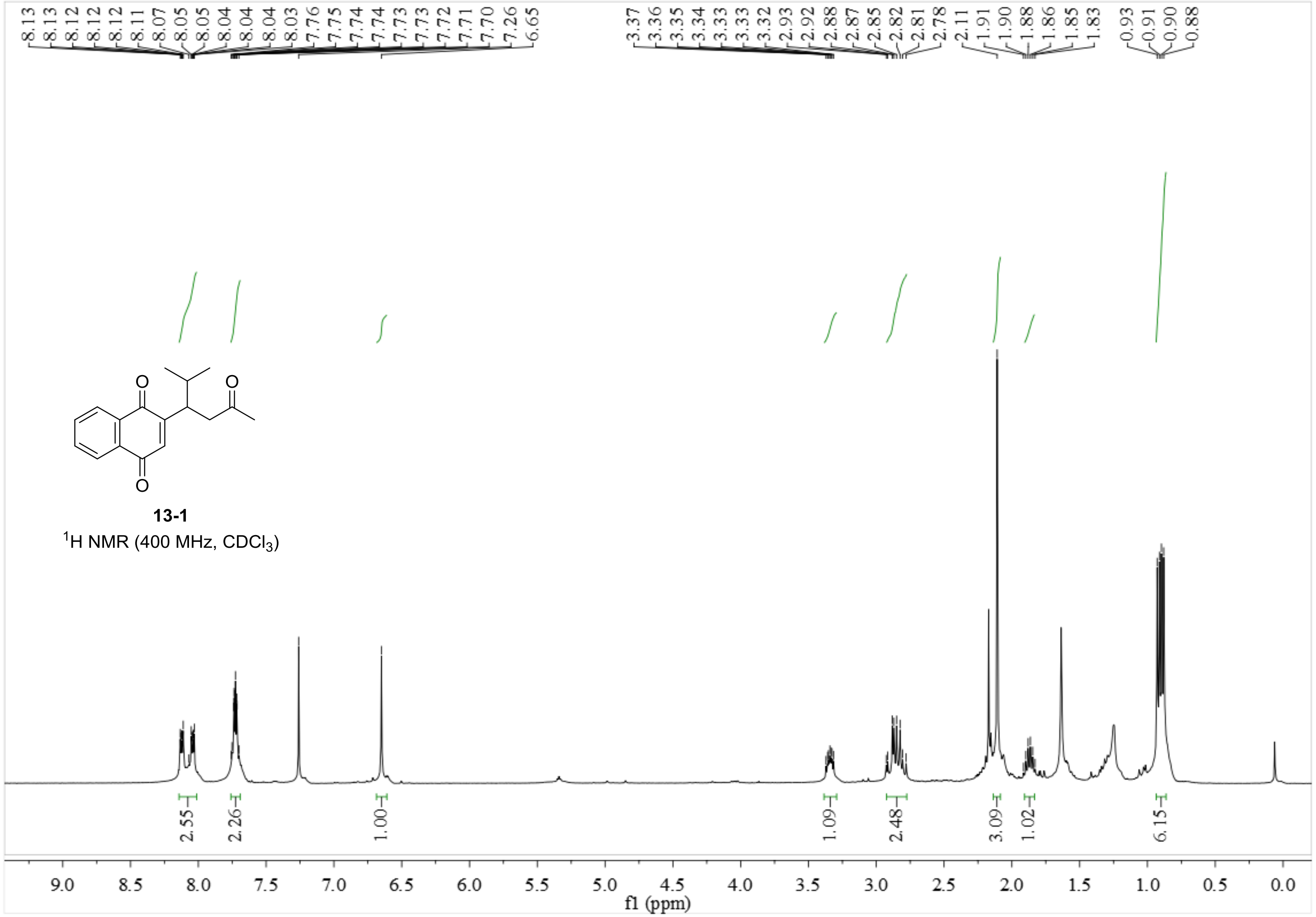
8.13 8.13 8.12 8.12 8.12 8.11 8.07 8.05 8.05 8.04 8.04 8.04 8.03 7.76 7.75 7.74 7.74 7.73 7.73 7.72 7.71 7.70 7.26 6.65
3.37 3.36 3.35 3.34 3.33 3.33 3.32 2.93 2.92 2.88 2.87 2.85 2.82 2.81 2.78 2.11 1.91 1.90 1.88 1.86 1.85 1.83 0.93 0.91 0.90 0.88
13-1
1H NMR (400 MHz, CDCl3)
2.55 2.26 1.00 1.09 2.48 3.09 1.02 6.15
9.0 8.5 8.0 7.5 7.0 6.5 6.0 5.5 5.0 4.5 4.0 3.5 3.0 2.5 2.0 1.5 1.0 0.5 0.0
f1 (ppm)

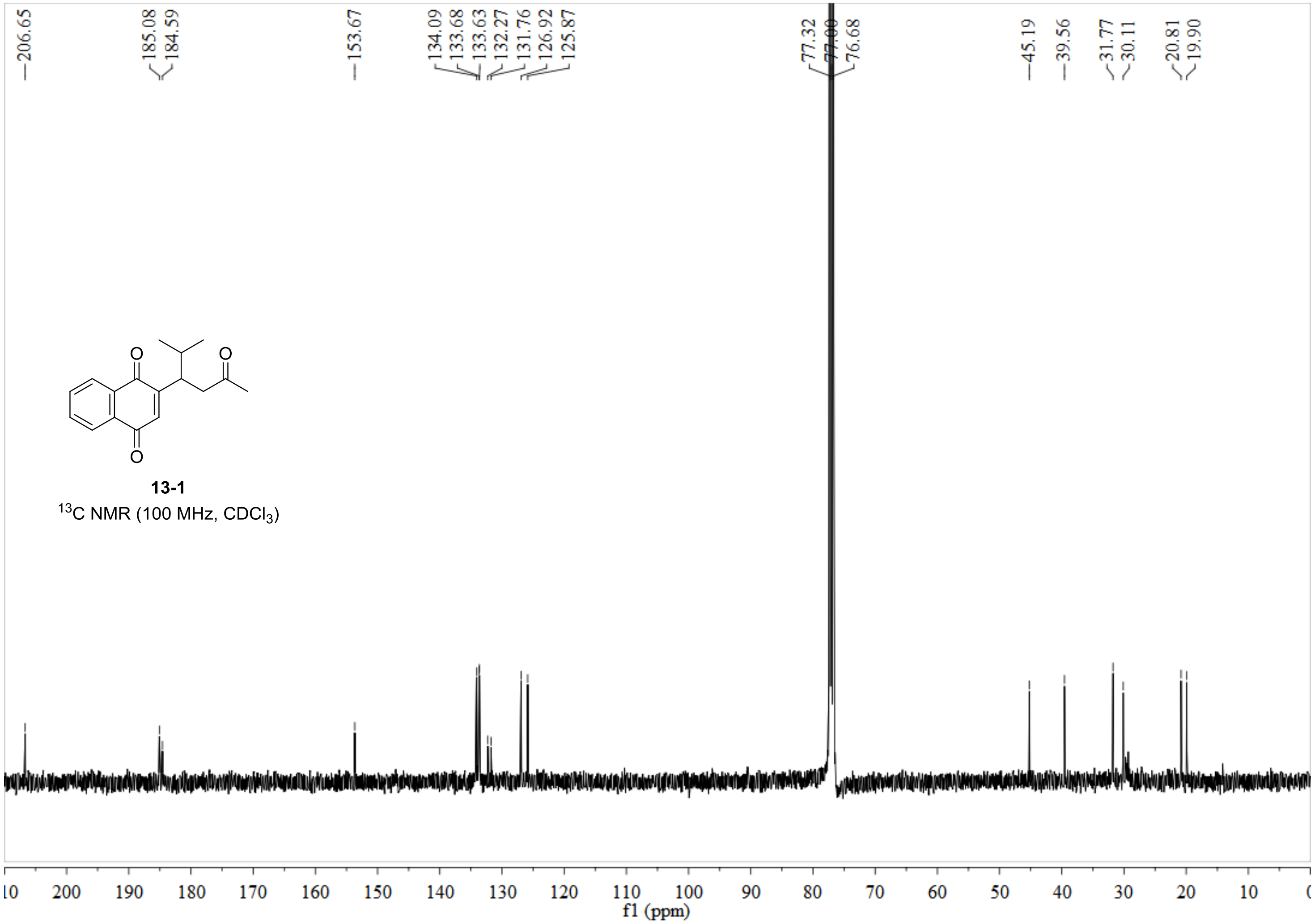
206.65
185.08
184.59
153.67
134.09
133.68
133.63
132.27
131.76
126.92
125.87
77.32
77.00
76.68
45.19
39.56
31.77
30.11
20.81
19.90
13-1
13C NMR (100 MHz, CDCl3)
f1 (ppm)

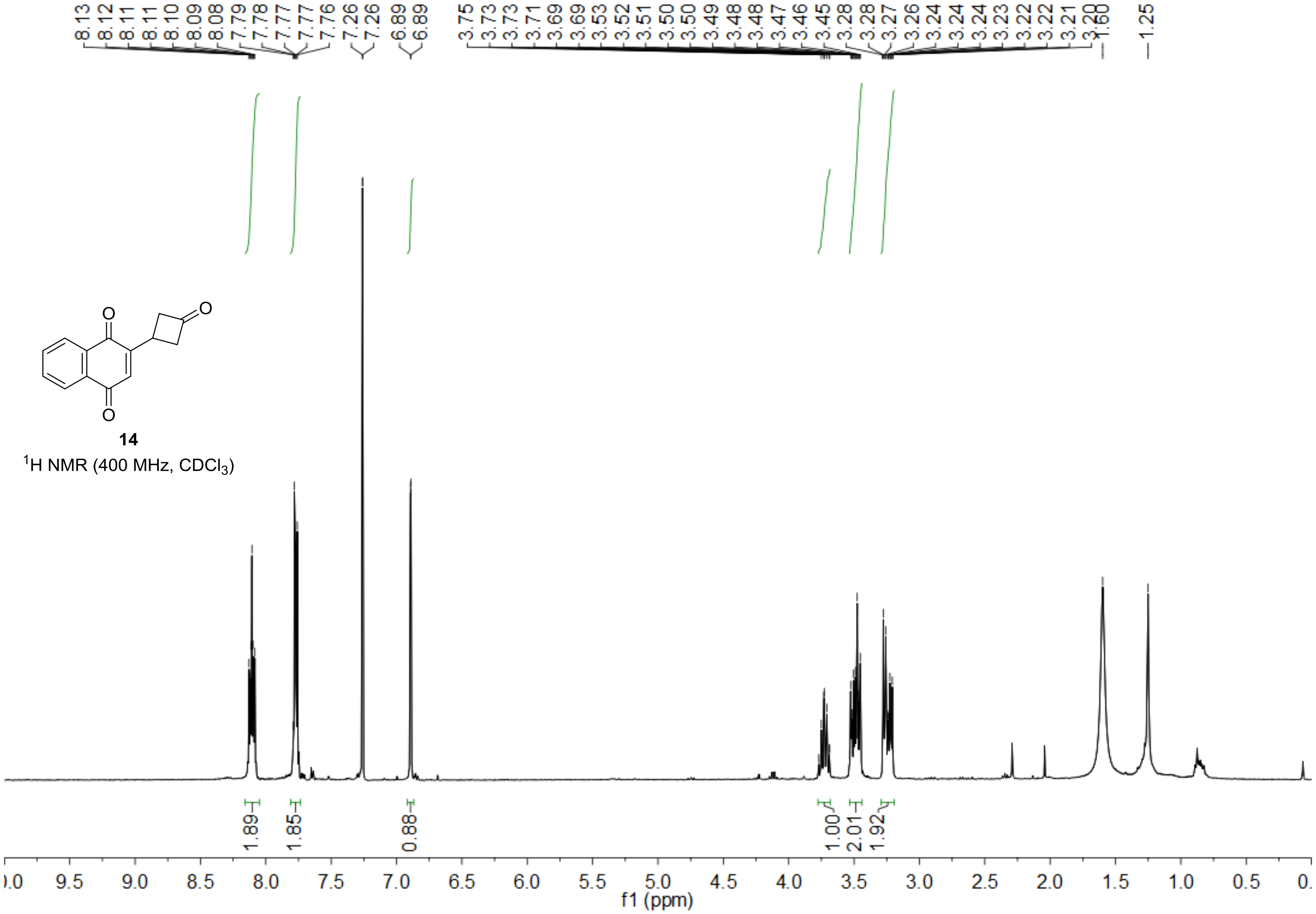

14
^1H NMR (400 MHz, $CDCl_3$)
f1 (ppm)

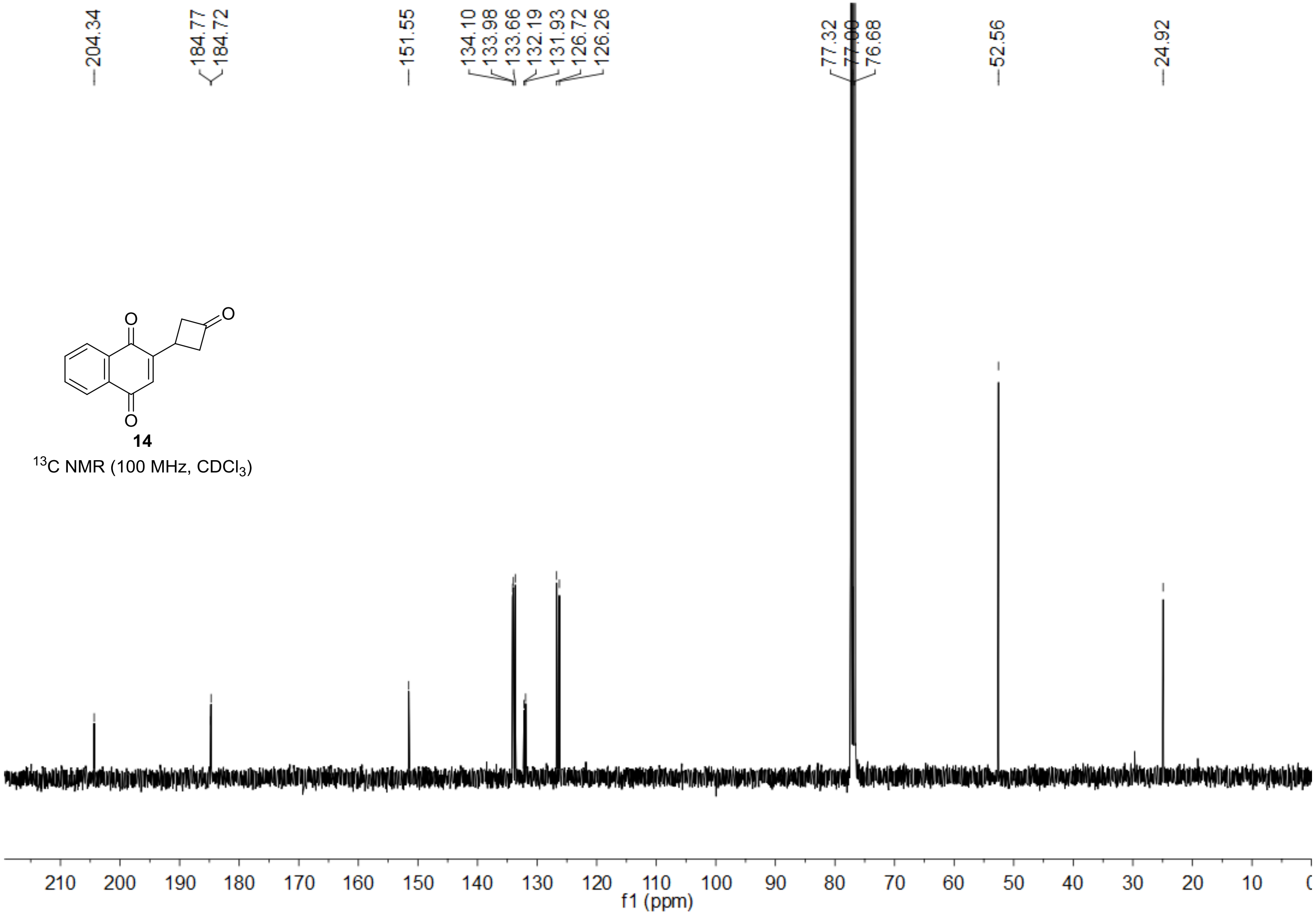

14

^{13}C NMR (100 MHz, $CDCl_3$)

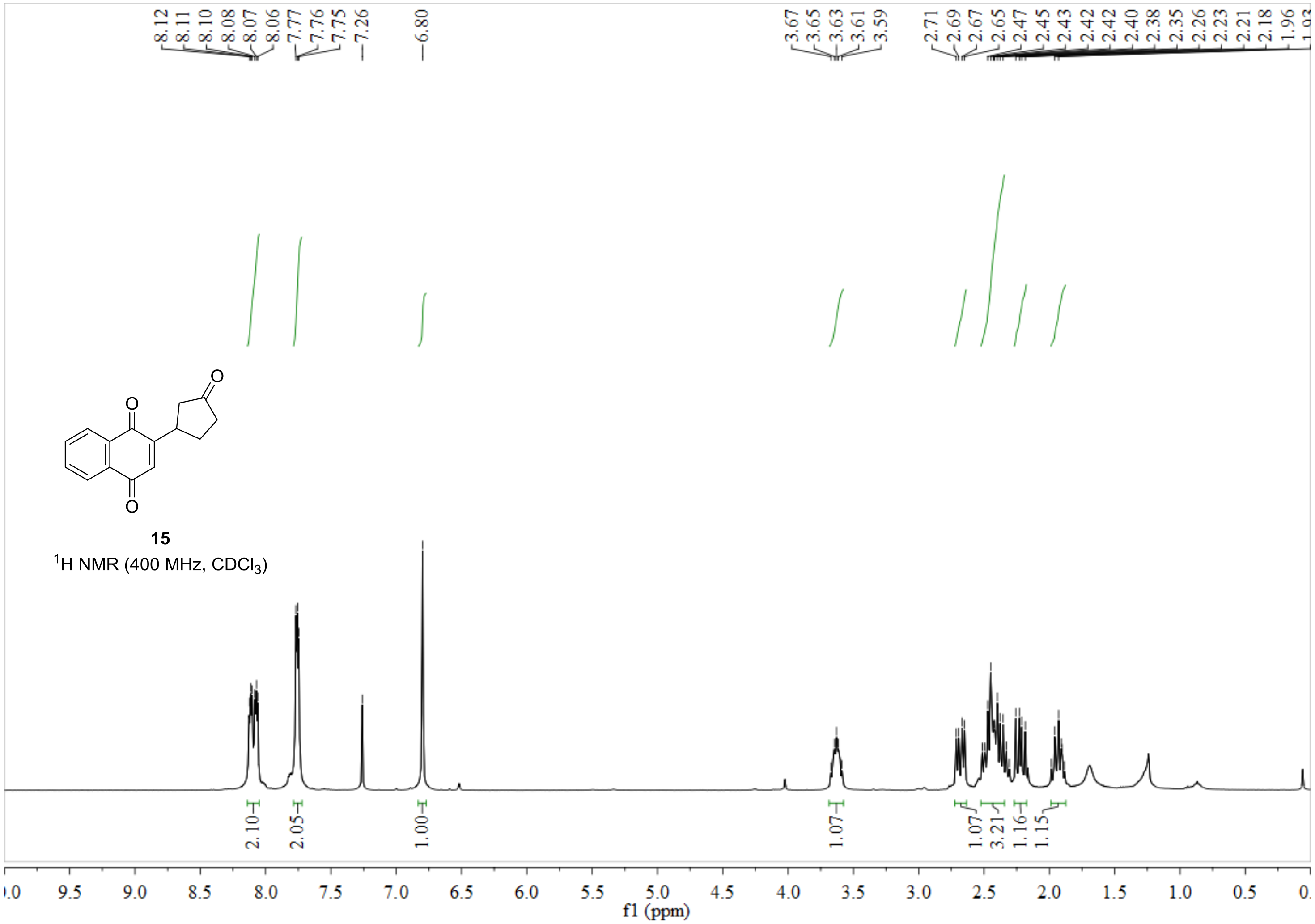
15
¹H NMR (400 MHz, CDCl3)
8.12
8.11
8.10
8.08
8.07
8.06
7.77
7.76
7.75
7.26
6.80
3.67
3.65
3.63
3.61
3.59
2.71
2.69
2.67
2.65
2.47
2.45
2.43
2.42
2.42
2.40
2.38
2.35
2.26
2.23
2.21
2.18
1.96
2.10
2.05
1.00
1.07
1.07
3.21
1.16
1.15
f1 (ppm)

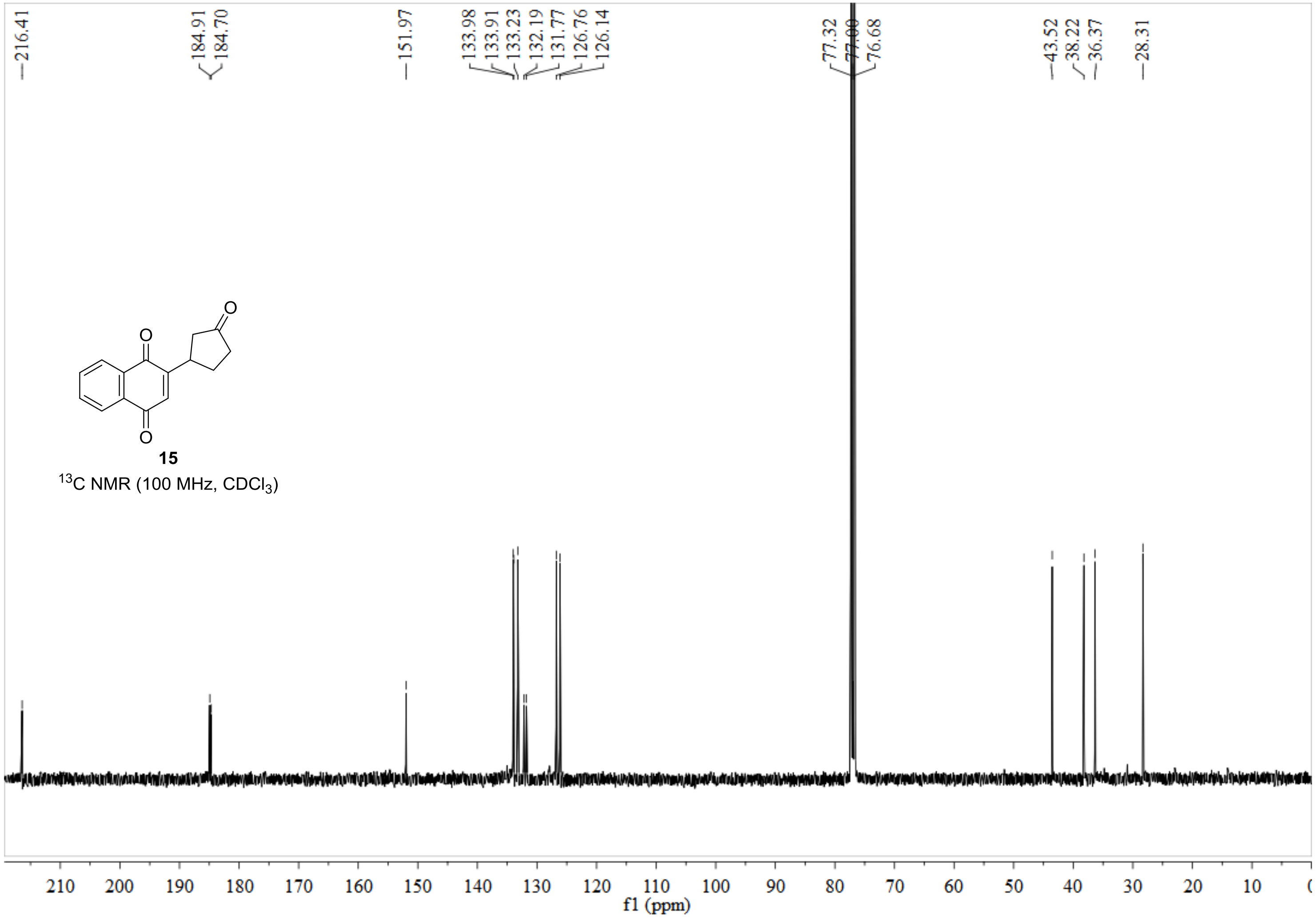

15

^{13}C NMR (100 MHz, $CDCl_3$)

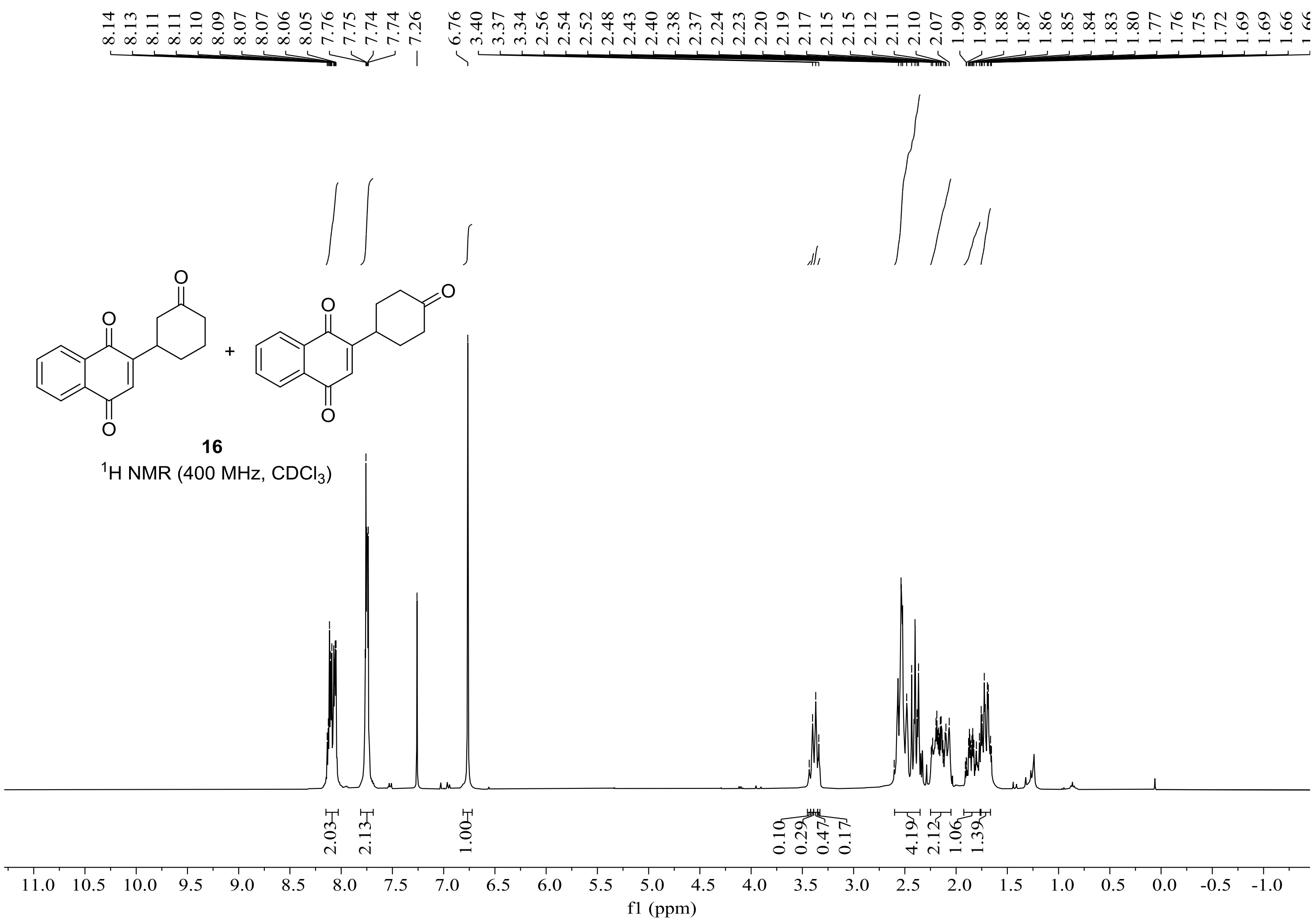
16
1H NMR (400 MHz, CDCl3)
f1 (ppm)

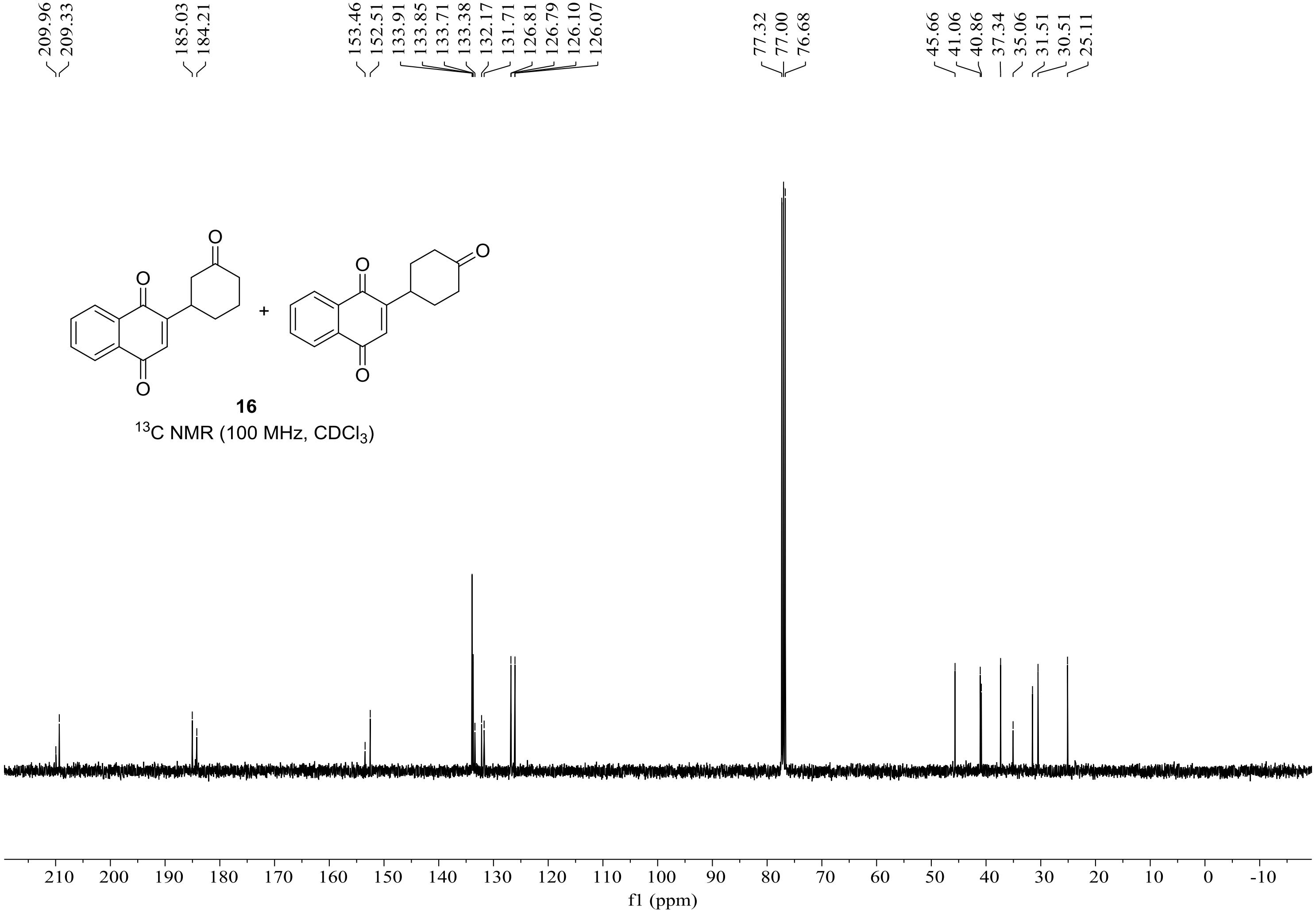

209.96
209.33
185.03
184.21
153.46
152.51
133.91
133.85
133.71
133.38
132.17
131.71
126.81
126.79
126.10
126.07
77.32
77.00
76.68
45.66
41.06
40.86
37.34
35.06
31.51
30.51
25.11
+
16
^{13}C NMR (100 MHz, $CDCl_3$)
210 200 190 180 170 160 150 140 130 120 110 100 90 80 70 60 50 40 30 20 10 0 -10
f1 (ppm)

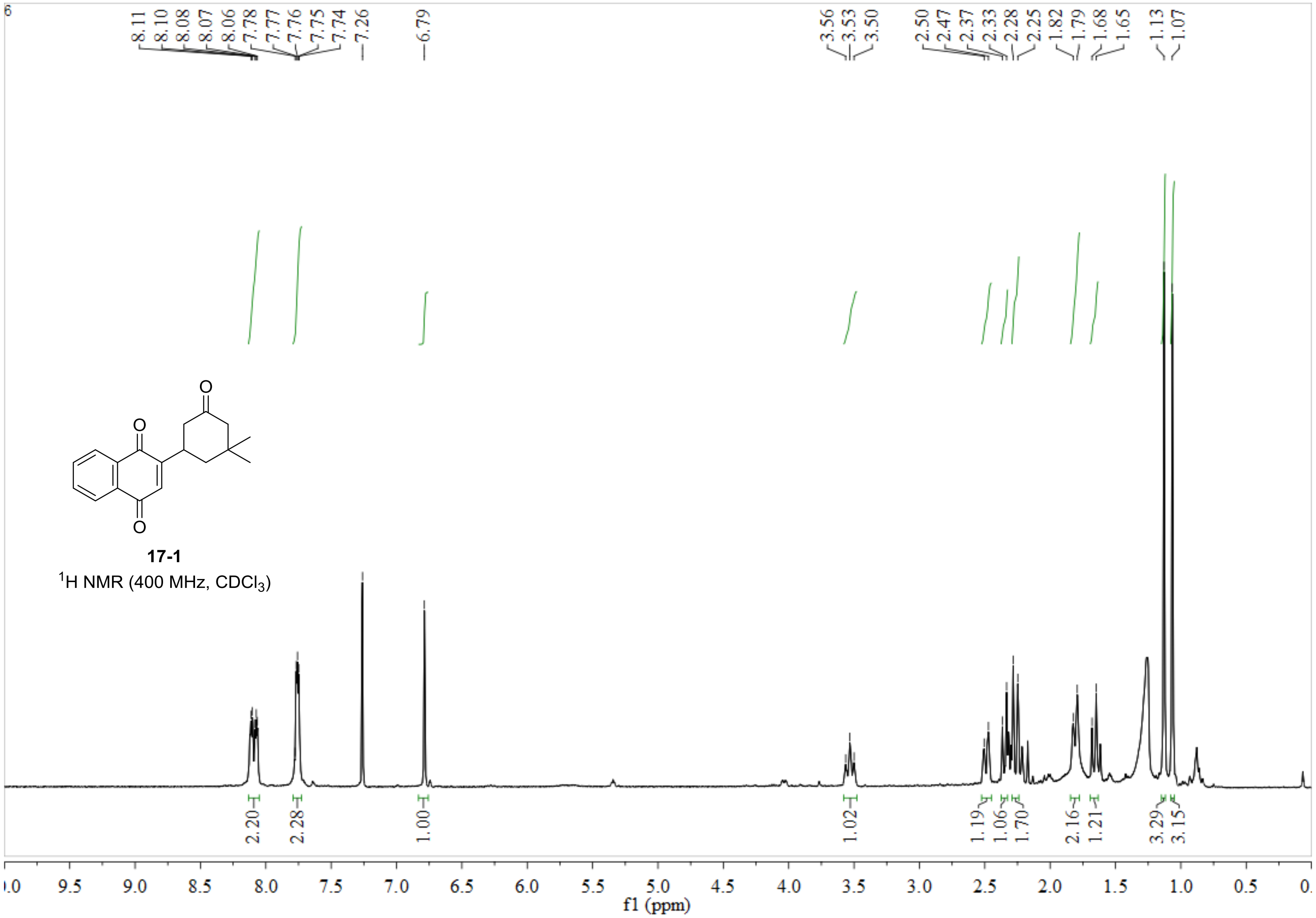

17-1
^{1}H NMR (400 MHz, CDCl$_3$)
8.11 8.10 8.08 8.07 8.06 7.78 7.77 7.76 7.75 7.74 7.26
6.79
3.56 3.53 3.50
2.50 2.47 2.37 2.33 2.28 2.25 1.82 1.79 1.68 1.65 1.13 1.07
2.20 2.28 1.00 1.02 1.19 1.06 1.70 2.16 1.21 3.29 3.15
f1 (ppm)

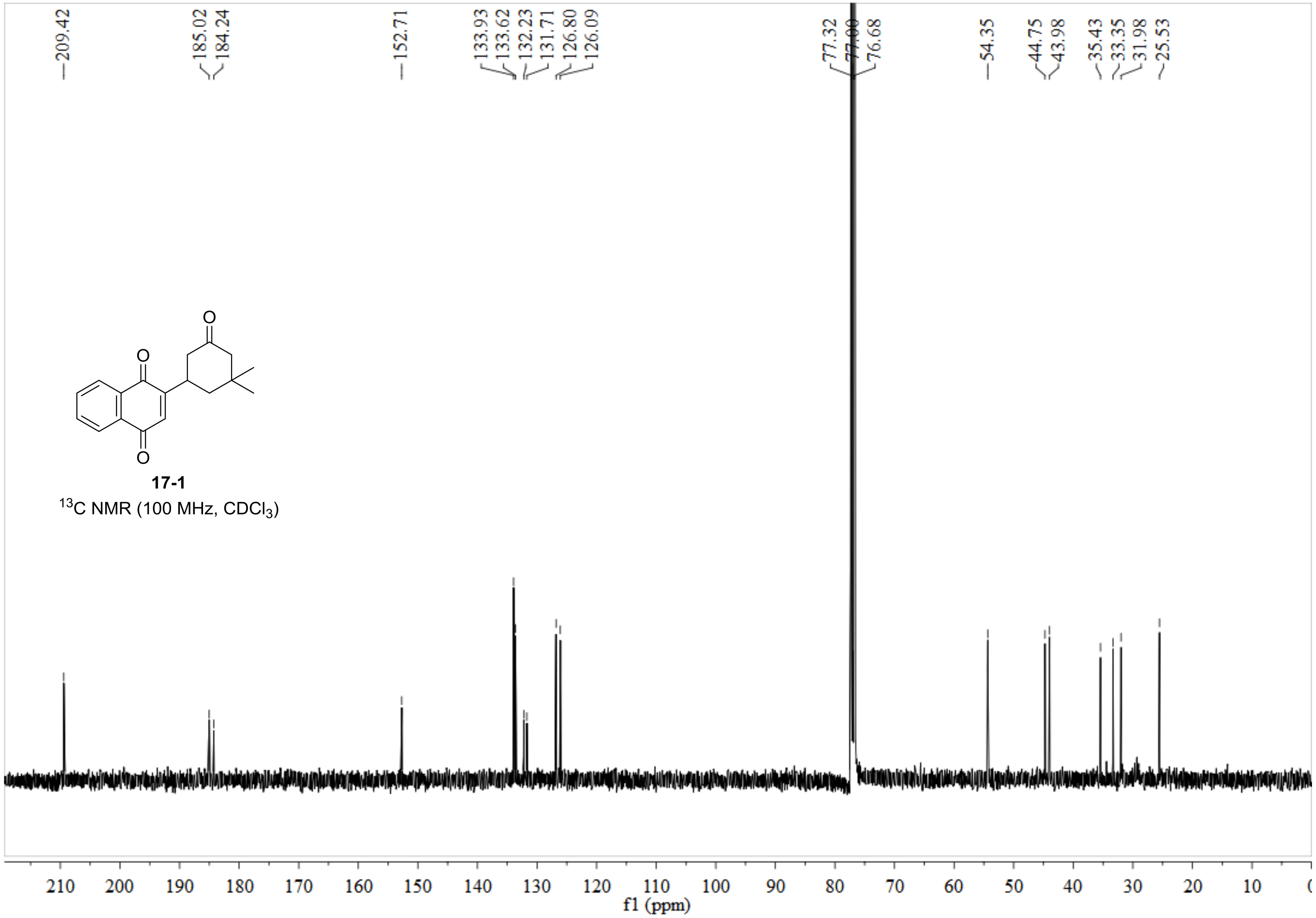
209.42
185.02
184.24
152.71
133.93
133.62
132.23
131.71
126.80
126.09
77.32
77.00
76.68
54.35
44.75
43.98
35.43
33.35
31.98
25.53
O
O
O
17-1
13C NMR (100 MHz, CDCl3)
210
200
190
180
170
160
150
140
130
120
110
100
90
80
70
60
50
40
30
20
10
f1 (ppm)

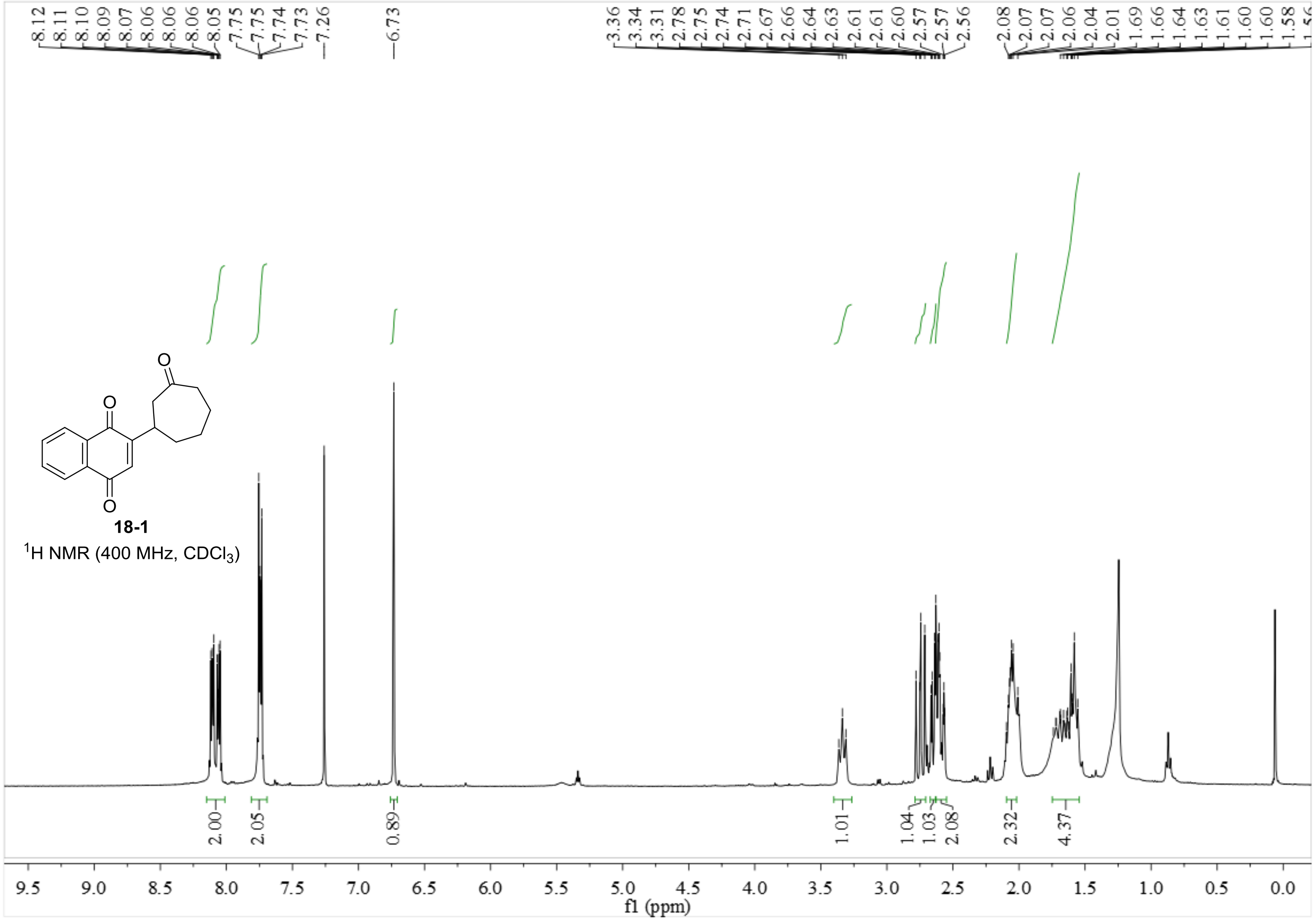
18-1
1H NMR (400 MHz, CDCl3)
f1 (ppm)

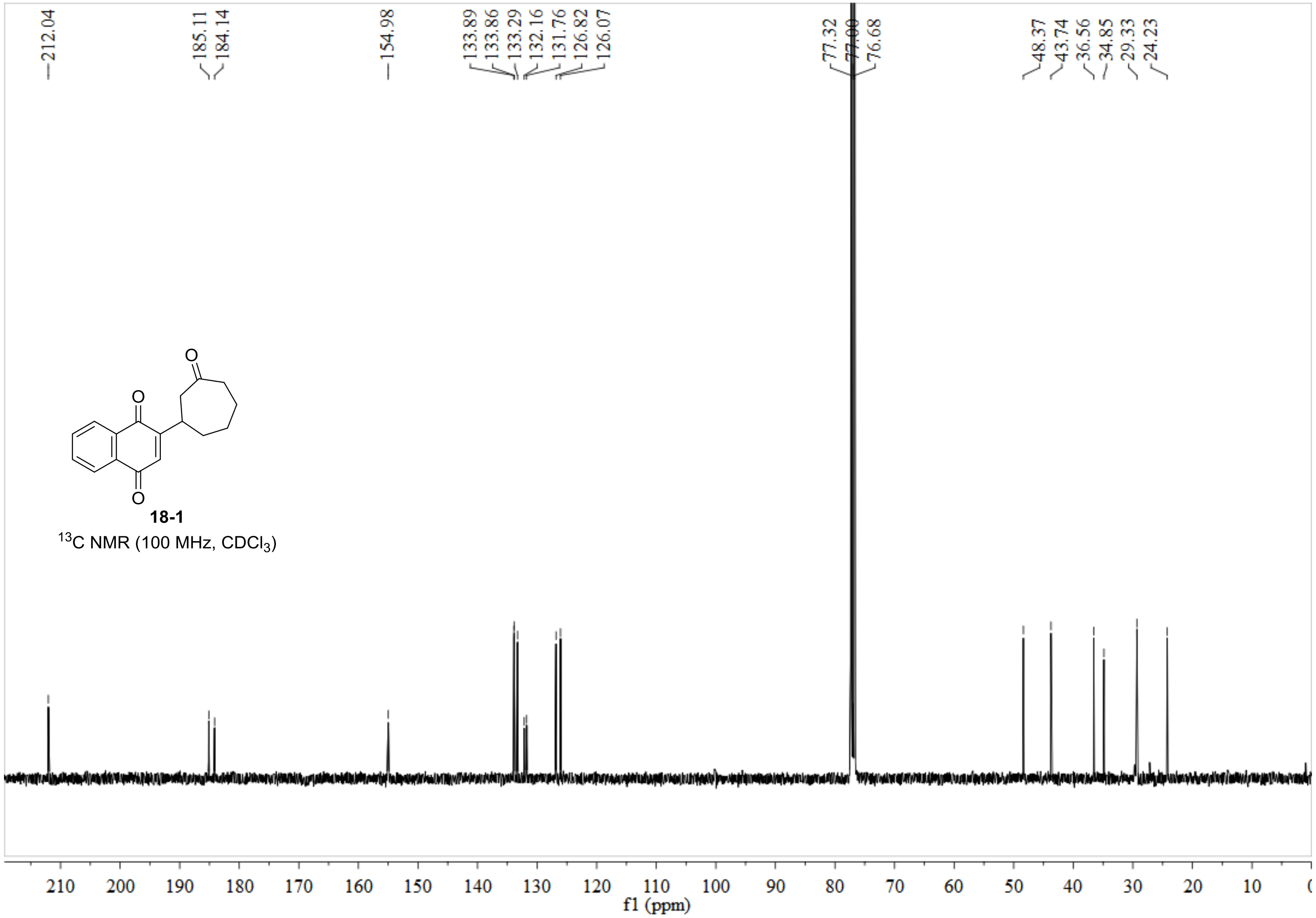

18-1

^{13}C NMR (100 MHz, $CDCl_3$)

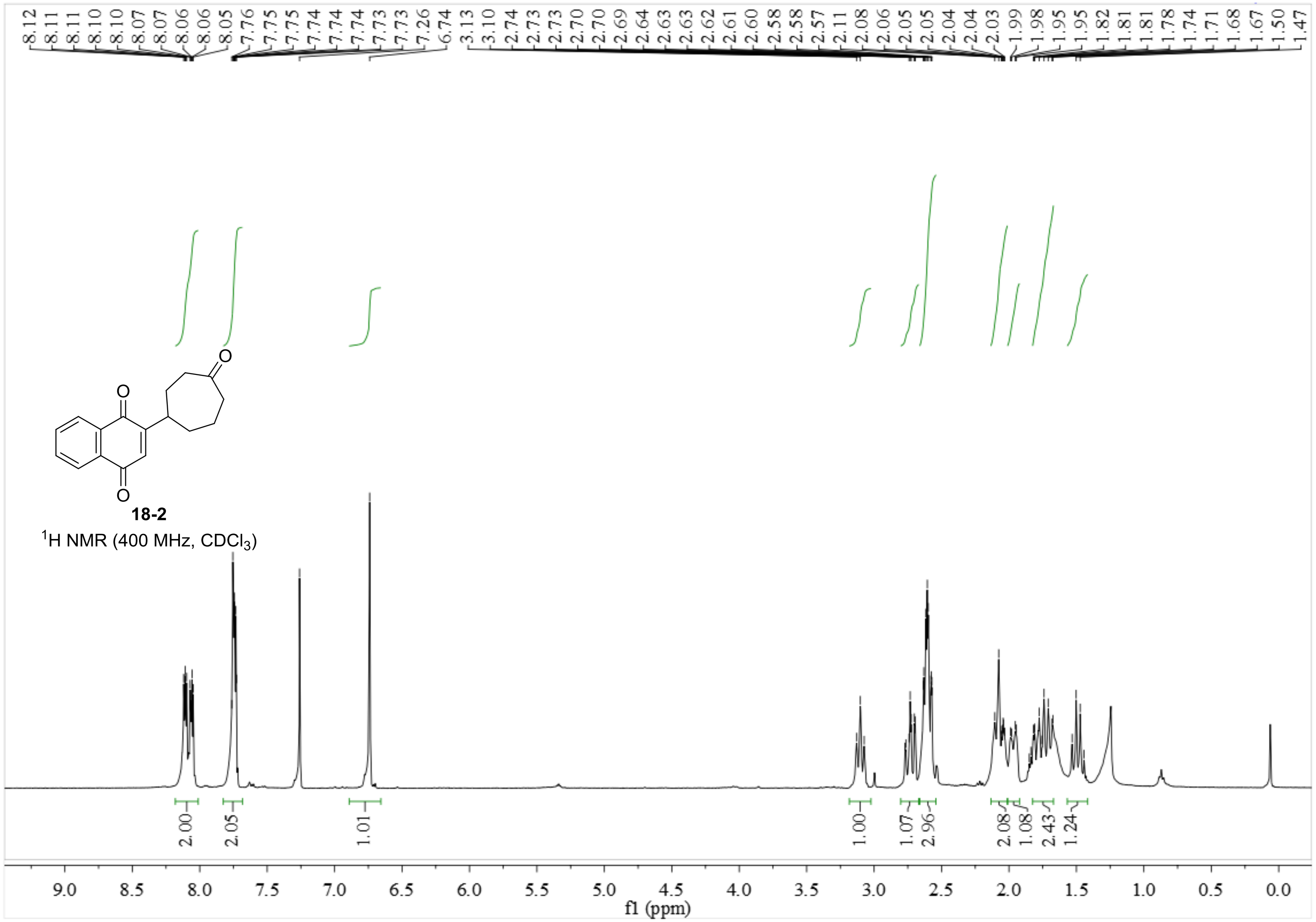

8.12 8.11 8.11 8.10 8.10 8.07 8.07 8.06 8.06 8.05 7.76 7.75 7.75 7.74 7.74 7.74 7.73 7.73 7.26 6.74 3.13 3.10 2.74 2.73 2.73 2.70 2.70 2.69 2.64 2.63 2.63 2.62 2.61 2.60 2.58 2.58 2.57 2.11 2.08 2.06 2.05 2.05 2.04 2.04 2.03 1.99 1.98 1.95 1.95 1.82 1.81 1.81 1.78 1.74 1.71 1.68 1.67 1.50 1.47
18-2
^{1}H NMR (400 MHz, CDCl$_3$)
2.00 2.05 1.01 1.00 1.07 2.96 2.08 1.08 2.43 1.24
9.0 8.5 8.0 7.5 7.0 6.5 6.0 5.5 5.0 4.5 4.0 3.5 3.0 2.5 2.0 1.5 1.0 0.5 0.0
f1 (ppm)

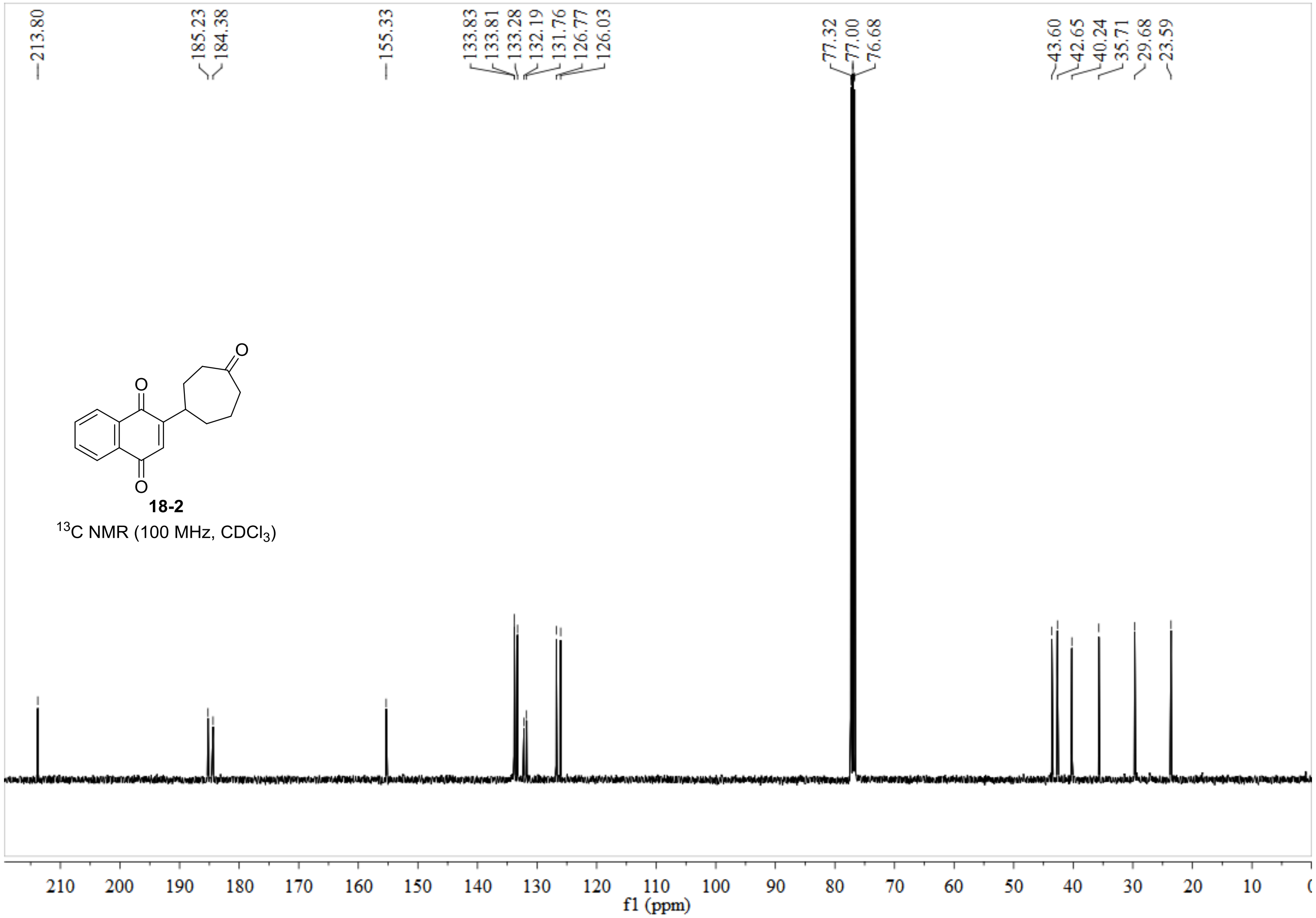
213.80
185.23
184.38
155.33
133.83
133.81
133.28
132.19
131.76
126.77
126.03
77.32
77.00
76.68
43.60
42.65
40.24
35.71
29.68
23.59
18-2
^{13}C NMR (100 MHz, $CDCl_3$)
210 200 190 180 170 160 150 140 130 120 110 100 90 80 70 60 50 40 30 20 10
f1 (ppm)

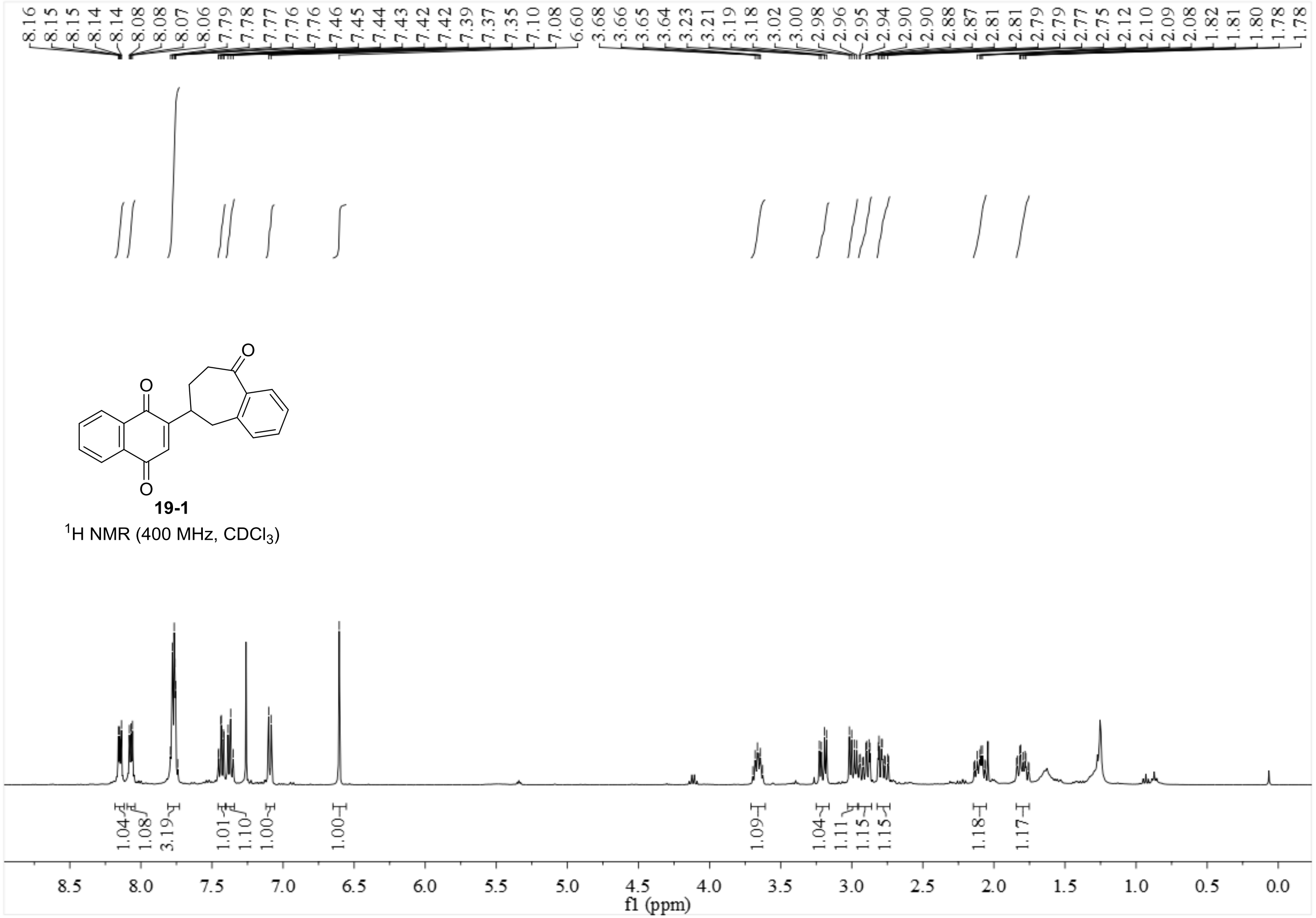

19-1

^{1}H NMR (400 MHz, $CDCl_3$)

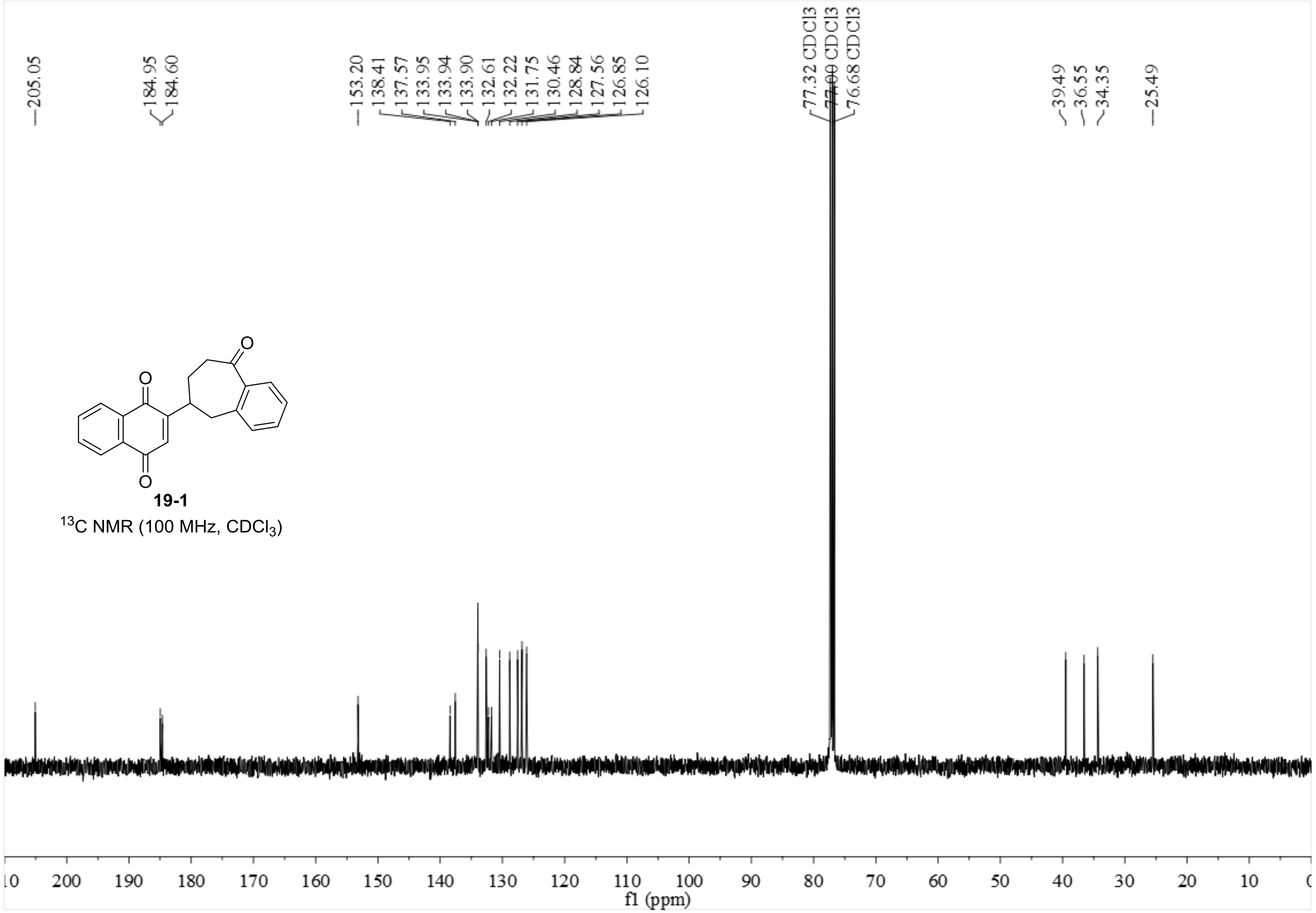
205.05
184.95
184.60
153.20
138.41
137.57
133.95
133.94
133.90
132.61
132.22
131.75
130.46
128.84
127.56
126.85
126.10
77.32 CDCl3
77.00 CDCl3
76.68 CDCl3
39.49
36.55
34.35
25.49
19-1
^{13}C NMR (100 MHz, $CDCl_3$)
10 200 190 180 170 160 150 140 130 120 110 100 90 80 70 60 50 40 30 20 10 0
f1 (ppm)

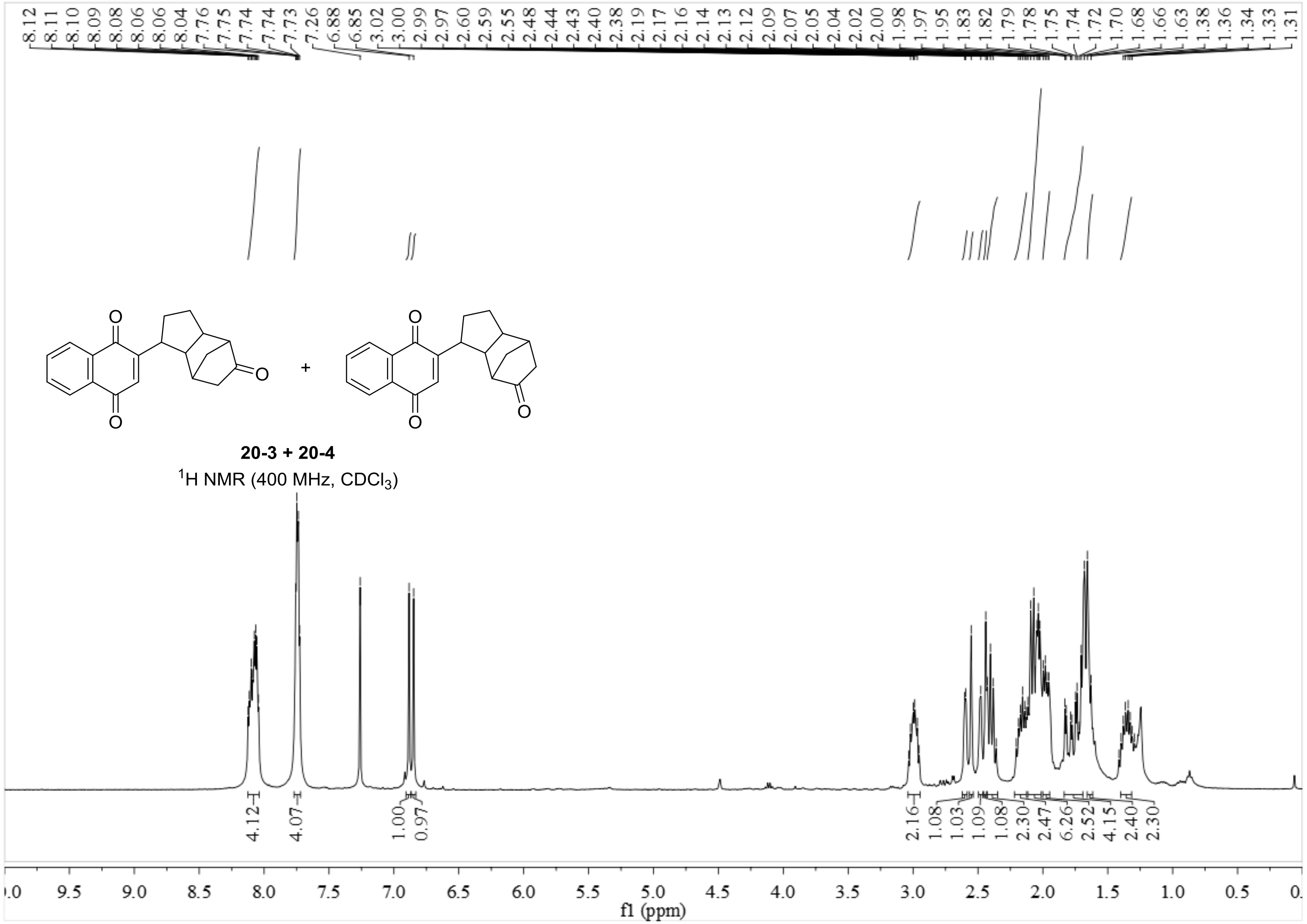

20-3 + 20-4
¹H NMR (400 MHz, CDCl3)
f1 (ppm)

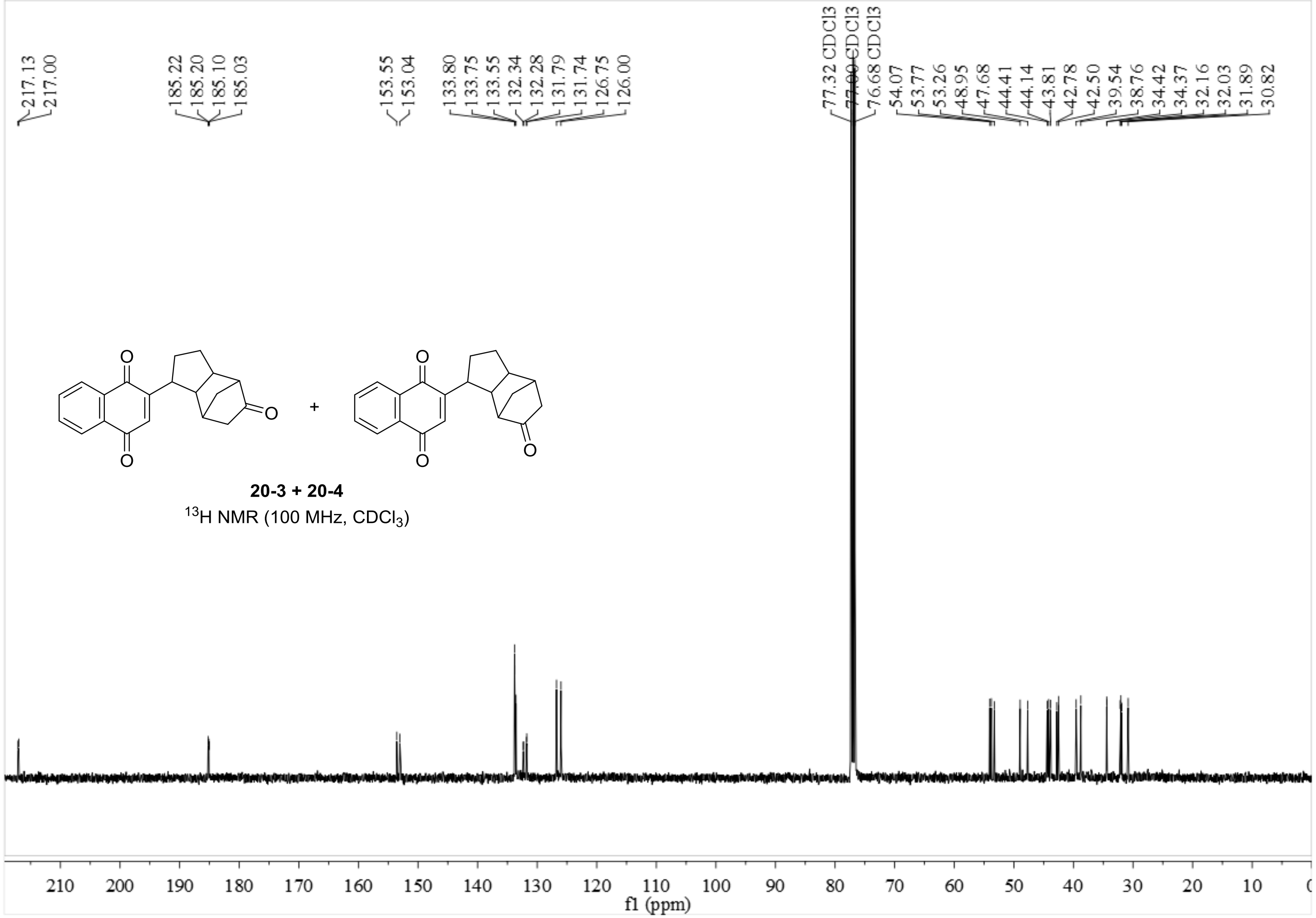

20-3 + 20-4

^{13}H NMR (100 MHz, $CDCl_3$)

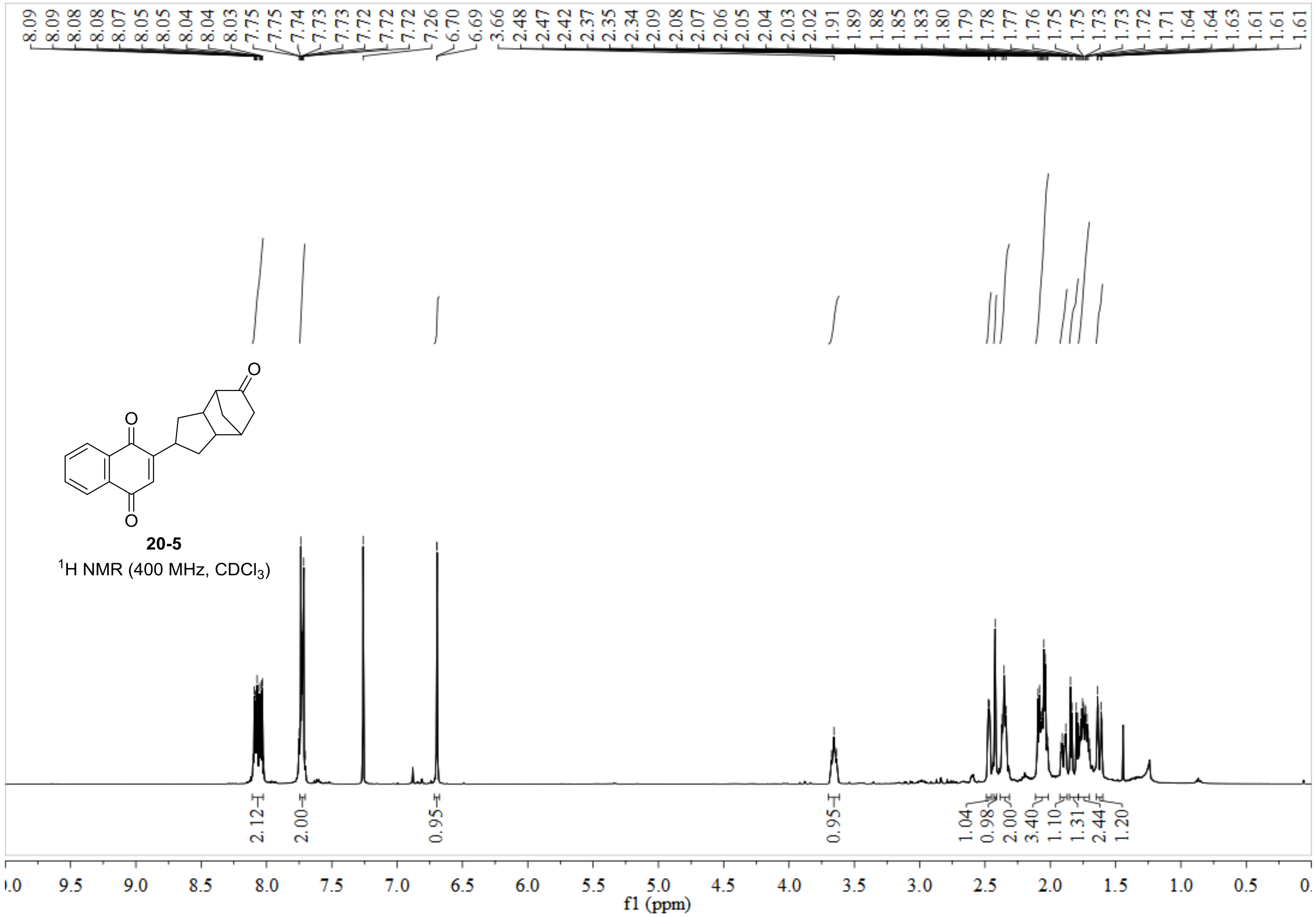

20-5
1H NMR (400 MHz, CDCl3)
8.09 8.09 8.08 8.08 8.07 8.05 8.05 8.04 8.04 8.03 7.75 7.75 7.74 7.73 7.73 7.72 7.72 7.72 7.26 6.70 6.69 3.66 2.48 2.47 2.42 2.37 2.35 2.34 2.09 2.08 2.07 2.06 2.05 2.04 2.03 2.02 1.91 1.89 1.88 1.85 1.83 1.80 1.79 1.78 1.77 1.76 1.75 1.75 1.73 1.73 1.72 1.71 1.64 1.64 1.63 1.61 1.61 1.61
2.12 2.00 0.95 0.95 1.04 0.98 2.00 3.40 1.10 1.31 2.44 1.20
9.5 9.0 8.5 8.0 7.5 7.0 6.5 6.0 5.5 5.0 4.5 4.0 3.5 3.0 2.5 2.0 1.5 1.0 0.5
f1 (ppm)

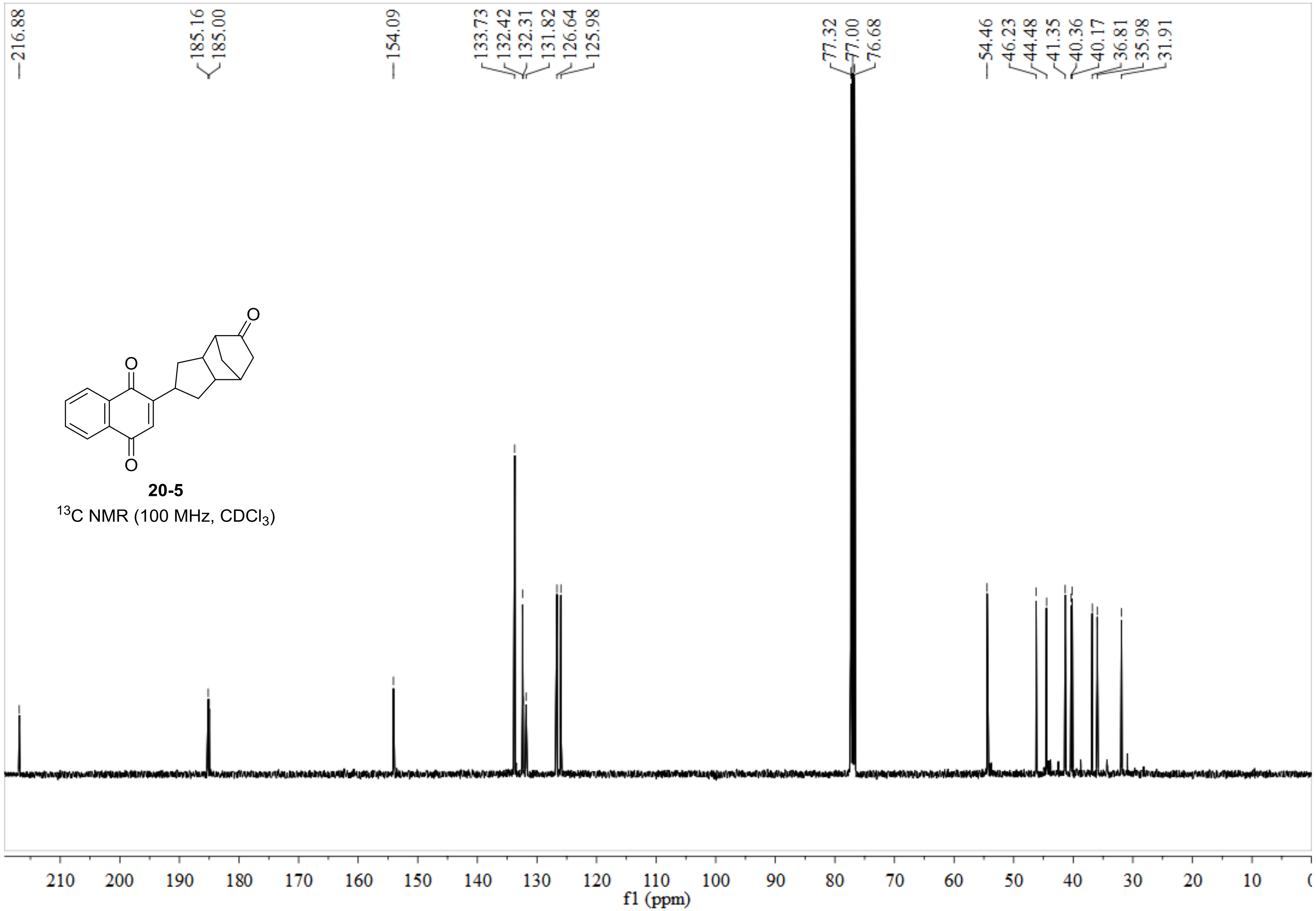

20-5

^{13}C NMR (100 MHz, $CDCl_3$)

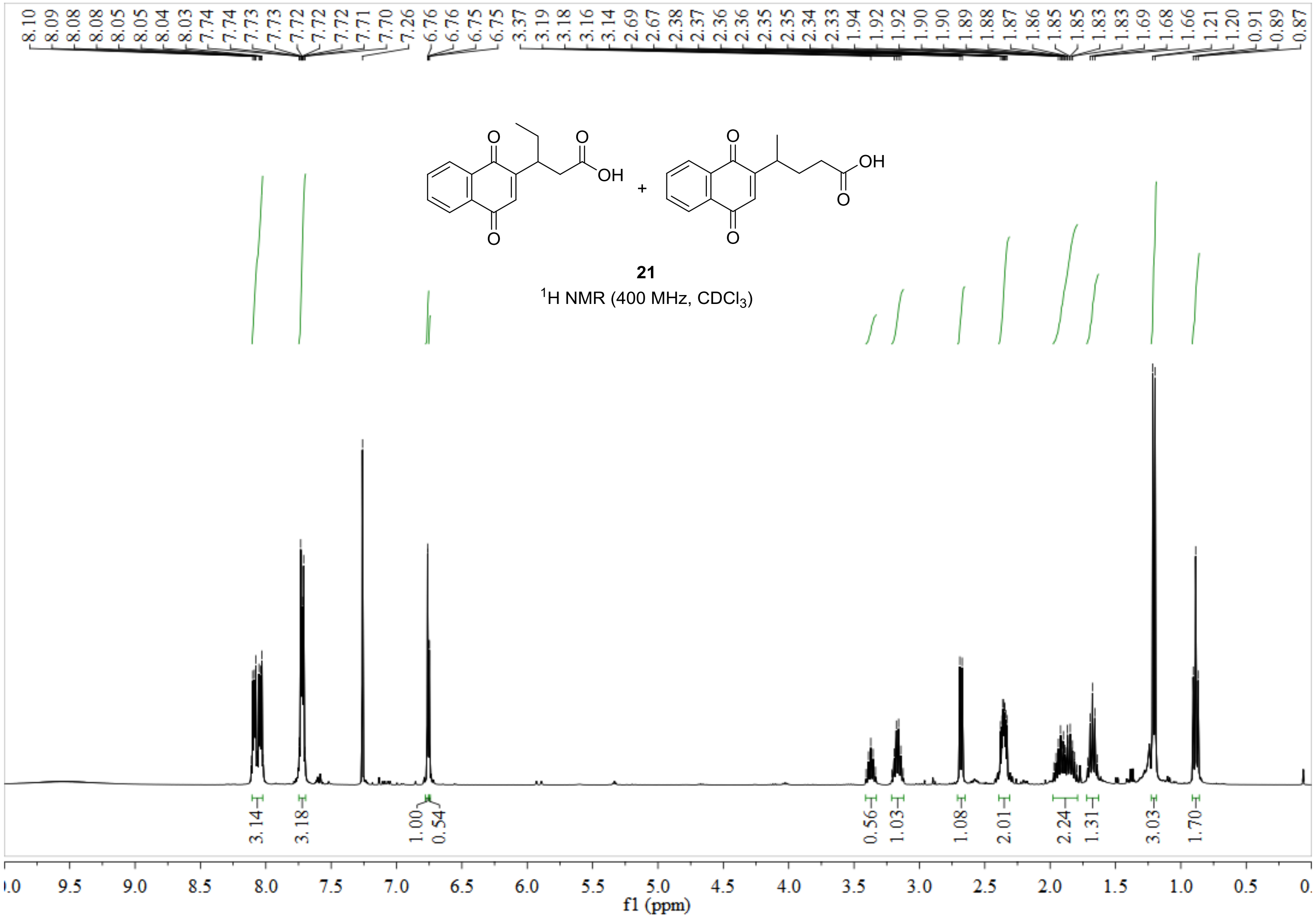

21
1H NMR (400 MHz, CDCl3)
f1 (ppm)

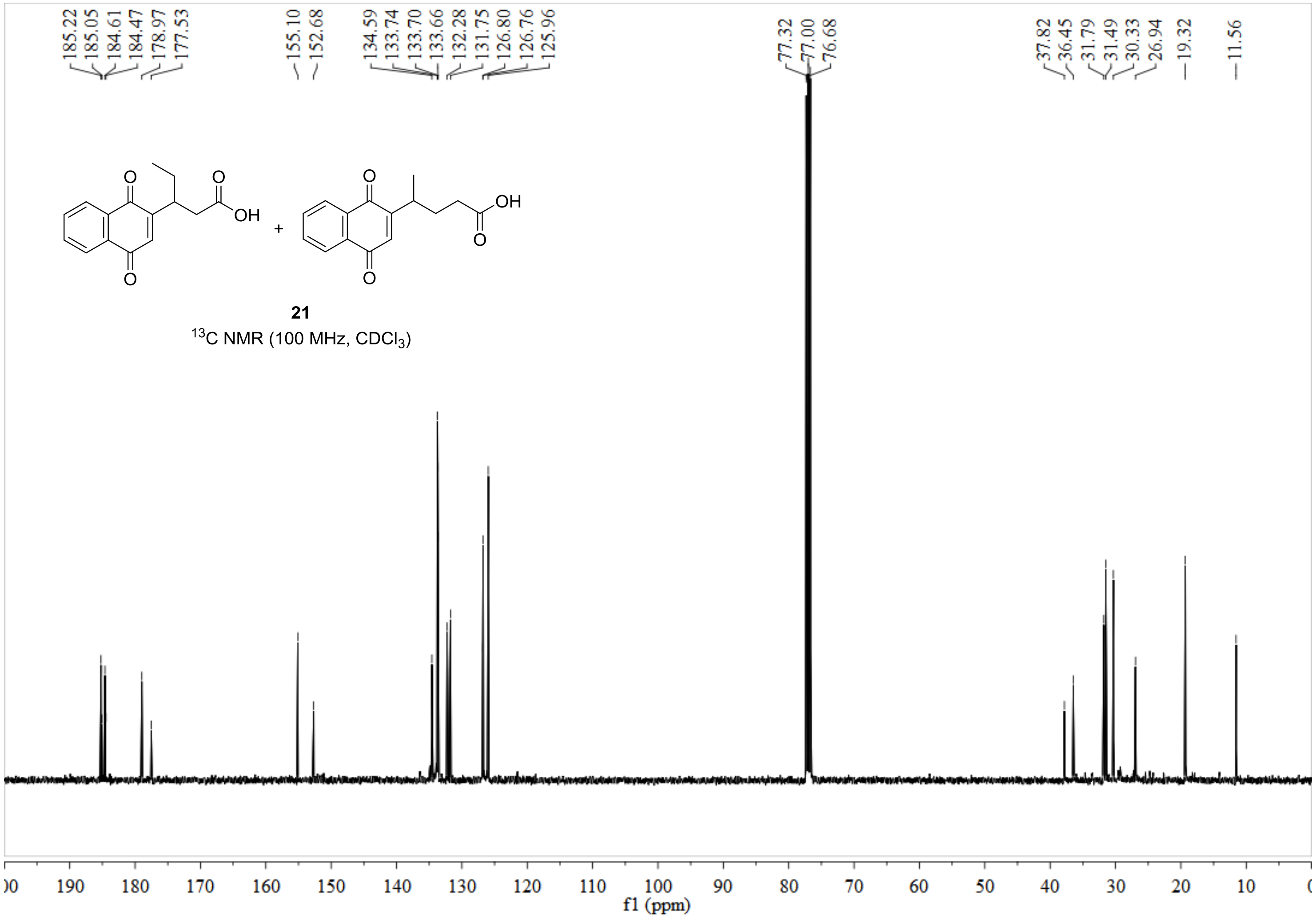

185.22
185.05
184.61
184.47
178.97
177.53
155.10
152.68
134.59
133.74
133.70
133.66
132.28
131.75
126.80
126.76
125.96
77.32
77.00
76.68
37.82
36.45
31.79
31.49
30.33
26.94
19.32
11.56
O
OH
+
21
^{13}C NMR (100 MHz, CDCl$_3$)
f1 (ppm)

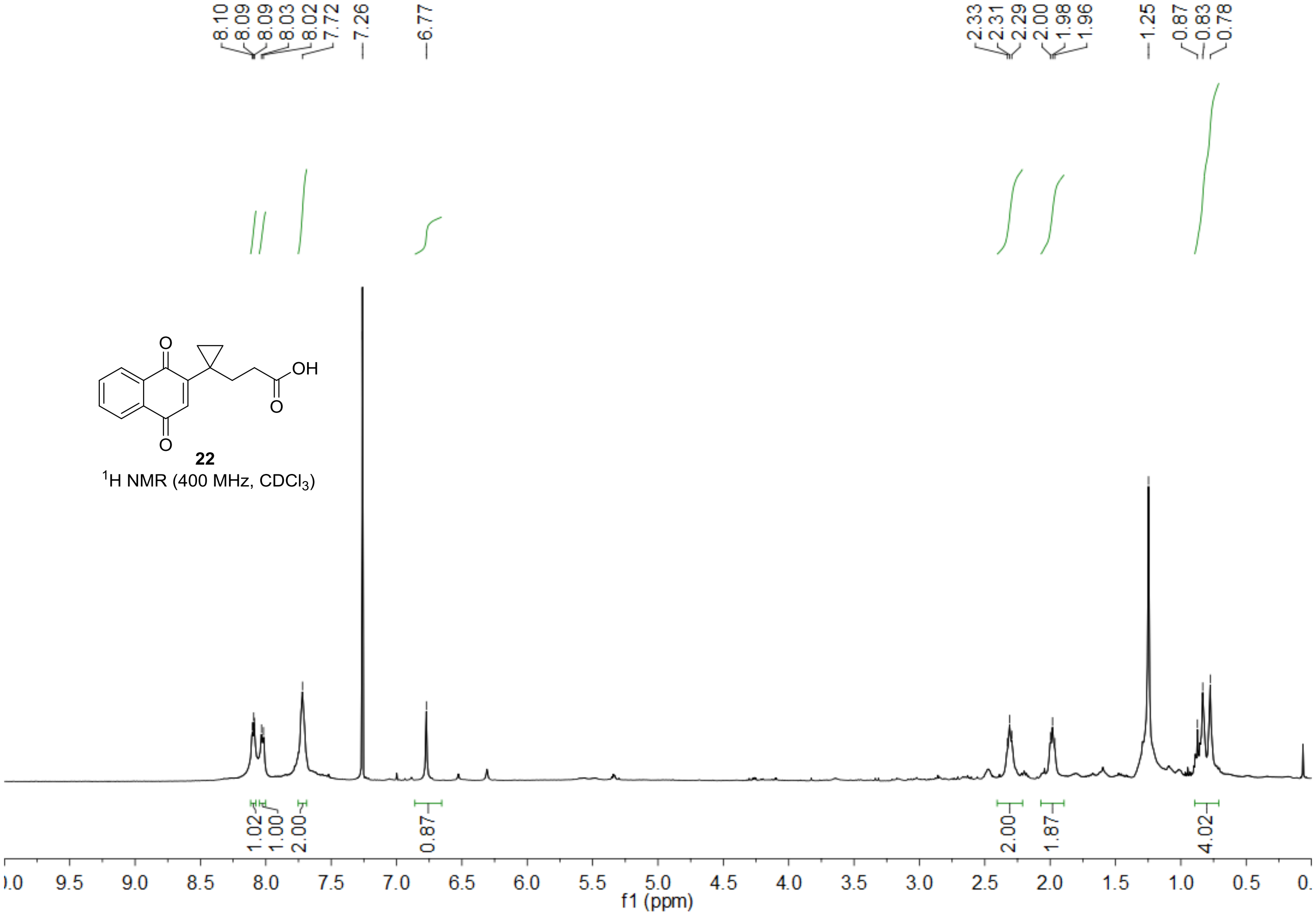
8.10
8.09
8.09
8.03
8.02
7.72
7.26
6.77
2.33
2.31
2.29
2.00
1.98
1.96
1.25
0.87
0.83
0.78
O
O
OH
O
22
¹H NMR (400 MHz, CDCl₃)
1.02
1.00
2.00
0.87
2.00
1.87
4.02
9.5
9.0
8.5
8.0
7.5
7.0
6.5
6.0
5.5
5.0
4.5
4.0
3.5
3.0
2.5
2.0
1.5
1.0
0.5
f1 (ppm)

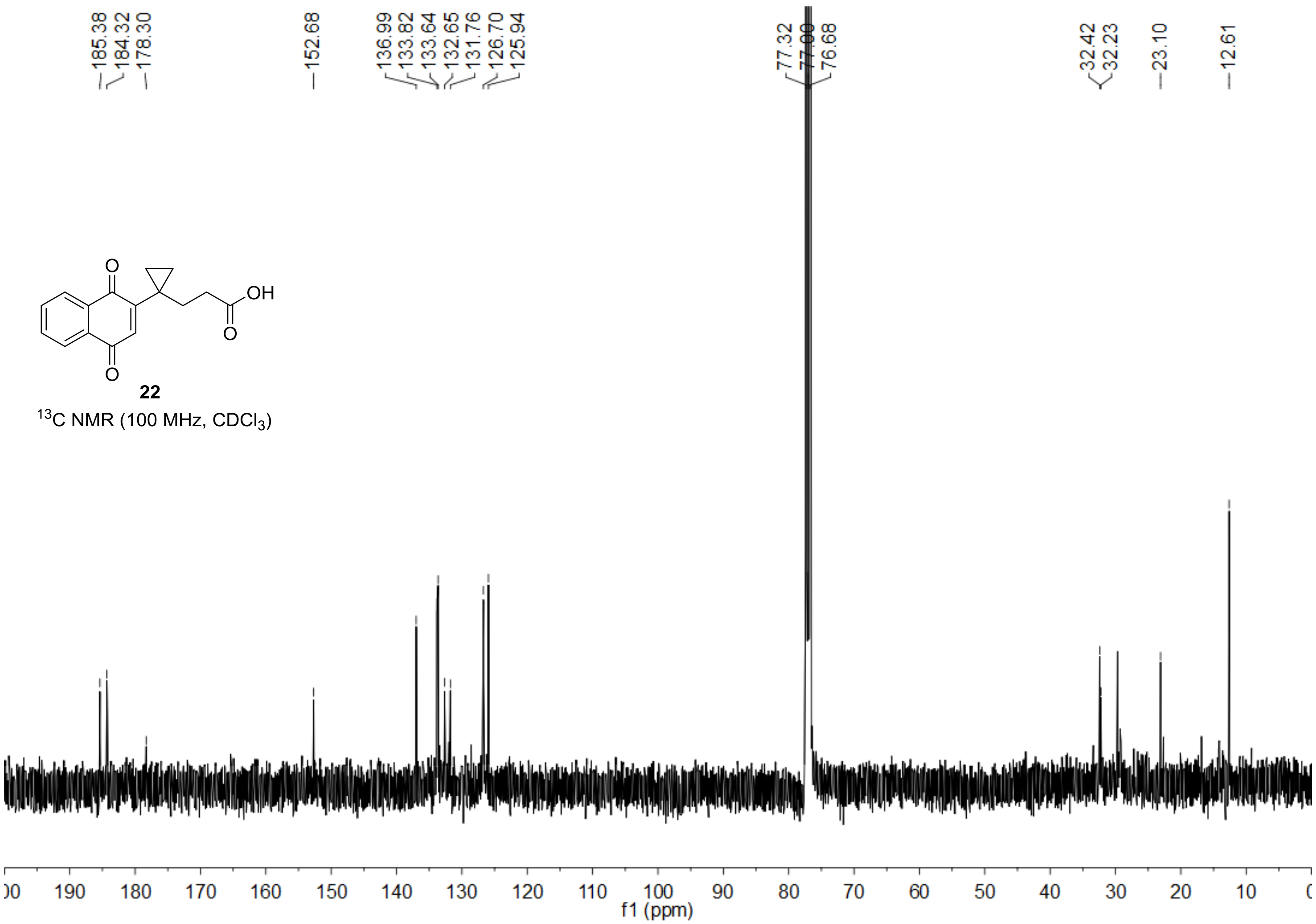

22

^{13}C NMR (100 MHz, $CDCl_3$)

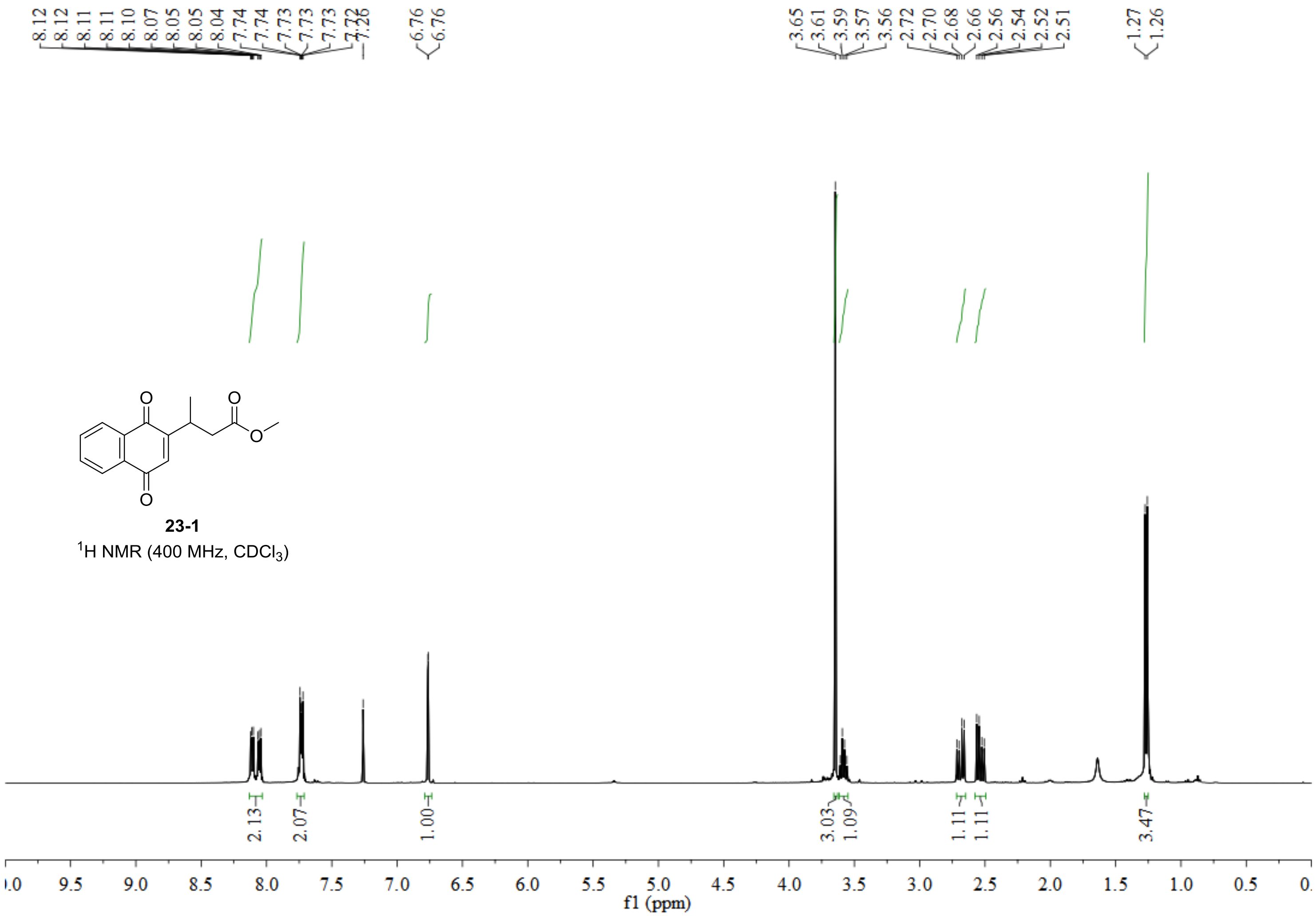
8.12
8.12
8.11
8.11
8.10
8.07
8.05
8.05
8.04
7.74
7.74
7.73
7.73
7.73
7.72
7.26
6.76
6.76
3.65
3.61
3.59
3.57
3.56
2.72
2.70
2.68
2.66
2.56
2.54
2.52
2.51
1.27
1.26
23-1
1H NMR (400 MHz, CDCl3)
2.13
2.07
1.00
3.03
1.09
1.11
1.11
3.47
9.5
9.0
8.5
8.0
7.5
7.0
6.5
6.0
5.5
5.0
4.5
4.0
3.5
3.0
2.5
2.0
1.5
1.0
0.5
f1 (ppm)

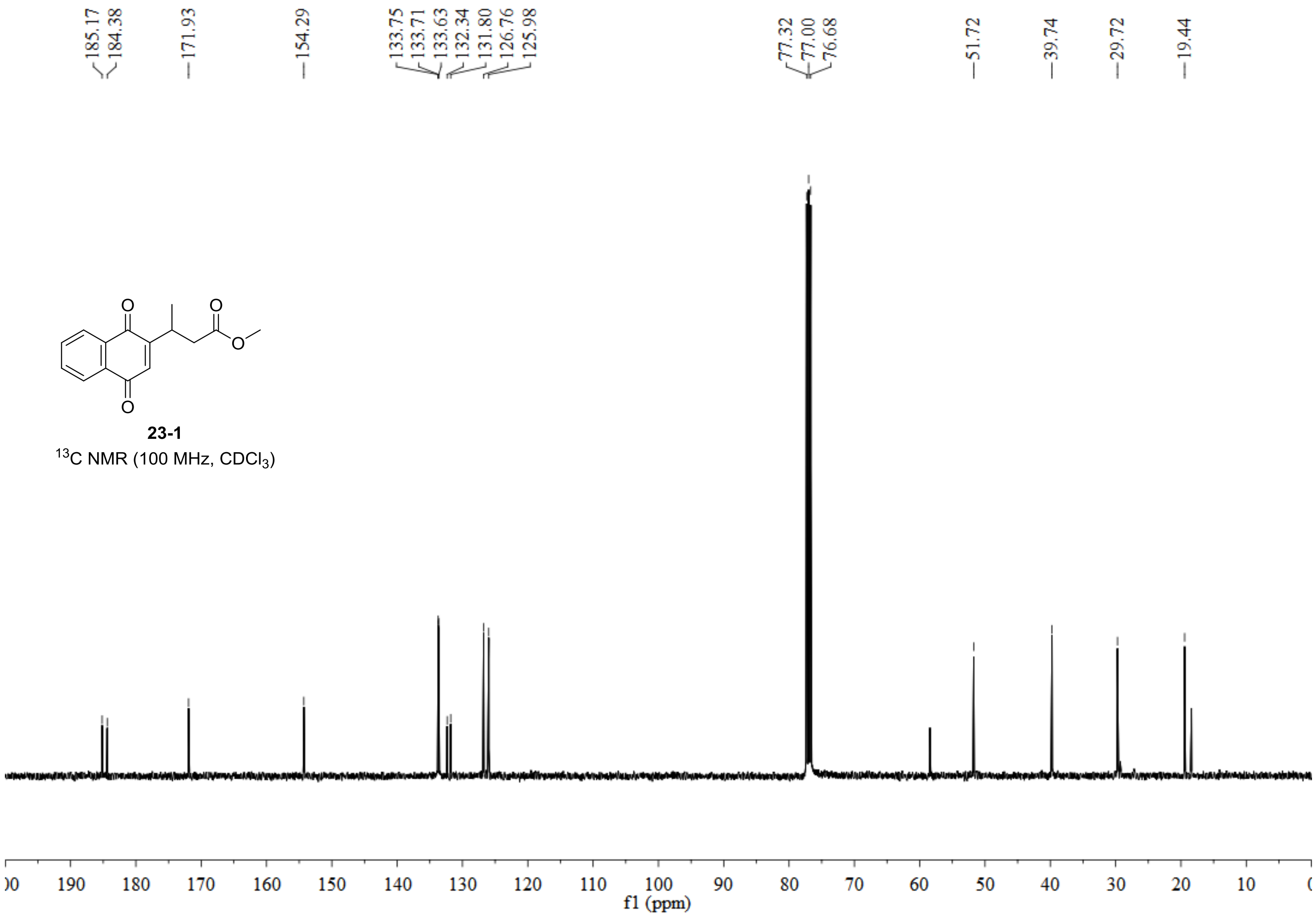

23-1

^{13}C NMR (100 MHz, $CDCl_3$)

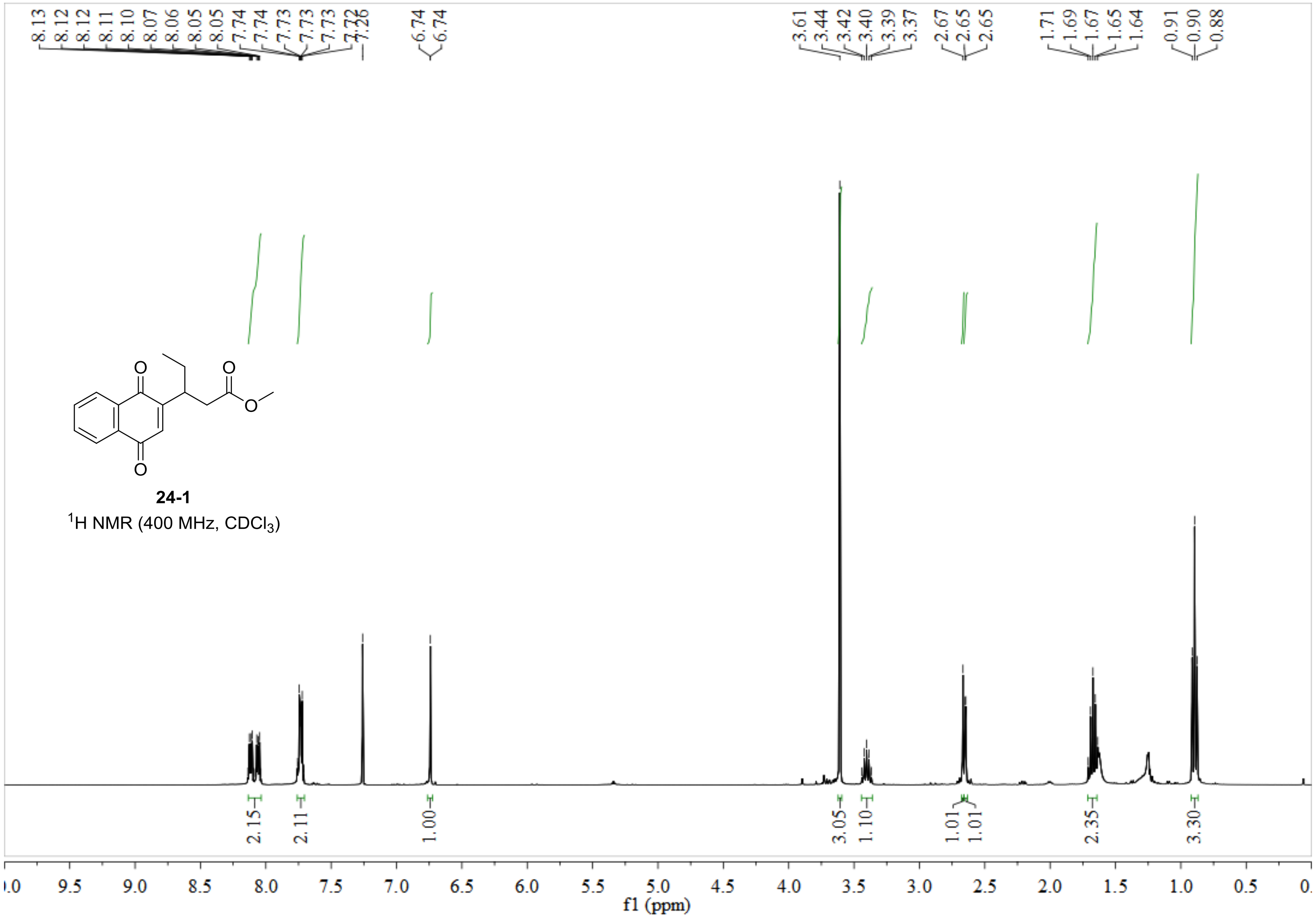

8.13
8.12
8.12
8.11
8.10
8.07
8.06
8.05
8.05
7.74
7.74
7.73
7.73
7.73
7.72
7.26
6.74
6.74
3.61
3.44
3.42
3.40
3.39
3.37
2.67
2.65
2.65
1.71
1.69
1.67
1.65
1.64
0.91
0.90
0.88
24-1
1H NMR (400 MHz, CDCl3)
2.15
2.11
1.00
3.05
1.10
1.01
1.01
2.35
3.30
9.5
9.0
8.5
8.0
7.5
7.0
6.5
6.0
5.5
5.0
4.5
4.0
3.5
3.0
2.5
2.0
1.5
1.0
0.5
f1 (ppm)

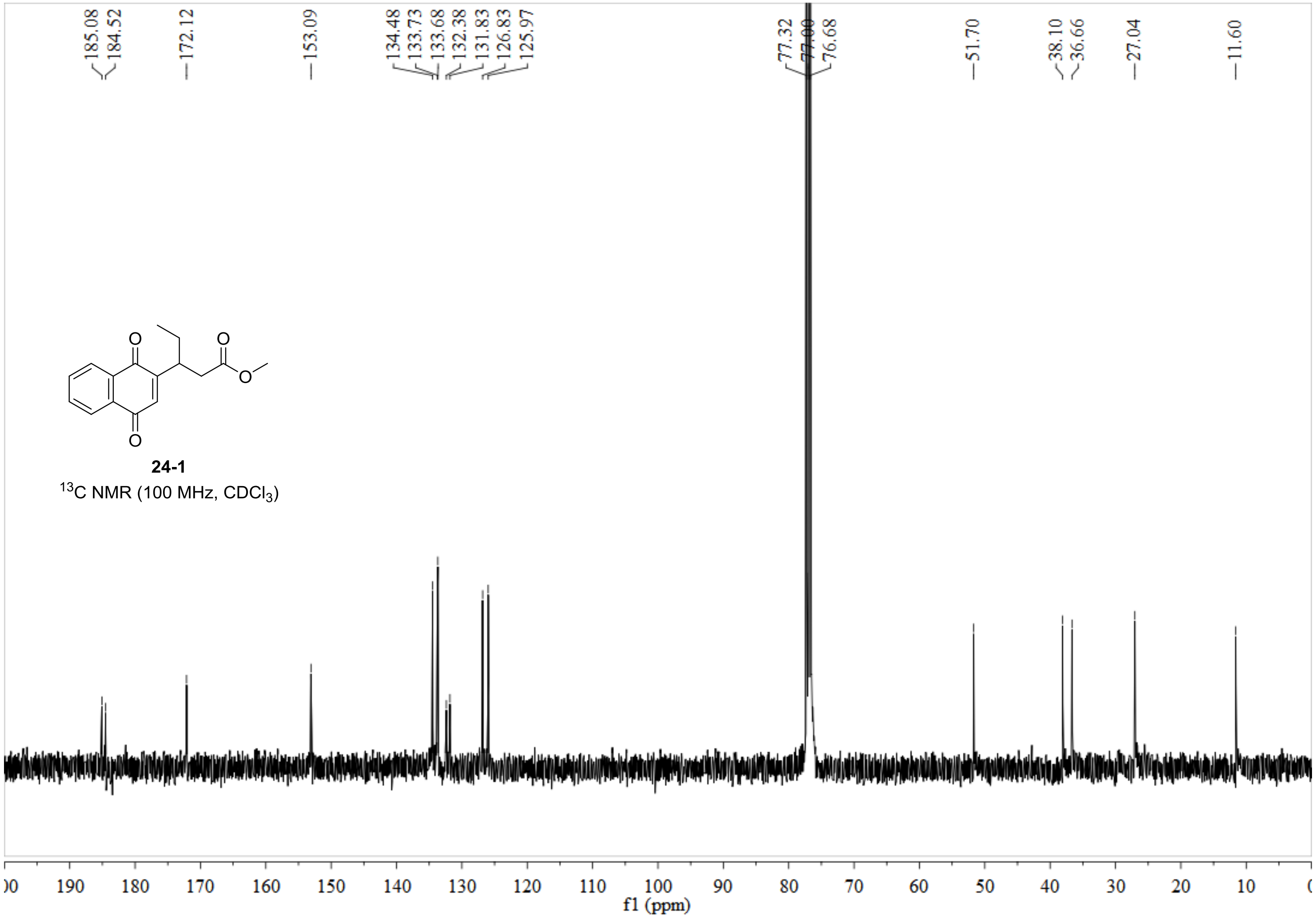
185.08
184.52
172.12
153.09
134.48
133.73
133.68
132.38
131.83
126.83
125.97
77.32
77.00
76.68
51.70
38.10
36.66
27.04
11.60
O
O
O
O
24-1
^{13}C NMR (100 MHz, $CDCl_3$)
190
180
170
160
150
140
130
120
110
100
90
80
70
60
50
40
30
20
10
f1 (ppm)

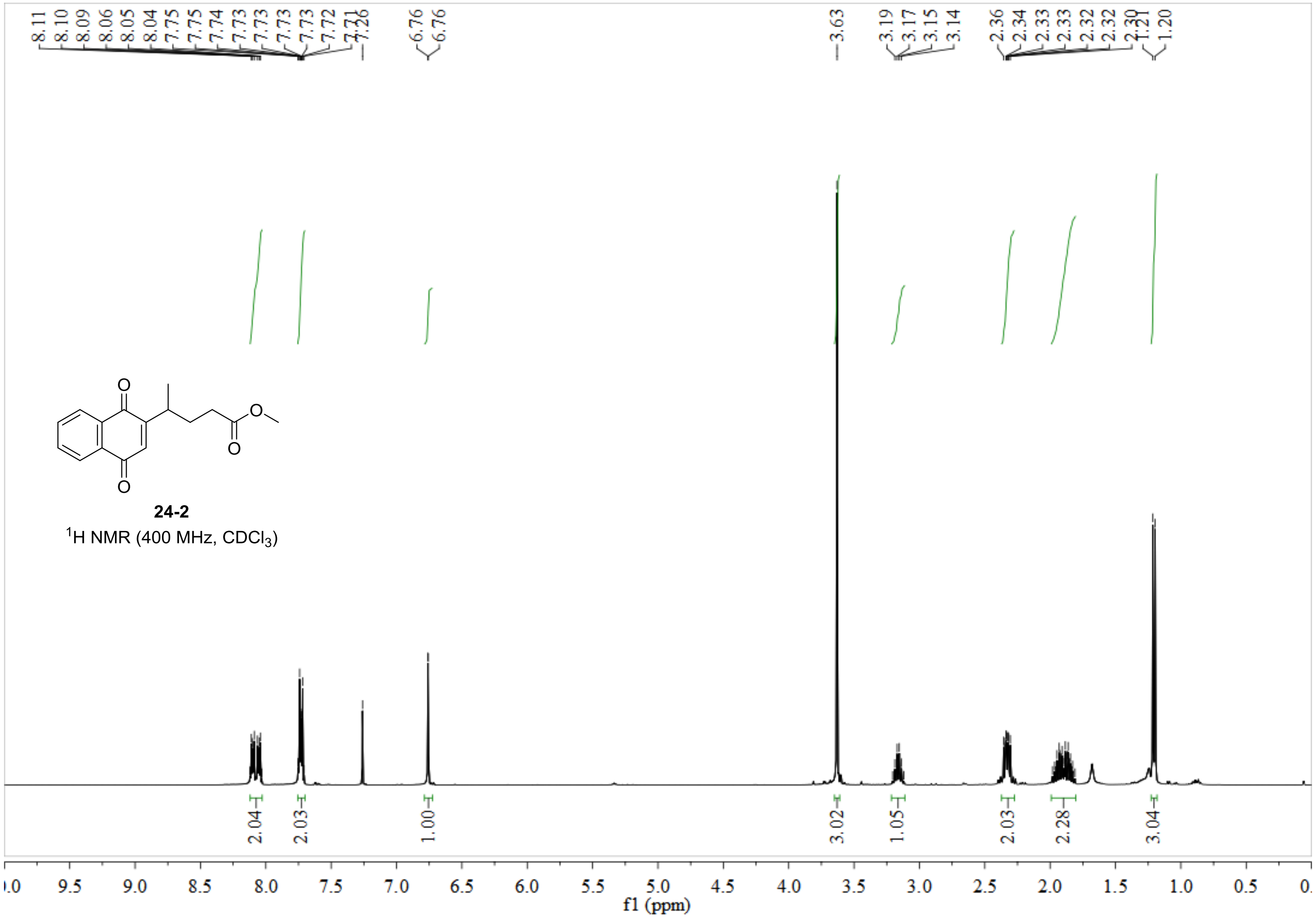

8.11
8.10
8.09
8.06
8.05
8.04
7.75
7.75
7.74
7.73
7.73
7.73
7.73
7.72
7.71
7.26
6.76
6.76
3.63
3.19
3.17
3.15
3.14
2.36
2.34
2.33
2.33
2.32
2.32
2.30
1.21
1.20
24-2
^{1}H NMR (400 MHz, $CDCl_3$)
2.04
2.03
1.00
3.02
1.05
2.03
2.28
3.04
f1 (ppm)

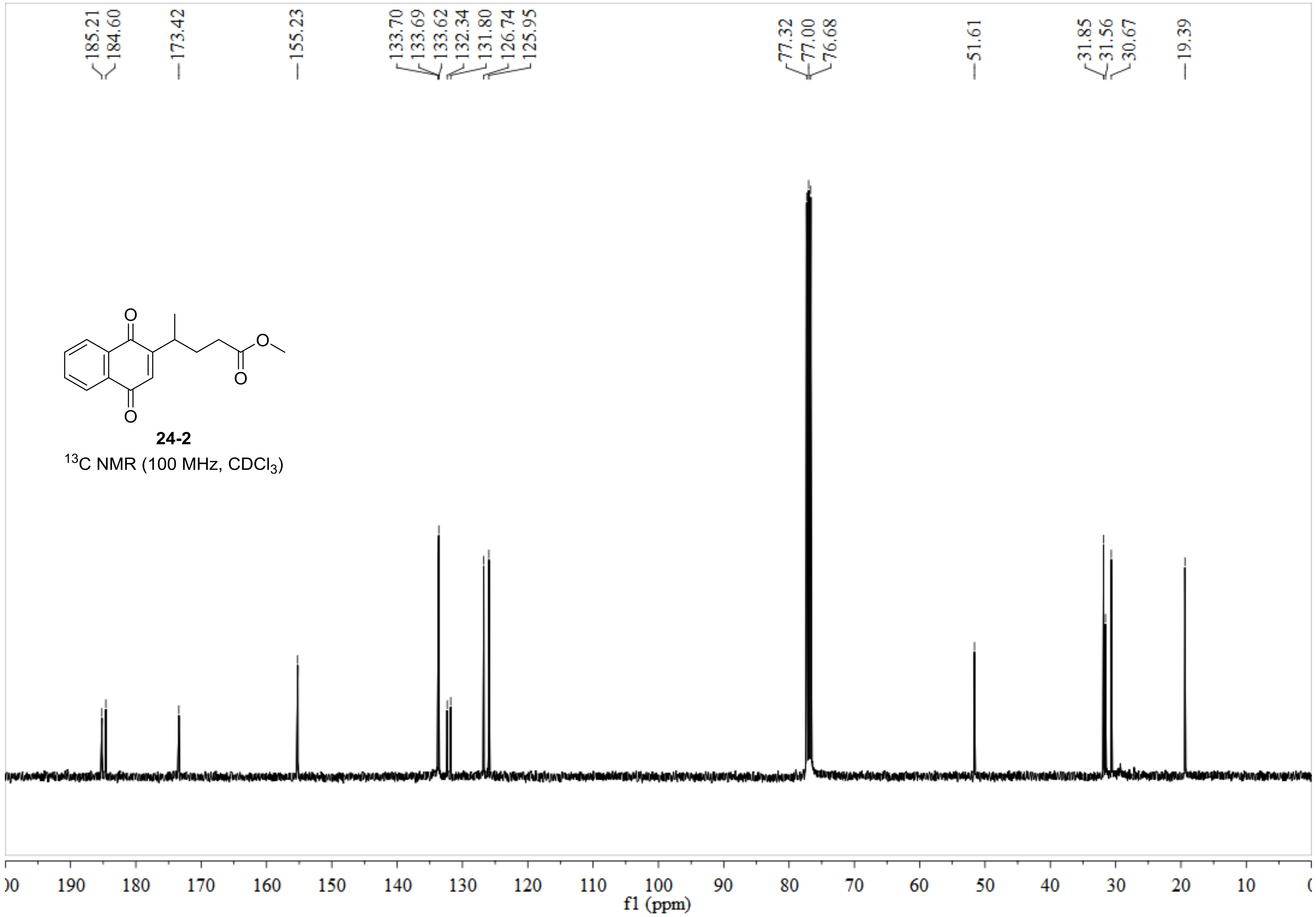
185.21
184.60
173.42
155.23
133.70
133.69
133.62
132.34
131.80
126.74
125.95
77.32
77.00
76.68
51.61
31.85
31.56
30.67
19.39
24-2
13C NMR (100 MHz, CDCl3)
f1 (ppm)

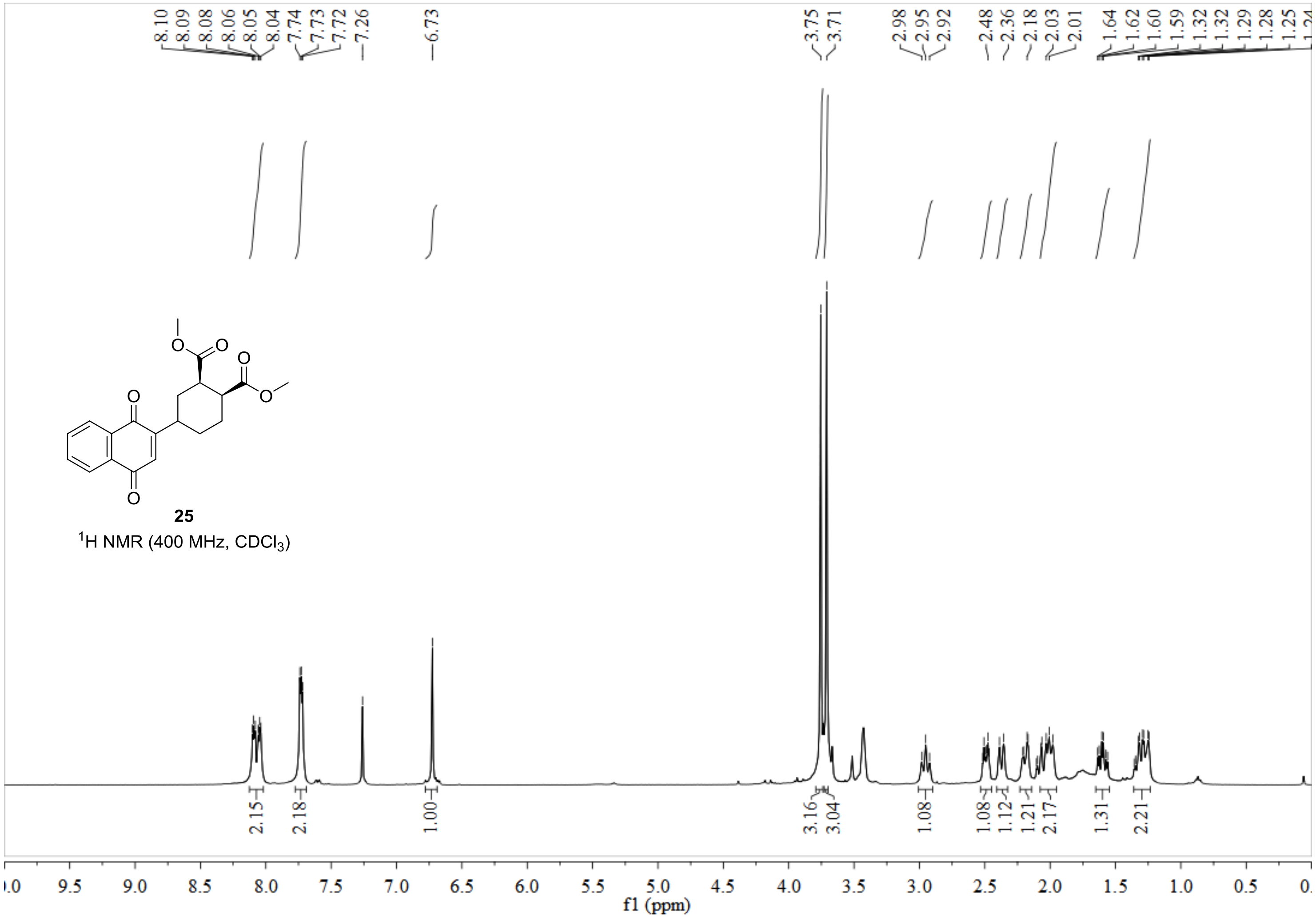

25

^{1}H NMR (400 MHz, $CDCl_3$)

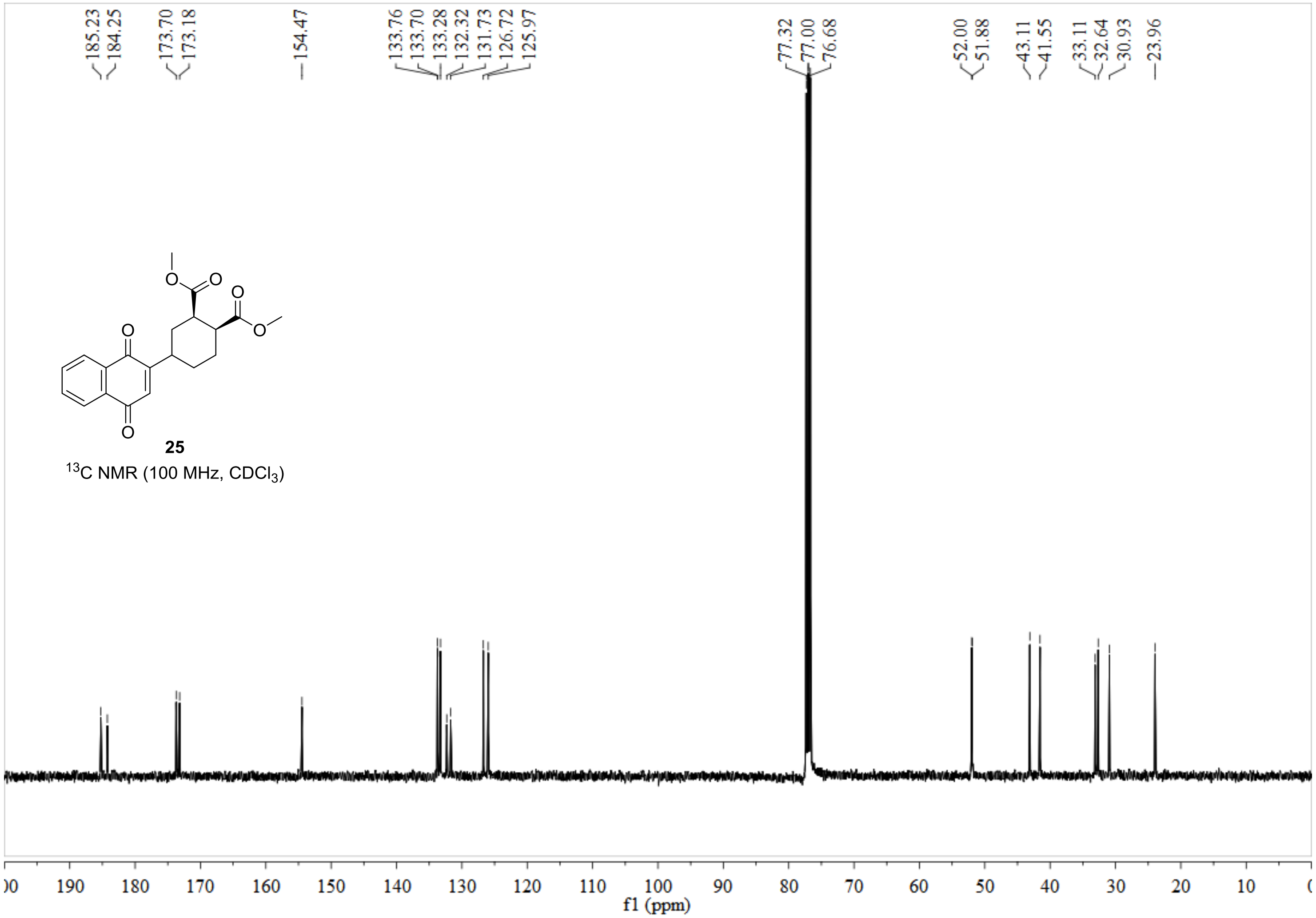
185.23
184.25
173.70
173.18
154.47
133.76
133.70
133.28
132.32
131.73
126.72
125.97
77.32
77.00
76.68
52.00
51.88
43.11
41.55
33.11
32.64
30.93
23.96
25
^{13}C NMR (100 MHz, $CDCl_3$)
f1 (ppm)

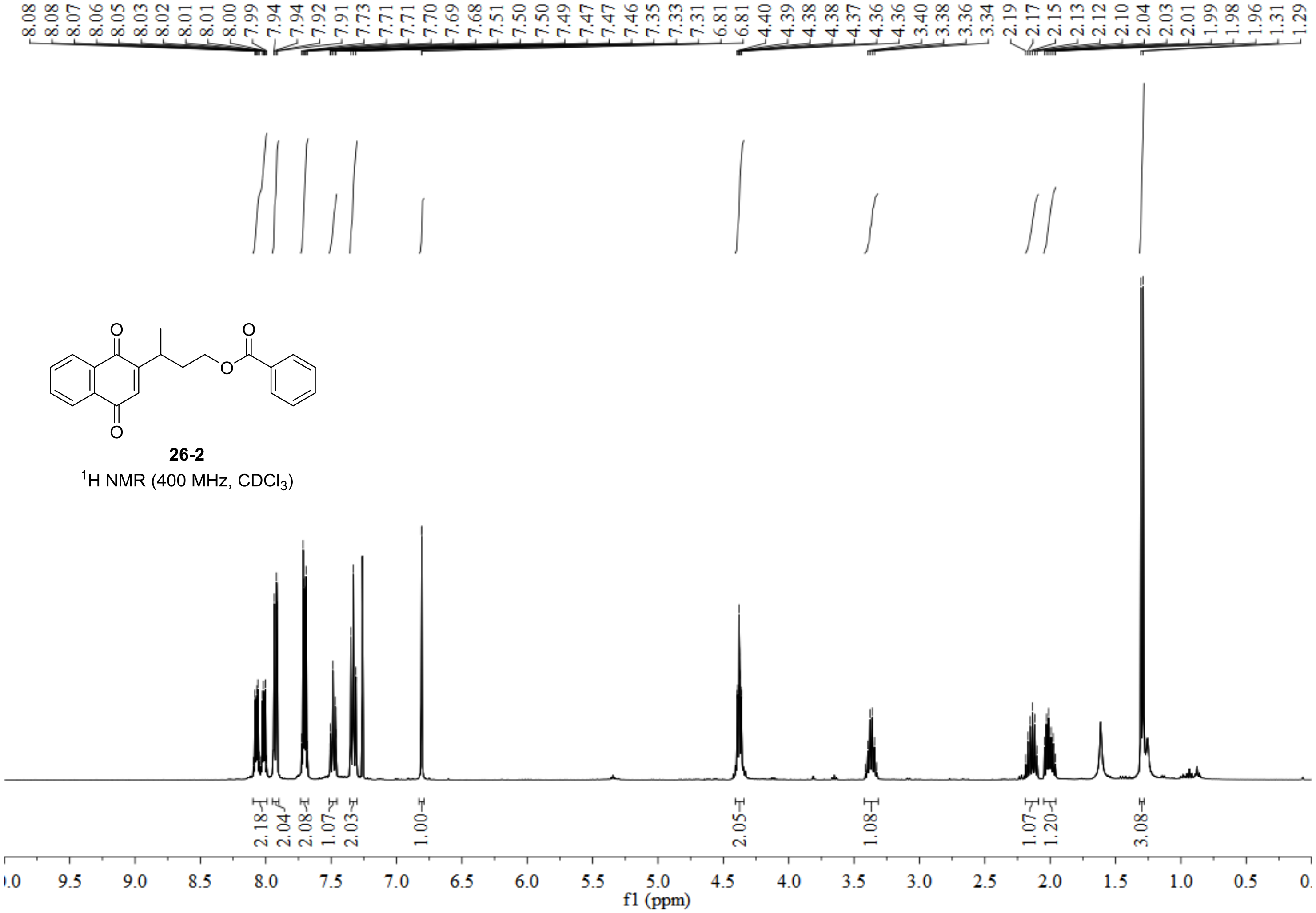

8.08
8.08
8.07
8.06
8.05
8.03
8.02
8.01
8.01
8.00
7.99
7.94
7.94
7.92
7.91
7.73
7.71
7.71
7.70
7.69
7.68
7.51
7.50
7.49
7.47
7.47
7.46
7.35
7.33
7.31
6.81
6.81
4.40
4.39
4.38
4.38
4.37
4.36
4.36
3.40
3.38
3.36
3.34
2.19
2.17
2.15
2.13
2.12
2.10
2.04
2.03
2.01
1.99
1.98
1.96
1.31
1.29
26-2
1H NMR (400 MHz, CDCl3)
2.18
2.04
2.08
1.07
2.03
1.00
2.05
1.08
1.07
1.20
3.08
9.5
9.0
8.5
8.0
7.5
7.0
6.5
6.0
5.5
5.0
4.5
4.0
3.5
3.0
2.5
2.0
1.5
1.0
0.5
f1 (ppm)

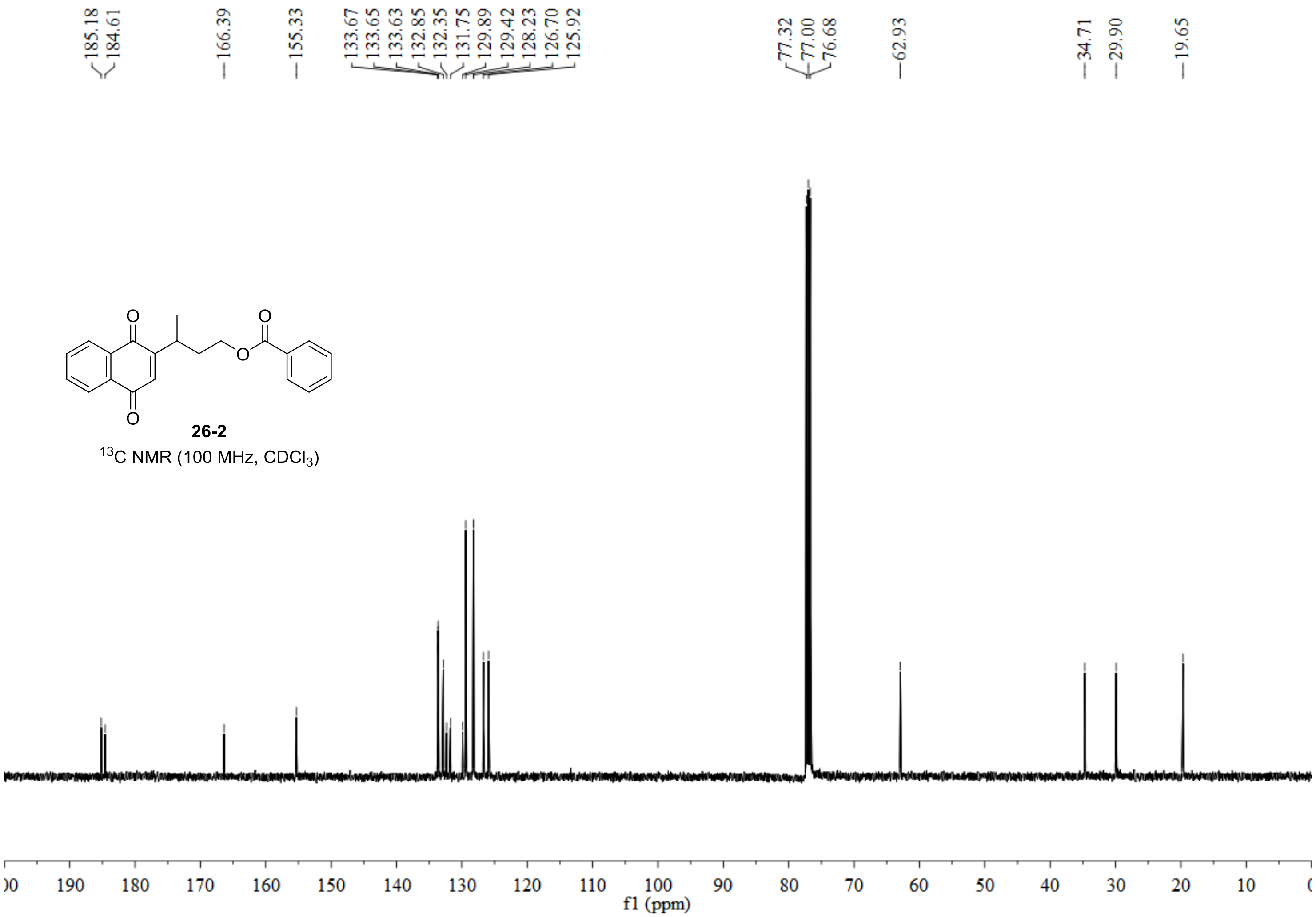
185.18
184.61
166.39
155.33
133.67
133.65
133.63
132.85
132.35
131.75
129.89
129.42
128.23
126.70
125.92
77.32
77.00
76.68
62.93
34.71
29.90
19.65
26-2
^{13}C NMR (100 MHz, $CDCl_3$)
f1 (ppm)

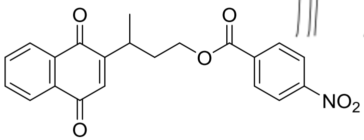

27-2

^1H NMR (400 MHz, $CDCl_3$)

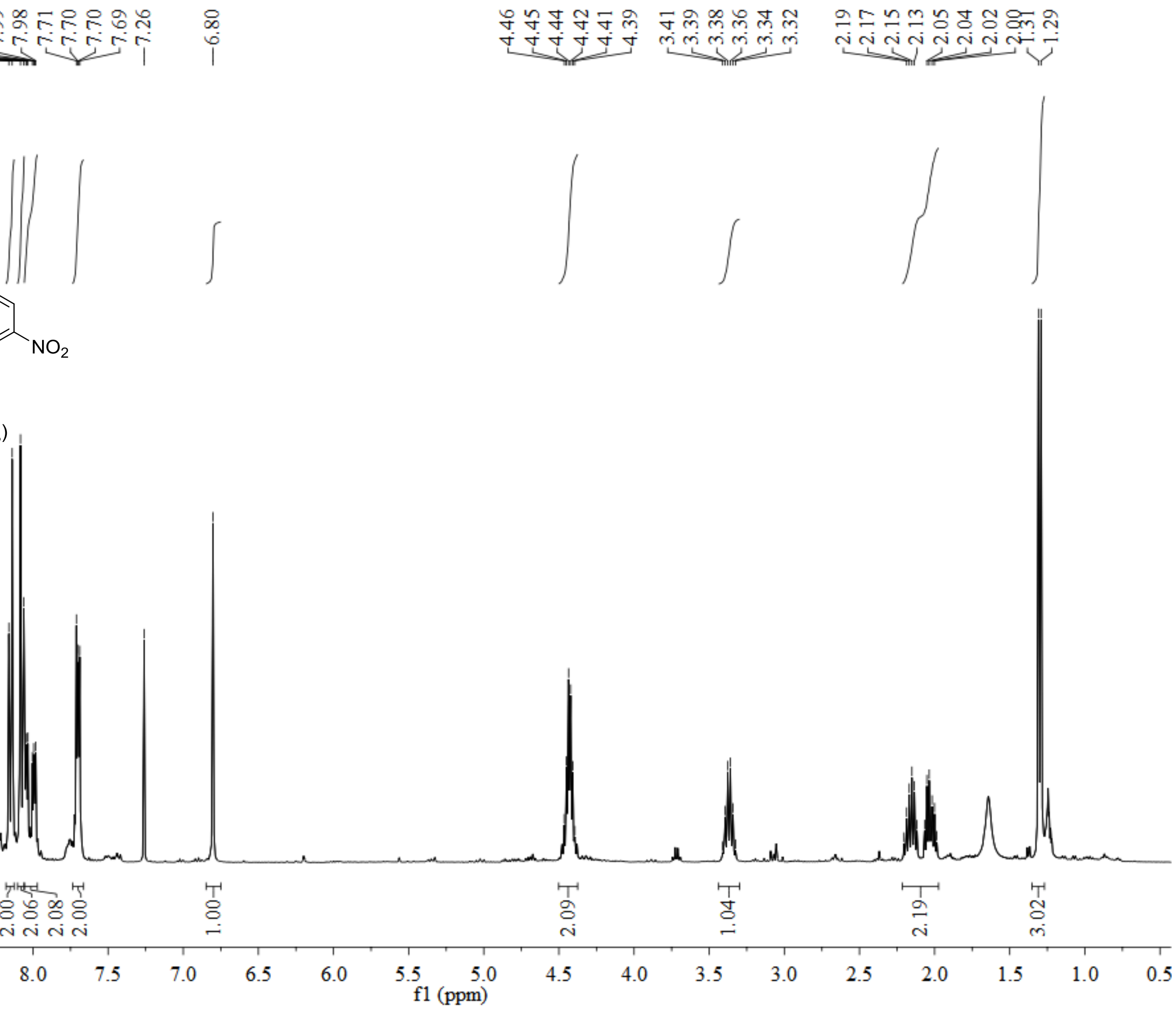

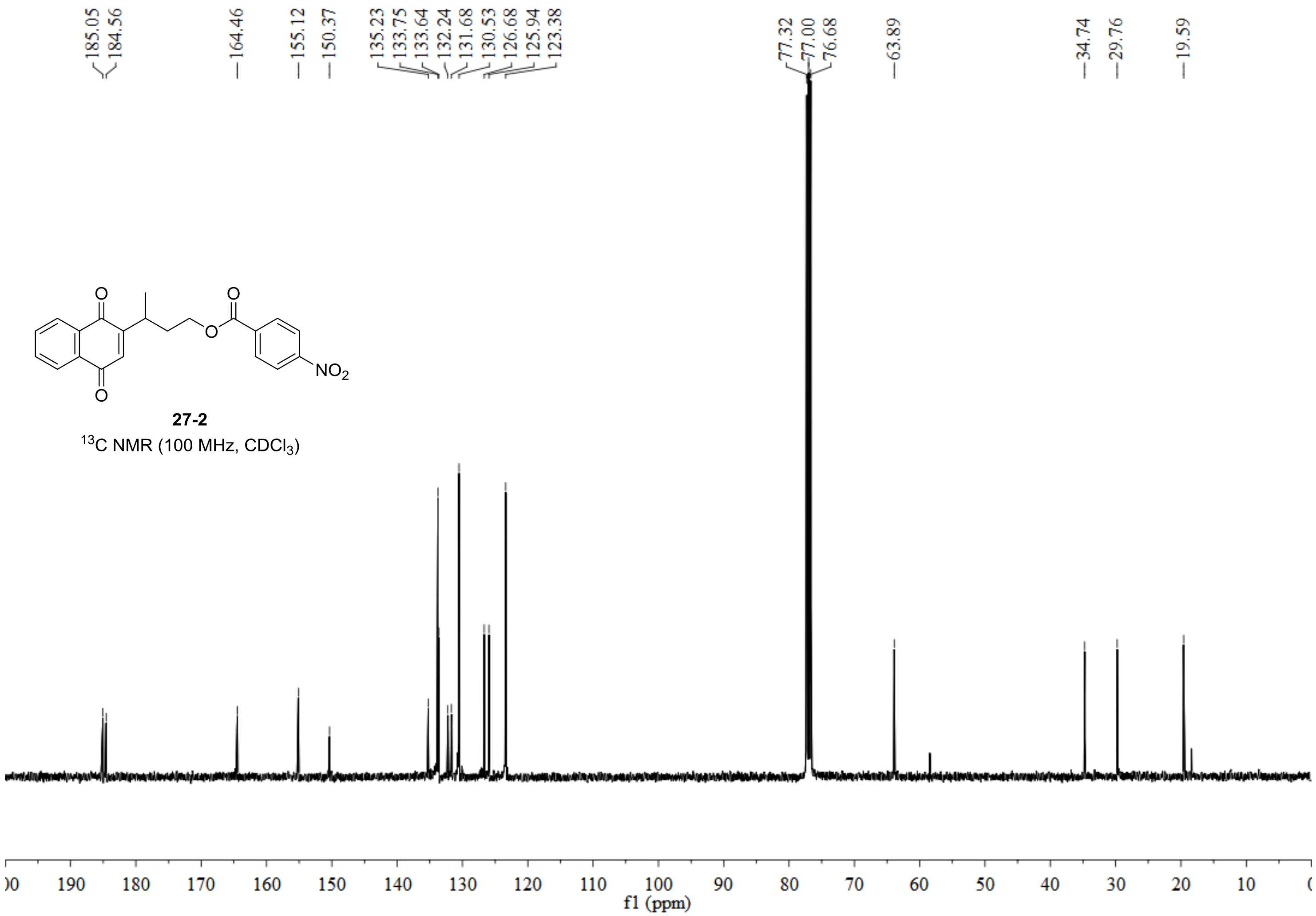
27-2
13C NMR (100 MHz, CDCl3)
185.05
184.56
164.46
155.12
150.37
135.23
133.75
133.64
132.24
131.68
130.53
126.68
125.94
123.38
77.32
77.00
76.68
63.89
34.74
29.76
19.59
f1 (ppm)

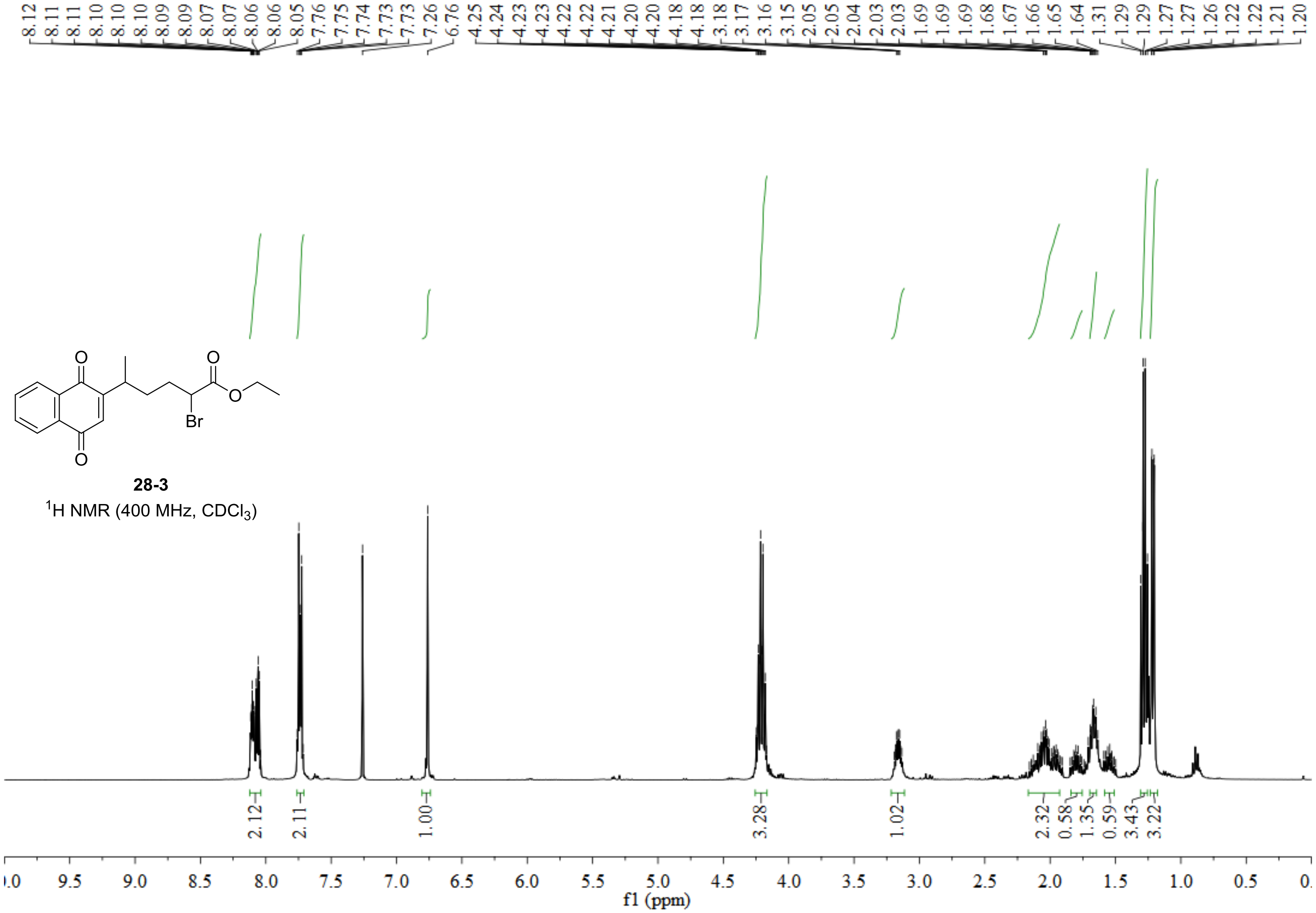

28-3

^1H NMR (400 MHz, $CDCl_3$)

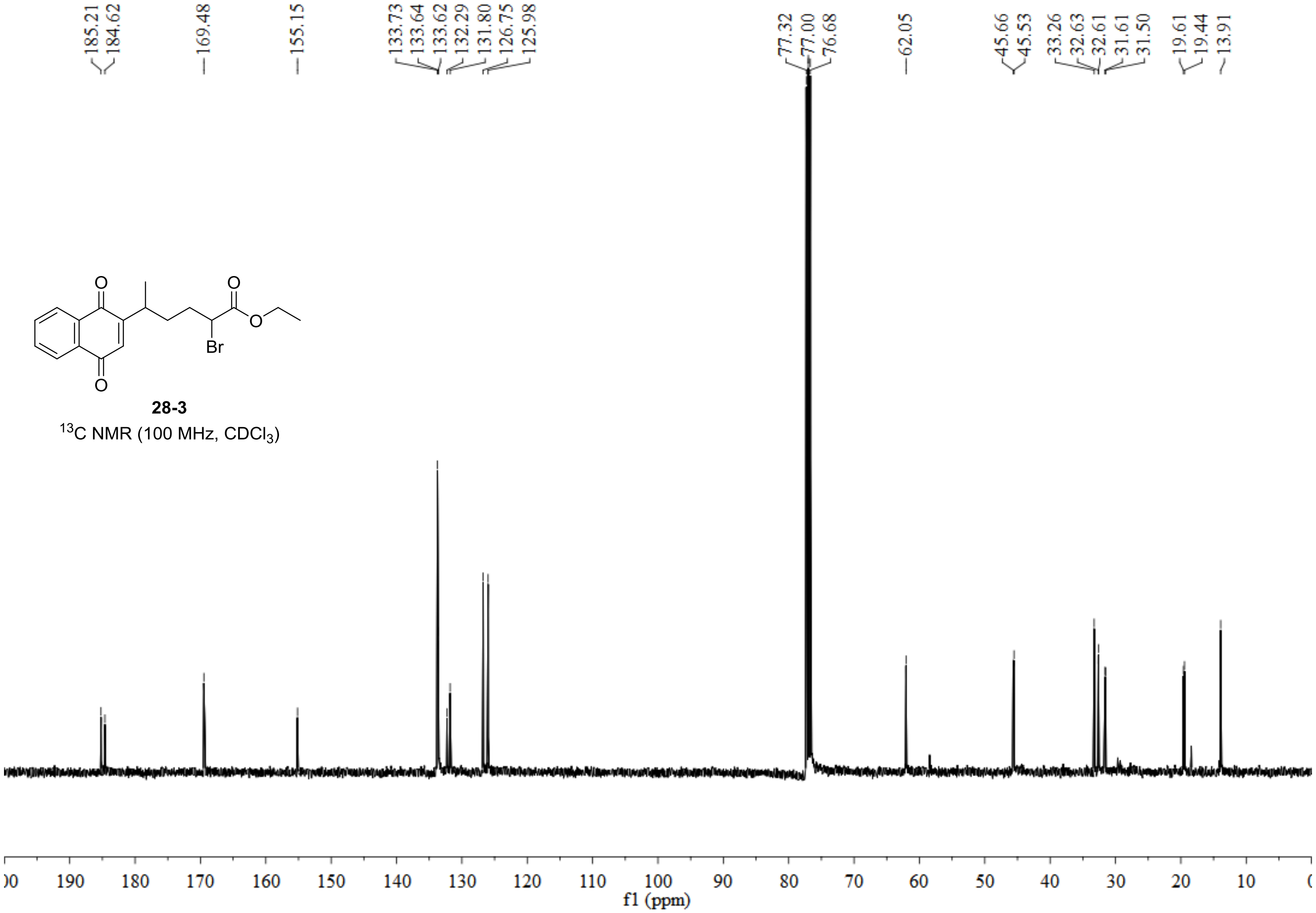
O
O
O
O
Br
28-3
^{13}C NMR (100 MHz, $CDCl_3$)
185.21
184.62
169.48
155.15
133.73
133.64
133.62
132.29
131.80
126.75
125.98
77.32
77.00
76.68
62.05
45.66
45.53
33.26
32.63
32.61
31.61
31.50
19.61
19.44
13.91
f1 (ppm)

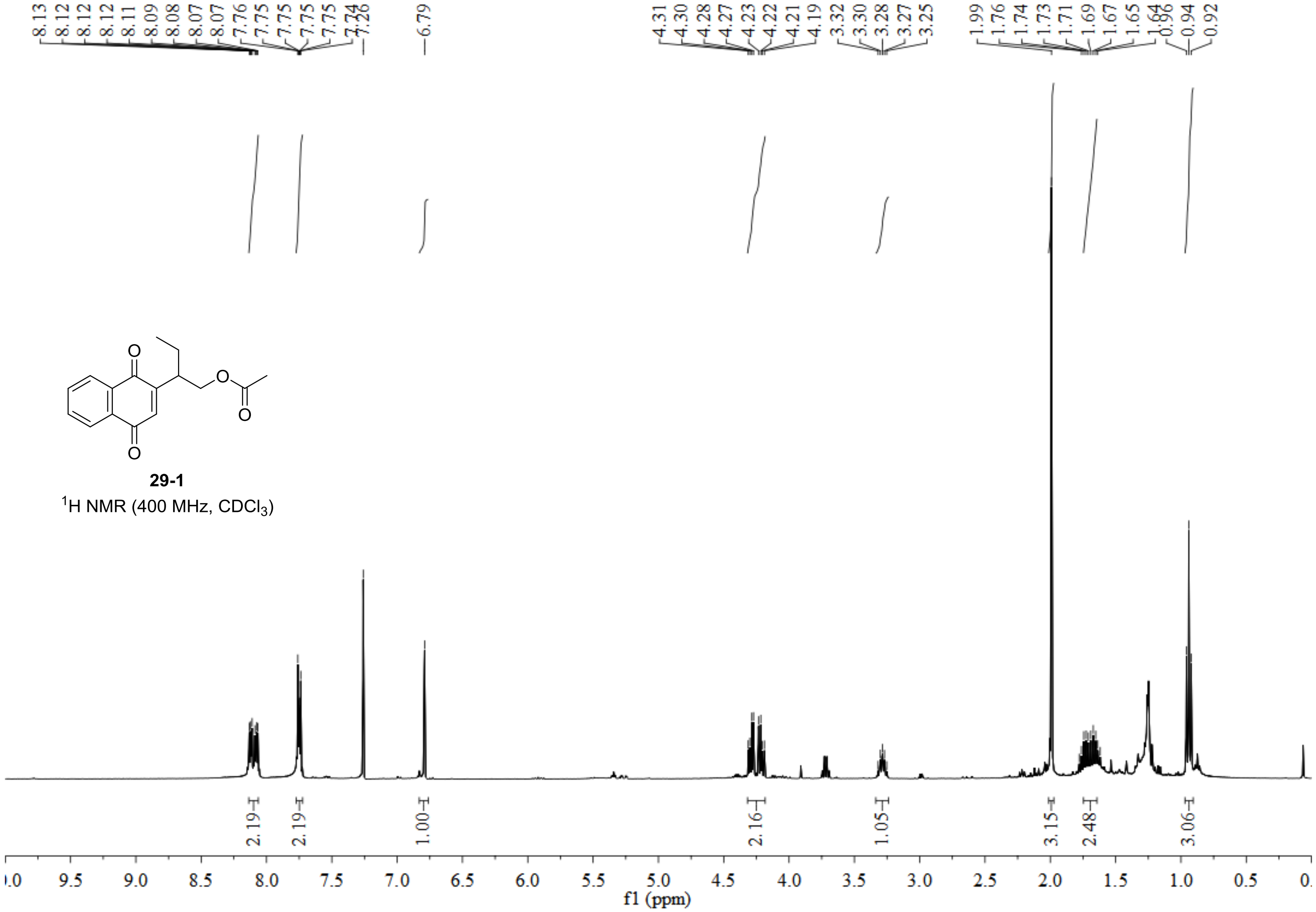

29-1

^{1}H NMR (400 MHz, $CDCl_3$)

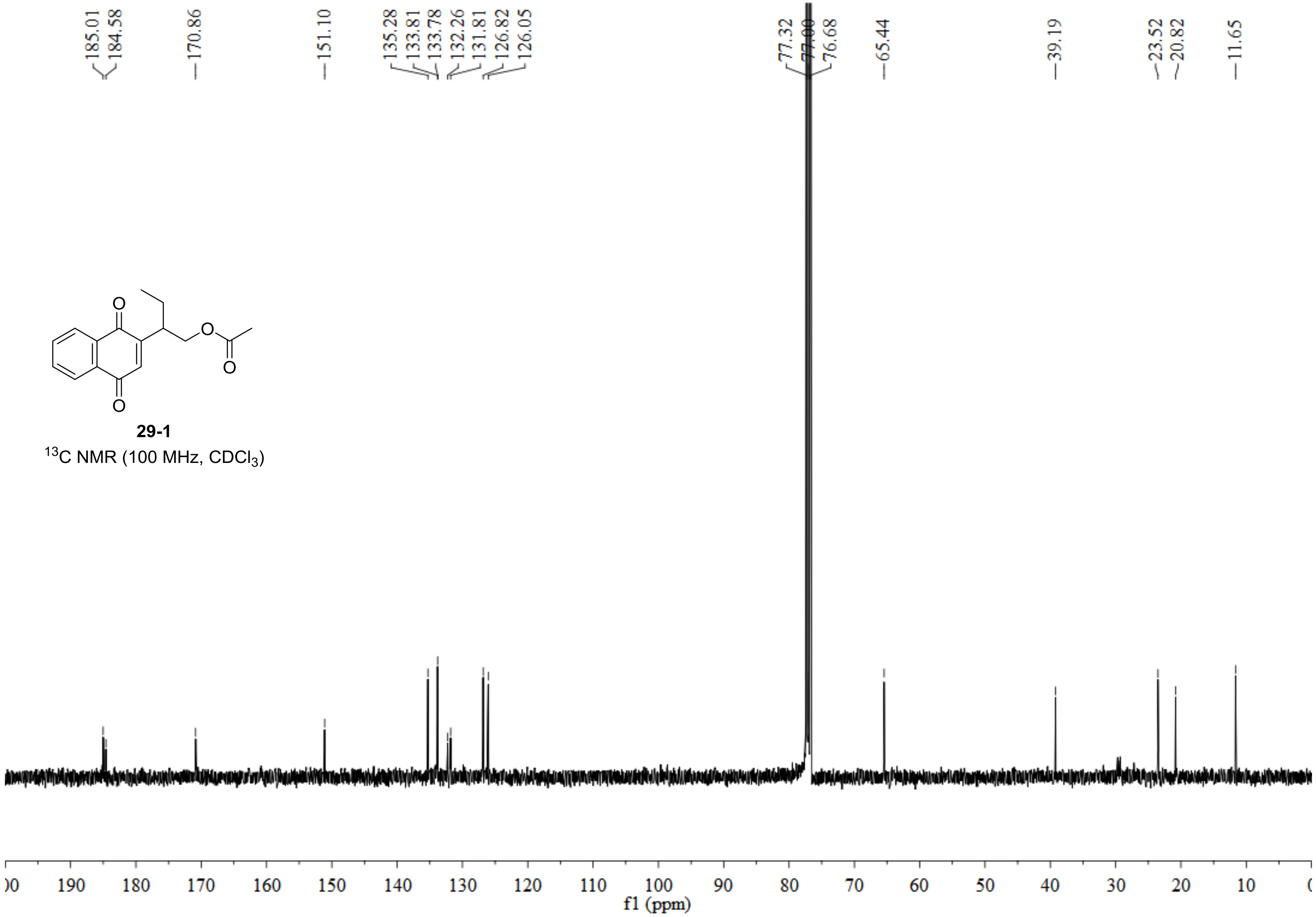

29-1

^{13}C NMR (100 MHz, $CDCl_3$)

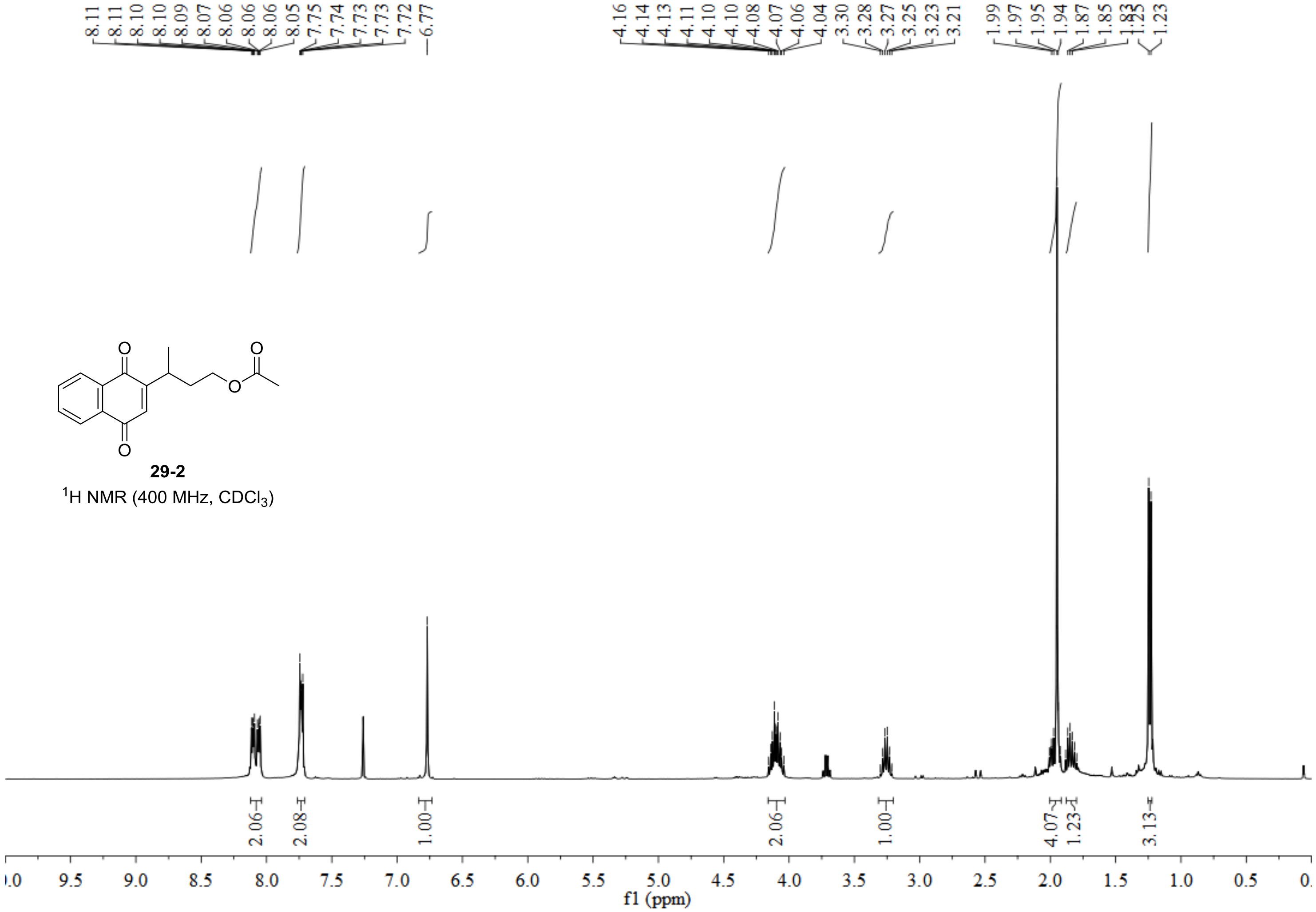

29-2
^{1}H NMR (400 MHz, $CDCl_3$)

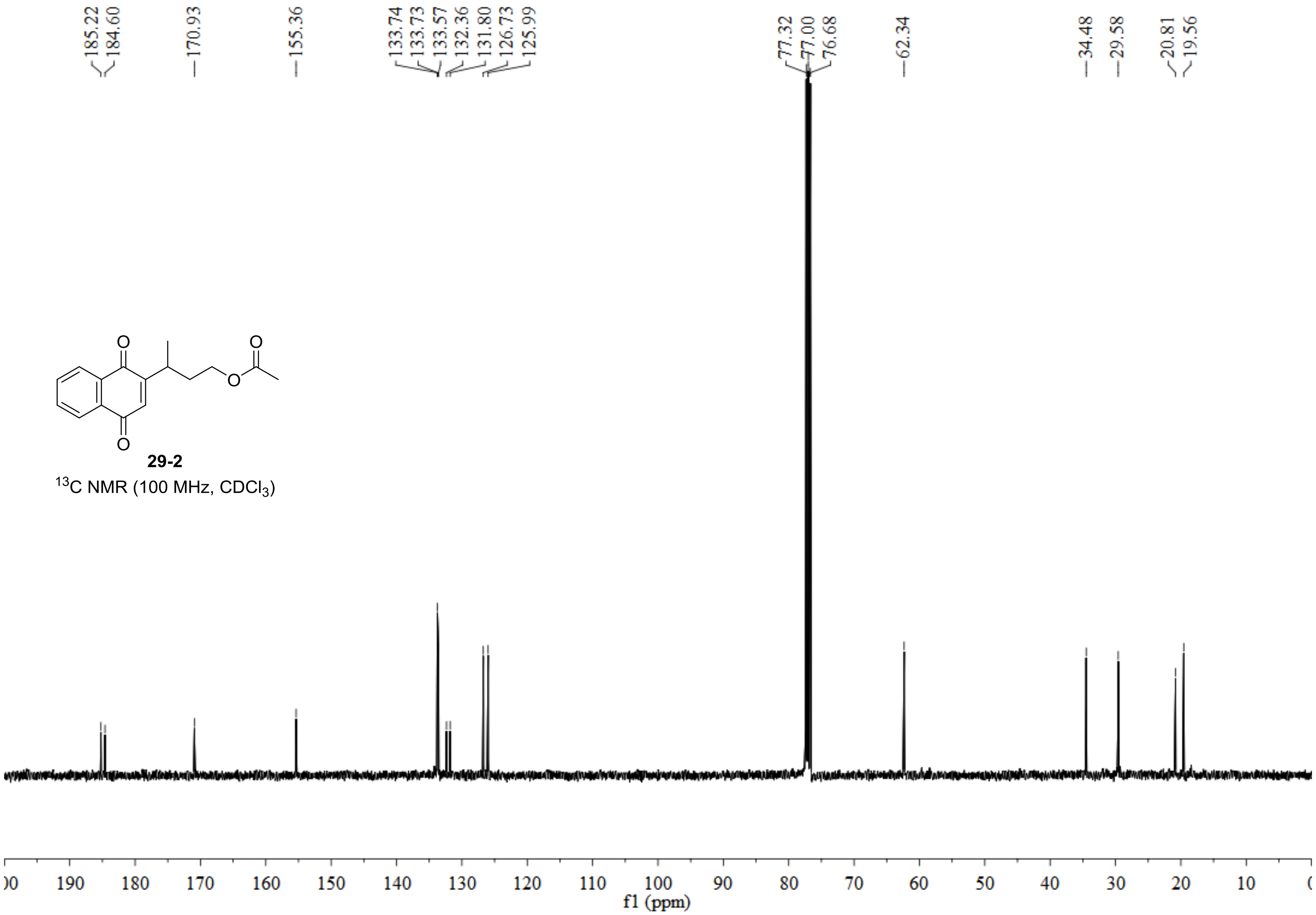

29-2

^{13}C NMR (100 MHz, $CDCl_3$)

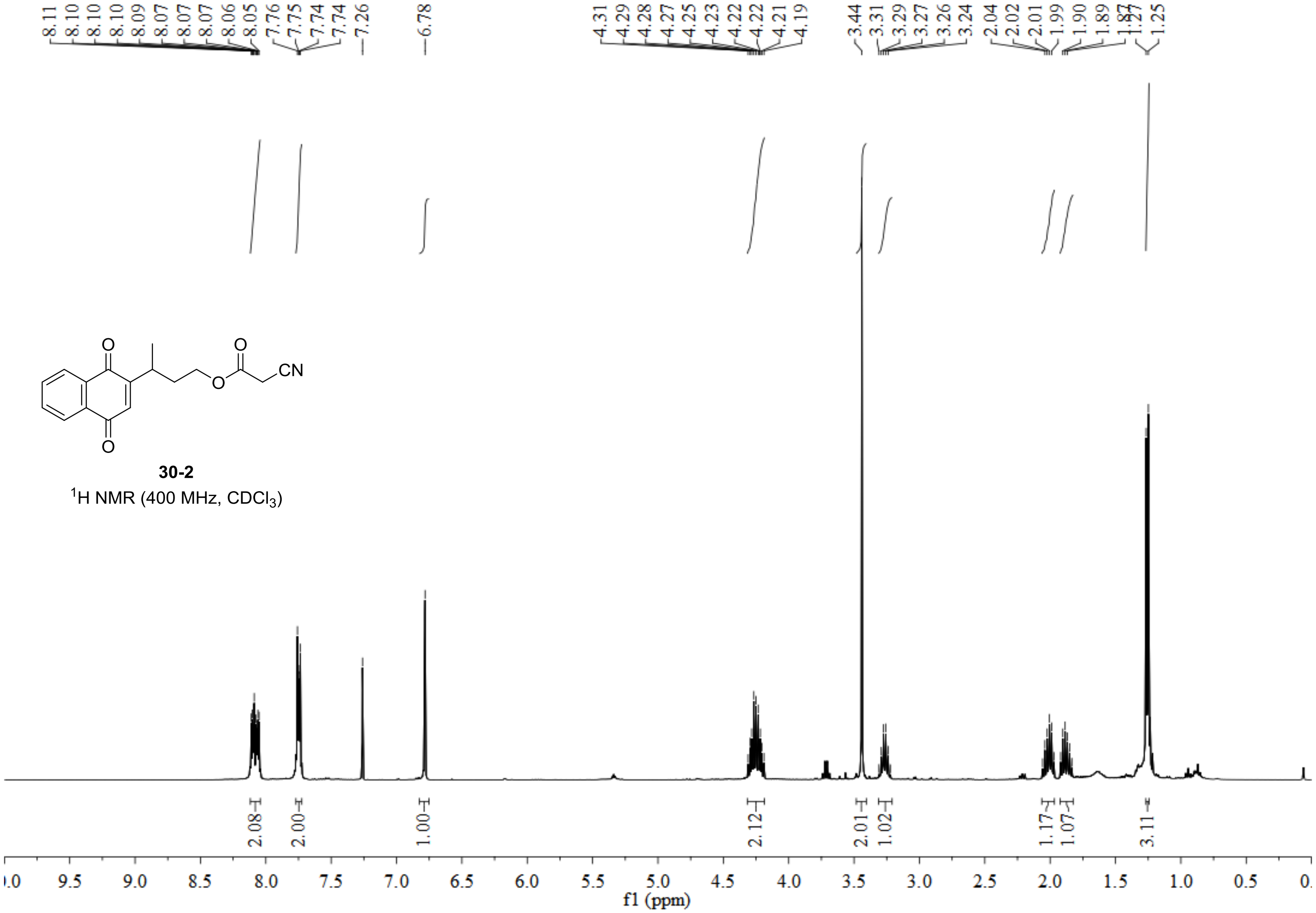

30-2

^{1}H NMR (400 MHz, $CDCl_3$)

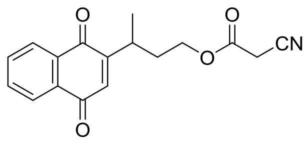

30-2

^{13}C NMR (100 MHz, $CDCl_3$)

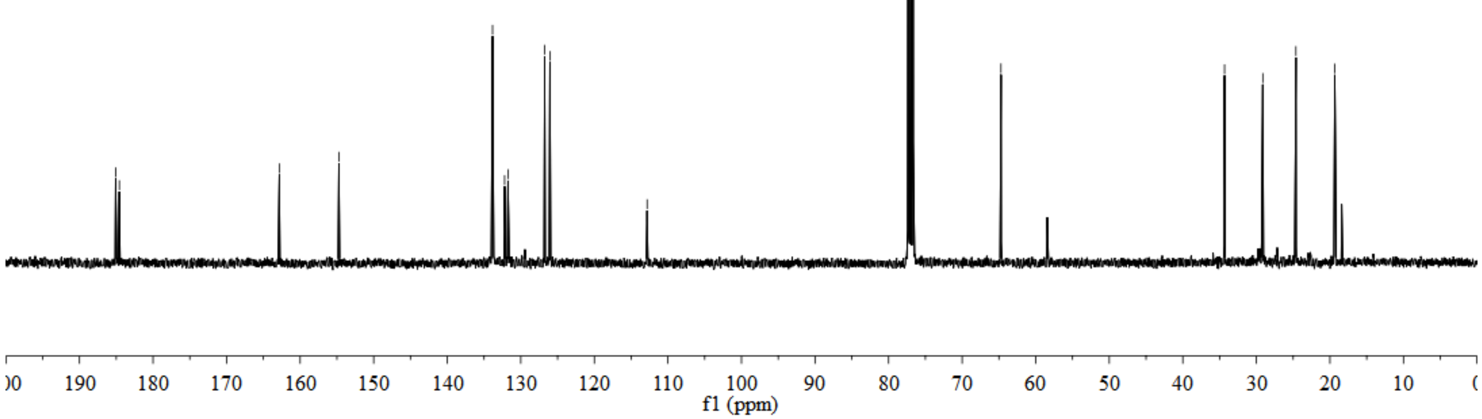

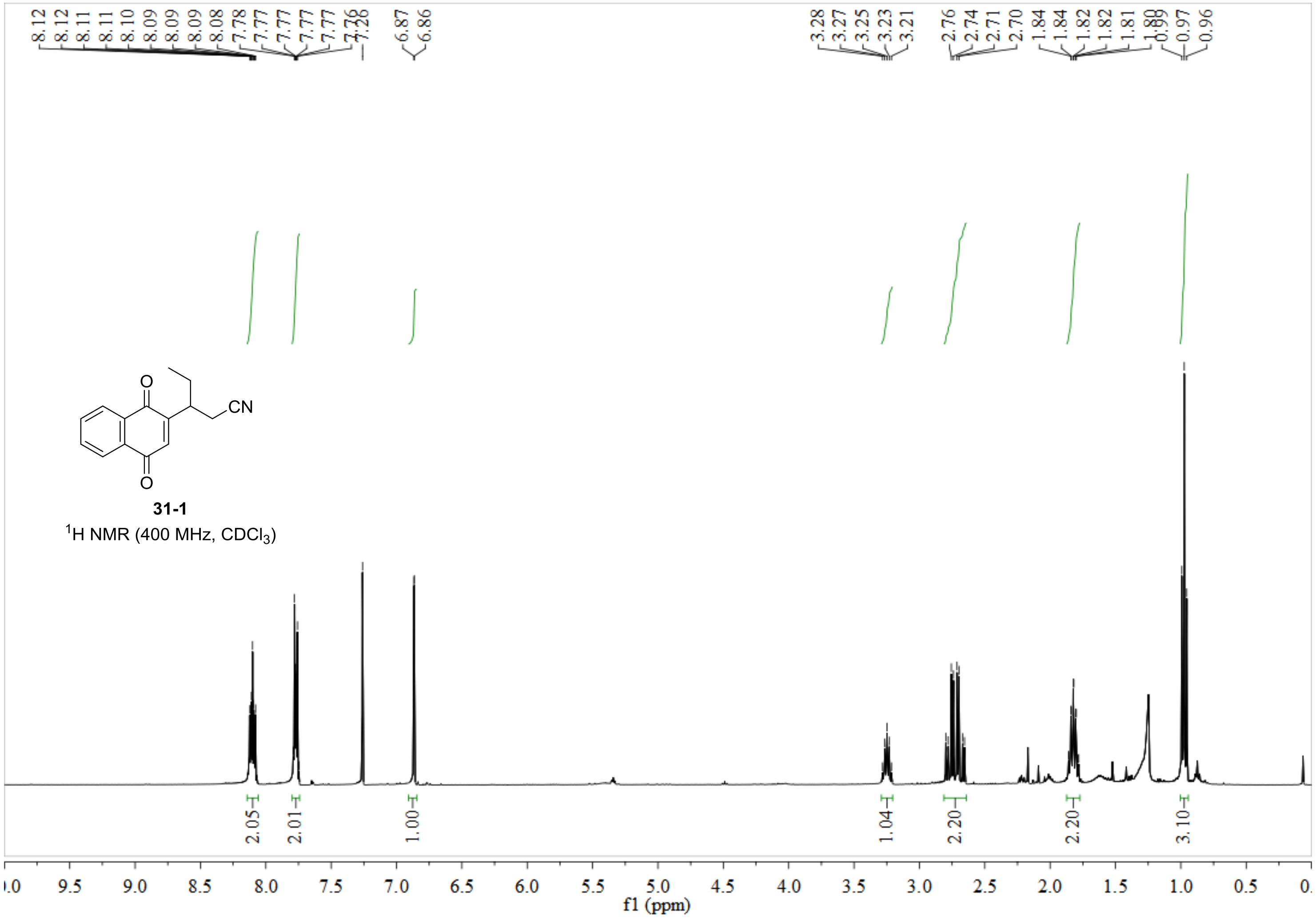

31-1

^{1}H NMR (400 MHz, $CDCl_3$)

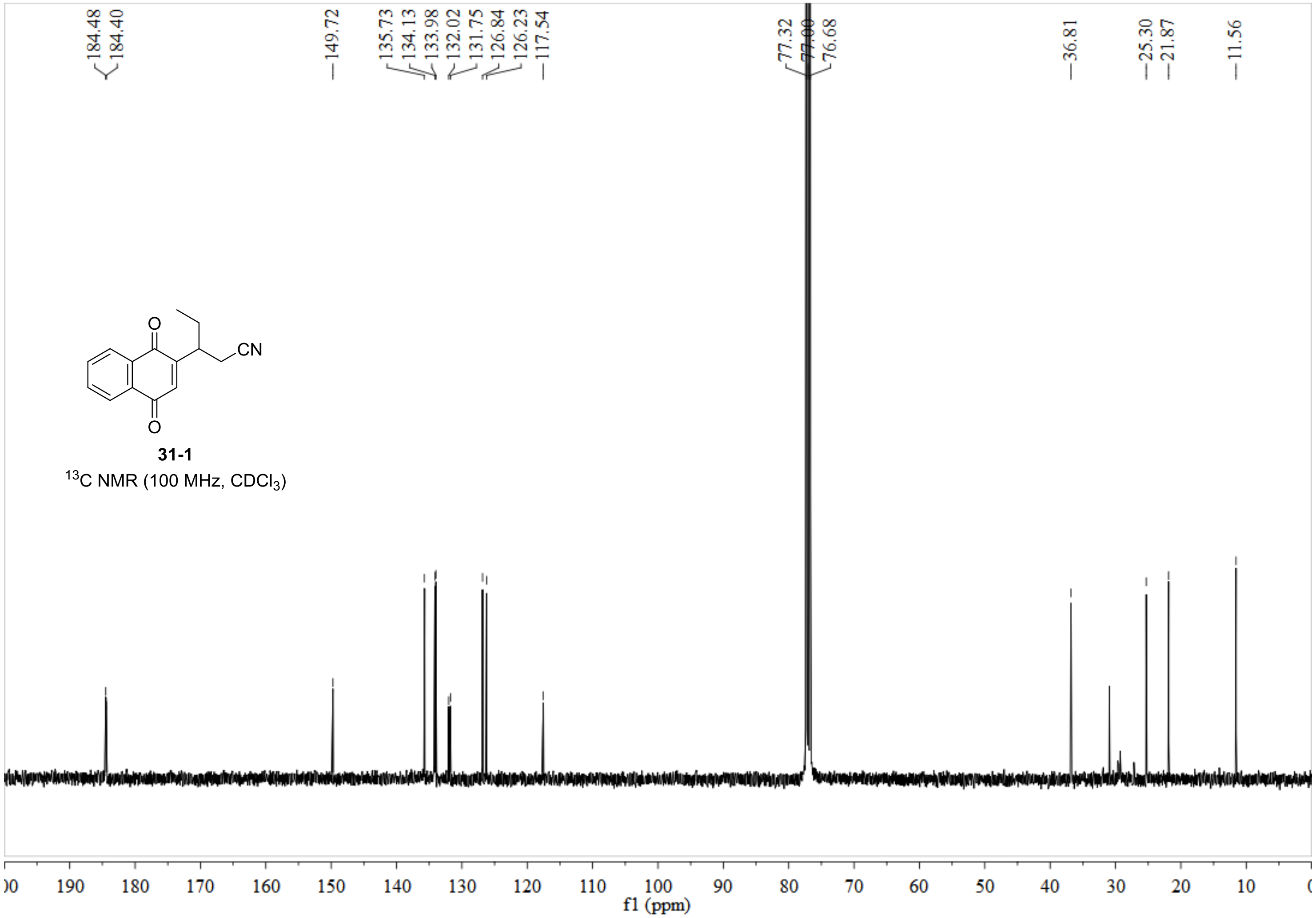

31-1

^{13}C NMR (100 MHz, $CDCl_3$)

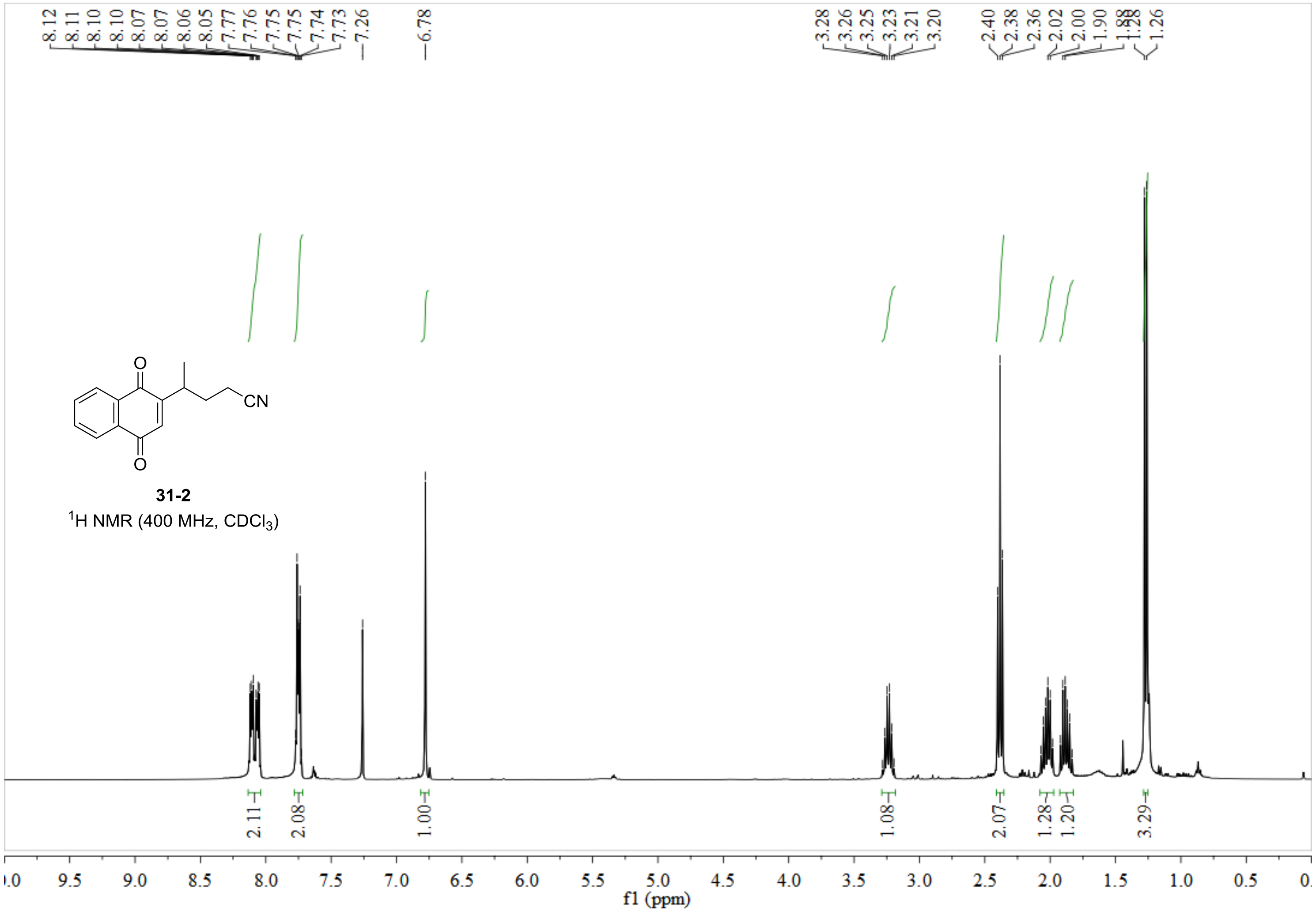

31-2

^{1}H NMR (400 MHz, $CDCl_3$)

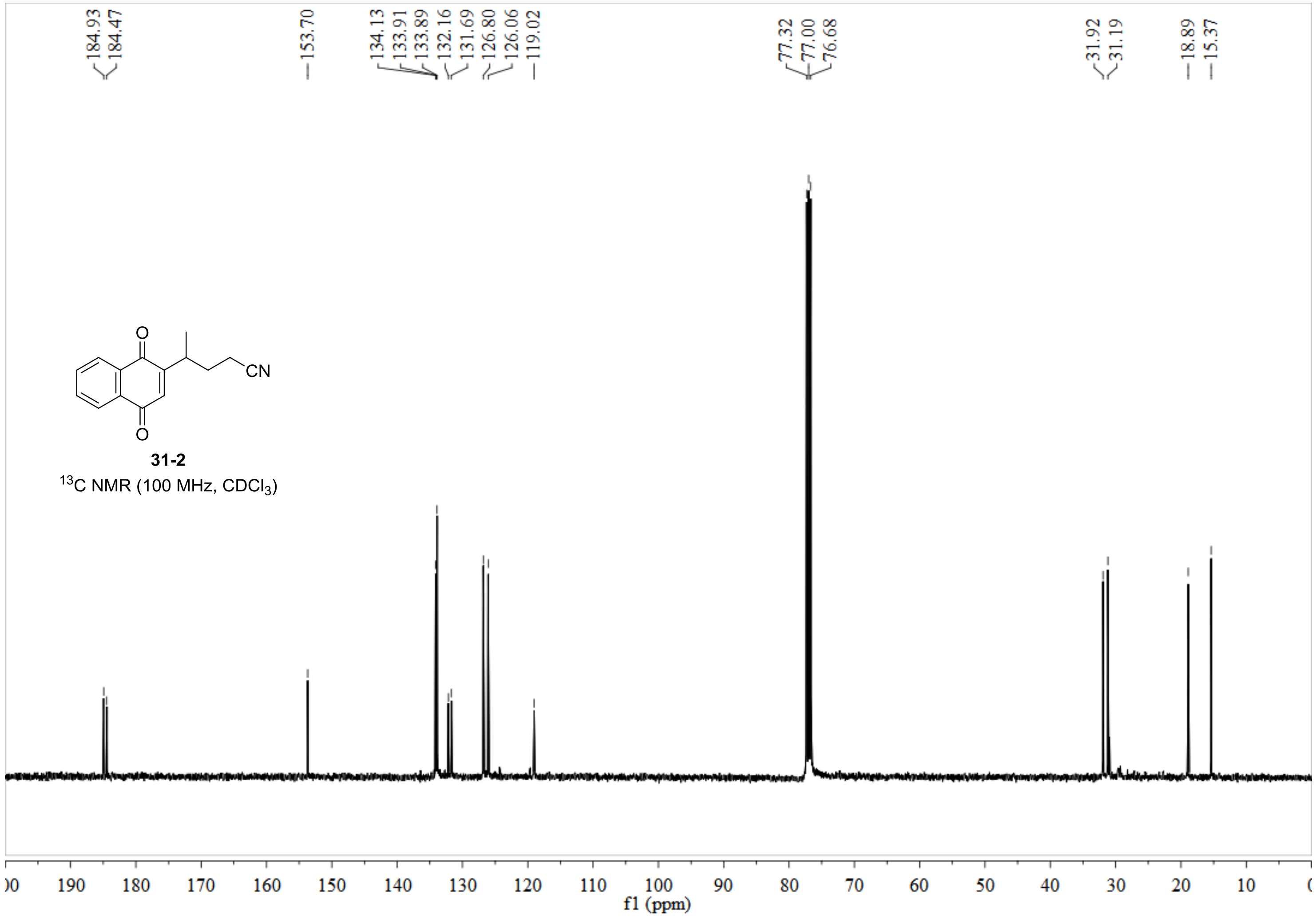
184.93
184.47
153.70
134.13
133.91
133.89
132.16
131.69
126.80
126.06
119.02
77.32
77.00
76.68
31.92
31.19
18.89
15.37
O
CN
O
31-2
13C NMR (100 MHz, CDCl3)
190
180
170
160
150
140
130
120
110
100
90
80
70
60
50
40
30
20
10
f1 (ppm)

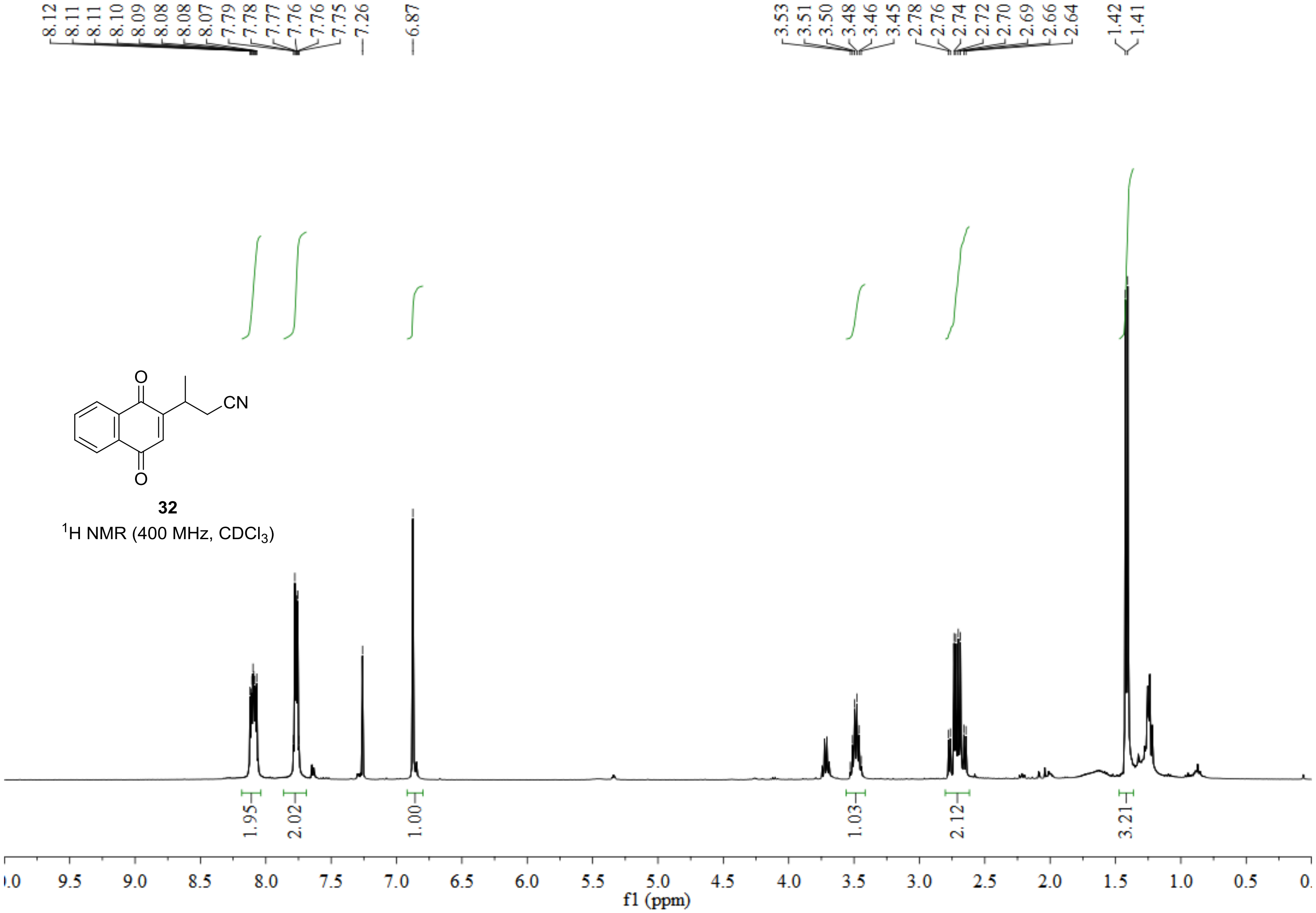
8.12 8.11 8.11 8.10 8.09 8.08 8.08 8.07 7.79 7.78 7.77 7.76 7.76 7.75 7.26 6.87
3.53 3.51 3.50 3.48 3.46 3.45 2.78 2.76 2.74 2.72 2.70 2.69 2.66 2.64 1.42 1.41
O
CN
O
32
1H NMR (400 MHz, CDCl3)
1.95 2.02 1.00 1.03 2.12 3.21
9.5 9.0 8.5 8.0 7.5 7.0 6.5 6.0 5.5 5.0 4.5 4.0 3.5 3.0 2.5 2.0 1.5 1.0 0.5
f1 (ppm)

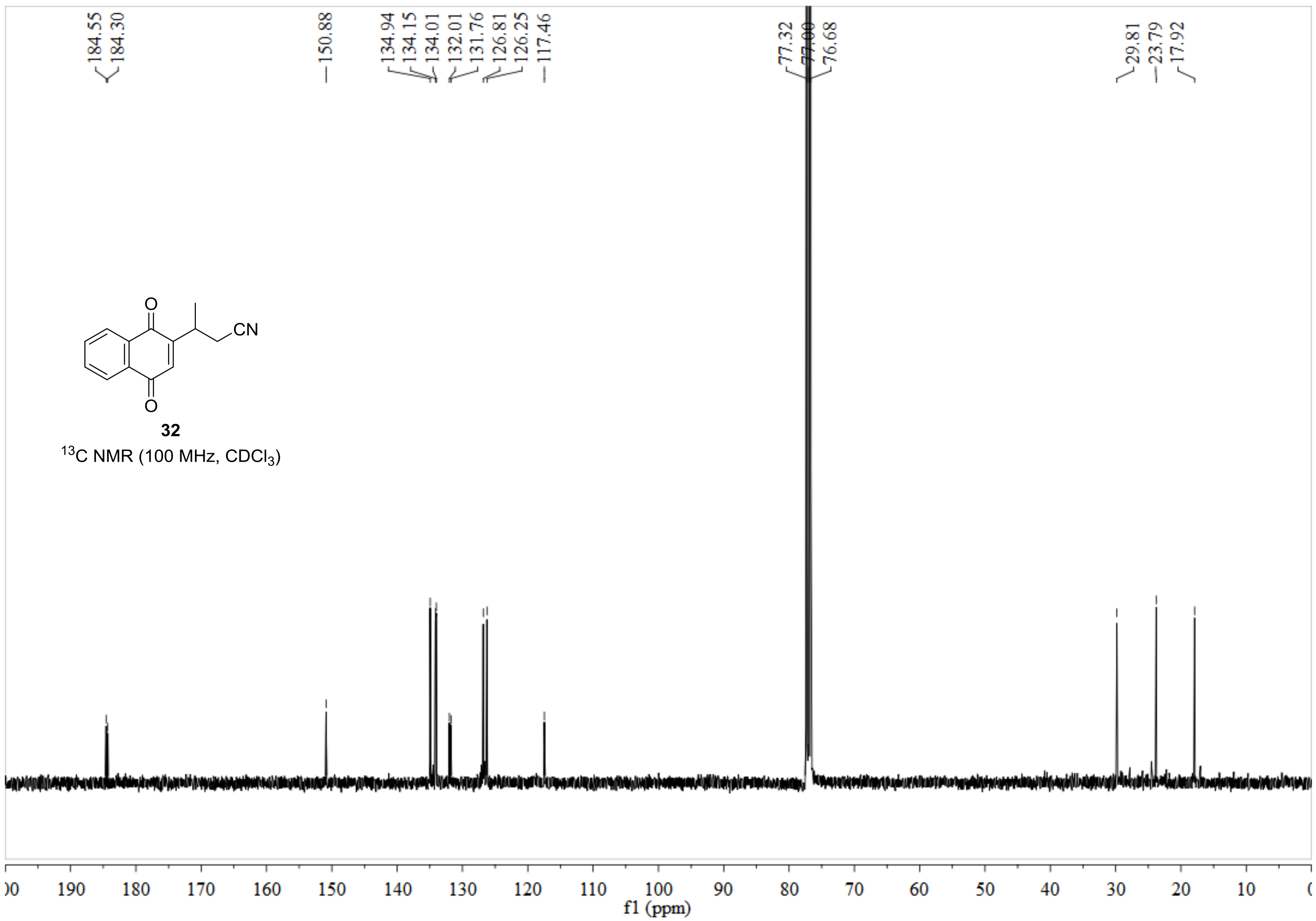

32

^{13}C NMR (100 MHz, $CDCl_3$)

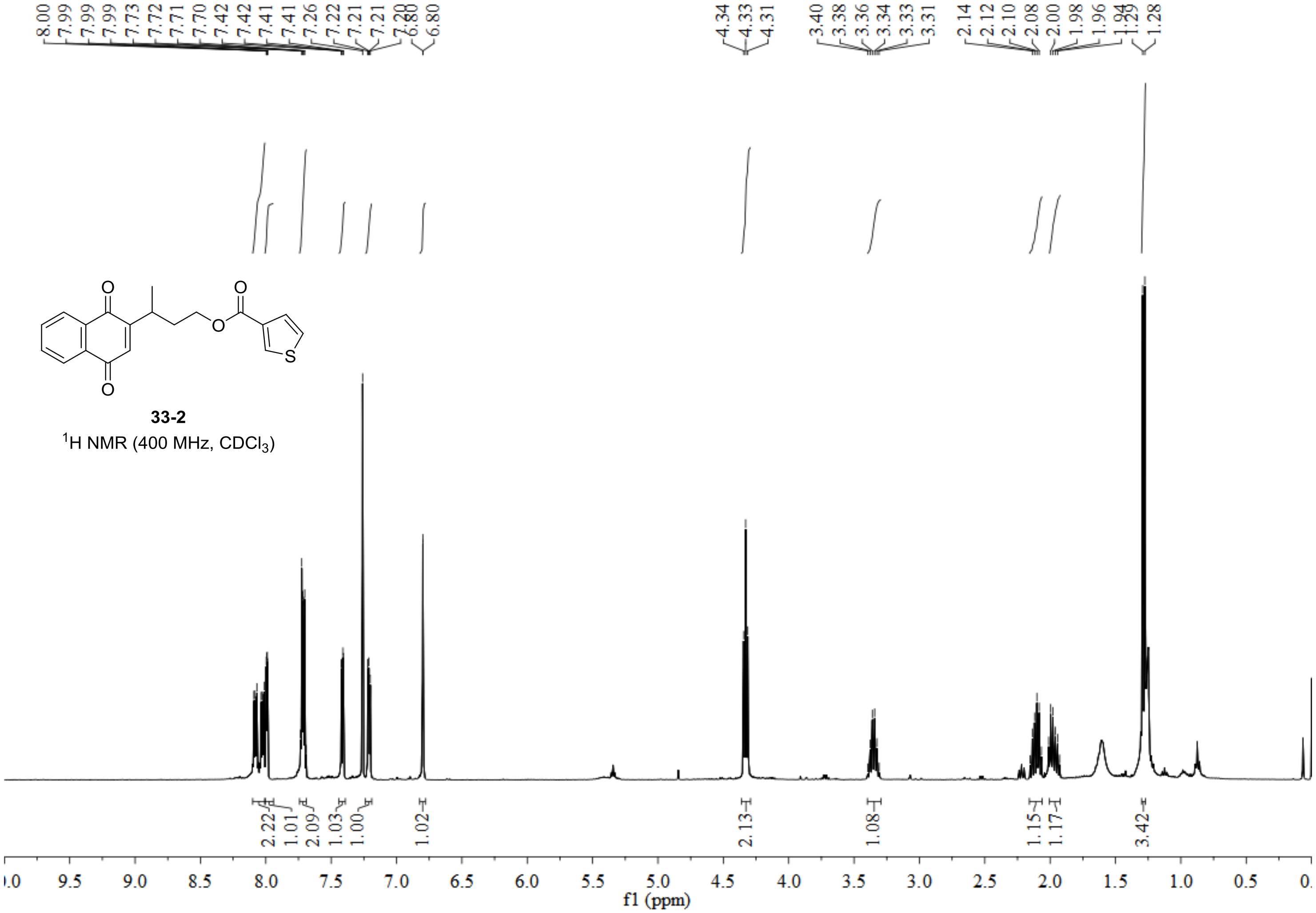

33-2

^1H NMR (400 MHz, $CDCl_3$)

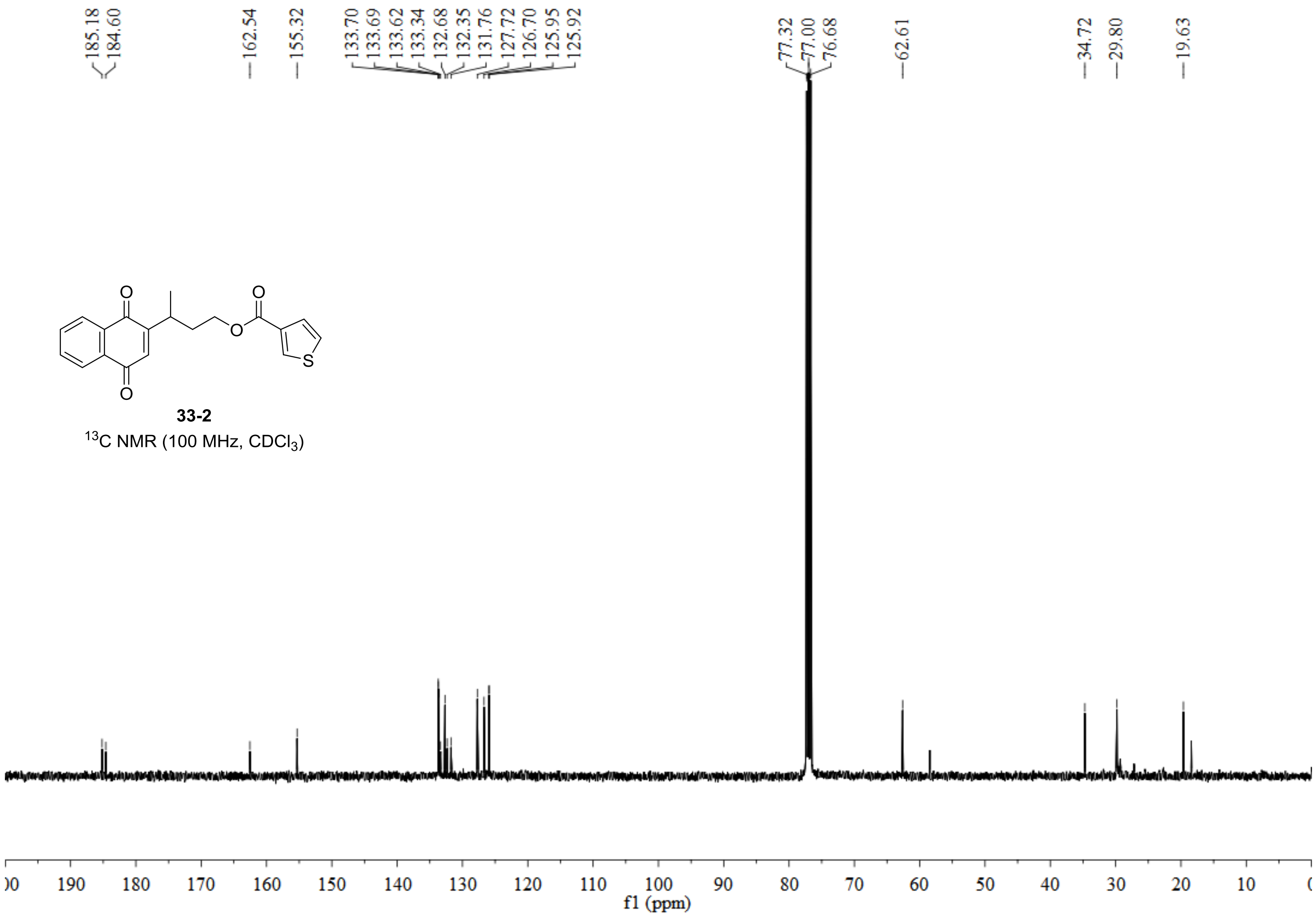

33-2

^{13}C NMR (100 MHz, $CDCl_3$)

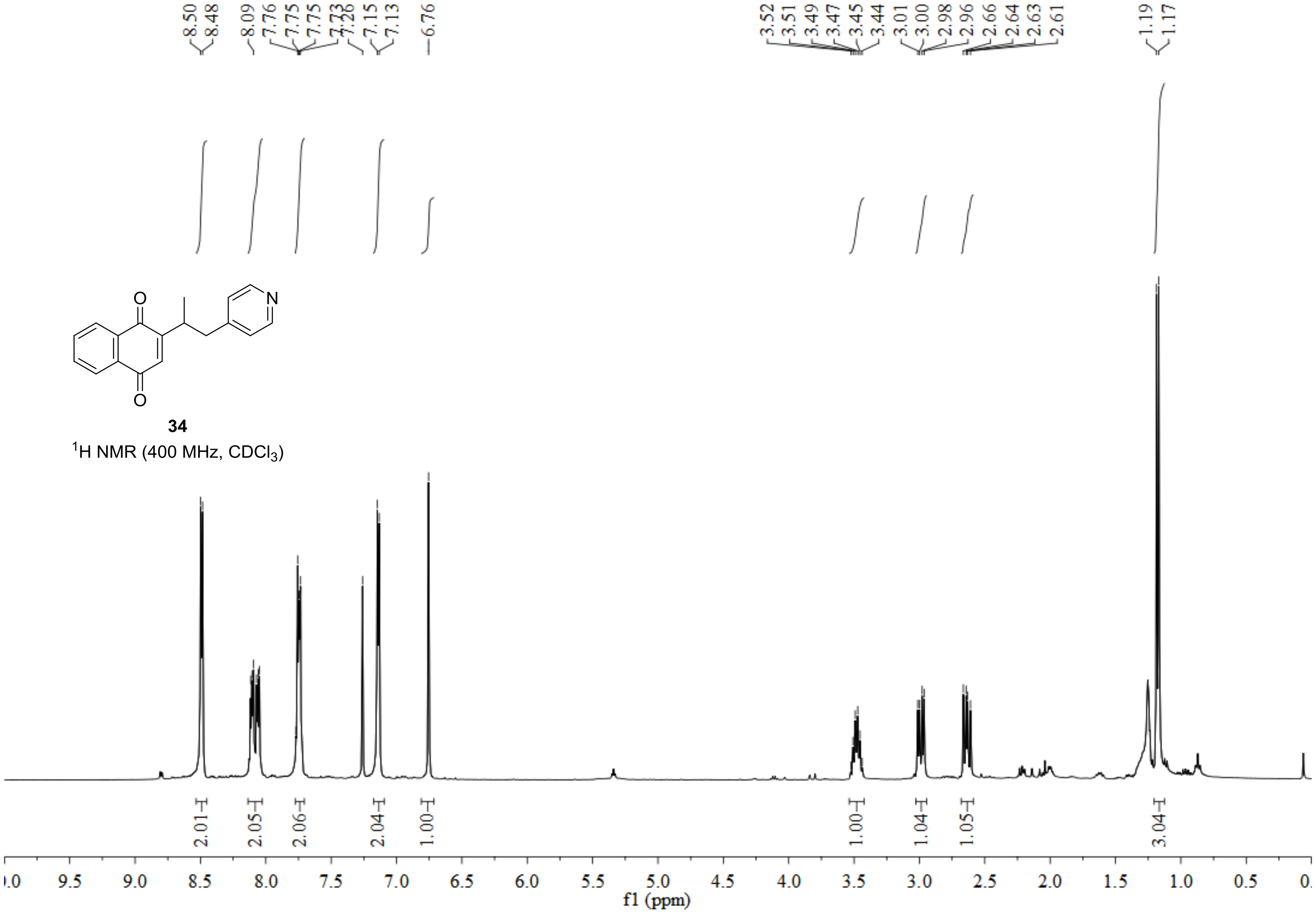
8.50
8.48
8.09
7.76
7.75
7.75
7.73
7.26
7.15
7.13
6.76
3.52
3.51
3.49
3.47
3.45
3.44
3.01
3.00
2.98
2.96
2.66
2.64
2.63
2.61
1.19
1.17
34
^{1}H NMR (400 MHz, $CDCl_3$)
2.01
2.05
2.06
2.04
1.00
1.00
1.04
1.05
3.04
f1 (ppm)

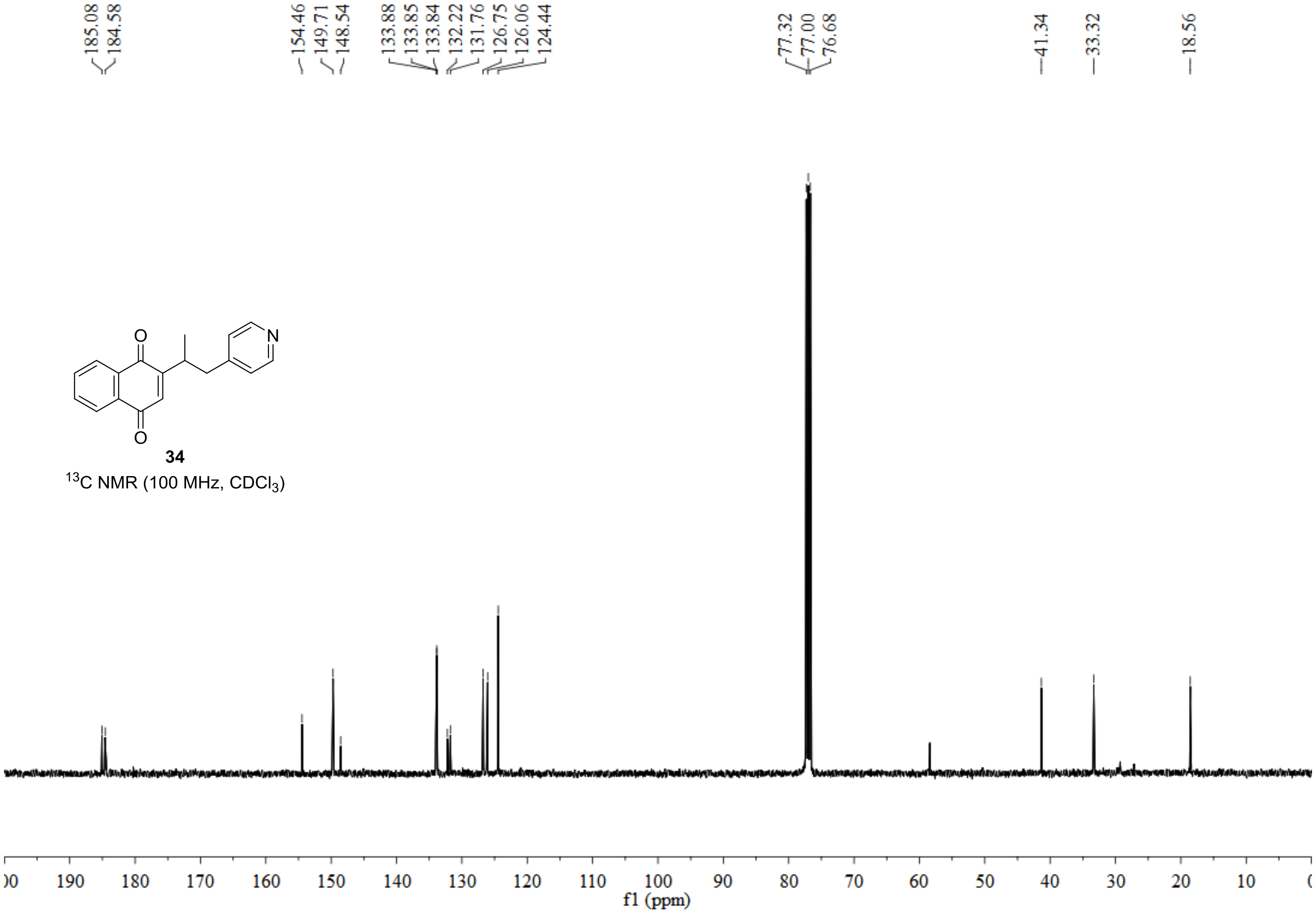
185.08
184.58
154.46
149.71
148.54
133.88
133.85
133.84
132.22
131.76
126.75
126.06
124.44
77.32
77.00
76.68
41.34
33.32
18.56
O
N
O
34
^{13}C NMR (100 MHz, CDCl$_3$)
190
180
170
160
150
140
130
120
110
100
90
80
70
60
50
40
30
20
10
f1 (ppm)

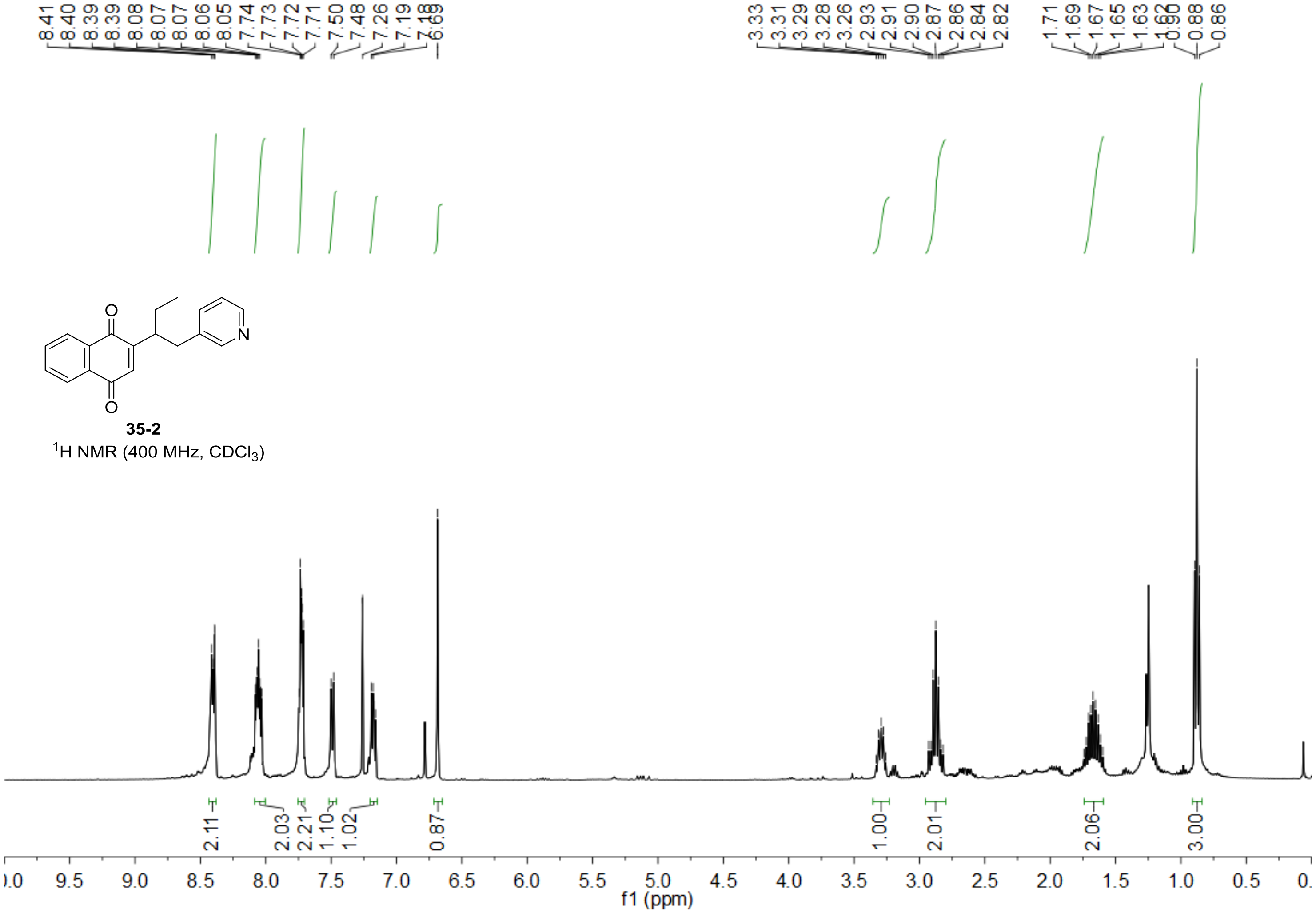

35-2

^{1}H NMR (400 MHz, $CDCl_3$)

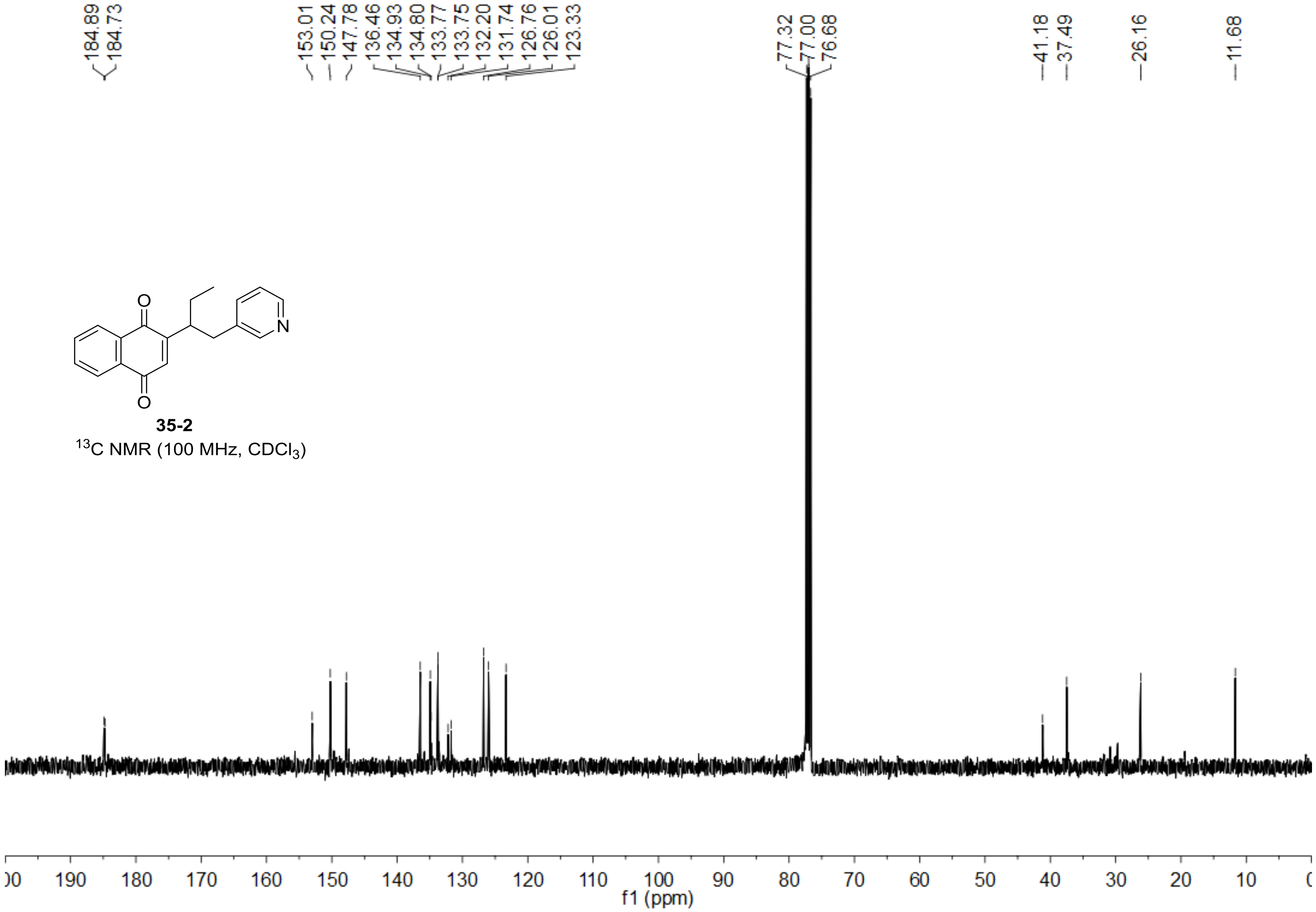
184.89
184.73
153.01
150.24
147.78
136.46
134.93
134.80
133.77
133.75
132.20
131.74
126.76
126.01
123.33
77.32
77.00
76.68
41.18
37.49
26.16
11.68
O
N
O
35-2
13C NMR (100 MHz, CDCl3)
f1 (ppm)

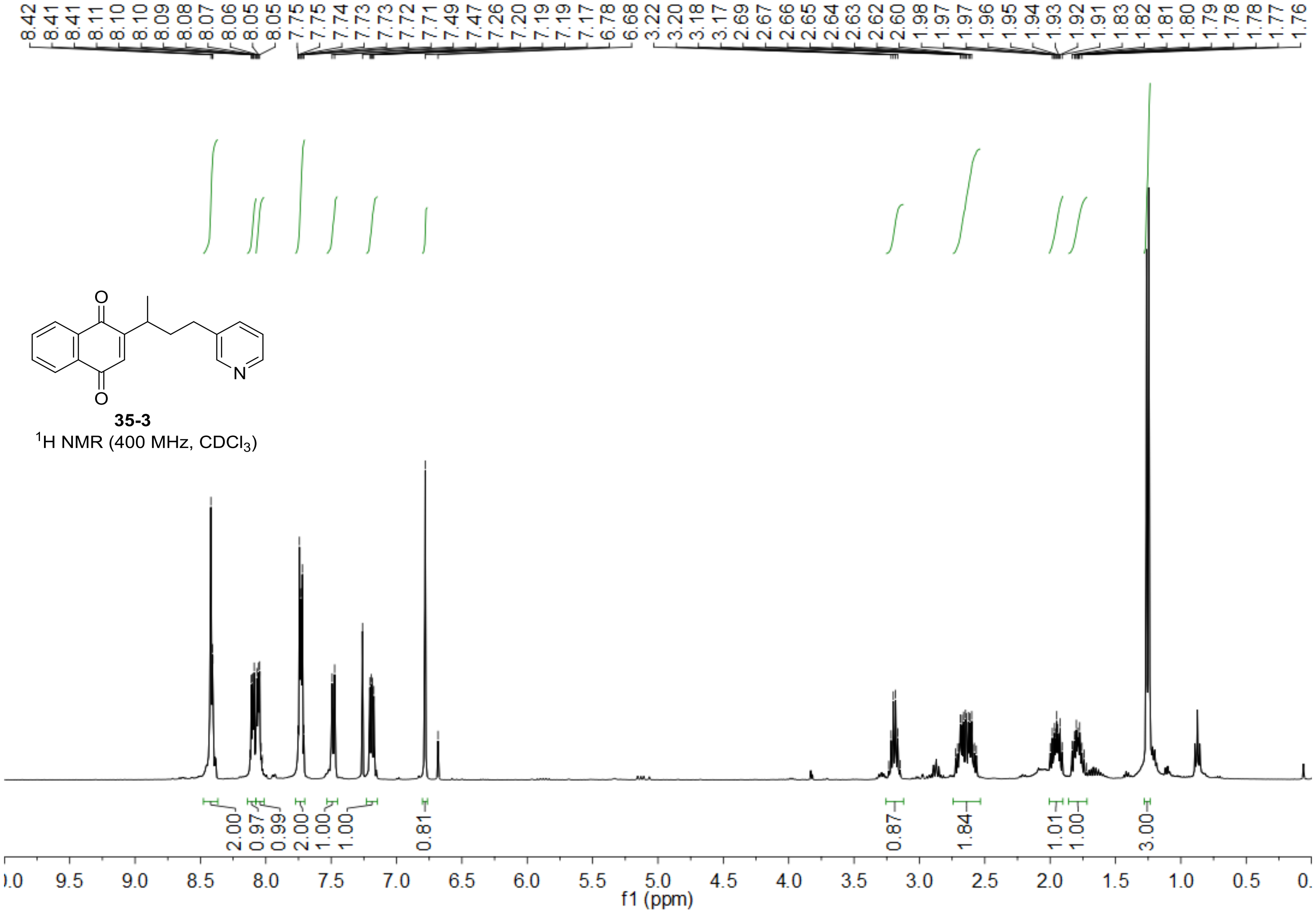

35-3
^{1}H NMR (400 MHz, CDCl3)
f1 (ppm)

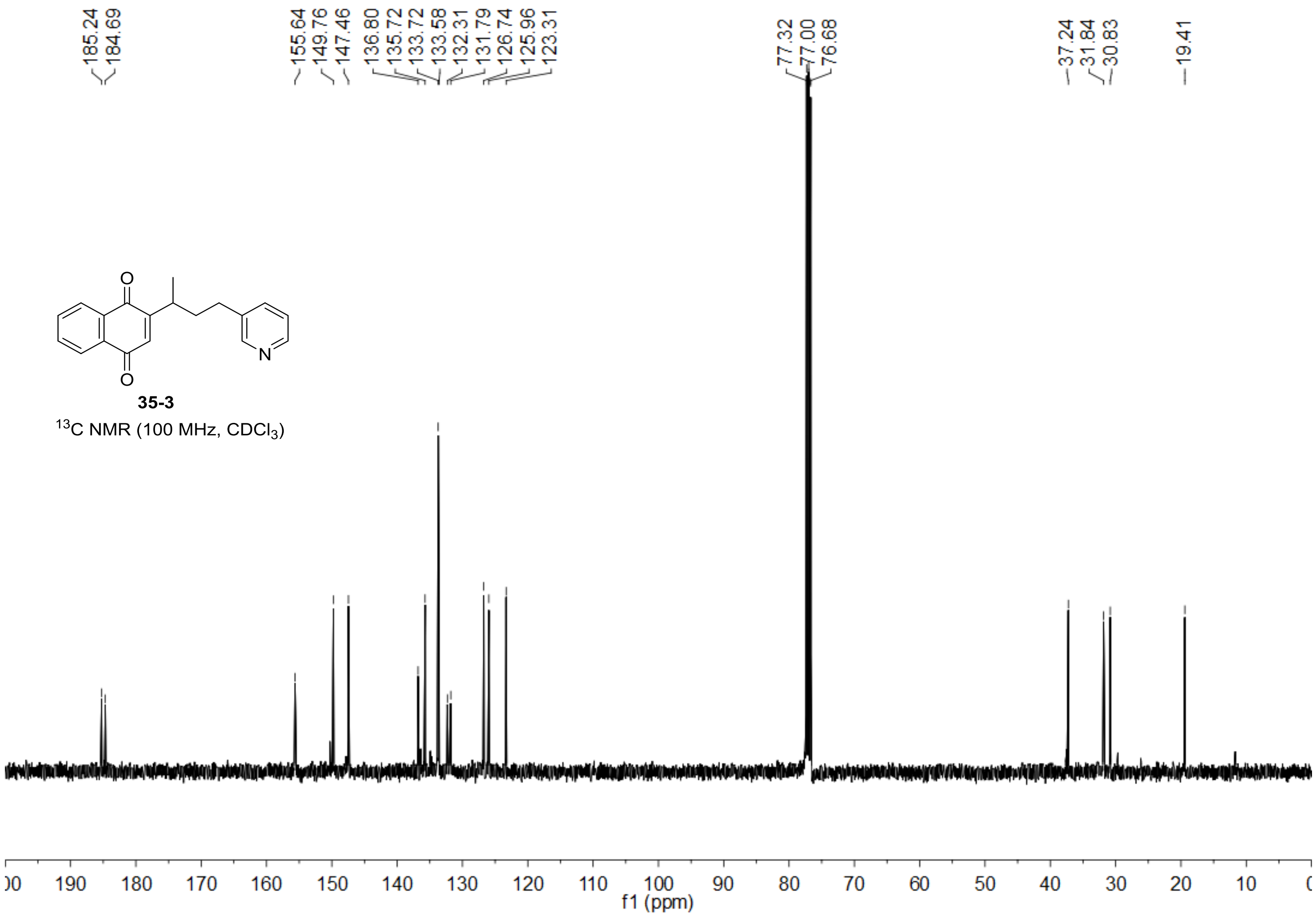

185.24
184.69
155.64
149.76
147.46
136.80
135.72
133.72
133.58
132.31
131.79
126.74
125.96
123.31
77.32
77.00
76.68
37.24
31.84
30.83
19.41
O
O
N
35-3
^{13}C NMR (100 MHz, $CDCl_3$)
190
180
170
160
150
140
130
120
110
100
90
80
70
60
50
40
30
20
10
f1 (ppm)

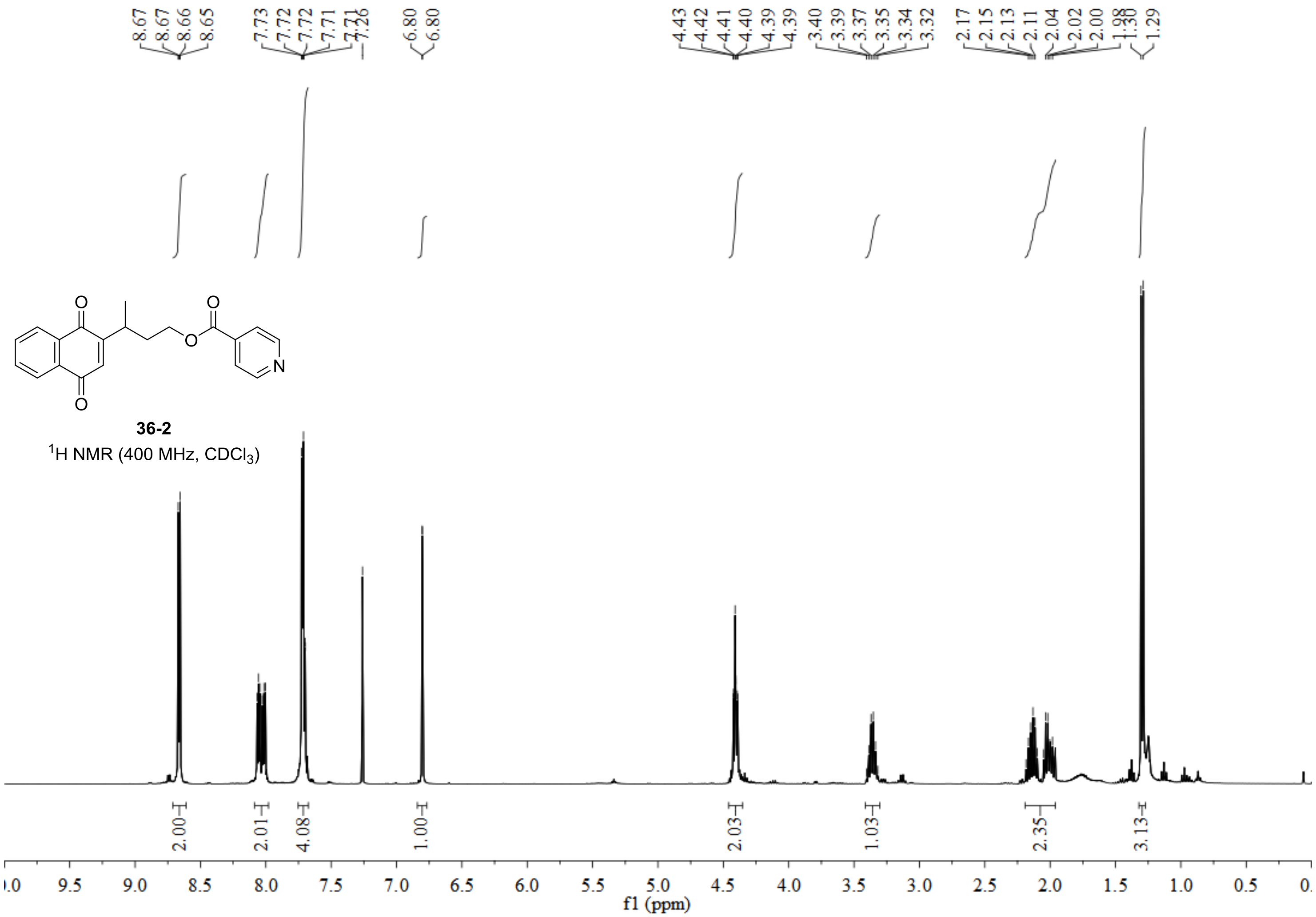

8.67
8.67
8.66
8.65
7.73
7.72
7.72
7.71
7.71
7.26
6.80
6.80
4.43
4.42
4.41
4.40
4.39
4.39
3.40
3.39
3.37
3.35
3.34
3.32
2.17
2.15
2.13
2.11
2.04
2.02
2.00
1.98
1.30
1.29
36-2
1H NMR (400 MHz, CDCl3)
2.00
2.01
4.08
1.00
2.03
1.03
2.35
3.13
9.5 9.0 8.5 8.0 7.5 7.0 6.5 6.0 5.5 5.0 4.5 4.0 3.5 3.0 2.5 2.0 1.5 1.0 0.5
f1 (ppm)

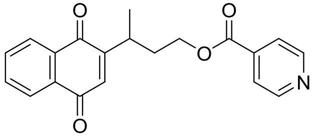

36-2

^{13}C NMR (100 MHz, $CDCl_3$)

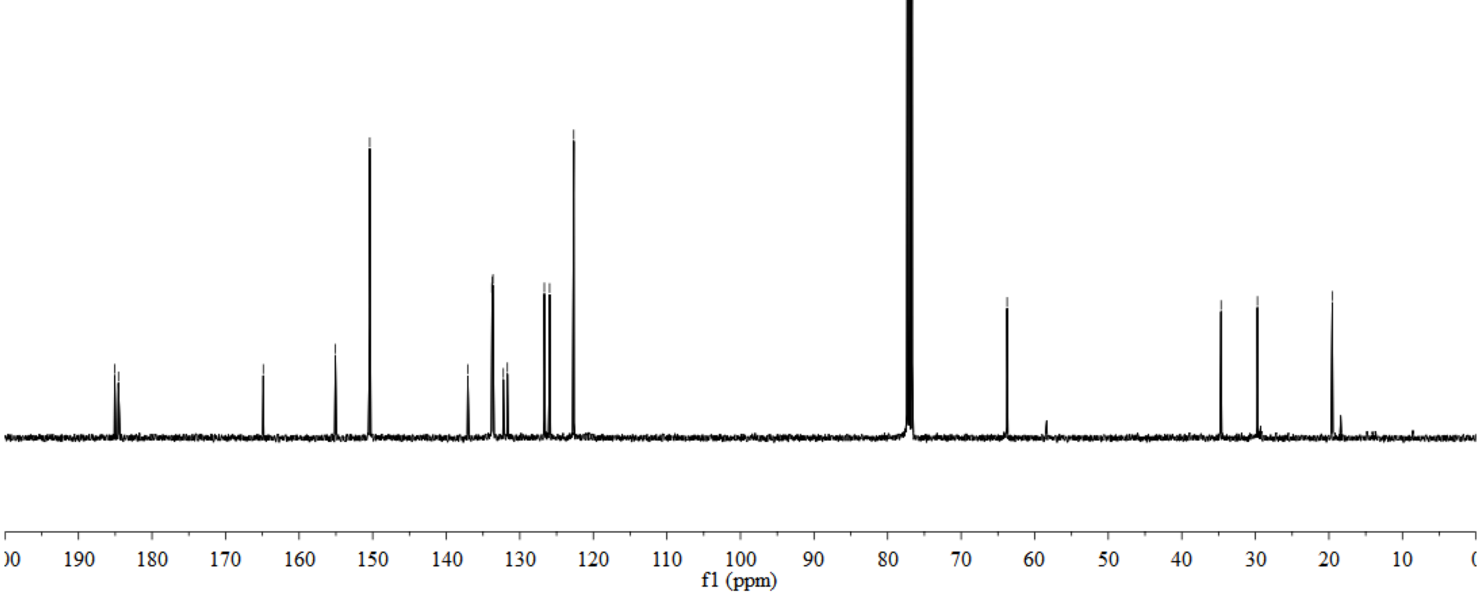

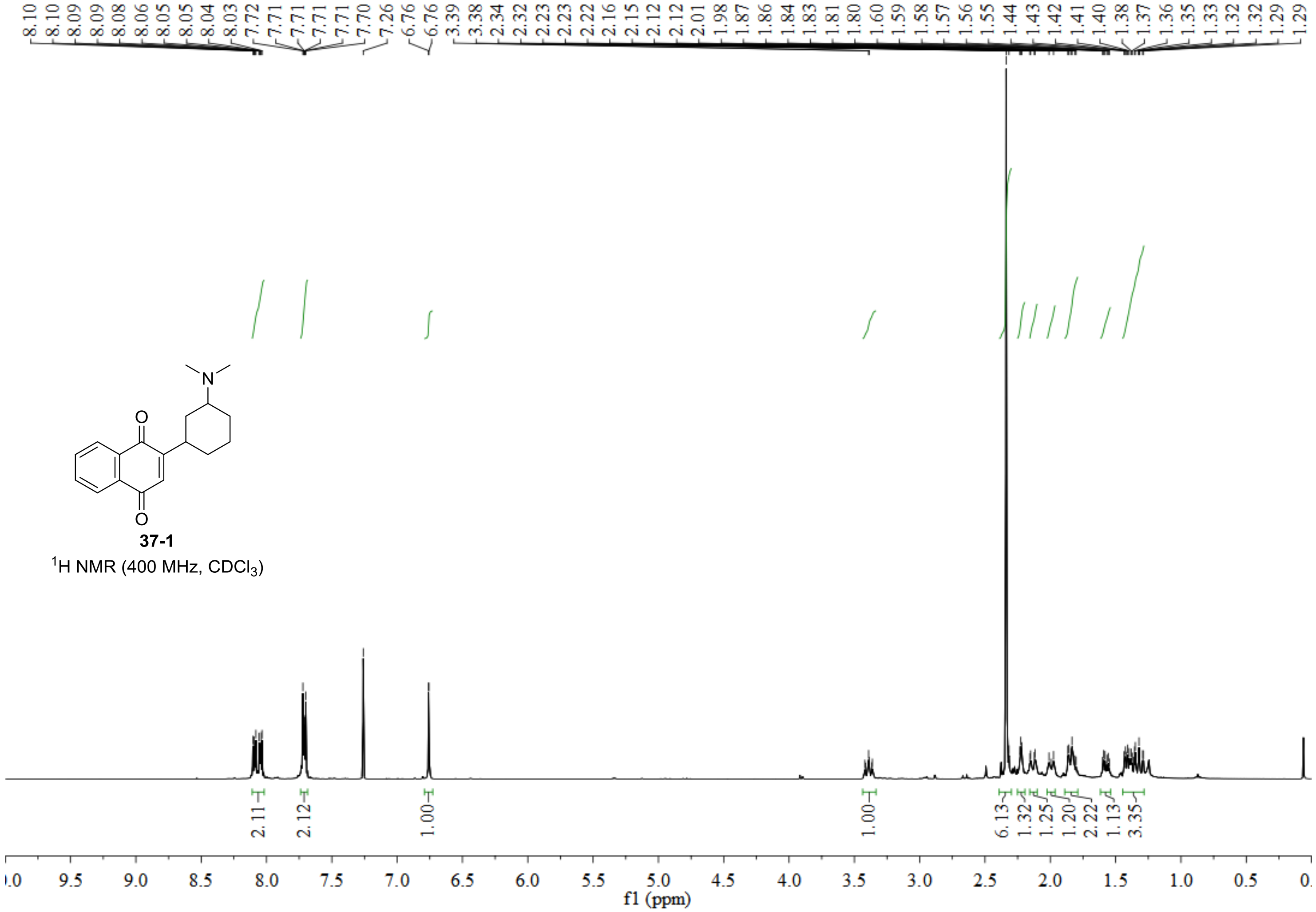

37-1

^{1}H NMR (400 MHz, $CDCl_3$)

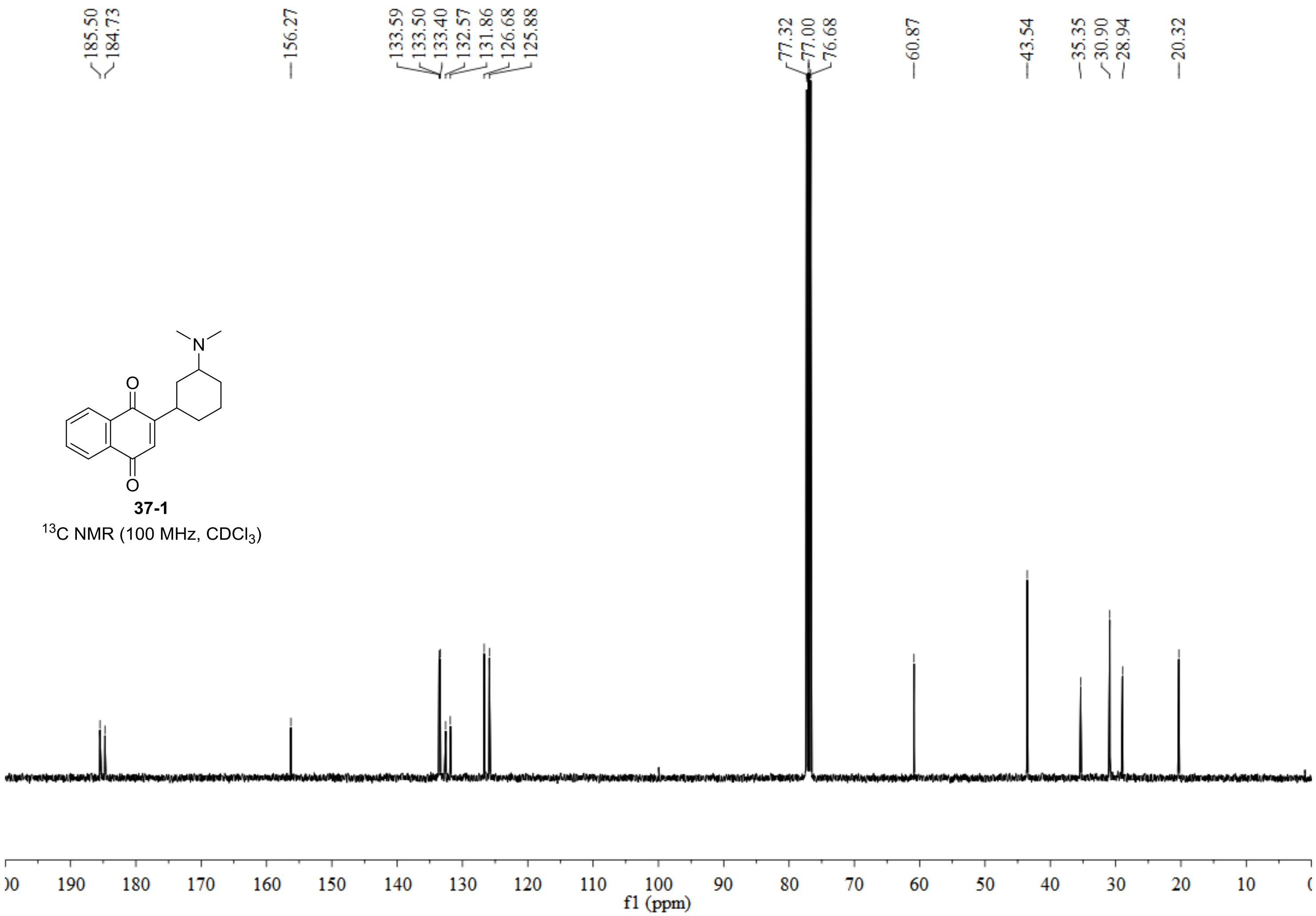

37-1

^{13}C NMR (100 MHz, $CDCl_3$)

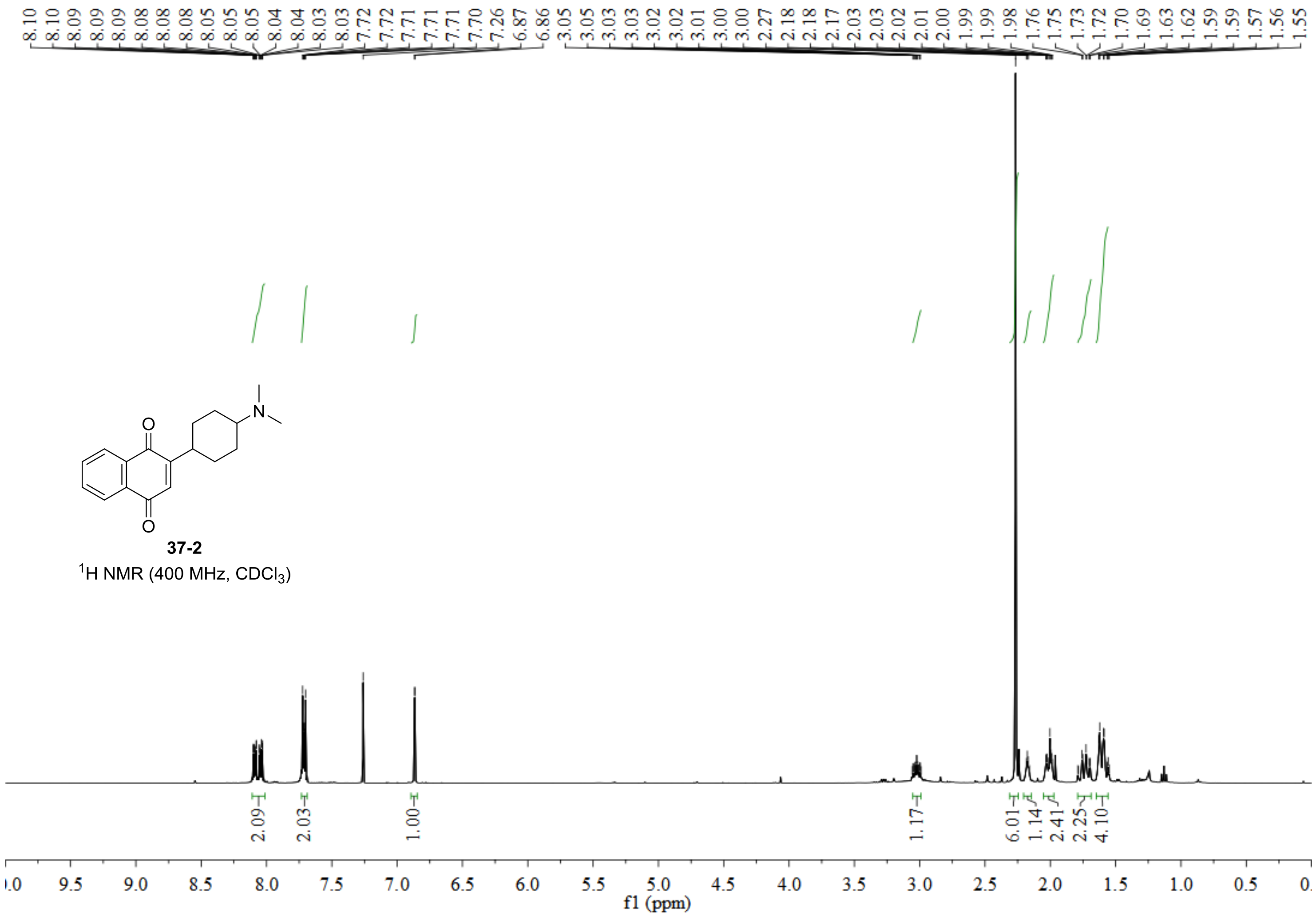
37-2
^{1}H NMR (400 MHz, $CDCl_3$)
8.10 8.10 8.09 8.09 8.09 8.08 8.08 8.08 8.05 8.05 8.05 8.04 8.04 8.03 8.03 7.72 7.72 7.71 7.71 7.71 7.70 7.26 6.87 6.86 3.05 3.05 3.03 3.03 3.02 3.02 3.01 3.00 3.00 2.27 2.18 2.18 2.17 2.03 2.03 2.02 2.01 2.00 1.99 1.99 1.98 1.76 1.75 1.73 1.72 1.70 1.69 1.63 1.62 1.59 1.59 1.57 1.56 1.55
2.09 2.03 1.00 1.17 6.01 1.14 2.41 2.25 4.10
f1 (ppm)

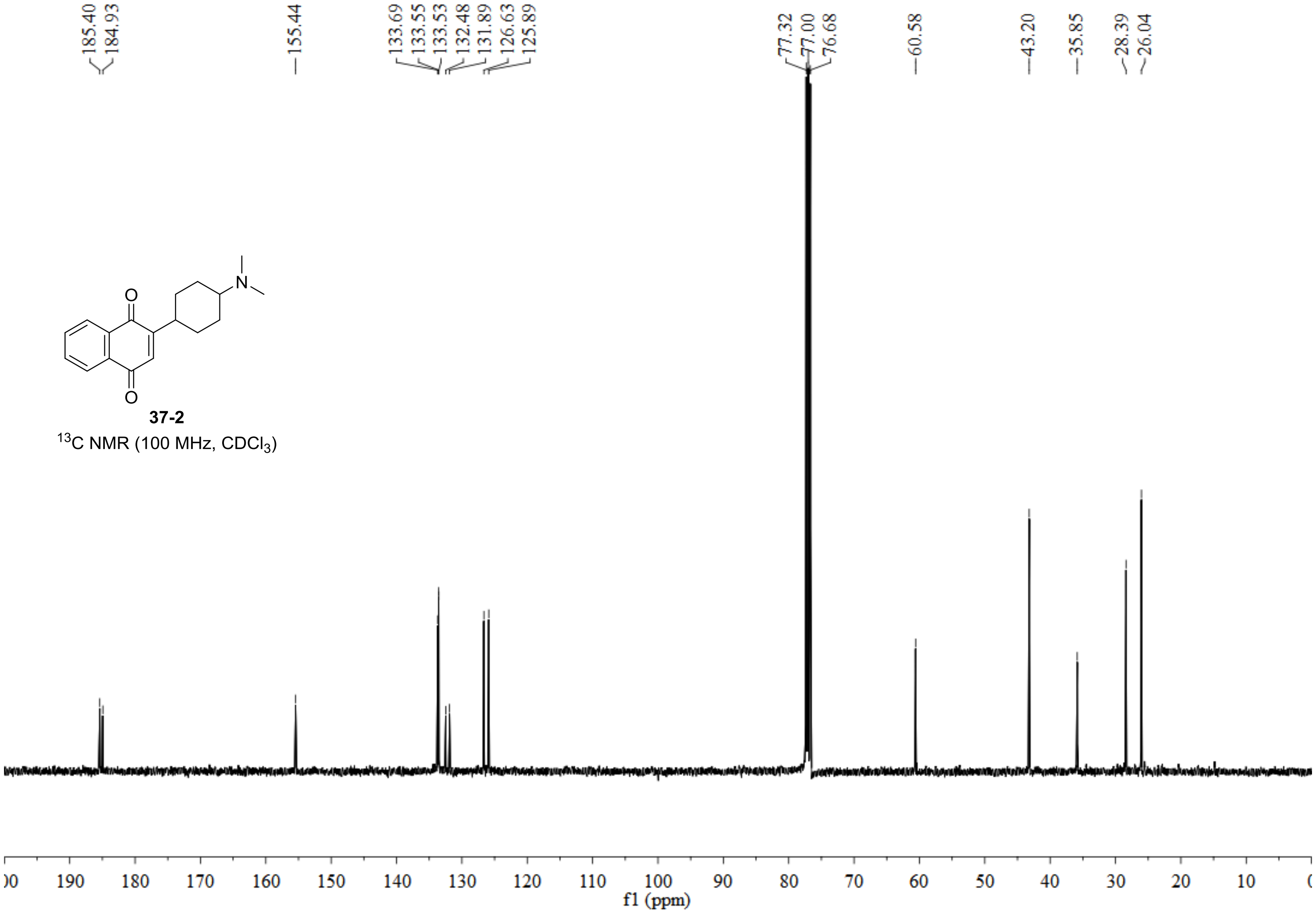

37-2

^{13}C NMR (100 MHz, $CDCl_3$)

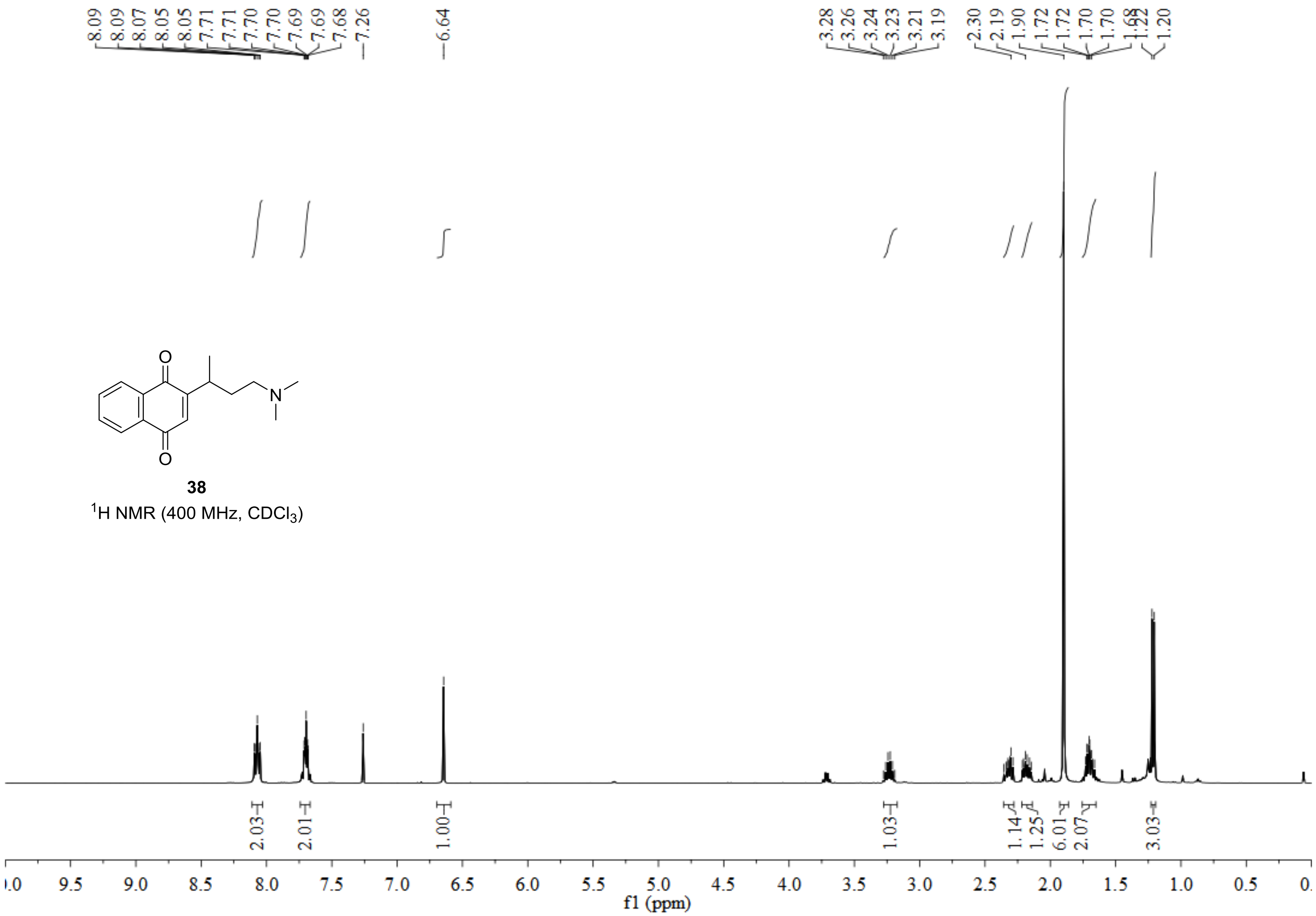

38

^1H NMR (400 MHz, $CDCl_3$)

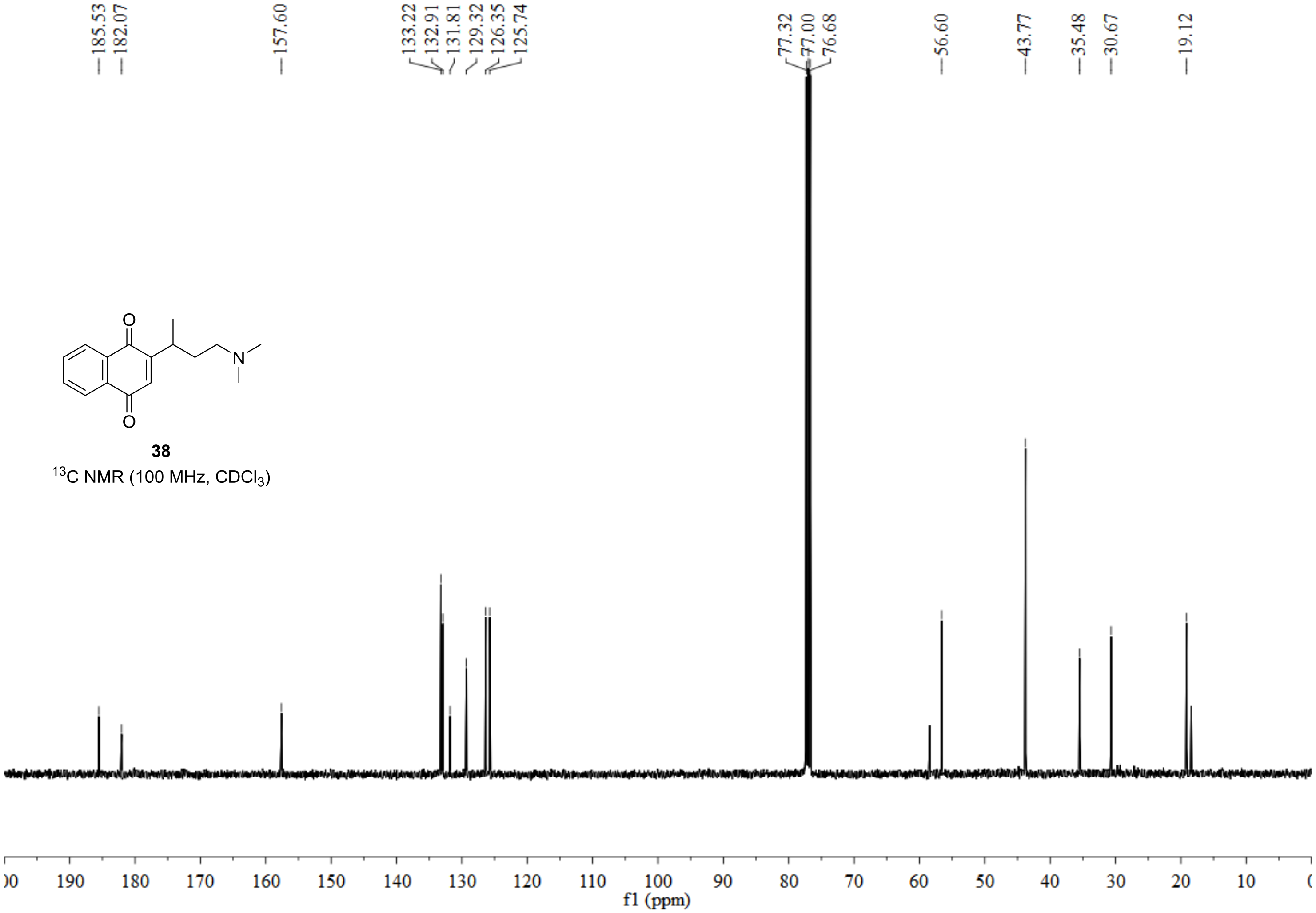

38

^{13}C NMR (100 MHz, $CDCl_3$)

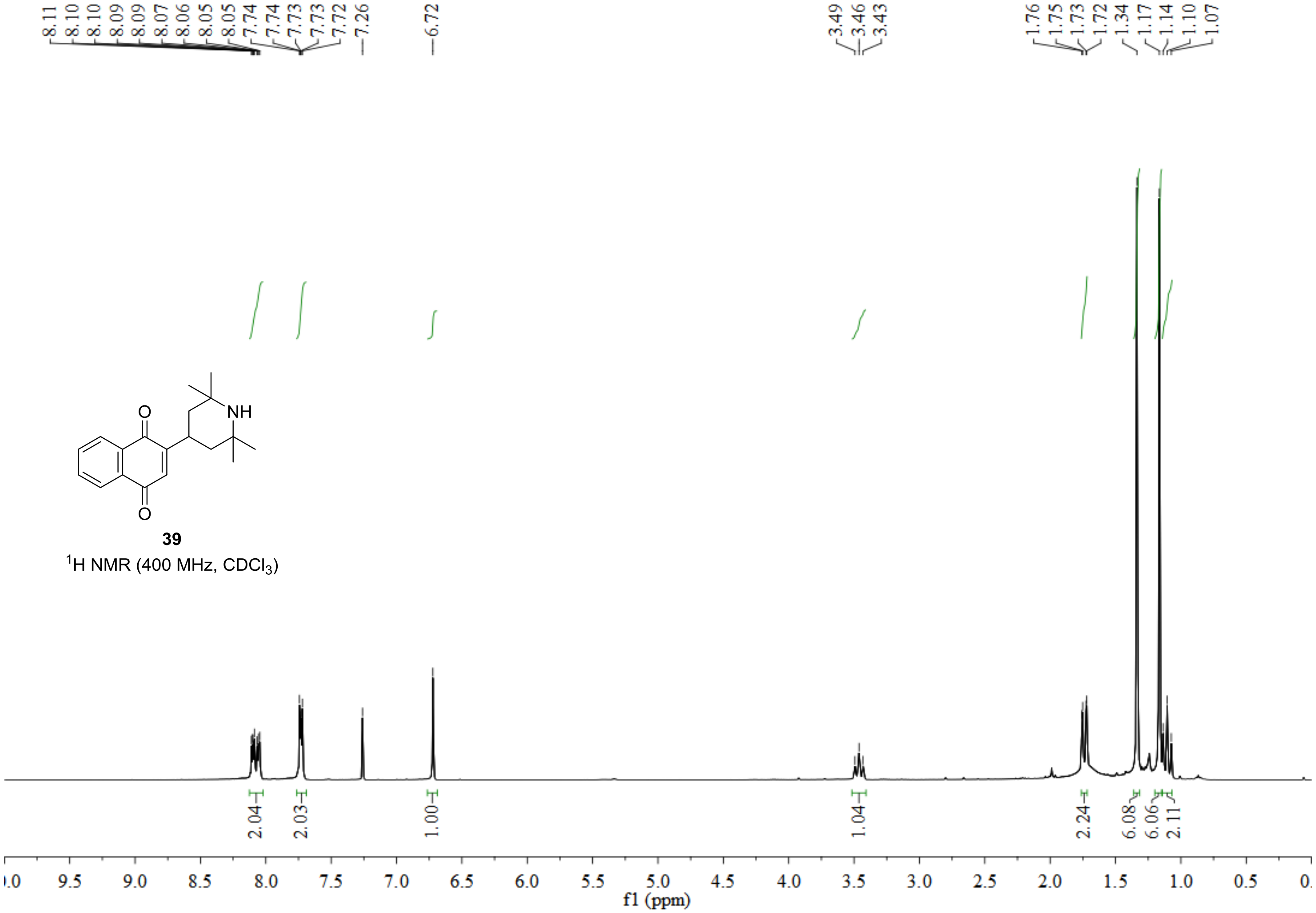
8.11
8.10
8.10
8.09
8.09
8.07
8.06
8.05
8.05
7.74
7.74
7.73
7.73
7.72
7.26
6.72
3.49
3.46
3.43
1.76
1.75
1.73
1.72
1.34
1.17
1.14
1.10
1.07
NH
O
O
39
1H NMR (400 MHz, CDCl3)
2.04
2.03
1.00
1.04
2.24
6.08
6.06
2.11
9.5
9.0
8.5
8.0
7.5
7.0
6.5
6.0
5.5
5.0
4.5
4.0
3.5
3.0
2.5
2.0
1.5
1.0
0.5
f1 (ppm)

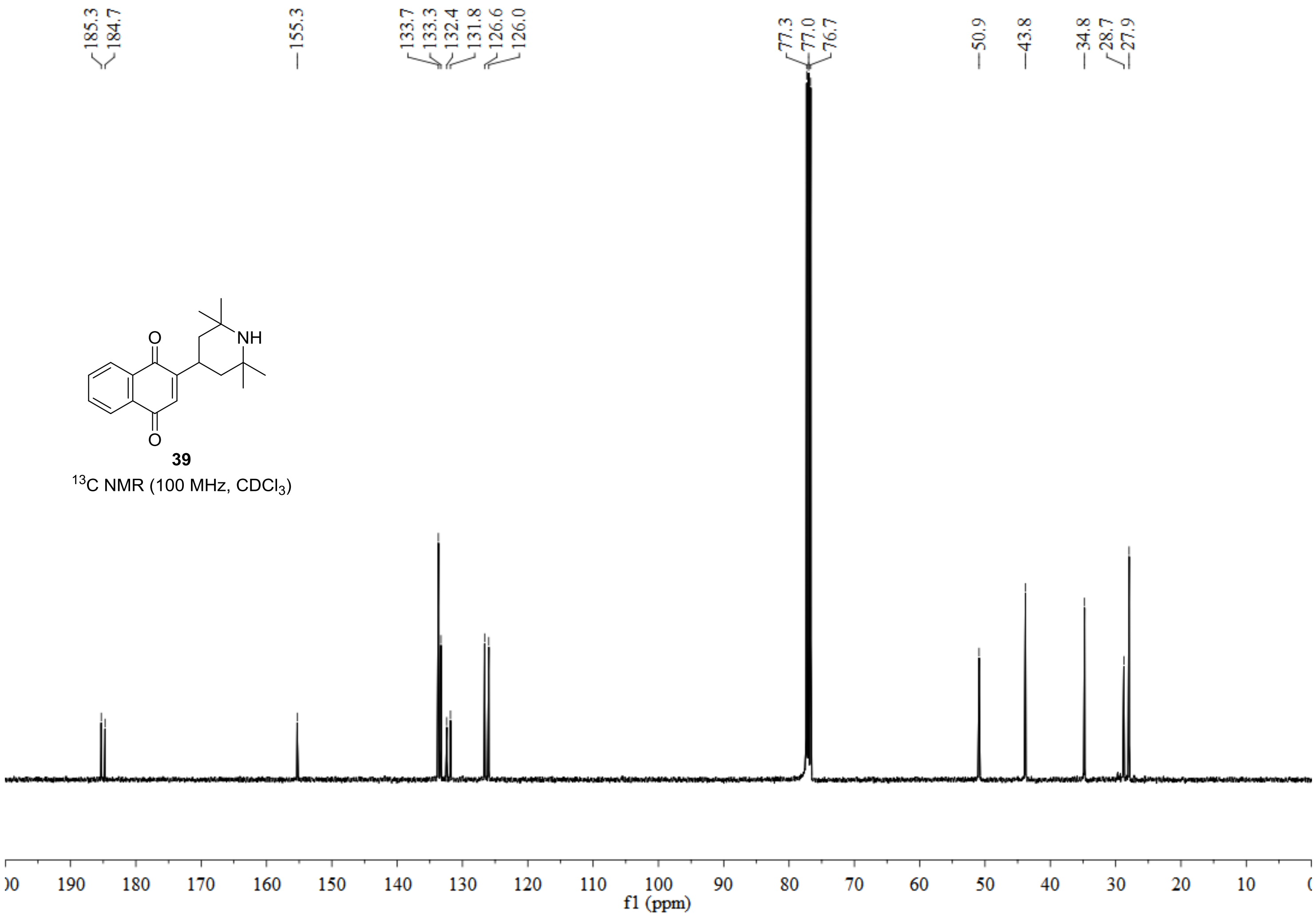

39

^{13}C NMR (100 MHz, $CDCl_3$)

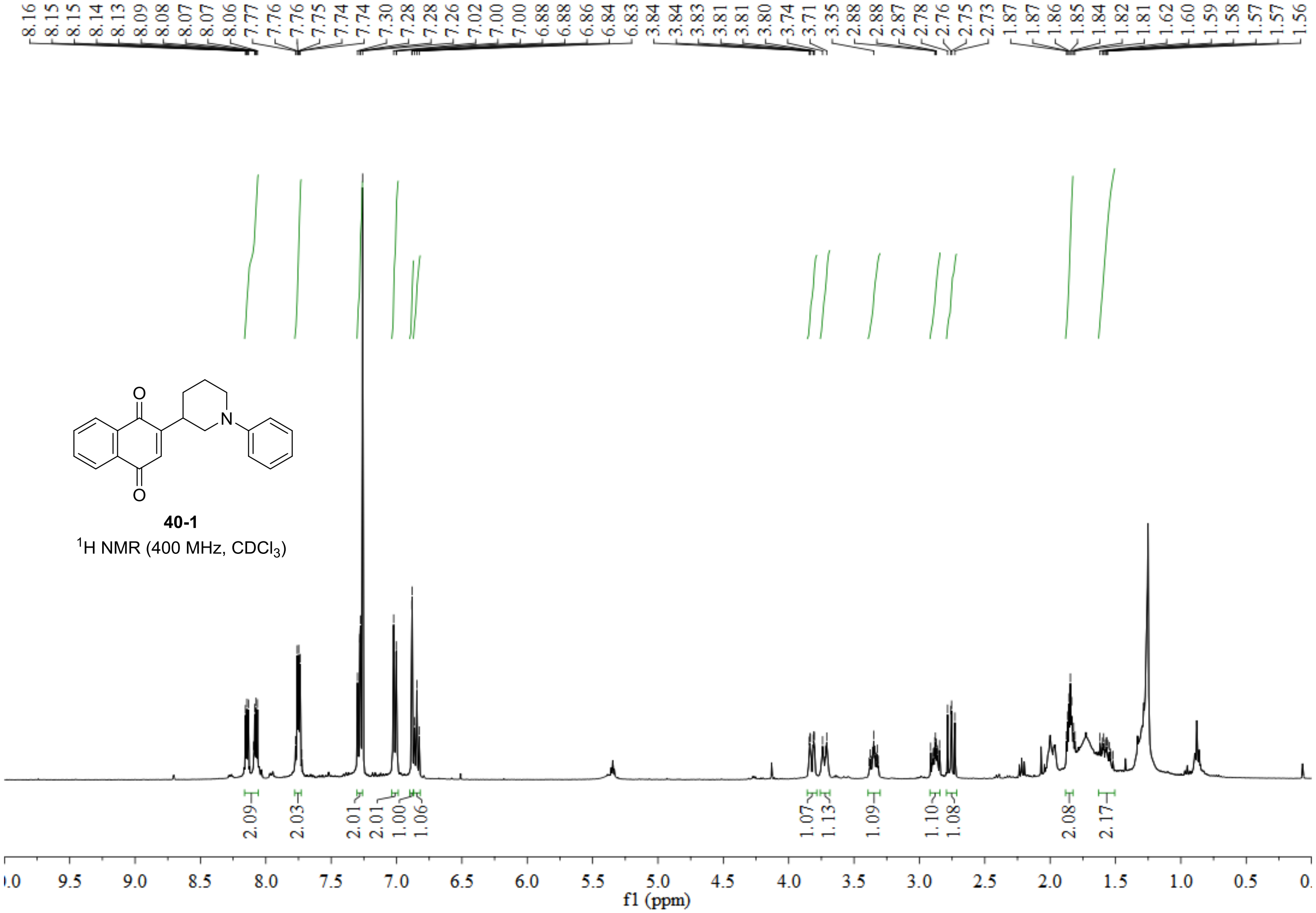

8.16 8.15 8.15 8.14 8.13 8.09 8.08 8.07 8.07 8.06 7.77 7.76 7.76 7.75 7.74 7.74 7.30 7.28 7.28 7.26 7.02 7.00 7.00 6.88 6.88 6.86 6.84 6.83 3.84 3.84 3.83 3.81 3.81 3.80 3.74 3.71 3.35 2.88 2.88 2.87 2.78 2.76 2.75 2.73 1.87 1.87 1.86 1.85 1.84 1.82 1.81 1.62 1.60 1.59 1.58 1.57 1.57 1.56
O
N
O
40-1
1H NMR (400 MHz, CDCl3)
2.09
2.03
2.01
2.01
1.00
1.06
1.07
1.13
1.09
1.10
1.08
2.08
2.17
f1 (ppm)

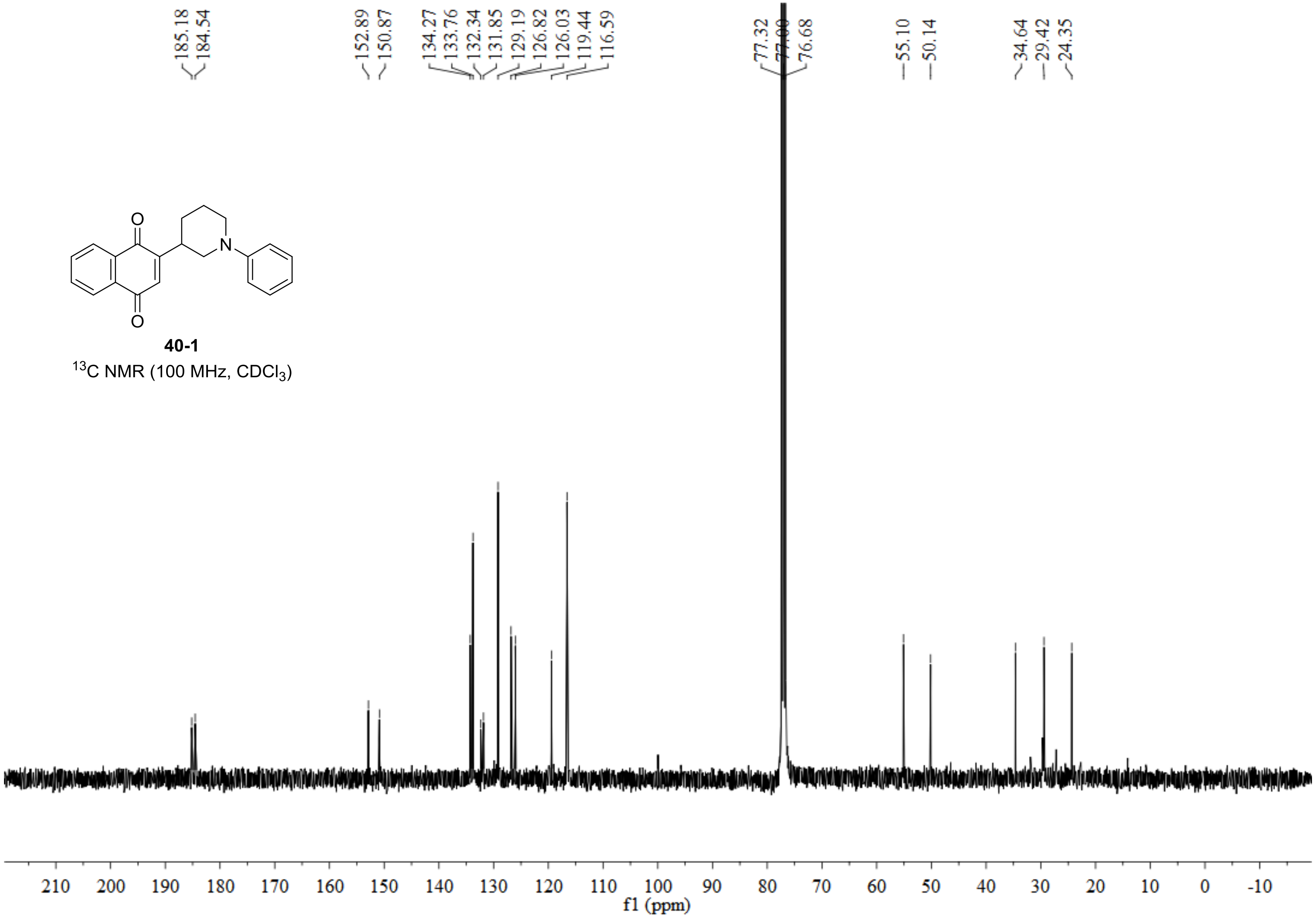

40-1

^{13}C NMR (100 MHz, $CDCl_3$)

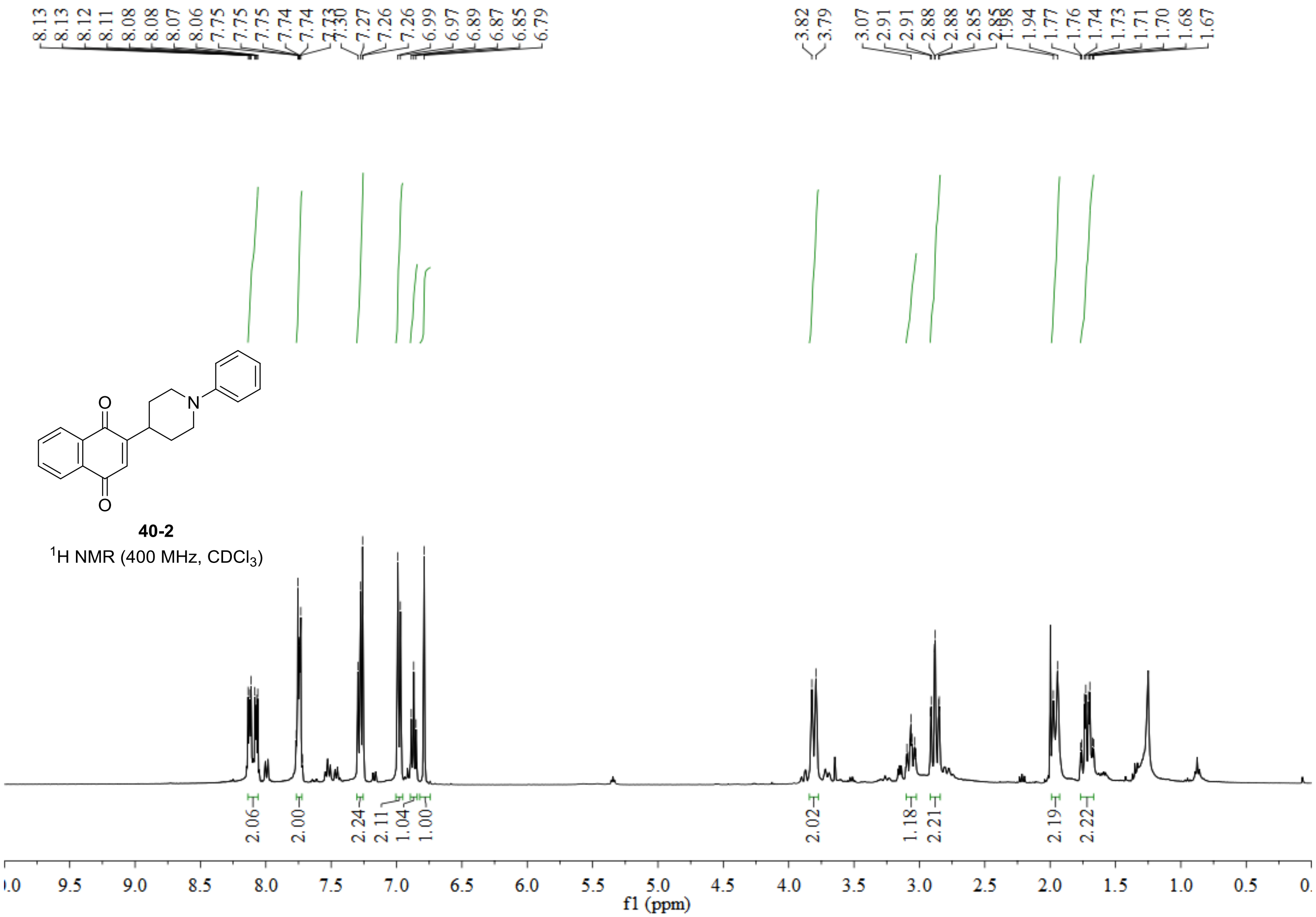

40-2

^{1}H NMR (400 MHz, $CDCl_3$)

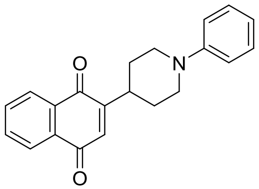

40-2

^{13}C NMR (100 MHz, $CDCl_3$)

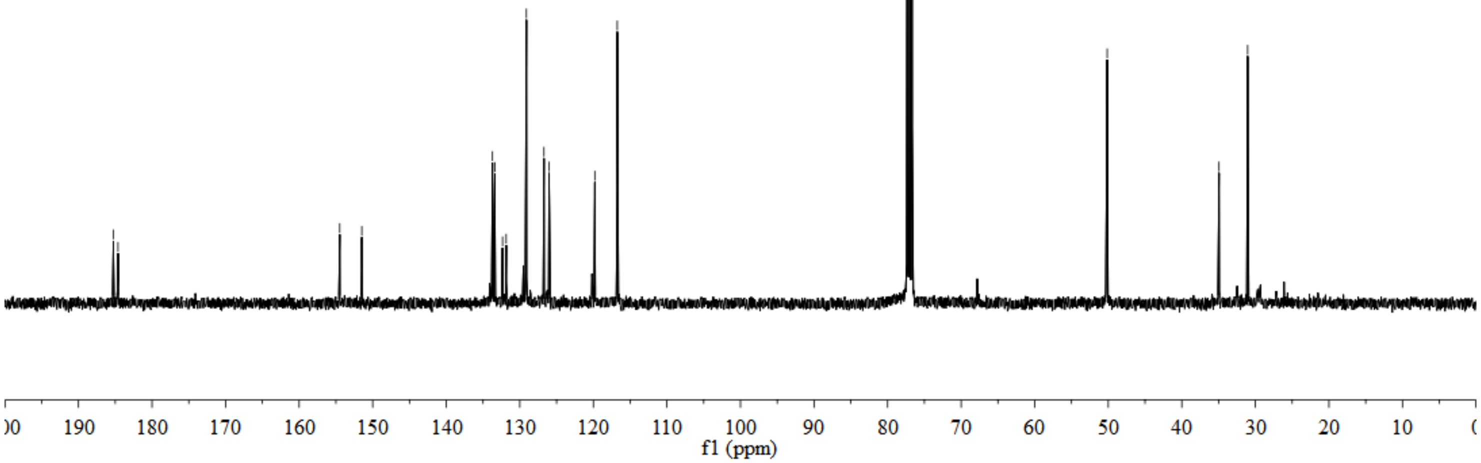

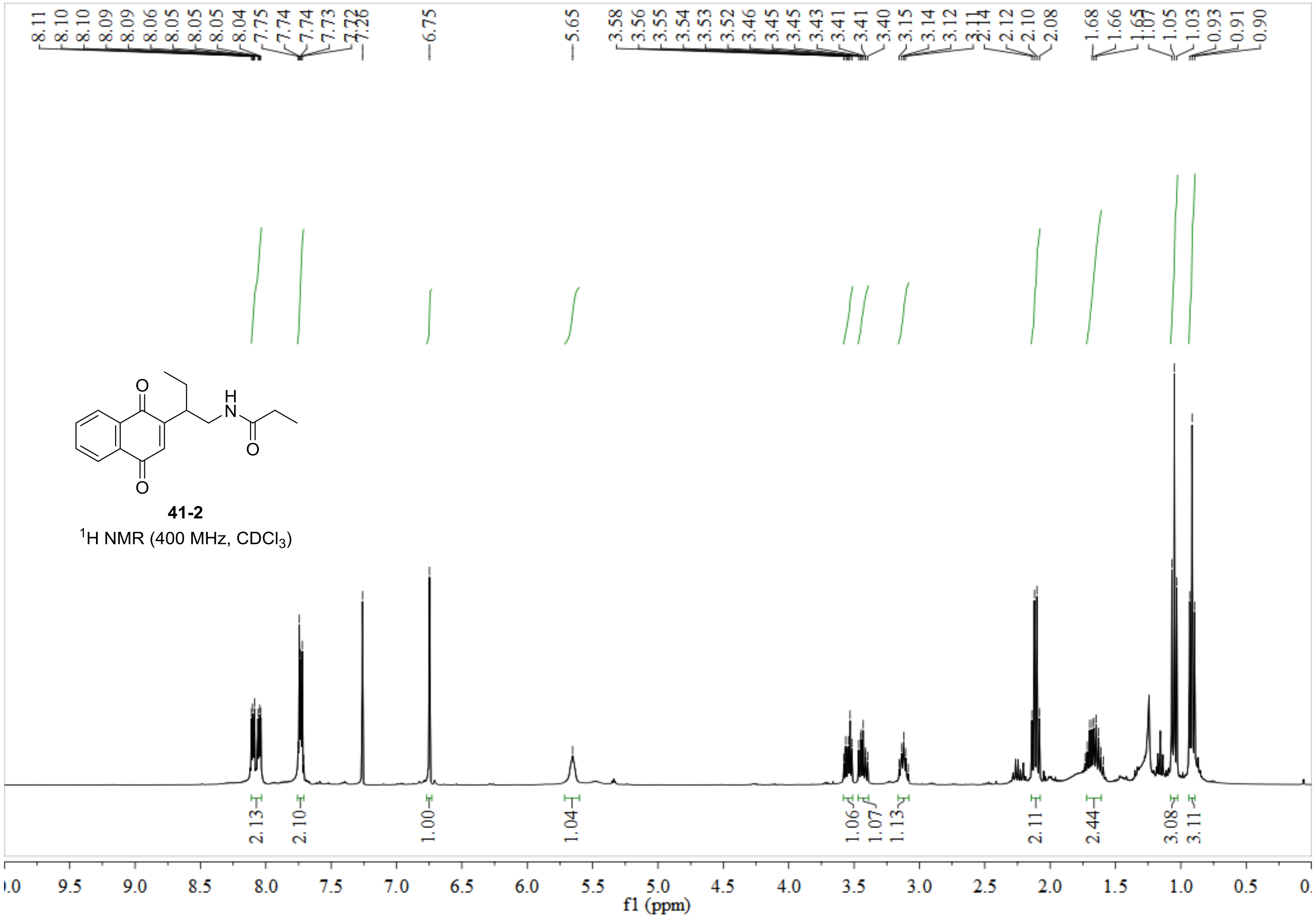
8.11 8.10 8.10 8.09 8.09 8.06 8.05 8.05 8.05 8.04 7.75 7.74 7.74 7.73 7.72 7.26
6.75
5.65
3.58 3.56 3.55 3.54 3.53 3.52 3.46 3.45 3.45 3.43 3.41 3.41 3.40 3.15 3.14 3.12 3.11 2.14 2.12 2.10 2.08
1.68 1.66 1.65 1.07 1.05 1.03 0.93 0.91 0.90
O
H
N
O
O
41-2
1H NMR (400 MHz, CDCl3)
2.13
2.10
1.00
1.04
1.06
1.07
1.13
2.11
2.44
3.08
3.11
9.5 9.0 8.5 8.0 7.5 7.0 6.5 6.0 5.5 5.0 4.5 4.0 3.5 3.0 2.5 2.0 1.5 1.0 0.5
f1 (ppm)

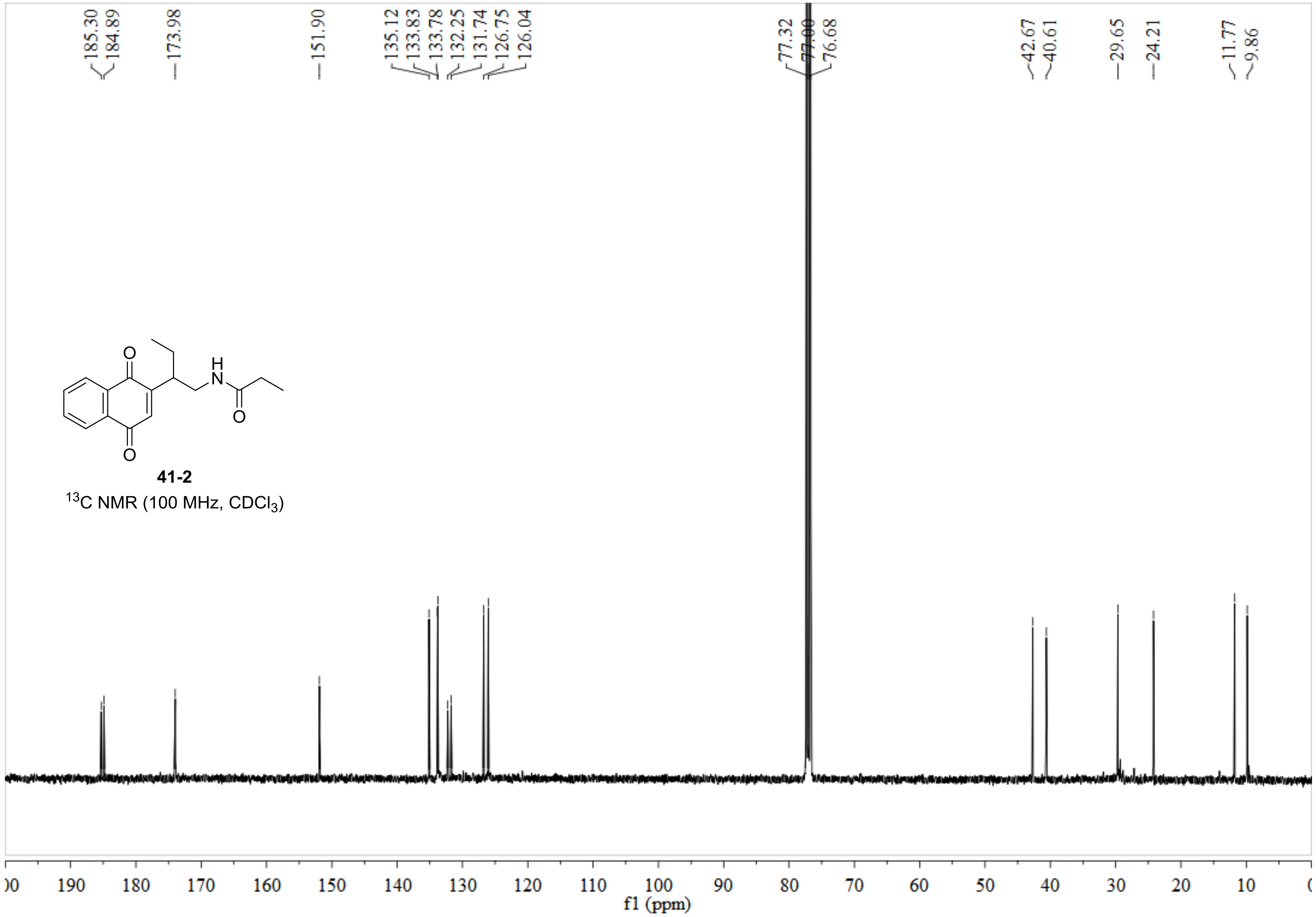
185.30
184.89
173.98
151.90
135.12
133.83
133.78
132.25
131.74
126.75
126.04
77.32
77.00
76.68
42.67
40.61
29.65
24.21
11.77
9.86
O
H
N
O
O
41-2
13C NMR (100 MHz, CDCl3)
00
190
180
170
160
150
140
130
120
110
100
90
80
70
60
50
40
30
20
10
f1 (ppm)

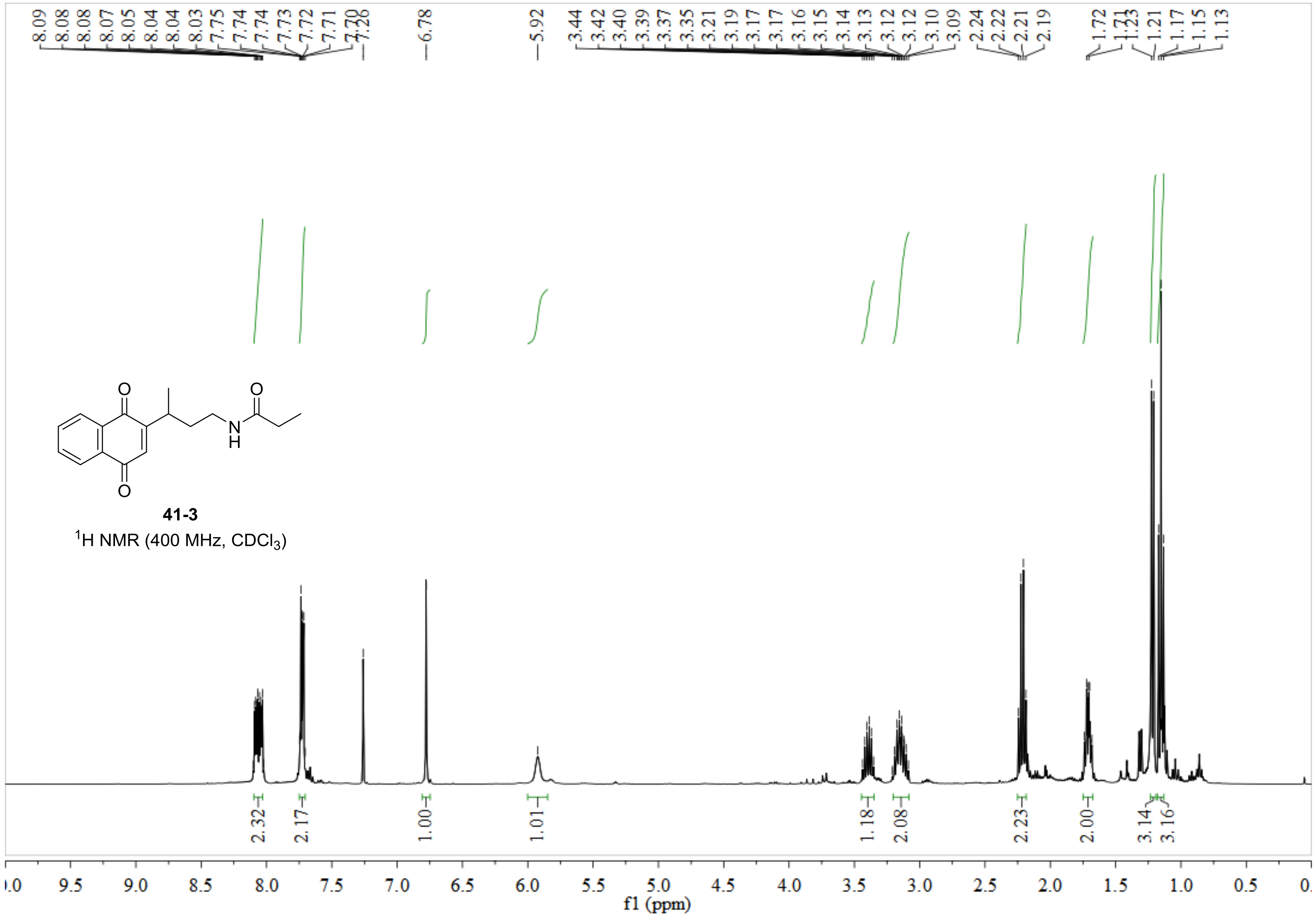
41-3
1H NMR (400 MHz, CDCl3)
f1 (ppm)

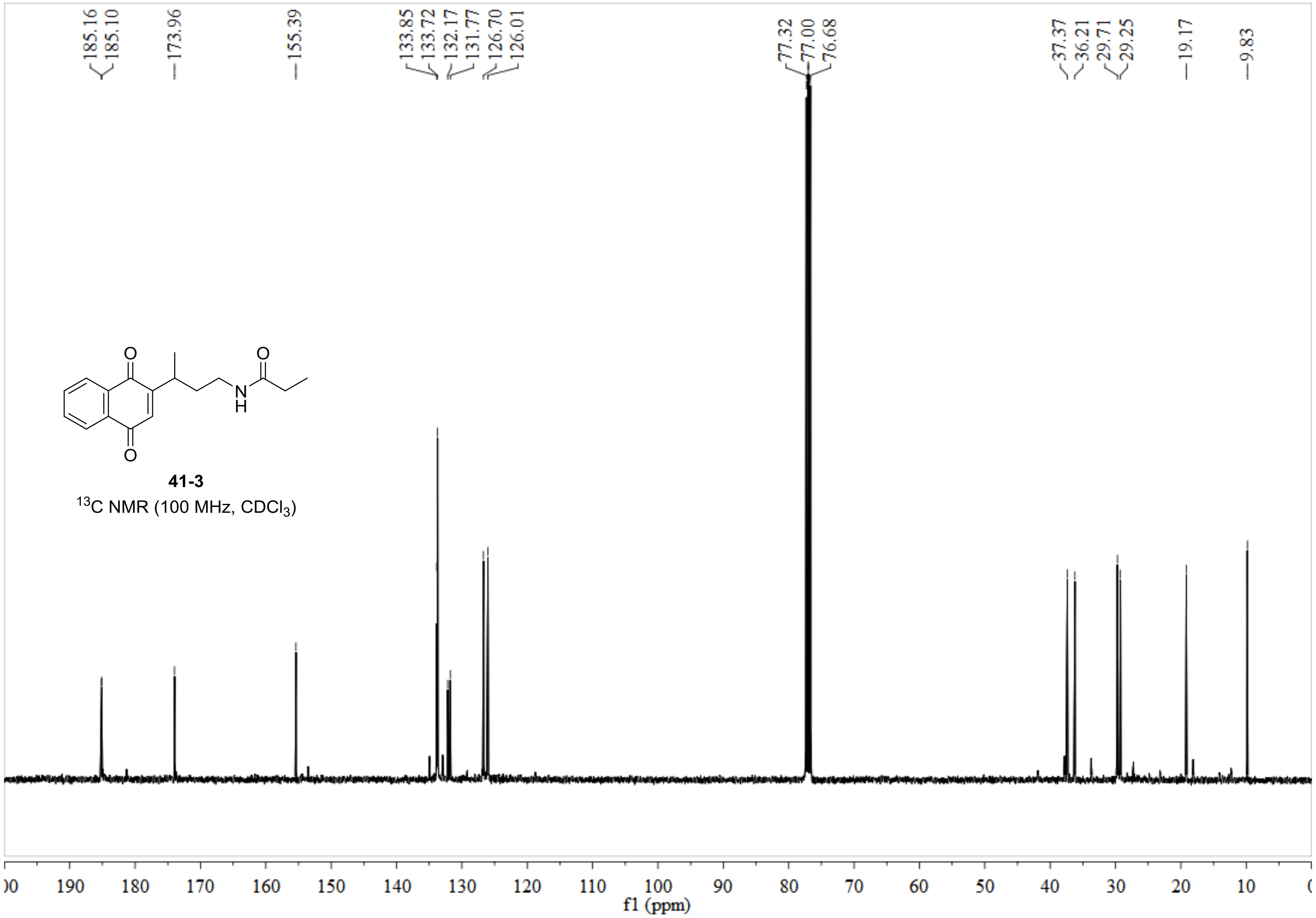
185.16
185.10
173.96
155.39
133.85
133.72
132.17
131.77
126.70
126.01
77.32
77.00
76.68
37.37
36.21
29.71
29.25
19.17
9.83
O
O
O
N
H
41-3
^{13}C NMR (100 MHz, $CDCl_3$)
190
180
170
160
150
140
130
120
110
100
90
80
70
60
50
40
30
20
10
f1 (ppm)

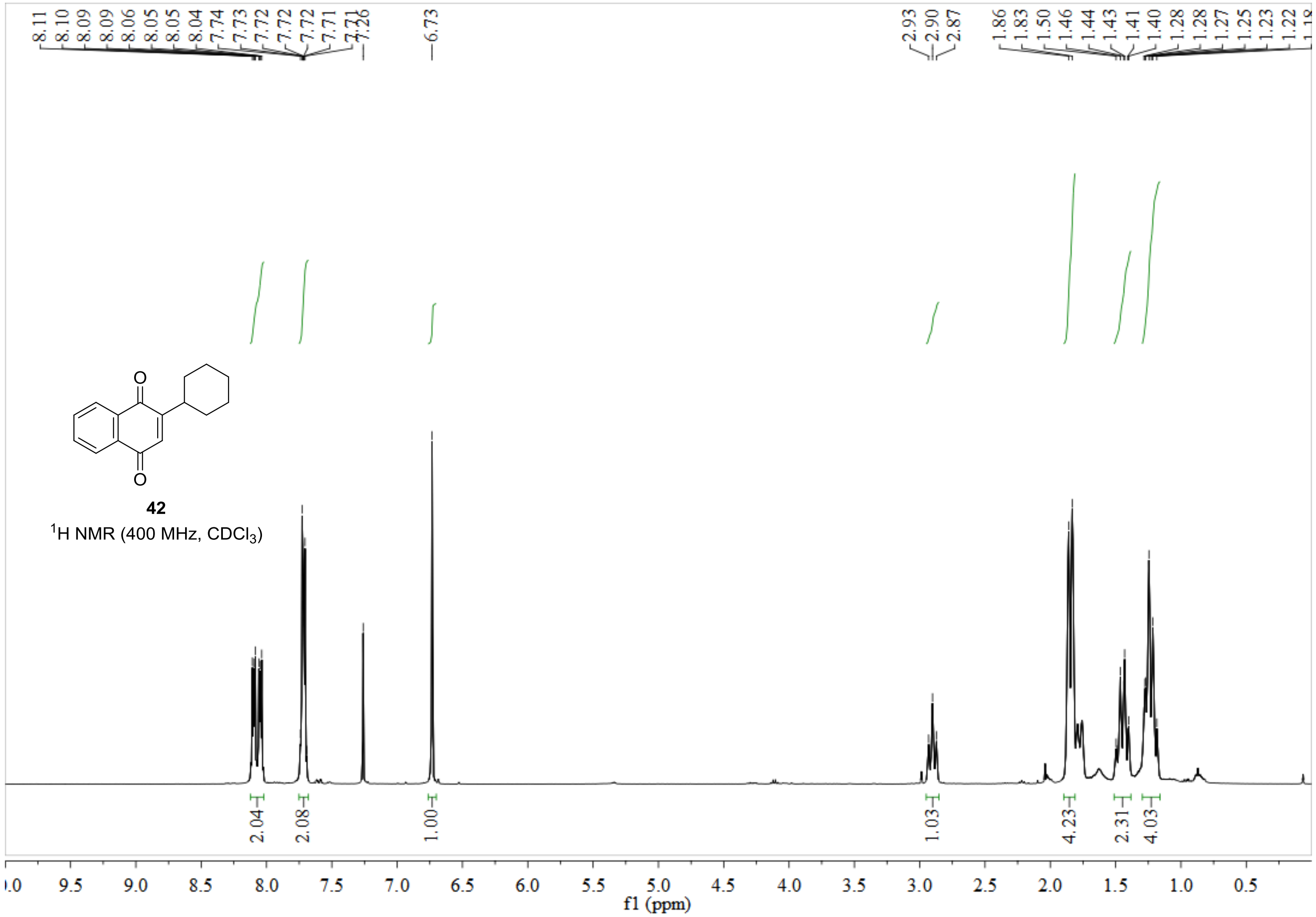

42

^{1}H NMR (400 MHz, $CDCl_3$)

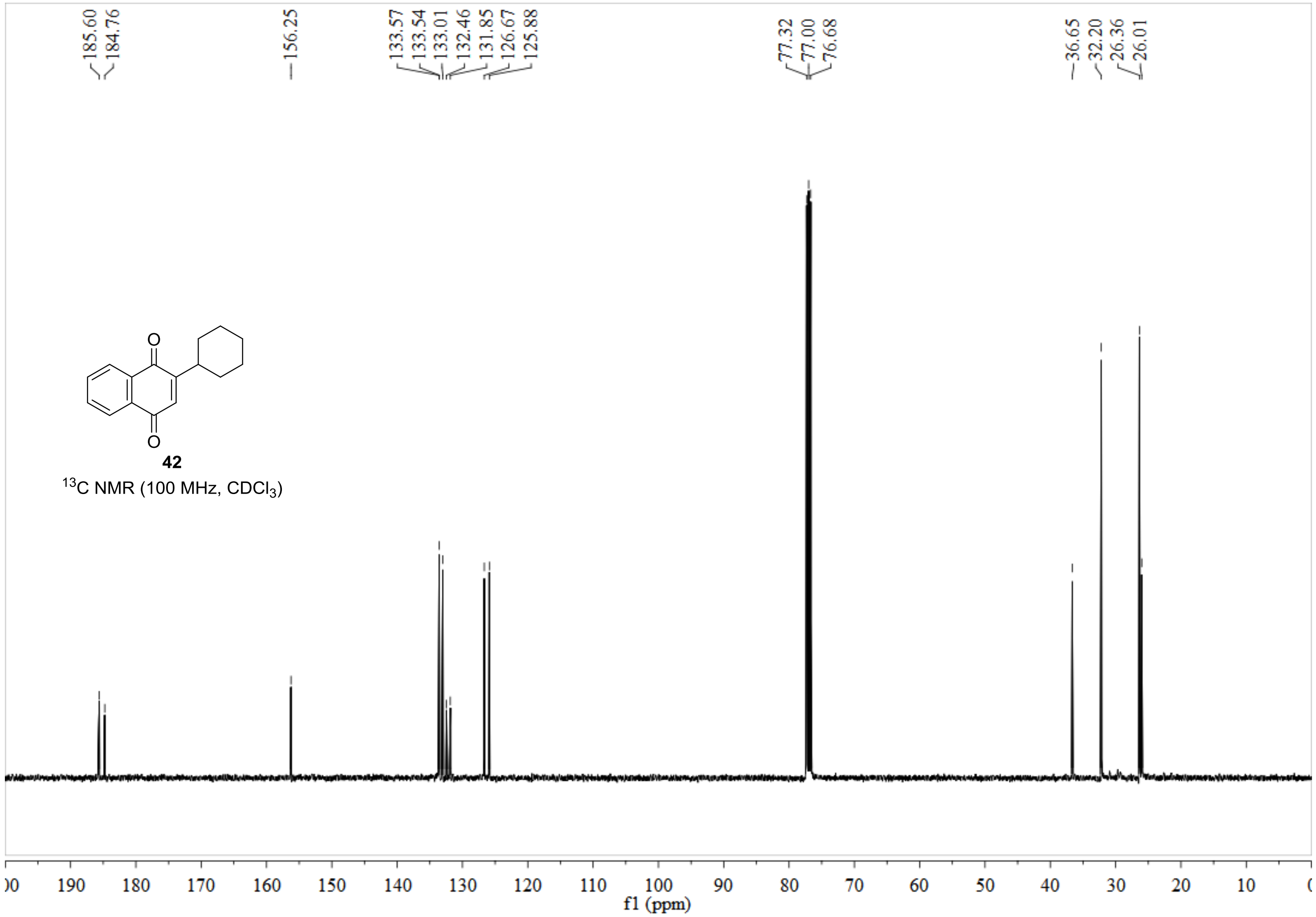

185.60
184.76
156.25
133.57
133.54
133.01
132.46
131.85
126.67
125.88
77.32
77.00
76.68
36.65
32.20
26.36
26.01
42
^{13}C NMR (100 MHz, CDCl$_3$)
190
180
170
160
150
140
130
120
110
100
90
80
70
60
50
40
30
20
10
f1 (ppm)

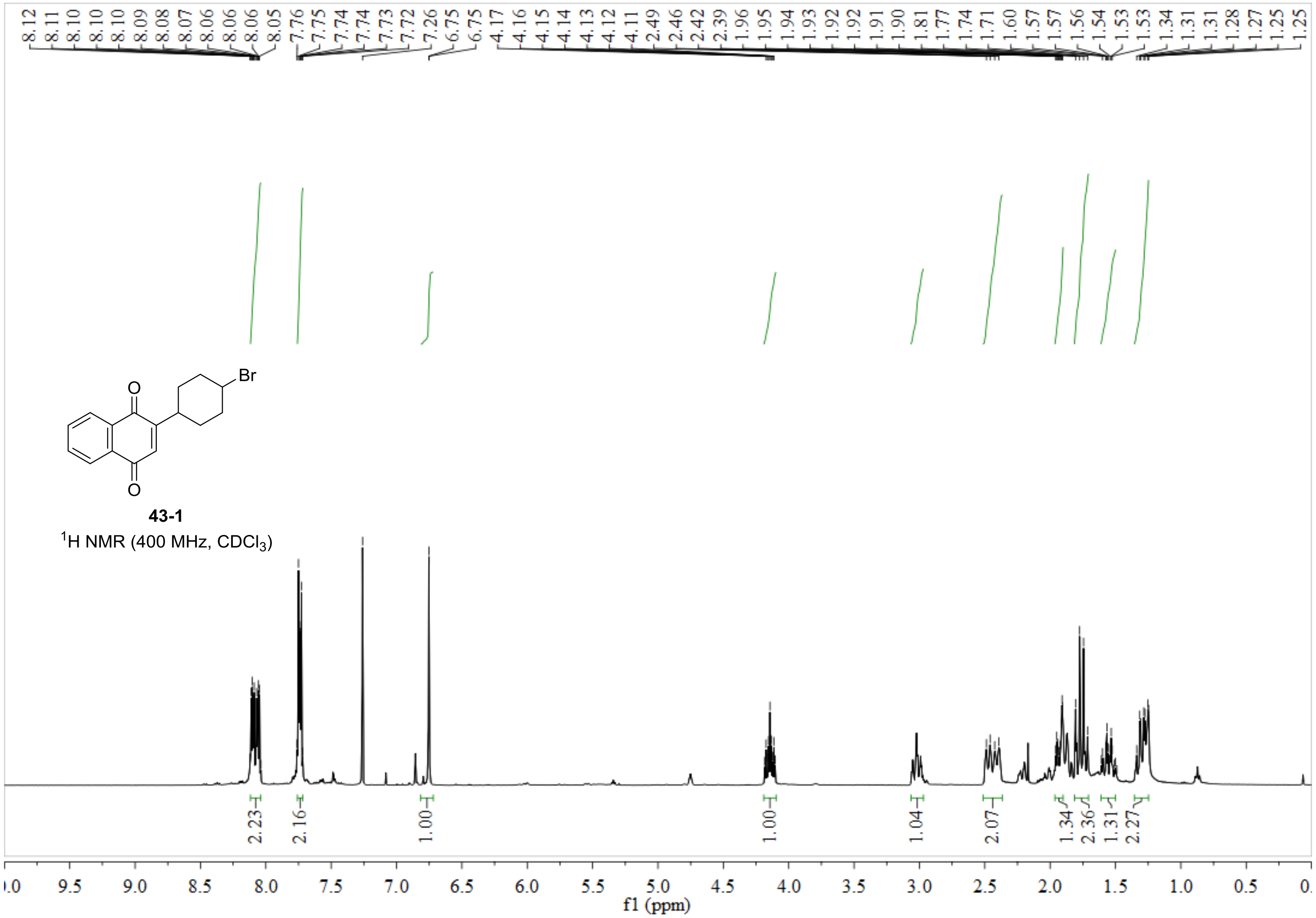

43-1
1H NMR (400 MHz, CDCl3)
f1 (ppm)

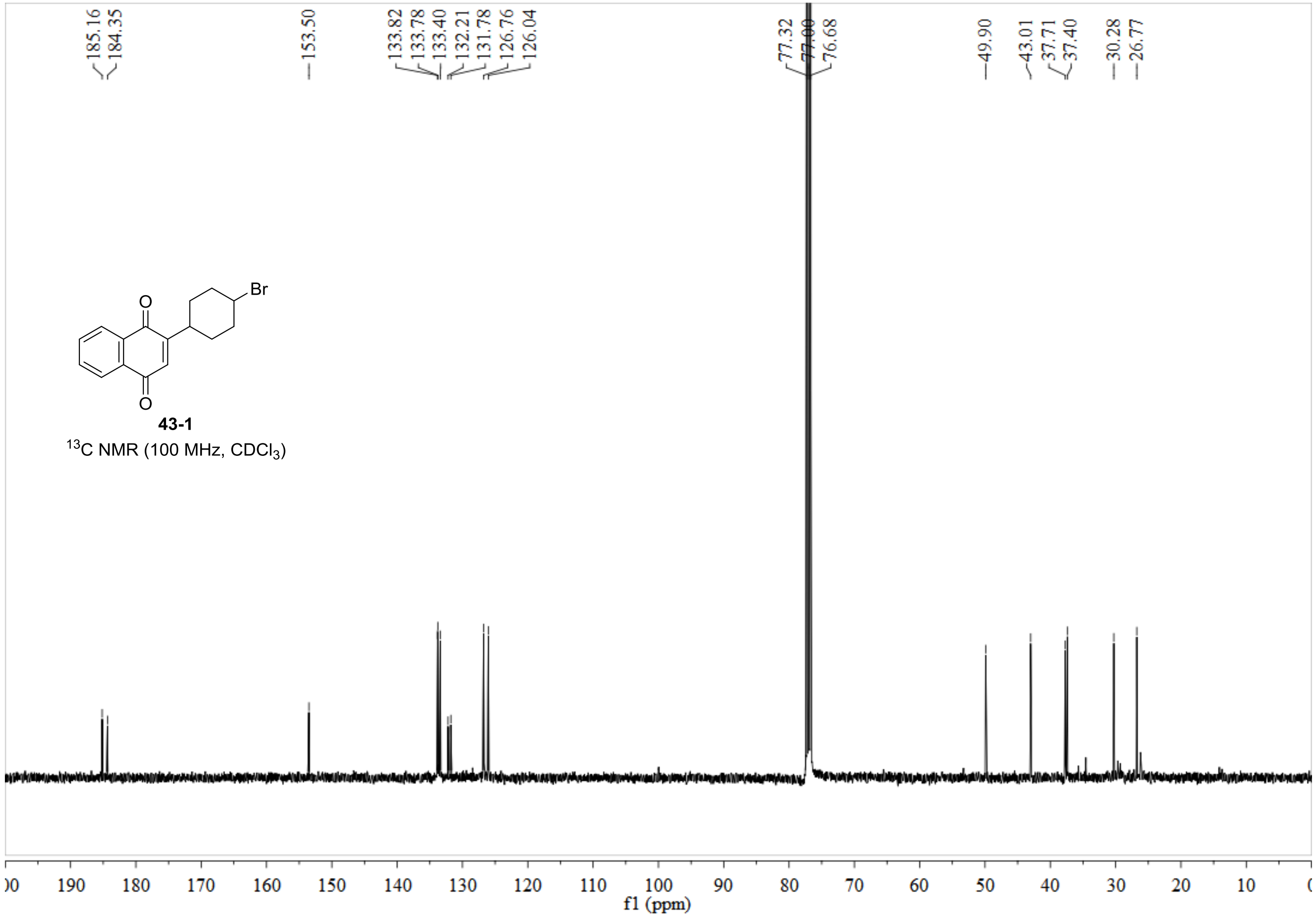
185.16
184.35
153.50
133.82
133.78
133.40
132.21
131.78
126.76
126.04
77.32
77.00
76.68
49.90
43.01
37.71
37.40
30.28
26.77
Br
O
O
43-1
^{13}C NMR (100 MHz, $CDCl_3$)
190 180 170 160 150 140 130 120 110 100 90 80 70 60 50 40 30 20 10
f1 (ppm)

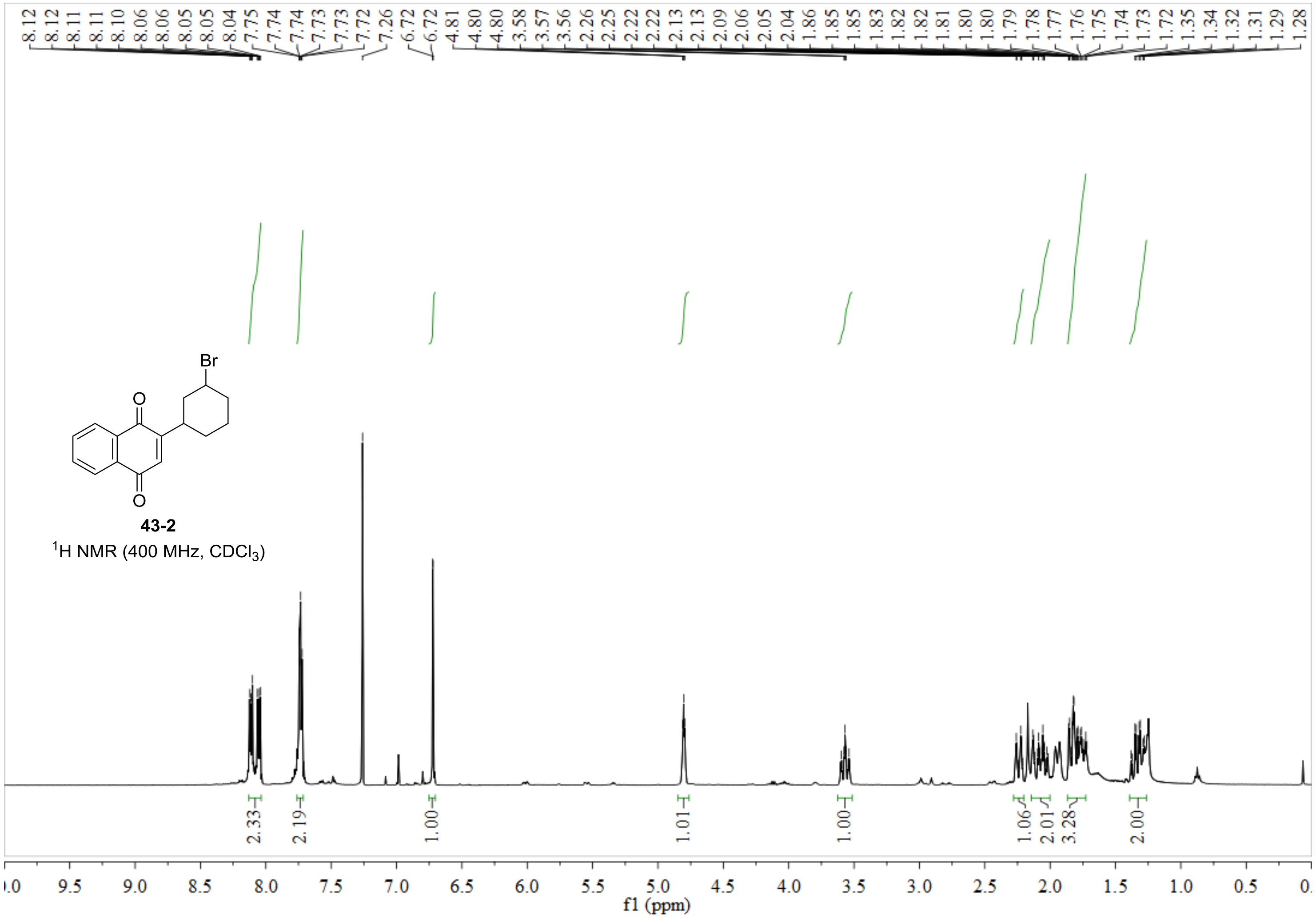

Br
O
O
43-2
^{1}H NMR (400 MHz, CDCl$_3$)
f1 (ppm)

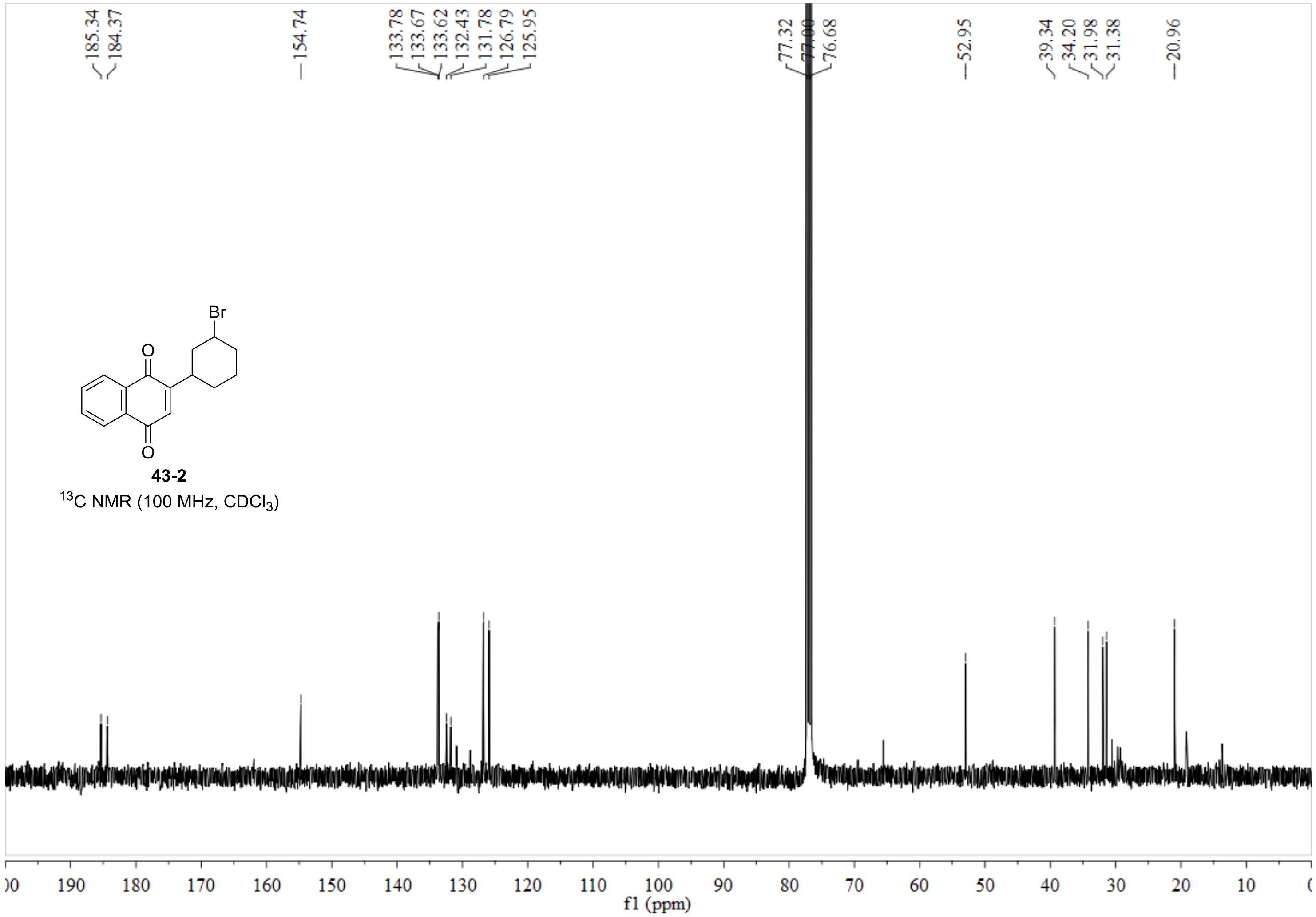

^{13}C NMR (100 MHz, $CDCl_3$)

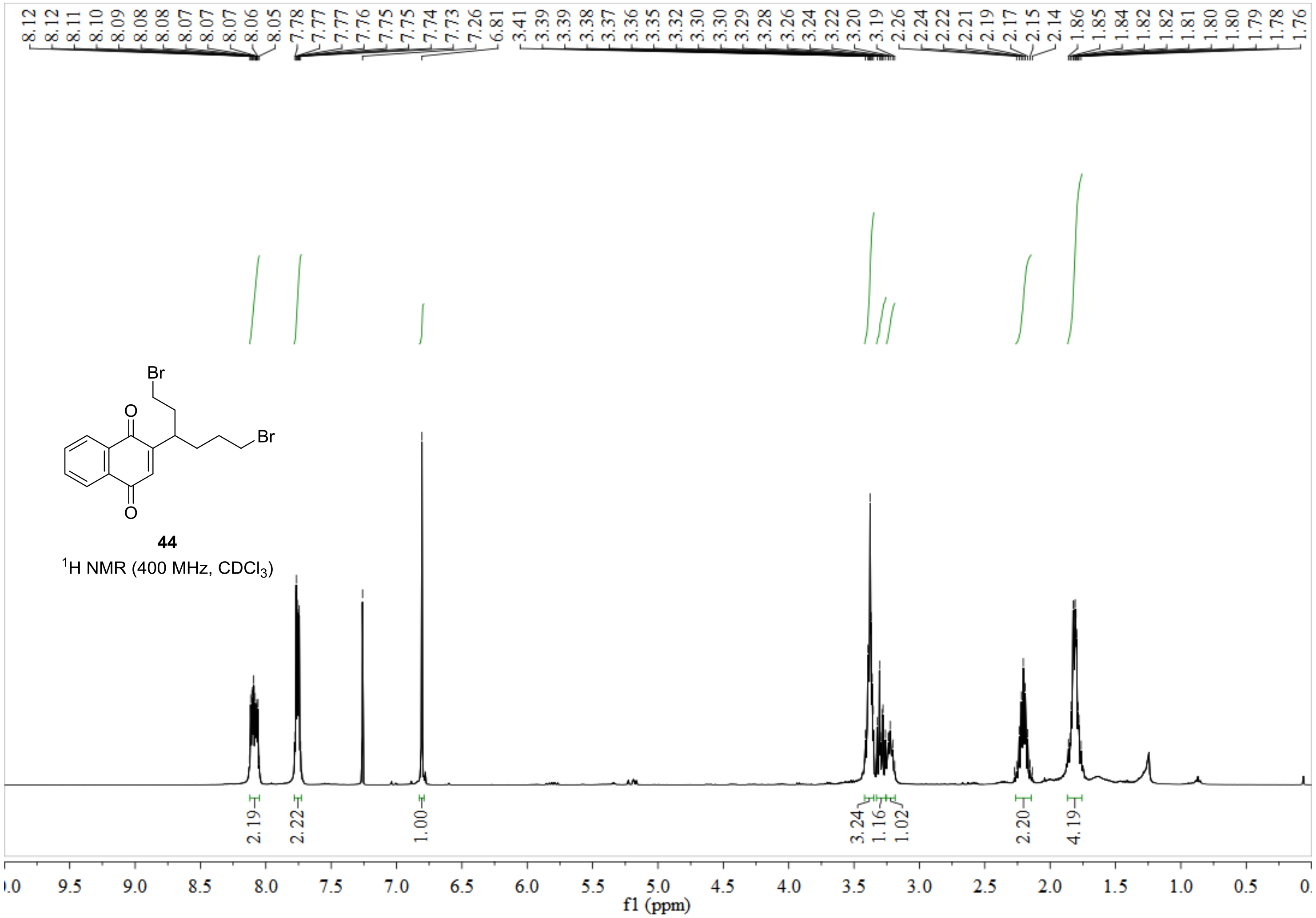
Br
O
Br
O
44
^{1}H NMR (400 MHz, CDCl$_3$)
8.12
8.12
8.11
8.10
8.09
8.08
8.08
8.07
8.07
8.07
8.06
8.05
7.78
7.77
7.77
7.76
7.75
7.75
7.74
7.73
7.26
6.81
3.41
3.39
3.39
3.38
3.37
3.36
3.35
3.32
3.30
3.30
3.29
3.28
3.26
3.24
3.22
3.20
3.19
2.26
2.24
2.24
2.22
2.21
2.19
2.17
2.15
2.14
1.86
1.85
1.84
1.82
1.82
1.81
1.80
1.80
1.79
1.78
1.76
2.19
2.22
1.00
3.24
1.16
1.02
2.20
4.19
f1 (ppm)

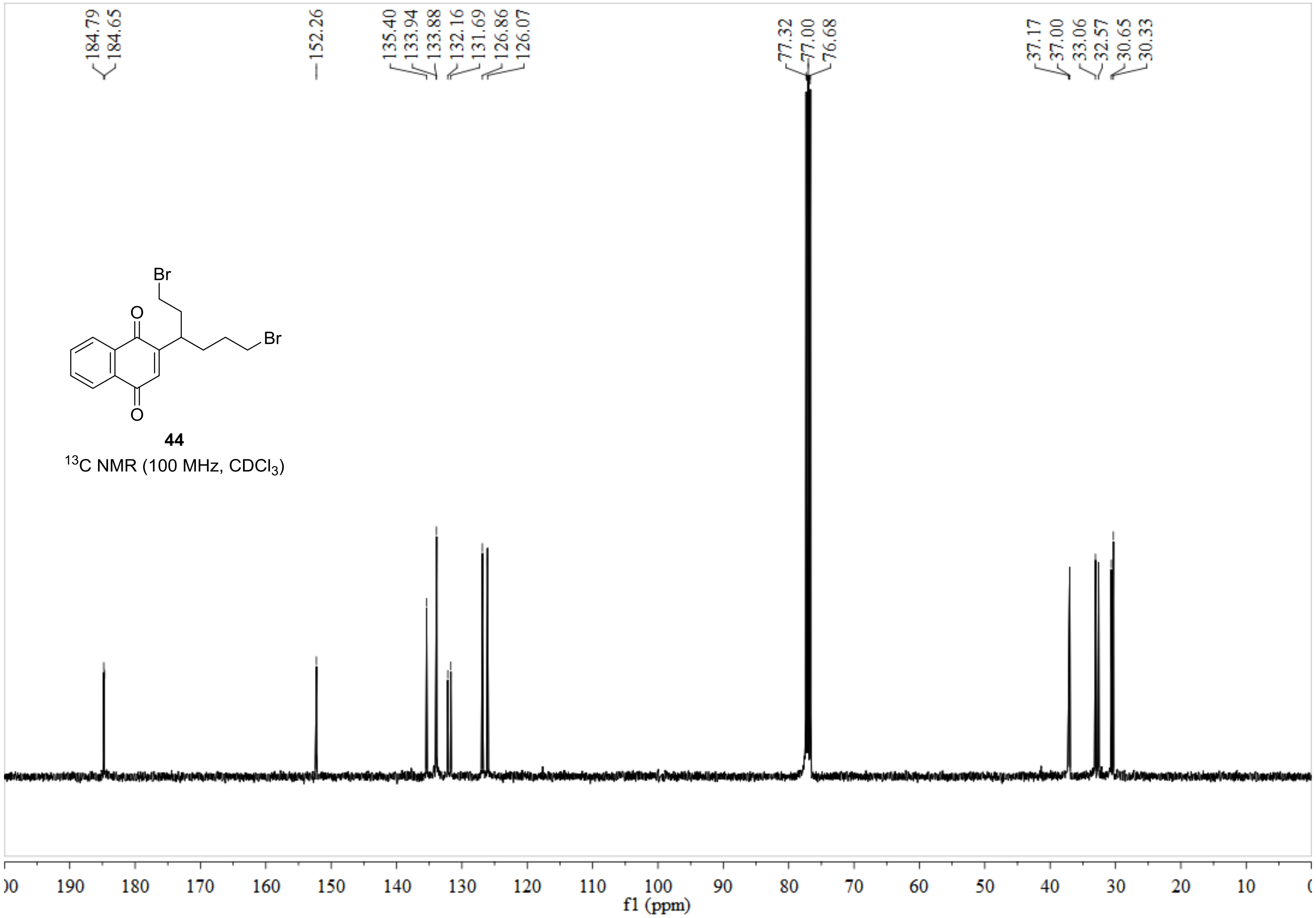
184.79
184.65
152.26
135.40
133.94
133.88
132.16
131.69
126.86
126.07
77.32
77.00
76.68
37.17
37.00
33.06
32.57
30.65
30.33
Br
O
Br
O
44
13C NMR (100 MHz, CDCl3)
f1 (ppm)

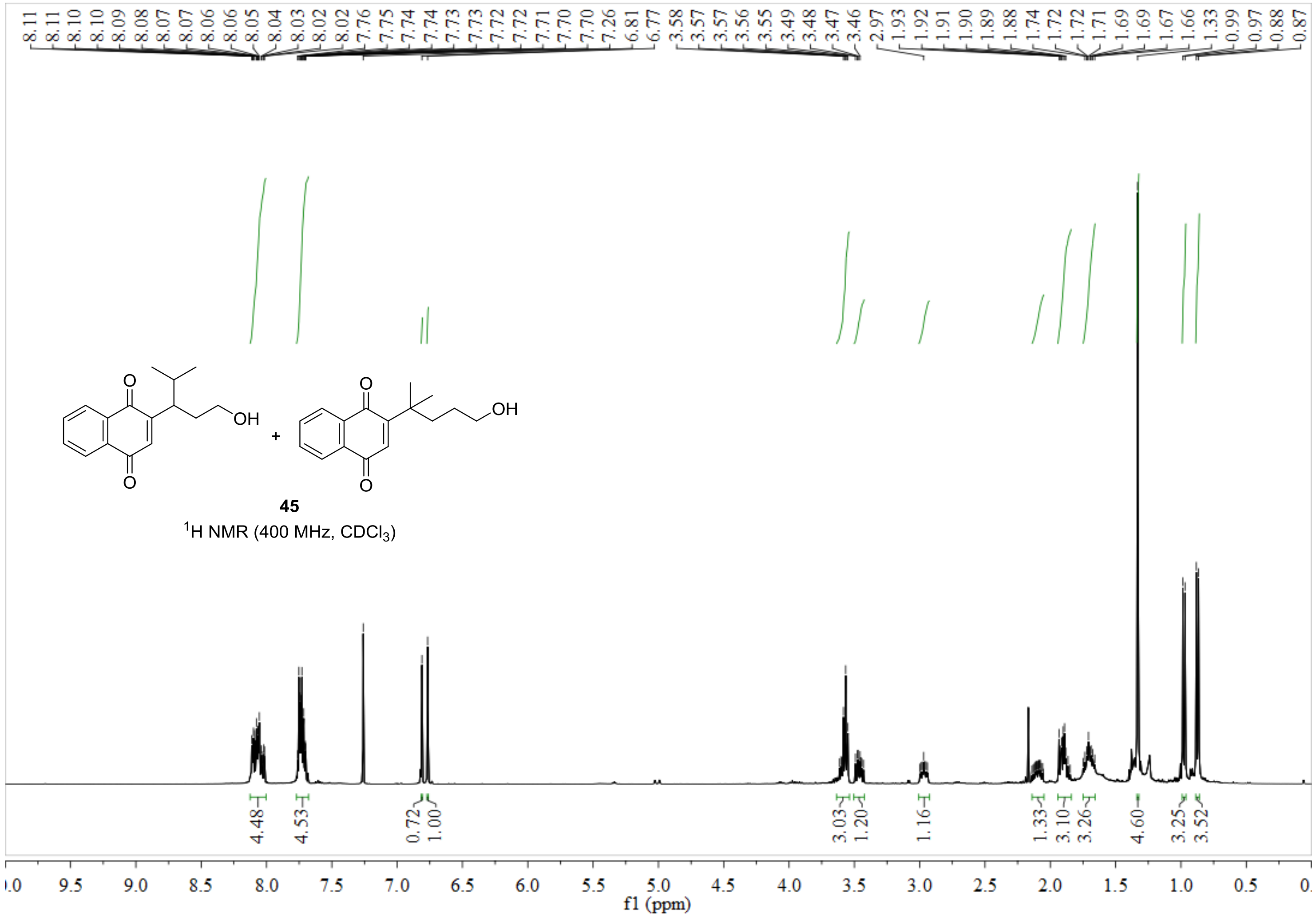
O
OH
+
OH
45
^{1}H NMR (400 MHz, CDCl3)
f1 (ppm)

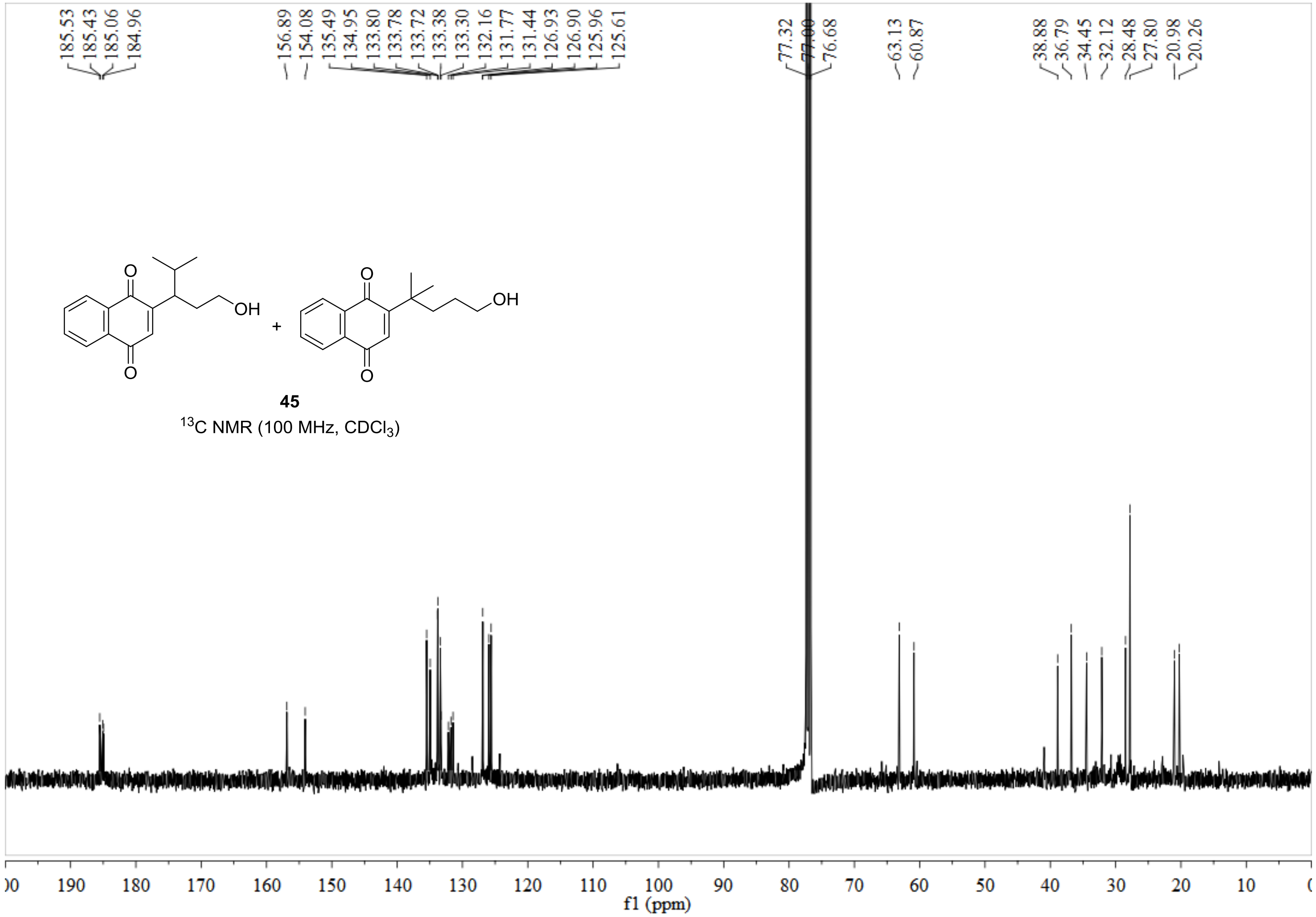

185.53
185.43
185.06
184.96
156.89
154.08
135.49
134.95
133.80
133.78
133.72
133.38
133.30
132.16
131.77
131.44
126.93
126.90
125.96
125.61
77.32
77.00
76.68
63.13
60.87
38.88
36.79
34.45
32.12
28.48
27.80
20.98
20.26
OH
+
OH
45
^{13}C NMR (100 MHz, $CDCl_3$)
f1 (ppm)

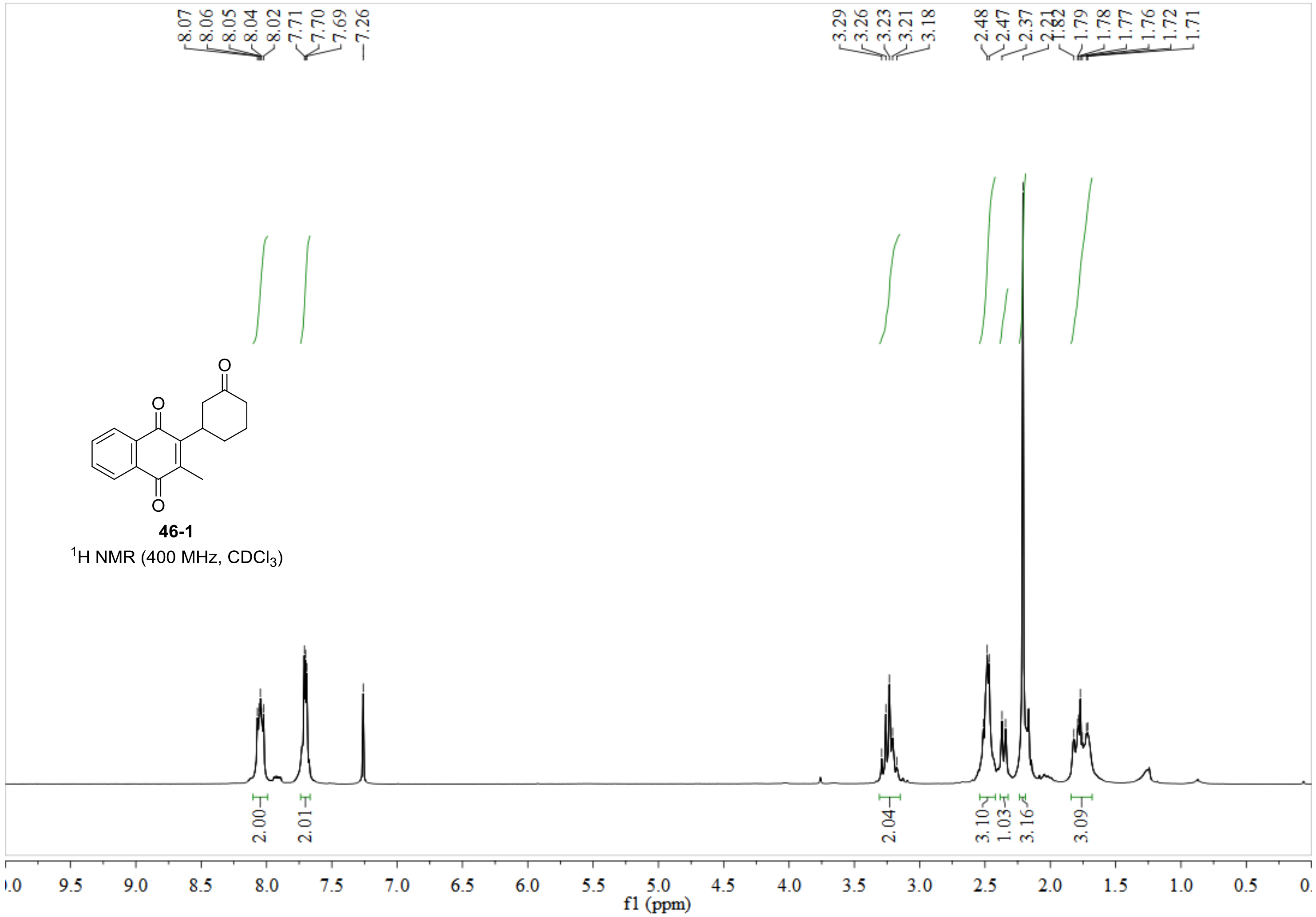
8.07
8.06
8.05
8.04
8.02
7.71
7.70
7.69
7.26
3.29
3.26
3.23
3.21
3.18
2.48
2.47
2.37
2.21
1.82
1.79
1.78
1.77
1.76
1.72
1.71
46-1
^{1}H NMR (400 MHz, $CDCl_3$)
2.00
2.01
2.04
3.10
1.03
3.16
3.09
f1 (ppm)

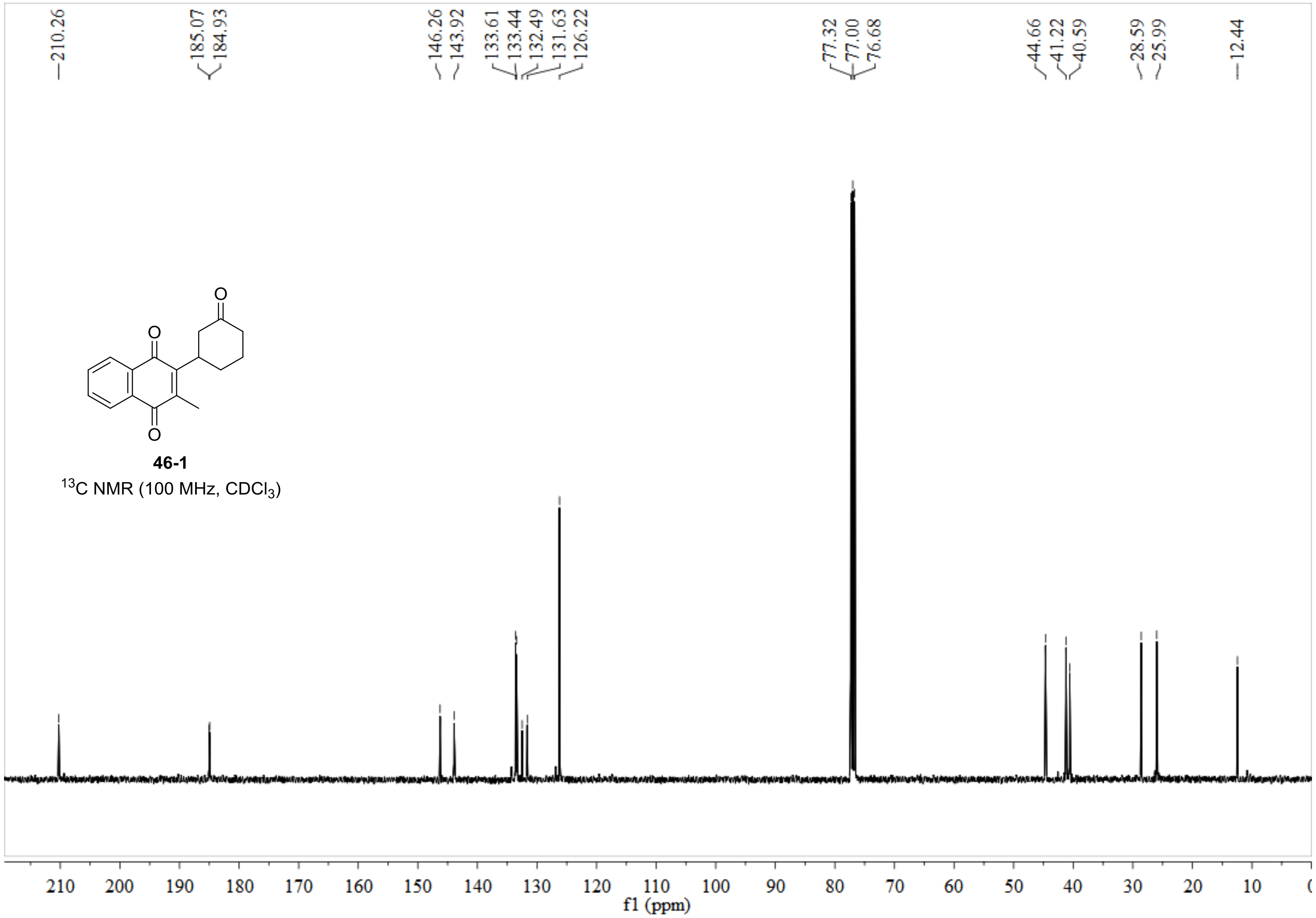
210.26
185.07
184.93
146.26
143.92
133.61
133.44
132.49
131.63
126.22
77.32
77.00
76.68
44.66
41.22
40.59
28.59
25.99
12.44
46-1
13C NMR (100 MHz, CDCl3)
210
200
190
180
170
160
150
140
130
120
110
100
90
80
70
60
50
40
30
20
10
f1 (ppm)

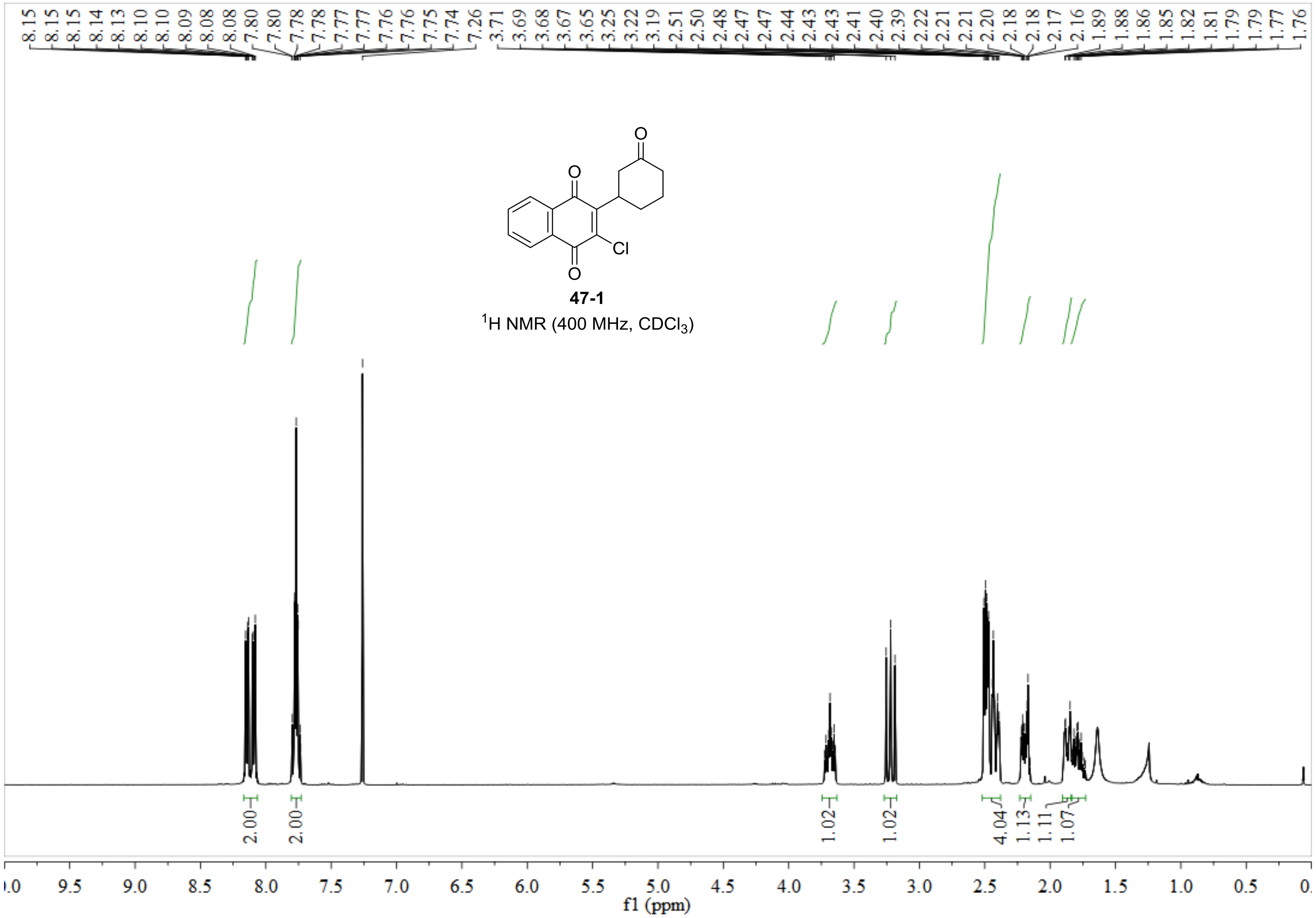

47-1

^{1}H NMR (400 MHz, $CDCl_3$)

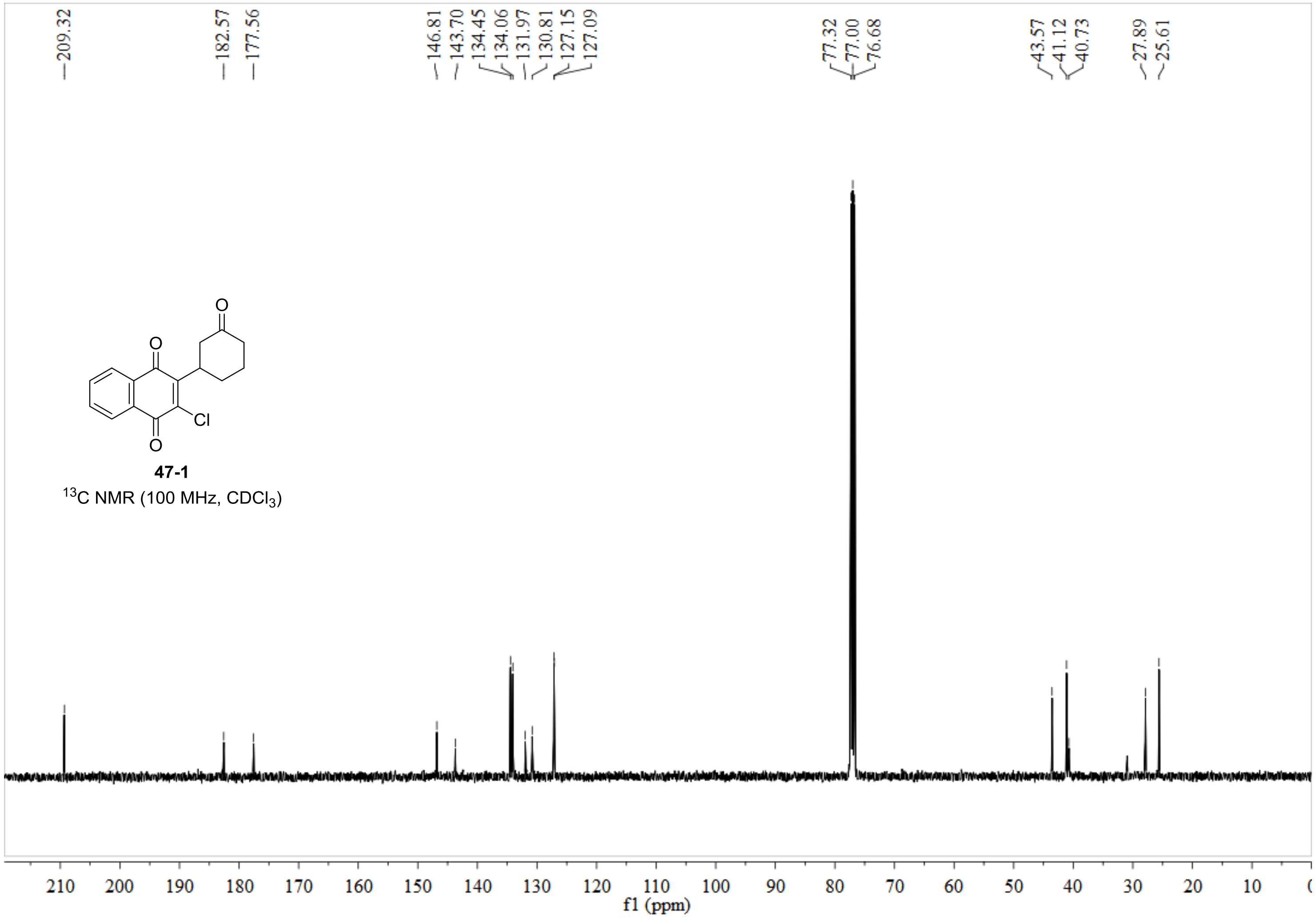
209.32
182.57
177.56
146.81
143.70
134.45
134.06
131.97
130.81
127.15
127.09
77.32
77.00
76.68
43.57
41.12
40.73
27.89
25.61
O
O
O
Cl
47-1
^{13}C NMR (100 MHz, $CDCl_3$)
210
200
190
180
170
160
150
140
130
120
110
100
90
80
70
60
50
40
30
20
10
f1 (ppm)

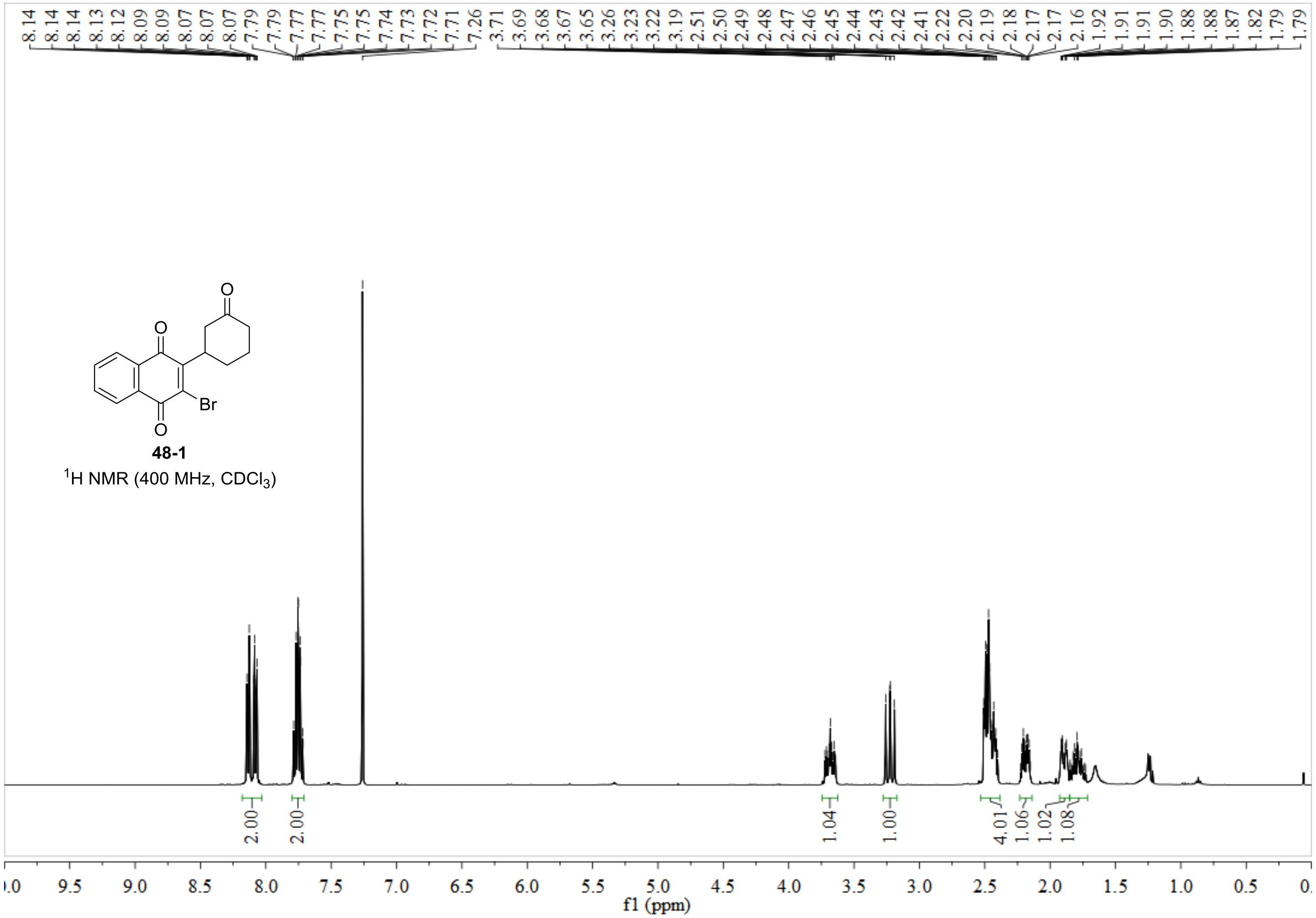
8.14
8.14
8.14
8.13
8.12
8.09
8.09
8.07
8.07
8.07
7.79
7.79
7.77
7.77
7.75
7.75
7.74
7.73
7.72
7.71
7.26
3.71
3.69
3.68
3.67
3.65
3.26
3.23
3.22
3.19
2.51
2.50
2.49
2.48
2.47
2.46
2.45
2.44
2.43
2.42
2.41
2.22
2.20
2.19
2.18
2.17
2.17
2.16
1.92
1.91
1.91
1.90
1.88
1.87
1.82
1.79
1.79
O
O
O
Br
48-1
¹H NMR (400 MHz, CDCl₃)
2.00
2.00
1.04
1.00
4.01
1.06
1.02
1.08
9.5
9.0
8.5
8.0
7.5
7.0
6.5
6.0
5.5
5.0
4.5
4.0
3.5
3.0
2.5
2.0
1.5
1.0
0.5
f1 (ppm)

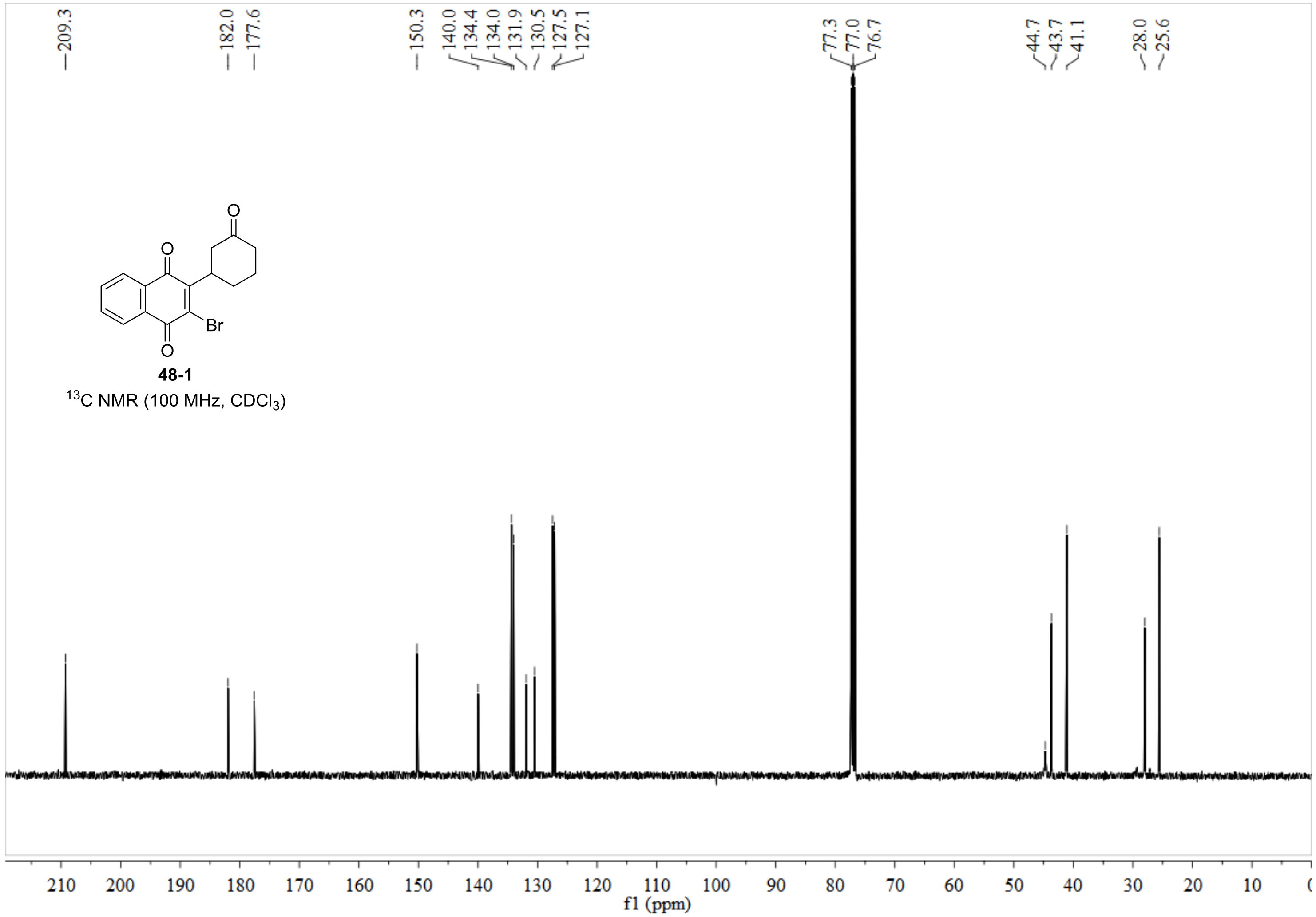
209.3
182.0
177.6
150.3
140.0
134.4
134.0
131.9
130.5
127.5
127.1
77.3
77.0
76.7
44.7
43.7
41.1
28.0
25.6
O
O
O
Br
48-1
^{13}C NMR (100 MHz, $CDCl_3$)
f1 (ppm)

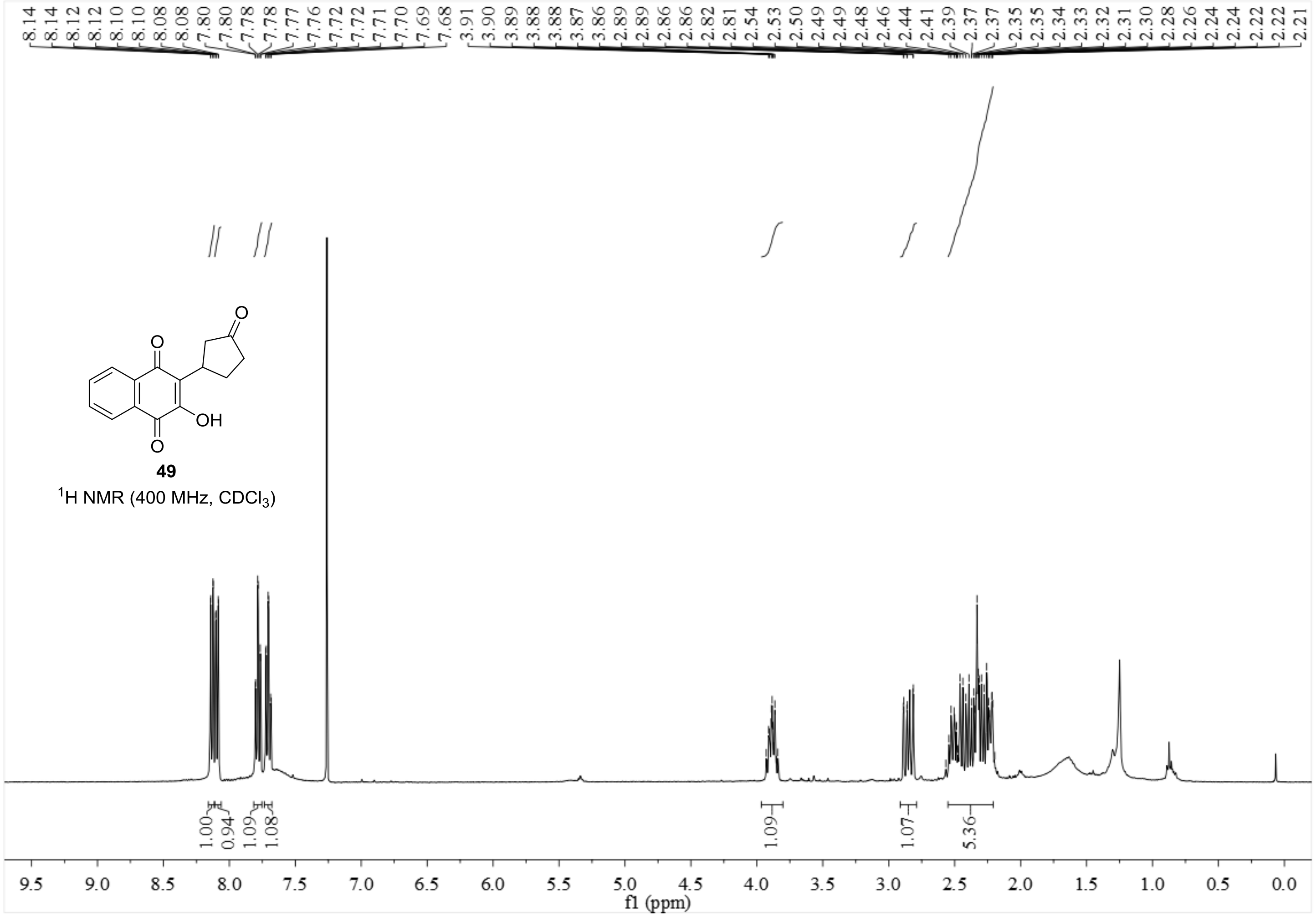

8.14 8.14 8.12 8.12 8.10 8.10 8.08 8.08 7.80 7.80 7.78 7.78 7.77 7.76 7.72 7.72 7.71 7.70 7.69 7.68 3.91 3.90 3.89 3.88 3.88 3.87 3.86 2.89 2.89 2.86 2.86 2.82 2.81 2.54 2.53 2.50 2.49 2.49 2.48 2.46 2.44 2.41 2.39 2.37 2.37 2.35 2.35 2.34 2.33 2.32 2.31 2.30 2.28 2.26 2.24 2.24 2.22 2.22 2.21
O
O
O
OH
49
¹H NMR (400 MHz, CDCl₃)
1.00
0.94
1.09
1.08
1.09
1.07
5.36
9.5 9.0 8.5 8.0 7.5 7.0 6.5 6.0 5.5 5.0 4.5 4.0 3.5 3.0 2.5 2.0 1.5 1.0 0.5 0.0
f1 (ppm)

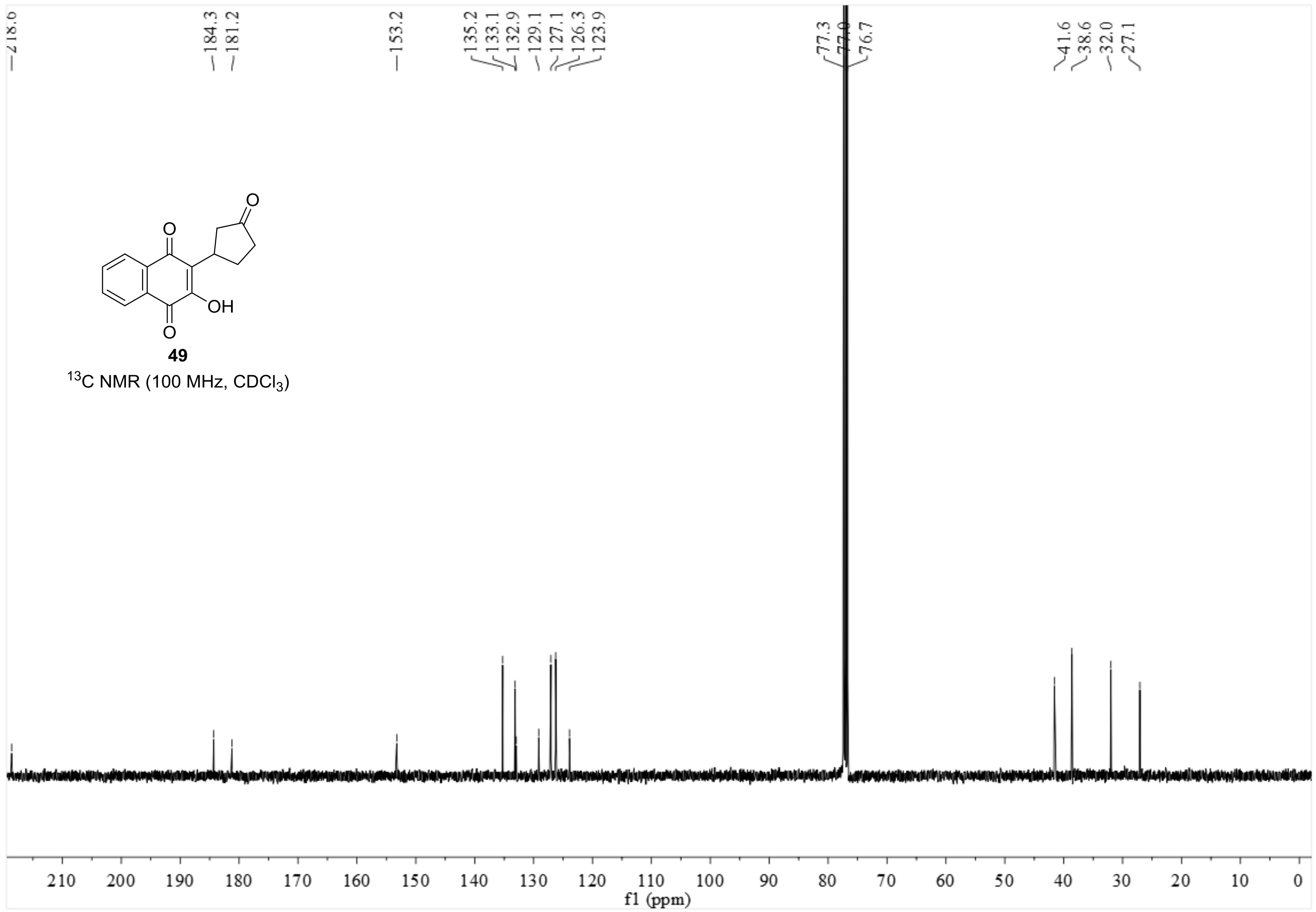
218.6
184.3
181.2
153.2
135.2
133.1
132.9
129.1
127.1
126.3
123.9
77.3
77.0
76.7
41.6
38.6
32.0
27.1
O
O
O
OH
49
^{13}C NMR (100 MHz, $CDCl_3$)
210 200 190 180 170 160 150 140 130 120 110 100 90 80 70 60 50 40 30 20 10 0
f1 (ppm)

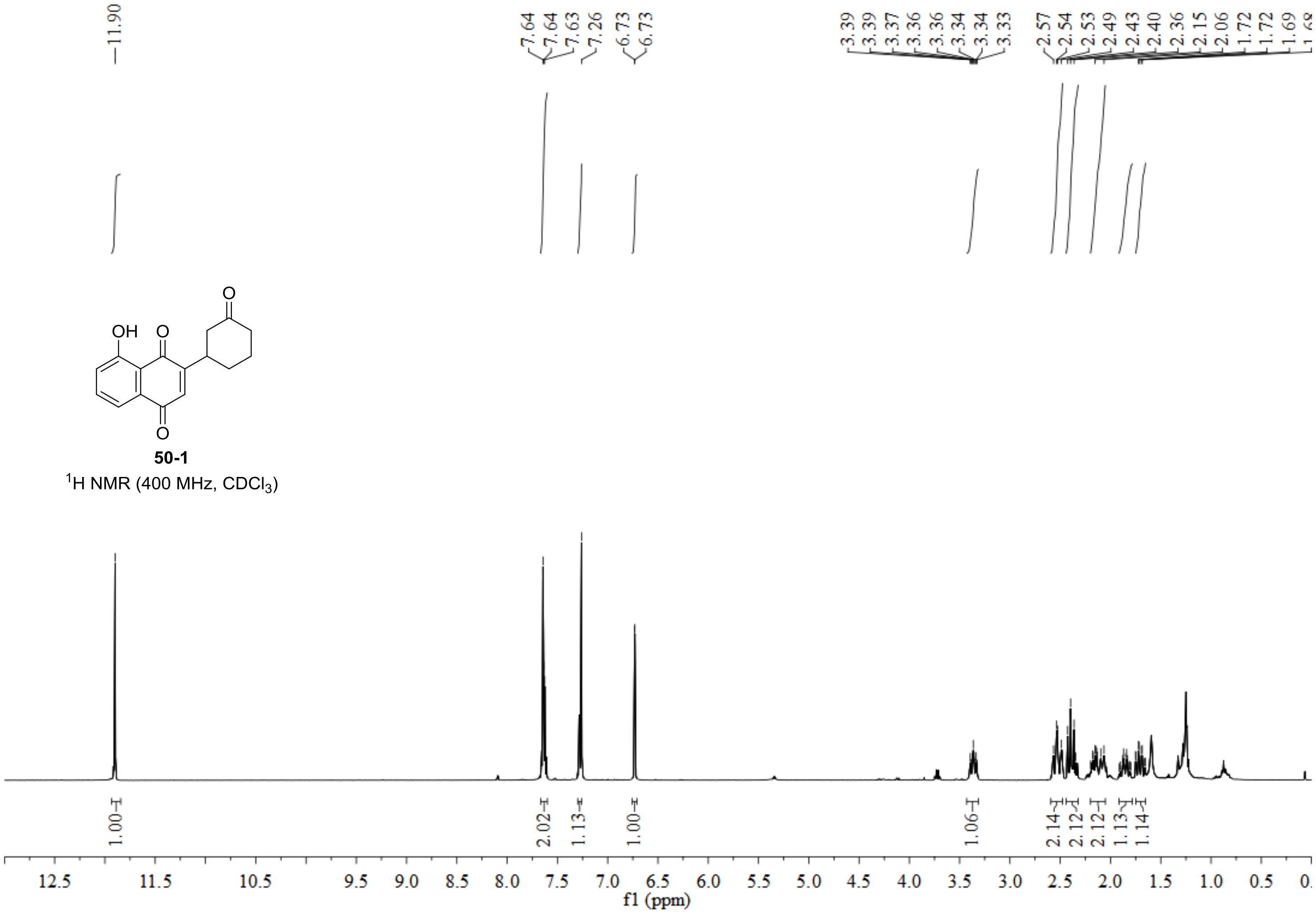

50-1
^{1}H NMR (400 MHz, $CDCl_3$)
11.90
7.64 7.64 7.63 7.26 6.73 6.73
3.39 3.39 3.37 3.36 3.36 3.34 3.34 3.33
2.57 2.54 2.53 2.49 2.43 2.40 2.36 2.15 2.06 1.72 1.72 1.69
1.00 2.02 1.13 1.00 1.06 2.14 2.12 2.12 1.13 1.14
12.5 11.5 10.5 9.5 9.0 8.5 8.0 7.5 7.0 6.5 6.0 5.5 5.0 4.5 4.0 3.5 3.0 2.5 2.0 1.5 1.0 0.5
f1 (ppm)

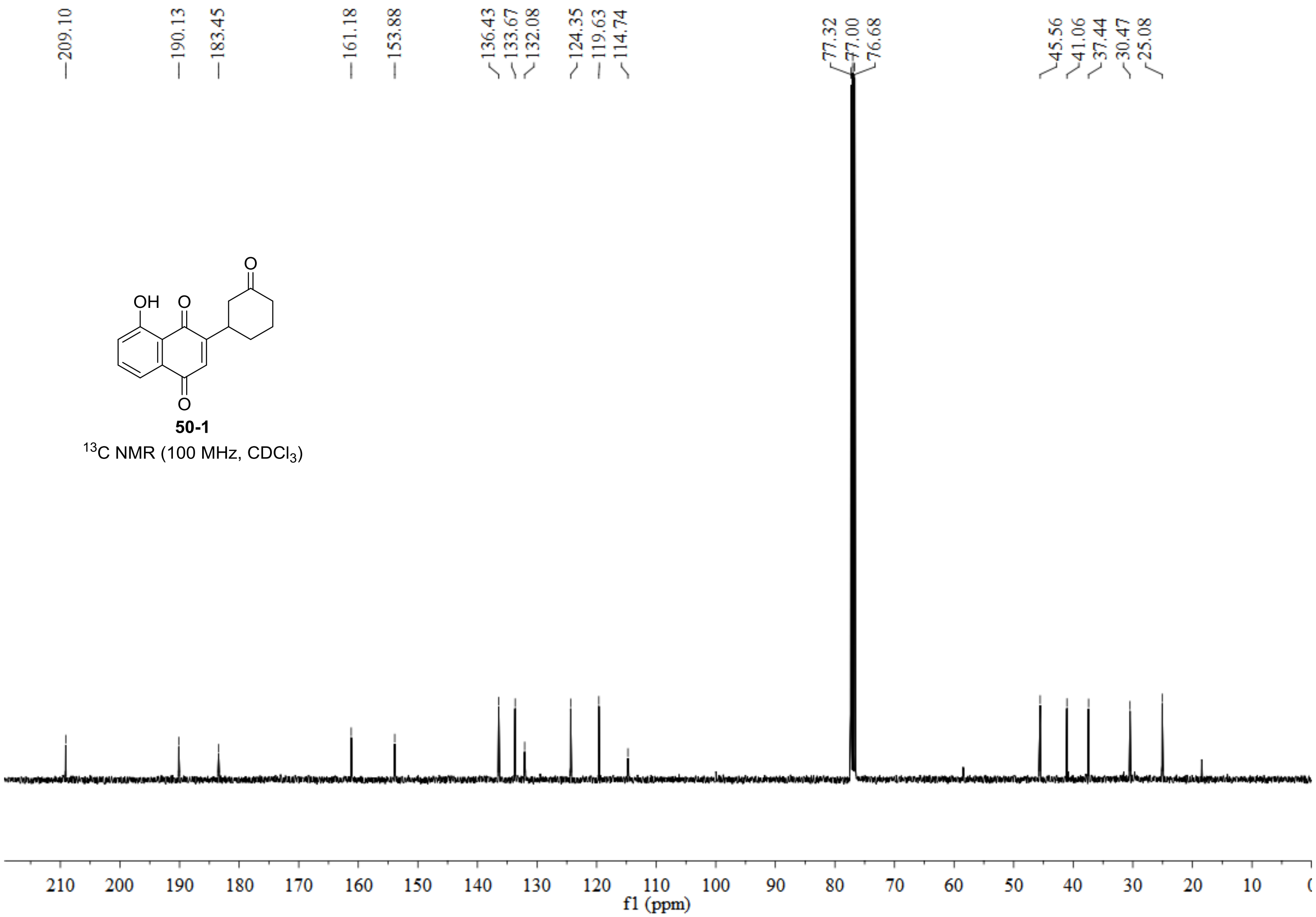

50-1

^{13}C NMR (100 MHz, $CDCl_3$)

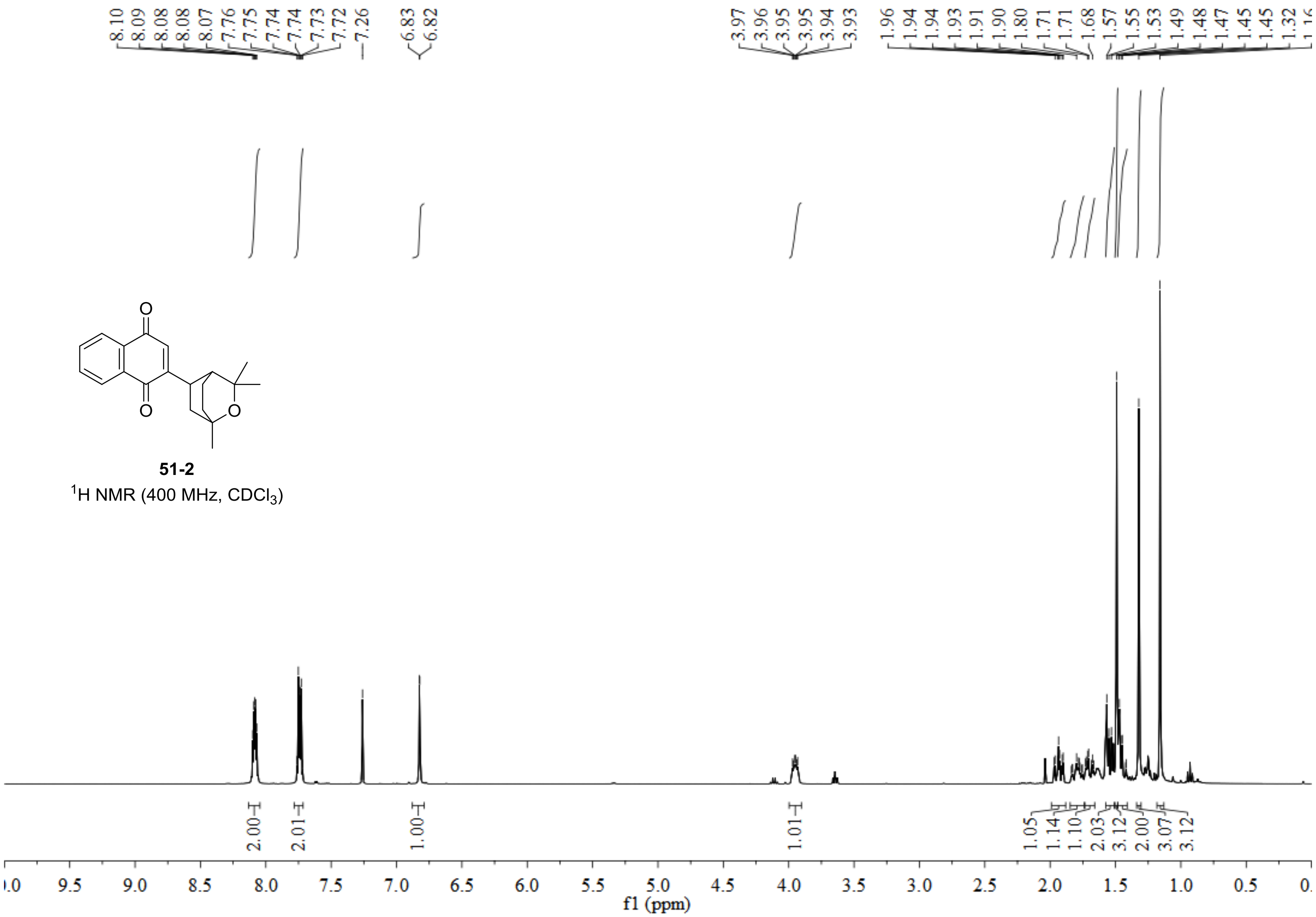

51-2
^{1}H NMR (400 MHz, $CDCl_3$)

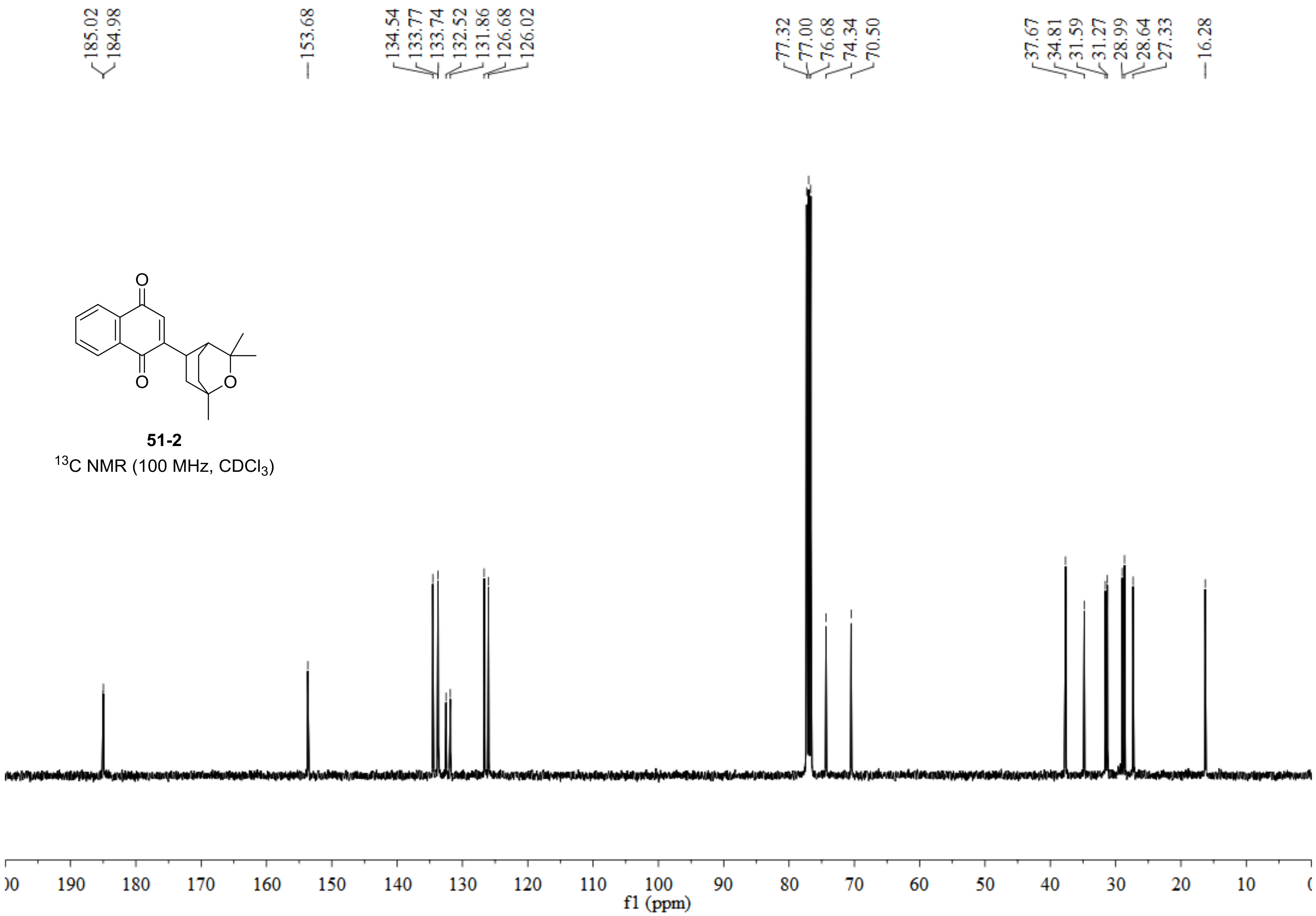
185.02
184.98
153.68
134.54
133.77
133.74
132.52
131.86
126.68
126.02
77.32
77.00
76.68
74.34
70.50
37.67
34.81
31.59
31.27
28.99
28.64
27.33
16.28
O
O
O
51-2
^{13}C NMR (100 MHz, $CDCl_3$)
f1 (ppm)

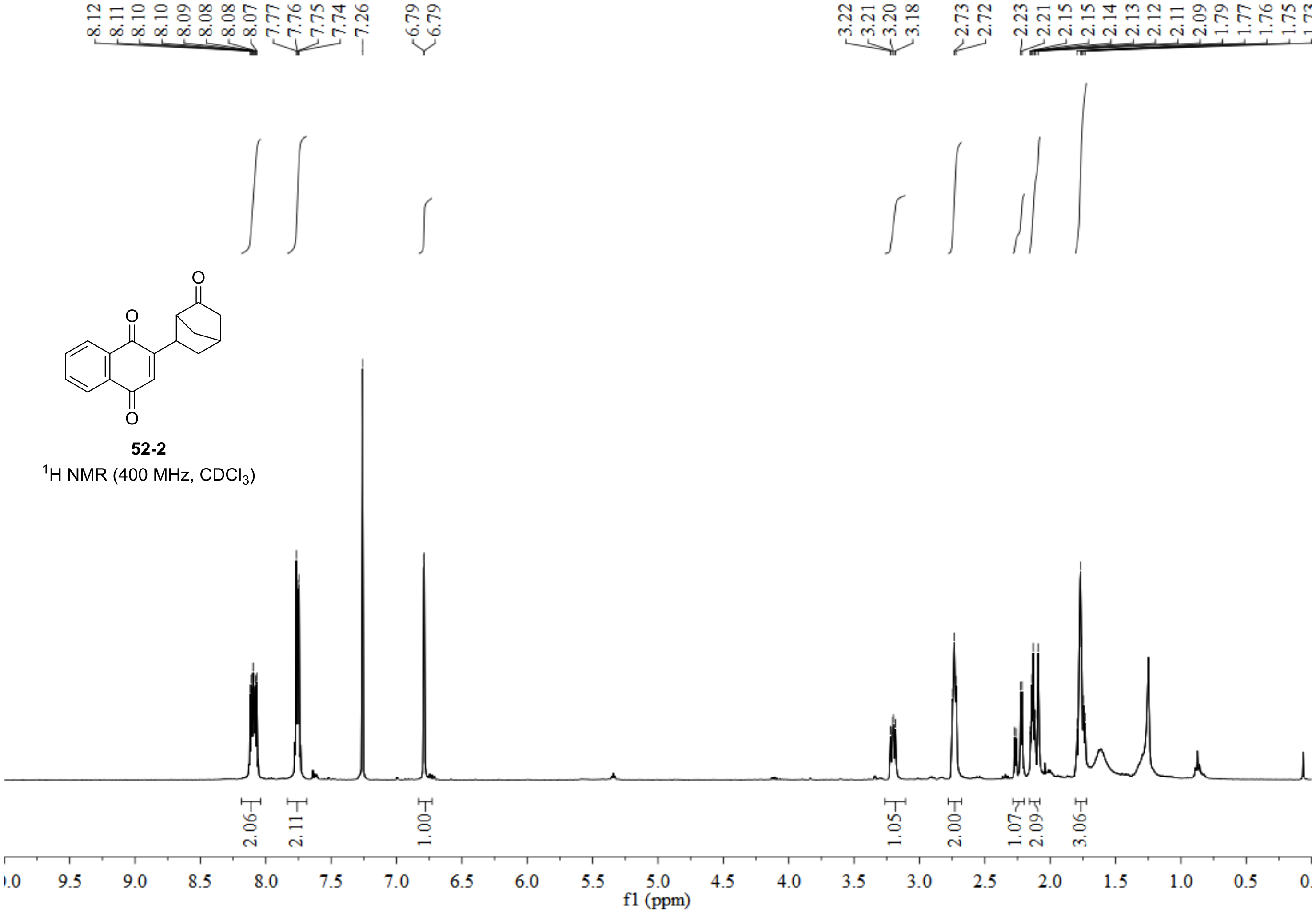

52-2

^{1}H NMR (400 MHz, $CDCl_3$)

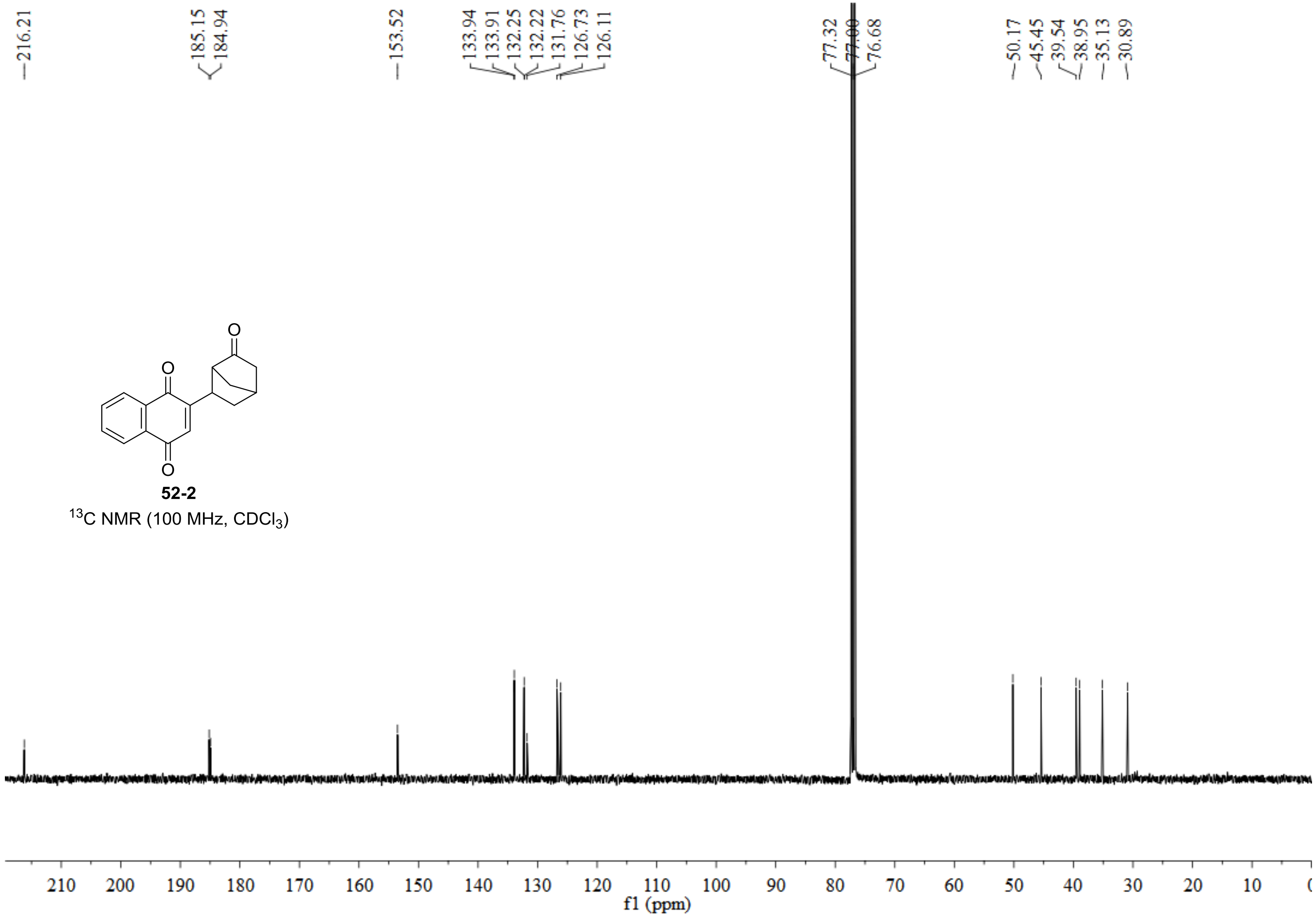
216.21
185.15
184.94
153.52
133.94
133.91
132.25
132.22
131.76
126.73
126.11
77.32
77.00
76.68
50.17
45.45
39.54
38.95
35.13
30.89
52-2
^{13}C NMR (100 MHz, $CDCl_3$)
210 200 190 180 170 160 150 140 130 120 110 100 90 80 70 60 50 40 30 20 10 0
f1 (ppm)

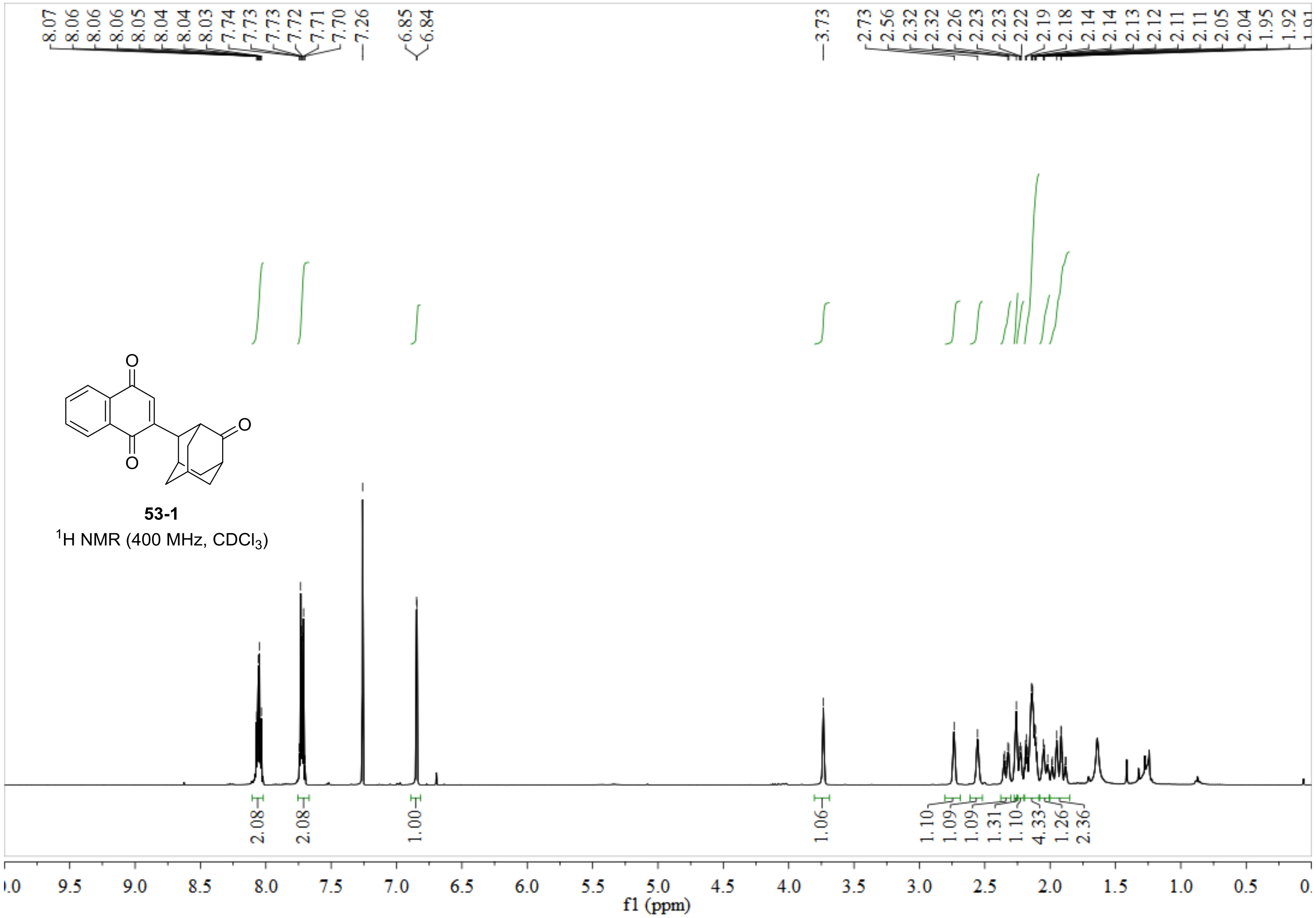

53-1

^{1}H NMR (400 MHz, $CDCl_3$)

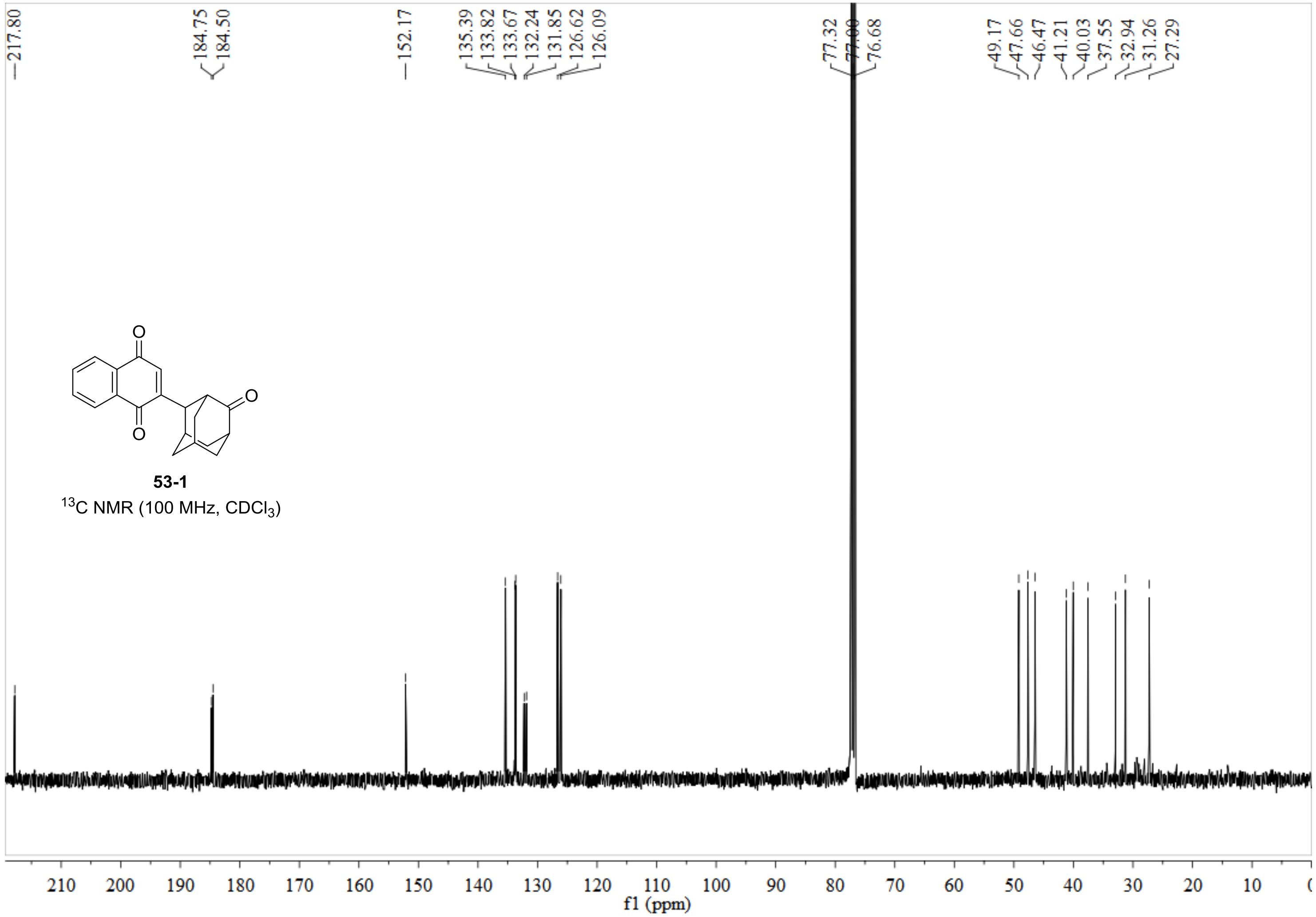

53-1

^{13}C NMR (100 MHz, $CDCl_3$)

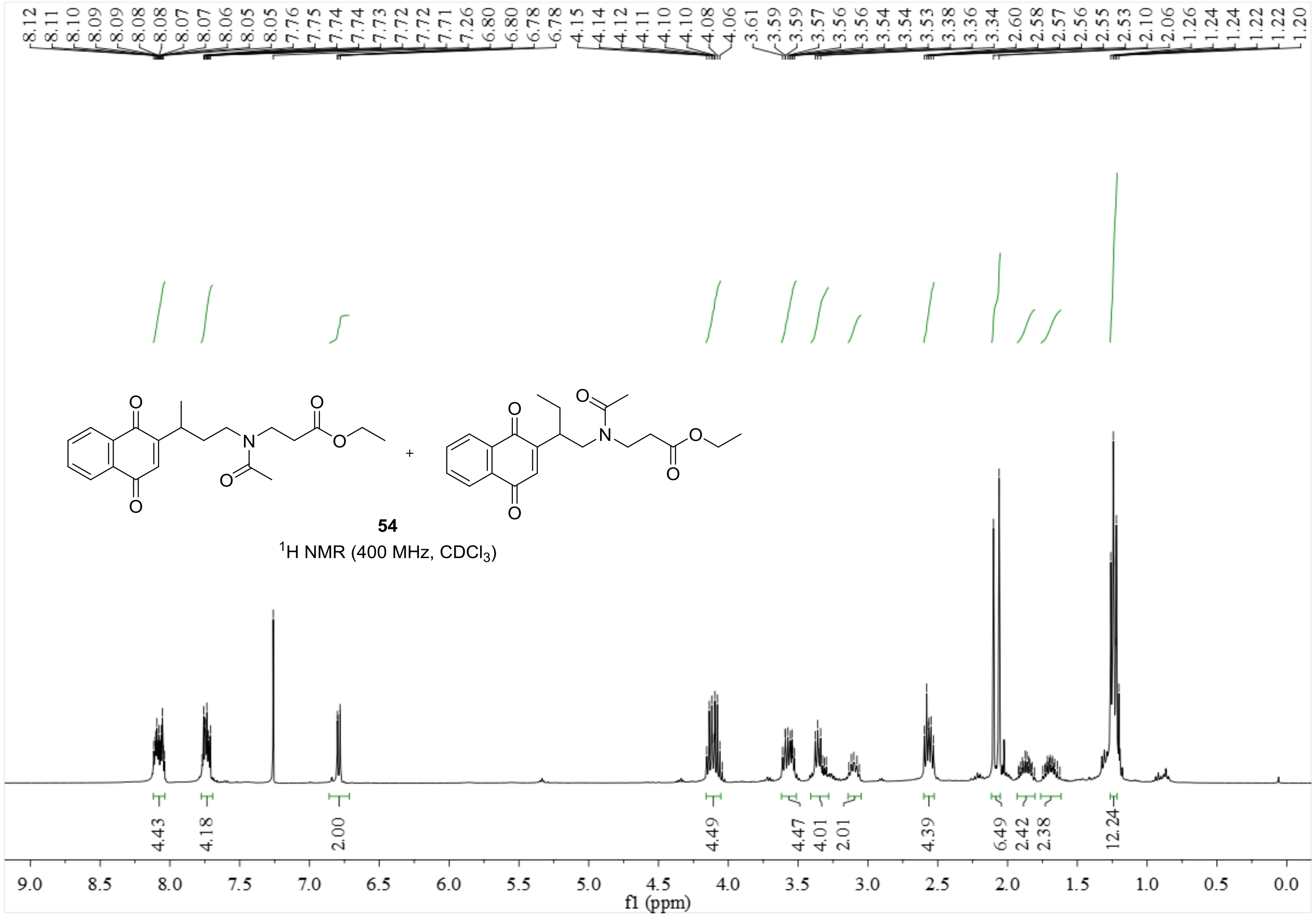

54
^{1}H NMR (400 MHz, CDCl$_3$)
f1 (ppm)

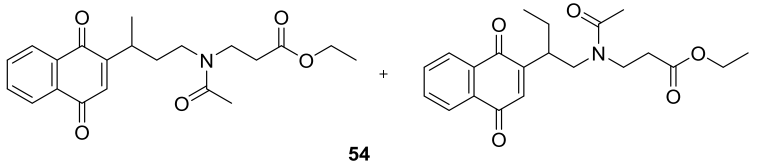

54

^{13}H NMR (100 MHz, $CDCl_3$)

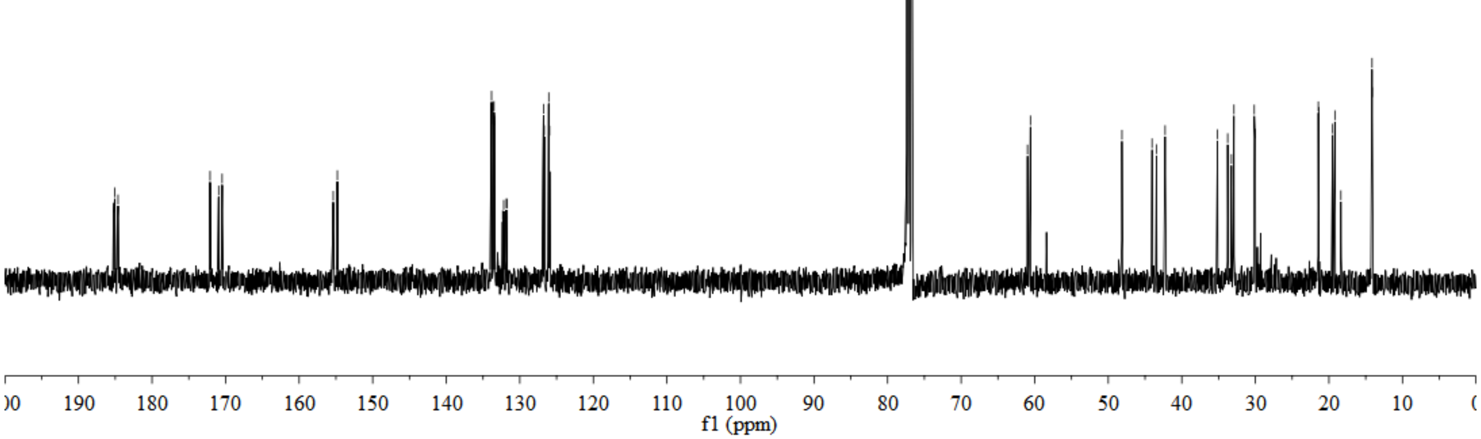

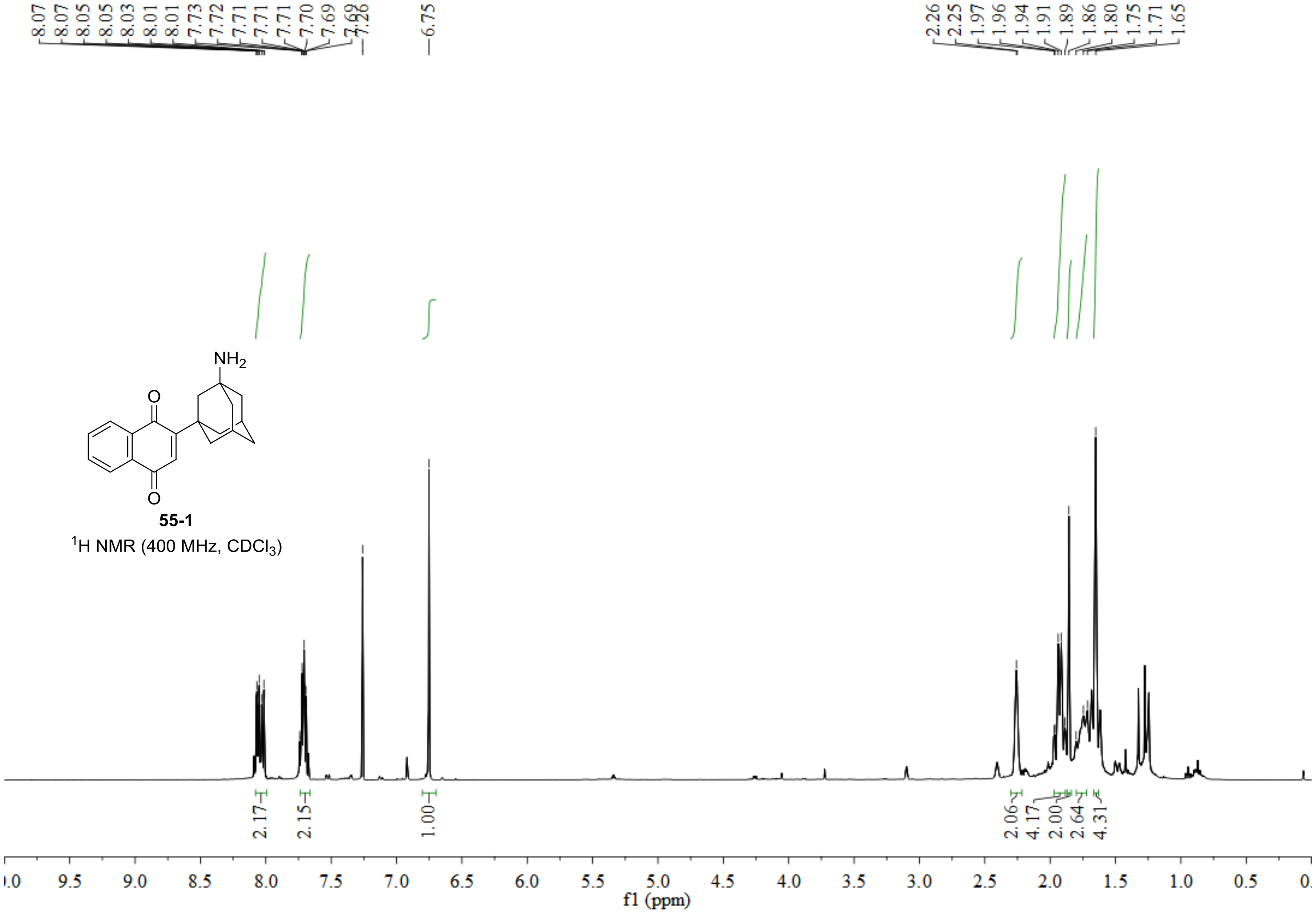

55-1

^{1}H NMR (400 MHz, $CDCl_3$)

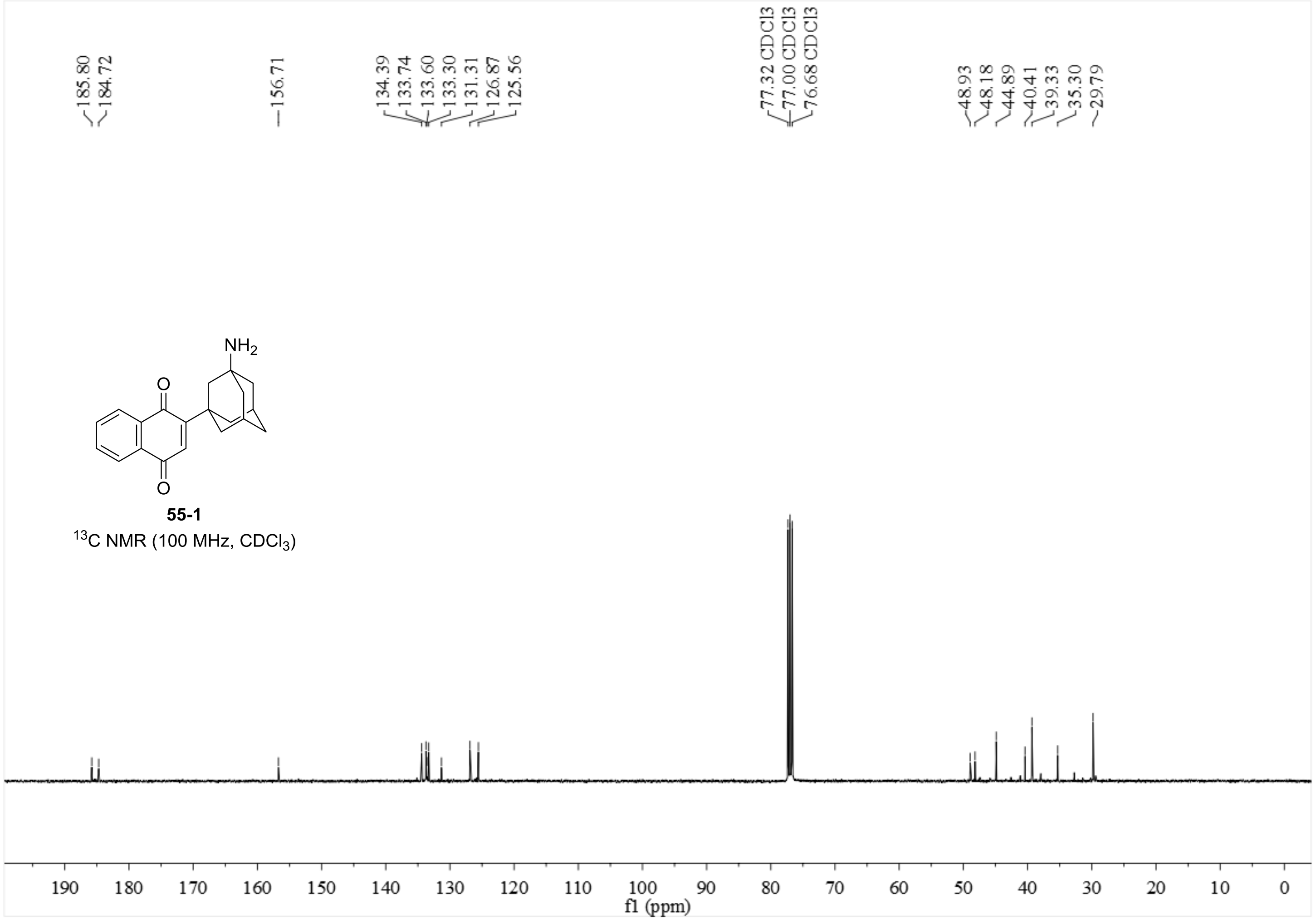

55-1

^{13}C NMR (100 MHz, $CDCl_3$)

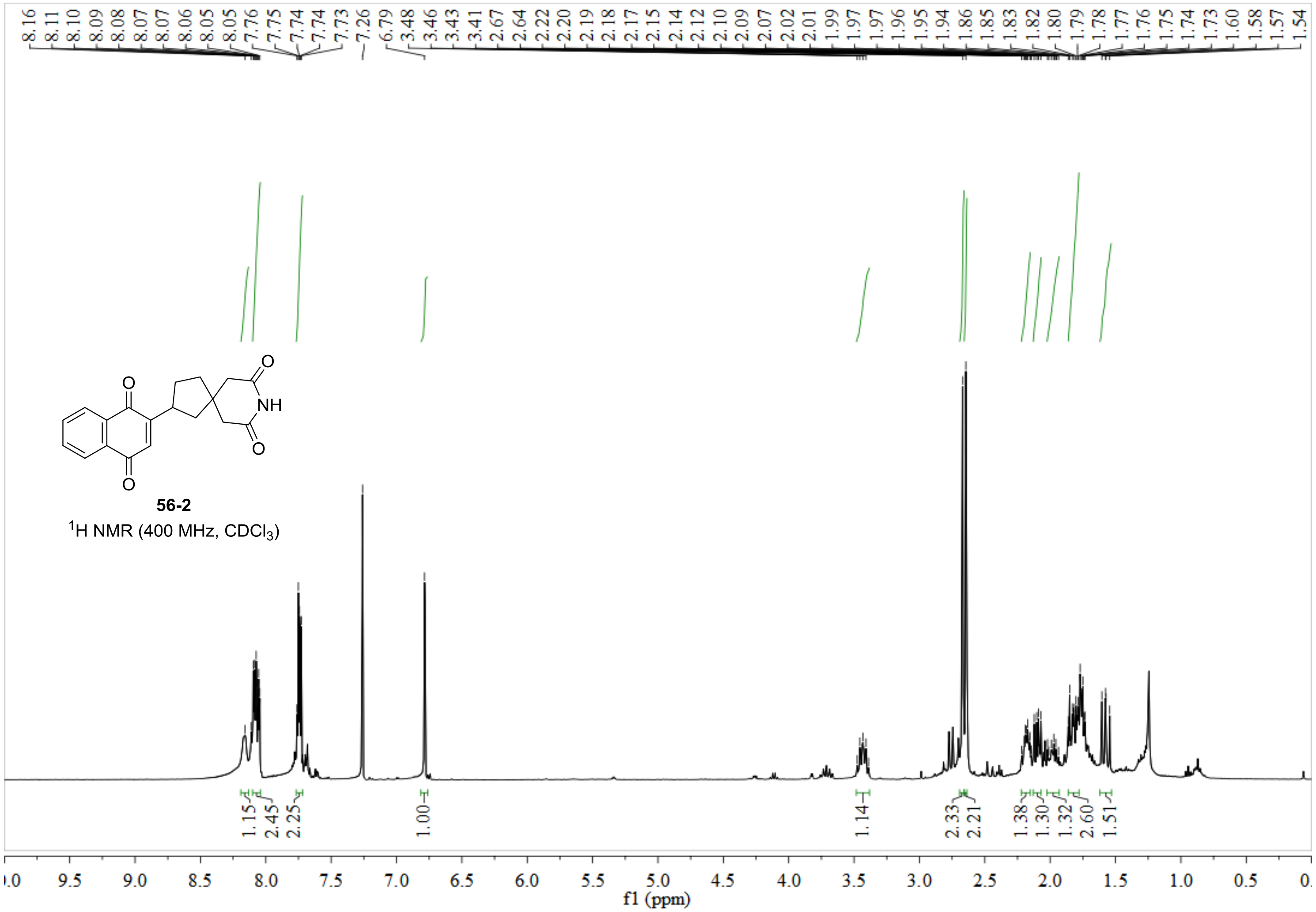
8.16 8.11 8.10 8.09 8.08 8.07 8.07 8.06 8.05 8.05 7.76 7.75 7.74 7.74 7.73 7.26 6.79 3.48 3.46 3.43 3.41 2.67 2.64 2.22 2.20 2.19 2.18 2.17 2.15 2.14 2.12 2.10 2.09 2.07 2.02 2.01 1.99 1.97 1.97 1.96 1.95 1.94 1.86 1.85 1.83 1.82 1.80 1.79 1.78 1.77 1.76 1.75 1.74 1.73 1.60 1.58 1.57 1.54
O
NH
O
O
O
56-2
^{1}H NMR (400 MHz, CDCl$_3$)
1.15 2.45 2.25 1.00 1.14 2.33 2.21 1.38 1.30 1.32 2.60 1.51
f1 (ppm)

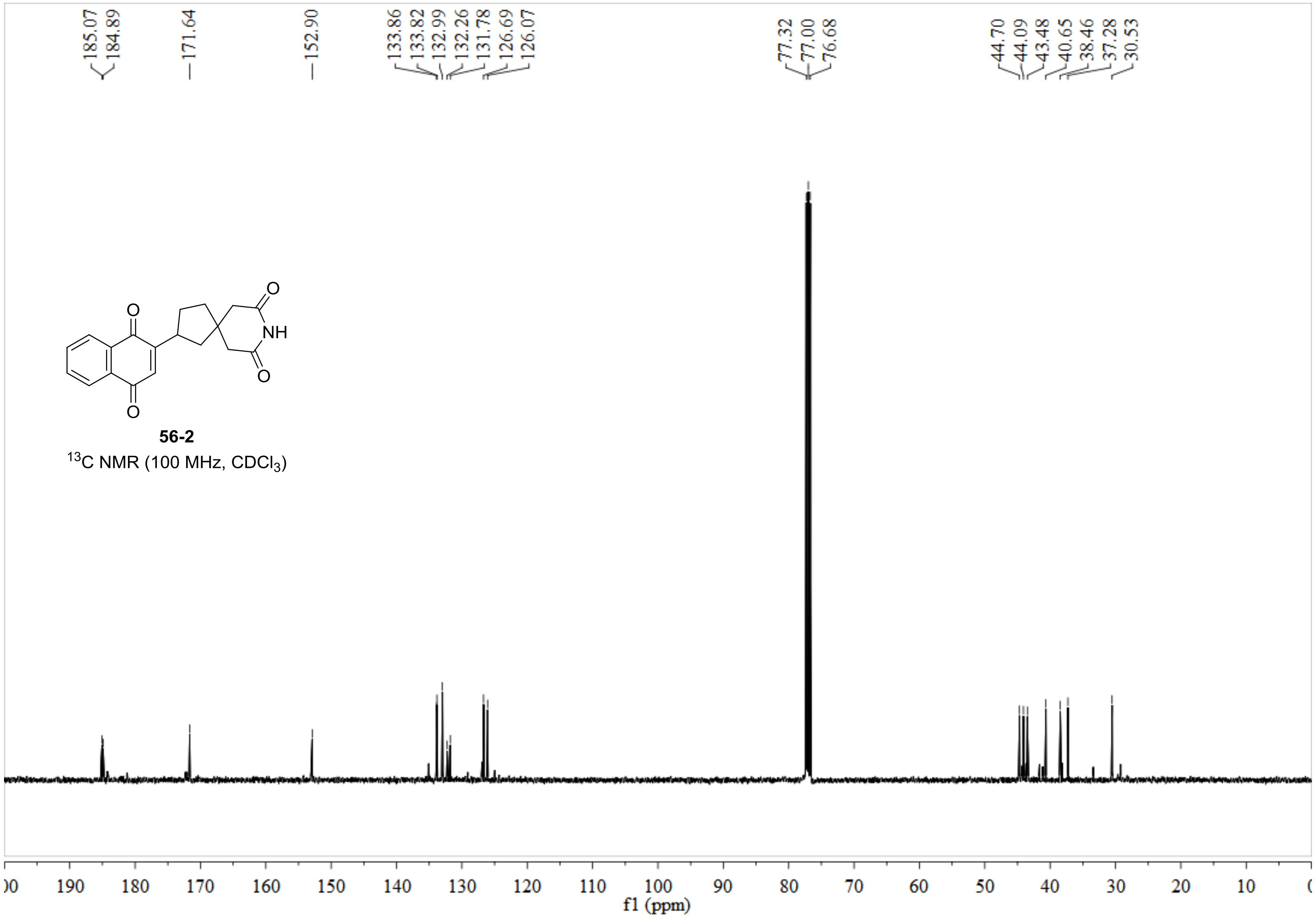

56-2

^{13}C NMR (100 MHz, $CDCl_3$)

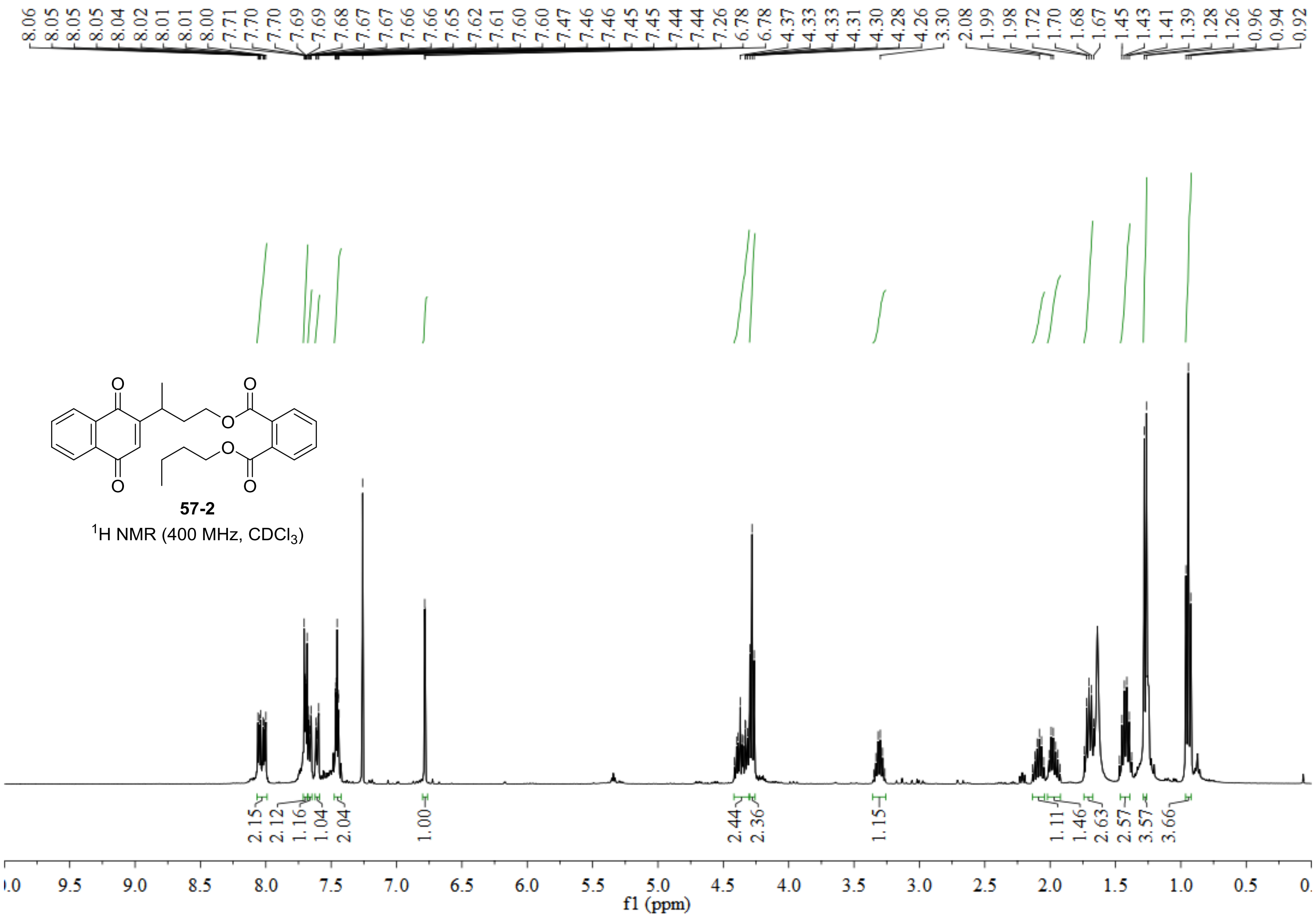

57-2

^{1}H NMR (400 MHz, $CDCl_3$)

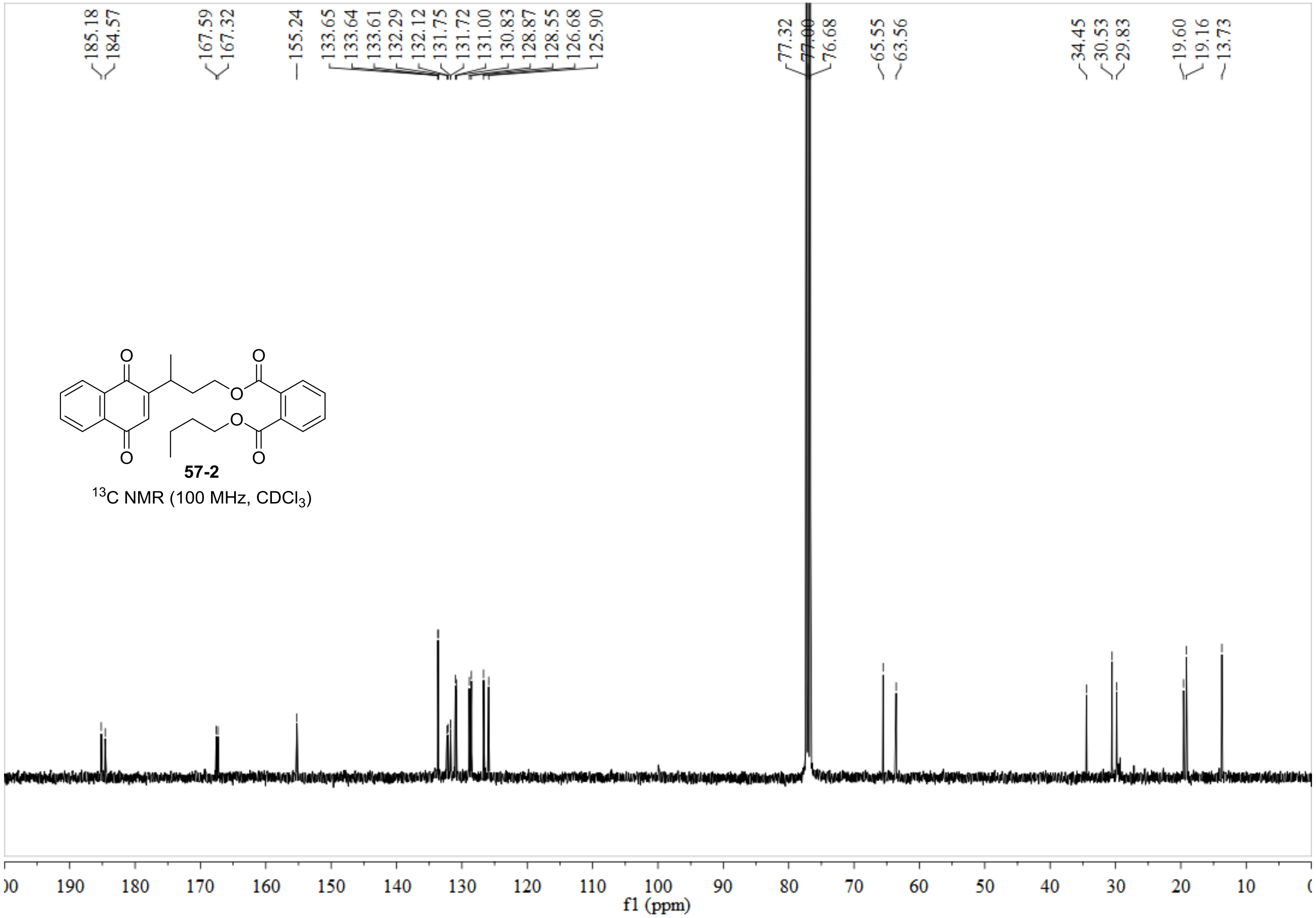
185.18
184.57
167.59
167.32
155.24
133.65
133.64
133.61
132.29
132.12
131.75
131.72
131.00
130.83
128.87
128.55
126.68
125.90
77.32
77.00
76.68
65.55
63.56
34.45
30.53
29.83
19.60
19.16
13.73
O
O
O
O
O
O
57-2
13C NMR (100 MHz, CDCl3)
190
180
170
160
150
140
130
120
110
100
90
80
70
60
50
40
30
20
10
f1 (ppm)

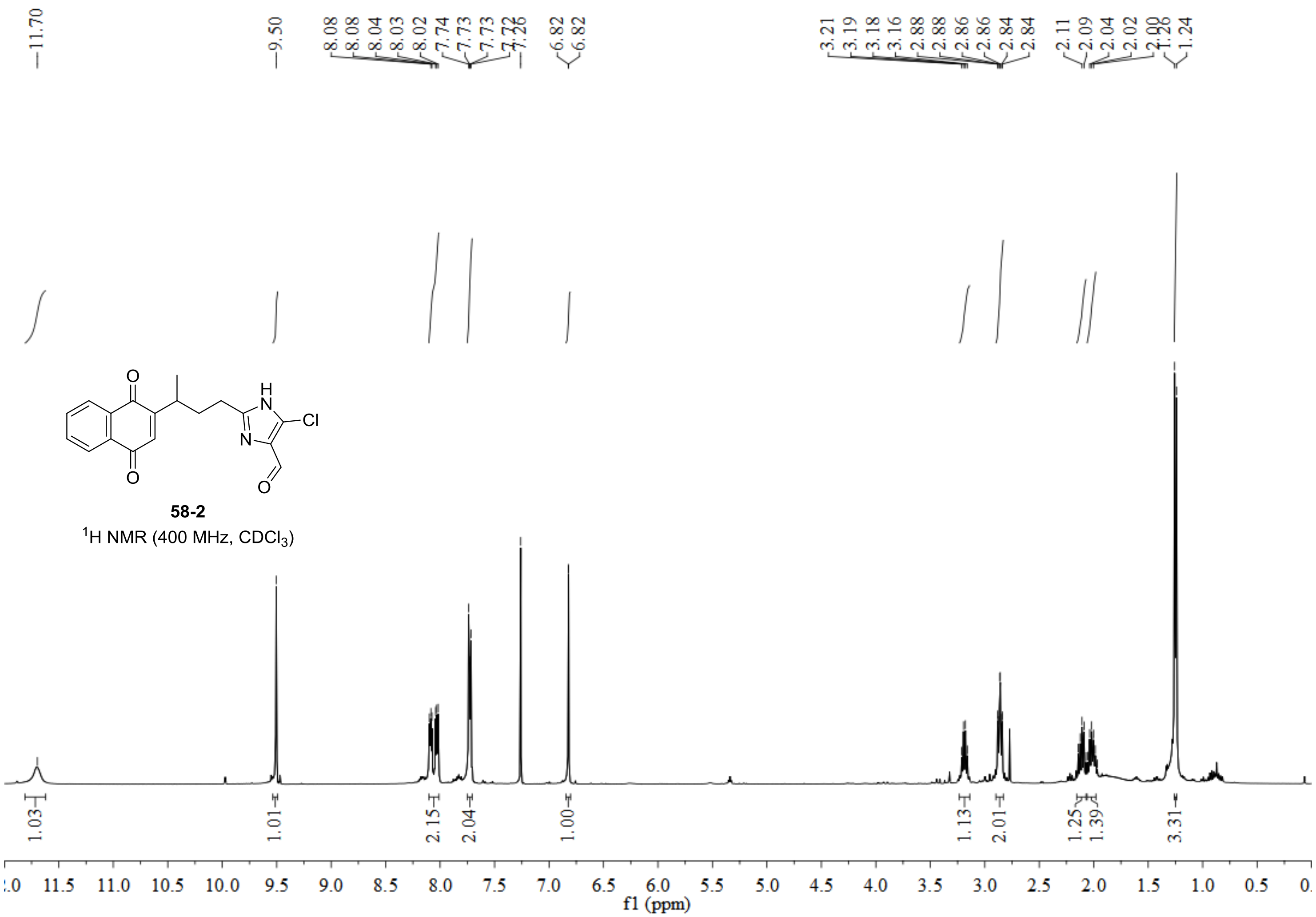

58-2

^{1}H NMR (400 MHz, $CDCl_3$)

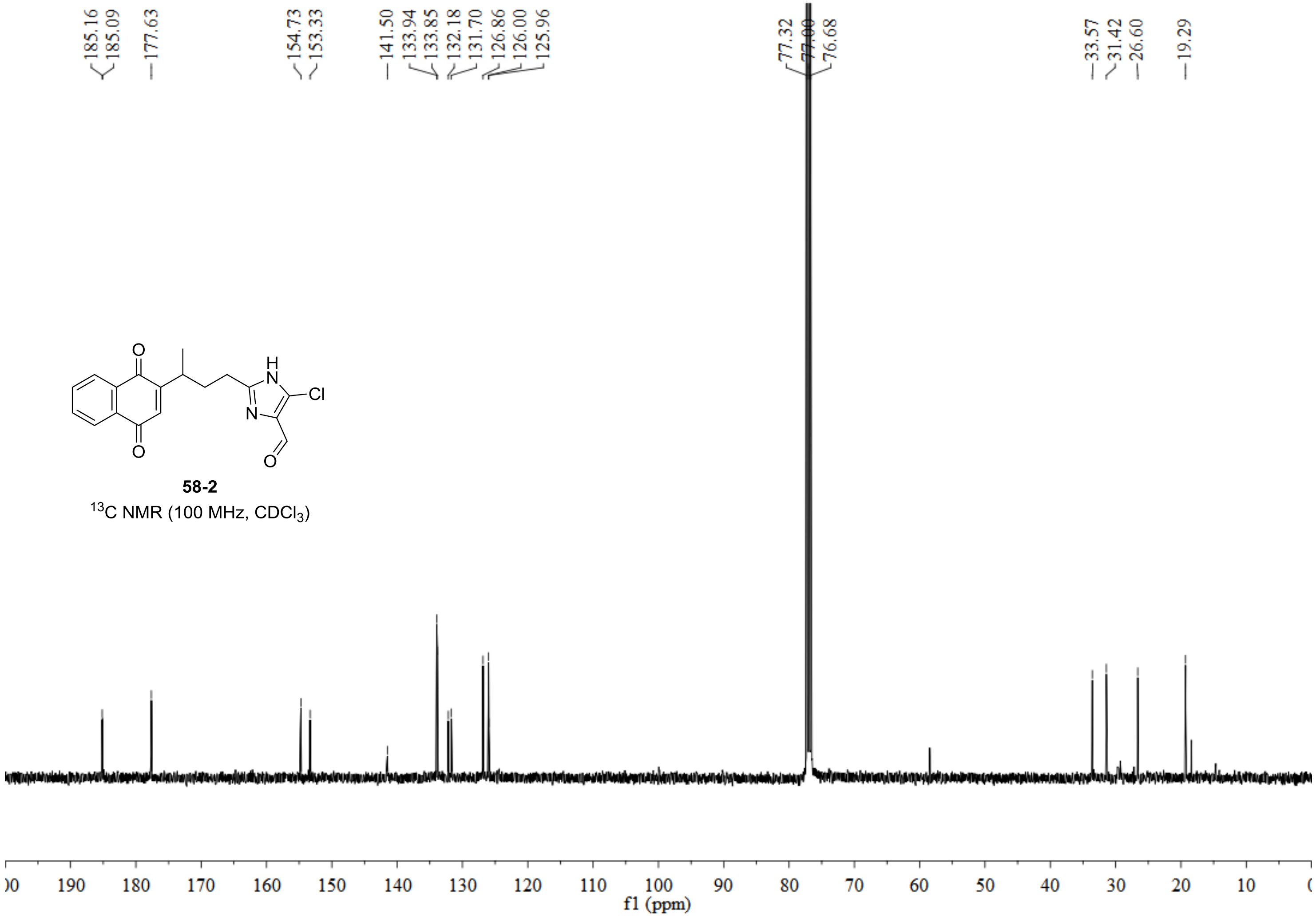

58-2

^{13}C NMR (100 MHz, $CDCl_3$)

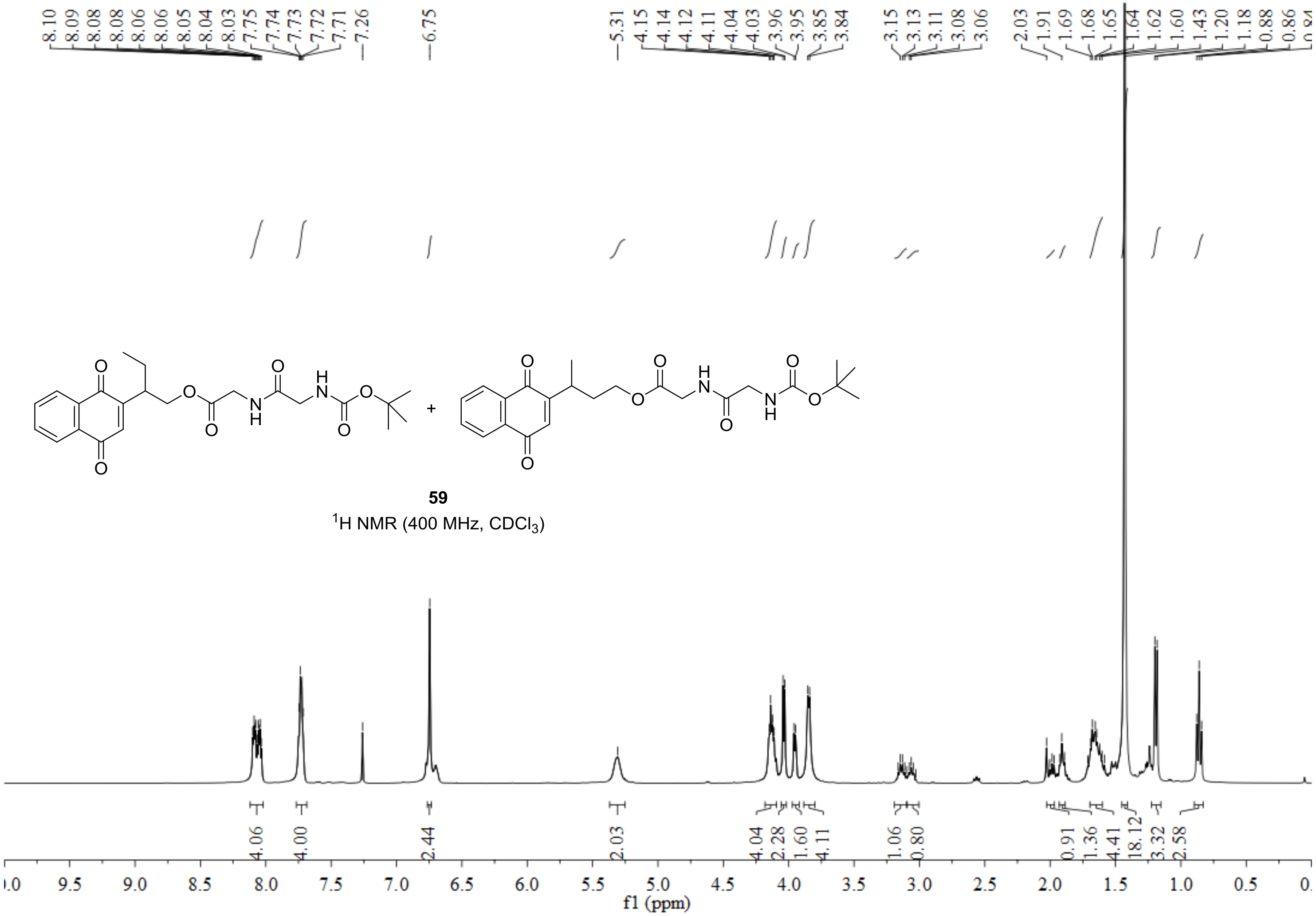

59

^{1}H NMR (400 MHz, $CDCl_3$)

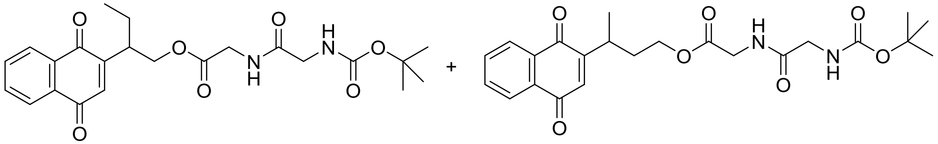

59

^{13}C NMR (100 MHz, CDCl$_3$)

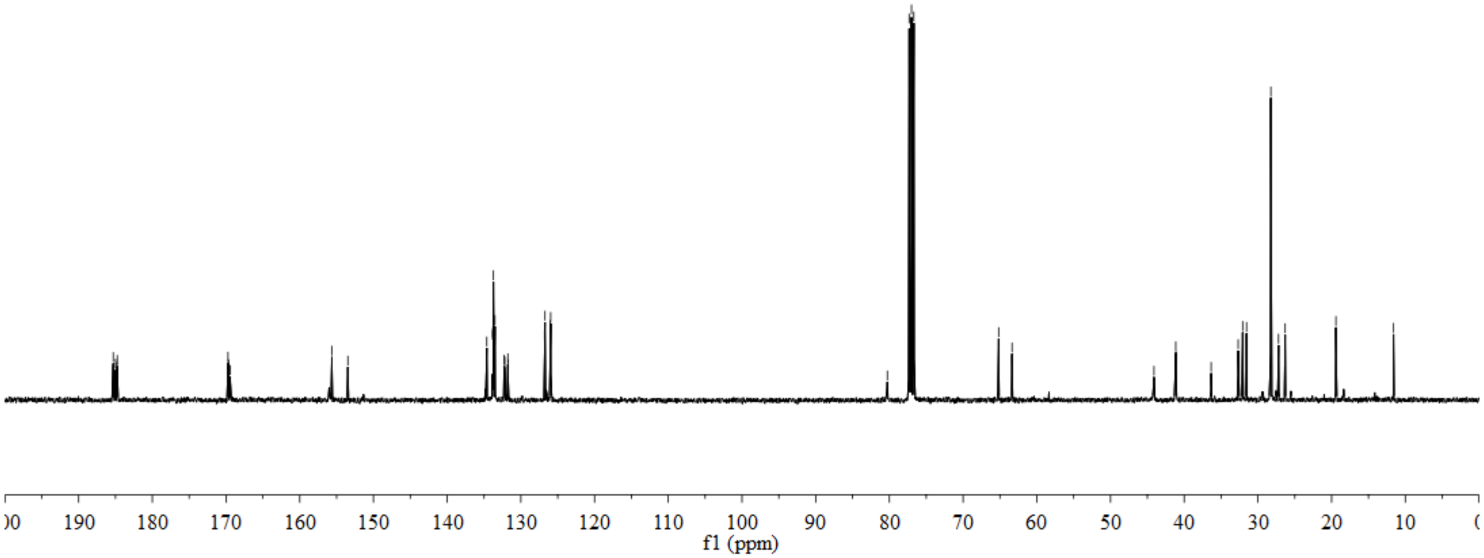

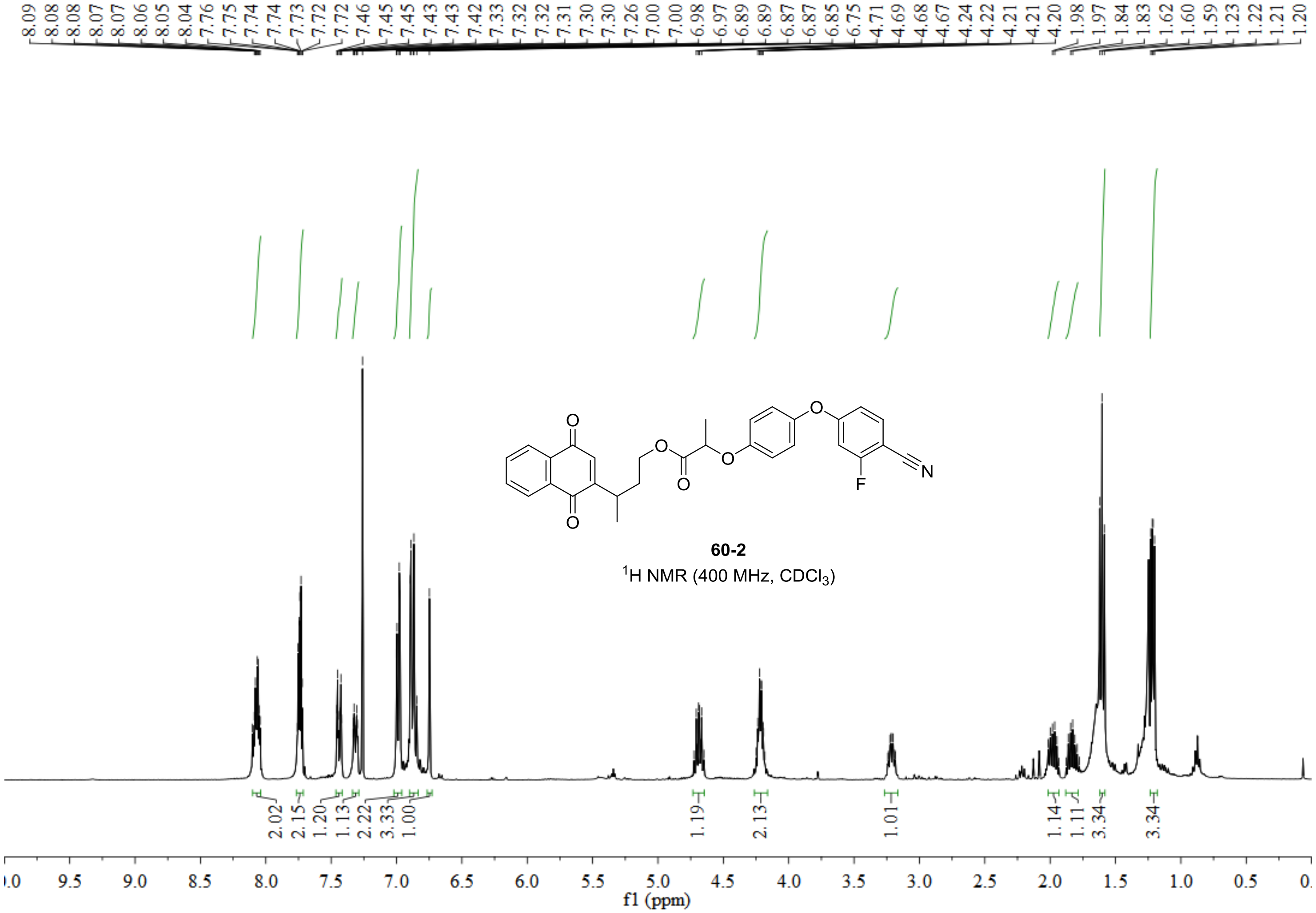

60-2

^{1}H NMR (400 MHz, $CDCl_3$)

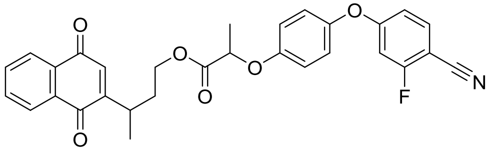

60-2

^{13}C NMR (100 MHz, $CDCl_3$)

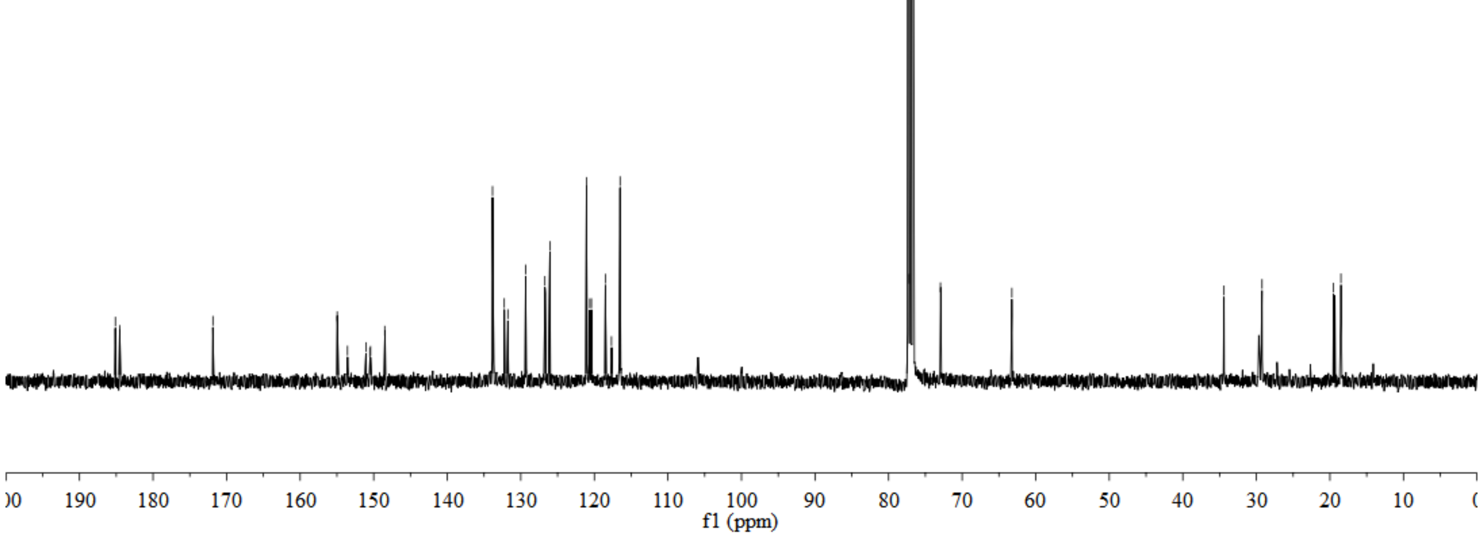

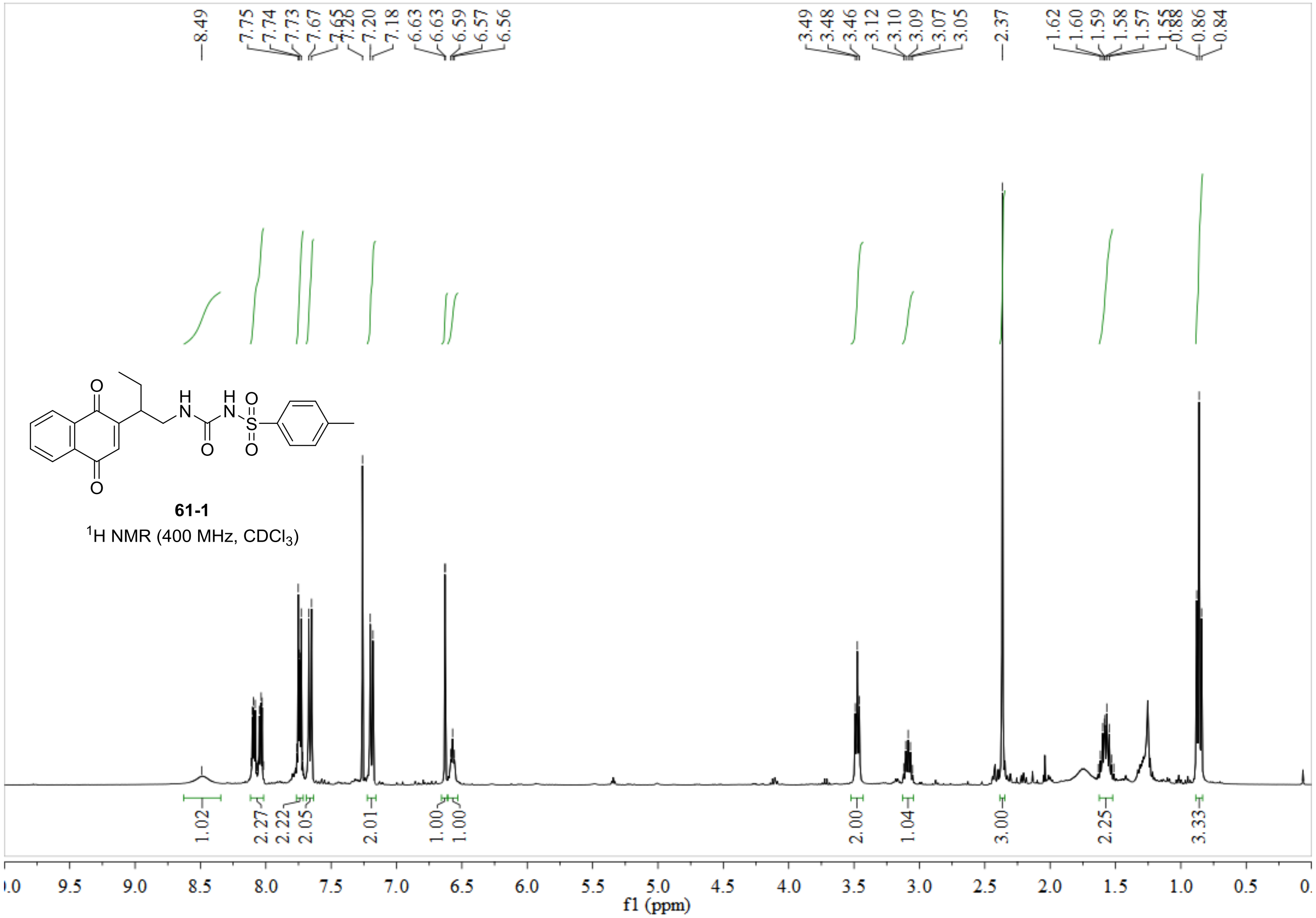

61-1

^{1}H NMR (400 MHz, $CDCl_3$)

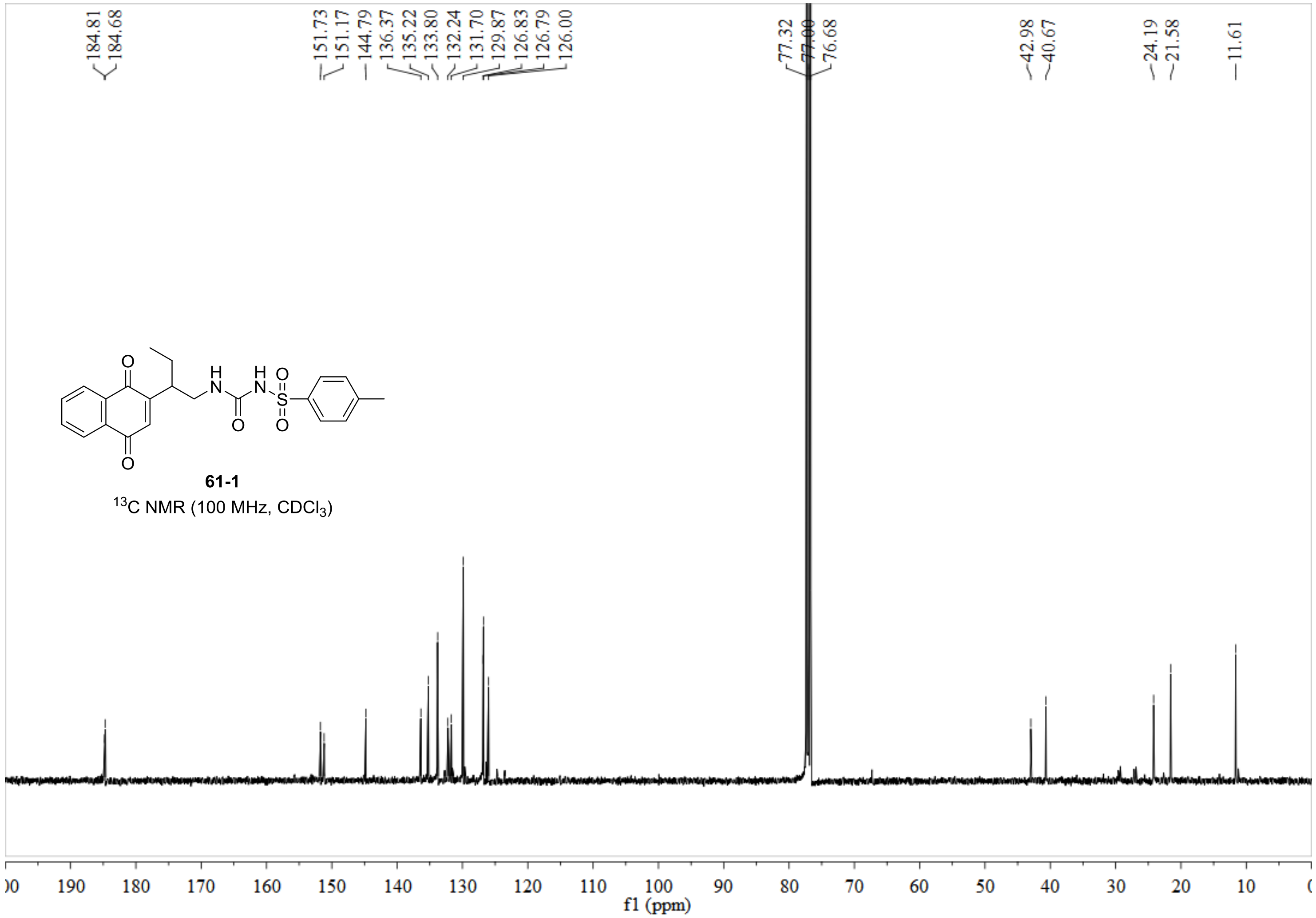
184.81
184.68
151.73
151.17
144.79
136.37
135.22
133.80
132.24
131.70
129.87
126.83
126.79
126.00
77.32
77.00
76.68
42.98
40.67
24.19
21.58
11.61
O
H
N
H
N
S
O
O
O
O
61-1
^{13}C NMR (100 MHz, $CDCl_3$)
190 180 170 160 150 140 130 120 110 100 90 80 70 60 50 40 30 20 10
f1 (ppm)

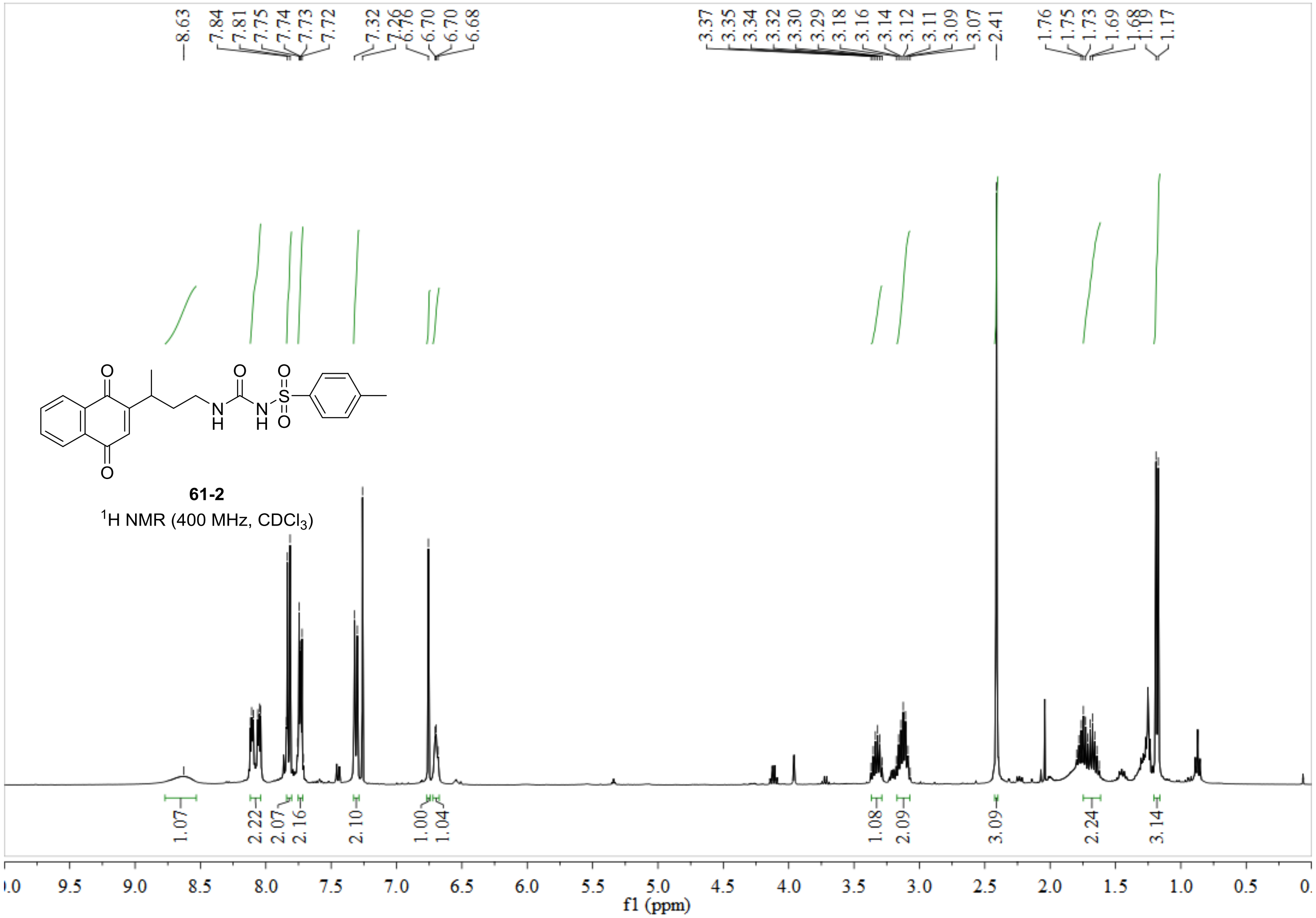

61-2
^{1}H NMR (400 MHz, CDCl$_3$)
f1 (ppm)

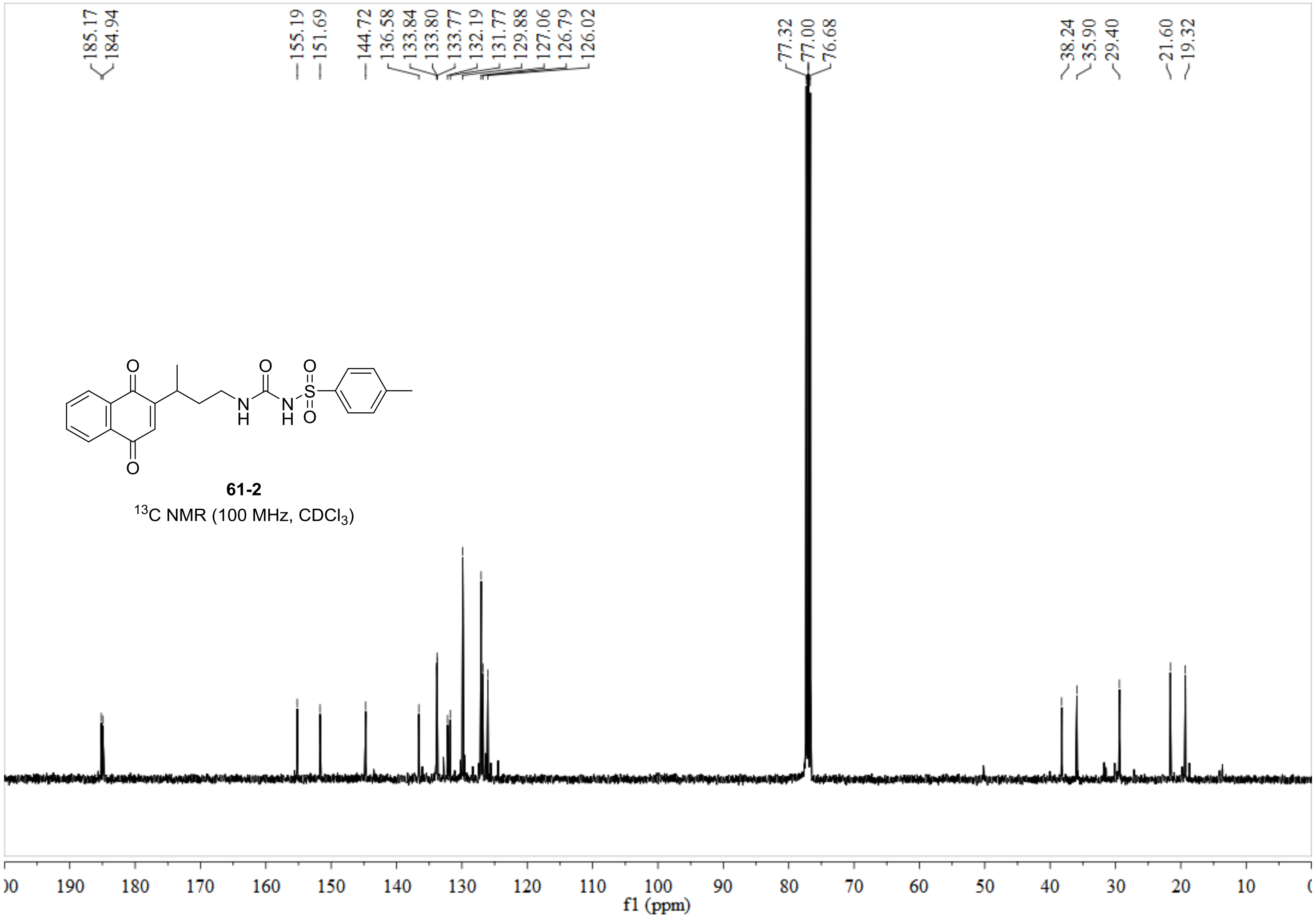

61-2

^{13}C NMR (100 MHz, $CDCl_3$)

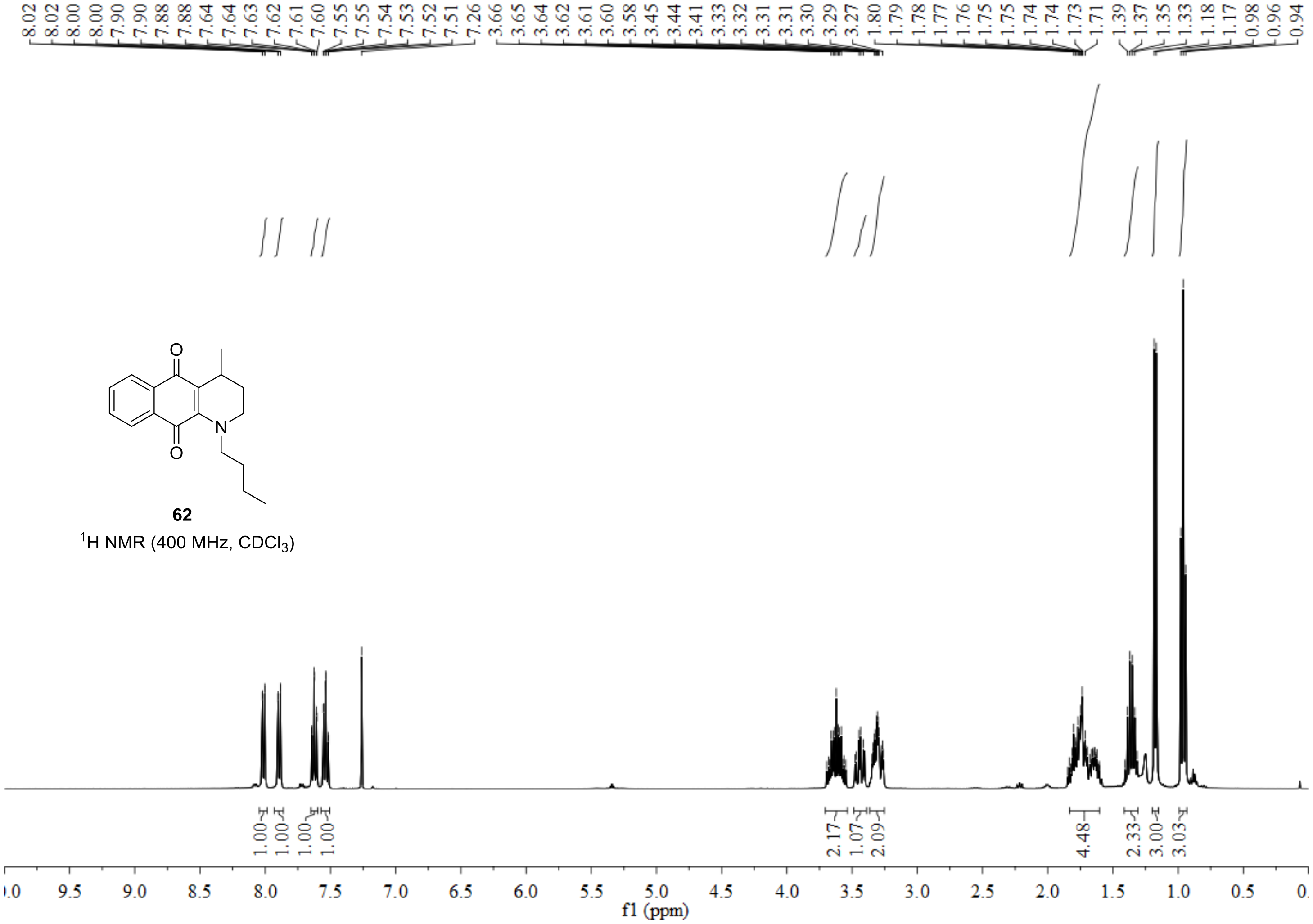
62
^{1}H NMR (400 MHz, $CDCl_3$)
f1 (ppm)

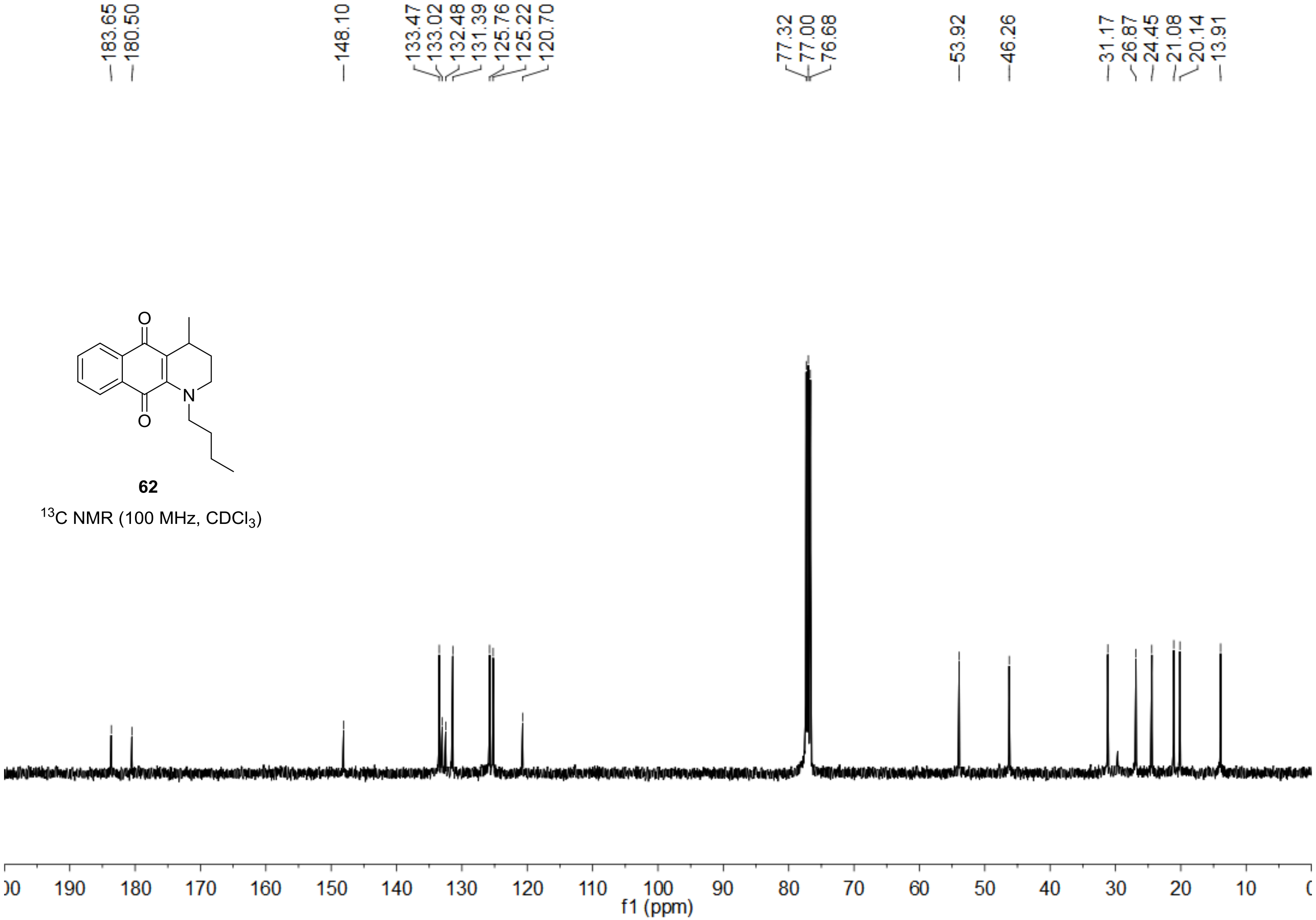

62

^{13}C NMR (100 MHz, $CDCl_3$)

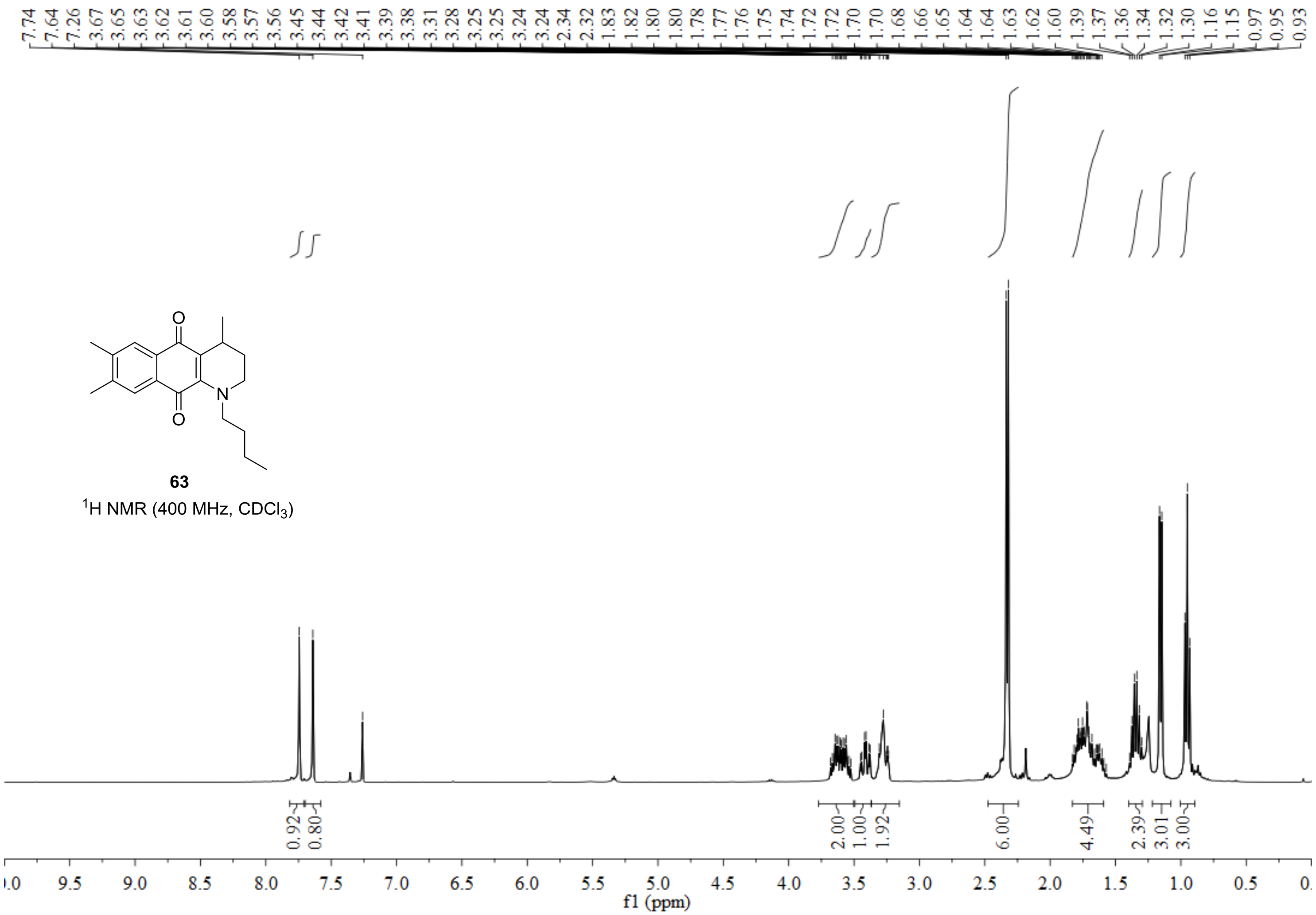

63

^{1}H NMR (400 MHz, $CDCl_3$)

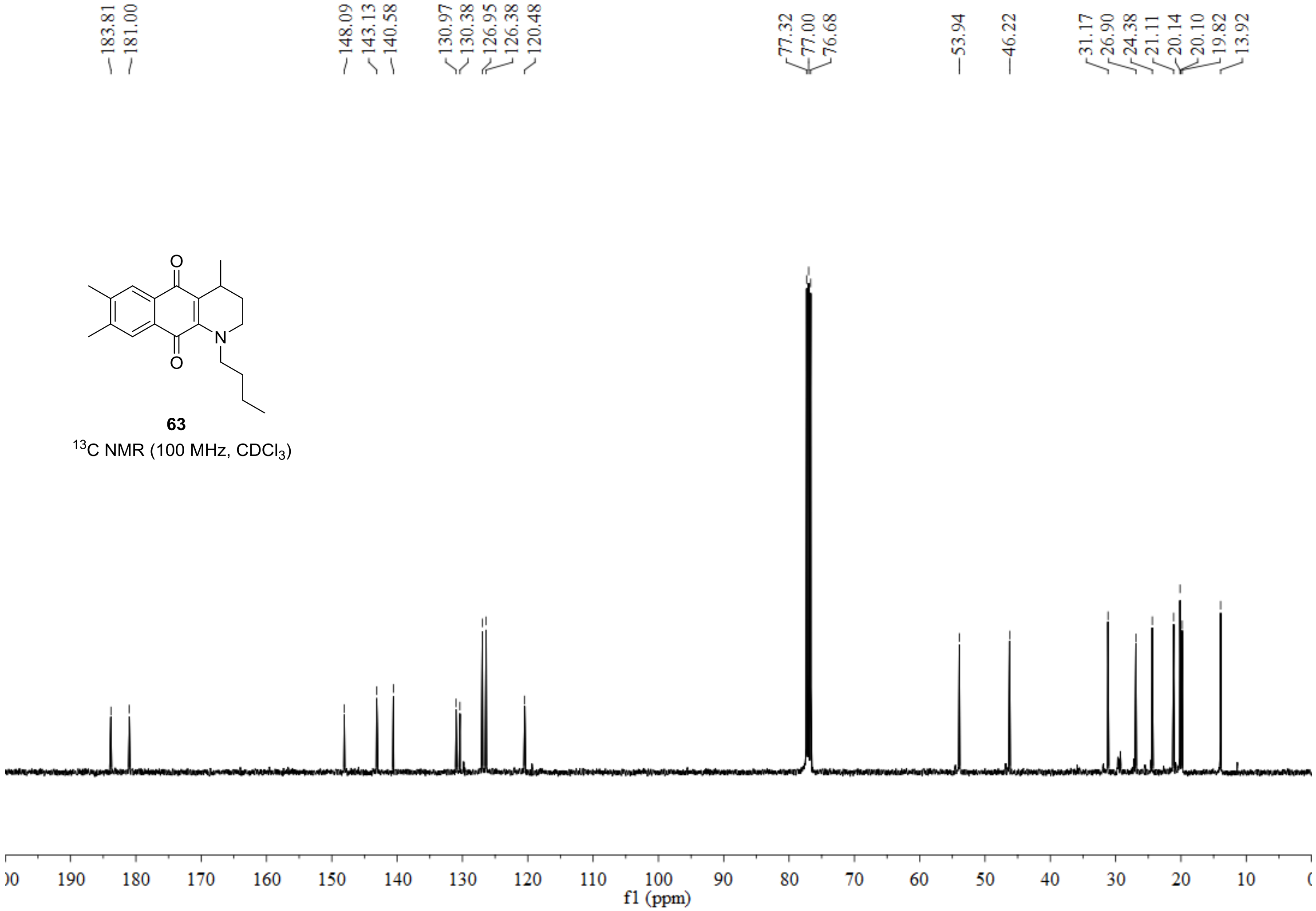

63

^{13}C NMR (100 MHz, $CDCl_3$)

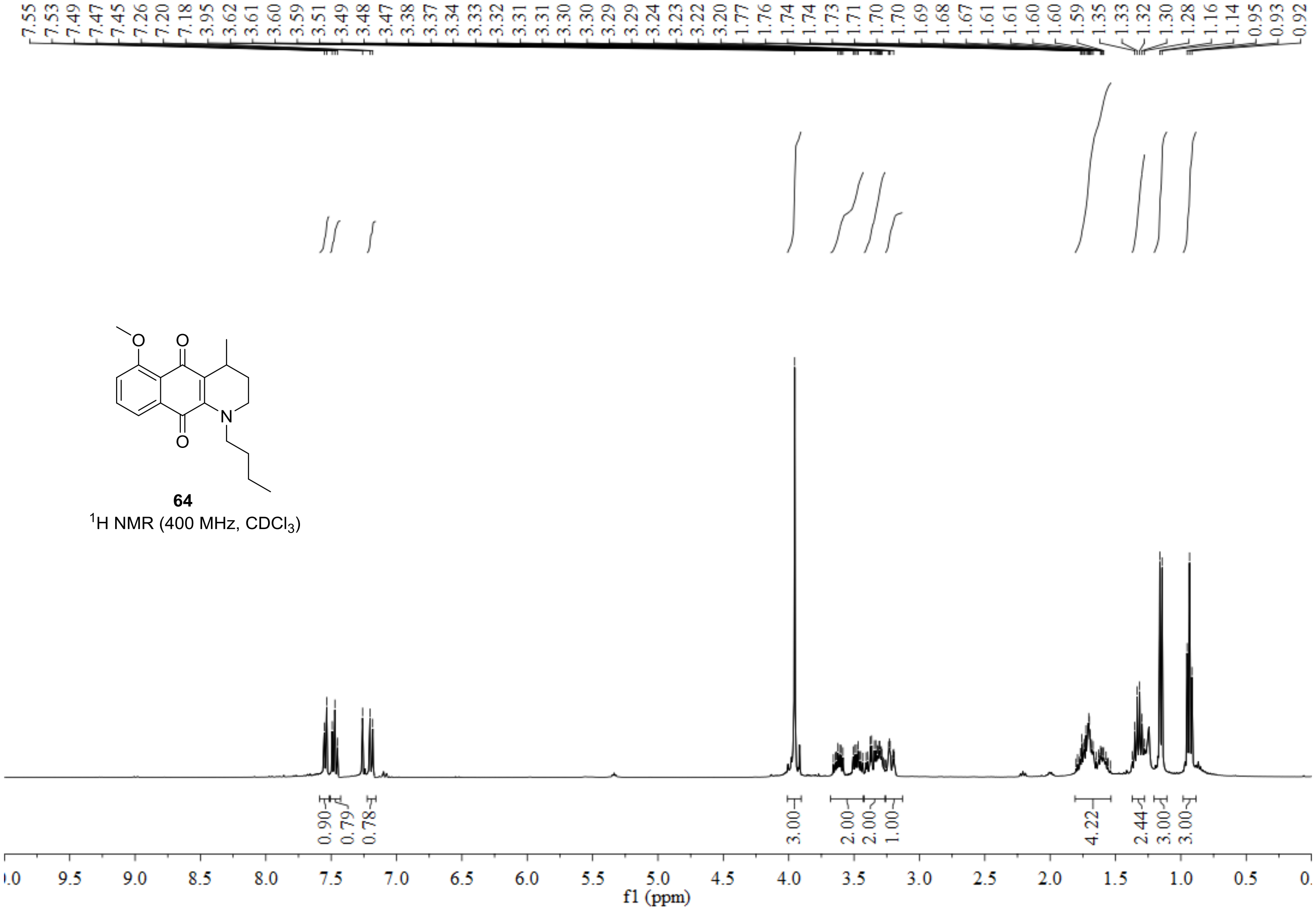
7.55 7.53 7.49 7.47 7.45 7.26 7.20 7.18 3.95 3.62 3.61 3.60 3.59 3.51 3.49 3.48 3.47 3.38 3.37 3.34 3.33 3.32 3.31 3.31 3.30 3.30 3.29 3.29 3.24 3.23 3.22 3.20 1.77 1.76 1.74 1.74 1.73 1.71 1.70 1.70 1.69 1.68 1.67 1.61 1.61 1.60 1.60 1.59 1.35 1.33 1.32 1.30 1.28 1.16 1.14 0.95 0.93 0.92
O
O
O
N
64
^{1}H NMR (400 MHz, $CDCl_3$)
0.90
0.79
0.78
3.00
2.00
2.00
1.00
4.22
2.44
3.00
3.00
f1 (ppm)

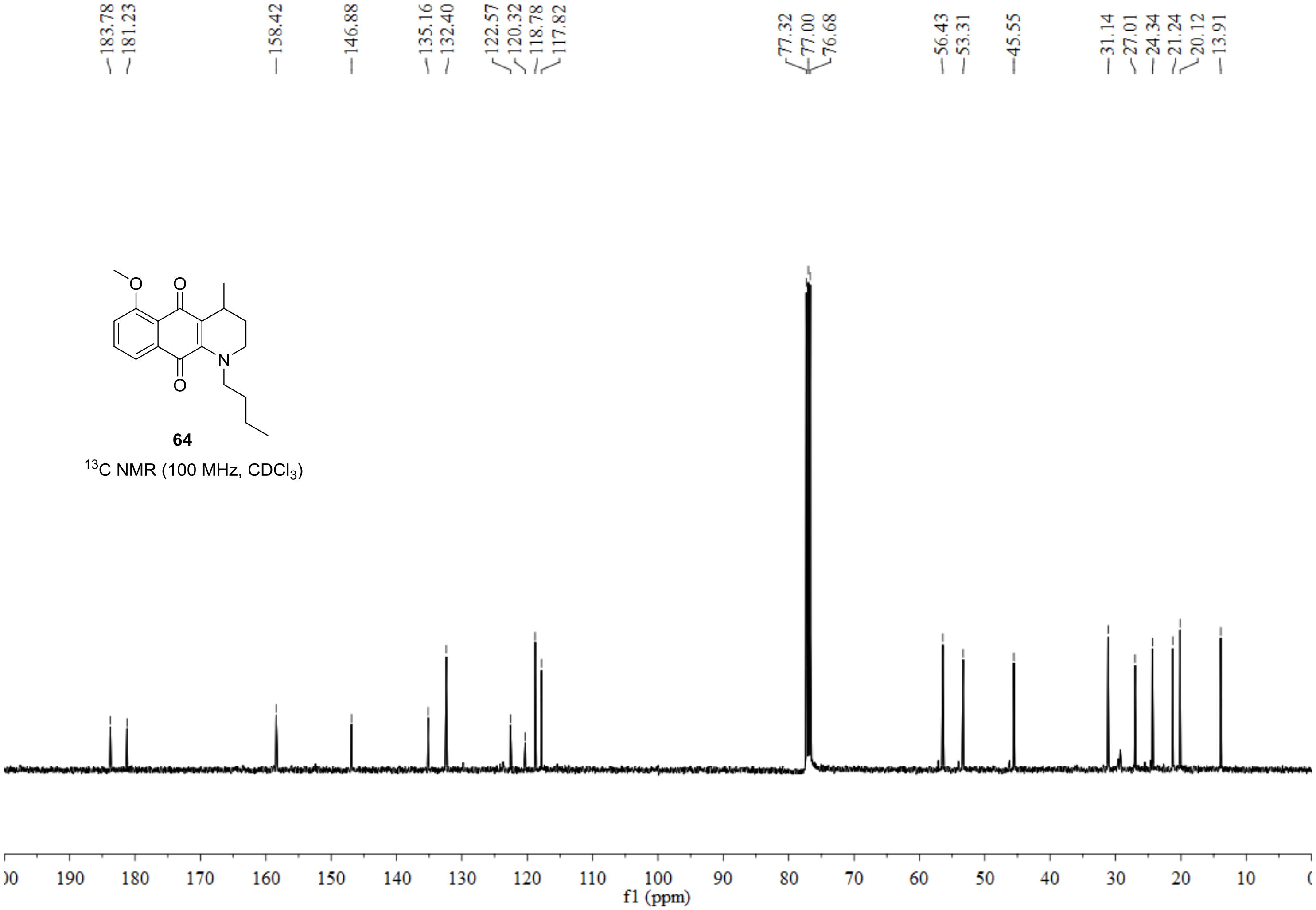

64

^{13}C NMR (100 MHz, $CDCl_3$)

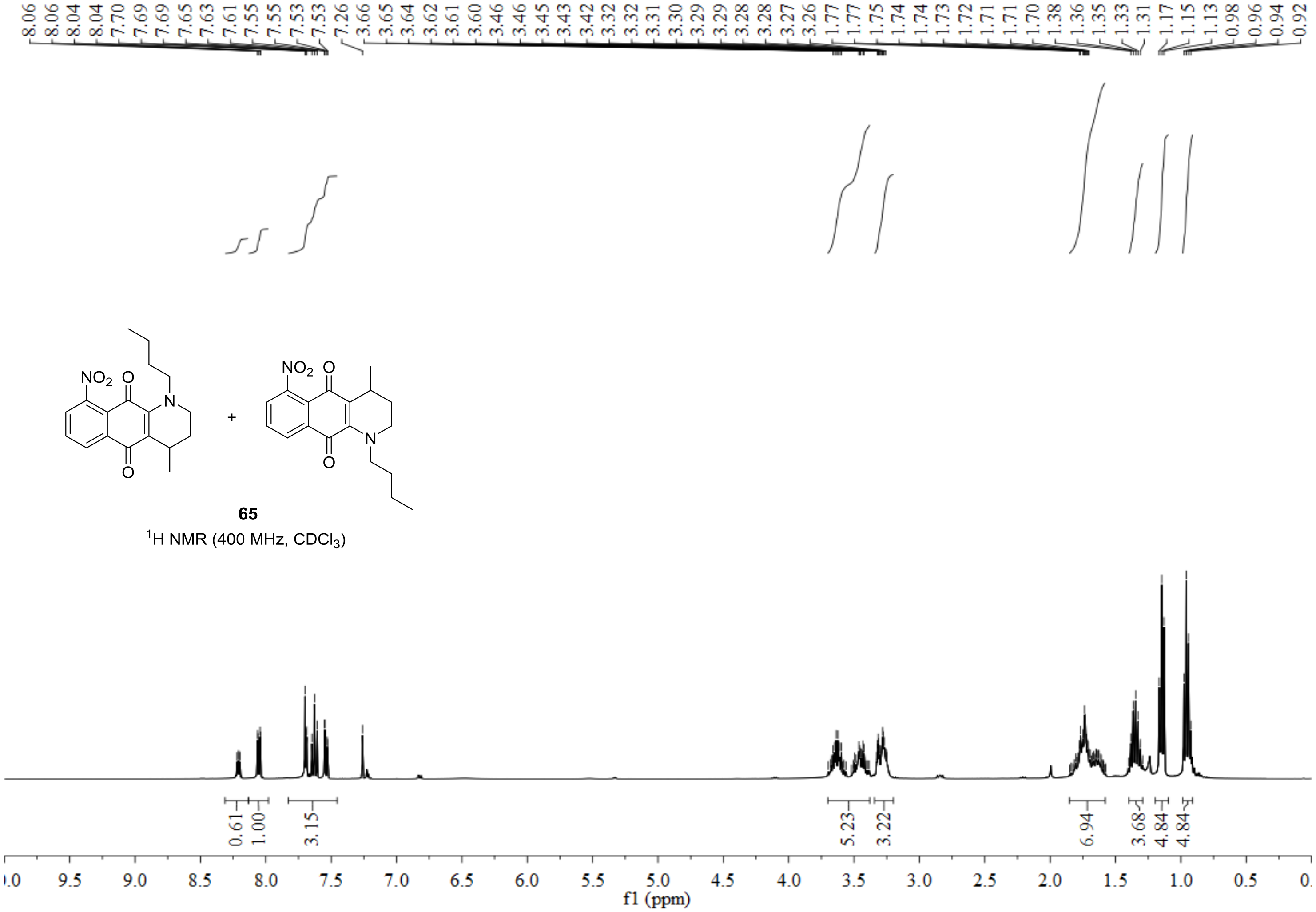
8.06 8.06 8.04 8.04 7.70 7.69 7.69 7.65 7.63 7.61 7.55 7.55 7.53 7.53 7.26 3.66 3.65 3.64 3.62 3.61 3.60 3.46 3.46 3.45 3.43 3.42 3.32 3.32 3.31 3.30 3.29 3.29 3.28 3.28 3.27 3.26 1.77 1.77 1.75 1.74 1.74 1.73 1.72 1.71 1.71 1.70 1.38 1.36 1.35 1.33 1.31 1.17 1.15 1.13 0.98 0.96 0.94 0.92
NO2
O
N
+
65
1H NMR (400 MHz, CDCl3)
0.61 1.00 3.15 5.23 3.22 6.94 3.68 4.84 4.84
9.5 9.0 8.5 8.0 7.5 7.0 6.5 6.0 5.5 5.0 4.5 4.0 3.5 3.0 2.5 2.0 1.5 1.0 0.5
f1 (ppm)

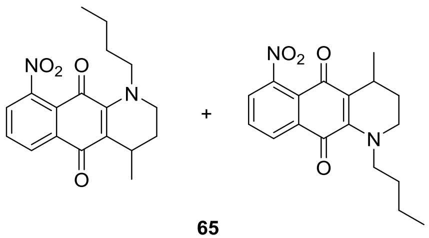

65

^{13}C NMR (100 MHz, $CDCl_3$)

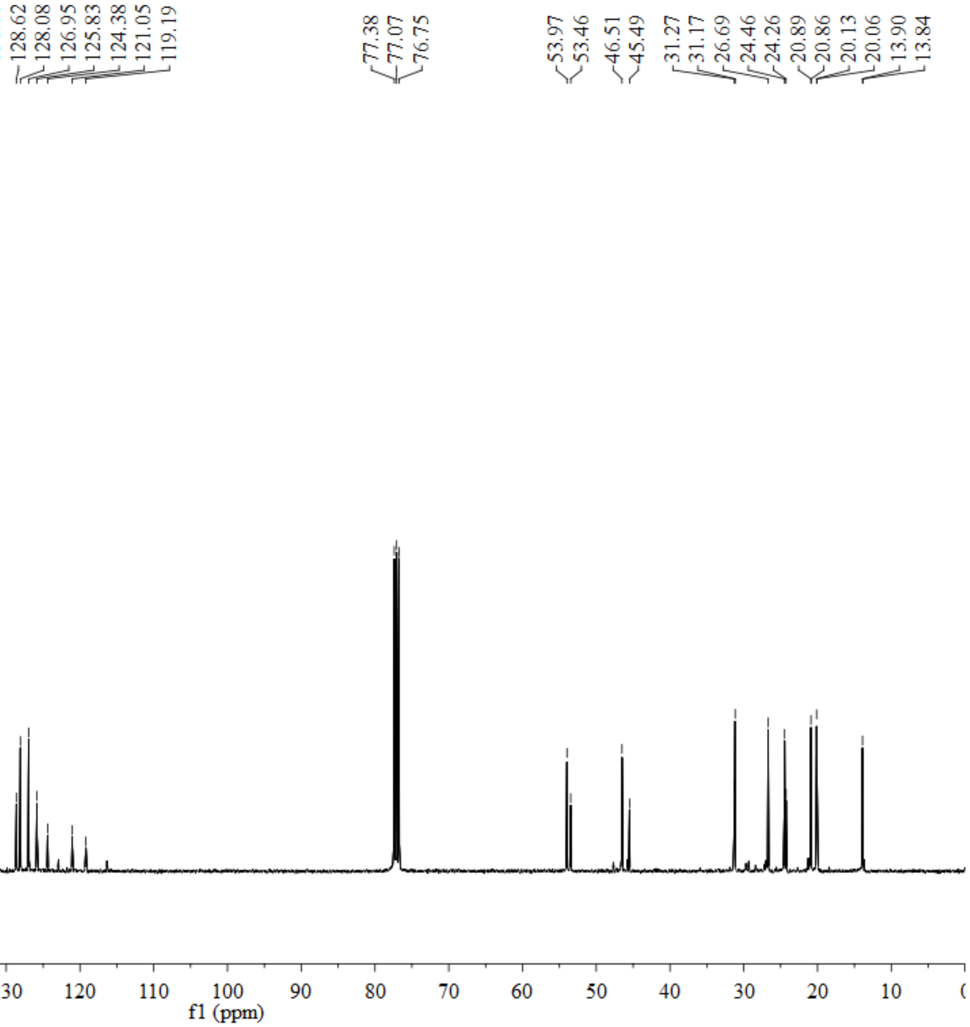

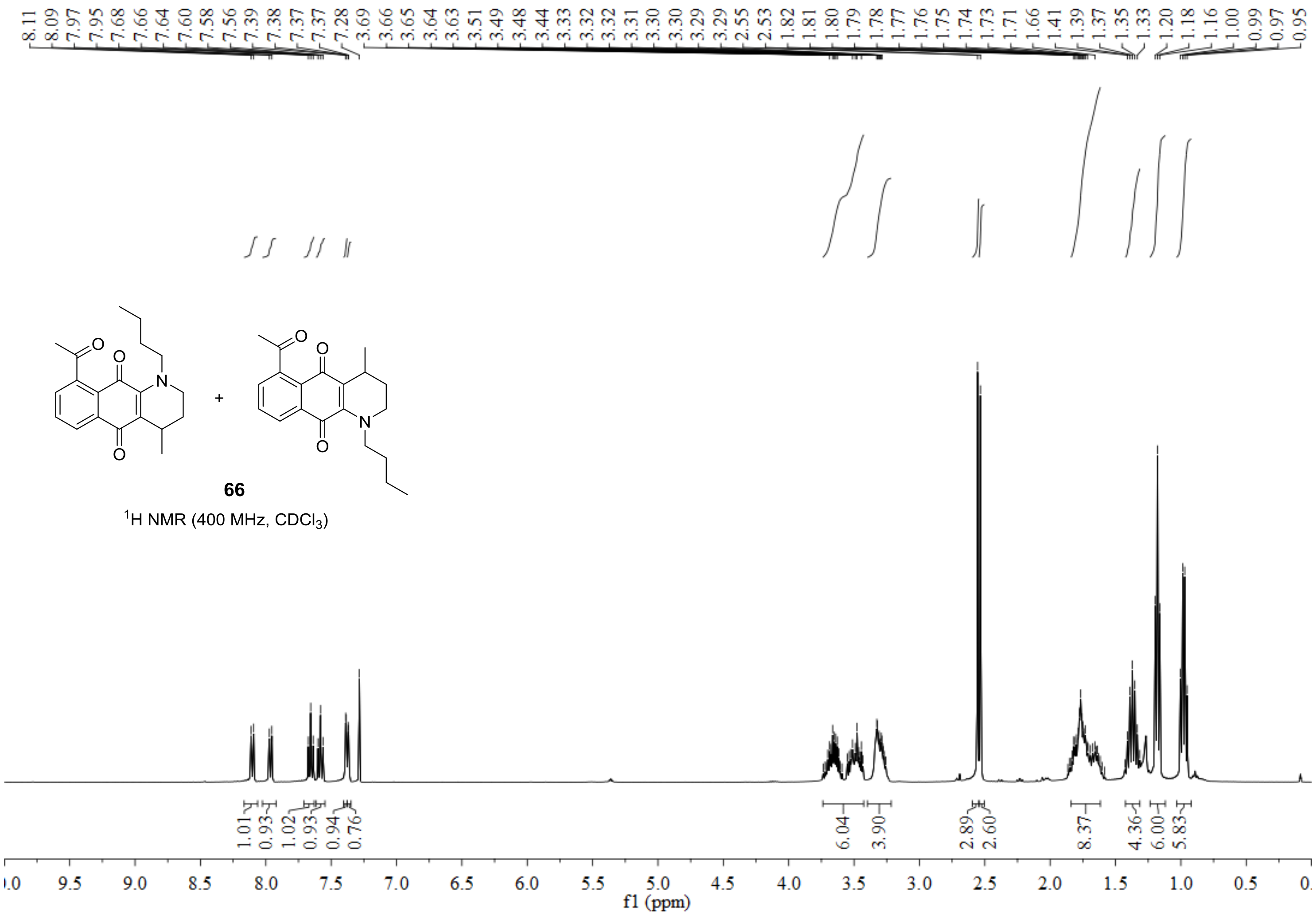

8.11 8.09 7.97 7.95 7.68 7.66 7.64 7.60 7.58 7.56 7.39 7.38 7.37 7.37 7.28 3.69 3.66 3.65 3.64 3.63 3.51 3.49 3.48 3.44 3.33 3.32 3.32 3.31 3.30 3.30 3.29 3.29 2.55 2.53 1.82 1.81 1.80 1.79 1.78 1.77 1.76 1.75 1.74 1.73 1.71 1.66 1.41 1.39 1.37 1.35 1.33 1.20 1.18 1.16 1.00 0.99 0.97 0.95
66
^{1}H NMR (400 MHz, CDCl$_3$)
1.01 0.93 1.02 0.93 0.94 0.76 6.04 3.90 2.89 2.60 8.37 4.36 6.00 5.83
f1 (ppm)

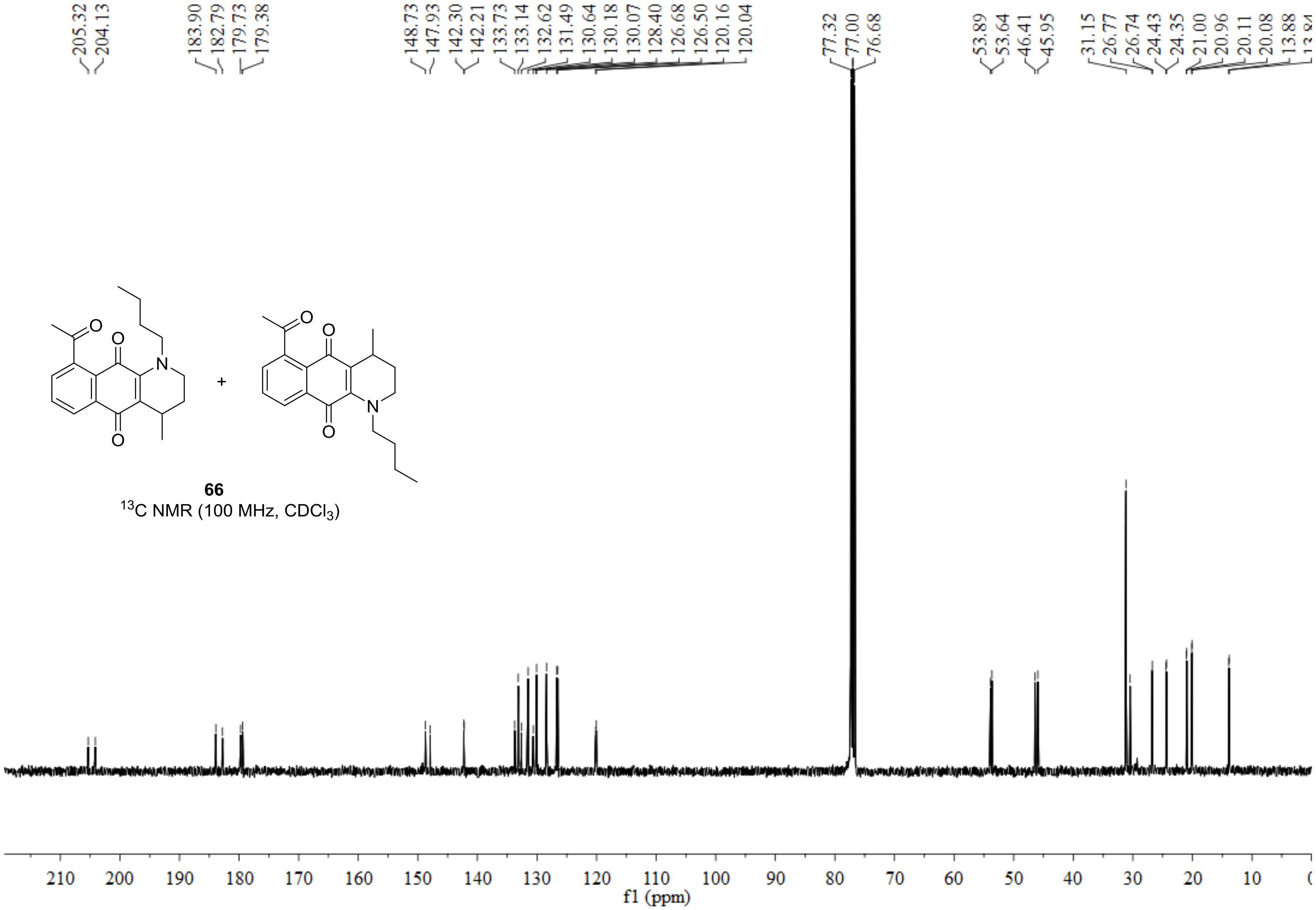

66
^{13}C NMR (100 MHz, $CDCl_3$)

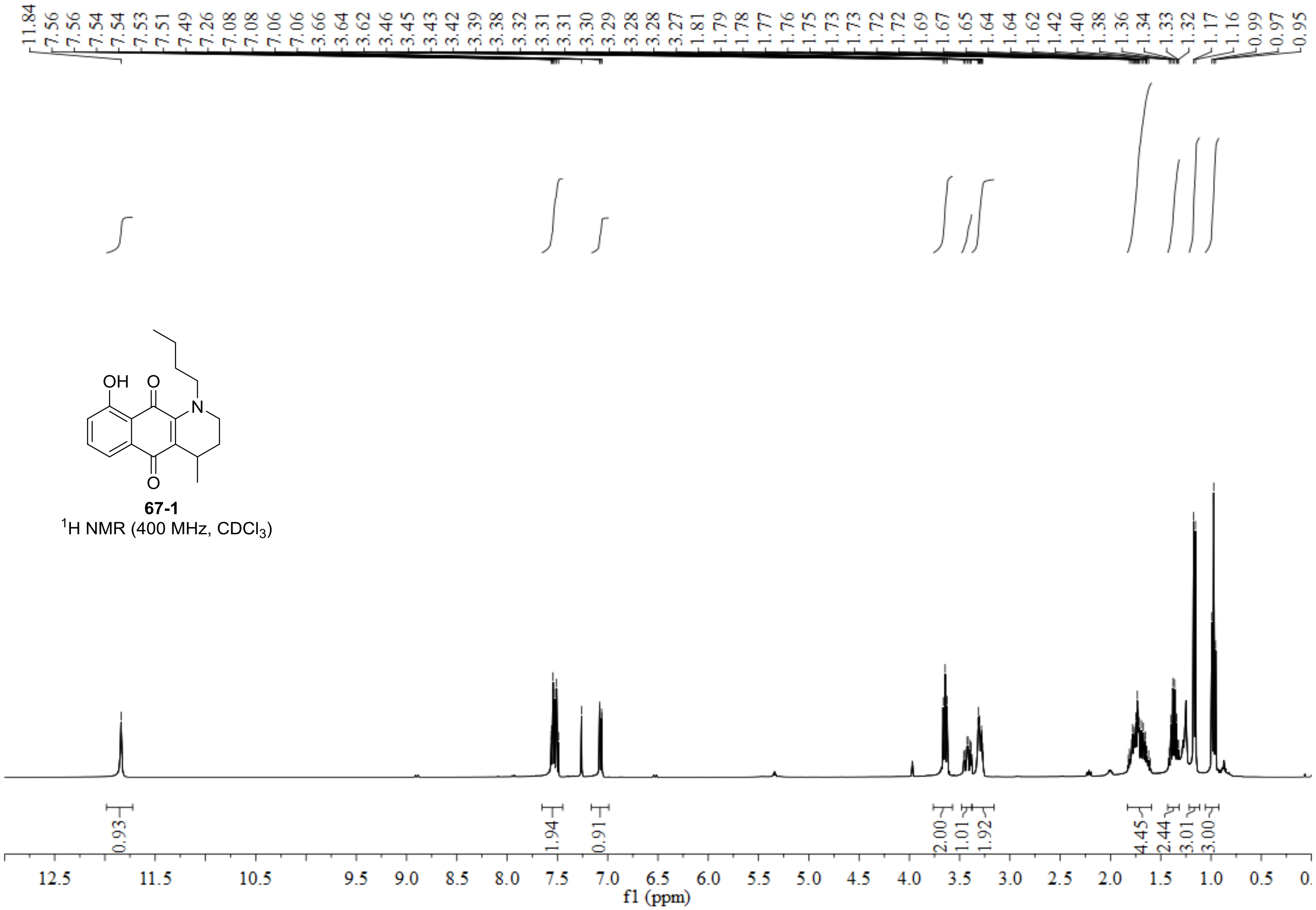

67-1

^{1}H NMR (400 MHz, $CDCl_3$)

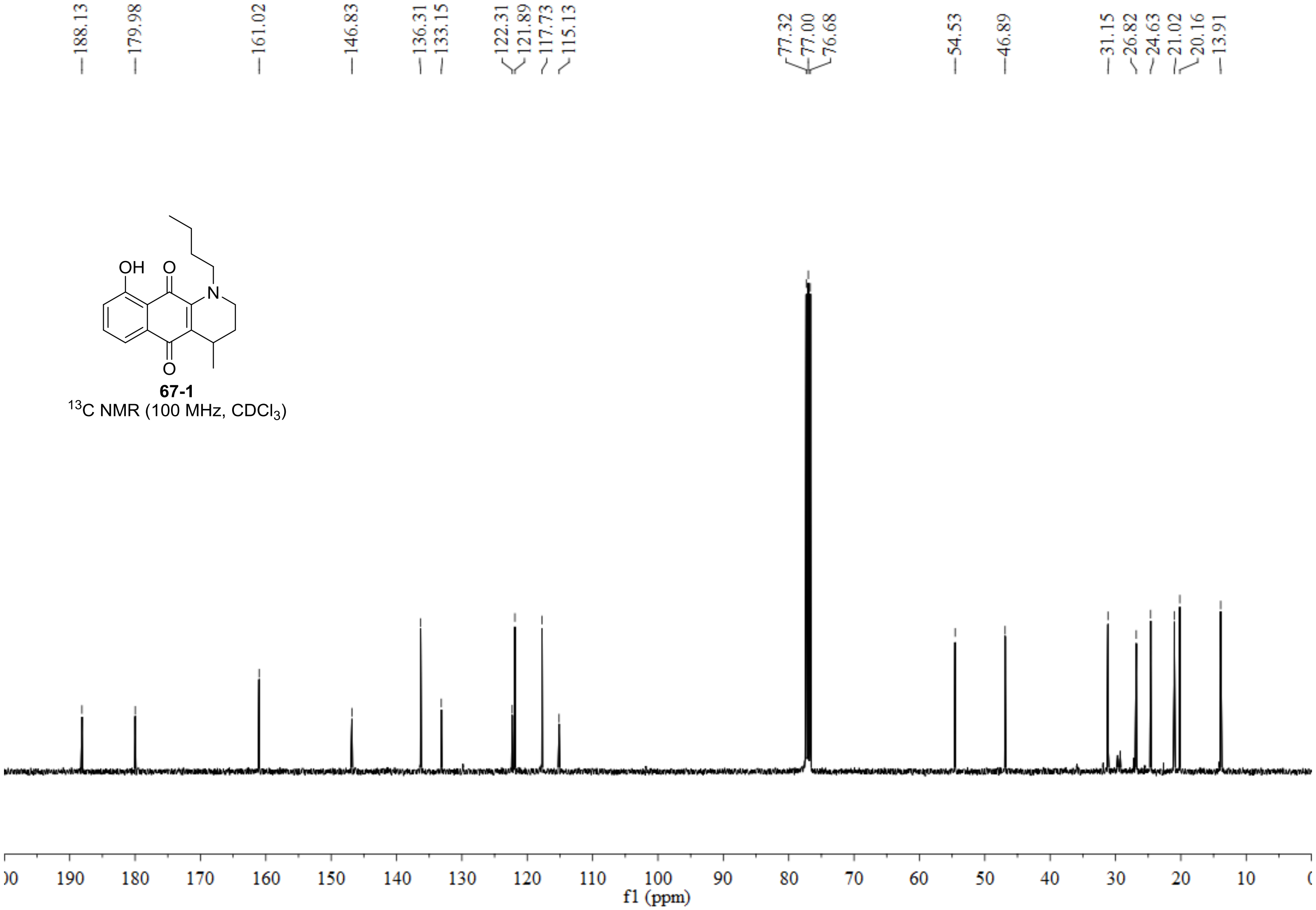

188.13
179.98
161.02
146.83
136.31
133.15
122.31
121.89
117.73
115.13
77.32
77.00
76.68
54.53
46.89
31.15
26.82
24.63
21.02
20.16
13.91
OH
O
N
O
67-1
^{13}C NMR (100 MHz, $CDCl_3$)
190 180 170 160 150 140 130 120 110 100 90 80 70 60 50 40 30 20 10
f1 (ppm)

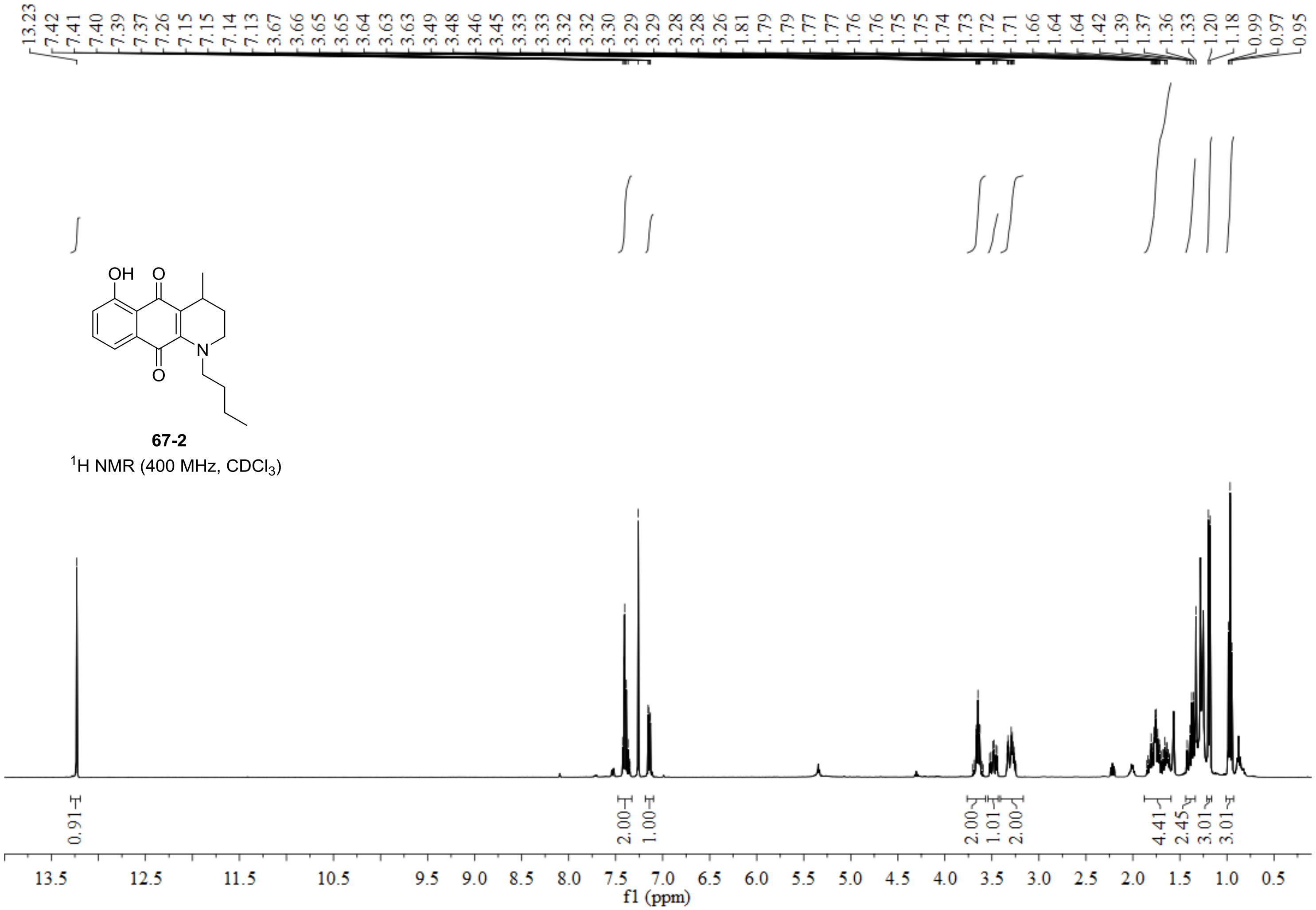

67-2

^{1}H NMR (400 MHz, $CDCl_3$)

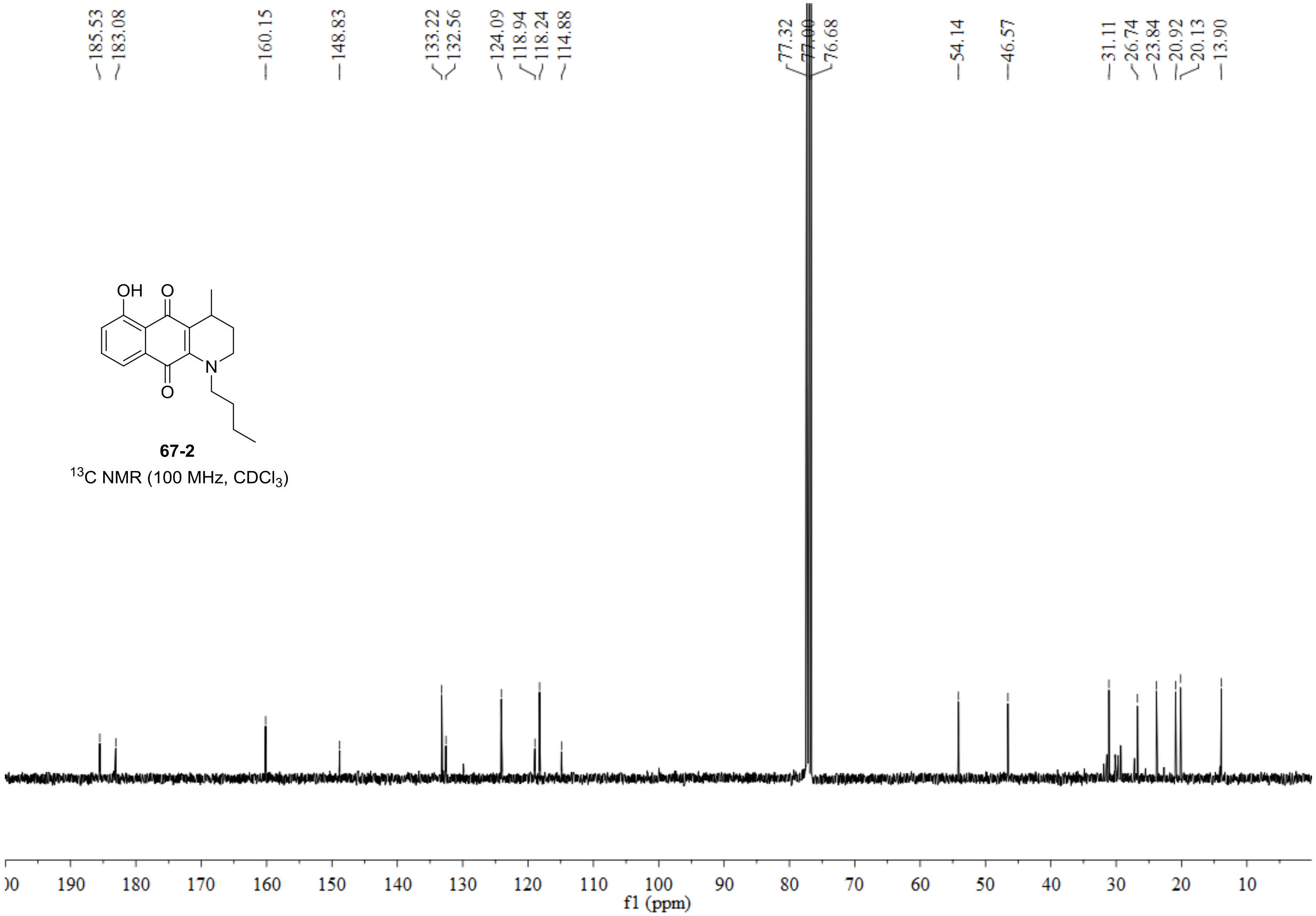

67-2

^{13}C NMR (100 MHz, $CDCl_3$)

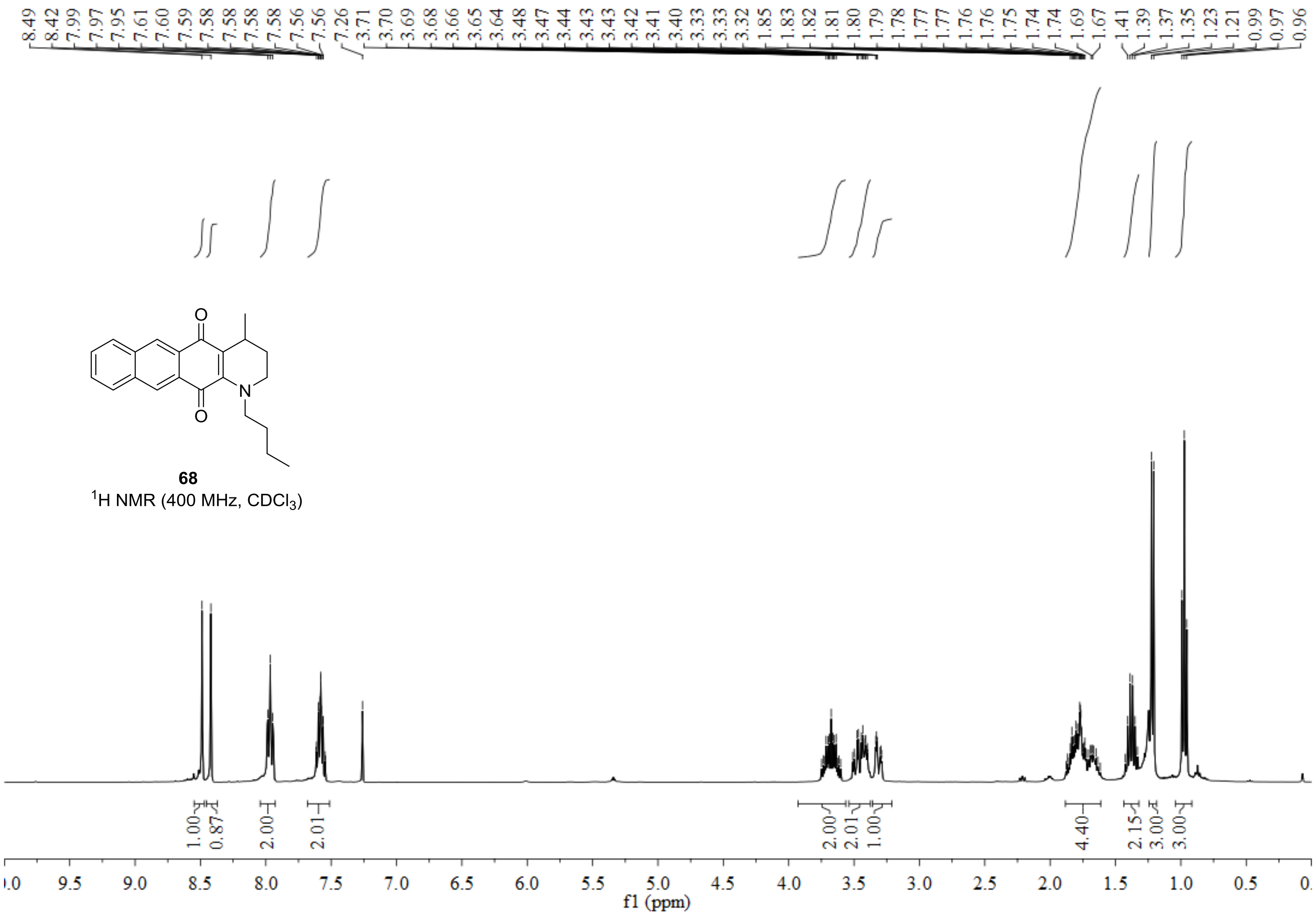

68

^{1}H NMR (400 MHz, $CDCl_3$)

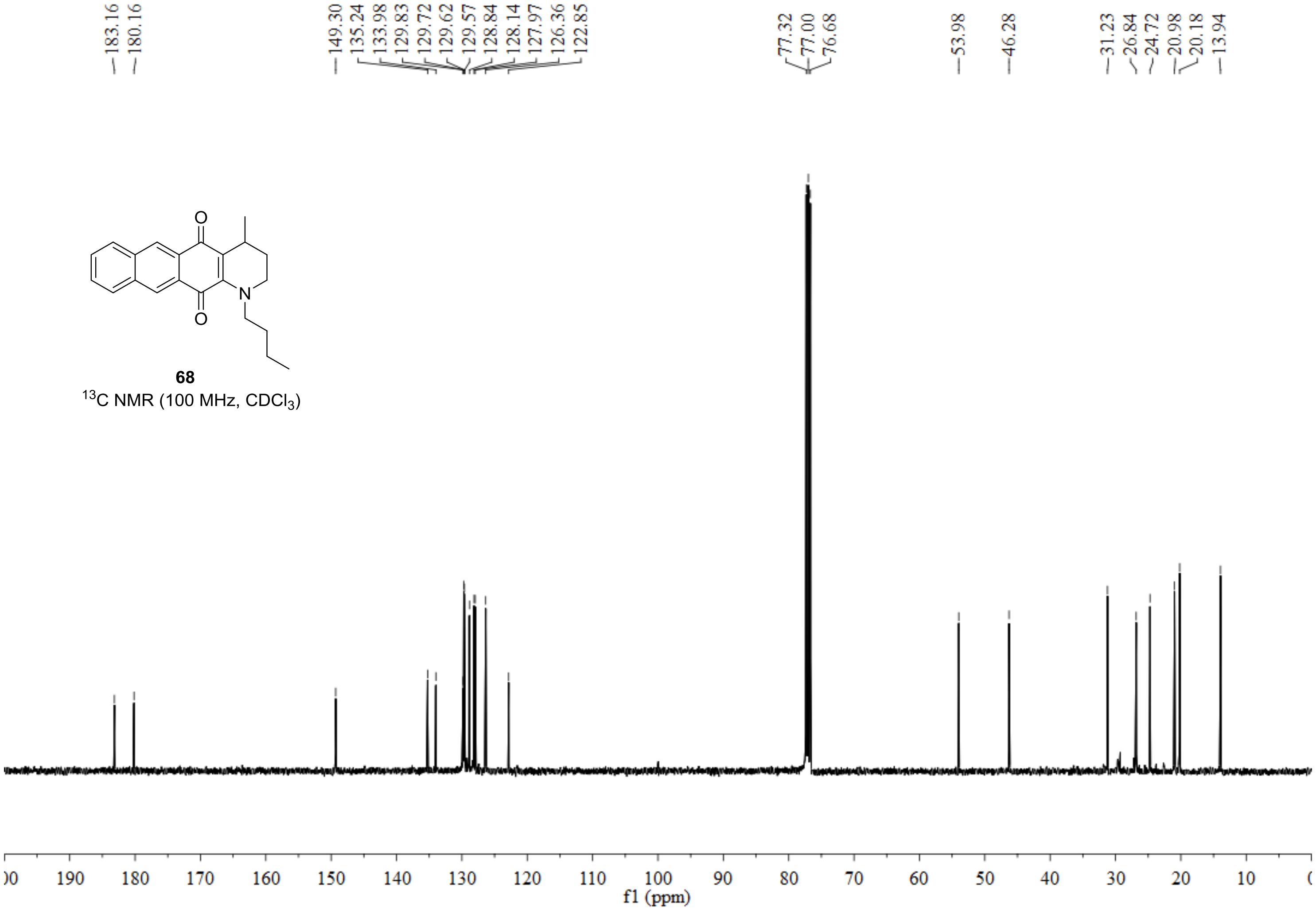

68

^{13}C NMR (100 MHz, $CDCl_3$)

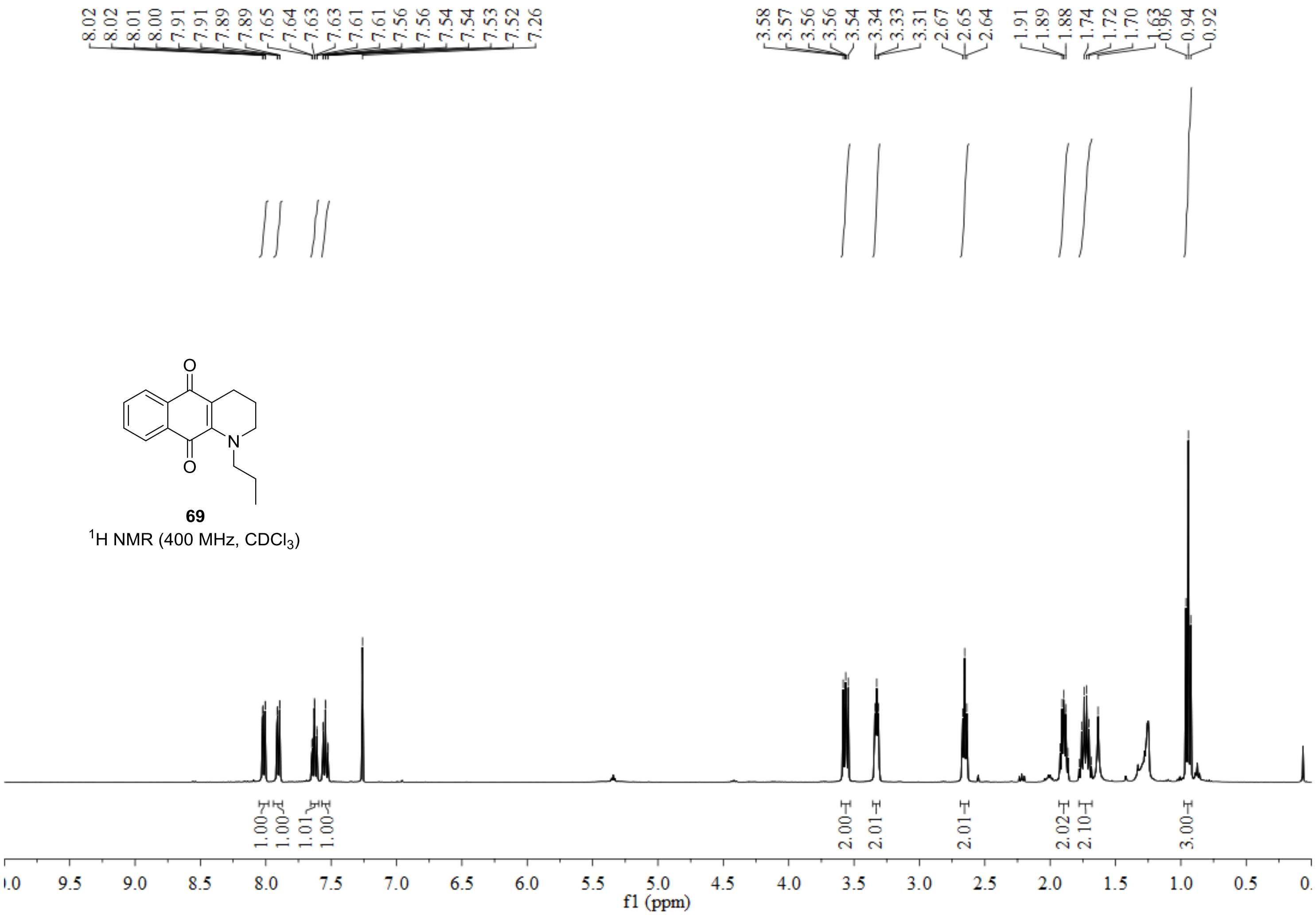

69

^1H NMR (400 MHz, $CDCl_3$)

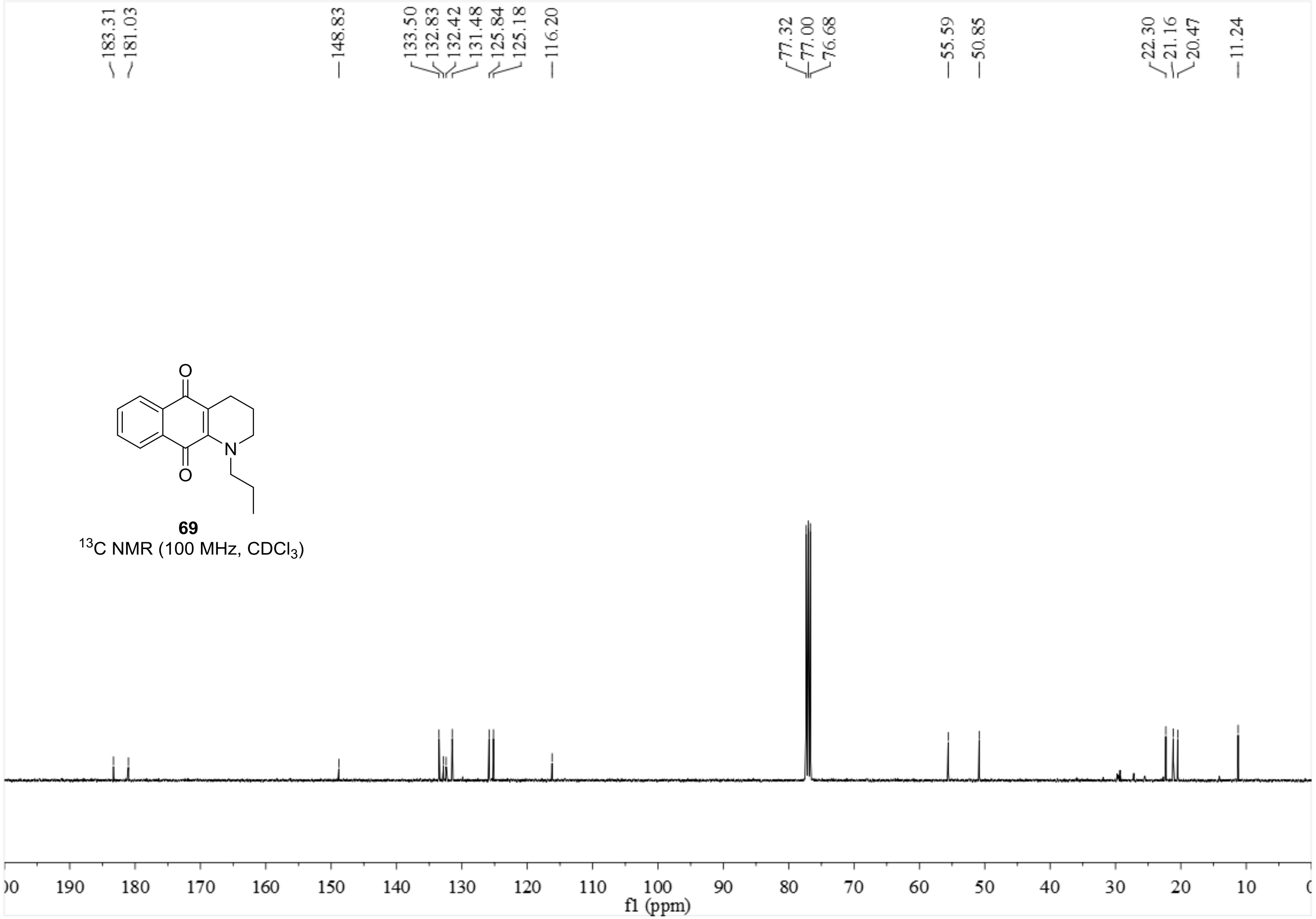
183.31
181.03
148.83
133.50
132.83
132.42
131.48
125.84
125.18
116.20
77.32
77.00
76.68
55.59
50.85
22.30
21.16
20.47
11.24
O
N
O
69
13C NMR (100 MHz, CDCl3)
190
180
170
160
150
140
130
120
110
100
90
80
70
60
50
40
30
20
10
f1 (ppm)

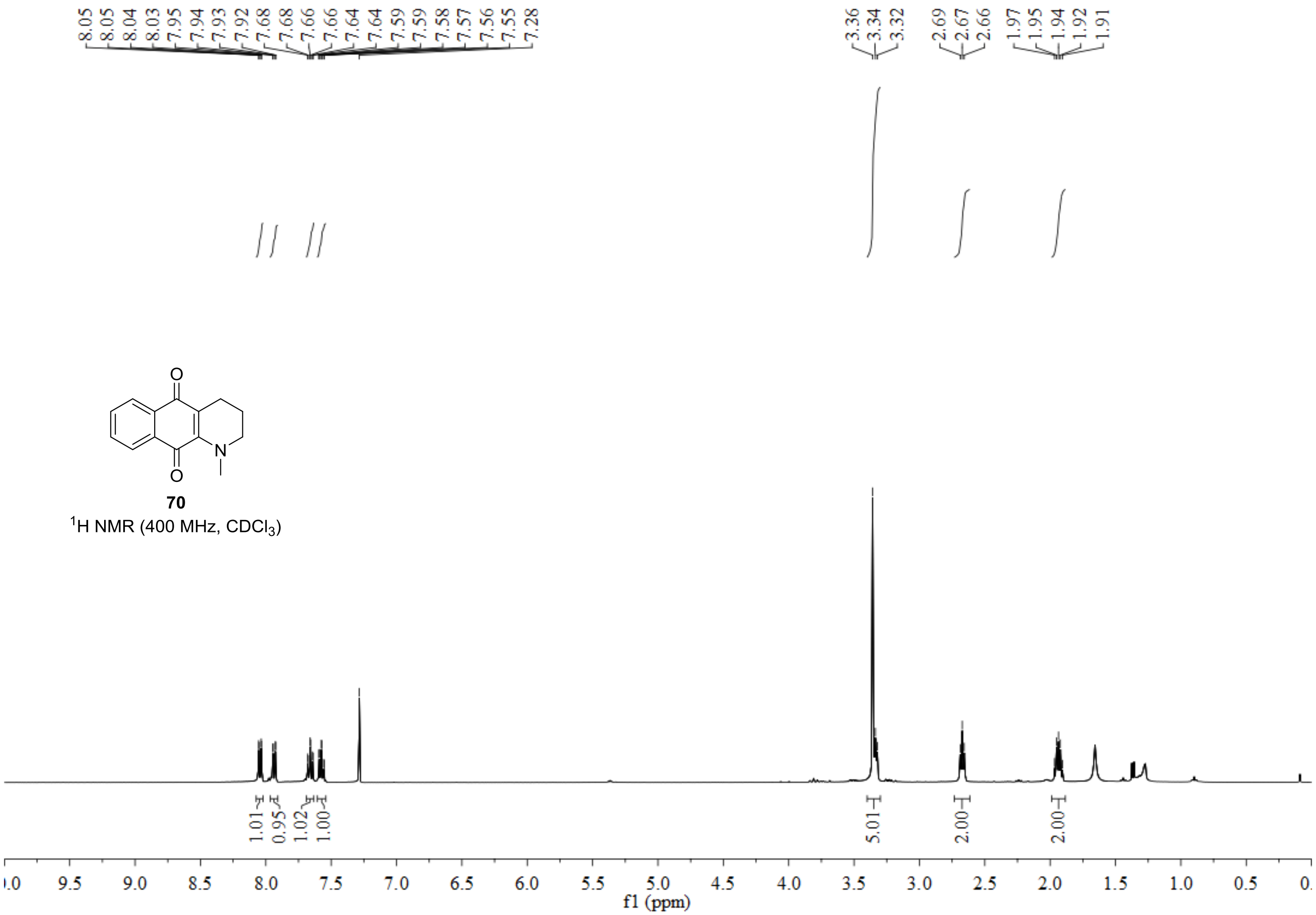

70

^{1}H NMR (400 MHz, $CDCl_3$)

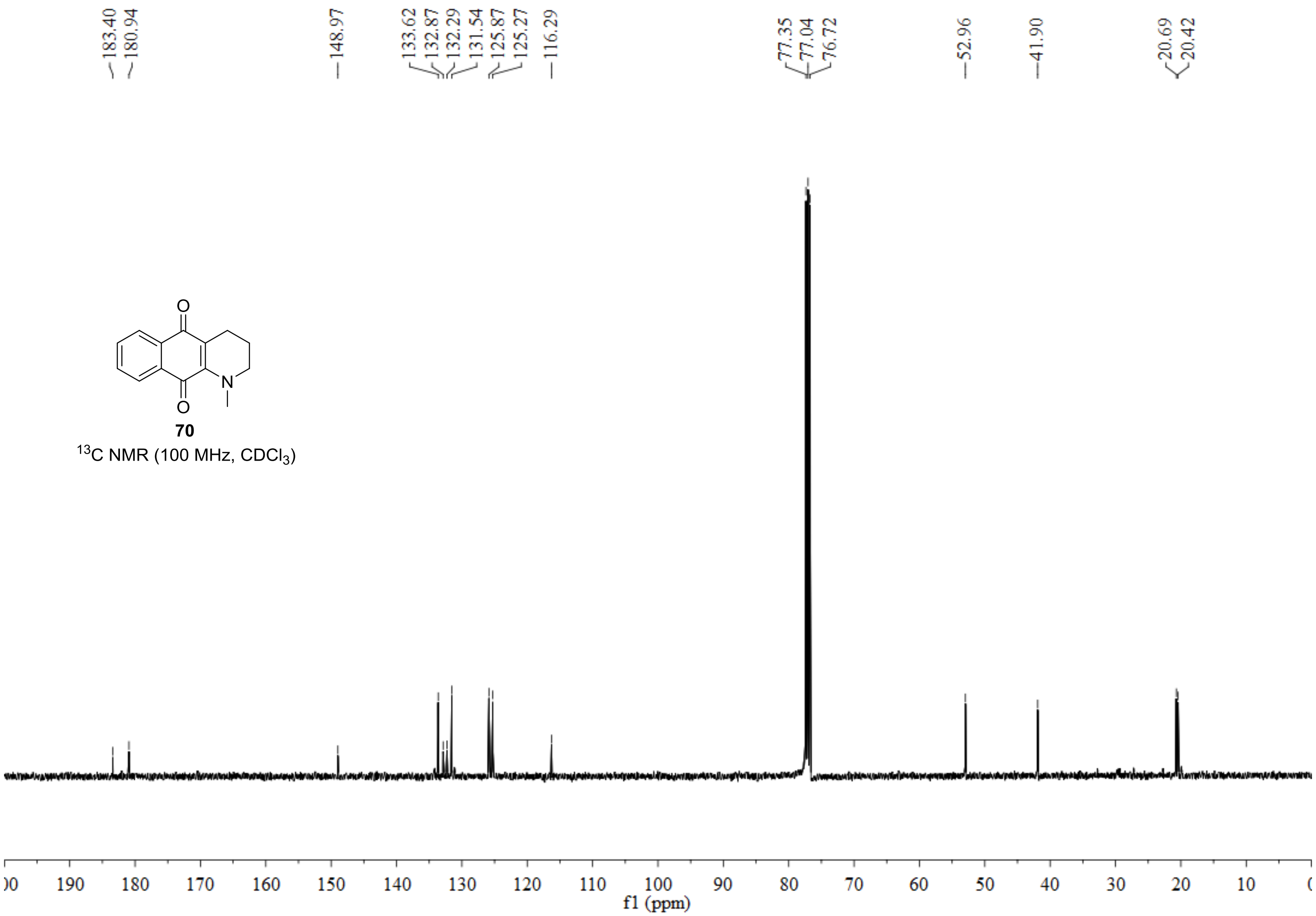

70

^{13}C NMR (100 MHz, $CDCl_3$)

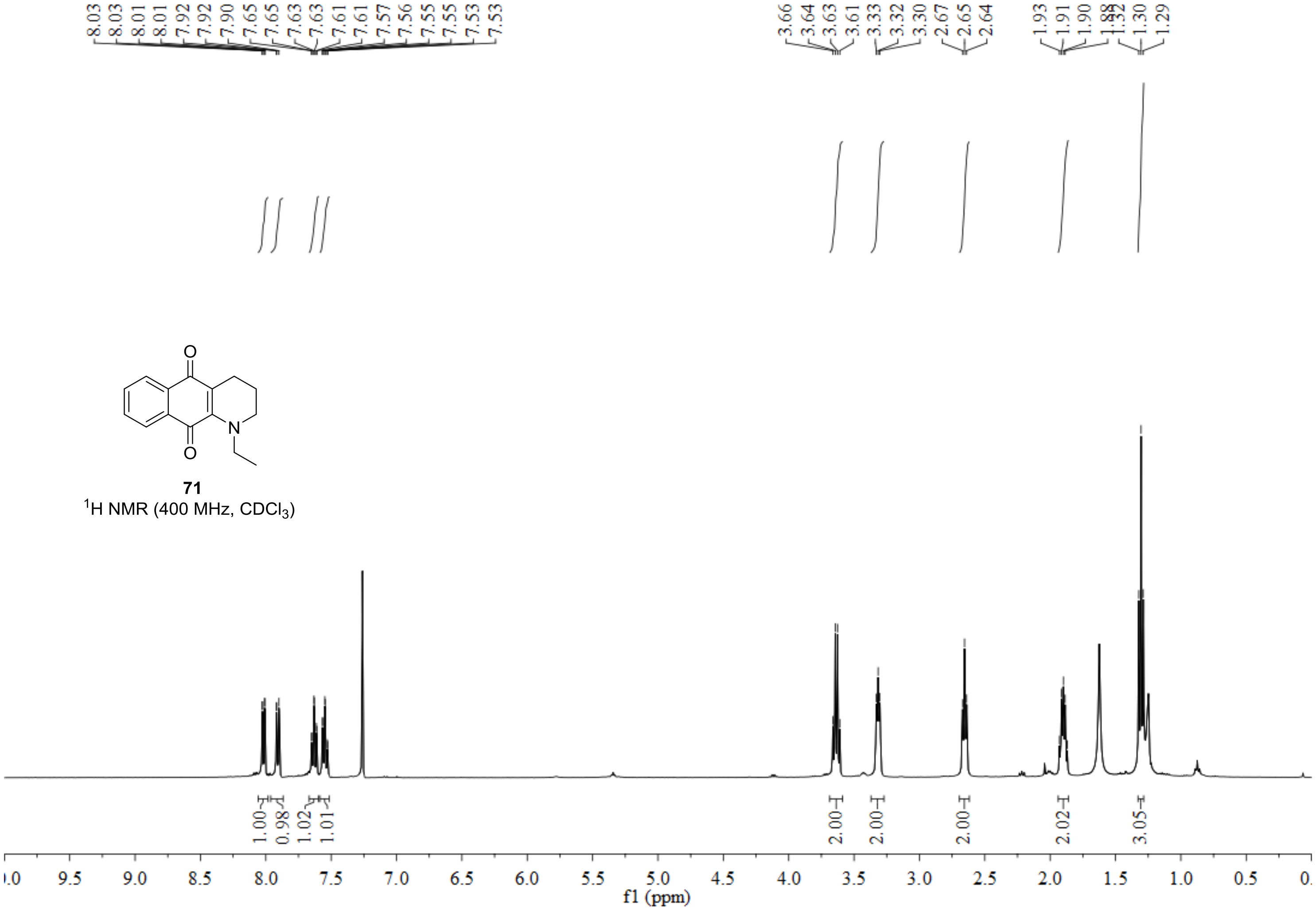
8.03 8.03 8.01 8.01 7.92 7.92 7.90 7.65 7.65 7.63 7.63 7.61 7.61 7.57 7.56 7.55 7.55 7.53 7.53
3.66 3.64 3.63 3.61 3.33 3.32 3.30 2.67 2.65 2.64 1.93 1.91 1.90 1.88 1.32 1.30 1.29
O
N
O
71
1H NMR (400 MHz, CDCl3)
1.00 0.98 1.02 1.01 2.00 2.00 2.00 2.02 3.05
f1 (ppm)

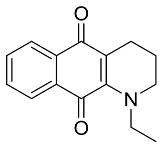

71

^{13}C NMR (100 MHz, $CDCl_3$)

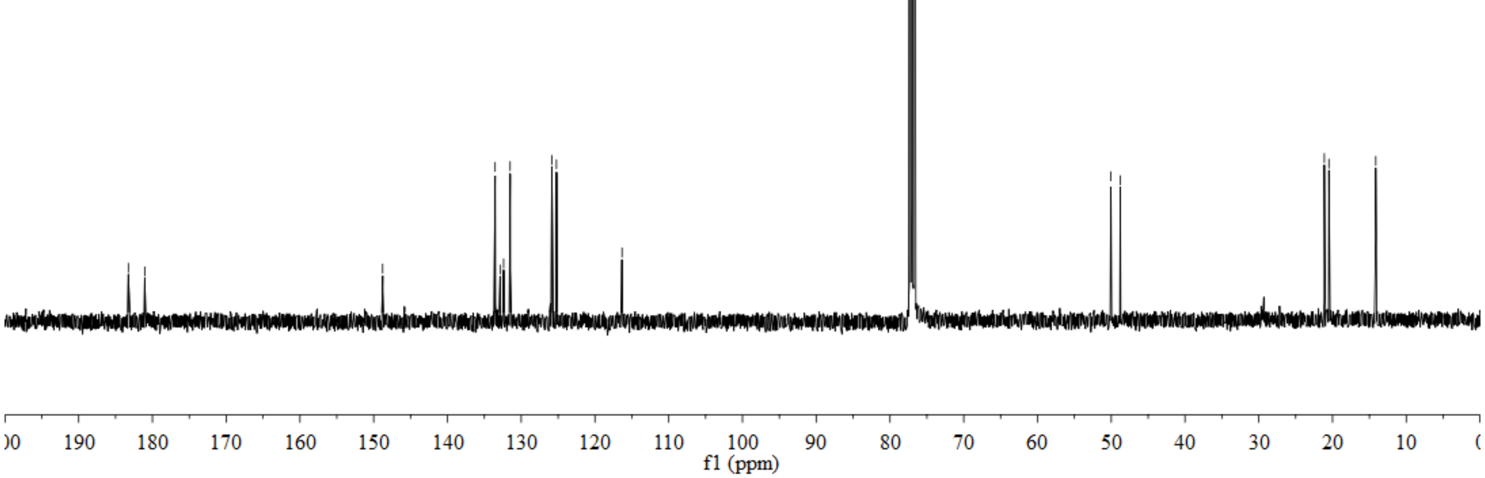

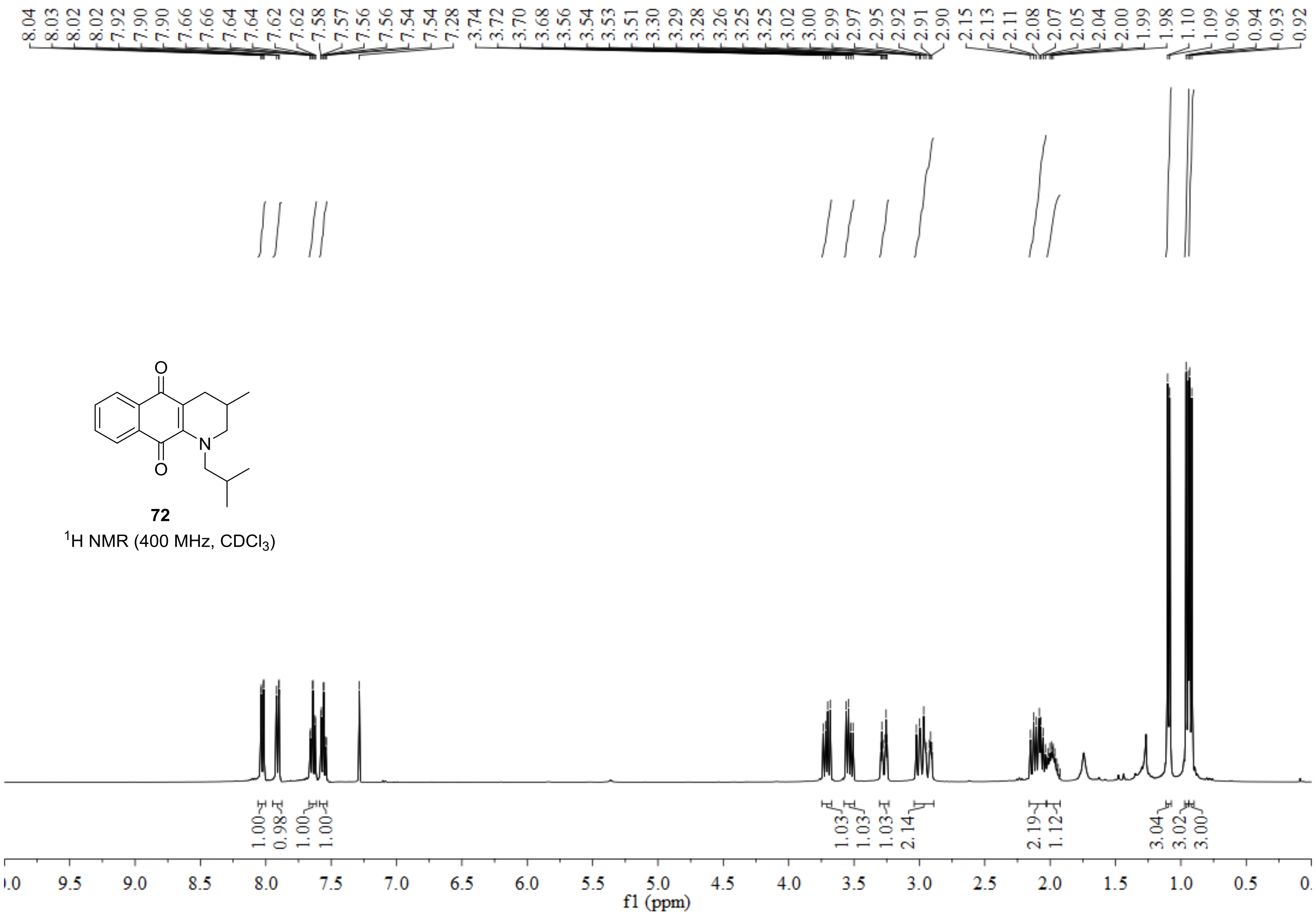

72

^{1}H NMR (400 MHz, $CDCl_3$)

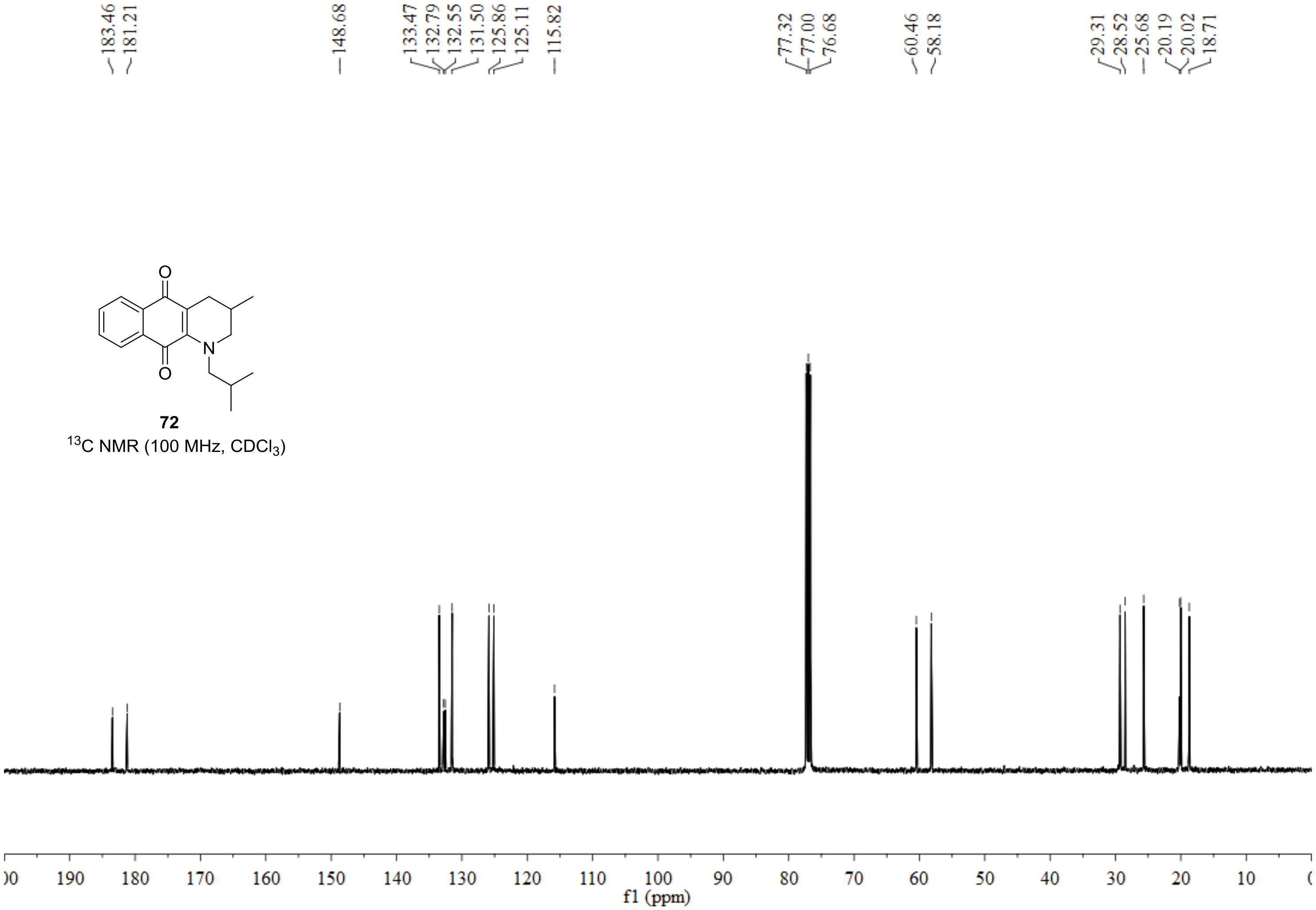

72

^{13}C NMR (100 MHz, $CDCl_3$)

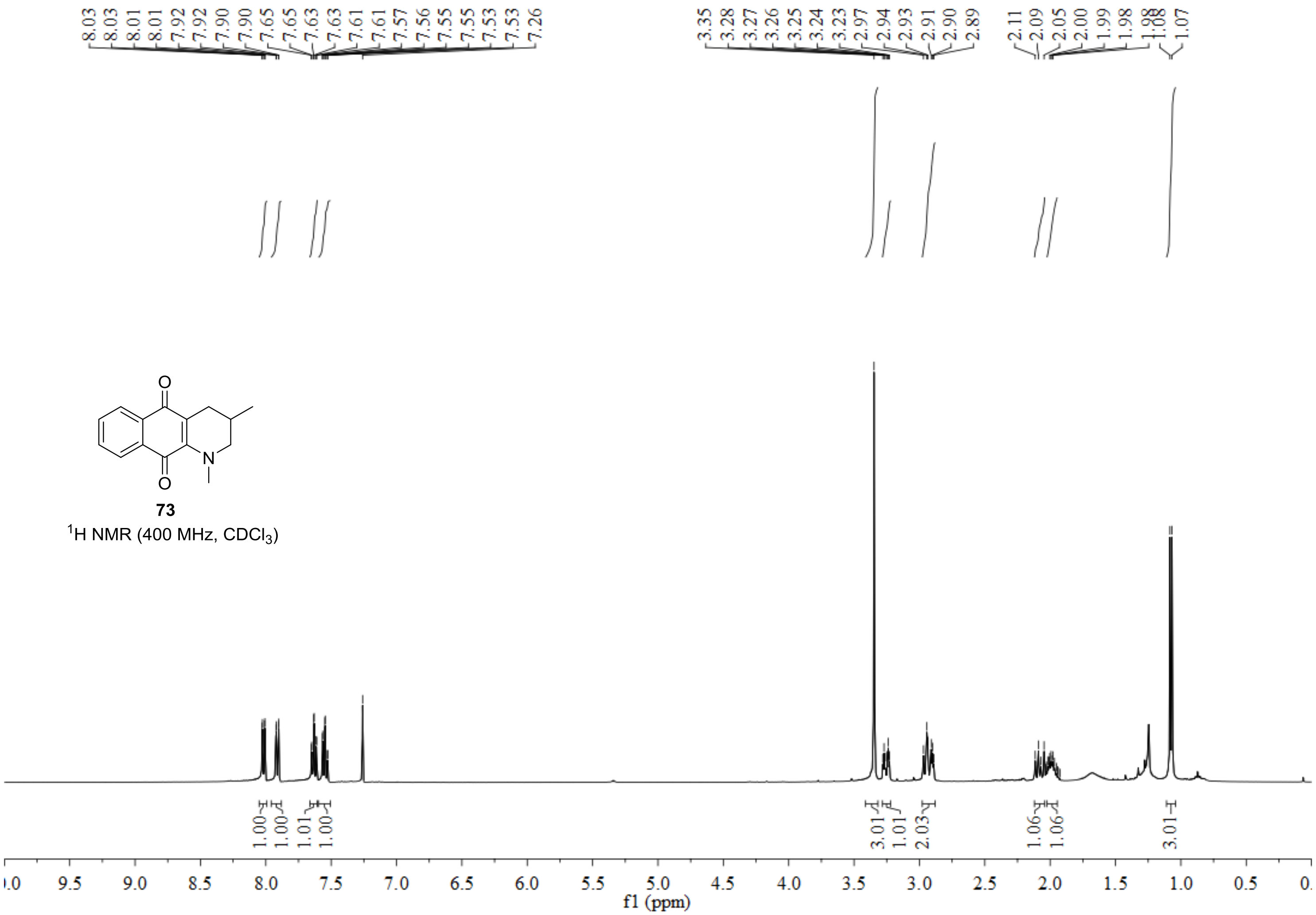

73
^{1}H NMR (400 MHz, CDCl$_3$)
f1 (ppm)

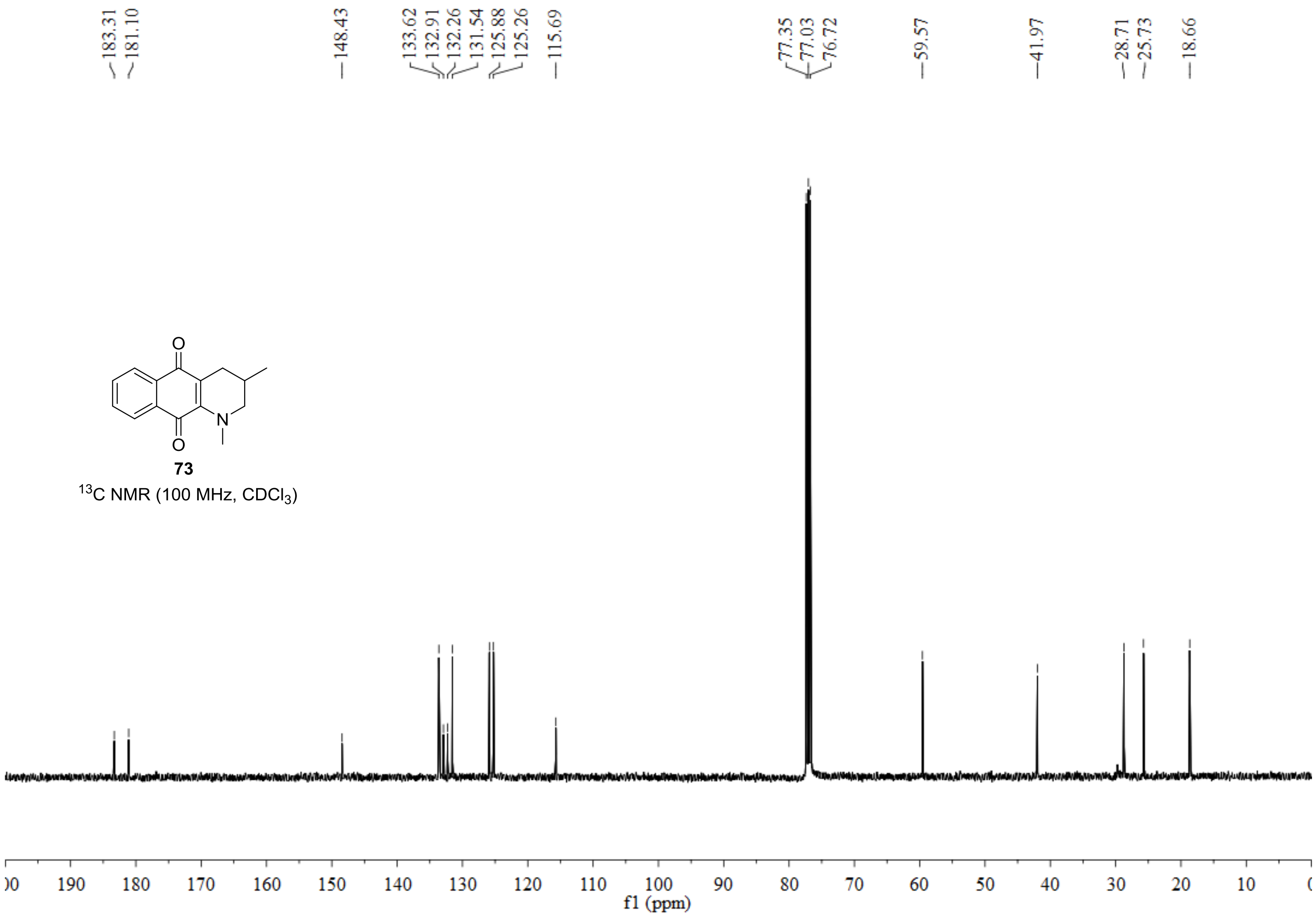

73

^{13}C NMR (100 MHz, $CDCl_3$)

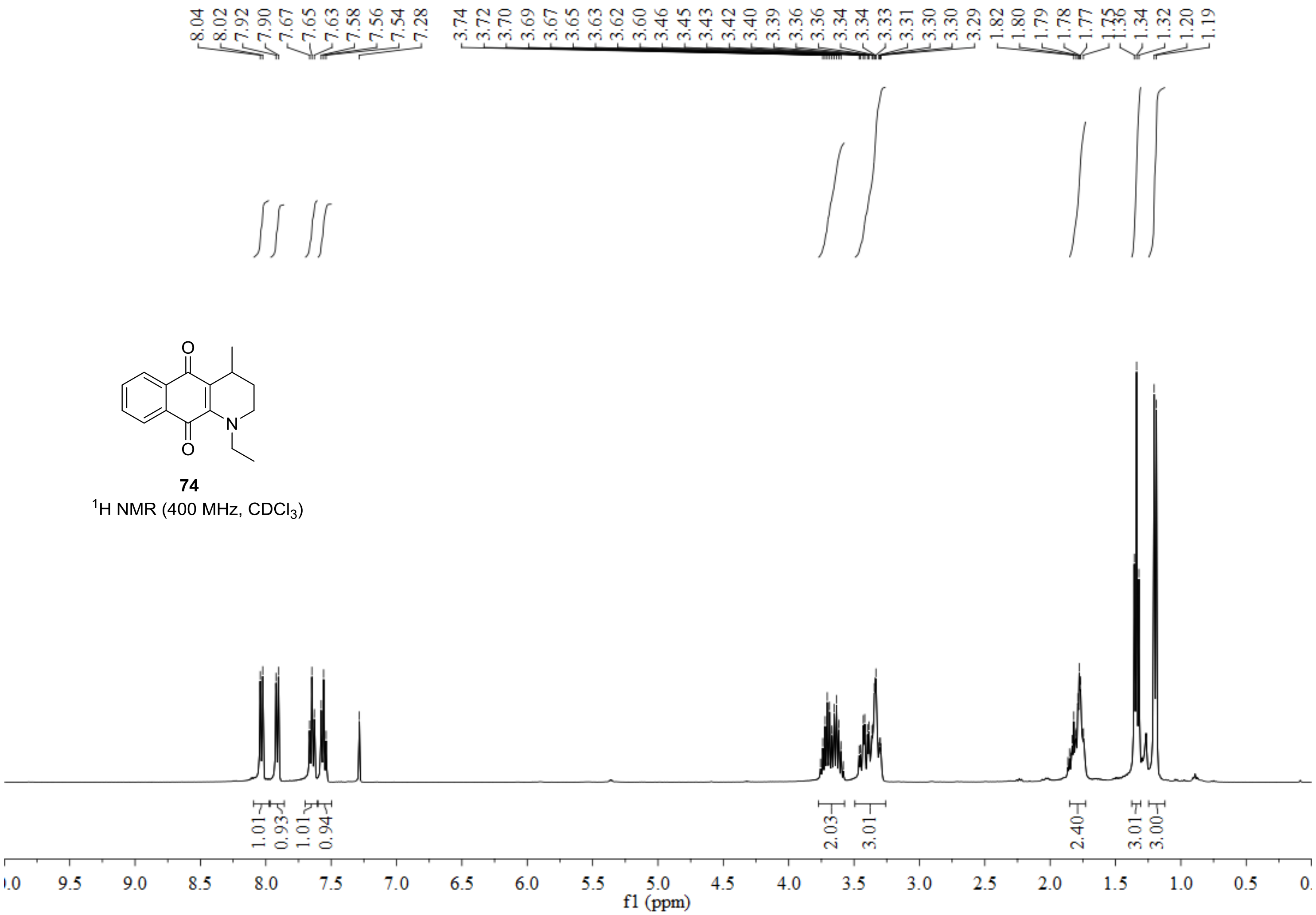
8.04 8.02 7.92 7.90 7.67 7.65 7.63 7.58 7.56 7.54 7.28
3.74 3.72 3.70 3.69 3.67 3.65 3.63 3.62 3.60 3.46 3.45 3.43 3.42 3.40 3.39 3.36 3.36 3.34 3.34 3.33 3.31 3.30 3.30 3.29
1.82 1.80 1.79 1.78 1.77 1.75 1.36 1.34 1.32 1.20 1.19
74
1H NMR (400 MHz, CDCl3)
1.01 0.93 1.01 0.94 2.03 3.01 2.40 3.01 3.00
f1 (ppm)

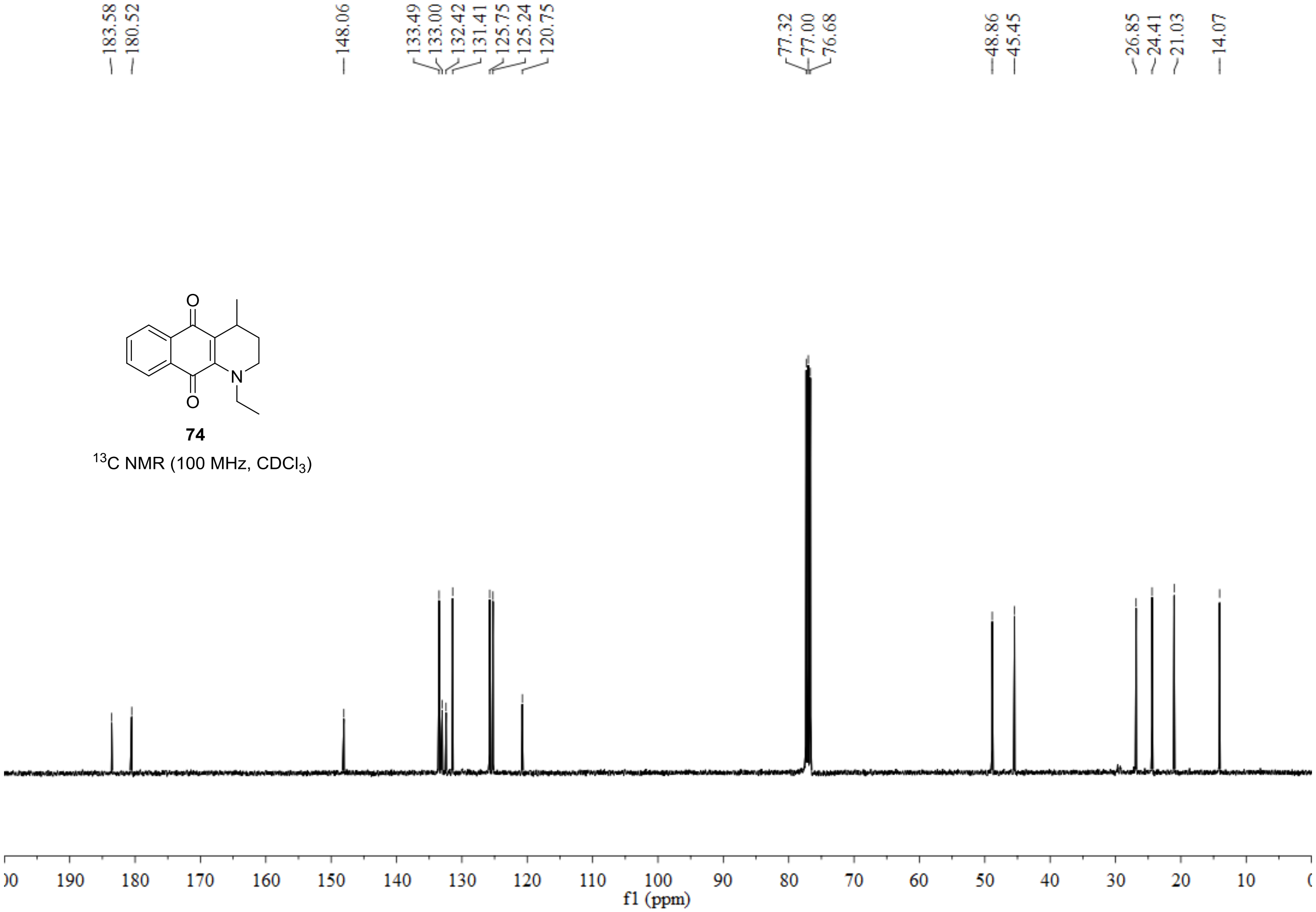
183.58
180.52
148.06
133.49
133.00
132.42
131.41
125.75
125.24
120.75
77.32
77.00
76.68
48.86
45.45
26.85
24.41
21.03
14.07
74
^{13}C NMR (100 MHz, $CDCl_3$)
190
180
170
160
150
140
130
120
110
100
90
80
70
60
50
40
30
20
10
f1 (ppm)

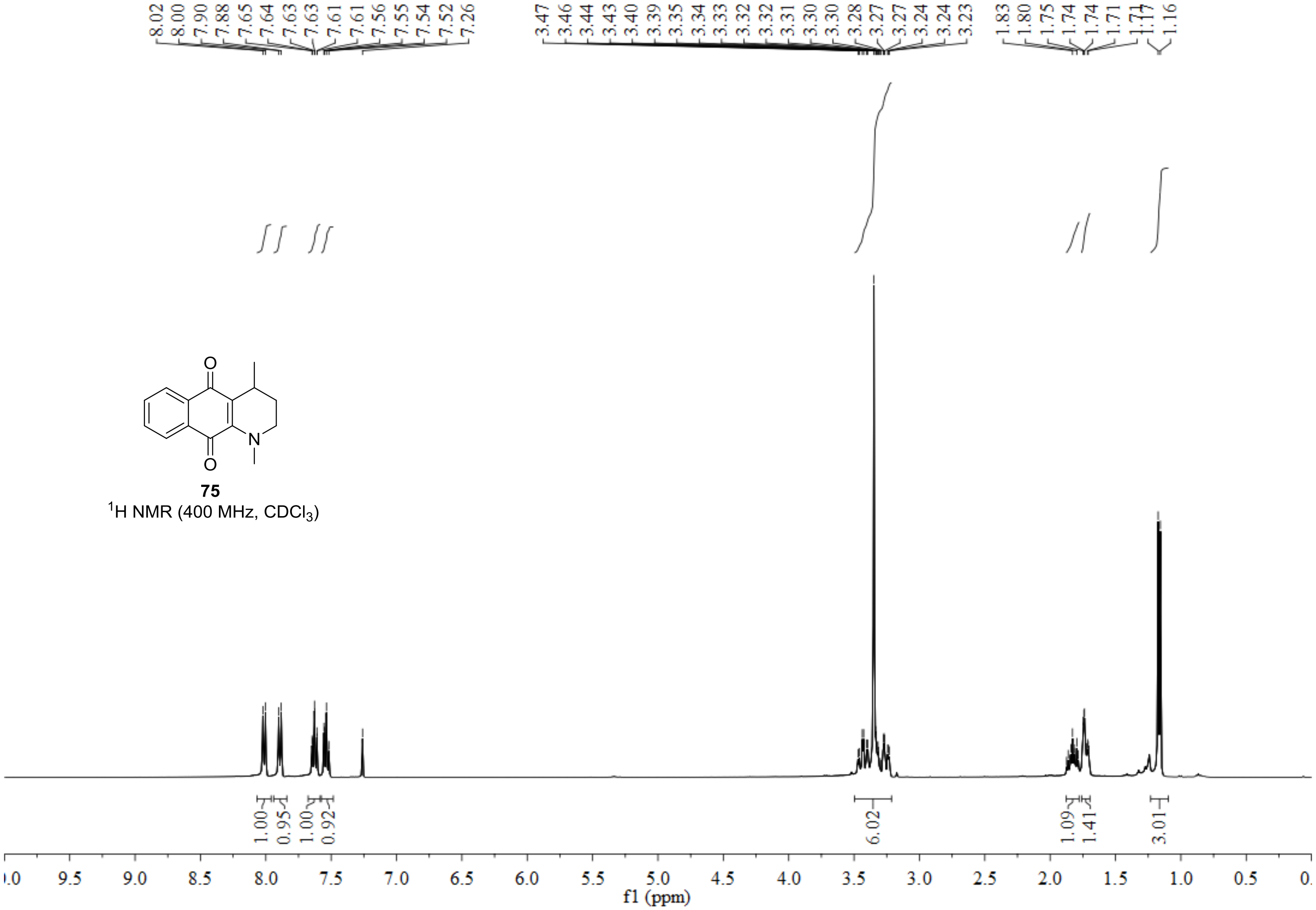
75
1H NMR (400 MHz, CDCl3)
f1 (ppm)

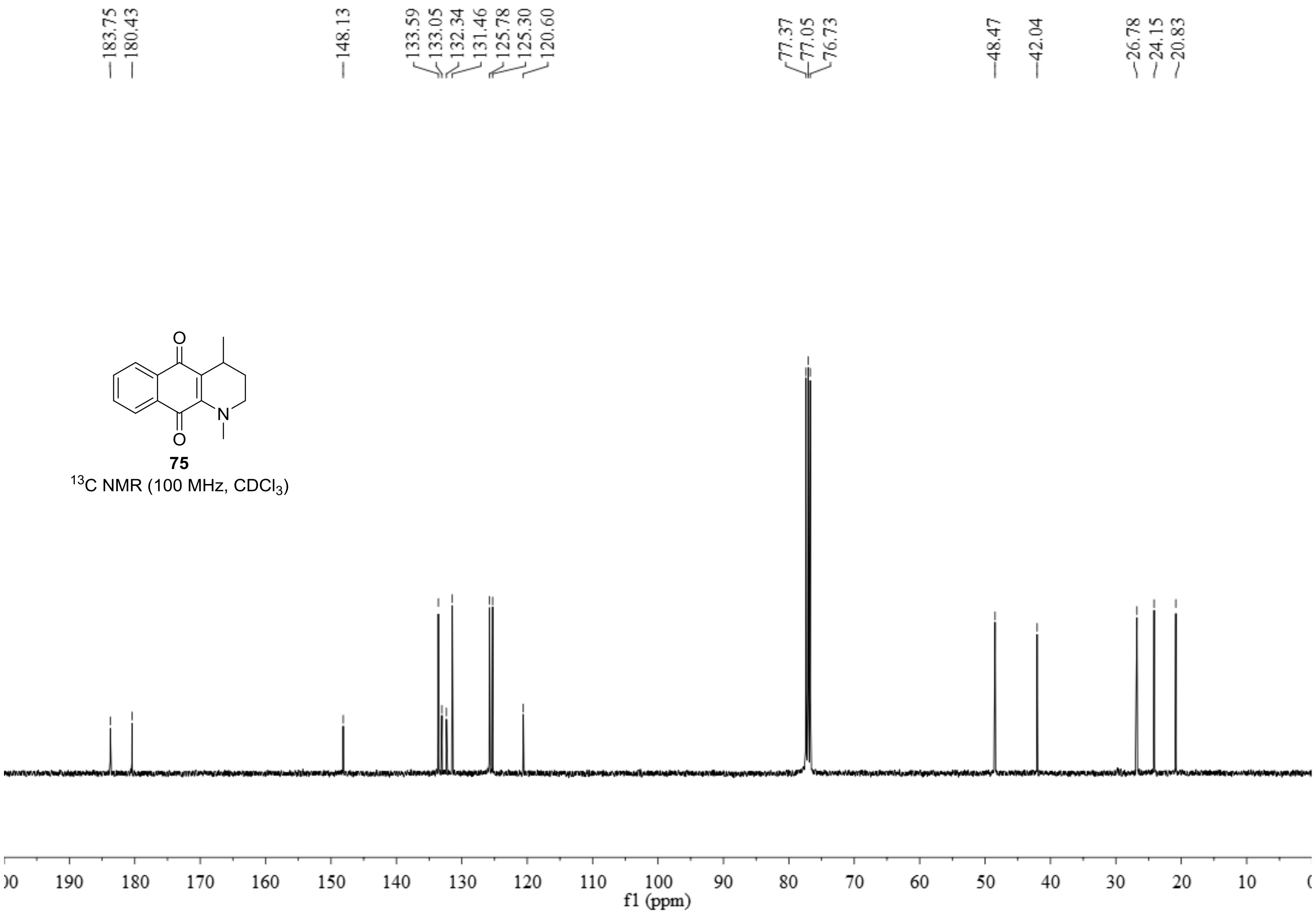

75

^{13}C NMR (100 MHz, $CDCl_3$)

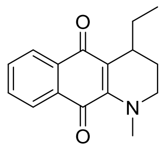

76

^1H NMR (400 MHz, $CDCl_3$)

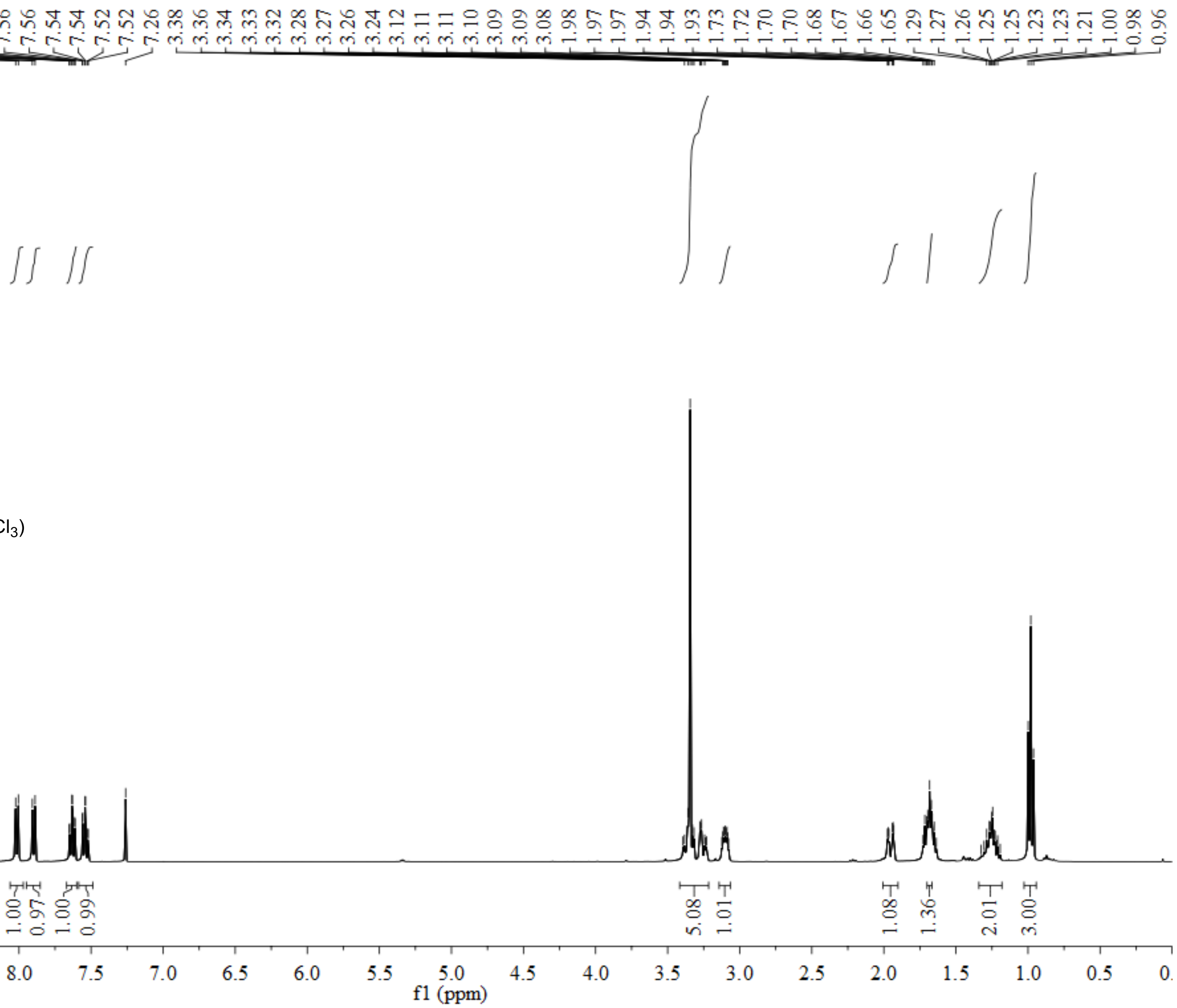

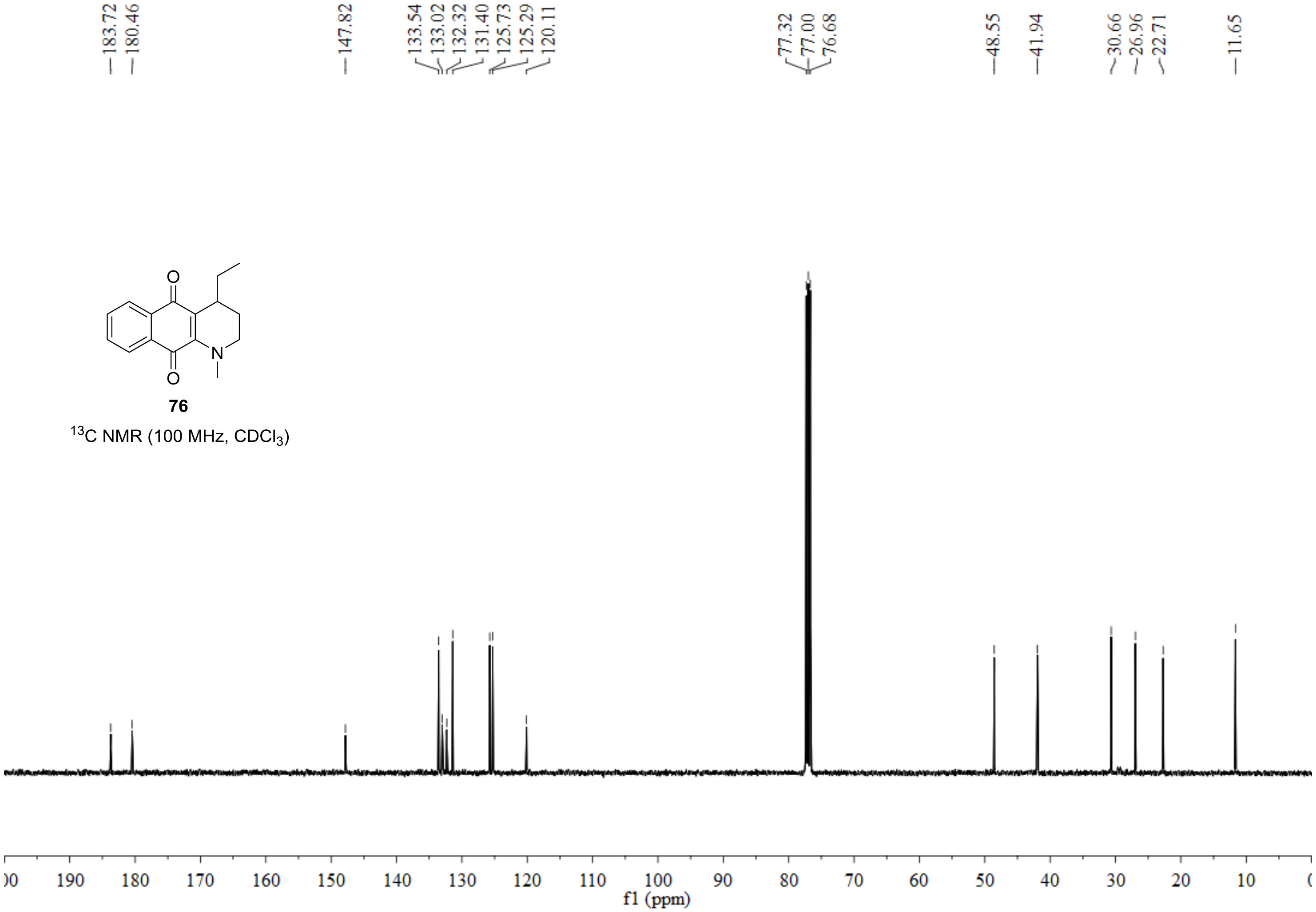
183.72
180.46
147.82
133.54
133.02
132.32
131.40
125.73
125.29
120.11
77.32
77.00
76.68
48.55
41.94
30.66
26.96
22.71
11.65
O
O
N
76
^{13}C NMR (100 MHz, $CDCl_3$)
f1 (ppm)

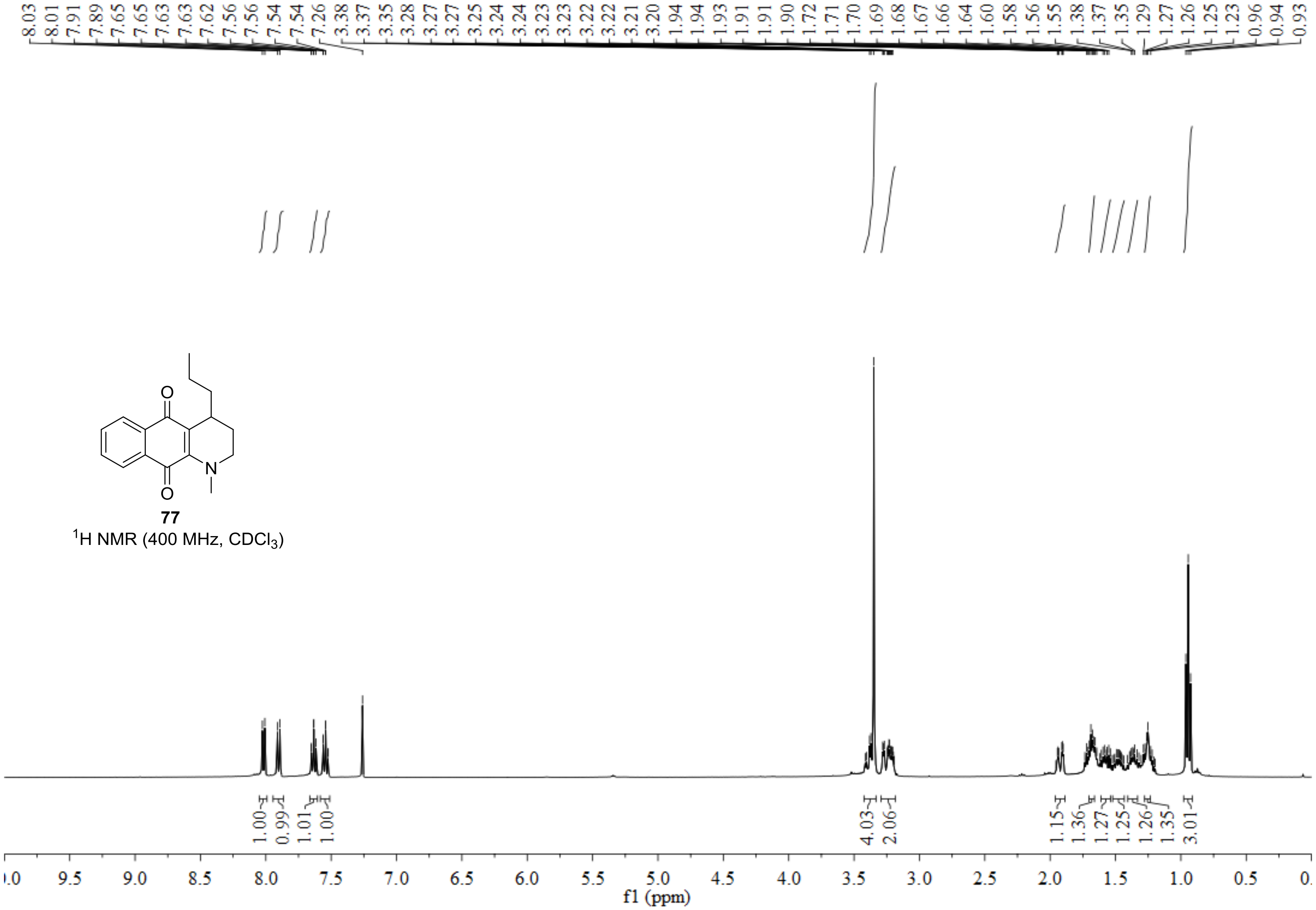
77
1H NMR (400 MHz, CDCl3)
f1 (ppm)

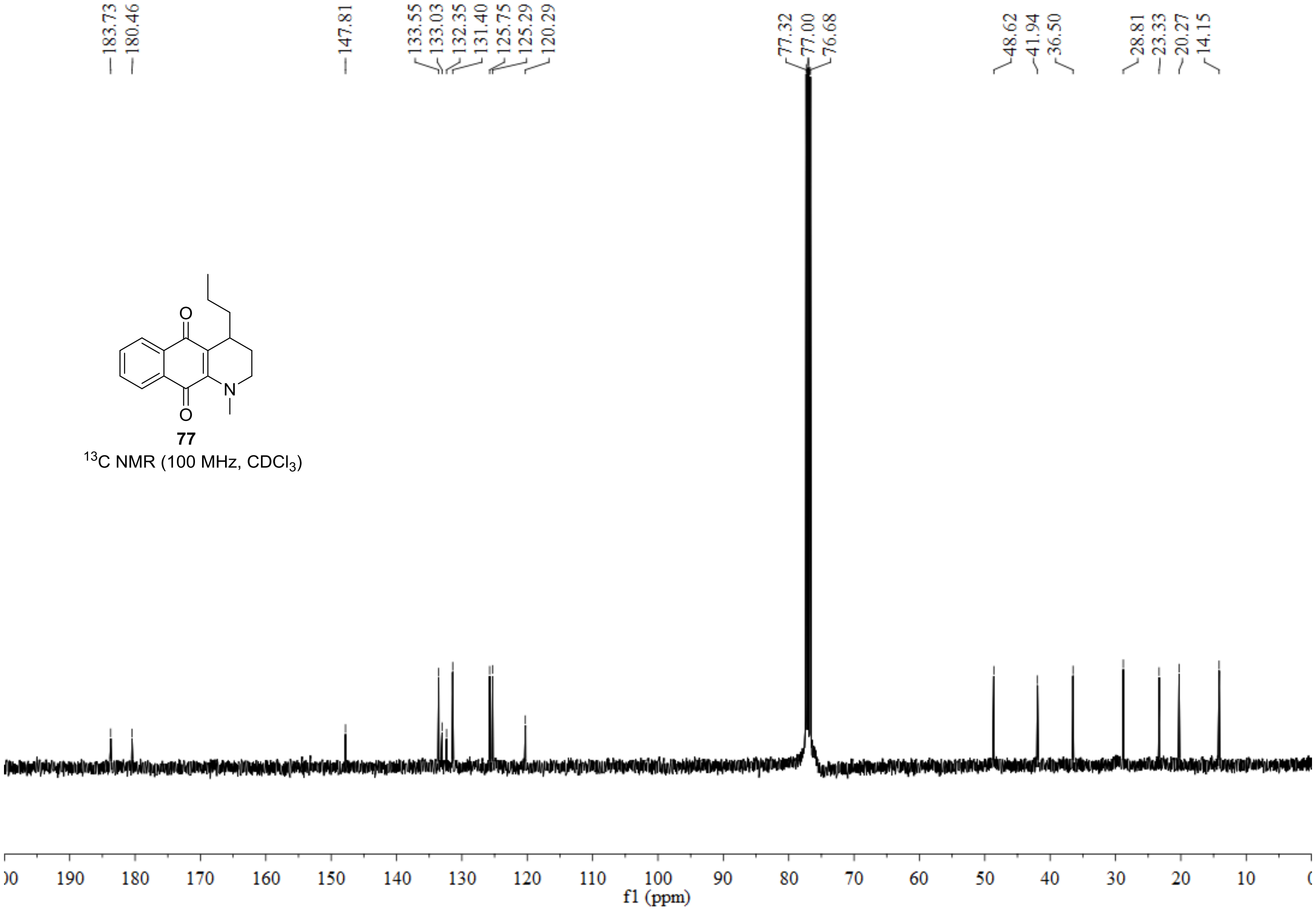

183.73
180.46
147.81
133.55
133.03
132.35
131.40
125.75
125.29
120.29
77.32
77.00
76.68
48.62
41.94
36.50
28.81
23.33
20.27
14.15
O
N
O
77
^{13}C NMR (100 MHz, CDCl$_3$)
190 180 170 160 150 140 130 120 110 100 90 80 70 60 50 40 30 20 10
f1 (ppm)

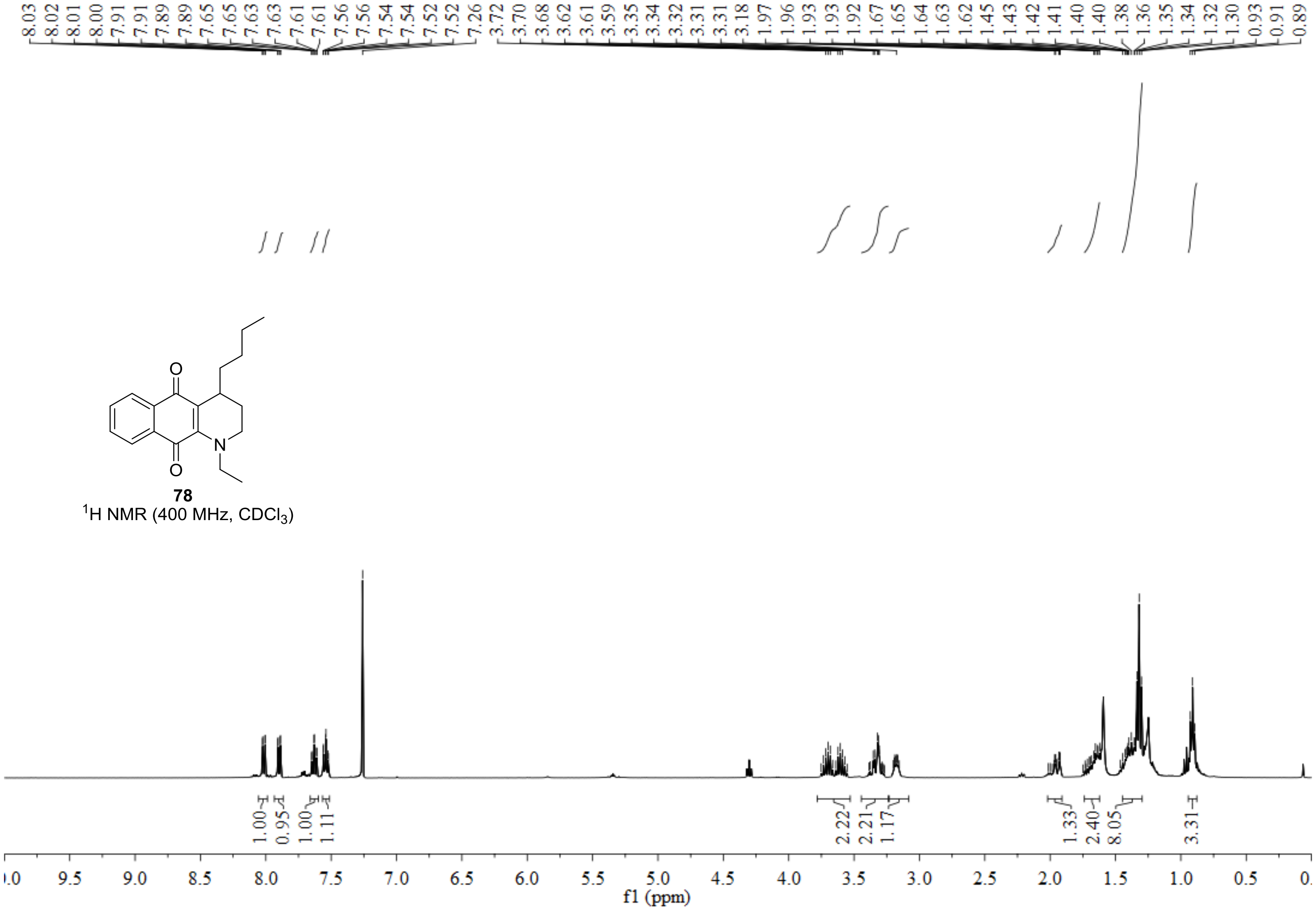

78
^{1}H NMR (400 MHz, CDCl$_3$)
f1 (ppm)

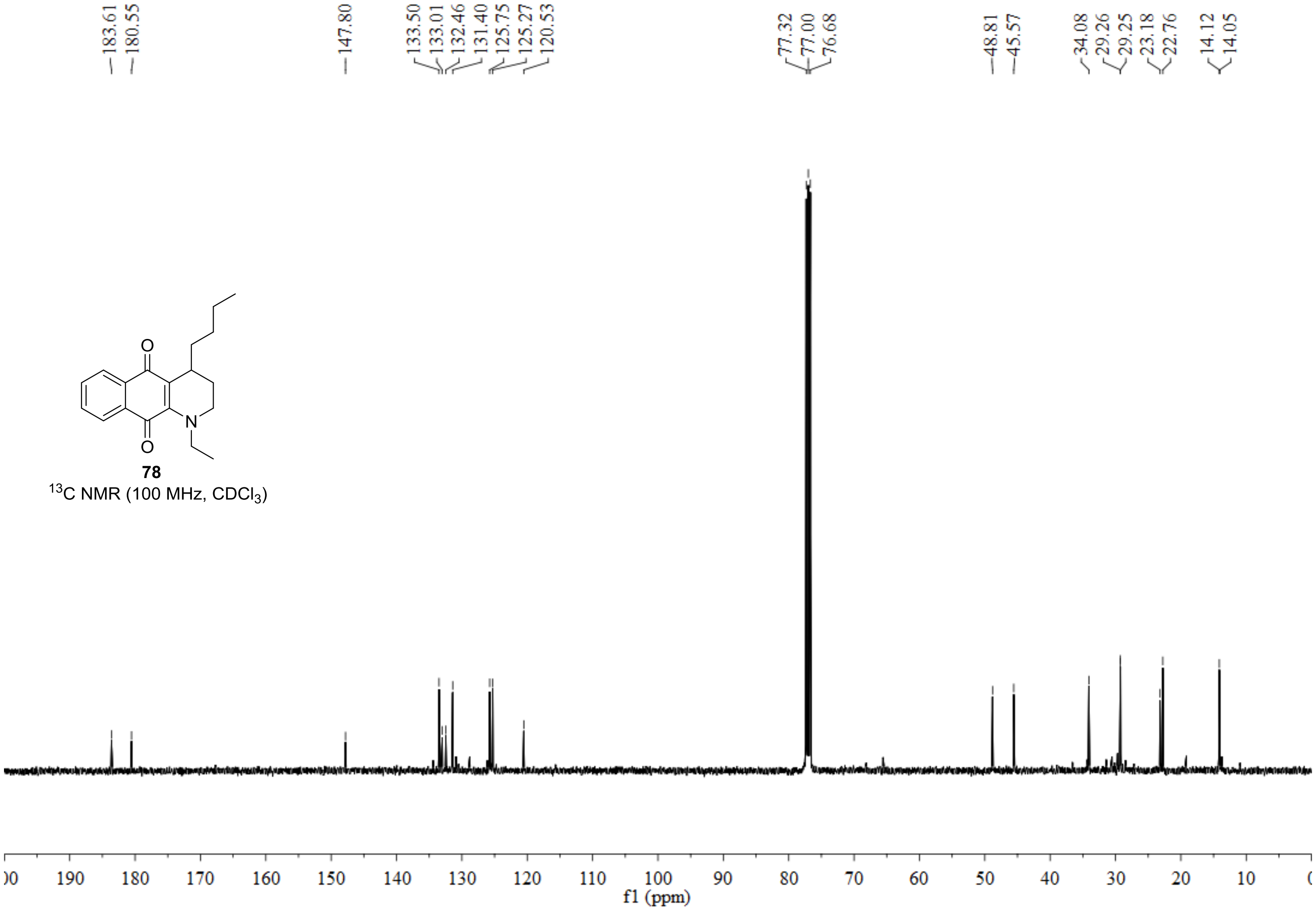

78

^{13}C NMR (100 MHz, $CDCl_3$)

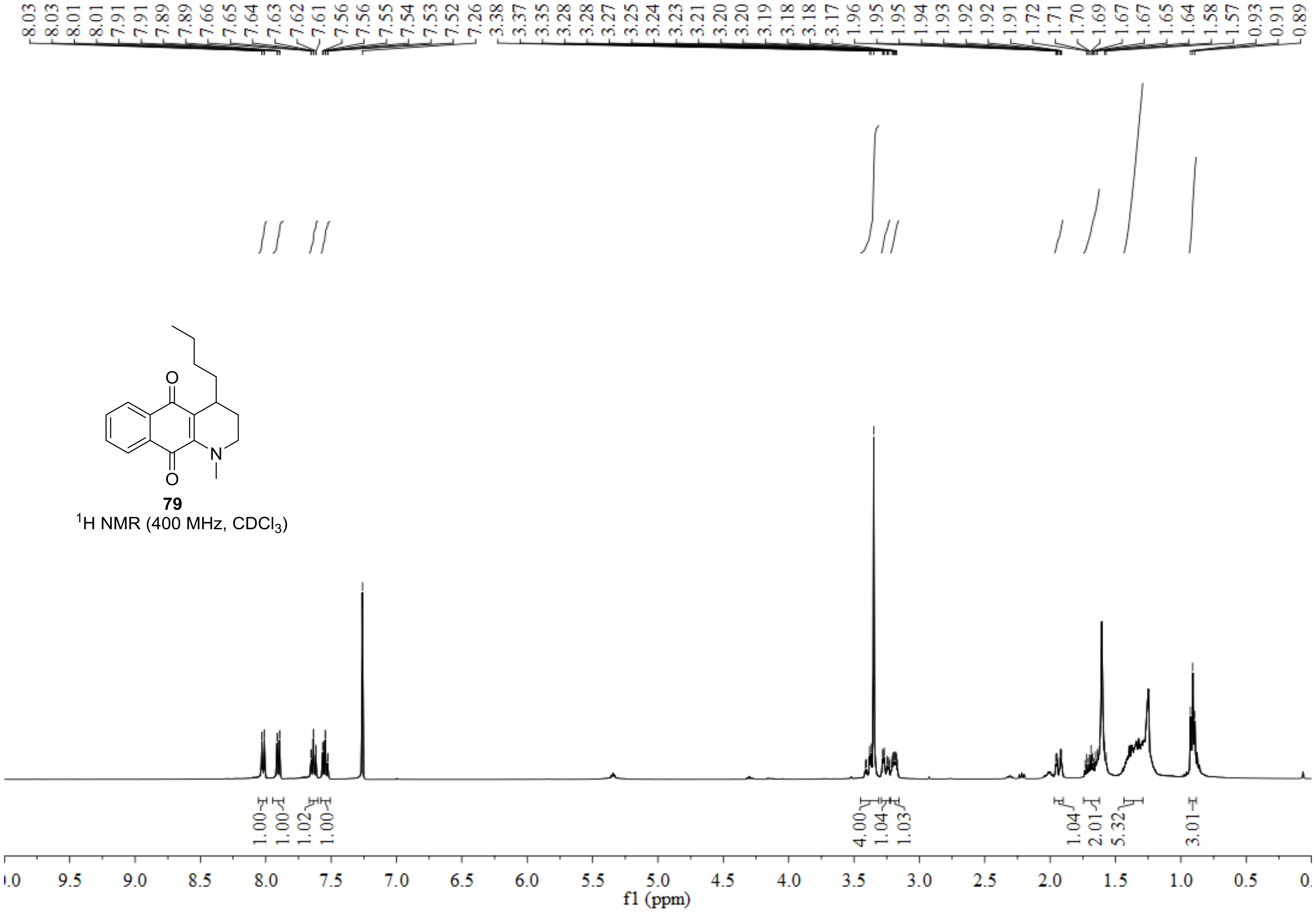

79

^{1}H NMR (400 MHz, $CDCl_3$)

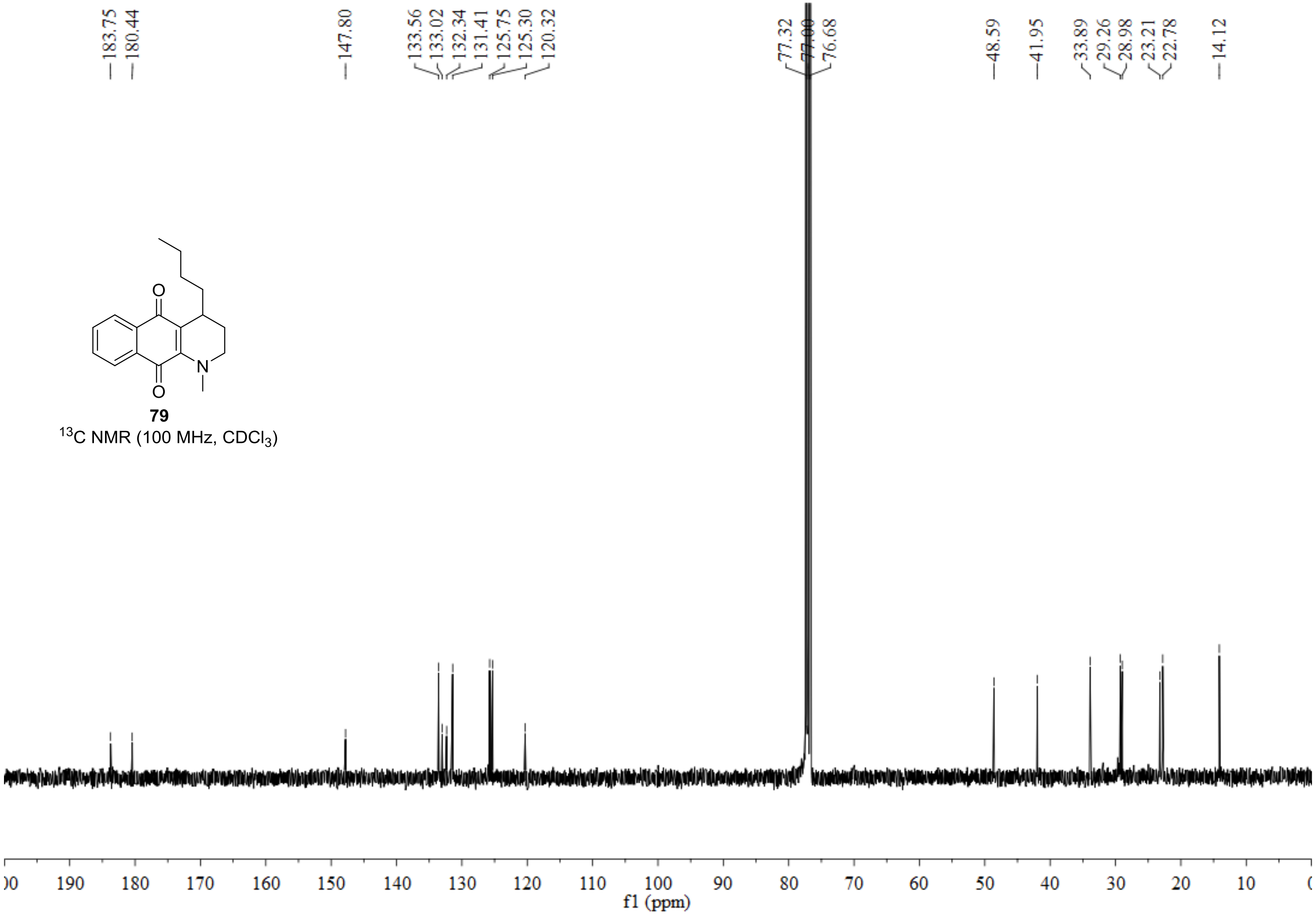
183.75
180.44
147.80
133.56
133.02
132.34
131.41
125.75
125.30
120.32
77.32
77.00
76.68
48.59
41.95
33.89
29.26
28.98
23.21
22.78
14.12
O
N
O
79
^{13}C NMR (100 MHz, $CDCl_3$)
190
180
170
160
150
140
130
120
110
100
90
80
70
60
50
40
30
20
10
f1 (ppm)

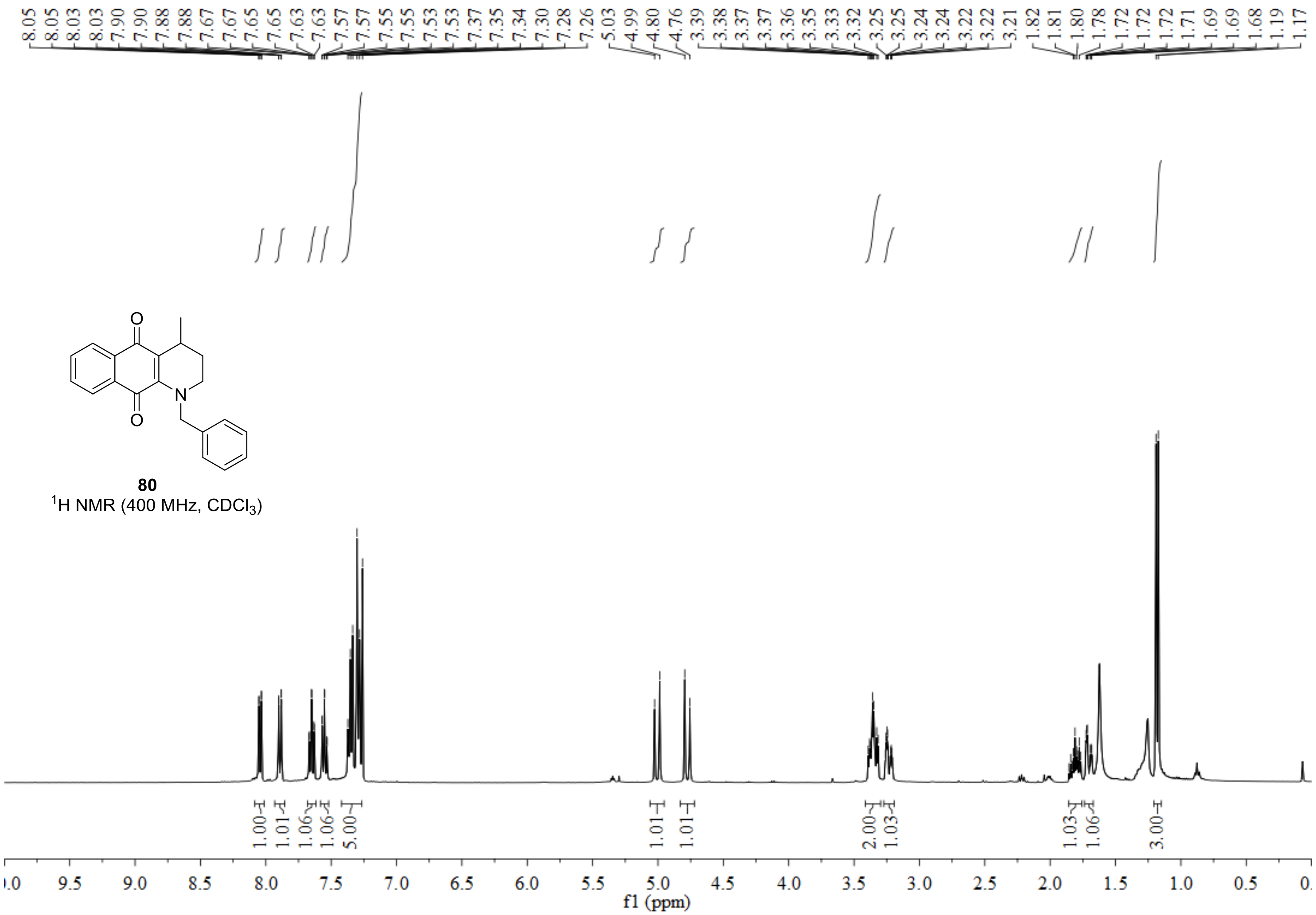

80
^1H NMR (400 MHz, $CDCl_3$)

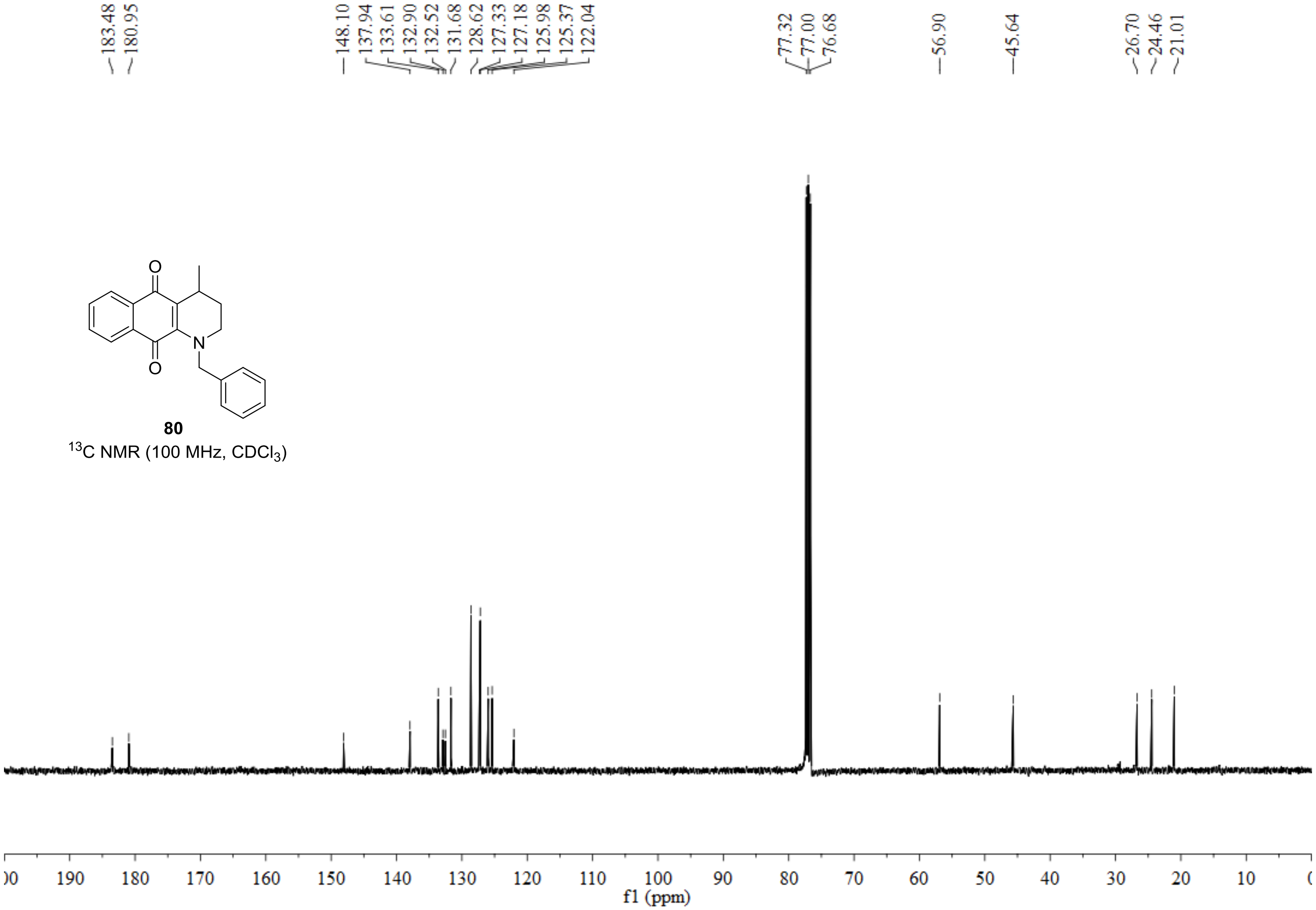

183.48
180.95
148.10
137.94
133.61
132.90
132.52
131.68
128.62
127.33
127.18
125.98
125.37
122.04
77.32
77.00
76.68
56.90
45.64
26.70
24.46
21.01
O
O
N
80
^{13}C NMR (100 MHz, $CDCl_3$)
190
180
170
160
150
140
130
120
110
100
90
80
70
60
50
40
30
20
10
f1 (ppm)

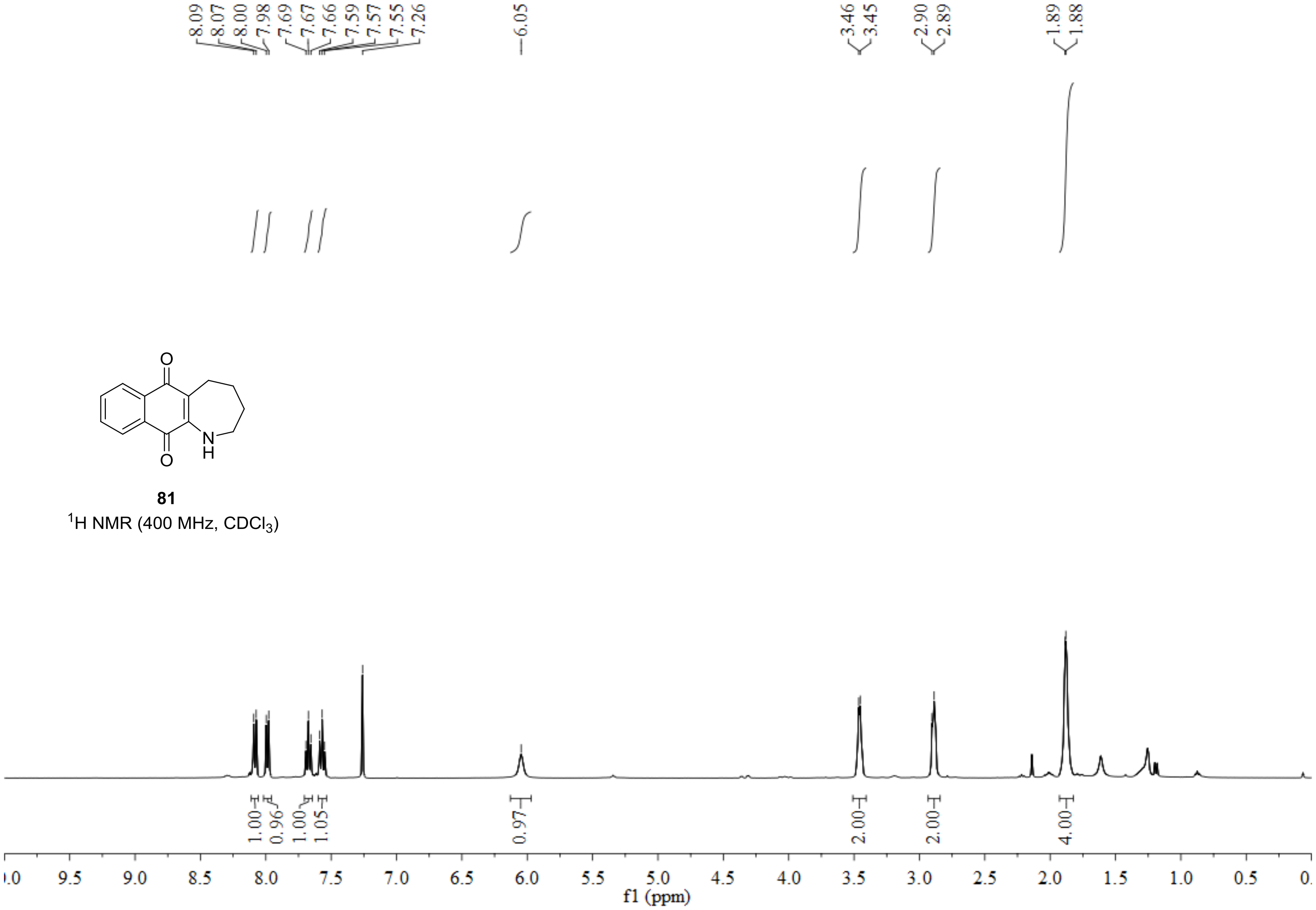

81

^1H NMR (400 MHz, $CDCl_3$)

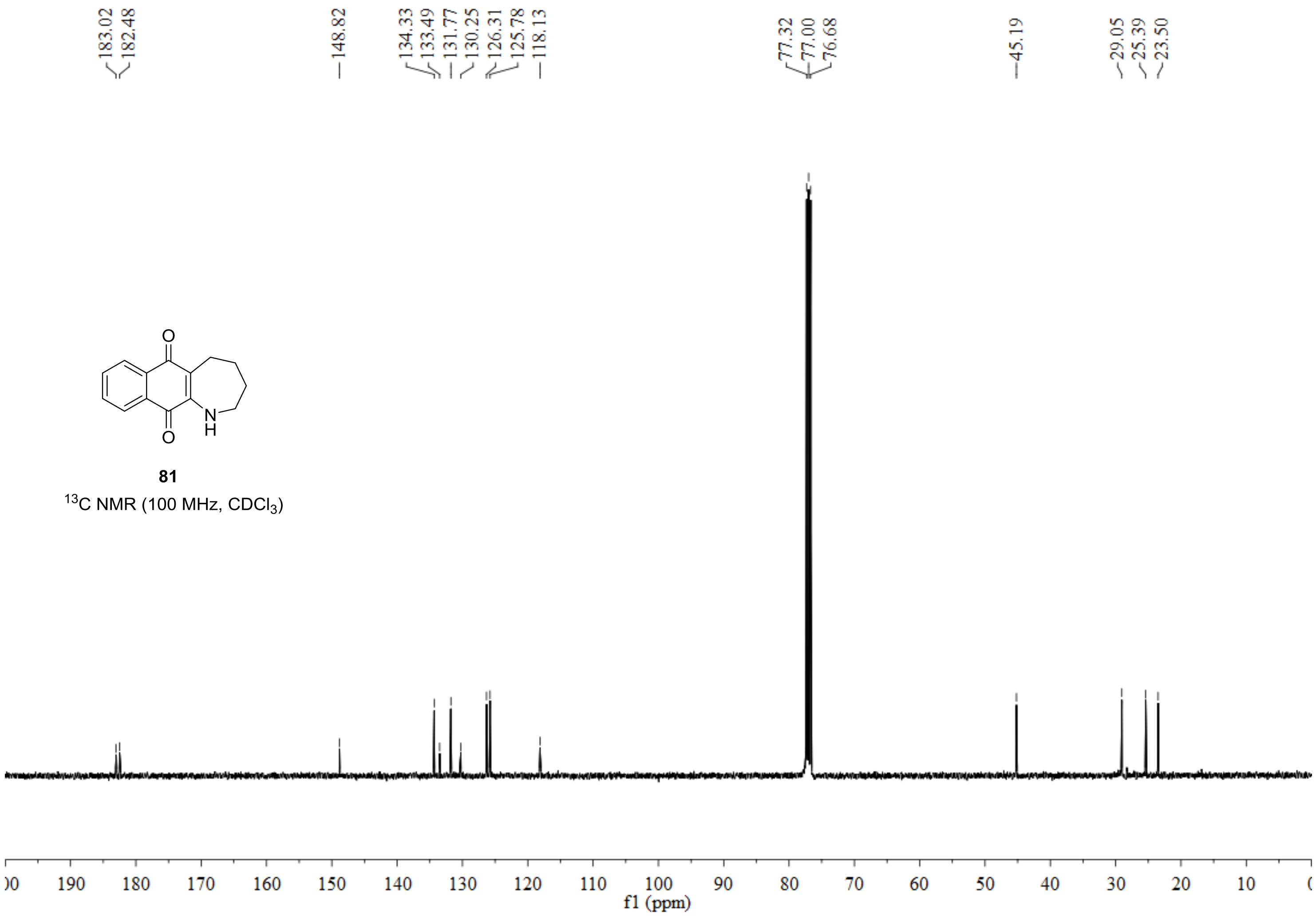

81

^{13}C NMR (100 MHz, $CDCl_3$)

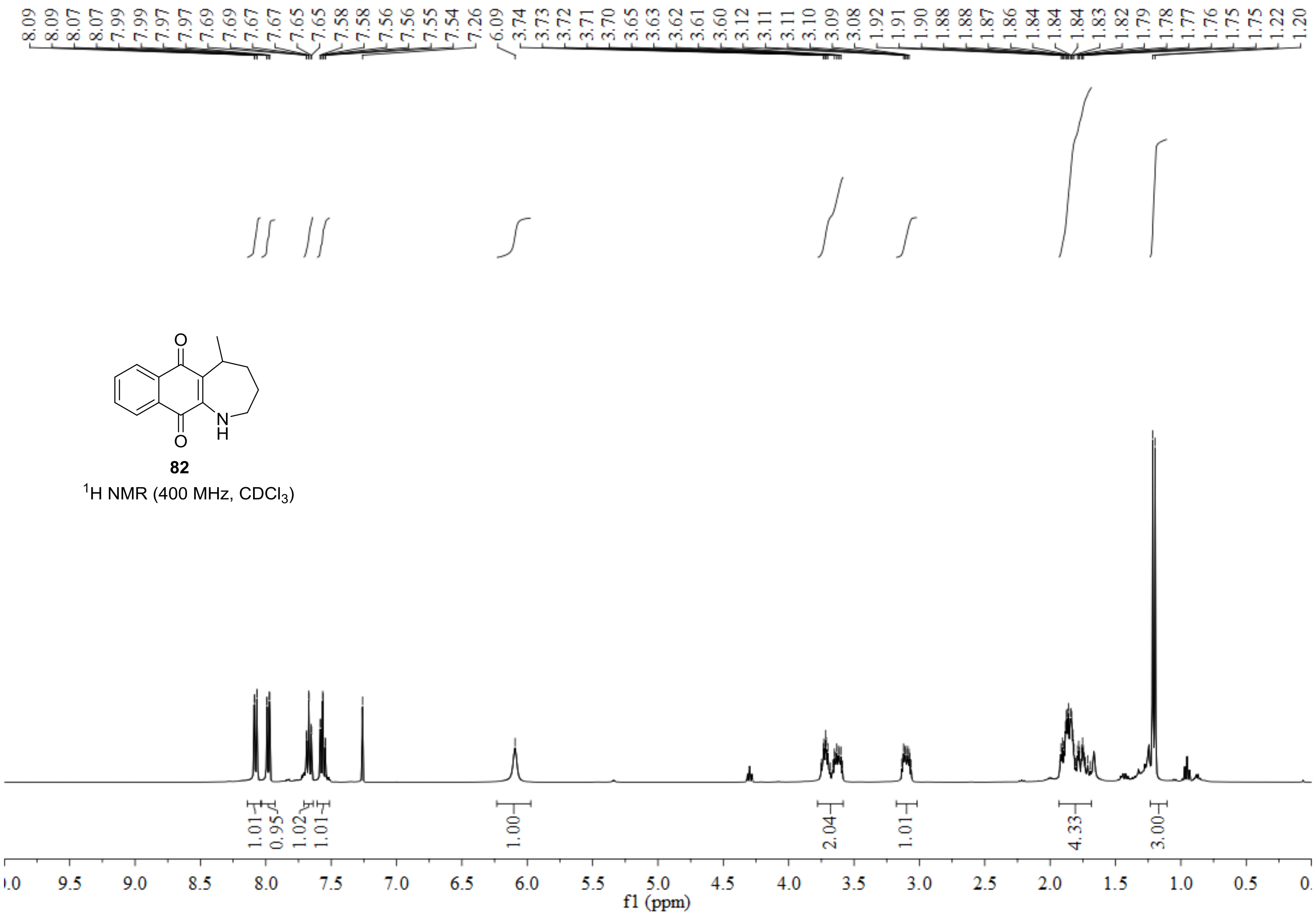

8.09 8.09 8.07 8.07 7.99 7.99 7.97 7.97 7.69 7.69 7.67 7.67 7.65 7.65 7.58 7.58 7.56 7.56 7.55 7.54 7.26 6.09 3.74 3.73 3.72 3.71 3.70 3.65 3.63 3.62 3.61 3.60 3.12 3.11 3.11 3.10 3.09 3.08 1.92 1.91 1.90 1.88 1.87 1.86 1.84 1.84 1.84 1.83 1.82 1.79 1.78 1.77 1.76 1.75 1.75 1.22 1.20
O
O
N
H
82
¹H NMR (400 MHz, CDCl₃)
1.01
0.95
1.02
1.01
1.00
2.04
1.01
4.33
3.00
9.5 9.0 8.5 8.0 7.5 7.0 6.5 6.0 5.5 5.0 4.5 4.0 3.5 3.0 2.5 2.0 1.5 1.0 0.5
f1 (ppm)

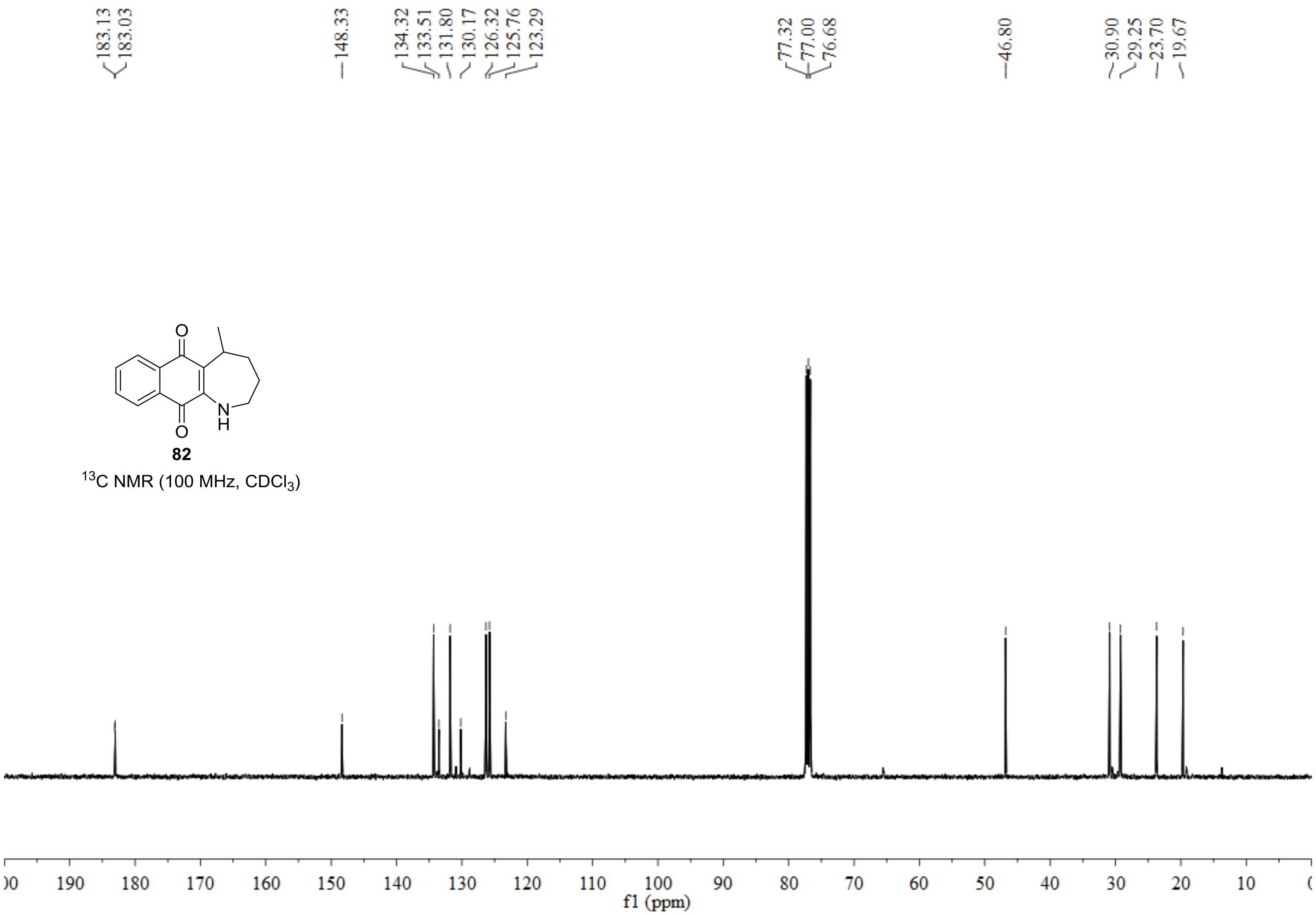
183.13
183.03
148.33
134.32
133.51
131.80
130.17
126.32
125.76
123.29
77.32
77.00
76.68
46.80
30.90
29.25
23.70
19.67
O
O
N
H
82
^{13}C NMR (100 MHz, $CDCl_3$)
190
180
170
160
150
140
130
120
110
100
90
80
70
60
50
40
30
20
10
f1 (ppm)

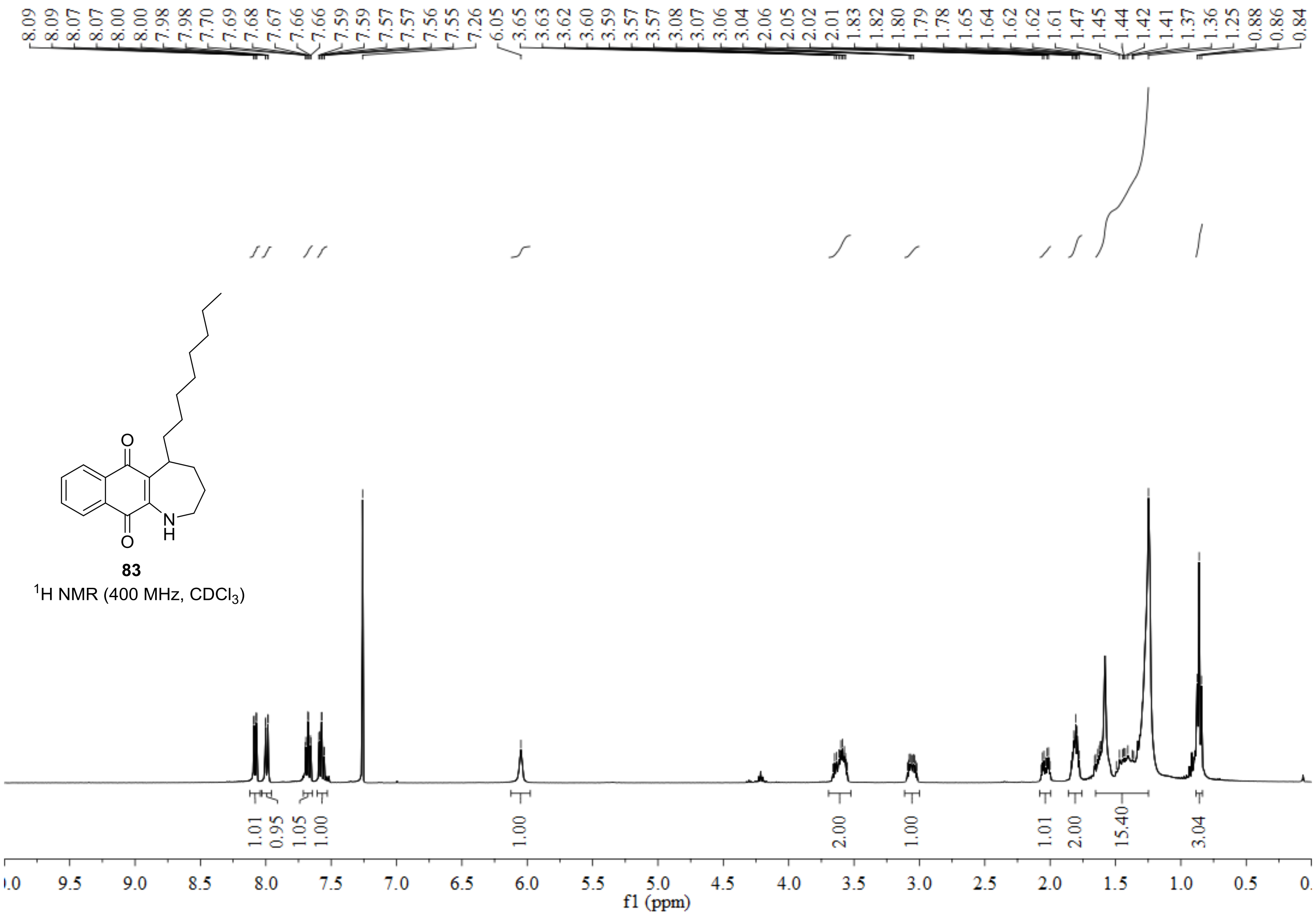

83

^1H NMR (400 MHz, $CDCl_3$)

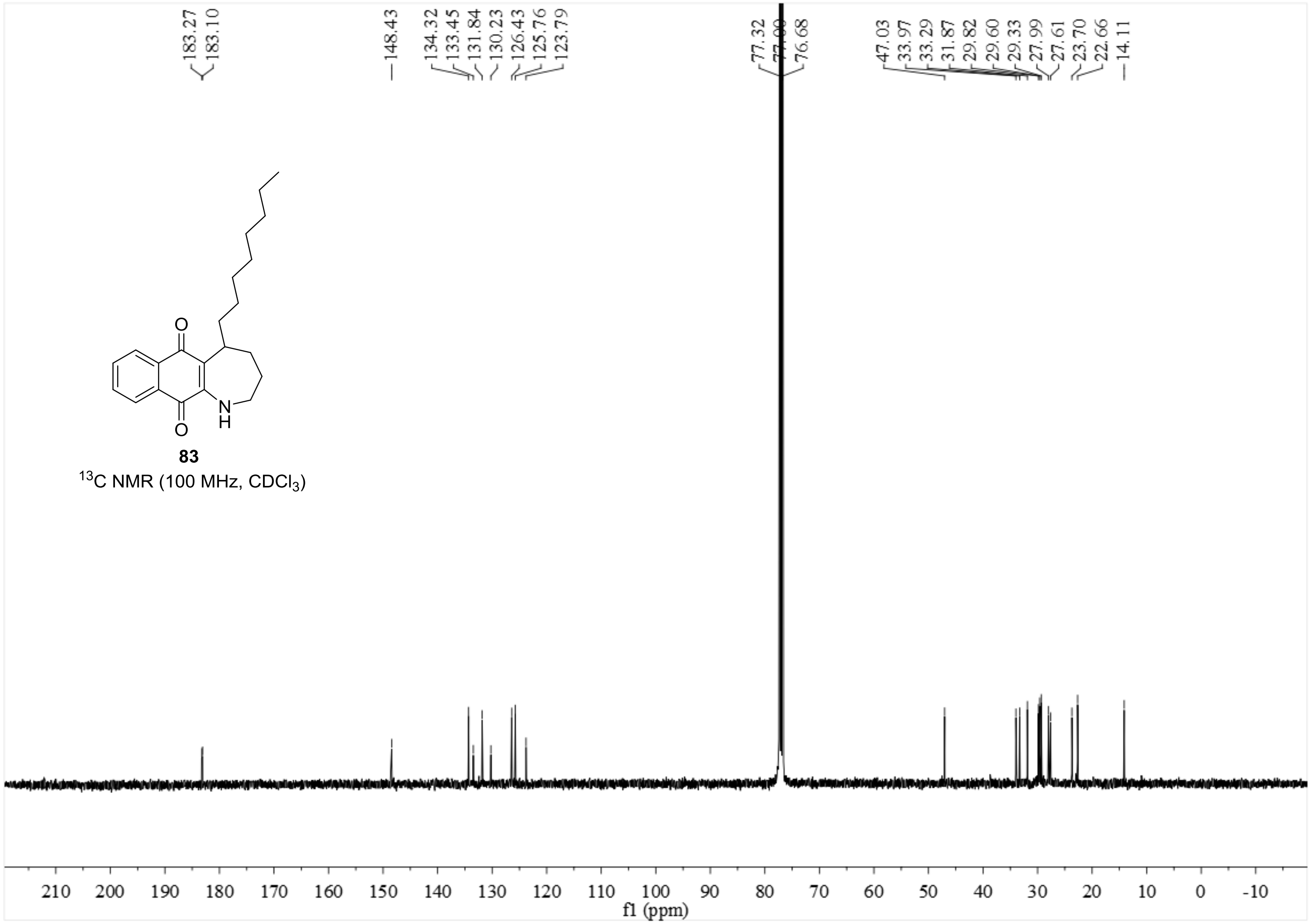
183.27
183.10
148.43
134.32
133.45
131.84
130.23
126.43
125.76
123.79
77.32
77.00
76.68
47.03
33.97
33.29
31.87
29.82
29.60
29.33
27.99
27.61
23.70
22.66
14.11
O
N
H
O
83
^{13}C NMR (100 MHz, $CDCl_3$)
210 200 190 180 170 160 150 140 130 120 110 100 90 80 70 60 50 40 30 20 10 0 -10
f1 (ppm)

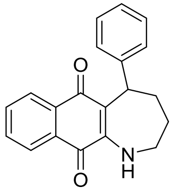

84

^{1}H NMR (400 MHz, $CDCl_3$)

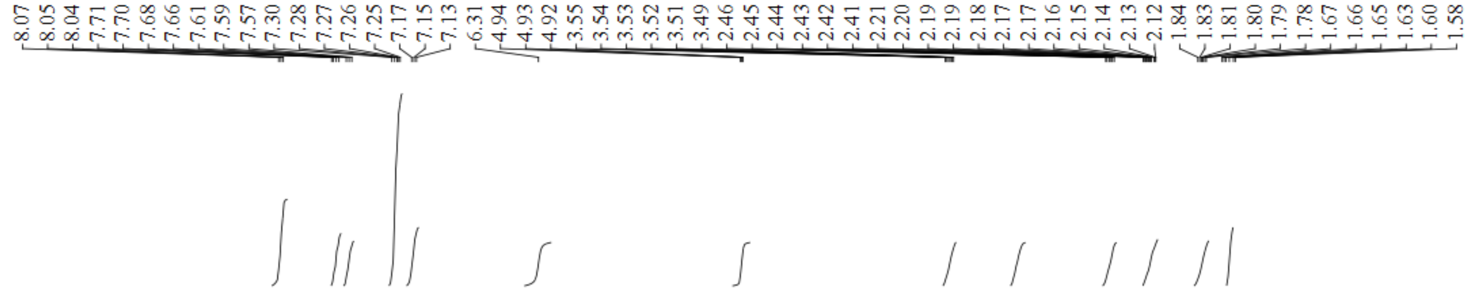
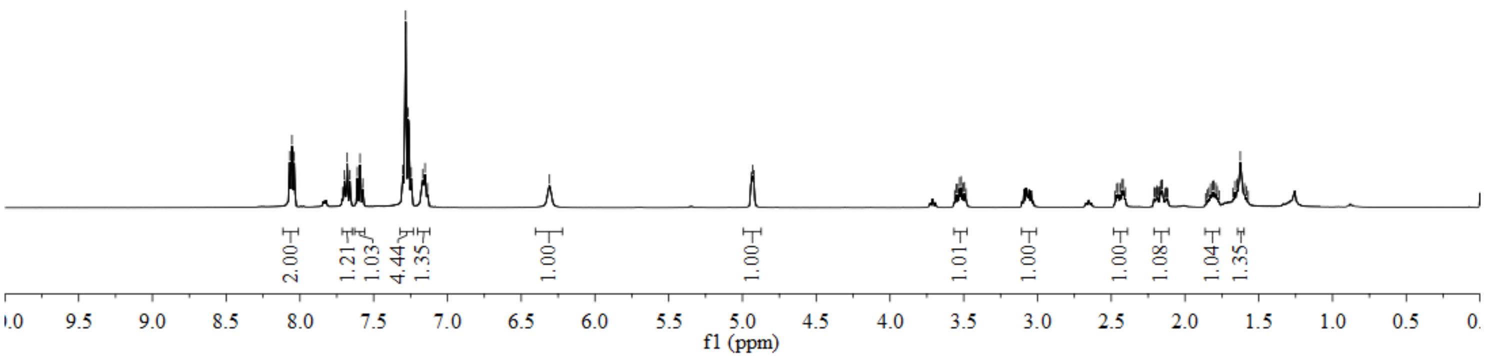

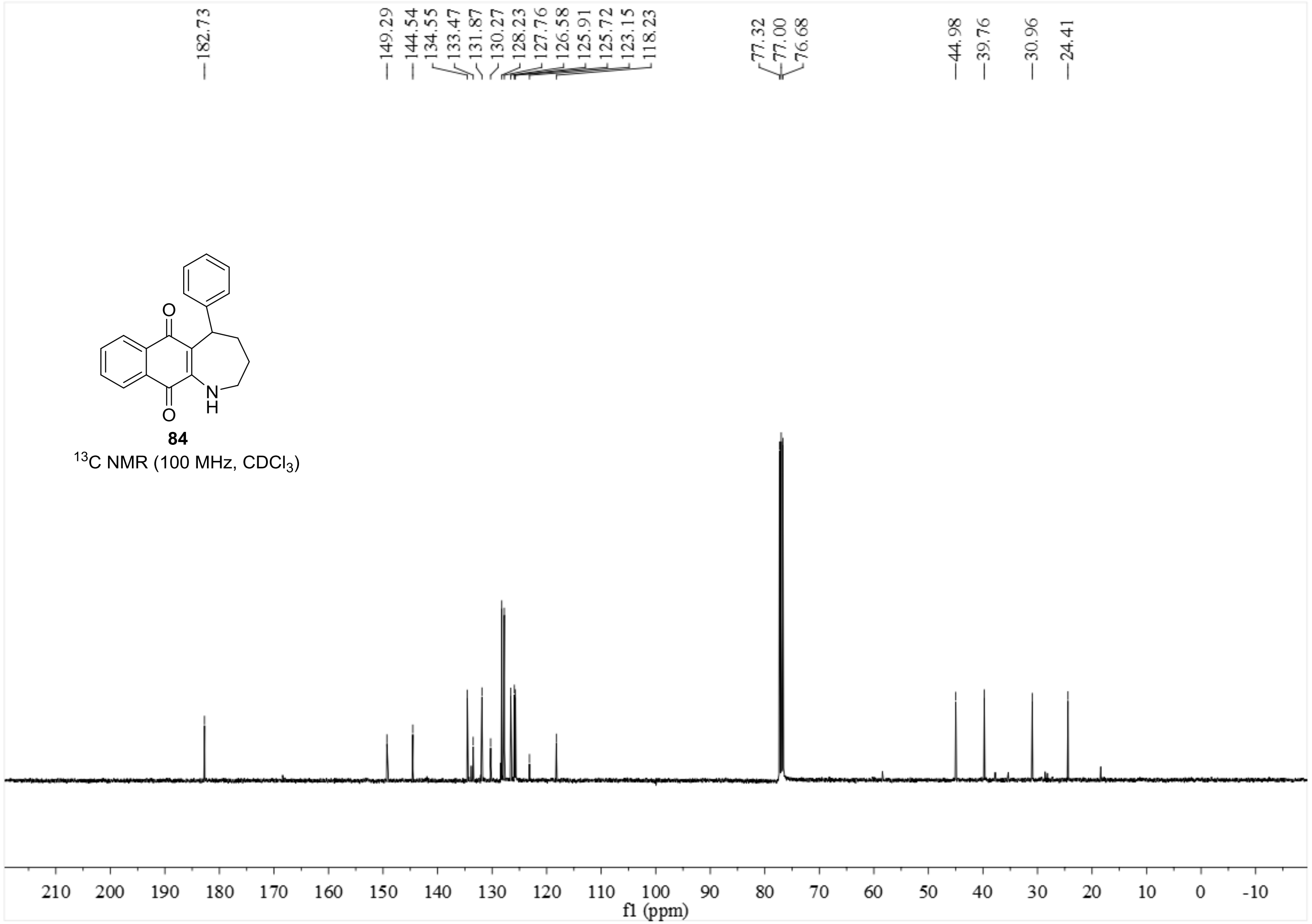

84

^{13}C NMR (100 MHz, $CDCl_3$)

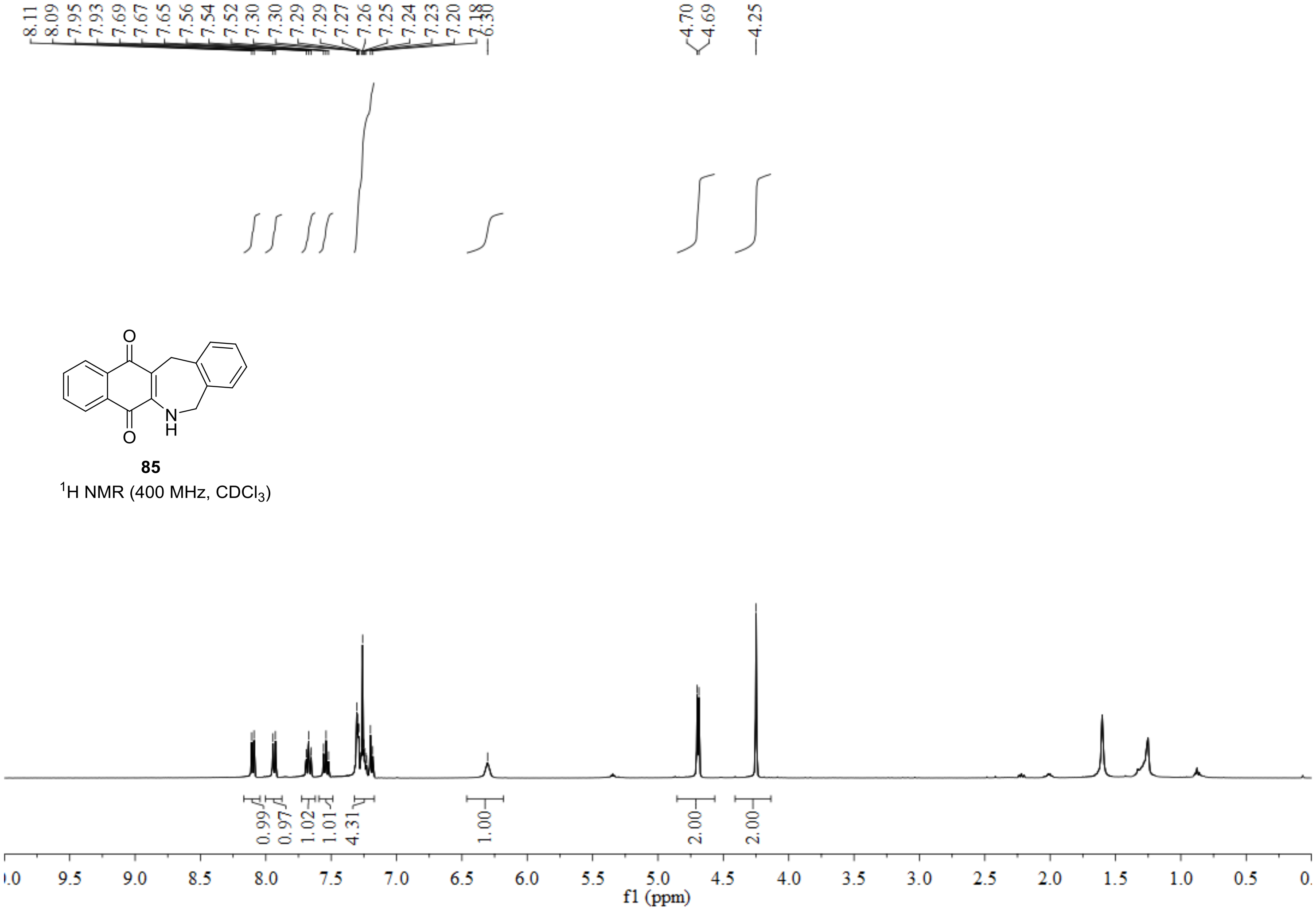
8.11
8.09
7.95
7.93
7.69
7.67
7.65
7.56
7.54
7.52
7.30
7.30
7.29
7.29
7.27
7.26
7.25
7.24
7.23
7.20
7.18
6.30
4.70
4.69
4.25
O
O
N
H
85
^{1}H NMR (400 MHz, $CDCl_3$)
0.99
0.97
1.02
1.01
4.31
1.00
2.00
2.00
9.5
9.0
8.5
8.0
7.5
7.0
6.5
6.0
5.5
5.0
4.5
4.0
3.5
3.0
2.5
2.0
1.5
1.0
0.5
f1 (ppm)

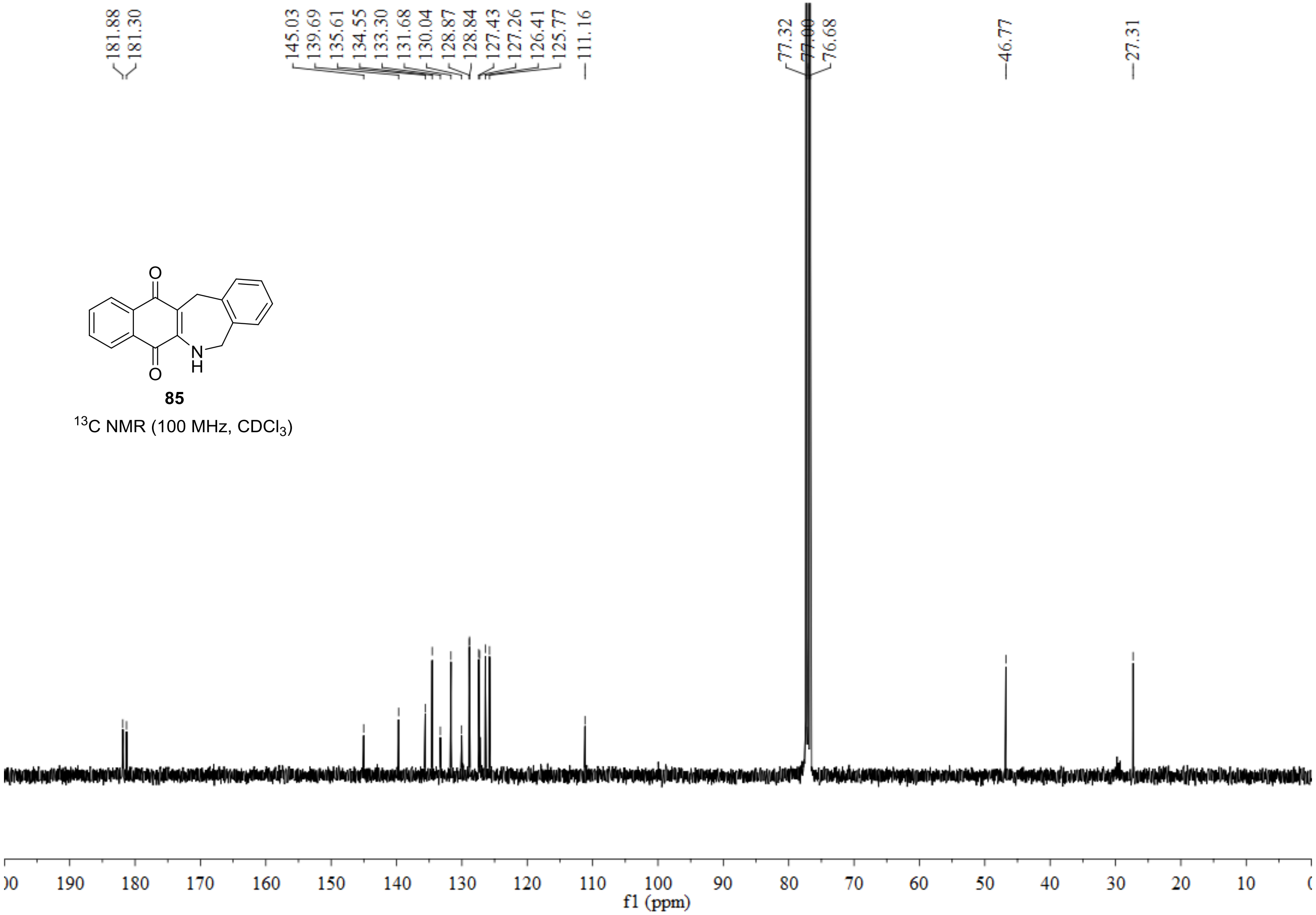

85

^{13}C NMR (100 MHz, $CDCl_3$)

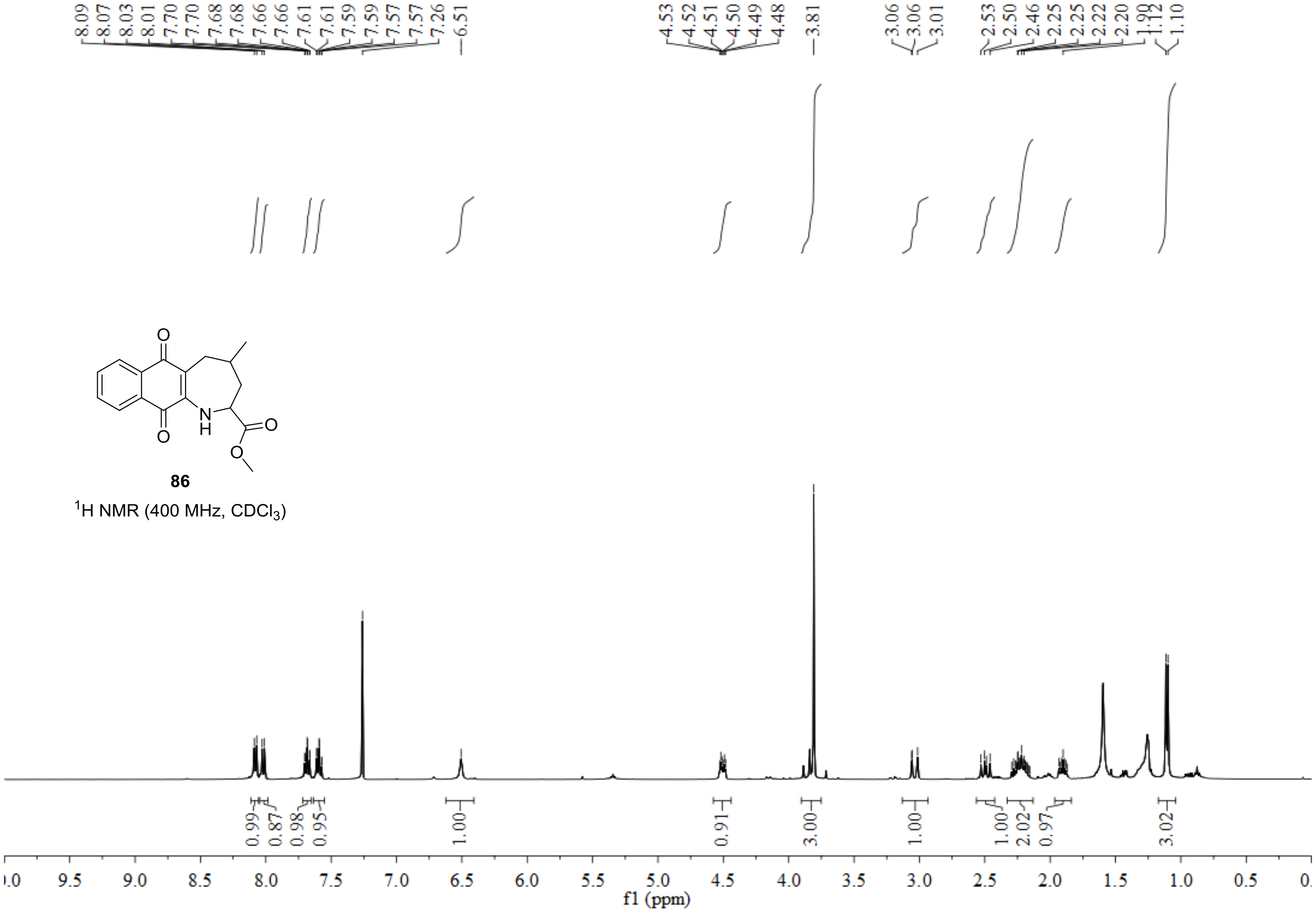

86

^1H NMR (400 MHz, $CDCl_3$)

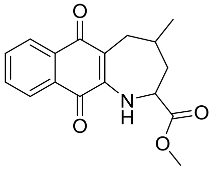

86

^{13}C NMR (100 MHz, $CDCl_3$)

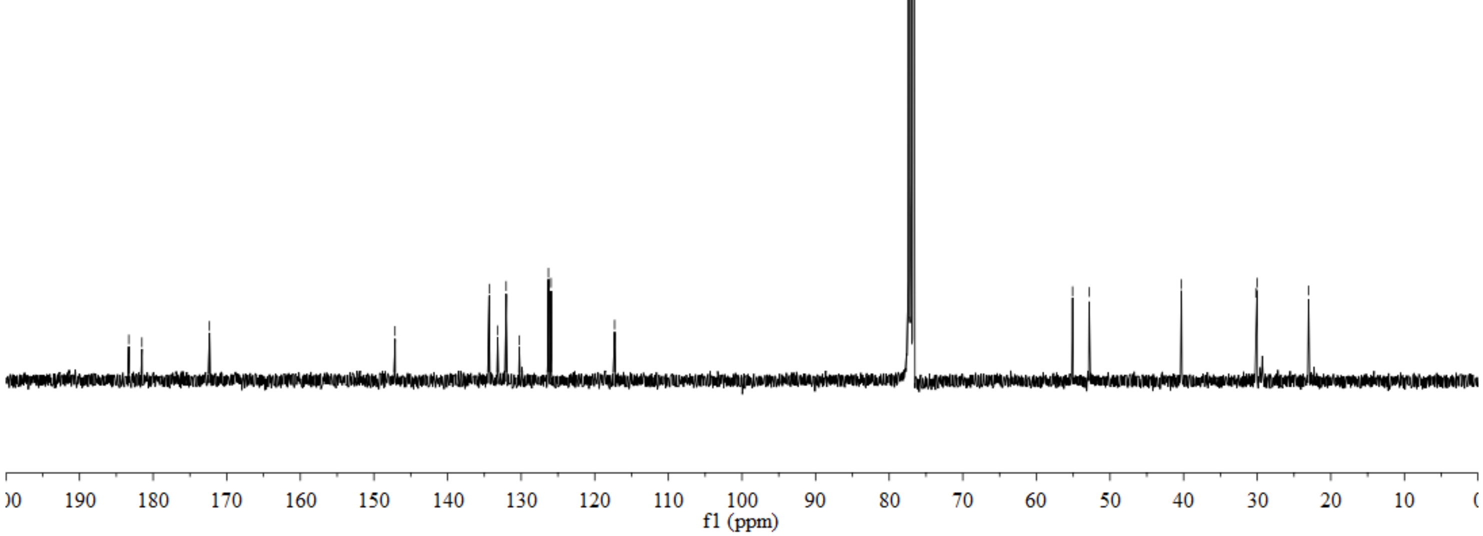

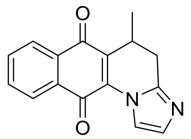

87
^1H NMR (400 MHz, $CDCl_3$)

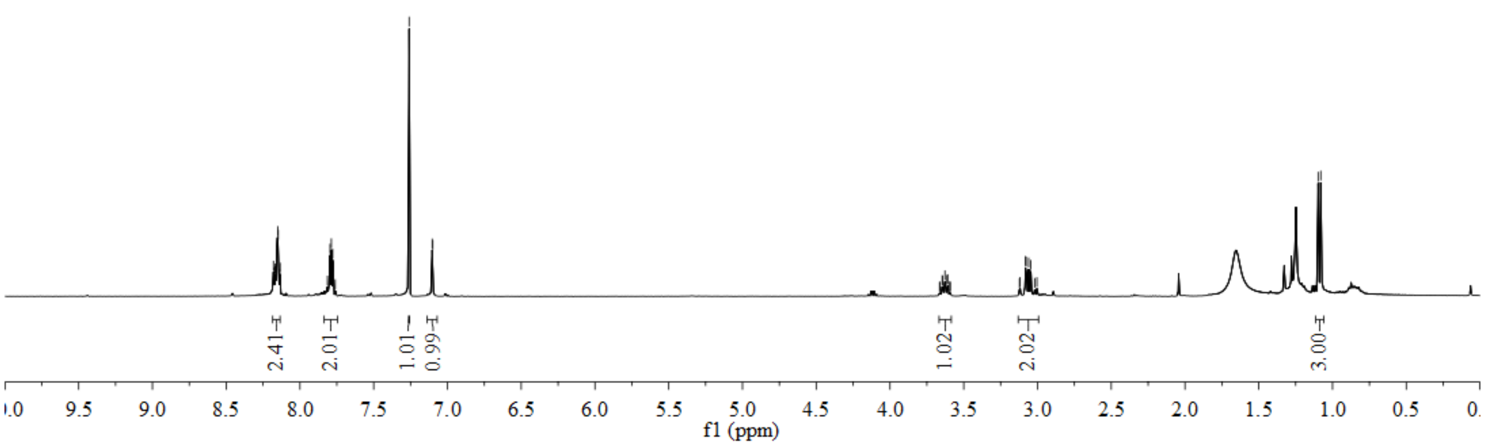

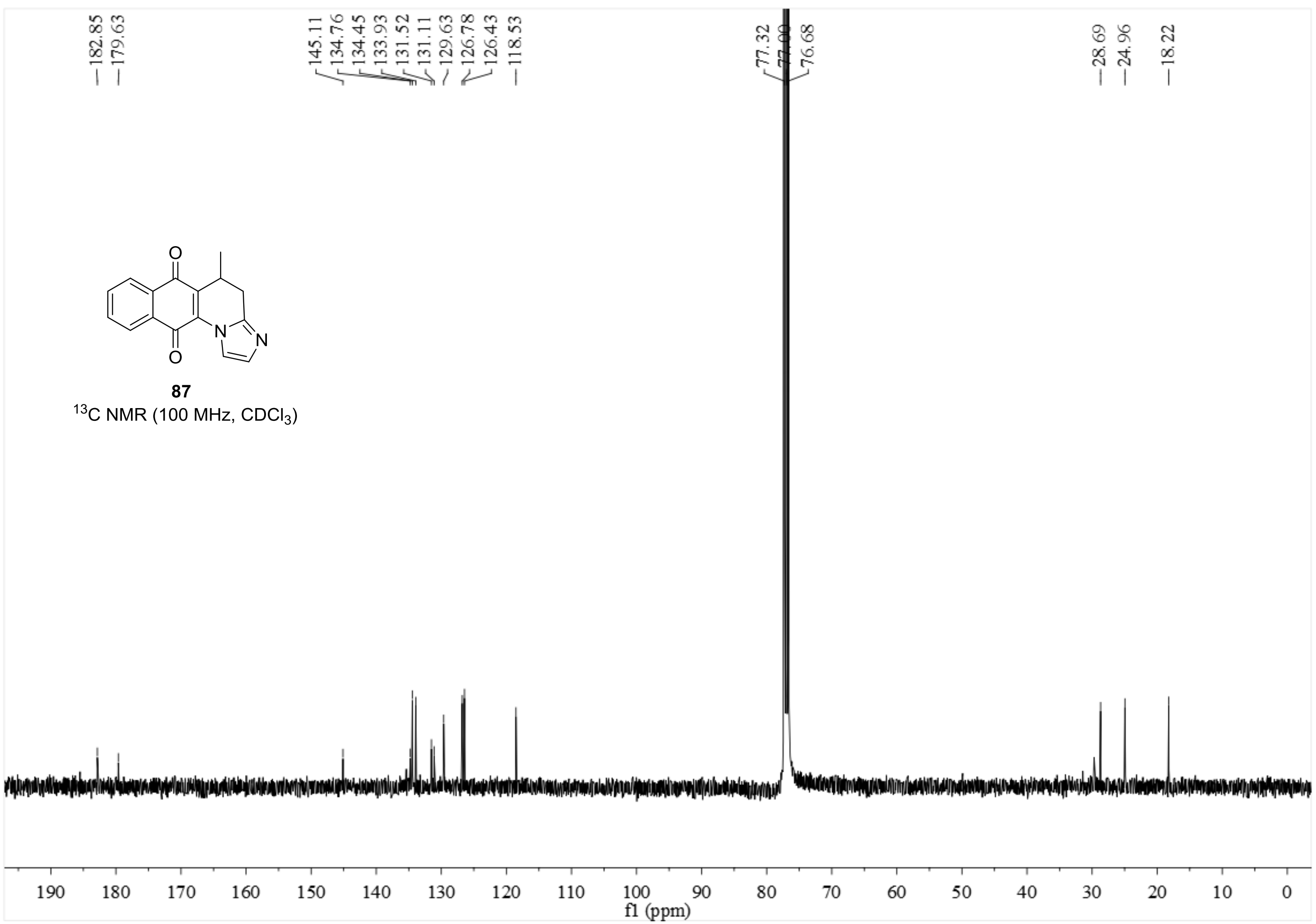

87

^{13}C NMR (100 MHz, $CDCl_3$)

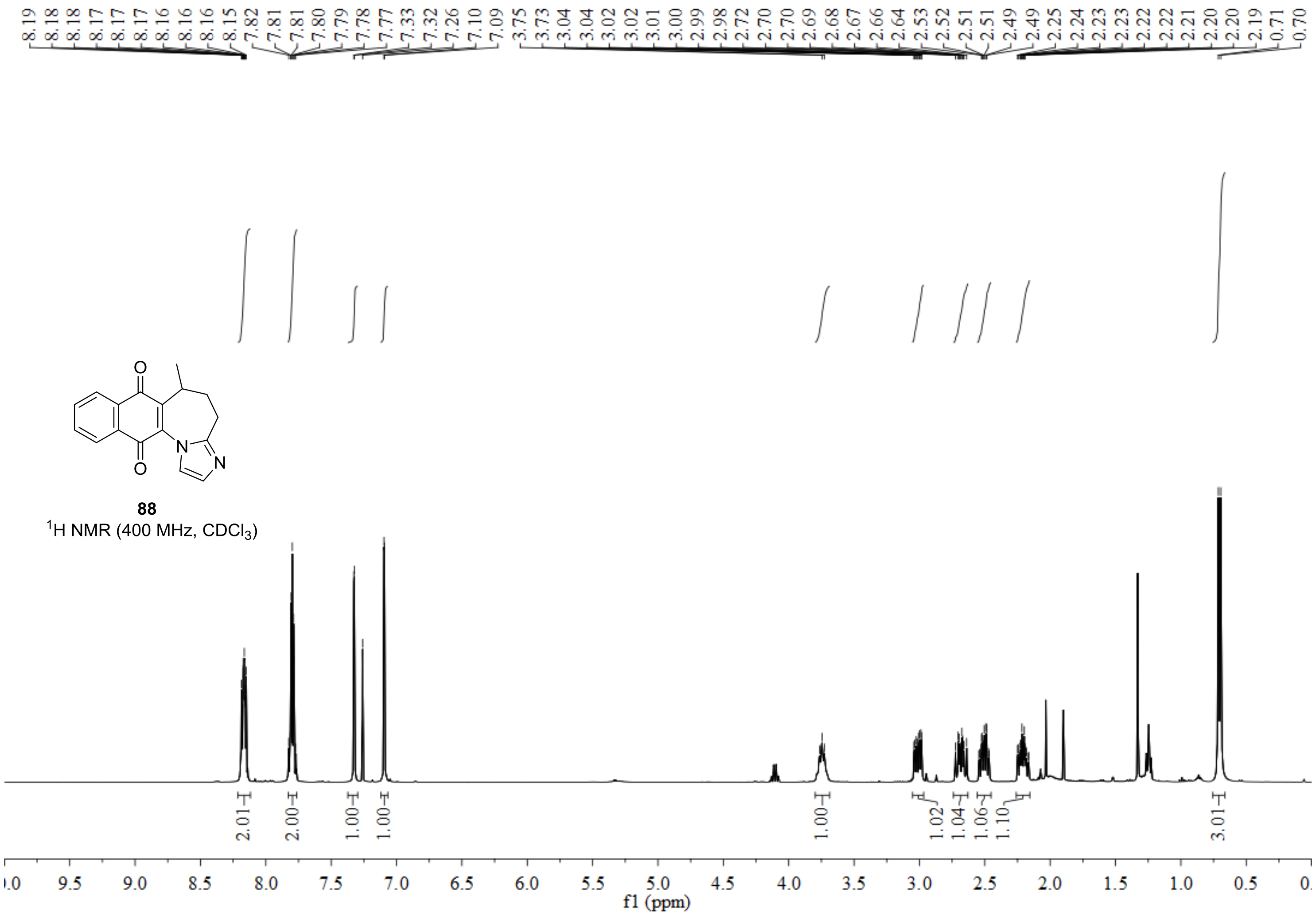

88

^{1}H NMR (400 MHz, $CDCl_3$)

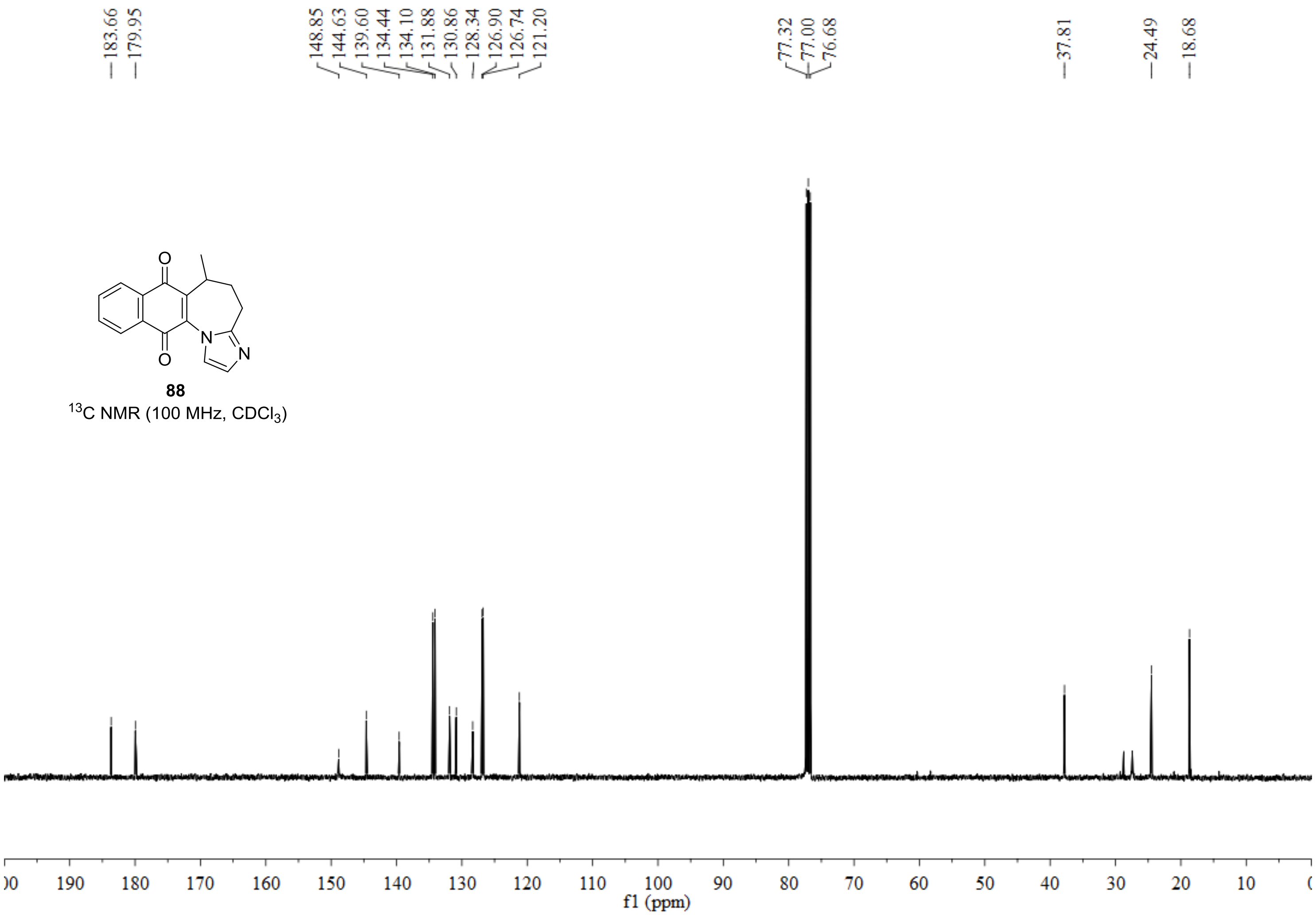
183.66
179.95
148.85
144.63
139.60
134.44
134.10
131.88
130.86
128.34
126.90
126.74
121.20
77.32
77.00
76.68
37.81
24.49
18.68
88
13C NMR (100 MHz, CDCl3)
f1 (ppm)

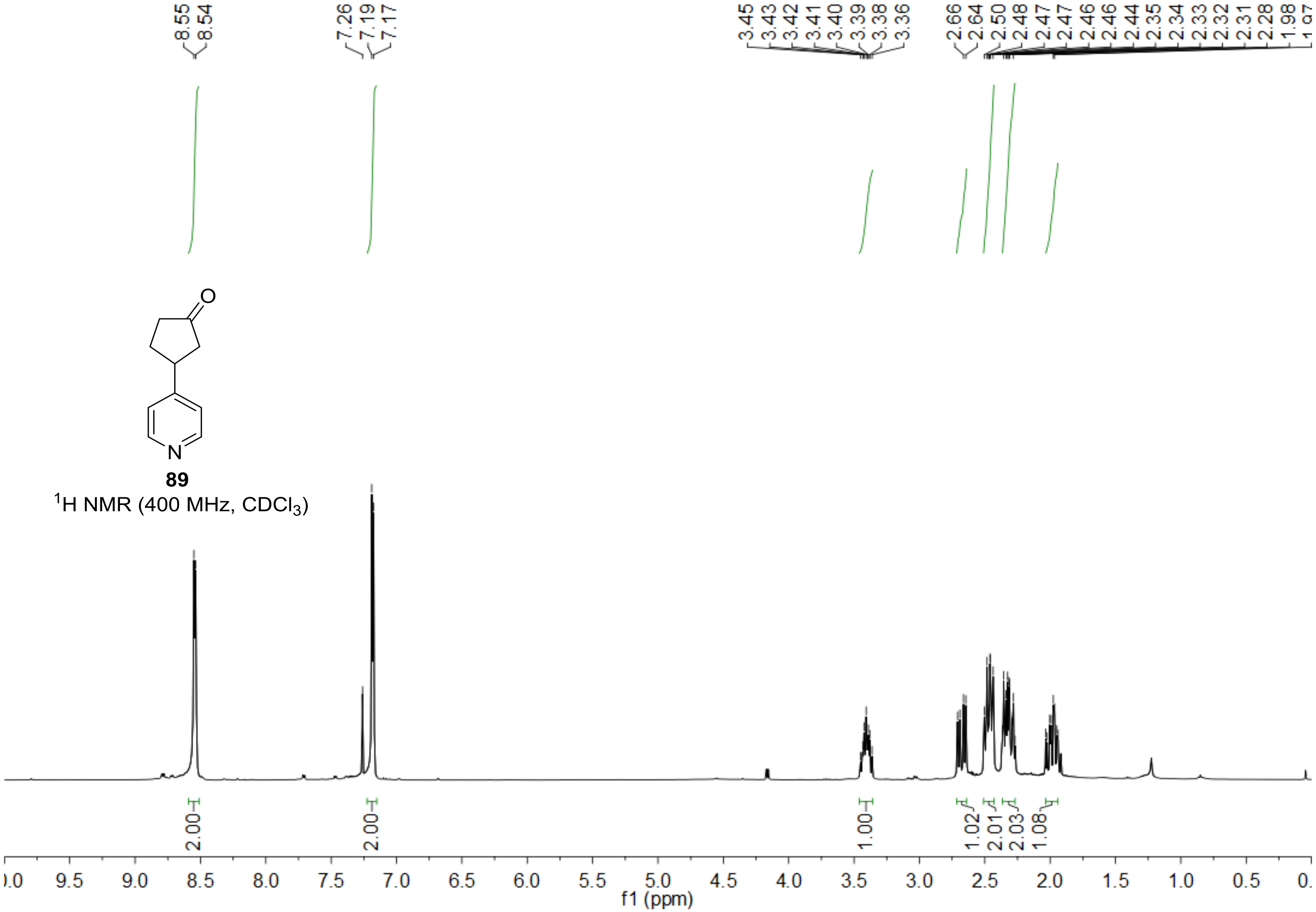
8.55
8.54
7.26
7.19
7.17
3.45
3.43
3.42
3.41
3.40
3.39
3.38
3.36
2.66
2.64
2.50
2.48
2.47
2.47
2.46
2.46
2.44
2.35
2.34
2.33
2.32
2.31
2.28
1.98
O
N
89
^{1}H NMR (400 MHz, $CDCl_3$)
2.00
2.00
1.00
1.02
2.01
2.03
1.08
9.5
9.0
8.5
8.0
7.5
7.0
6.5
6.0
5.5
5.0
4.5
4.0
3.5
3.0
2.5
2.0
1.5
1.0
0.5
f1 (ppm)

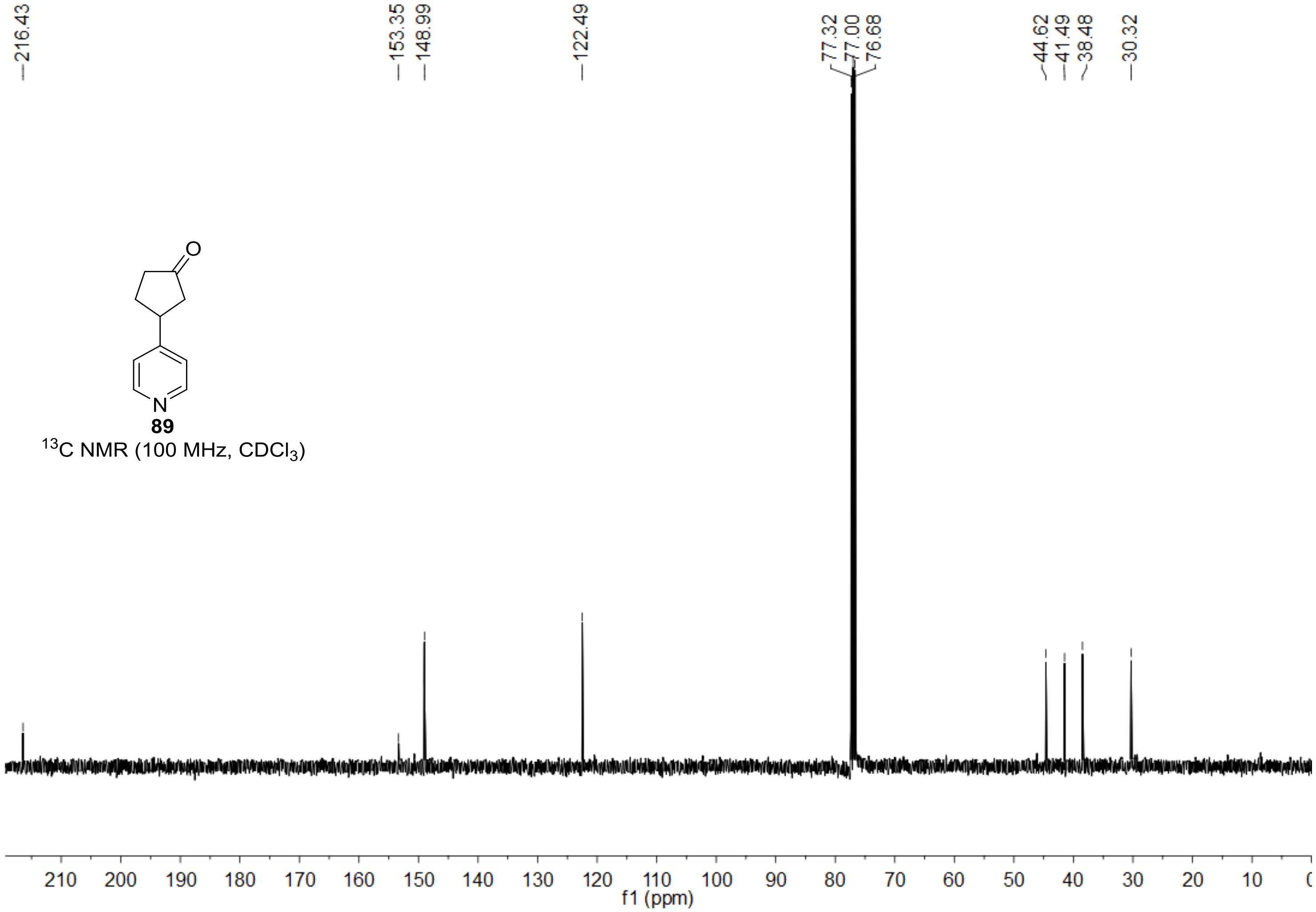

89

^{13}C NMR (100 MHz, $CDCl_3$)

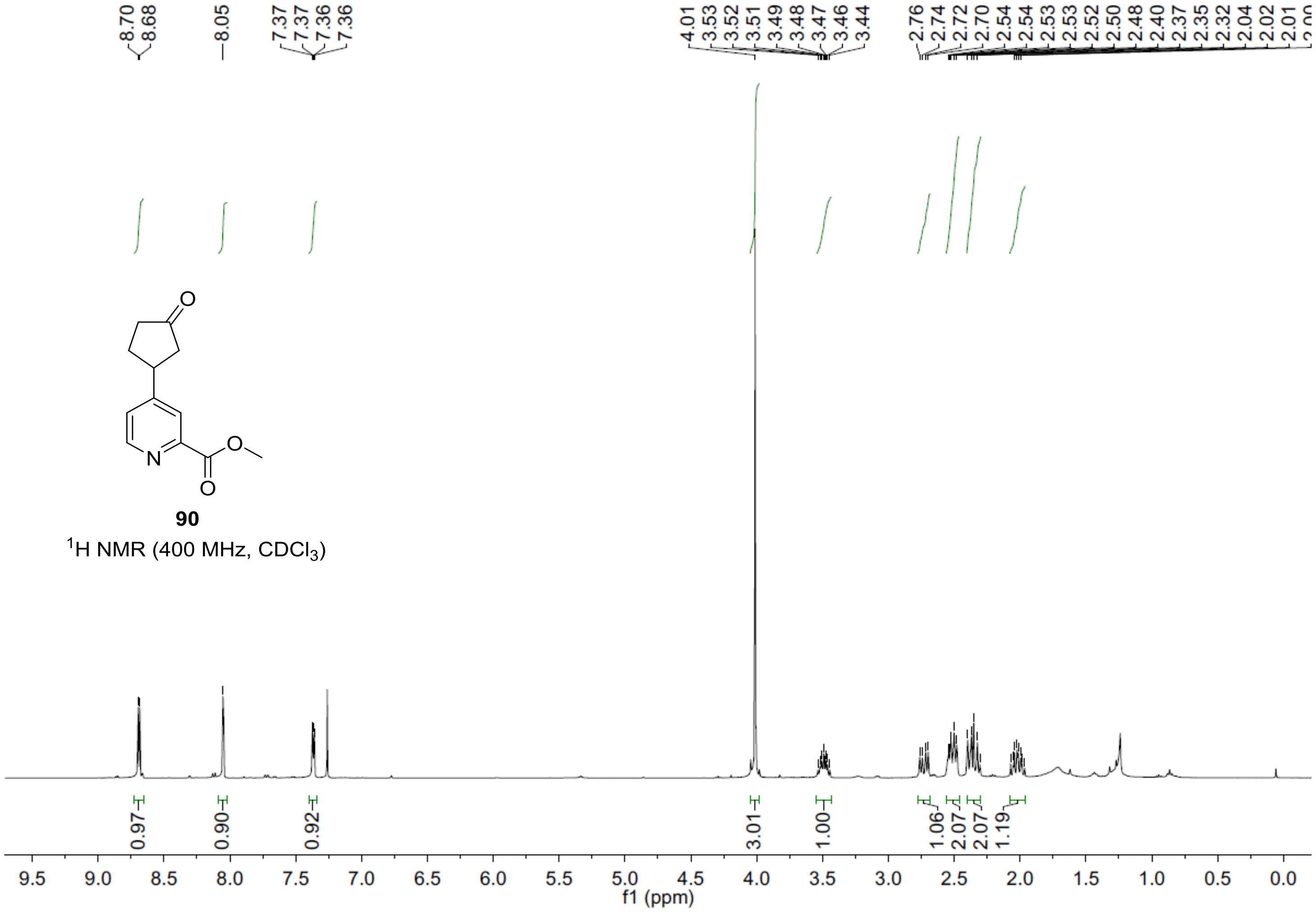

90

^{1}H NMR (400 MHz, $CDCl_3$)

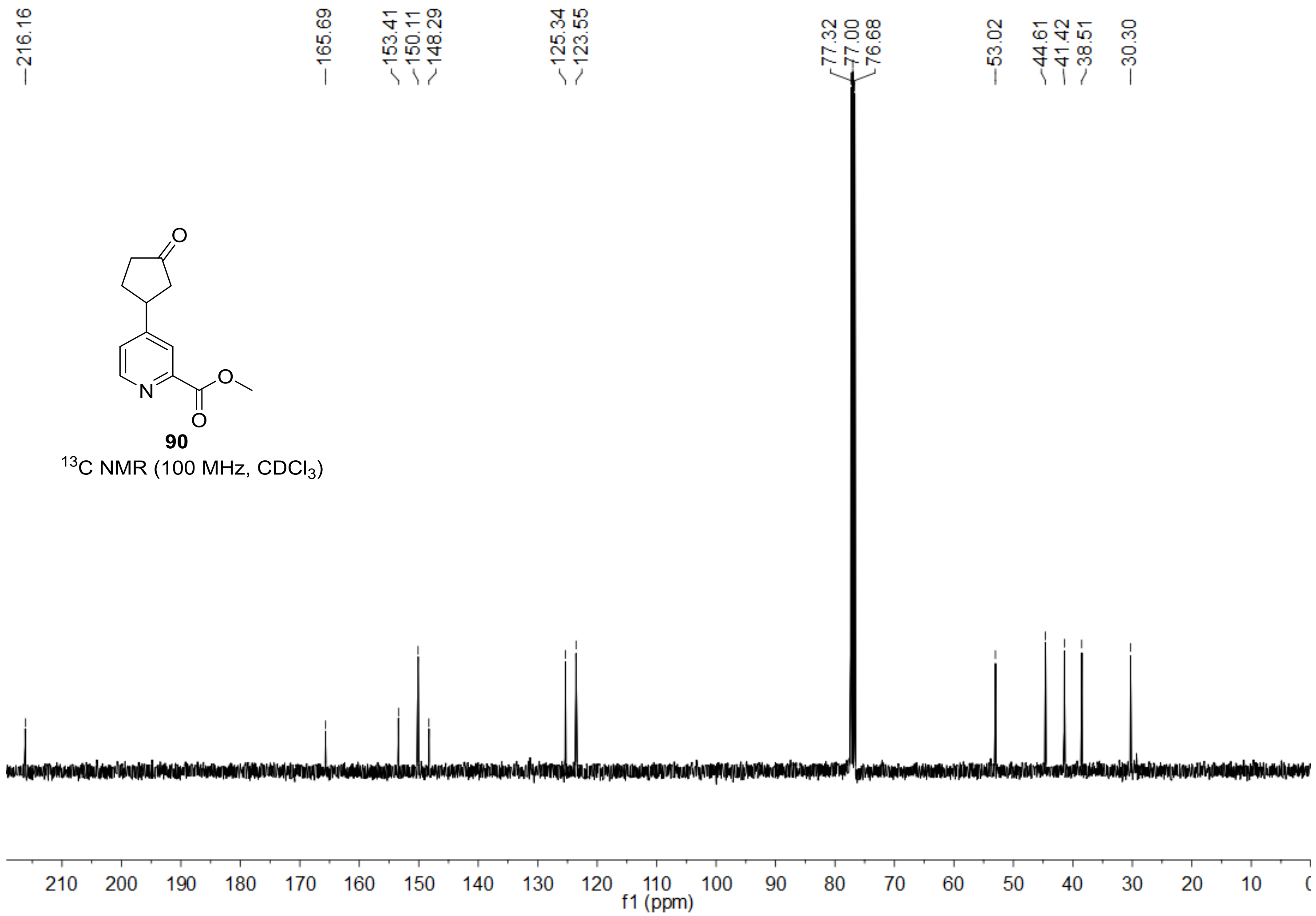

90

^{13}C NMR (100 MHz, $CDCl_3$)

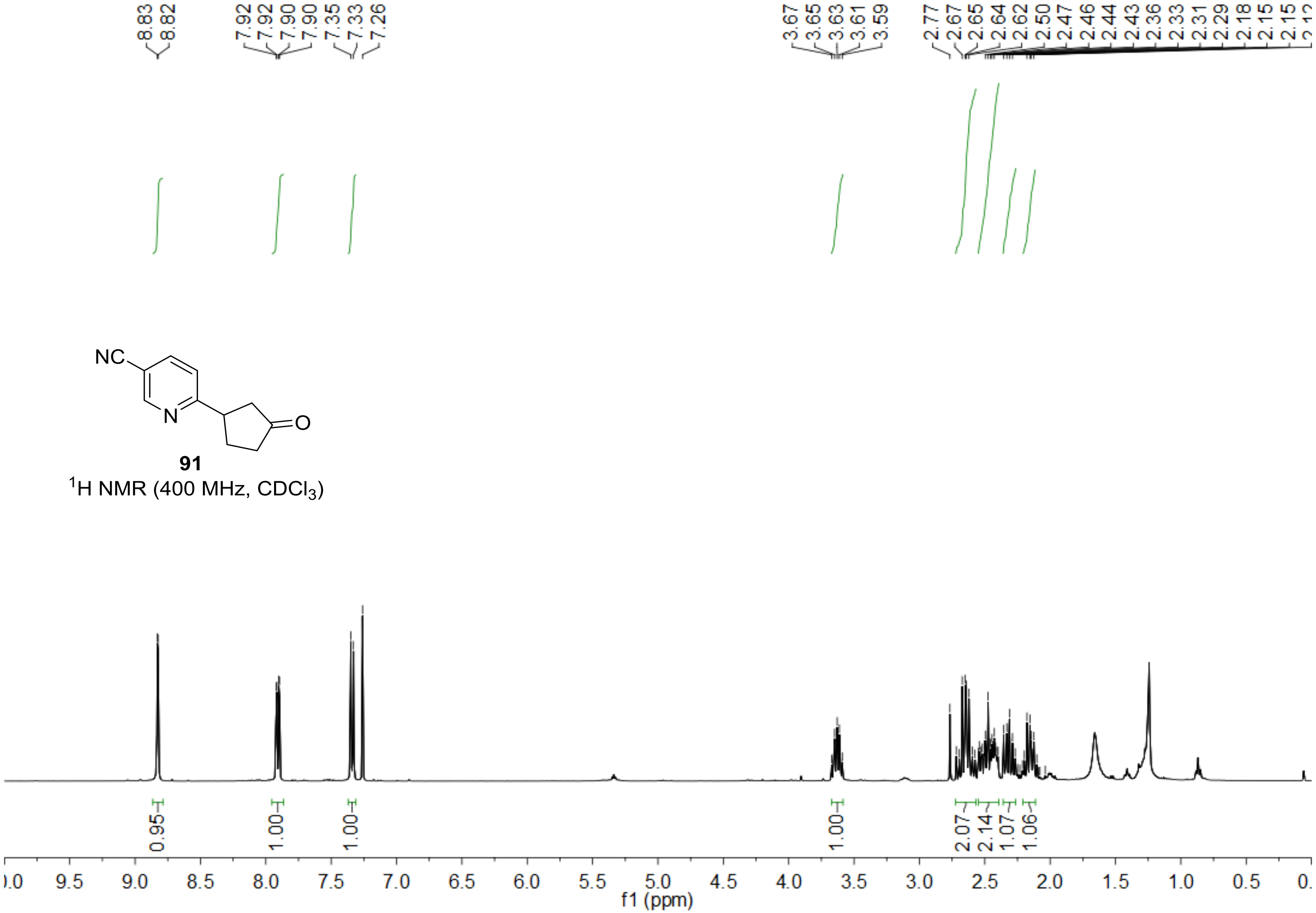
8.83
8.82
7.92
7.92
7.90
7.90
7.35
7.33
7.26
3.67
3.65
3.63
3.61
3.59
2.77
2.67
2.65
2.64
2.62
2.50
2.47
2.46
2.44
2.43
2.36
2.33
2.31
2.29
2.18
2.15
2.15
NC
N
O
91
1H NMR (400 MHz, CDCl3)
0.95
1.00
1.00
1.00
2.07
2.14
1.07
1.06
9.5
9.0
8.5
8.0
7.5
7.0
6.5
6.0
5.5
5.0
4.5
4.0
3.5
3.0
2.5
2.0
1.5
1.0
0.5
f1 (ppm)

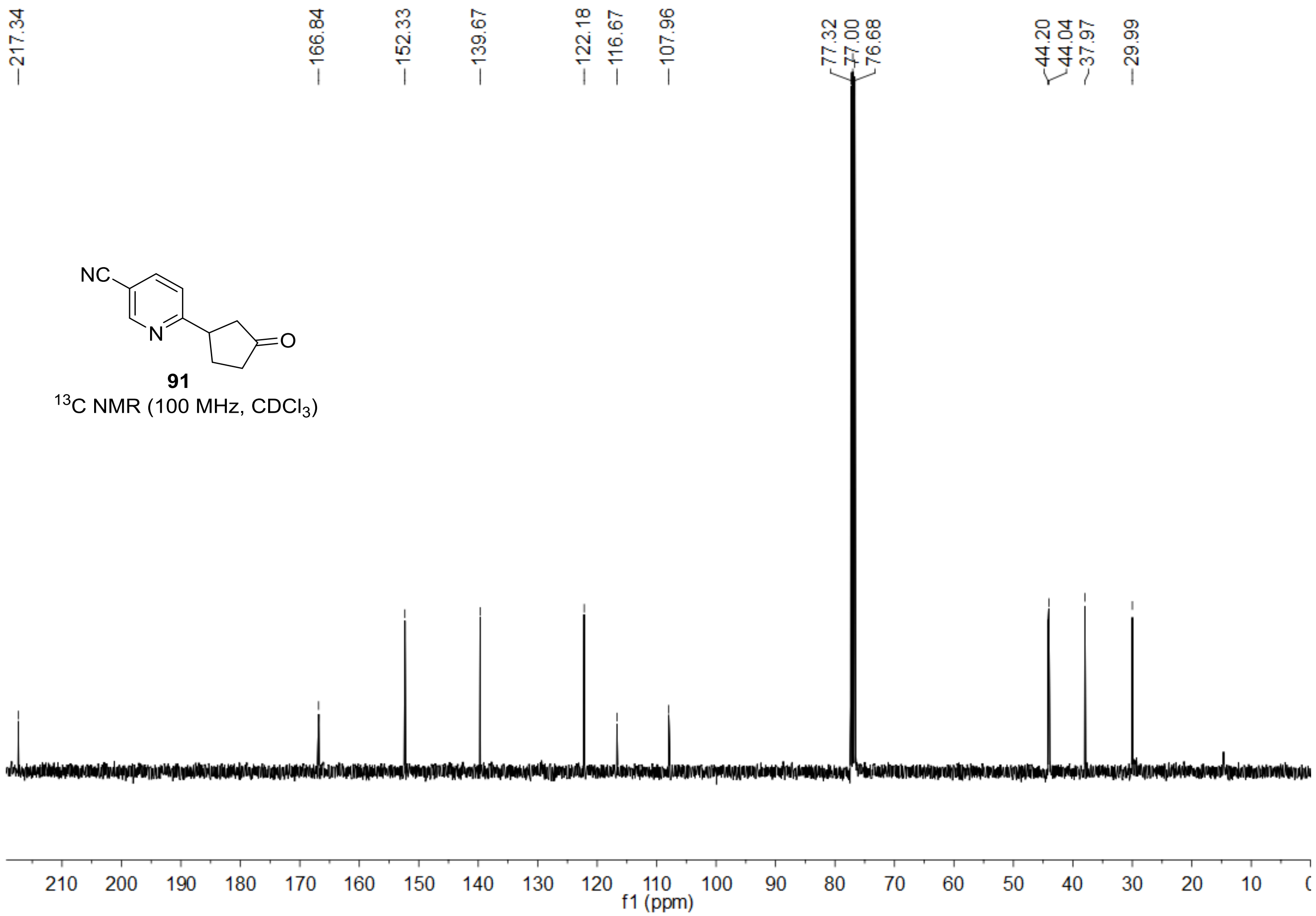
217.34
166.84
152.33
139.67
122.18
116.67
107.96
77.32
77.00
76.68
44.20
44.04
37.97
29.99
NC
N
O
91
13C NMR (100 MHz, CDCl3)
210 200 190 180 170 160 150 140 130 120 110 100 90 80 70 60 50 40 30 20 10
f1 (ppm)

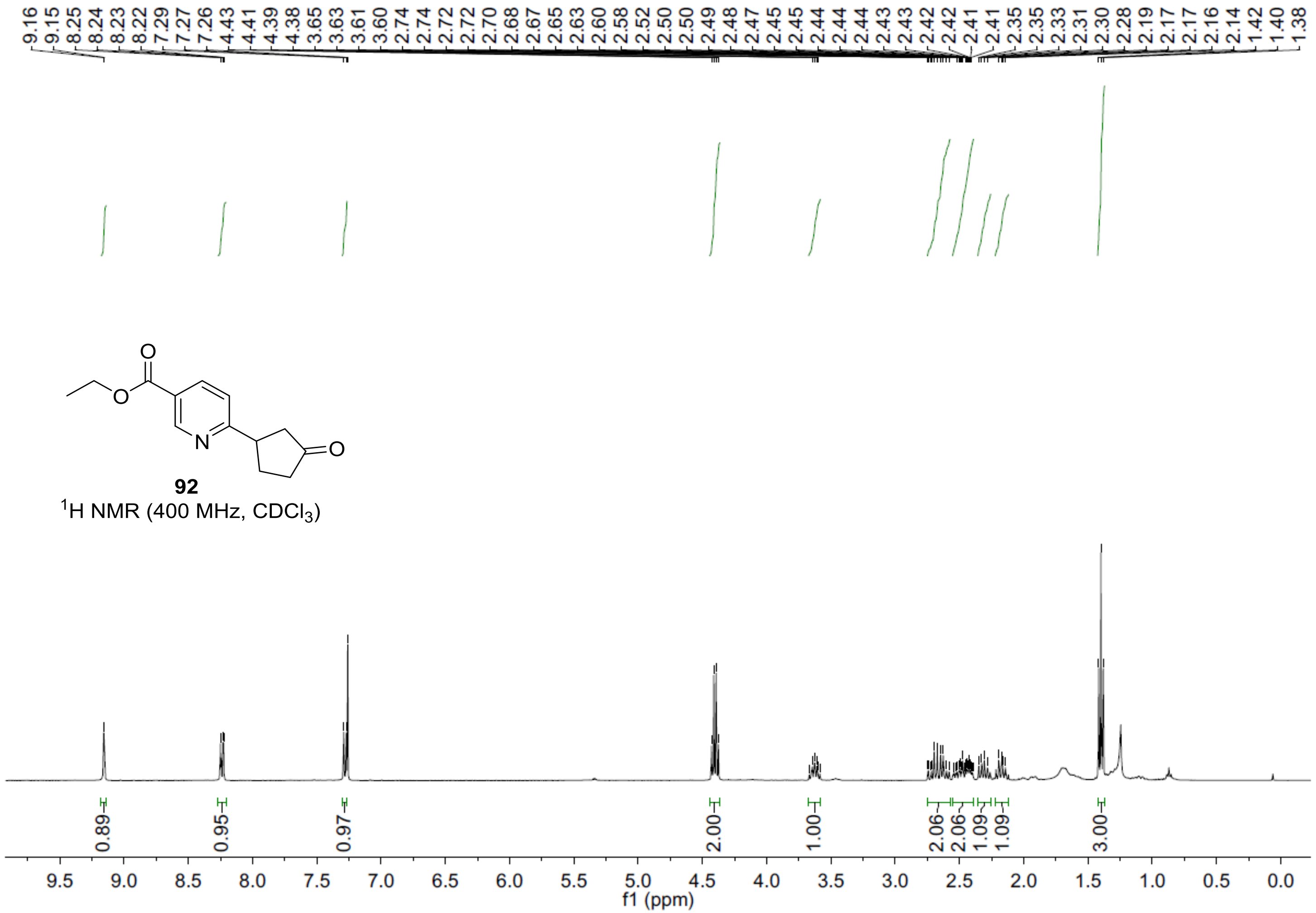

92

^{1}H NMR (400 MHz, $CDCl_3$)

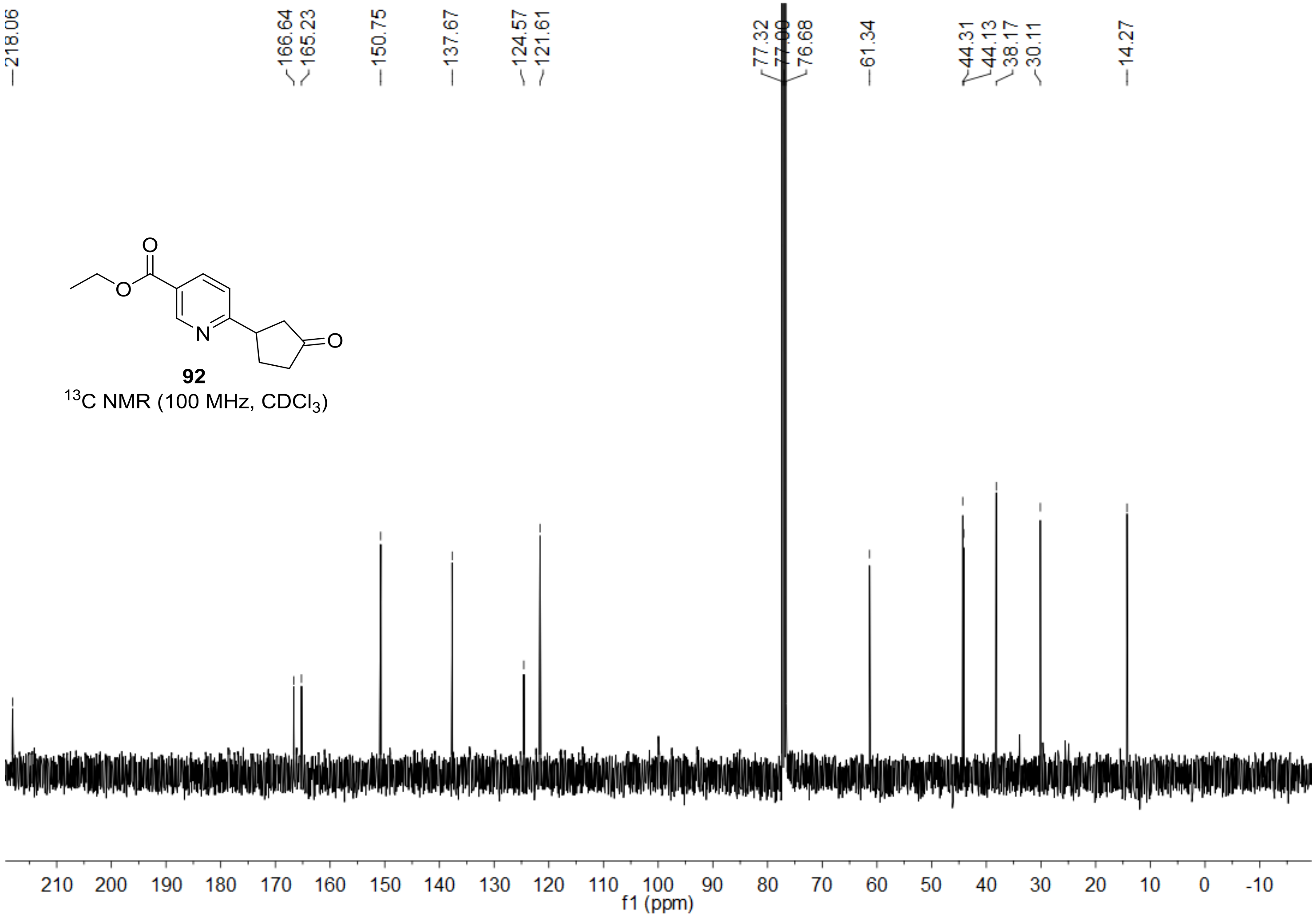
218.06
166.64
165.23
150.75
137.67
124.57
121.61
77.32
77.00
76.68
61.34
44.31
44.13
38.17
30.11
14.27
O
O
O
N
O
92
13C NMR (100 MHz, CDCl3)
210 200 190 180 170 160 150 140 130 120 110 100 90 80 70 60 50 40 30 20 10 0 -10
f1 (ppm)

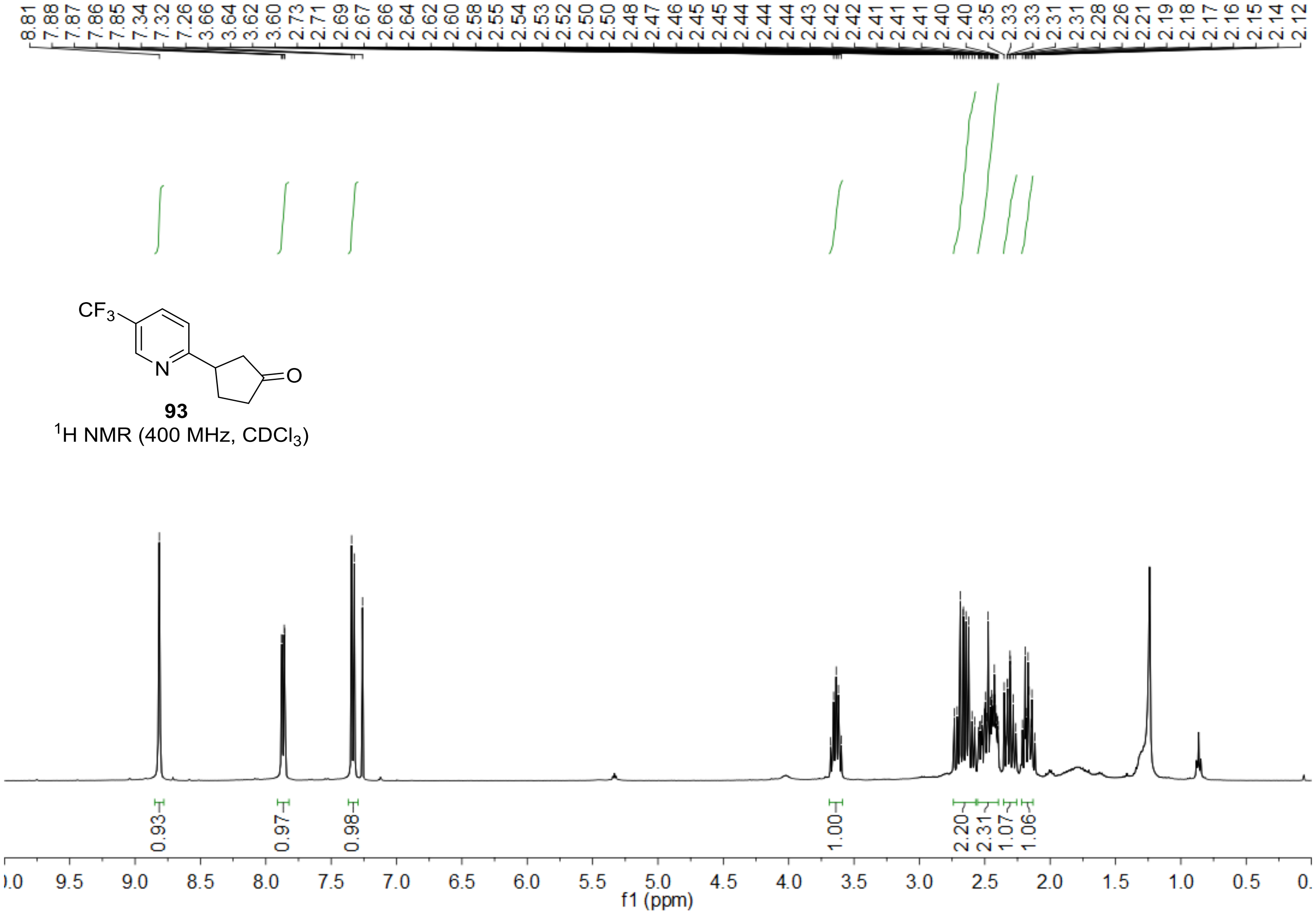
8.81 7.88 7.87 7.86 7.85 7.34 7.32 7.26 3.66 3.64 3.62 3.60 2.73 2.71 2.69 2.67 2.66 2.64 2.62 2.60 2.58 2.55 2.54 2.53 2.52 2.50 2.50 2.48 2.47 2.46 2.45 2.45 2.44 2.44 2.44 2.43 2.42 2.42 2.41 2.41 2.41 2.40 2.40 2.35 2.33 2.33 2.31 2.31 2.28 2.26 2.21 2.19 2.18 2.17 2.16 2.15 2.14 2.12
CF3
N
O
93
1H NMR (400 MHz, CDCl3)
0.93
0.97
0.98
1.00
2.20
2.31
1.07
1.06
f1 (ppm)

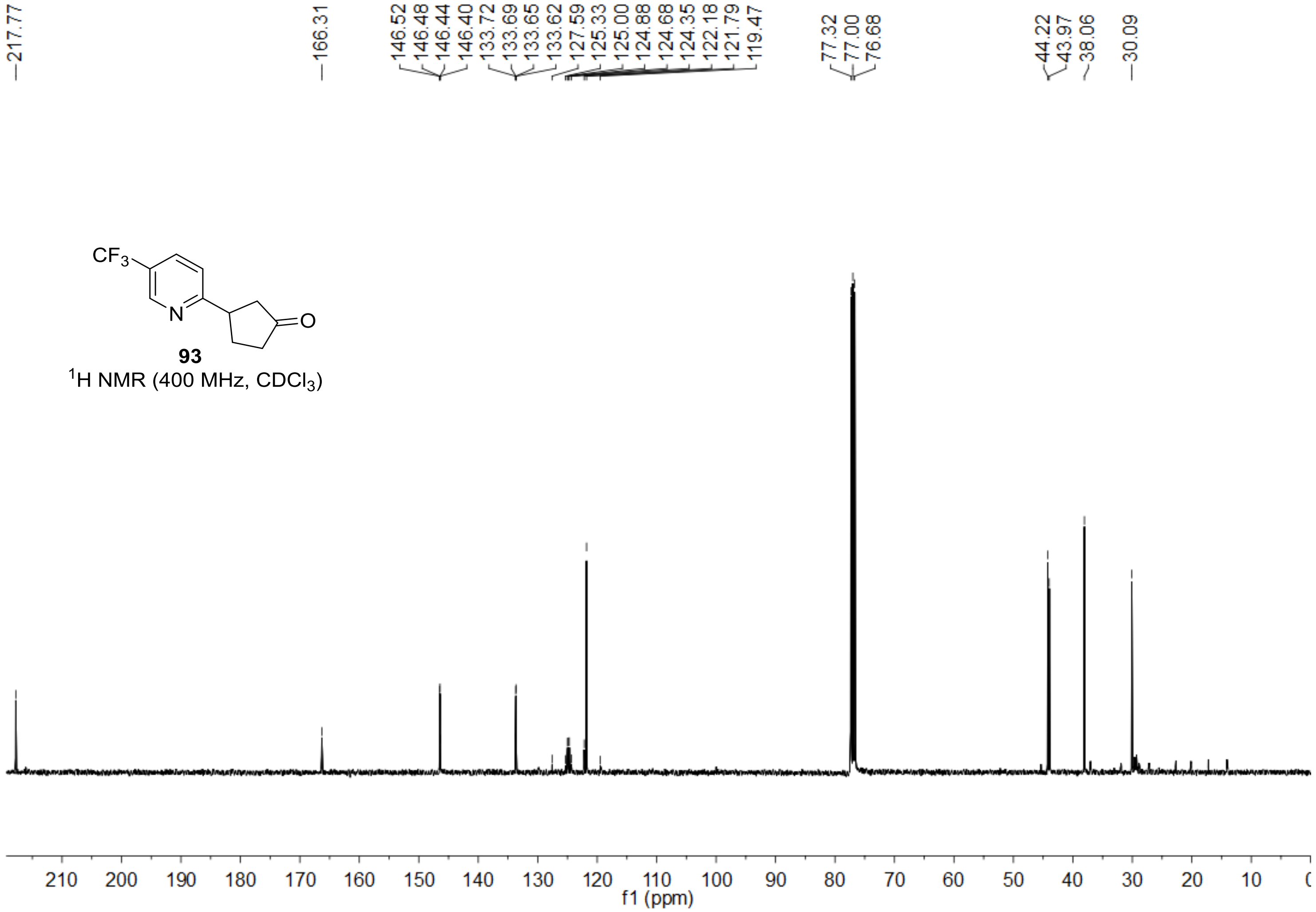
CF3
N
O
93
1H NMR (400 MHz, CDCl3)
217.77
166.31
146.52
146.48
146.44
146.40
133.72
133.69
133.65
133.62
127.59
125.33
125.00
124.88
124.68
124.35
122.18
121.79
119.47
77.32
77.00
76.68
44.22
43.97
38.06
30.09
f1 (ppm)

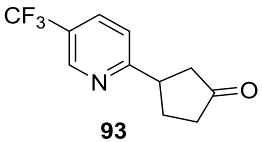

93

^{19}F NMR (376 MHz, $CDCl_3$)

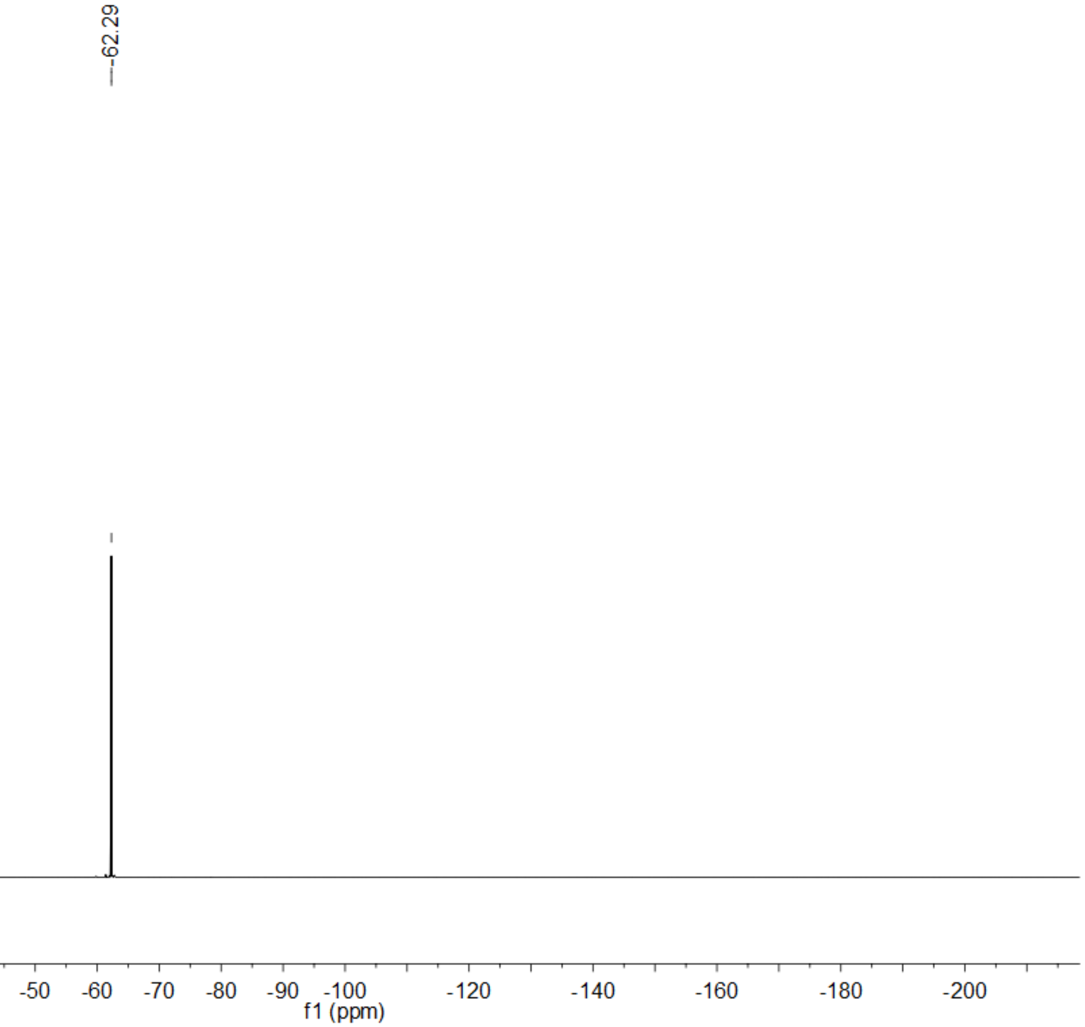

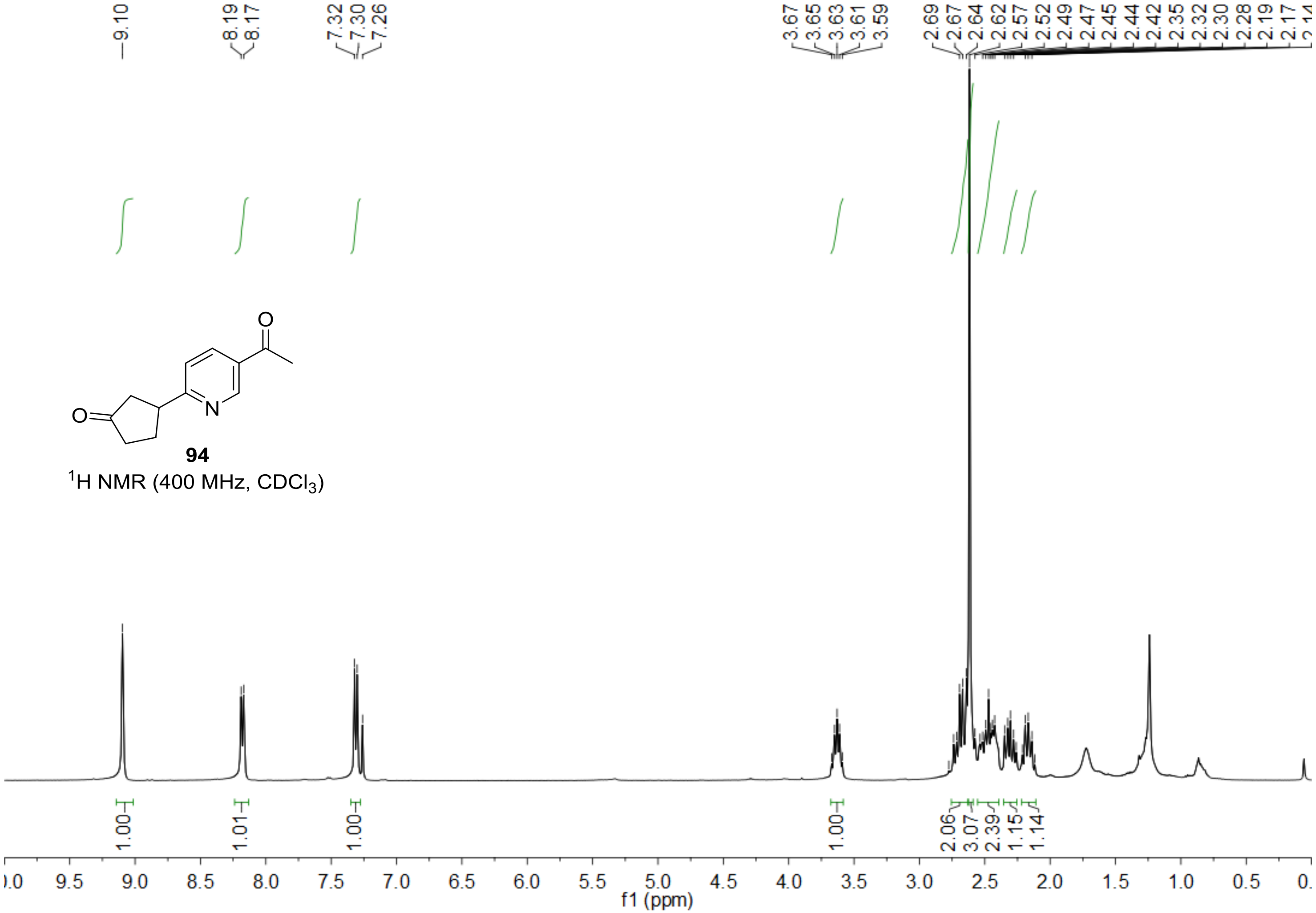
94
^{1}H NMR (400 MHz, CDCl$_3$)
9.10
8.19
8.17
7.32
7.30
7.26
3.67
3.65
3.63
3.61
3.59
2.69
2.67
2.64
2.62
2.57
2.52
2.49
2.47
2.45
2.44
2.42
2.35
2.32
2.30
2.28
2.19
2.17
1.00
1.01
1.00
1.00
2.06
3.07
2.39
1.15
1.14
9.5
9.0
8.5
8.0
7.5
7.0
6.5
6.0
5.5
5.0
4.5
4.0
3.5
3.0
2.5
2.0
1.5
1.0
0.5
f1 (ppm)

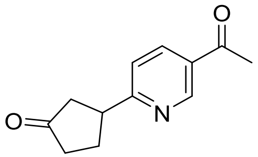

94

^{13}C NMR (100 MHz, $CDCl_3$)

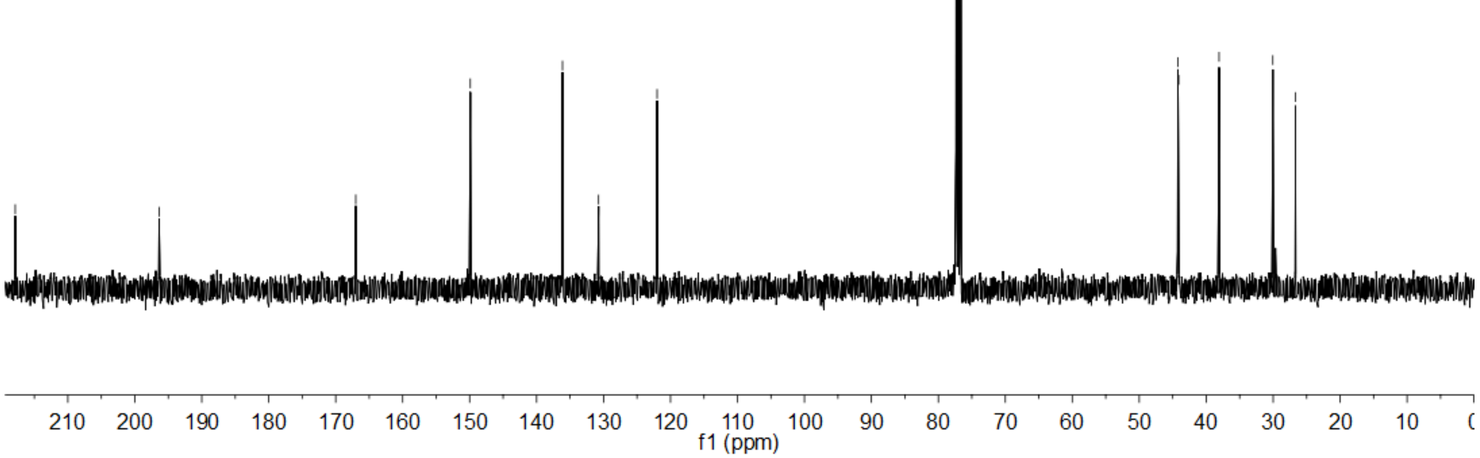

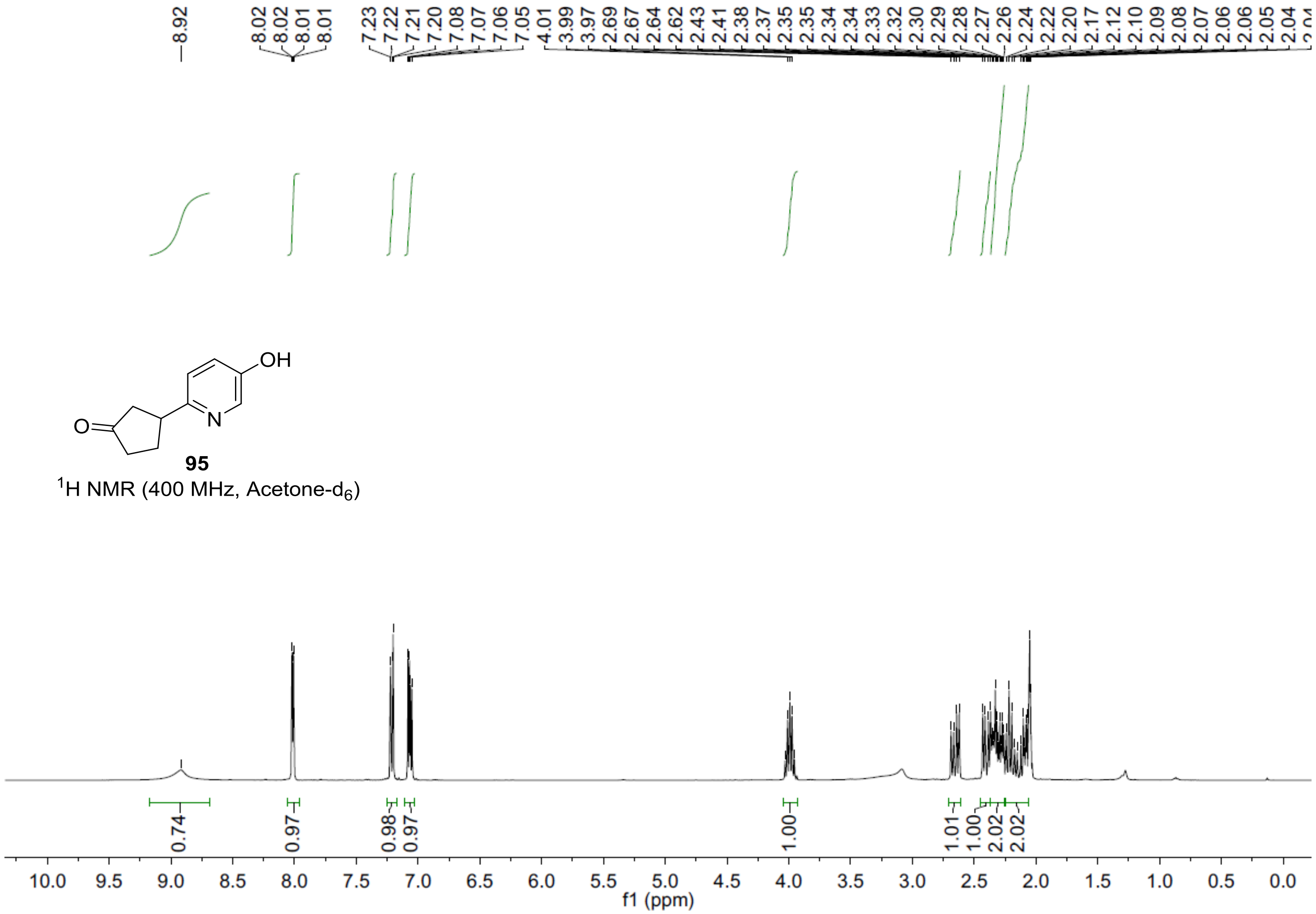

95

^{1}H NMR (400 MHz, Acetone-d_6)

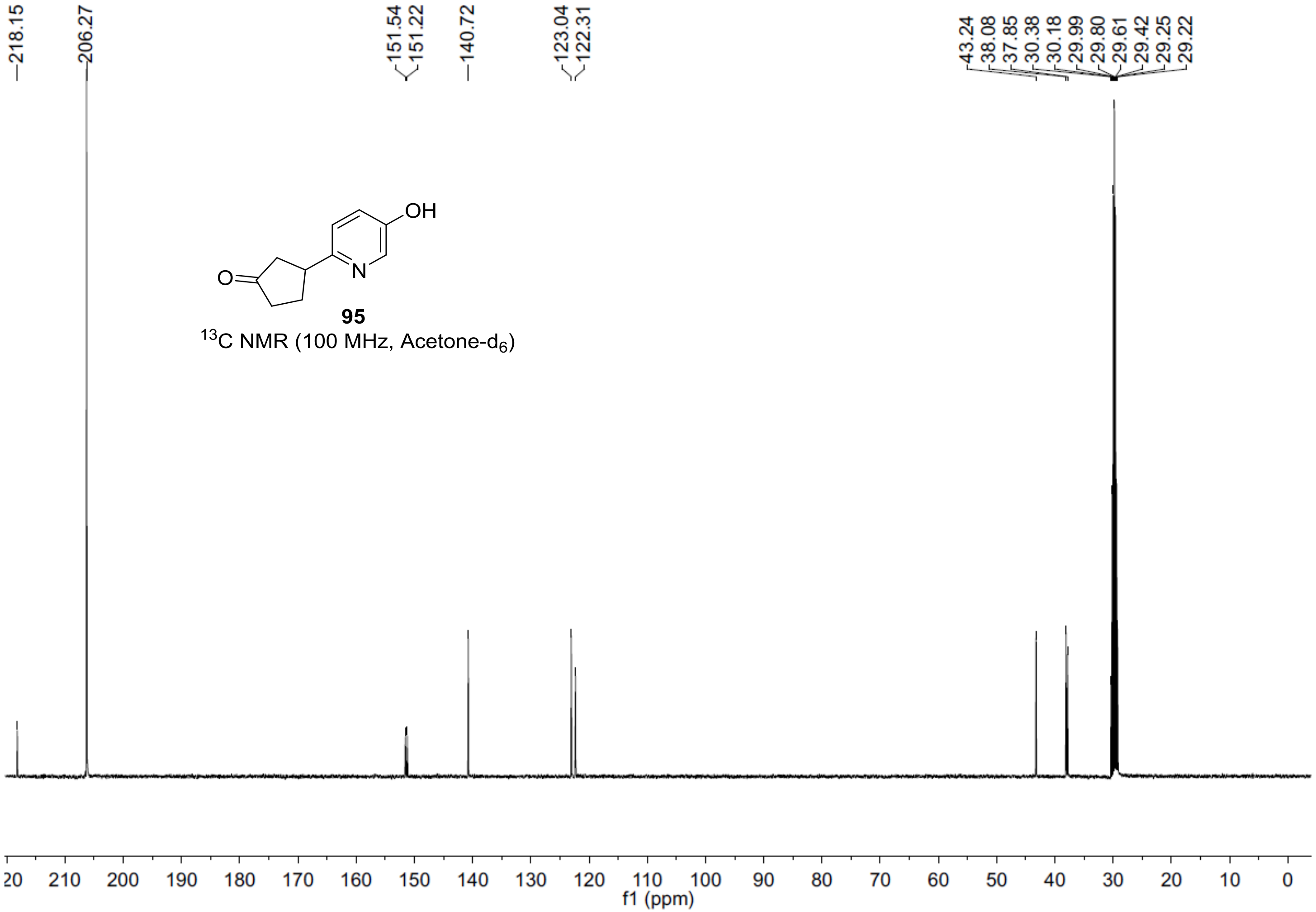

95

^{13}C NMR (100 MHz, Acetone-d_6)

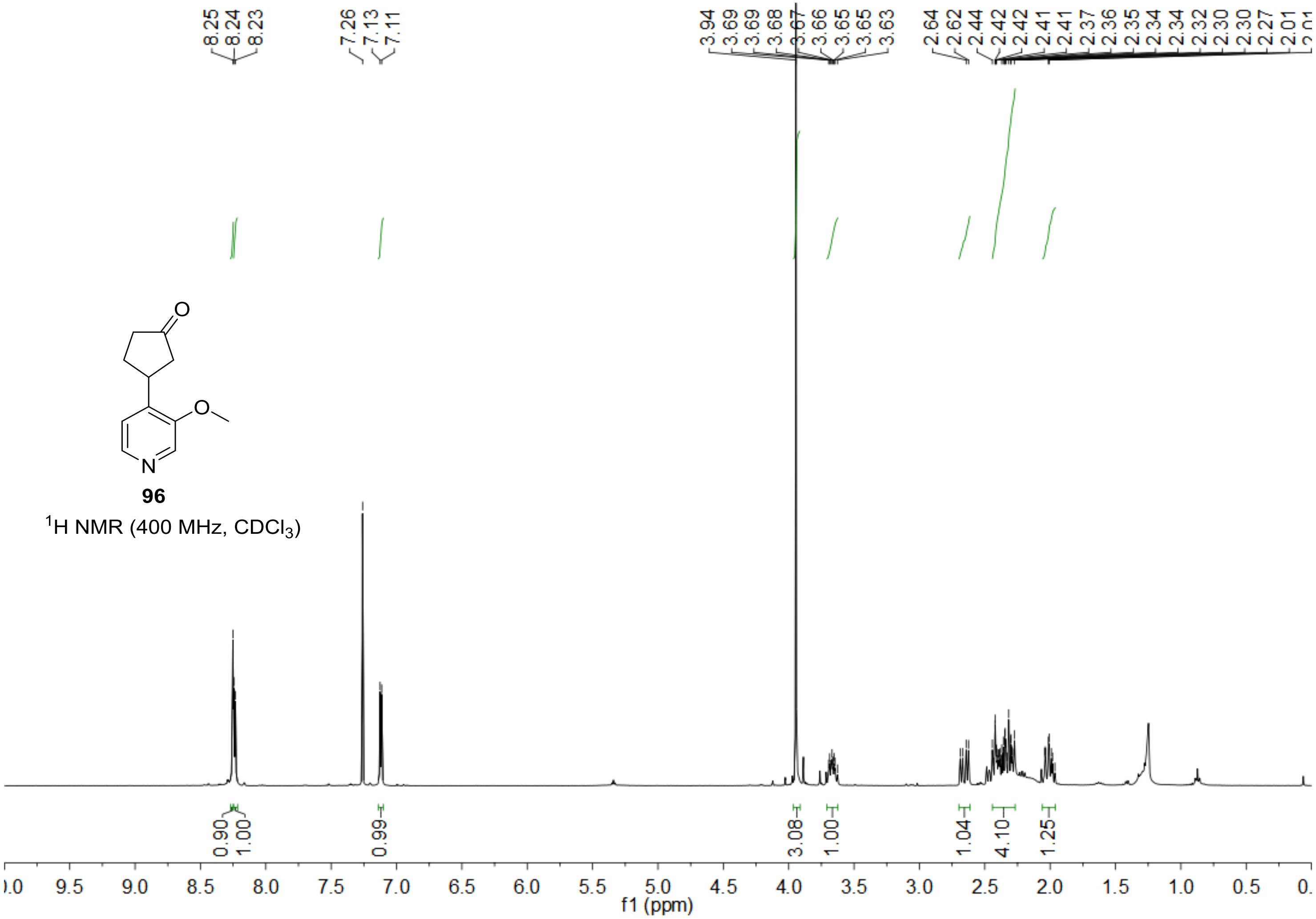

8.25
8.24
8.23
7.26
7.13
7.11
3.94
3.69
3.69
3.68
3.67
3.66
3.65
3.65
3.63
2.64
2.62
2.44
2.42
2.42
2.41
2.41
2.37
2.36
2.35
2.34
2.34
2.32
2.30
2.30
2.27
2.01
O
N
96
^{1}H NMR (400 MHz, CDCl$_3$)
0.90
1.00
0.99
3.08
1.00
1.04
4.10
1.25
9.5
9.0
8.5
8.0
7.5
7.0
6.5
6.0
5.5
5.0
4.5
4.0
3.5
3.0
2.5
2.0
1.5
1.0
0.5
f1 (ppm)

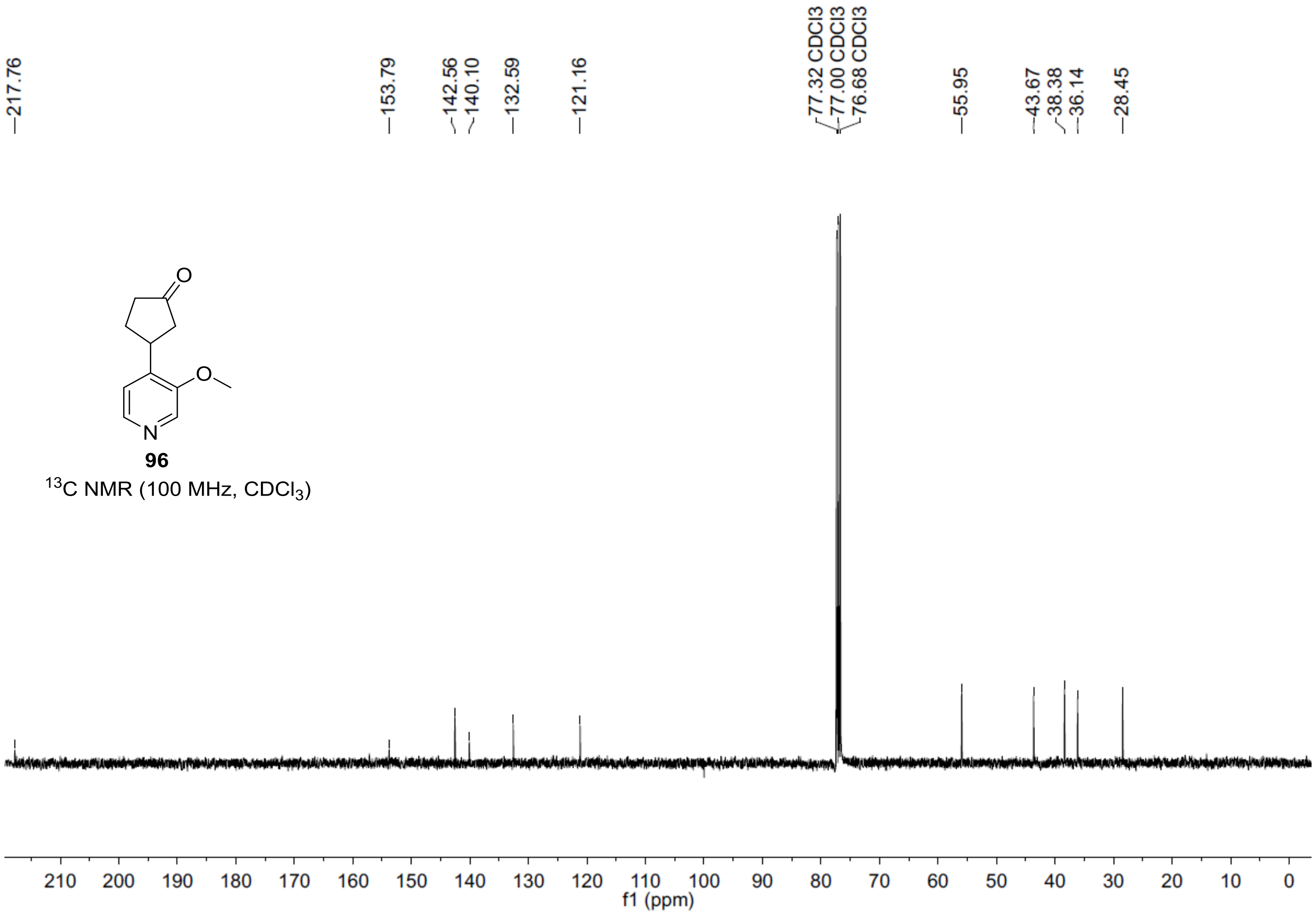

96

^{13}C NMR (100 MHz, $CDCl_3$)

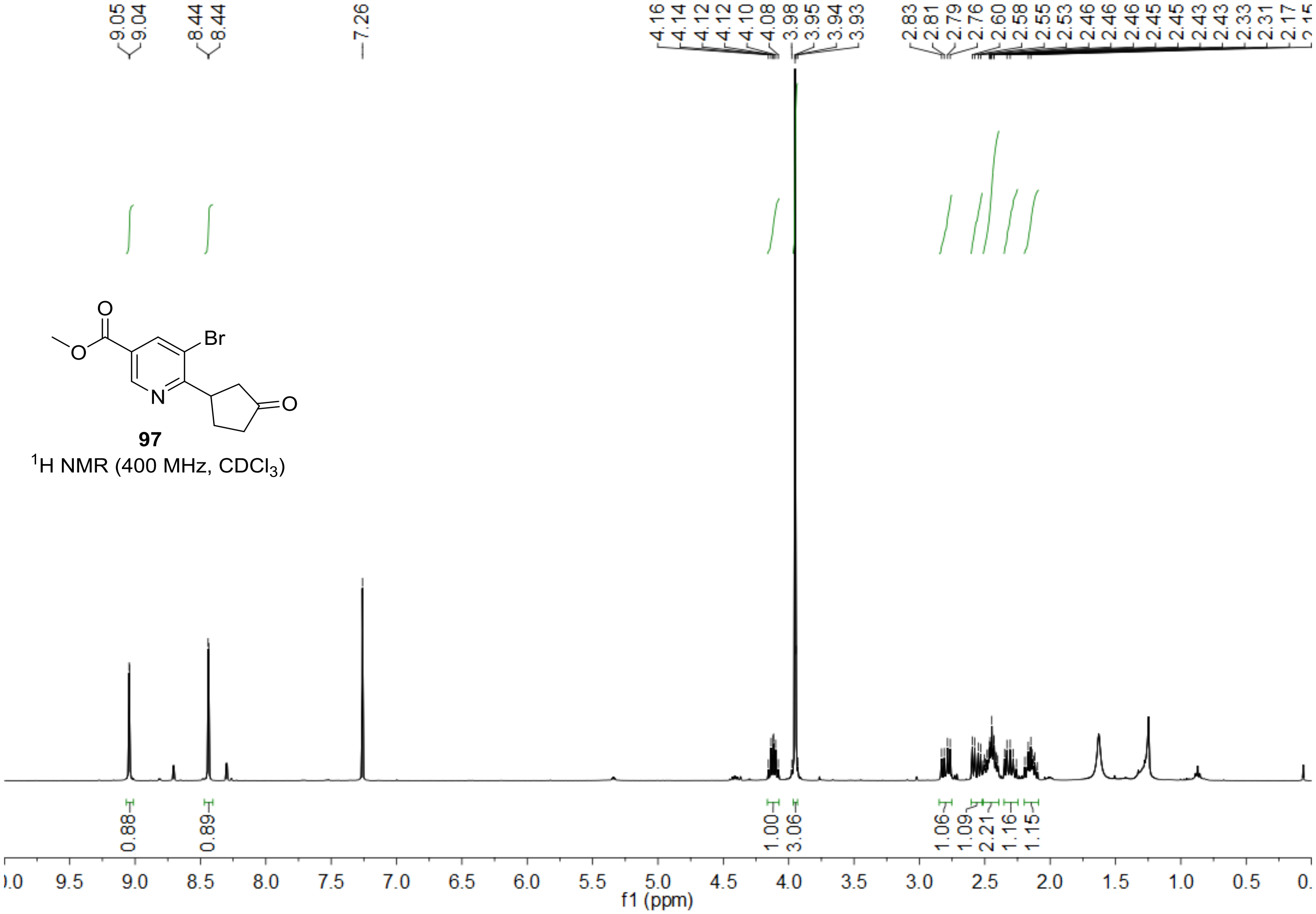

97

^{1}H NMR (400 MHz, $CDCl_3$)

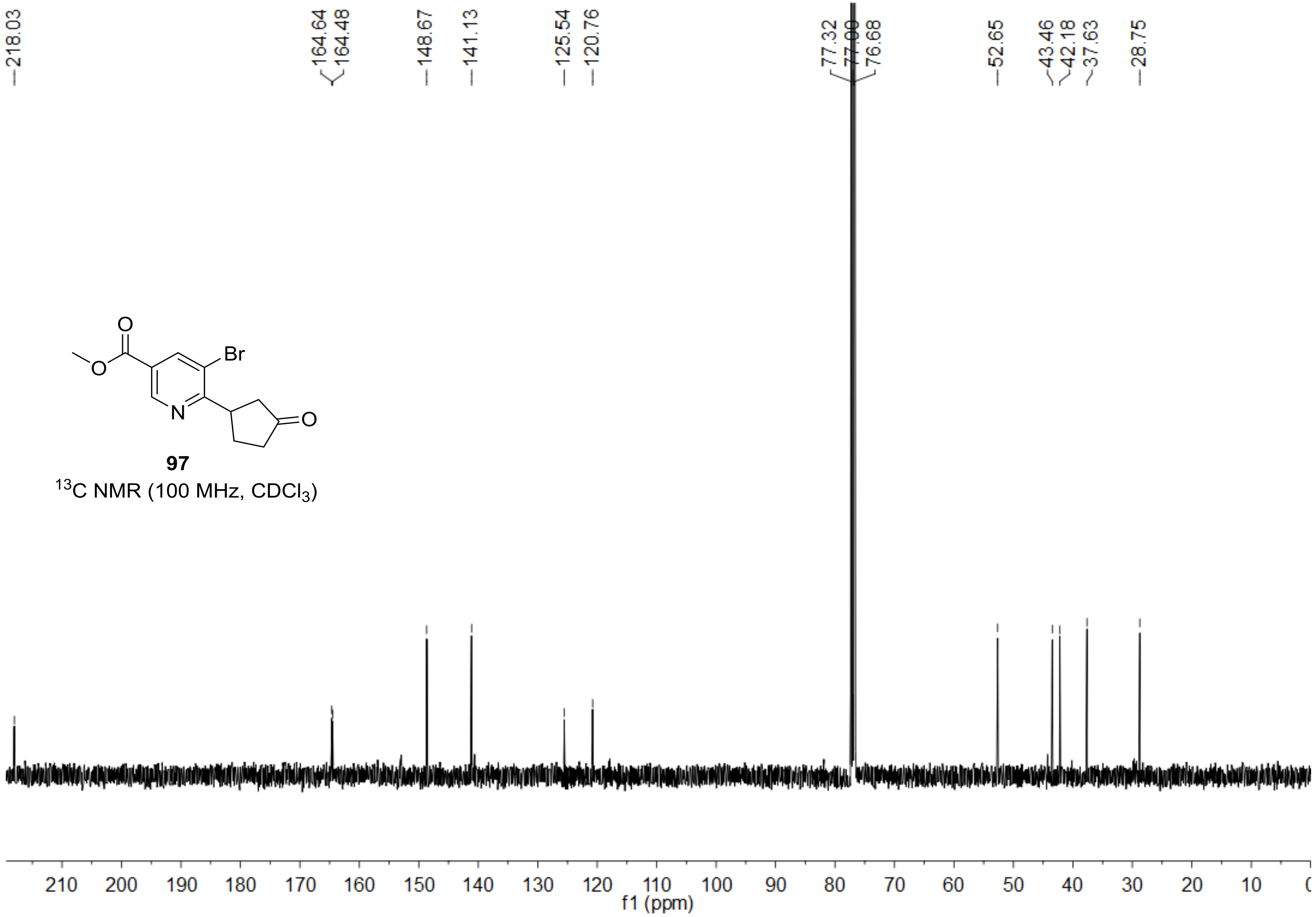

97

^{13}C NMR (100 MHz, $CDCl_3$)

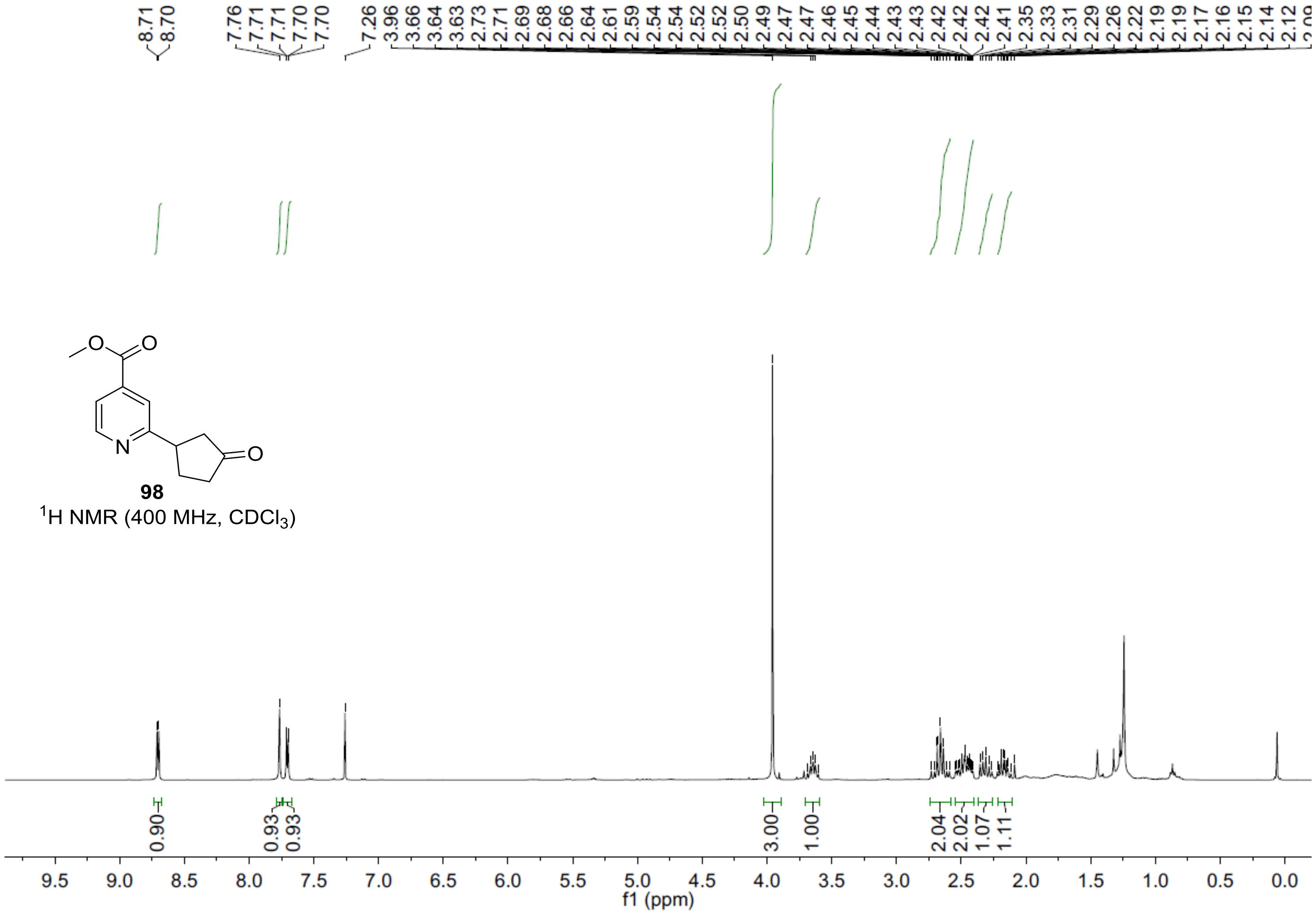

98

^{1}H NMR (400 MHz, $CDCl_3$)

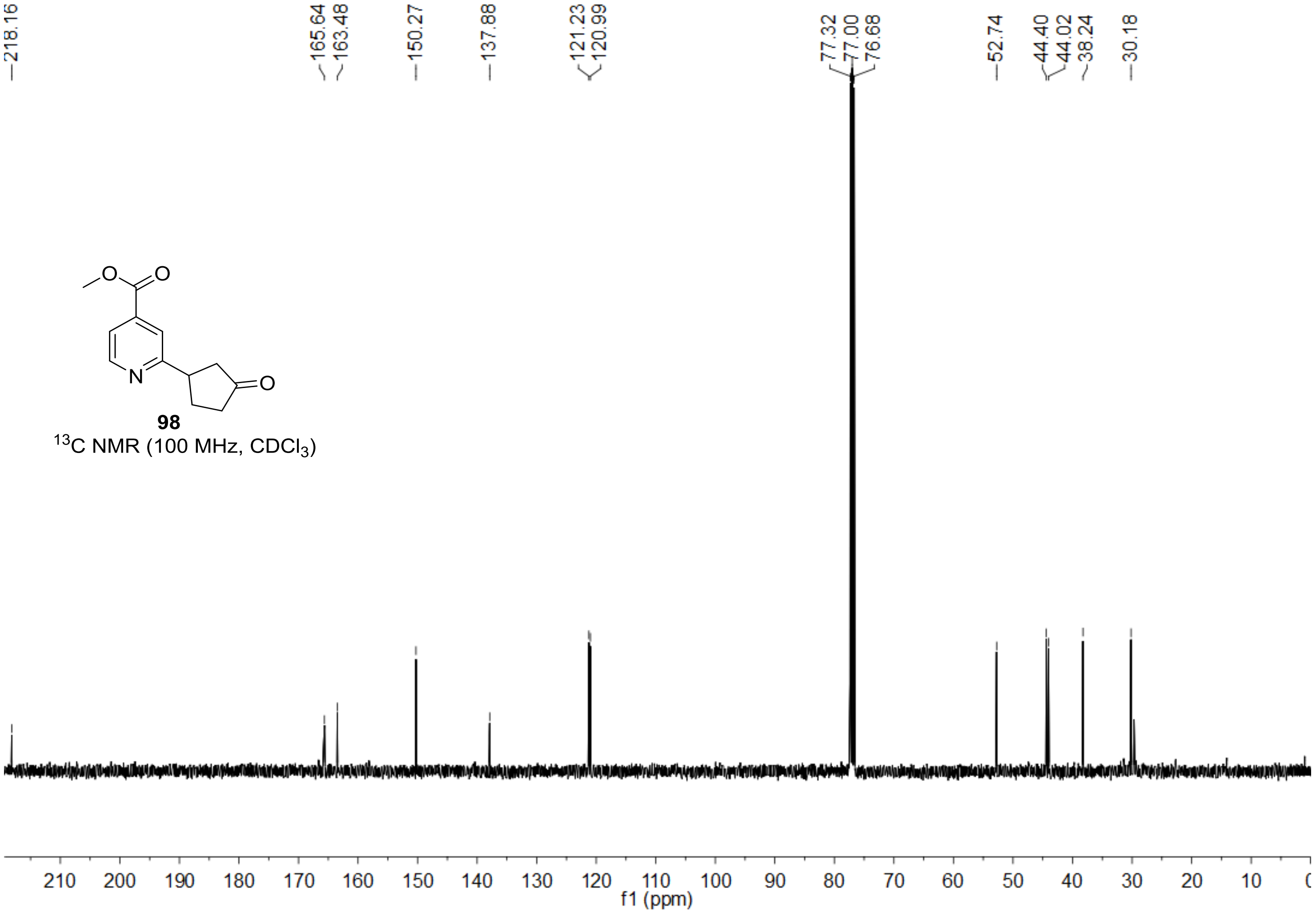
98
13C NMR (100 MHz, CDCl3)
218.16
165.64
163.48
150.27
137.88
121.23
120.99
77.32
77.00
76.68
52.74
44.40
44.02
38.24
30.18
210 200 190 180 170 160 150 140 130 120 110 100 90 80 70 60 50 40 30 20 10
f1 (ppm)

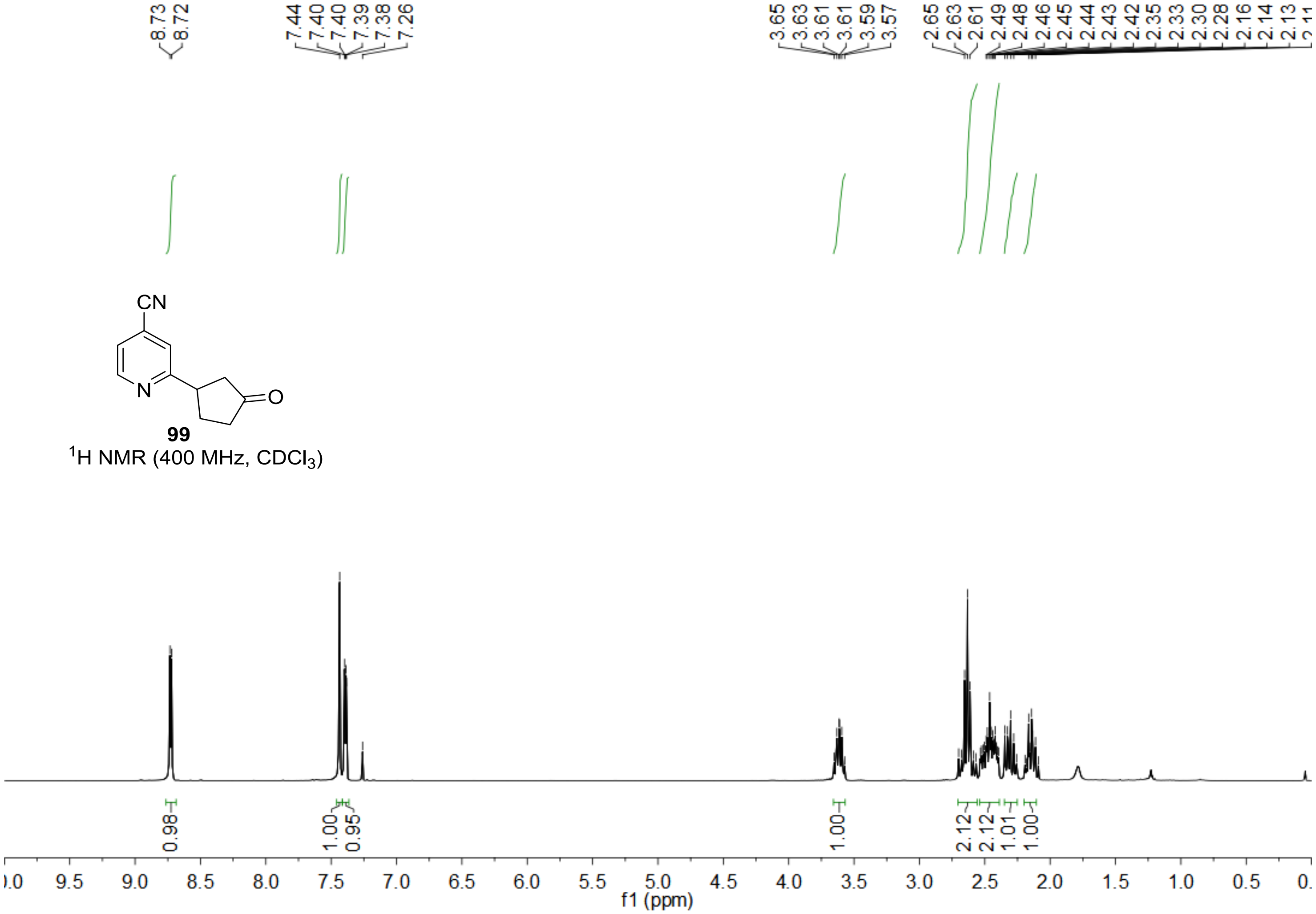
CN
N
O
99
^{1}H NMR (400 MHz, $CDCl_3$)
f1 (ppm)

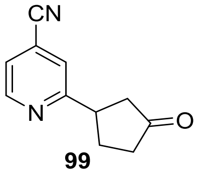

99

^{13}C NMR (100 MHz, $CDCl_3$)

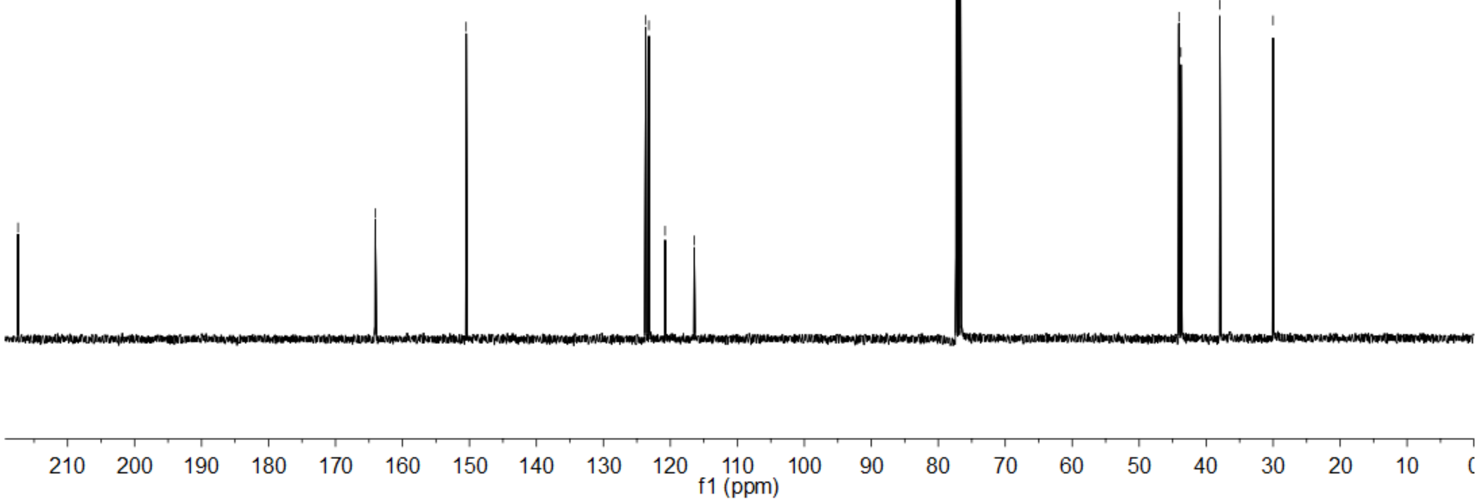

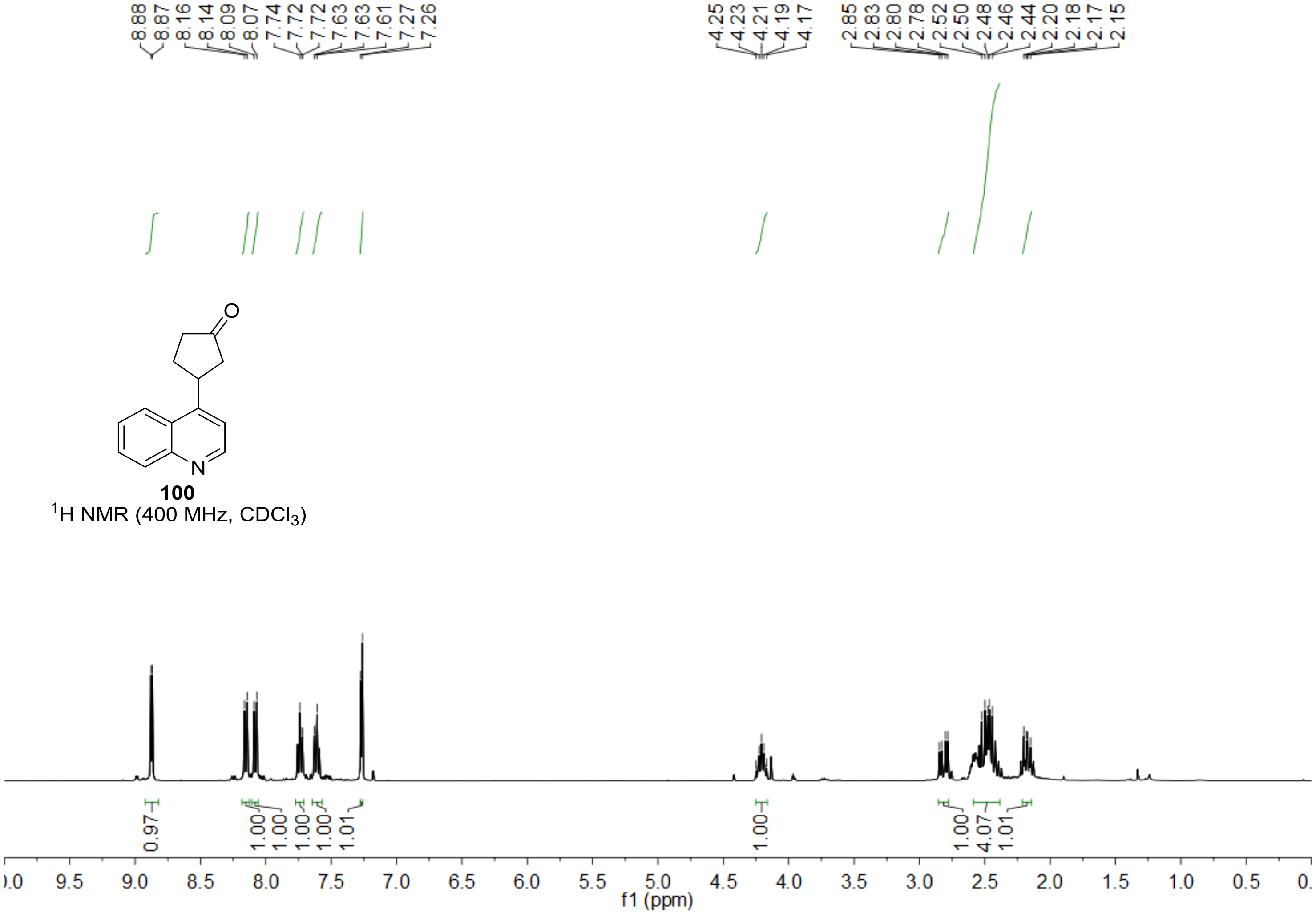

100

^{1}H NMR (400 MHz, CDCl$_3$)

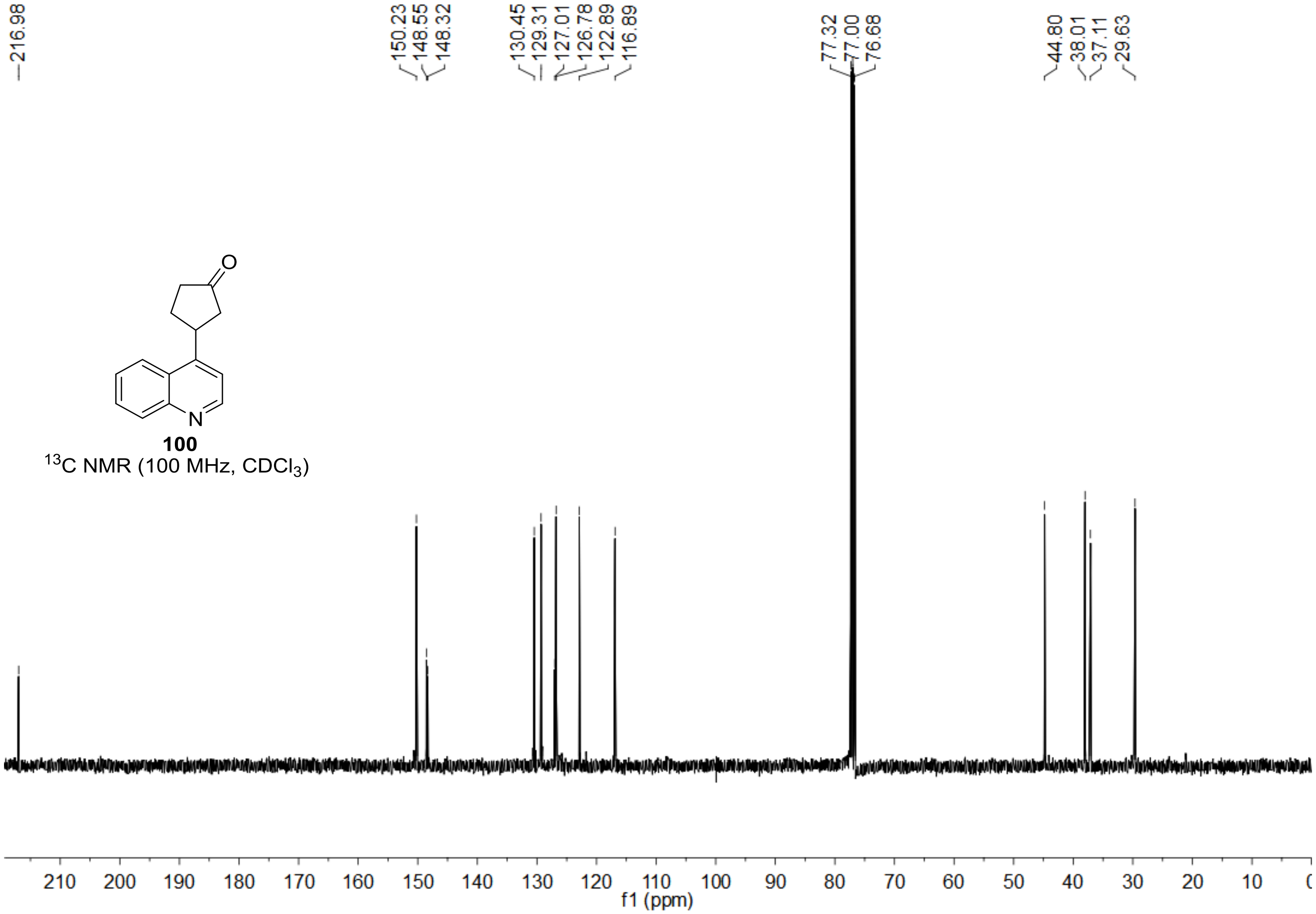
216.98
150.23
148.55
148.32
130.45
129.31
127.01
126.78
122.89
116.89
77.32
77.00
76.68
44.80
38.01
37.11
29.63
O
N
100
13C NMR (100 MHz, CDCl3)
210 200 190 180 170 160 150 140 130 120 110 100 90 80 70 60 50 40 30 20 10
f1 (ppm)

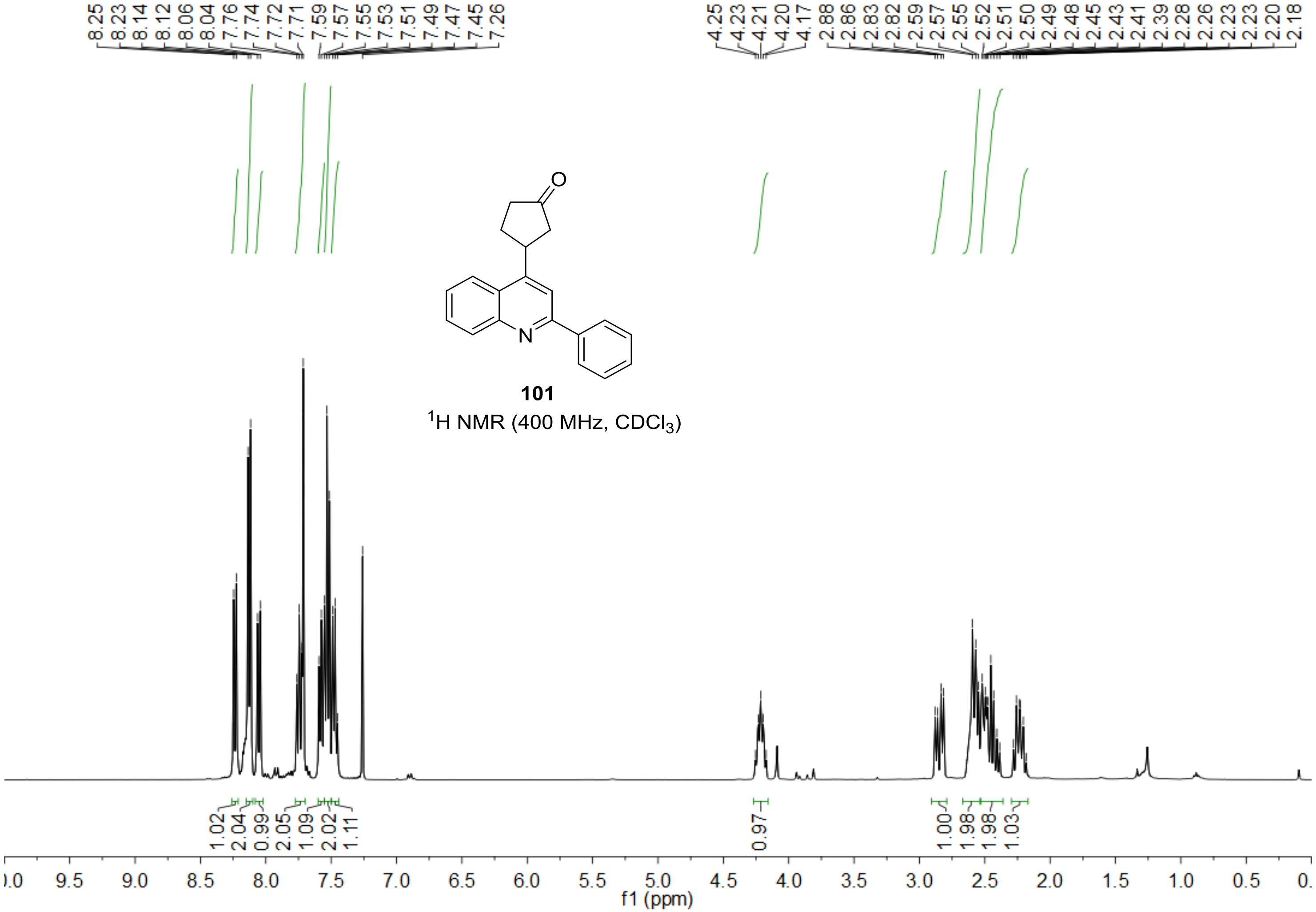
8.25 8.23 8.14 8.12 8.06 8.04 7.76 7.74 7.72 7.71 7.59 7.57 7.55 7.53 7.51 7.49 7.47 7.45 7.26
4.25 4.23 4.21 4.20 4.17 2.88 2.86 2.83 2.82 2.59 2.57 2.55 2.52 2.51 2.50 2.49 2.48 2.45 2.43 2.41 2.39 2.28 2.26 2.23 2.23 2.20 2.18
O
N
101
^{1}H NMR (400 MHz, $CDCl_3$)
1.02 2.04 0.99 2.05 1.09 2.02 1.11
0.97
1.00 1.98 1.98 1.03
9.5 9.0 8.5 8.0 7.5 7.0 6.5 6.0 5.5 5.0 4.5 4.0 3.5 3.0 2.5 2.0 1.5 1.0 0.5
f1 (ppm)

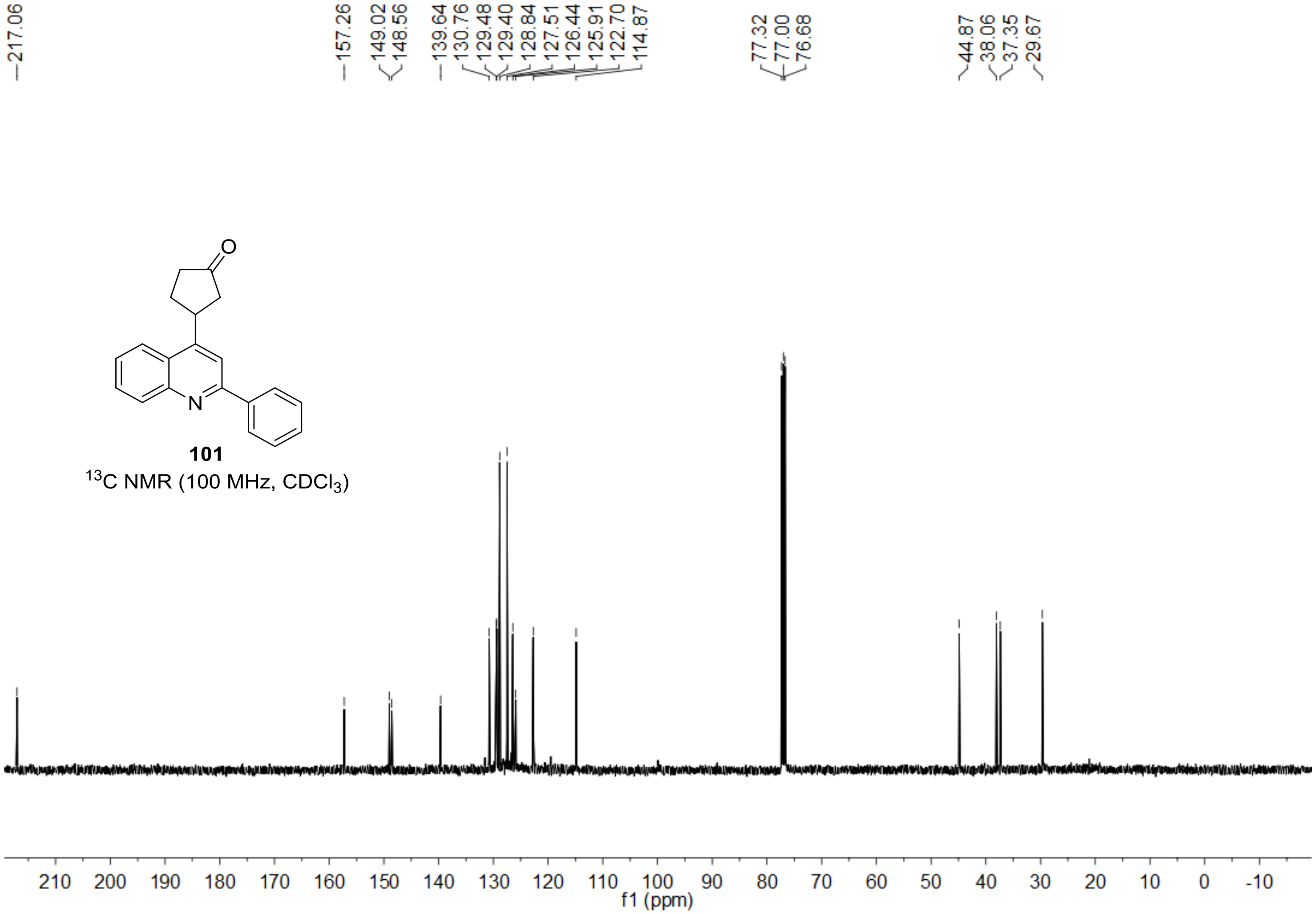

101

^{13}C NMR (100 MHz, $CDCl_3$)

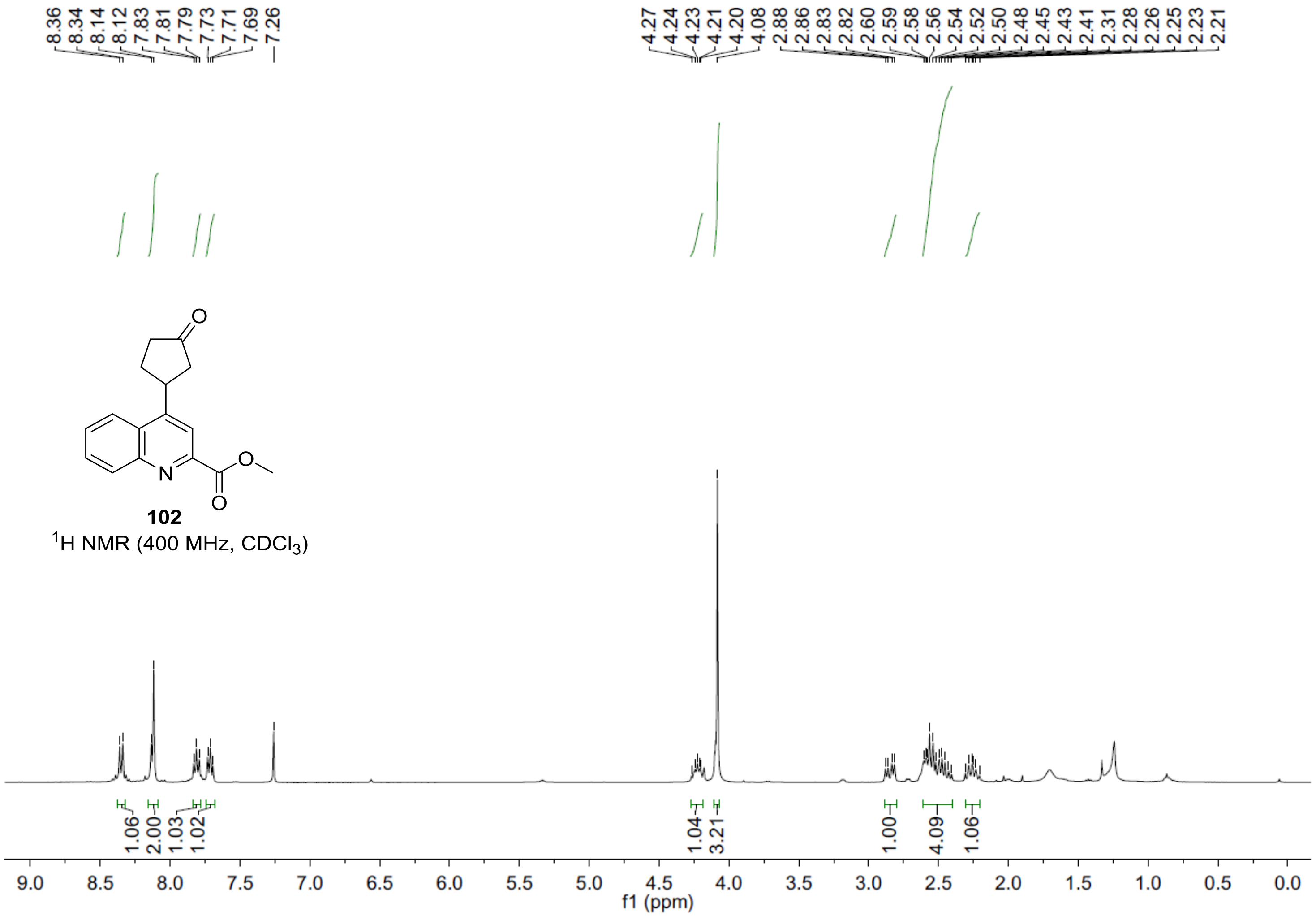

8.36
8.34
8.14
8.12
7.83
7.81
7.79
7.73
7.71
7.69
7.26
4.27
4.24
4.23
4.21
4.20
4.08
2.88
2.86
2.83
2.82
2.60
2.59
2.58
2.56
2.54
2.52
2.50
2.48
2.45
2.43
2.41
2.31
2.28
2.26
2.25
2.23
2.21
O
N
O
O
102
^{1}H NMR (400 MHz, $CDCl_3$)
1.06
2.00
1.03
1.02
1.04
3.21
1.00
4.09
1.06
9.0 8.5 8.0 7.5 7.0 6.5 6.0 5.5 5.0 4.5 4.0 3.5 3.0 2.5 2.0 1.5 1.0 0.5 0.0
f1 (ppm)

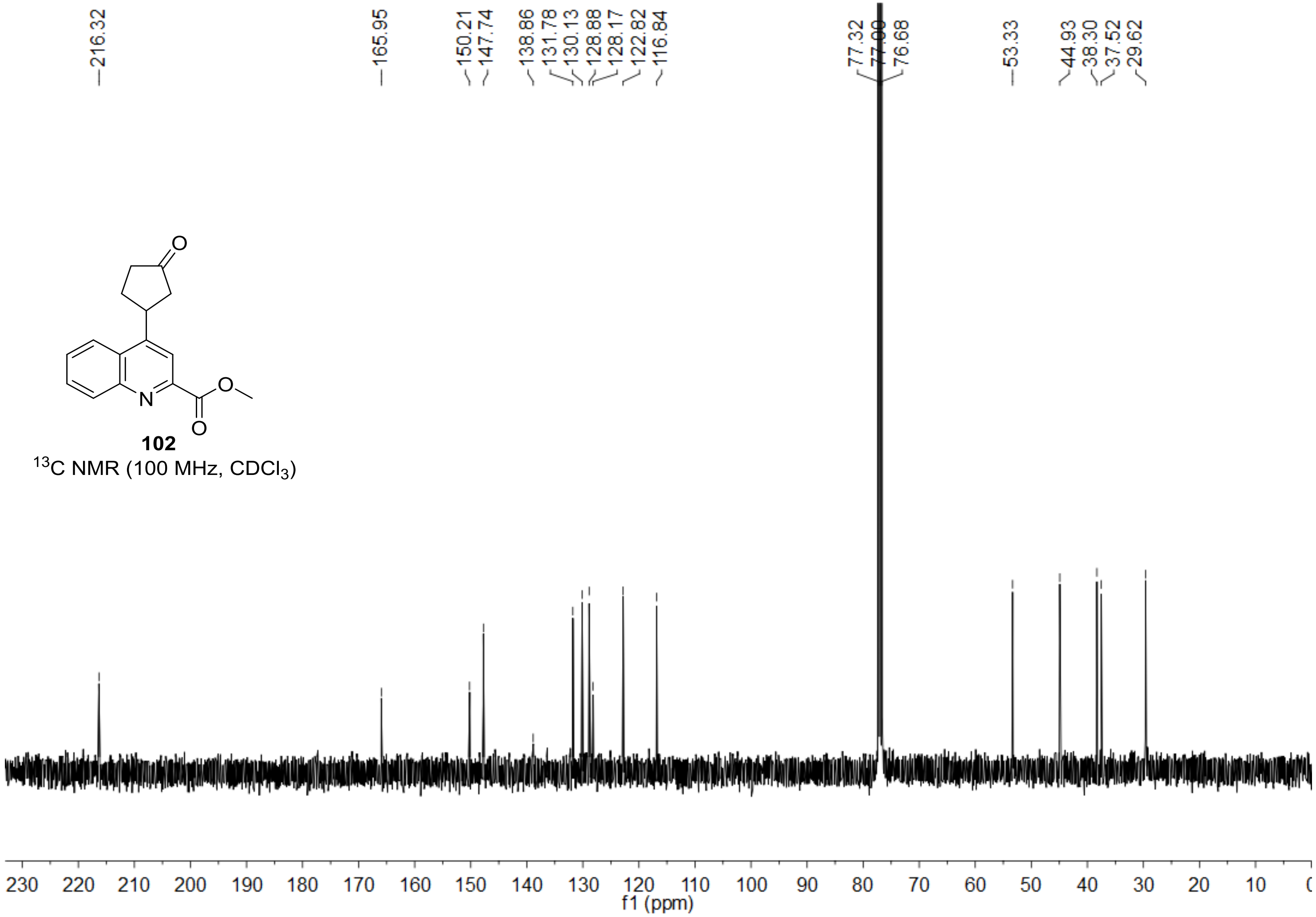

102

^{13}C NMR (100 MHz, $CDCl_3$)

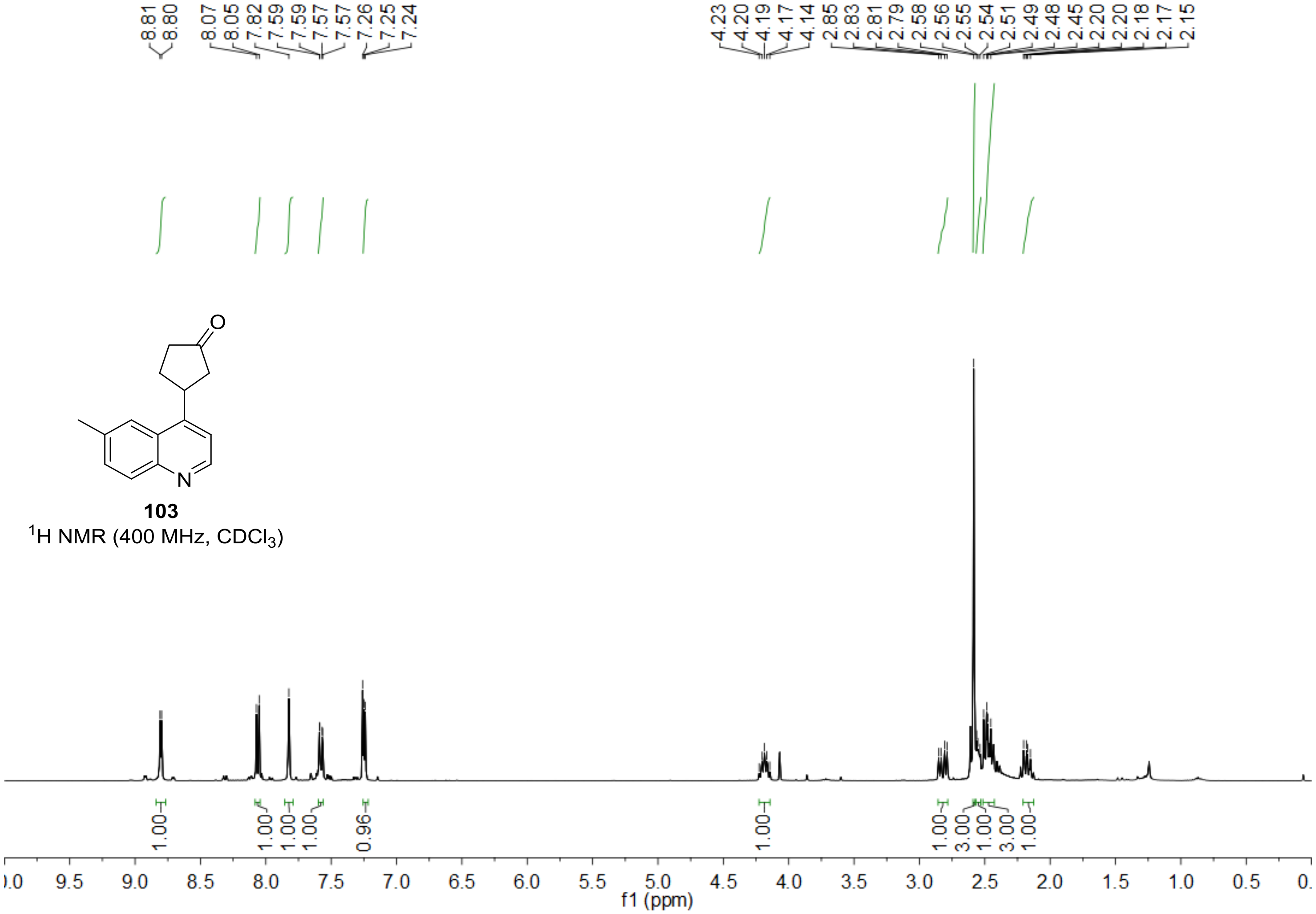

103

^{1}H NMR (400 MHz, $CDCl_3$)

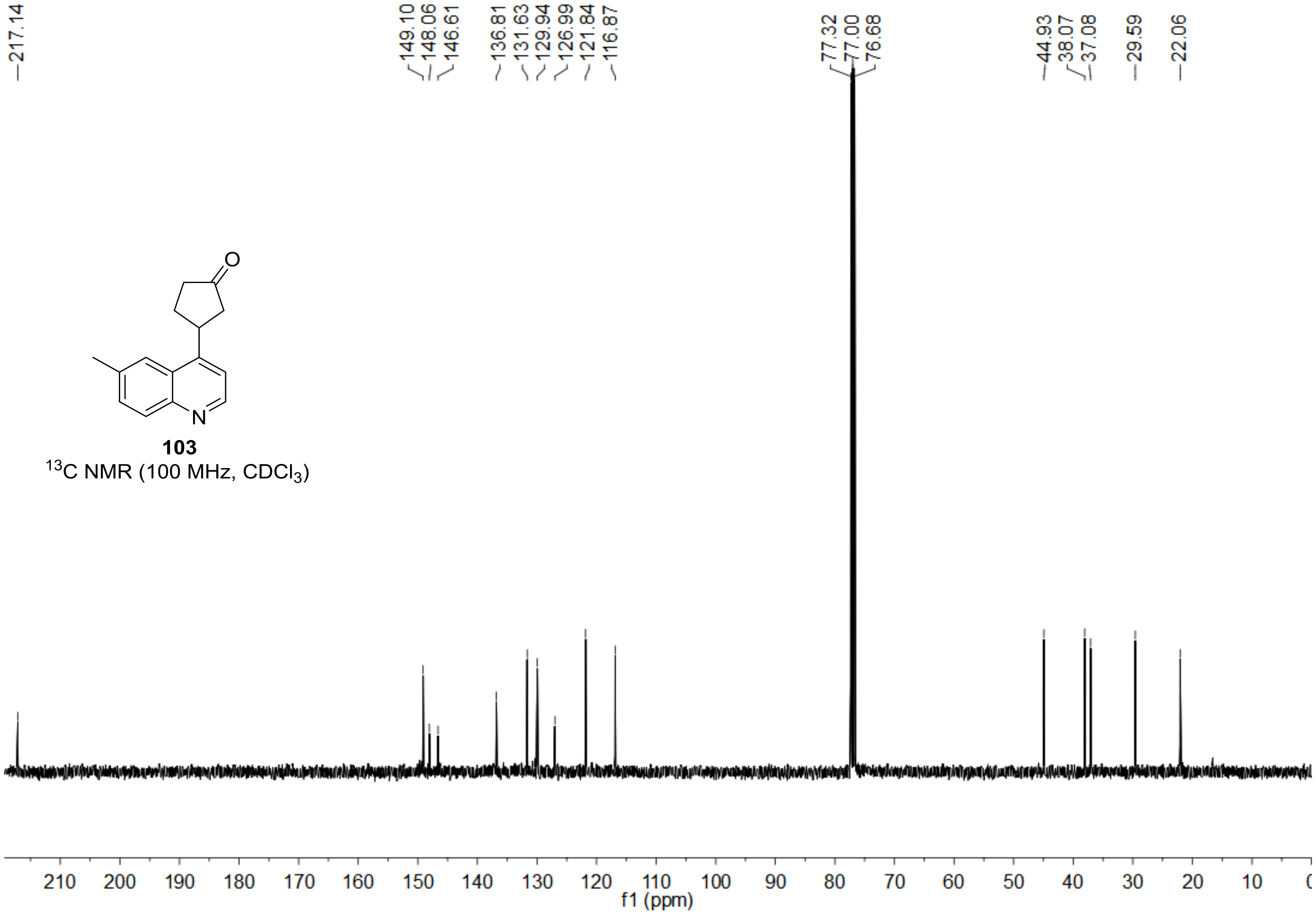

103

^{13}C NMR (100 MHz, $CDCl_3$)

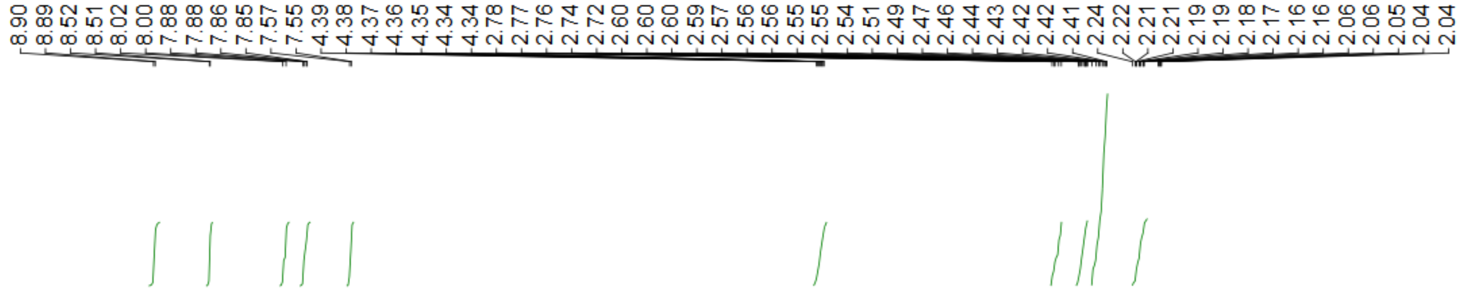

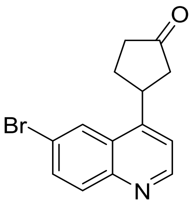

104

^{1}H NMR (400 MHz, Acetone-d_6)

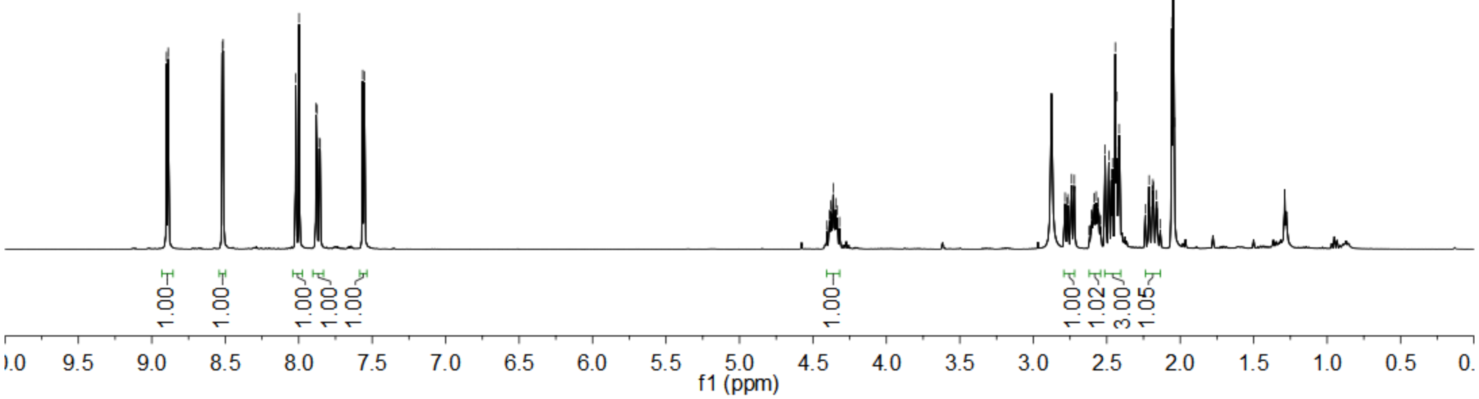

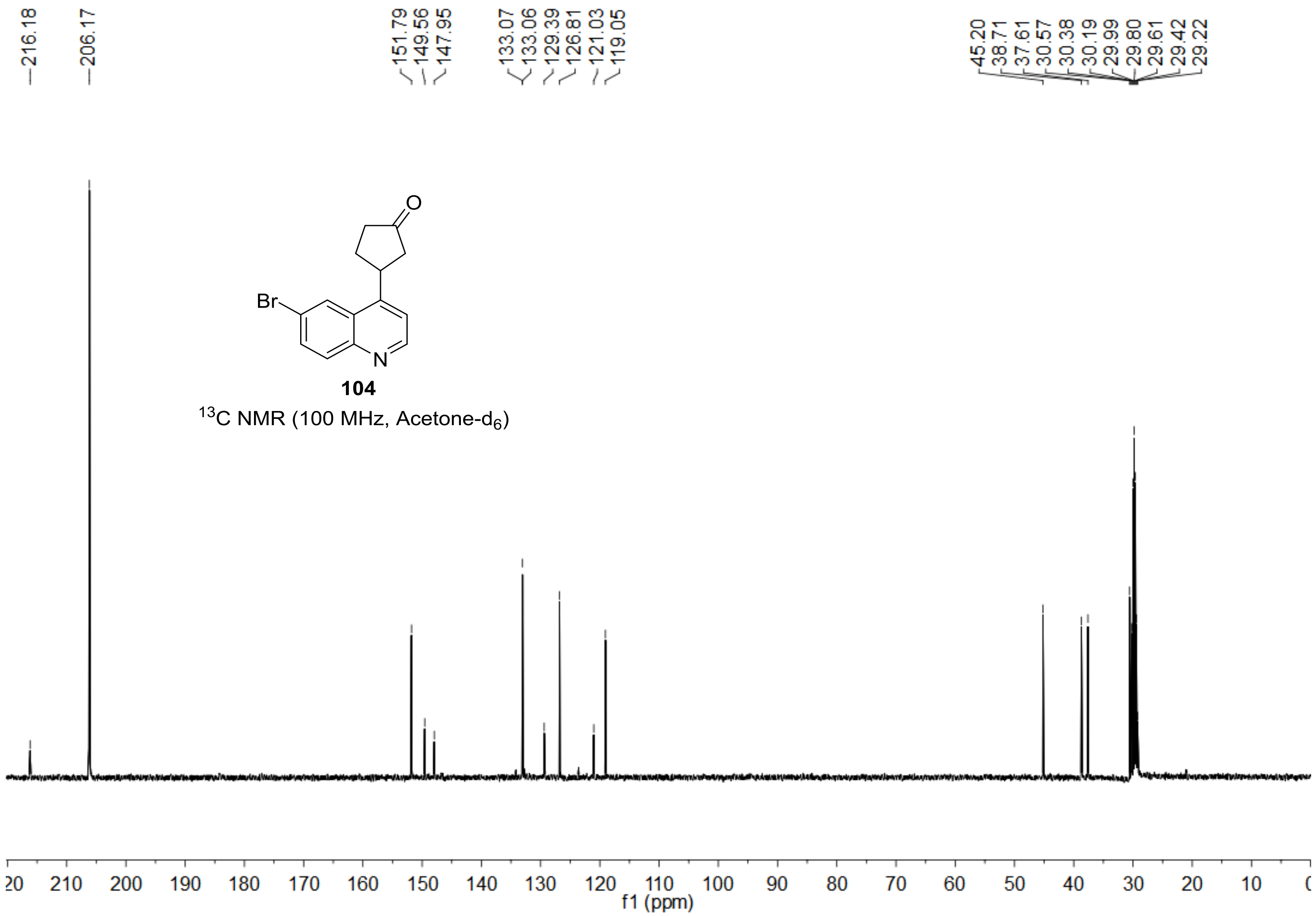

216.18
206.17
151.79
149.56
147.95
133.07
133.06
129.39
126.81
121.03
119.05
45.20
38.71
37.61
30.57
30.38
30.19
29.99
29.80
29.61
29.42
29.22
O
Br
N
104
^{13}C NMR (100 MHz, Acetone-d$_6$)
20 210 200 190 180 170 160 150 140 130 120 110 100 90 80 70 60 50 40 30 20 10
f1 (ppm)

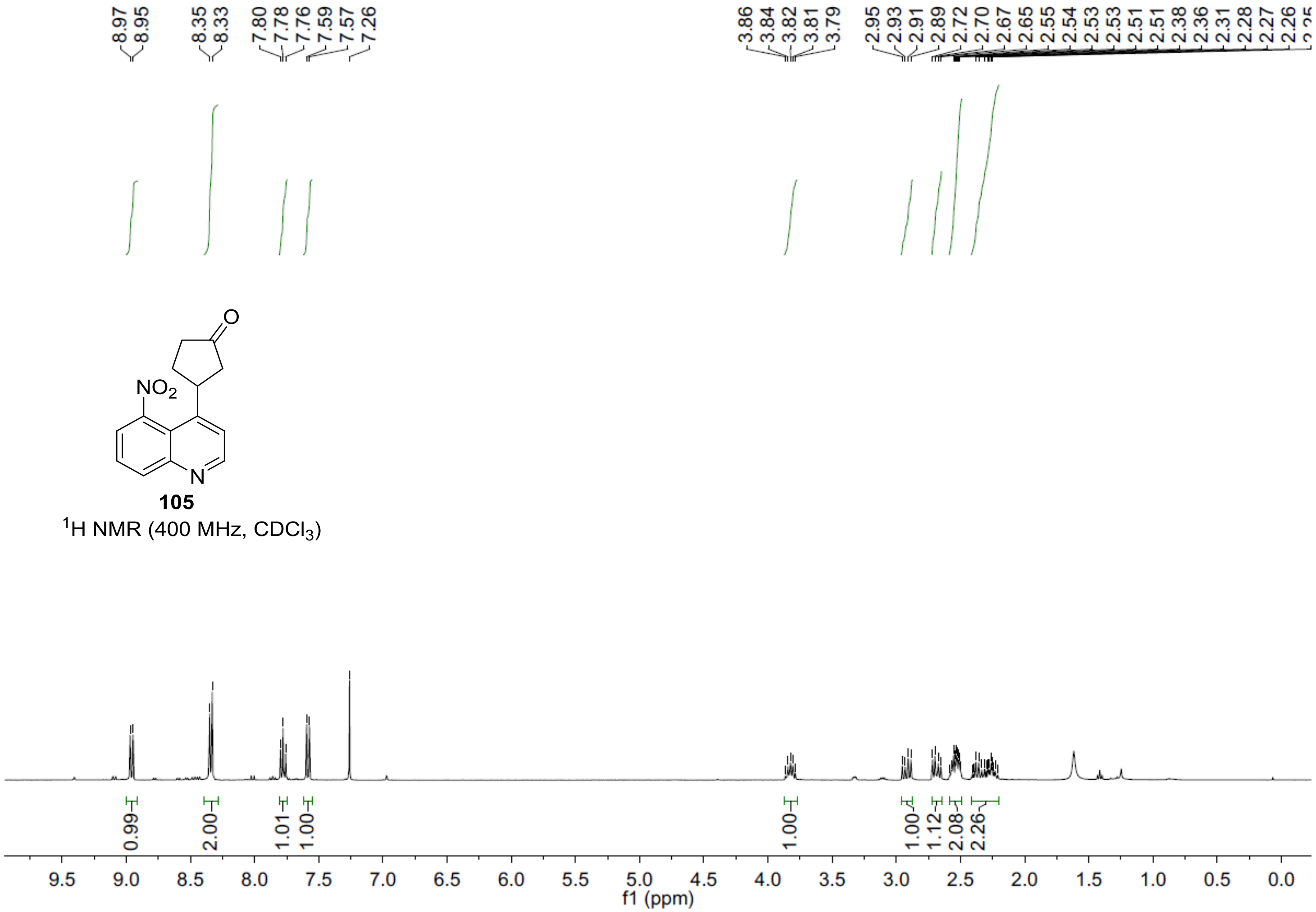

105

^{1}H NMR (400 MHz, $CDCl_3$)

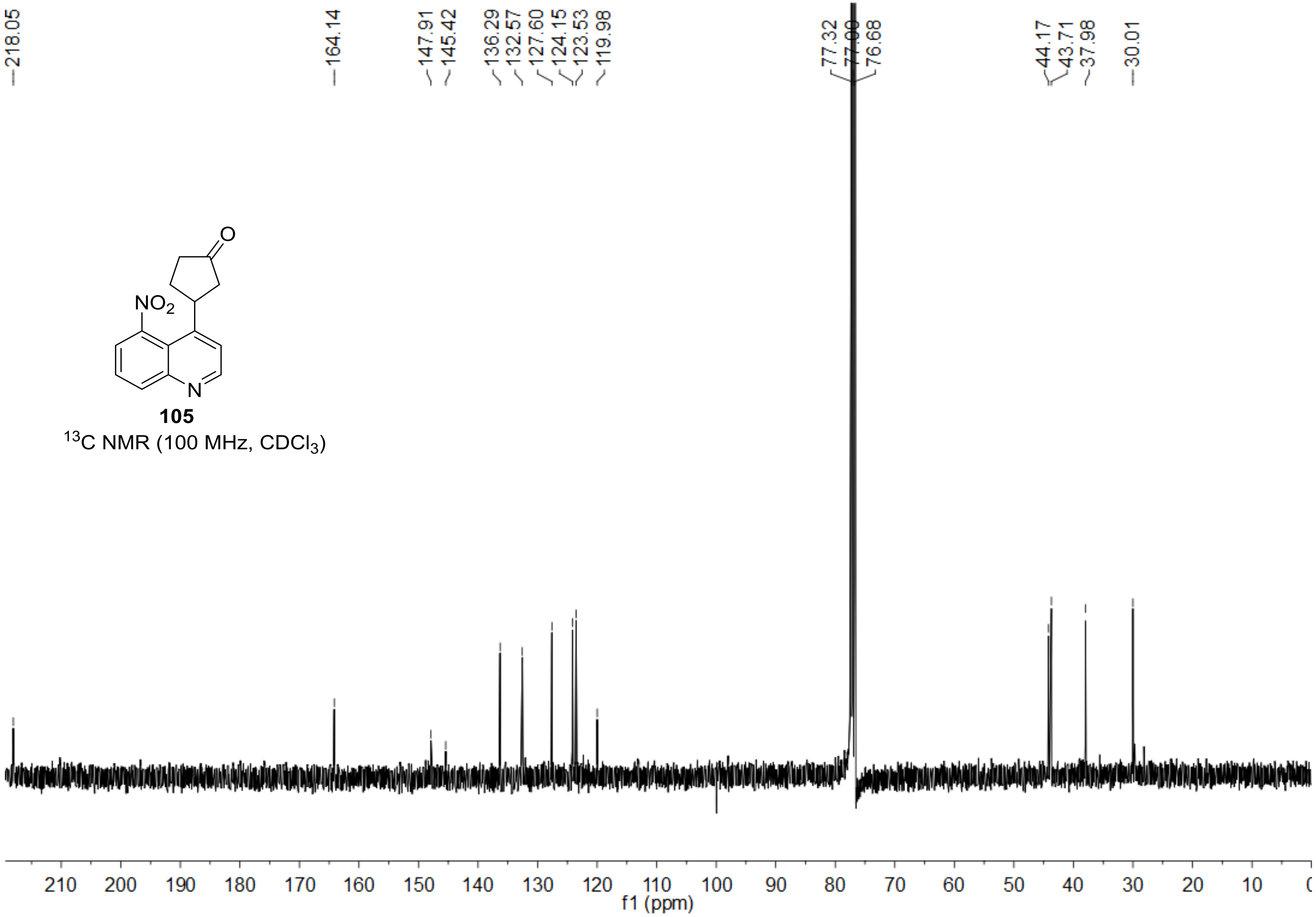

218.05
164.14
147.91
145.42
136.29
132.57
127.60
124.15
123.53
119.98
77.32
77.00
76.68
44.17
43.71
37.98
30.01
NO2
O
N
105
13C NMR (100 MHz, CDCl3)
210 200 190 180 170 160 150 140 130 120 110 100 90 80 70 60 50 40 30 20 10 0
f1 (ppm)

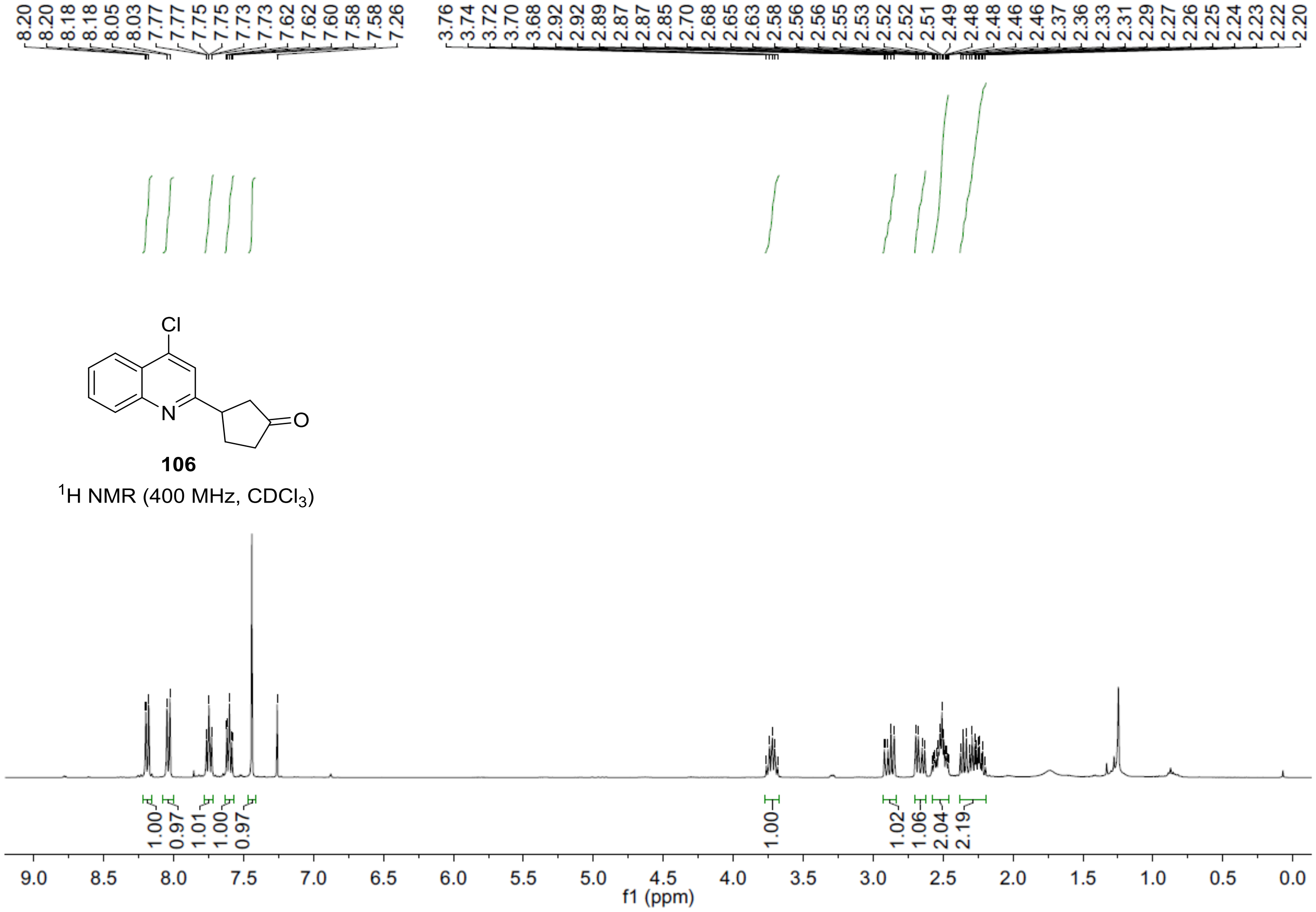

106

^{1}H NMR (400 MHz, $CDCl_3$)

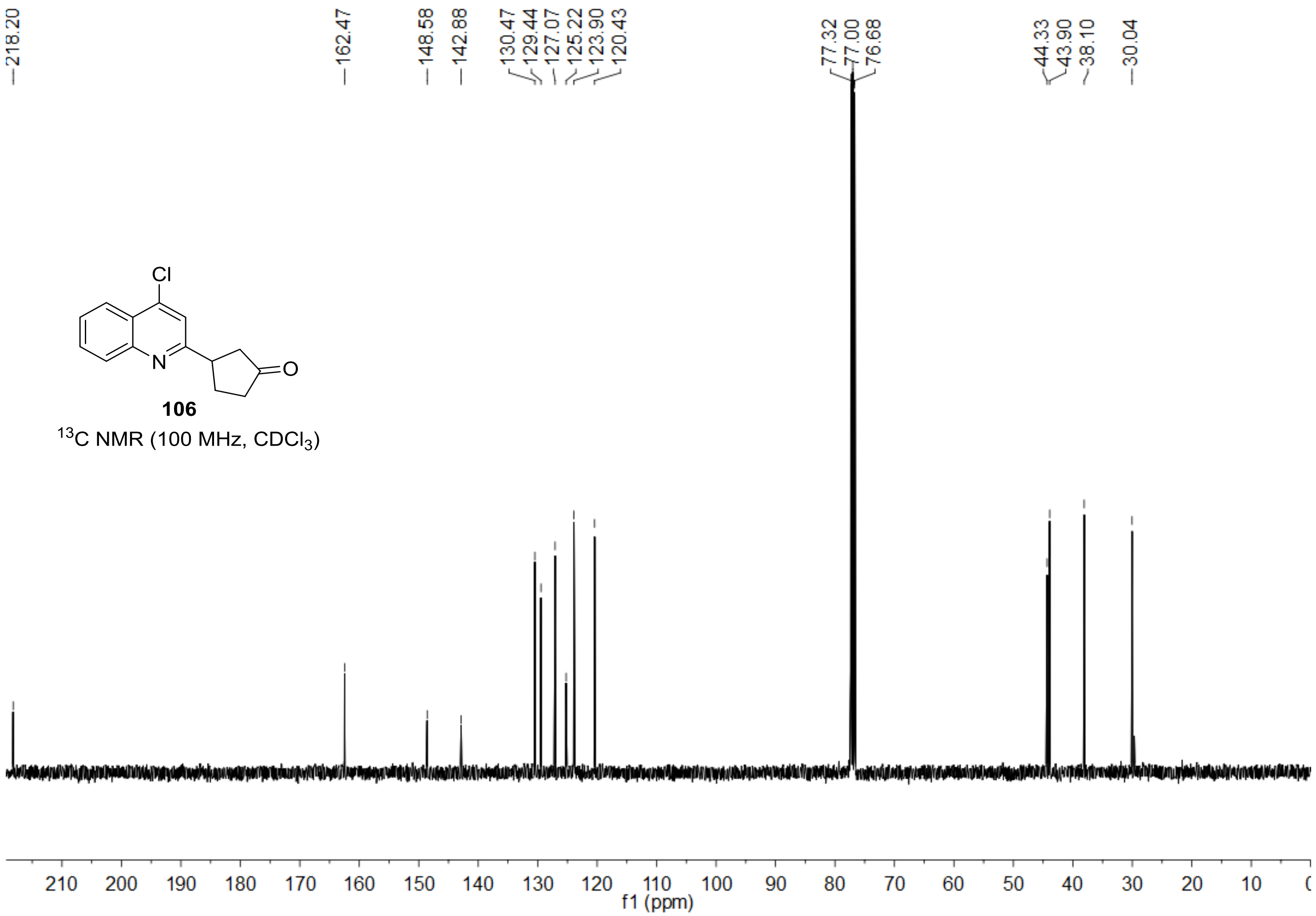

218.20
162.47
148.58
142.88
130.47
129.44
127.07
125.22
123.90
120.43
77.32
77.00
76.68
44.33
43.90
38.10
30.04
Cl
N
O
106
^{13}C NMR (100 MHz, $CDCl_3$)
f1 (ppm)

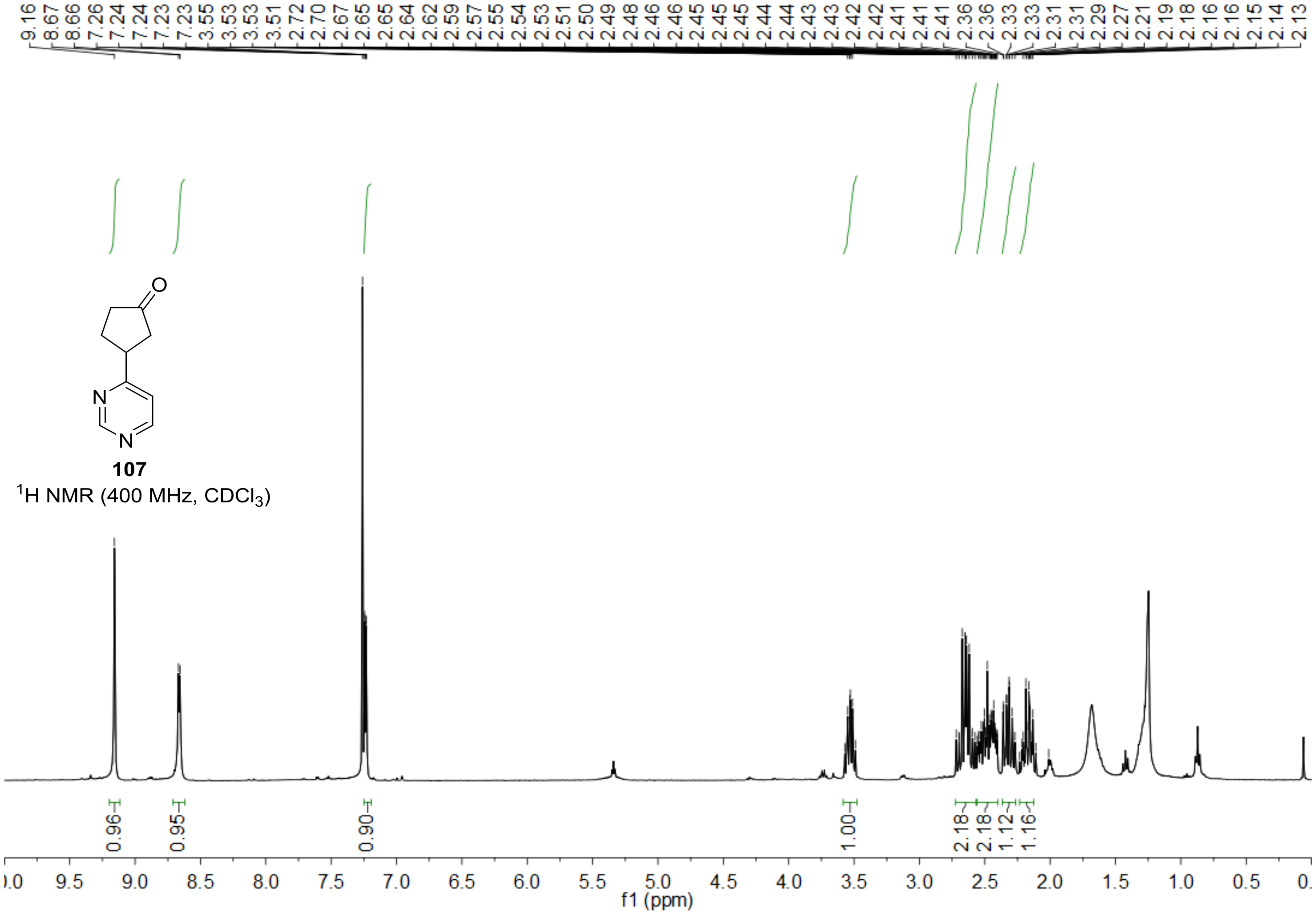
9.16 8.67 8.66 7.26 7.24 7.24 7.23 7.23 3.55 3.53 3.53 3.51 2.72 2.70 2.67 2.65 2.65 2.64 2.62 2.59 2.57 2.55 2.54 2.53 2.51 2.50 2.49 2.48 2.46 2.46 2.45 2.45 2.45 2.44 2.44 2.43 2.43 2.42 2.42 2.41 2.41 2.41 2.36 2.36 2.33 2.33 2.31 2.31 2.29 2.27 2.21 2.19 2.18 2.16 2.16 2.15 2.14 2.13
O
N
N
107
^{1}H NMR (400 MHz, $CDCl_3$)
0.96
0.95
0.90
1.00
2.18
2.18
1.12
1.16
f1 (ppm)

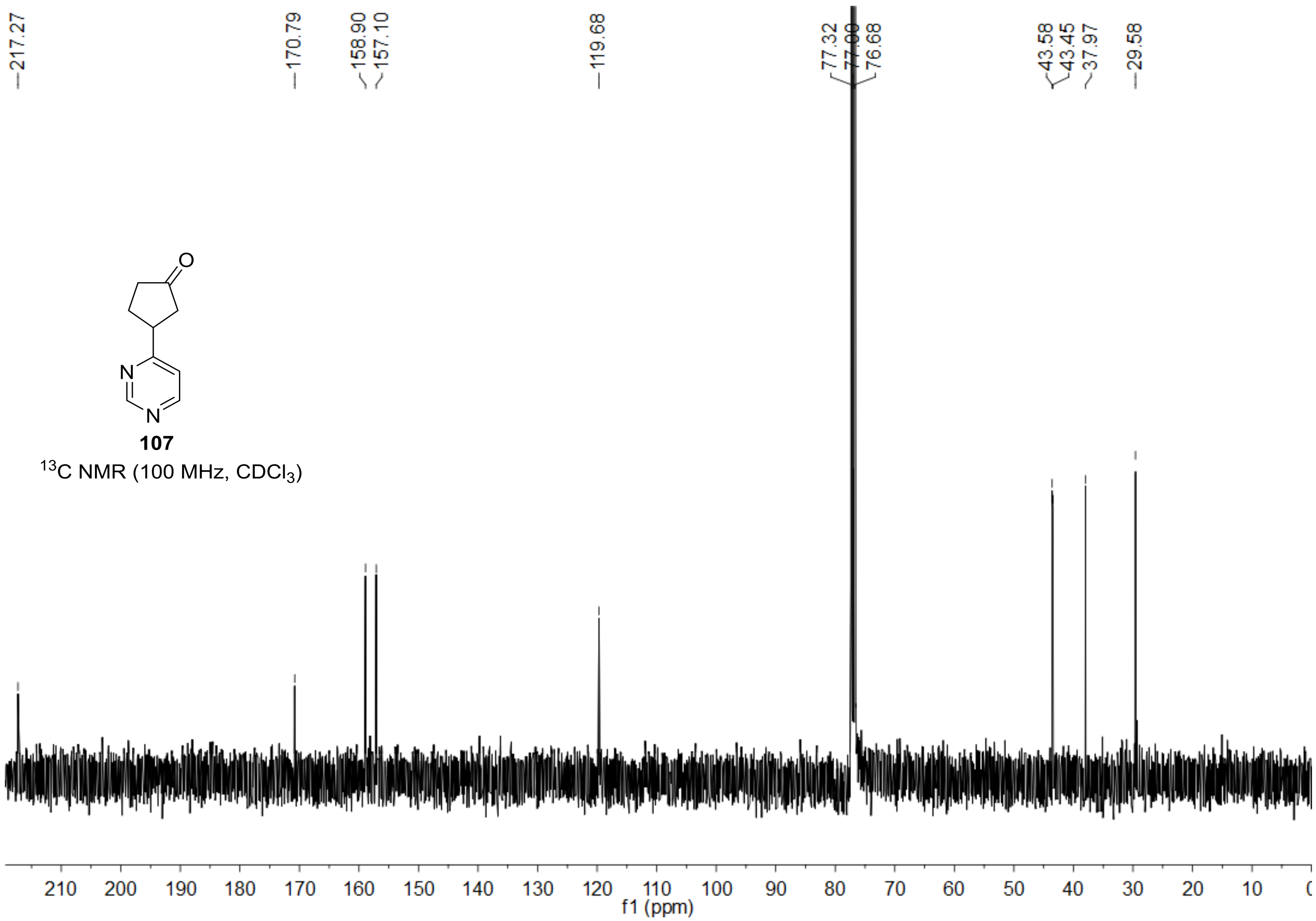

217.27
170.79
158.90
157.10
119.68
77.32
77.00
76.68
43.58
43.45
37.97
29.58
O
N
N
107
^{13}C NMR (100 MHz, $CDCl_3$)
210
200
190
180
170
160
150
140
130
120
110
100
90
80
70
60
50
40
30
20
10
f1 (ppm)

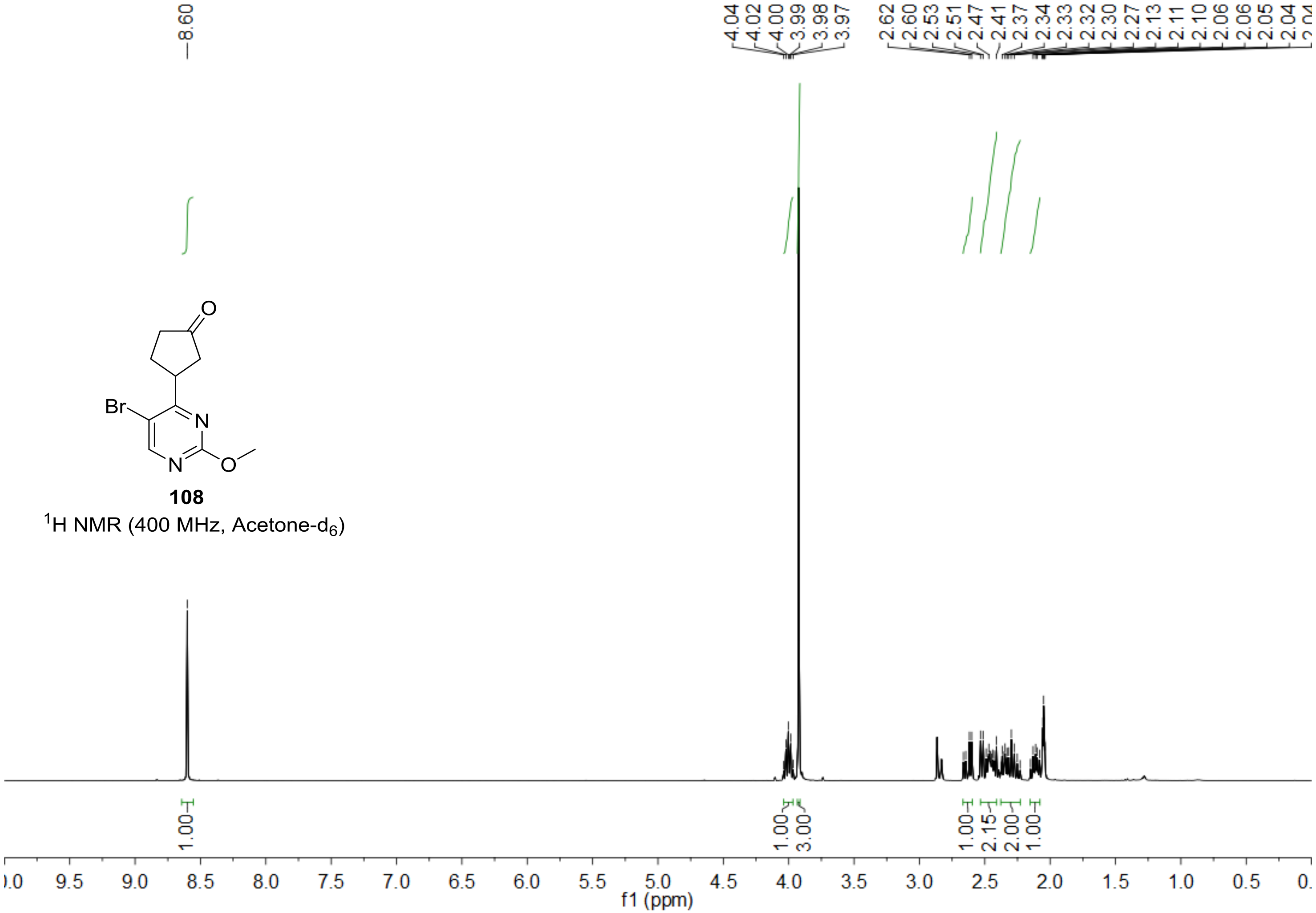

108

^{1}H NMR (400 MHz, Acetone-d_6)

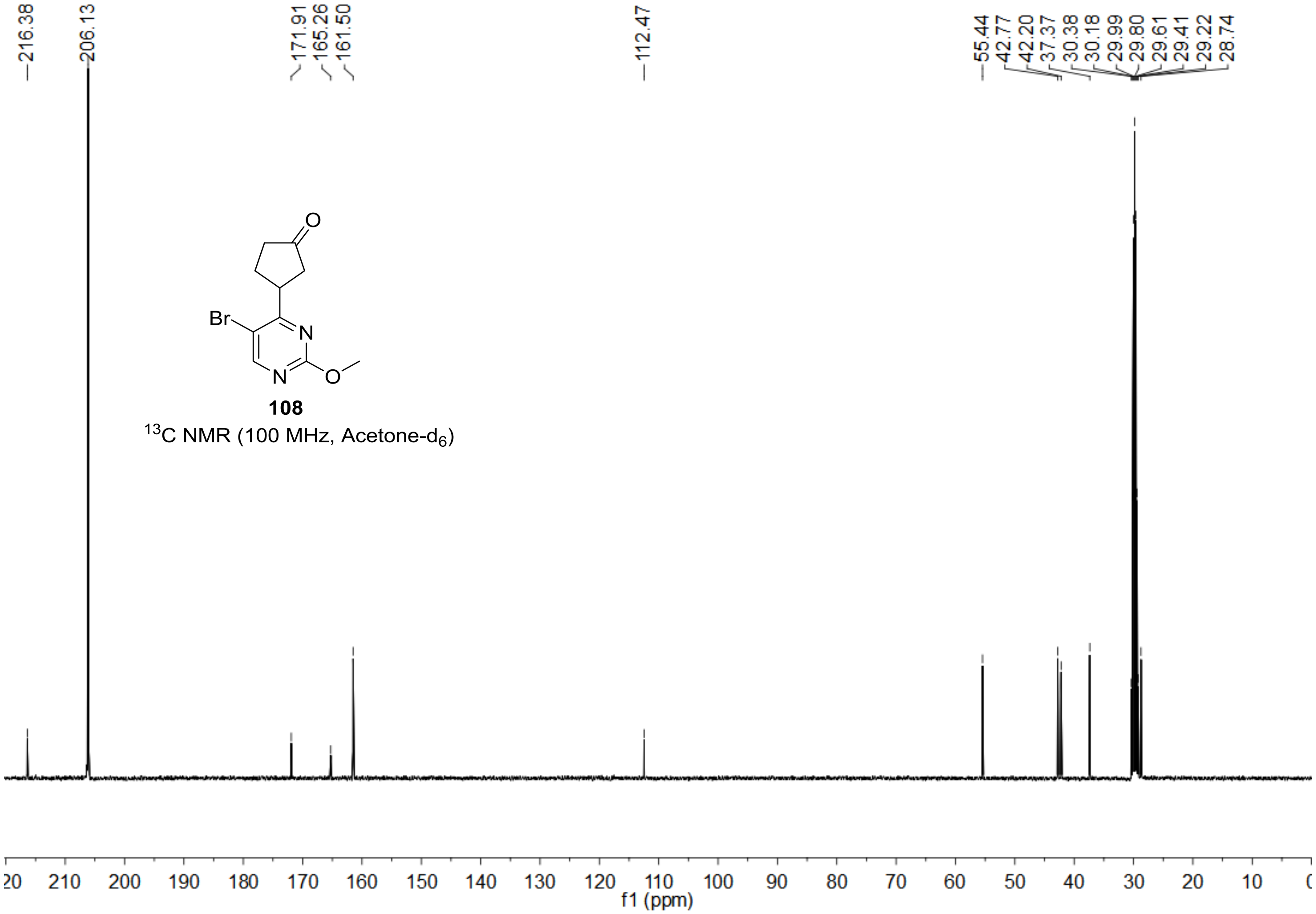
216.38
206.13
171.91
165.26
161.50
112.47
55.44
42.77
42.20
37.37
30.38
30.18
29.99
29.80
29.61
29.41
29.22
28.74
Br
N
N
O
O
108
^{13}C NMR (100 MHz, Acetone-d$_6$)
f1 (ppm)

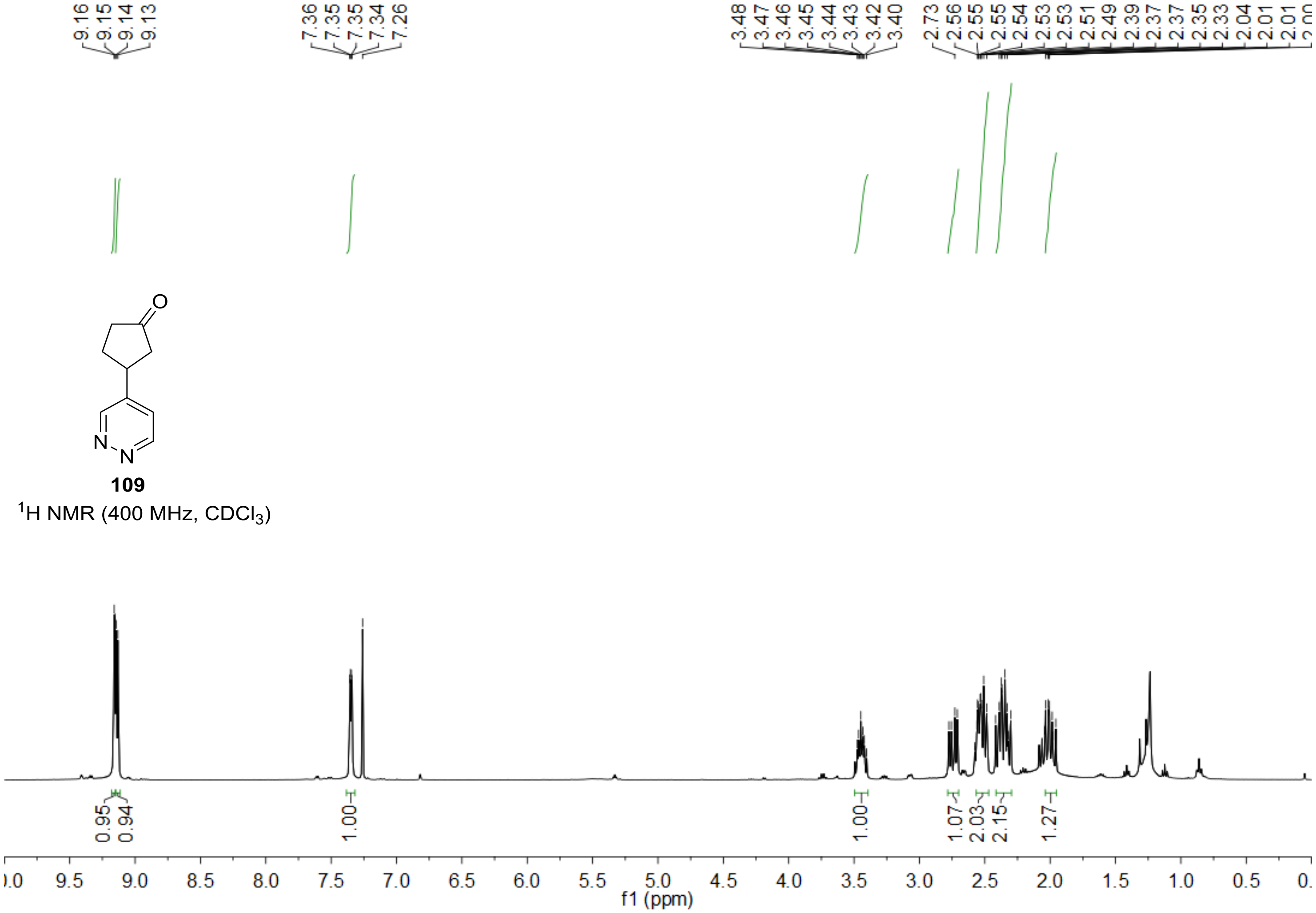

109

^{1}H NMR (400 MHz, $CDCl_3$)

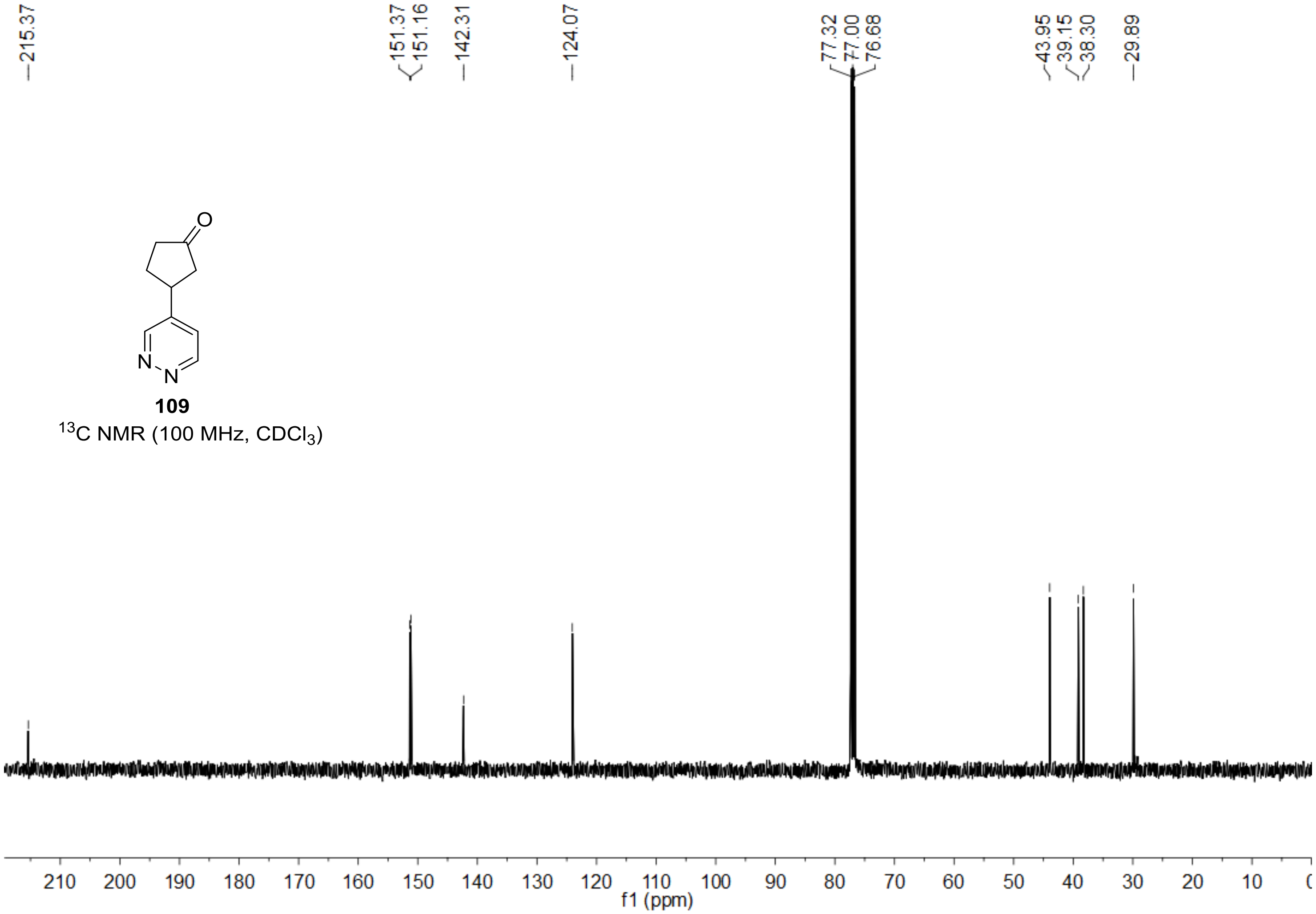

109

^{13}C NMR (100 MHz, $CDCl_3$)

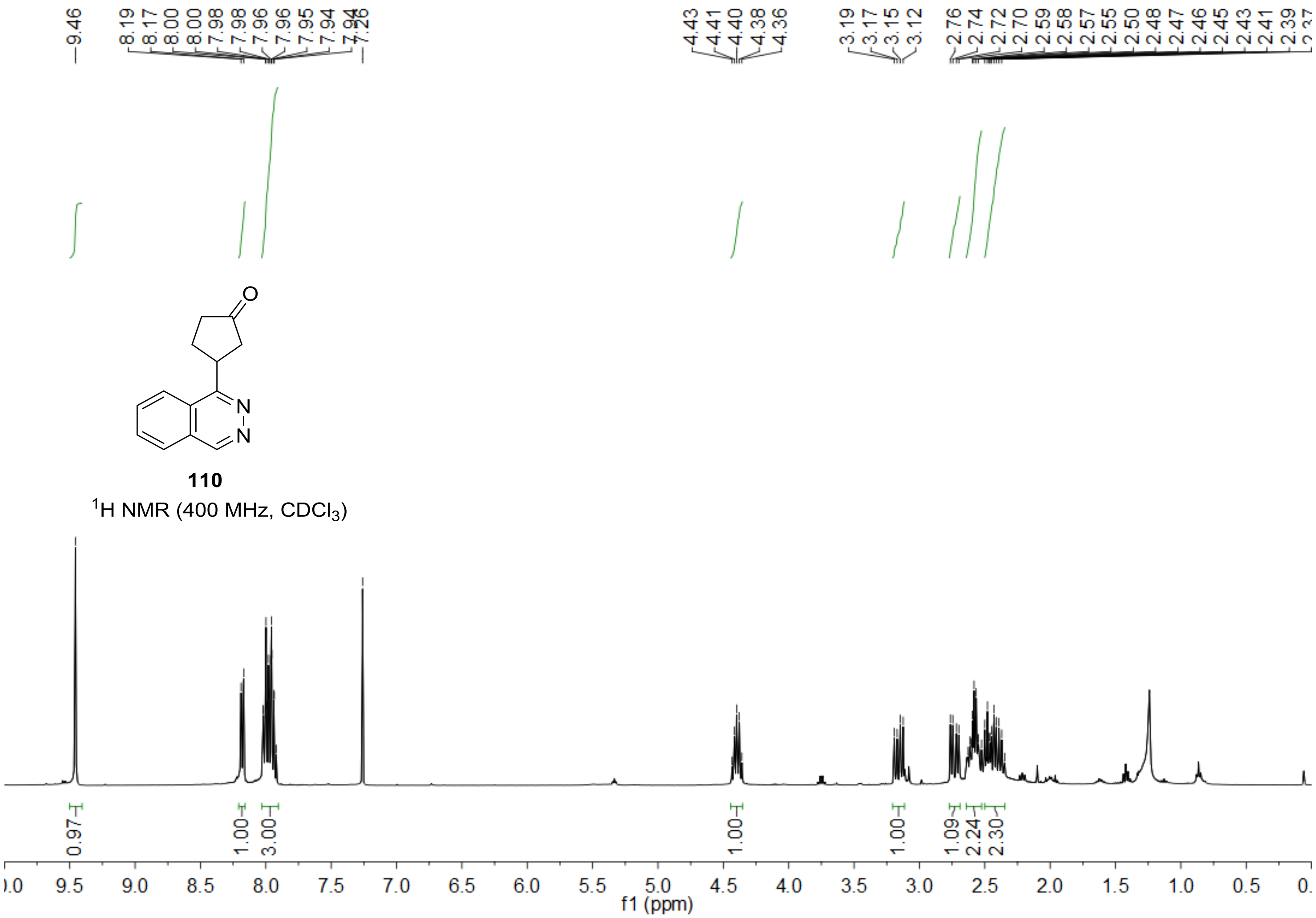

110
^{1}H NMR (400 MHz, CDCl$_3$)
9.46
8.19
8.17
8.00
8.00
7.98
7.96
7.96
7.95
7.94
4.43
4.41
4.40
4.38
4.36
3.19
3.17
3.15
3.12
2.76
2.74
2.72
2.70
2.59
2.58
2.57
2.55
2.50
2.48
2.47
2.46
2.45
2.43
2.41
2.39
0.97
1.00
3.00
1.00
1.00
1.09
2.24
2.30
f1 (ppm)

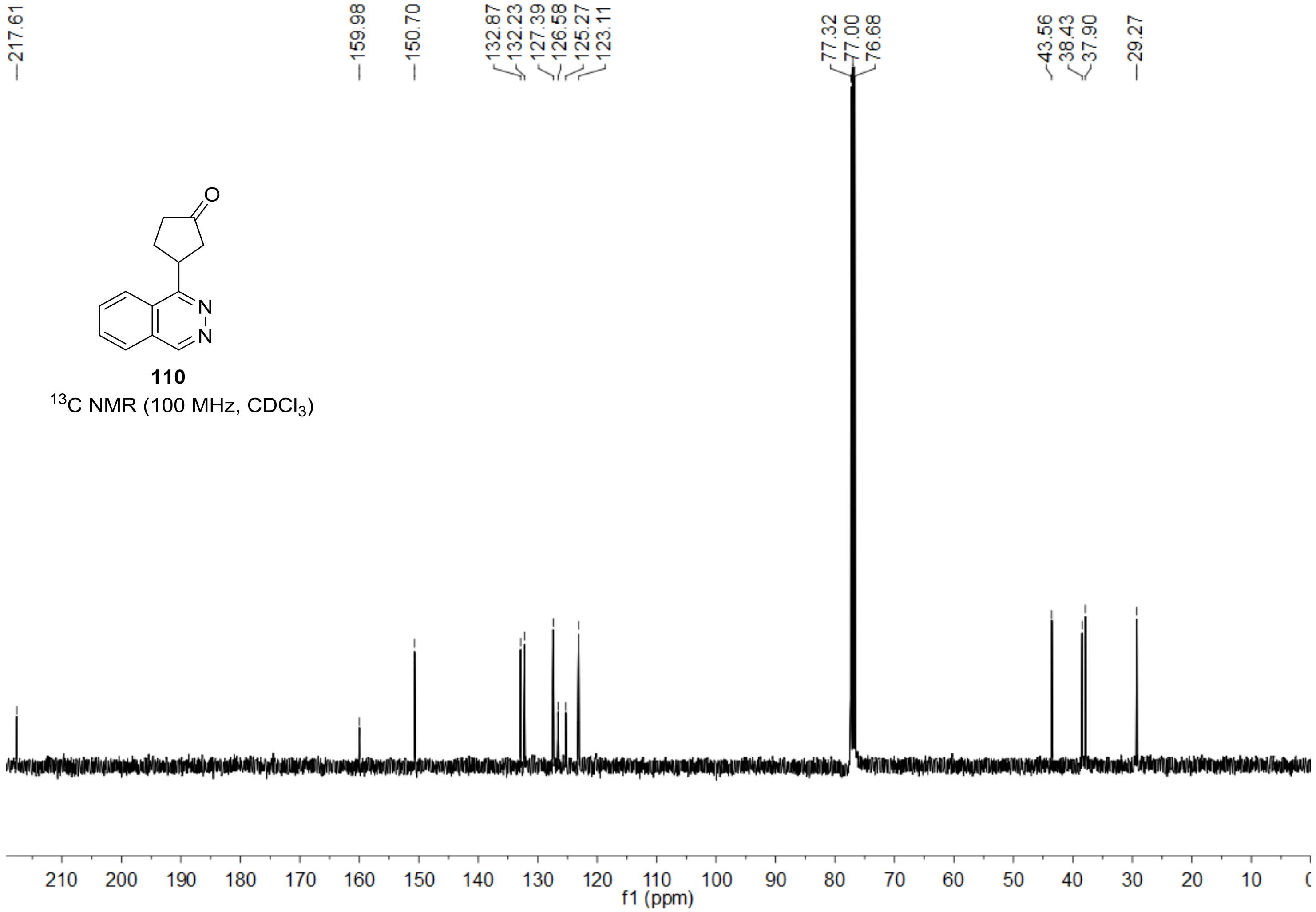

217.61
159.98
150.70
132.87
132.23
127.39
126.58
125.27
123.11
77.32
77.00
76.68
43.56
38.43
37.90
29.27
O
N
N
110
^{13}C NMR (100 MHz, $CDCl_3$)
210 200 190 180 170 160 150 140 130 120 110 100 90 80 70 60 50 40 30 20 10 0
f1 (ppm)

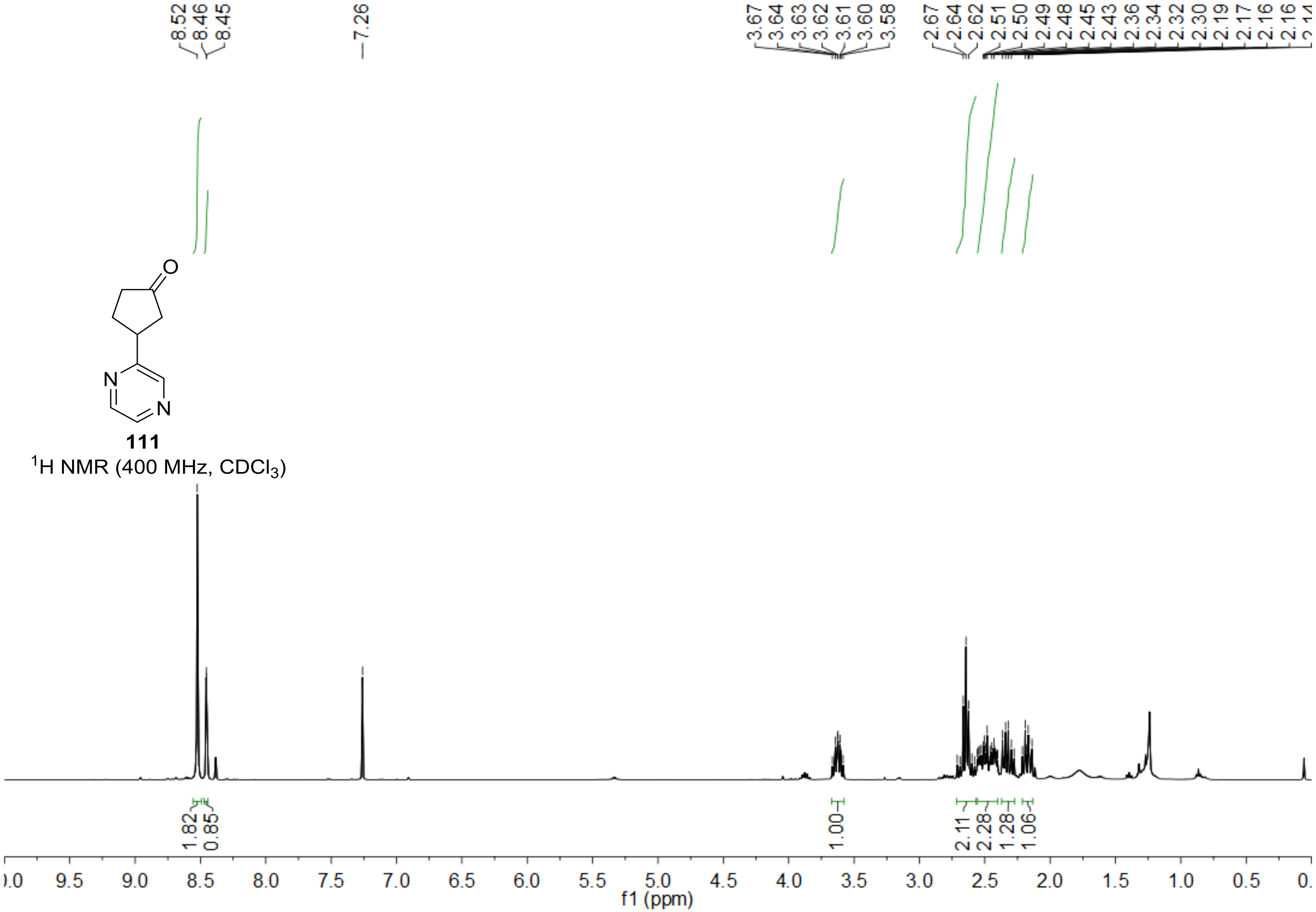

111

^{1}H NMR (400 MHz, $CDCl_3$)

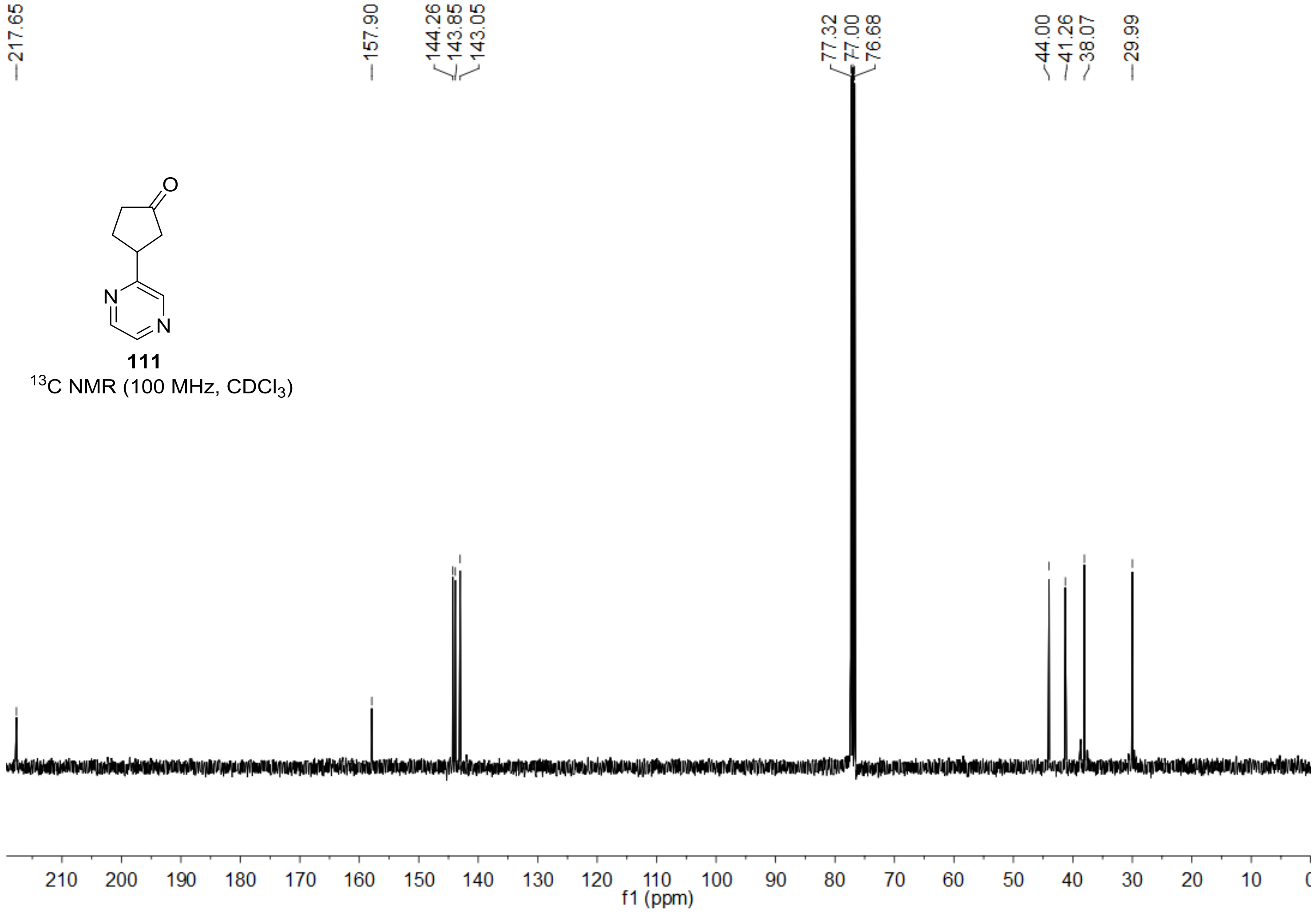

111

^{13}C NMR (100 MHz, $CDCl_3$)

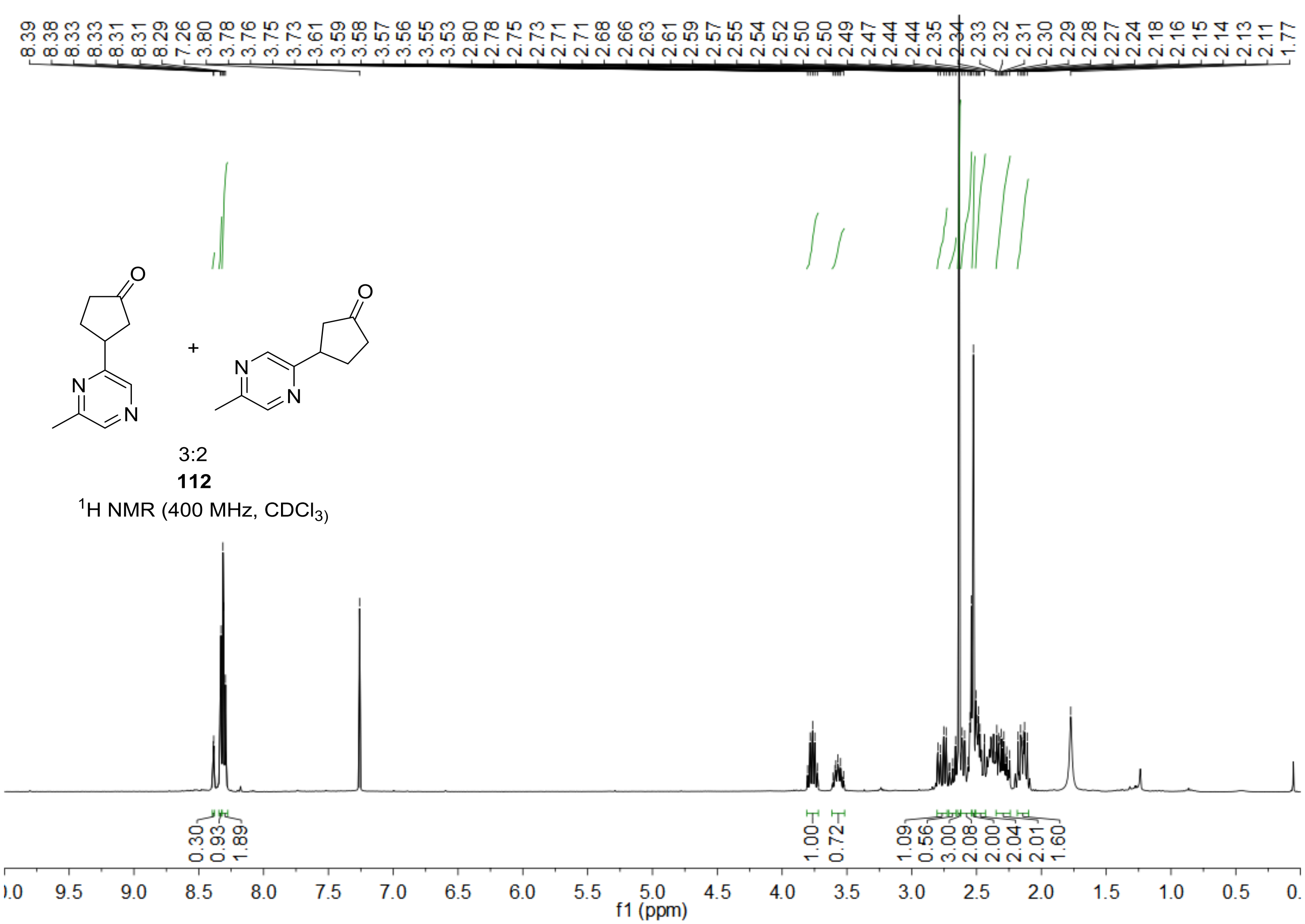

3:2

112

^{1}H NMR (400 MHz, $CDCl_3$)

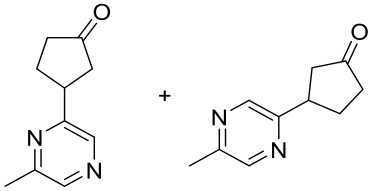

3:2
112
^{13}C NMR (100 MHz, CDC_{l3})

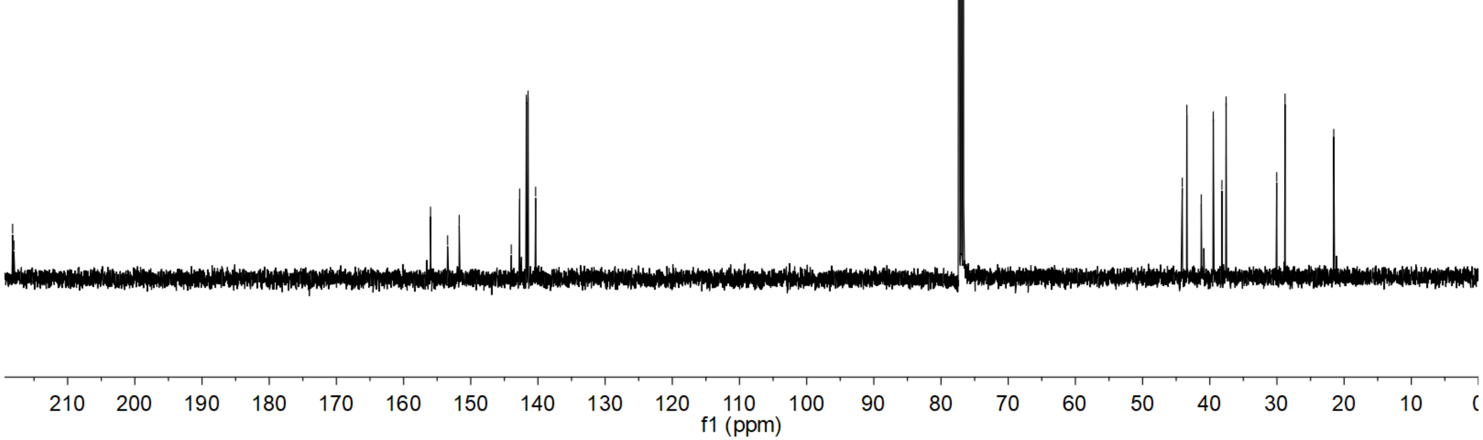

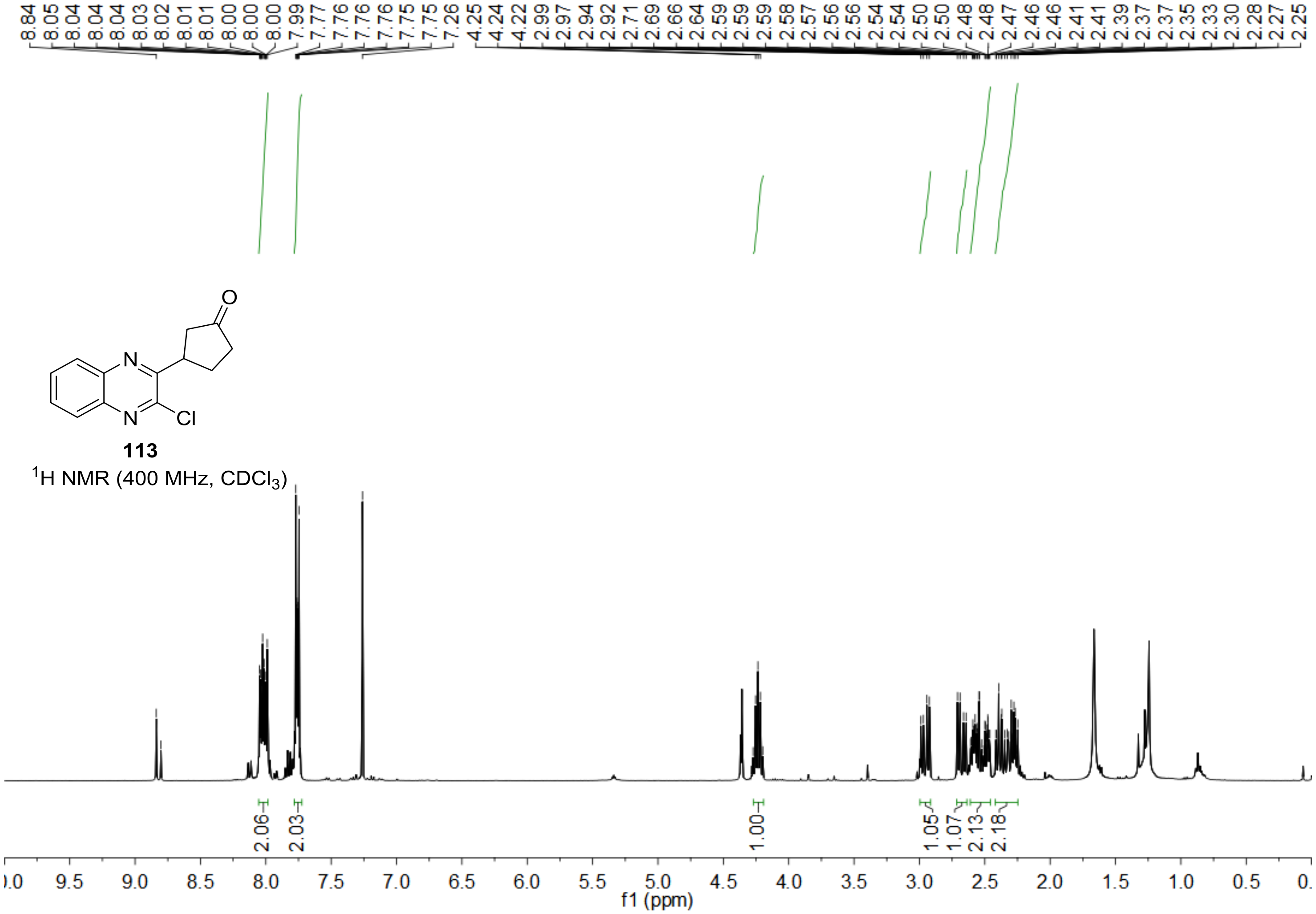

O
N
N
Cl
113
^{1}H NMR (400 MHz, CDCl$_3$)
2.06
2.03
1.00
1.05
1.07
2.13
2.18
f1 (ppm)

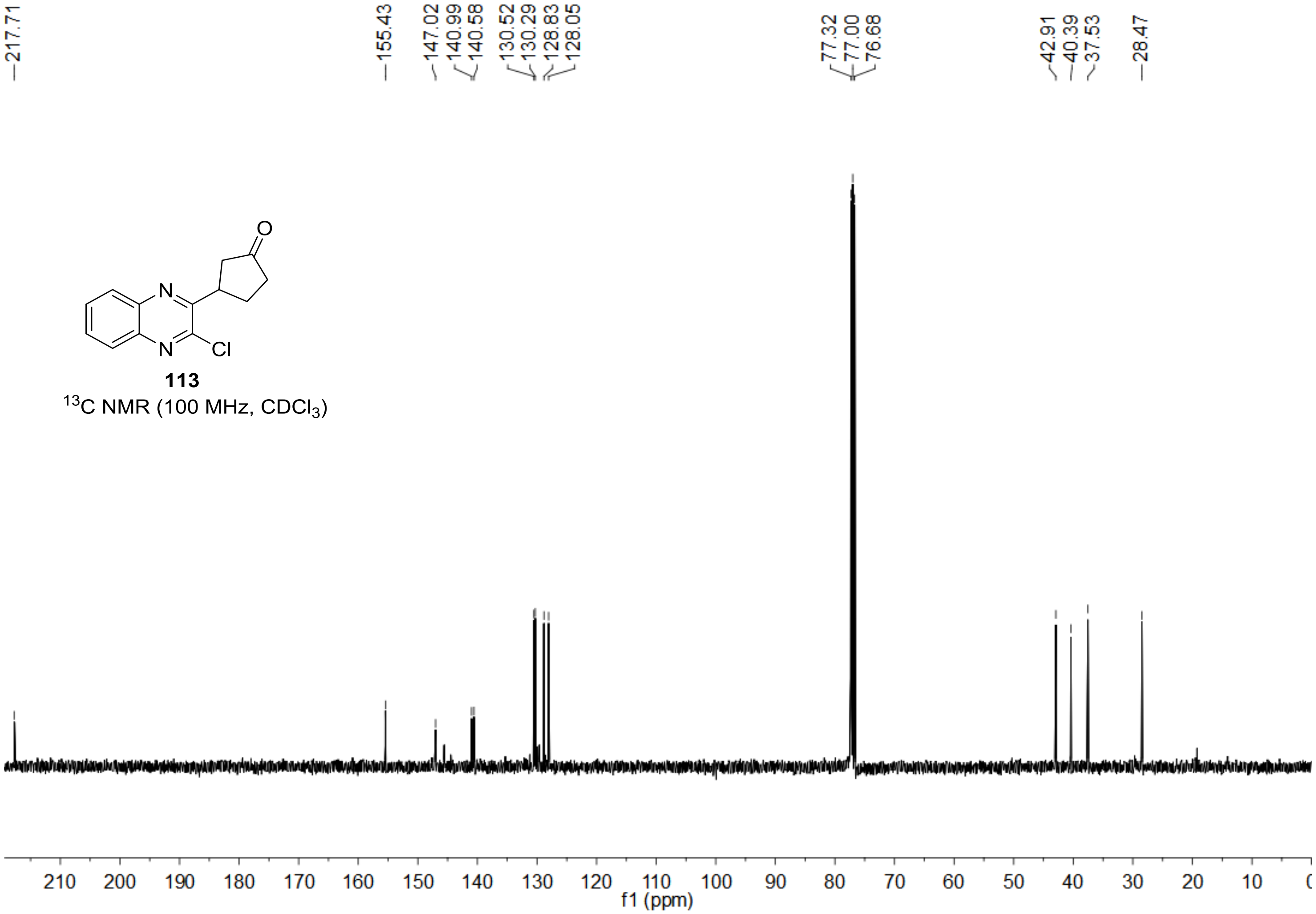

113

^{13}C NMR (100 MHz, $CDCl_3$)

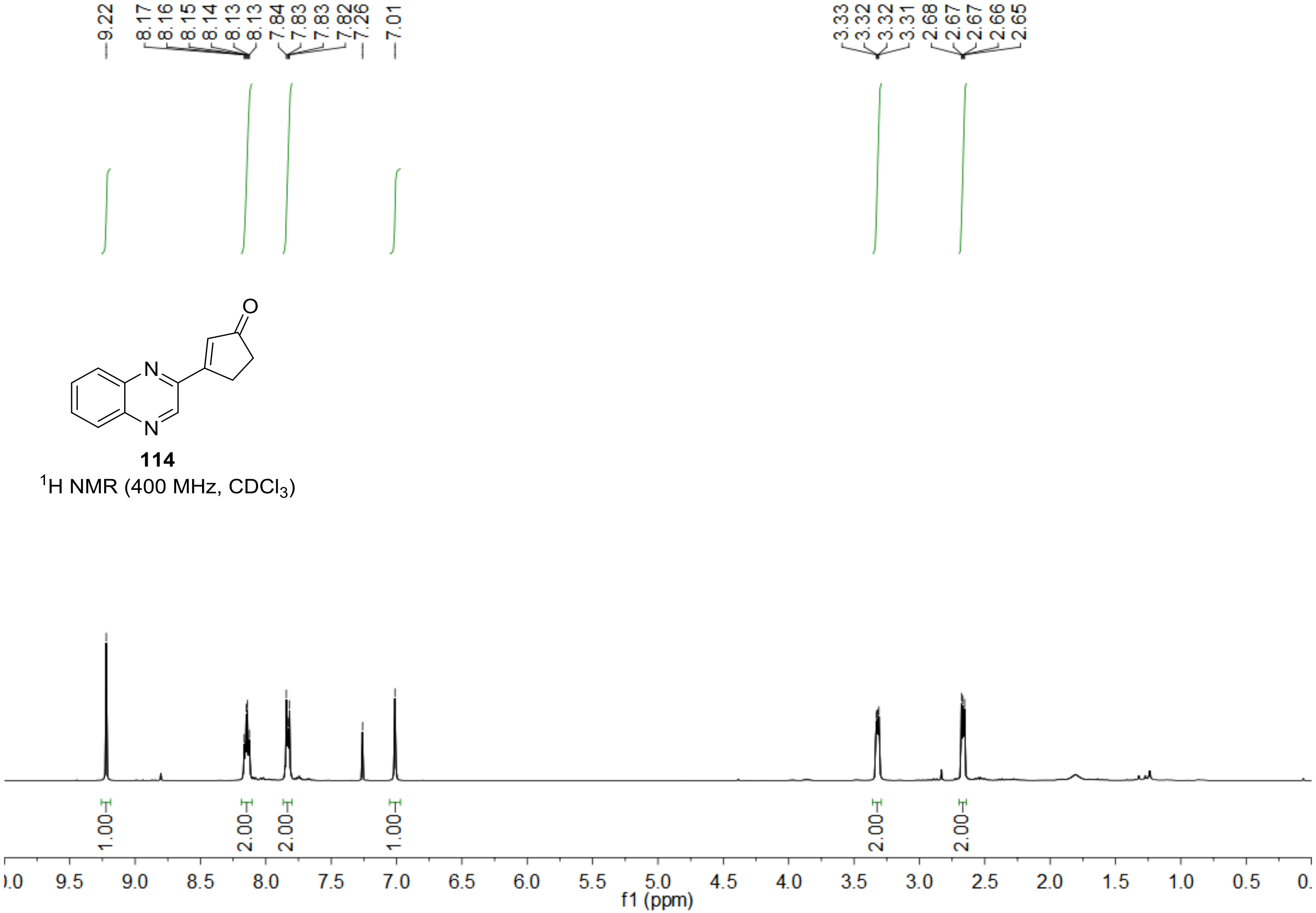

114

^{1}H NMR (400 MHz, $CDCl_3$)

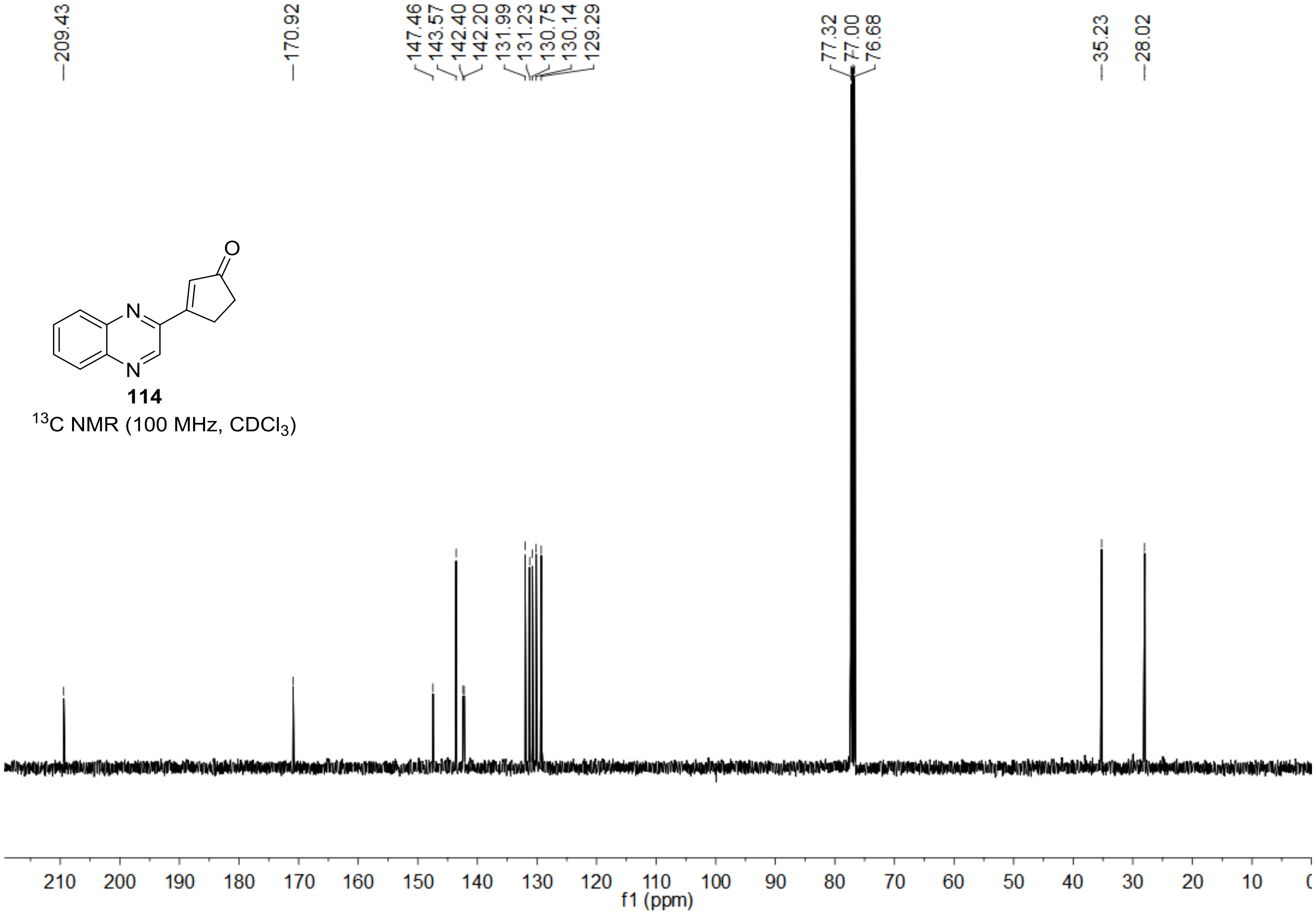

114

^{13}C NMR (100 MHz, $CDCl_3$)

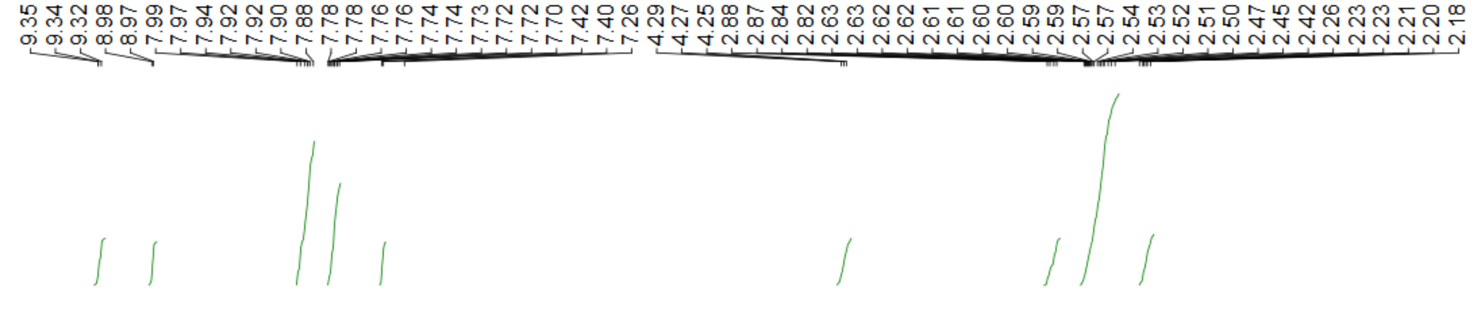

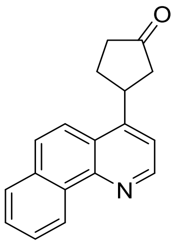

115-1

^{1}H NMR (400 MHz, $CDCl_3$)

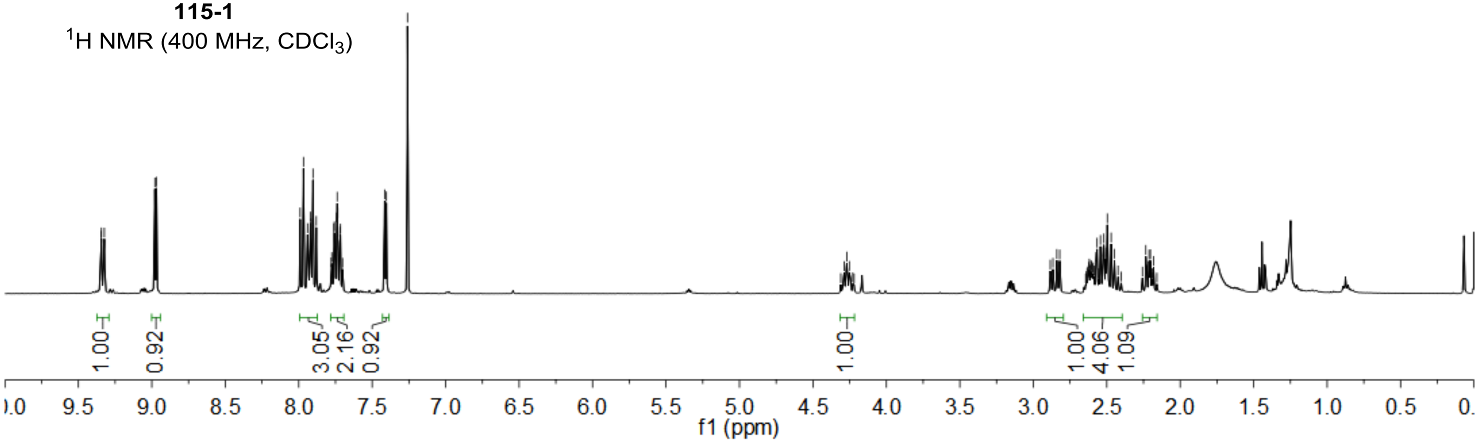

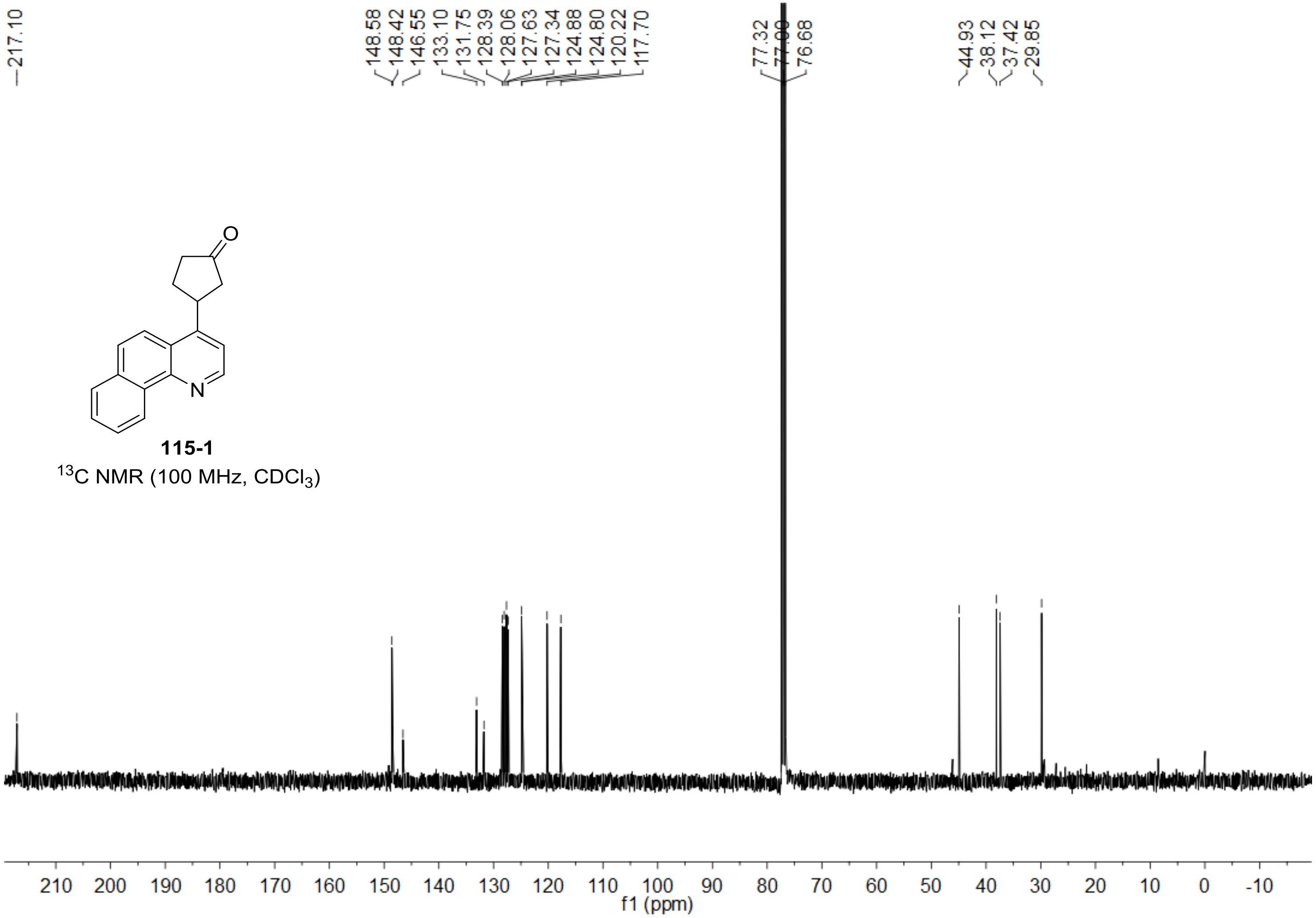

115-1

^{13}C NMR (100 MHz, $CDCl_3$)

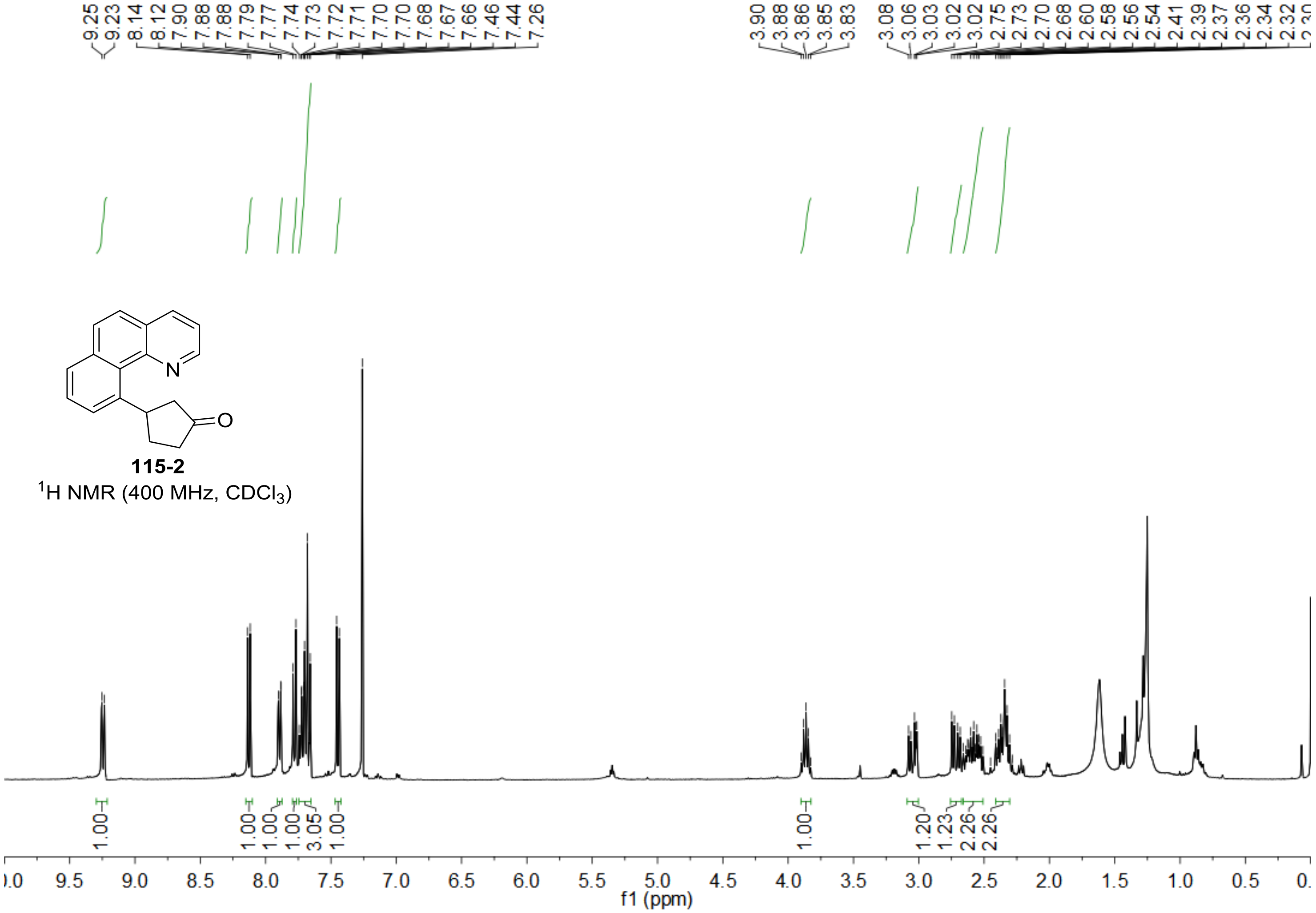

115-2

^{1}H NMR (400 MHz, $CDCl_3$)

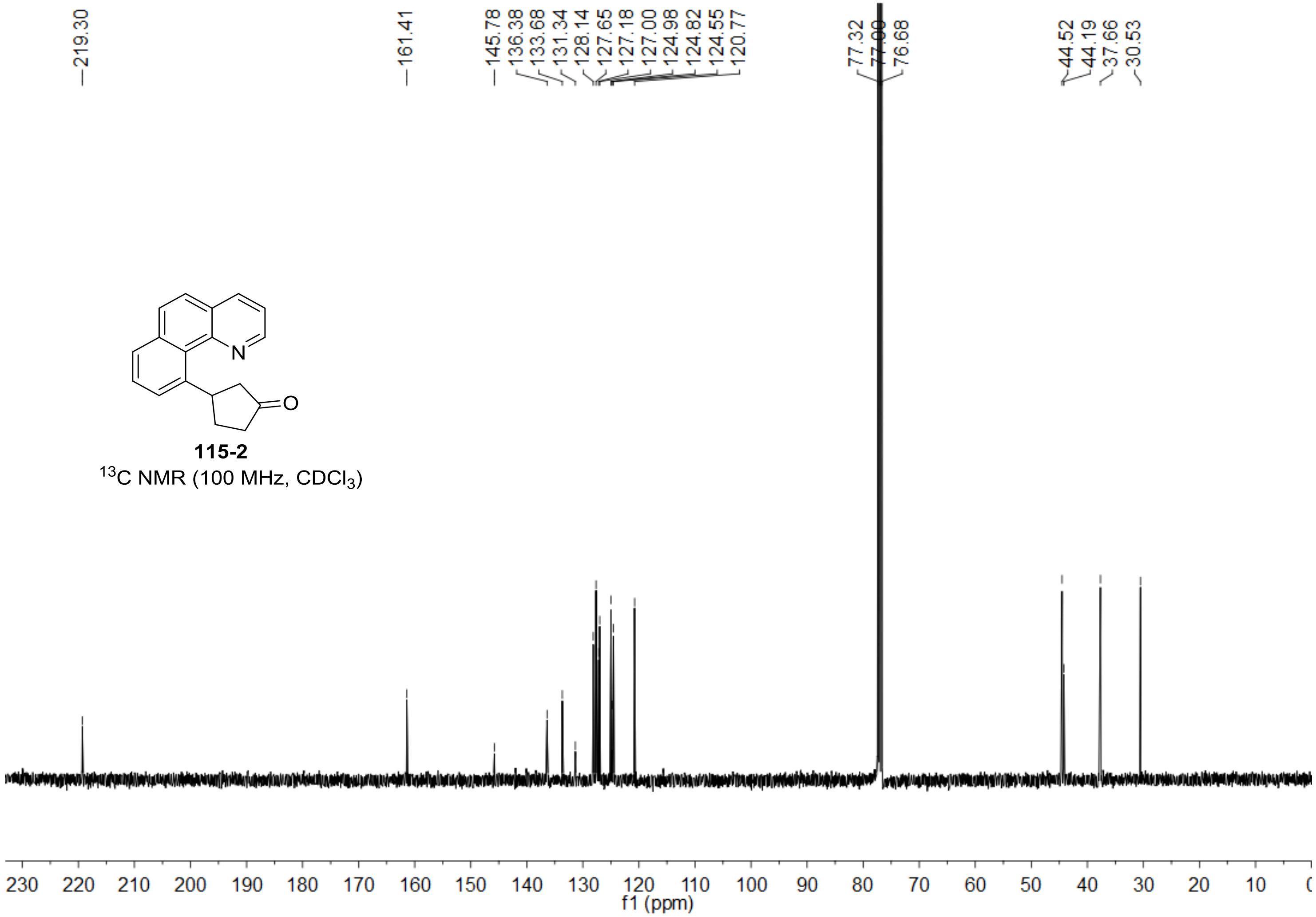

115-2

^{13}C NMR (100 MHz, $CDCl_3$)

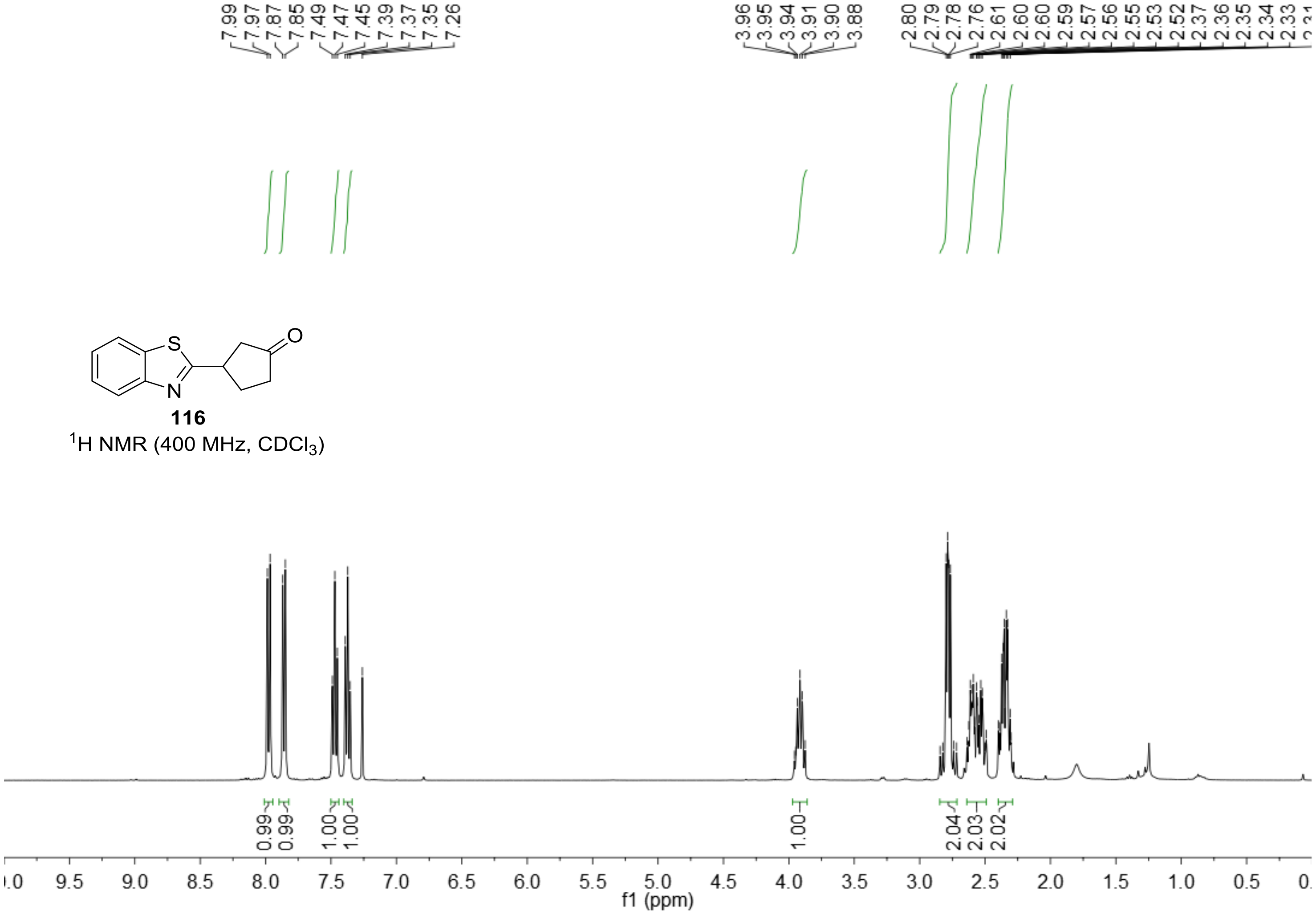

116

^{1}H NMR (400 MHz, $CDCl_3$)

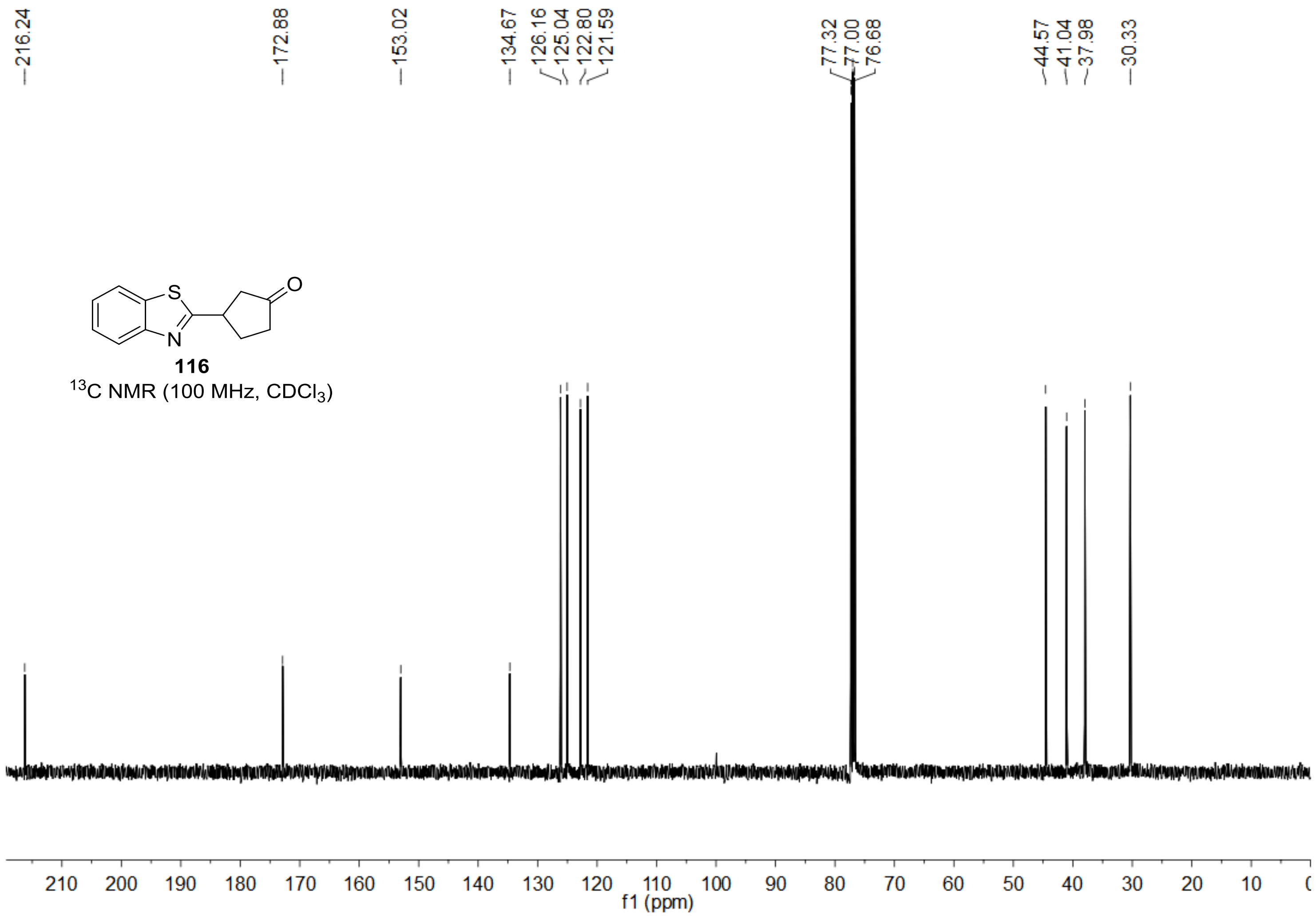
216.24
172.88
153.02
134.67
126.16
125.04
122.80
121.59
77.32
77.00
76.68
44.57
41.04
37.98
30.33
S
N
O
116
13C NMR (100 MHz, CDCl3)
210 200 190 180 170 160 150 140 130 120 110 100 90 80 70 60 50 40 30 20 10
f1 (ppm)

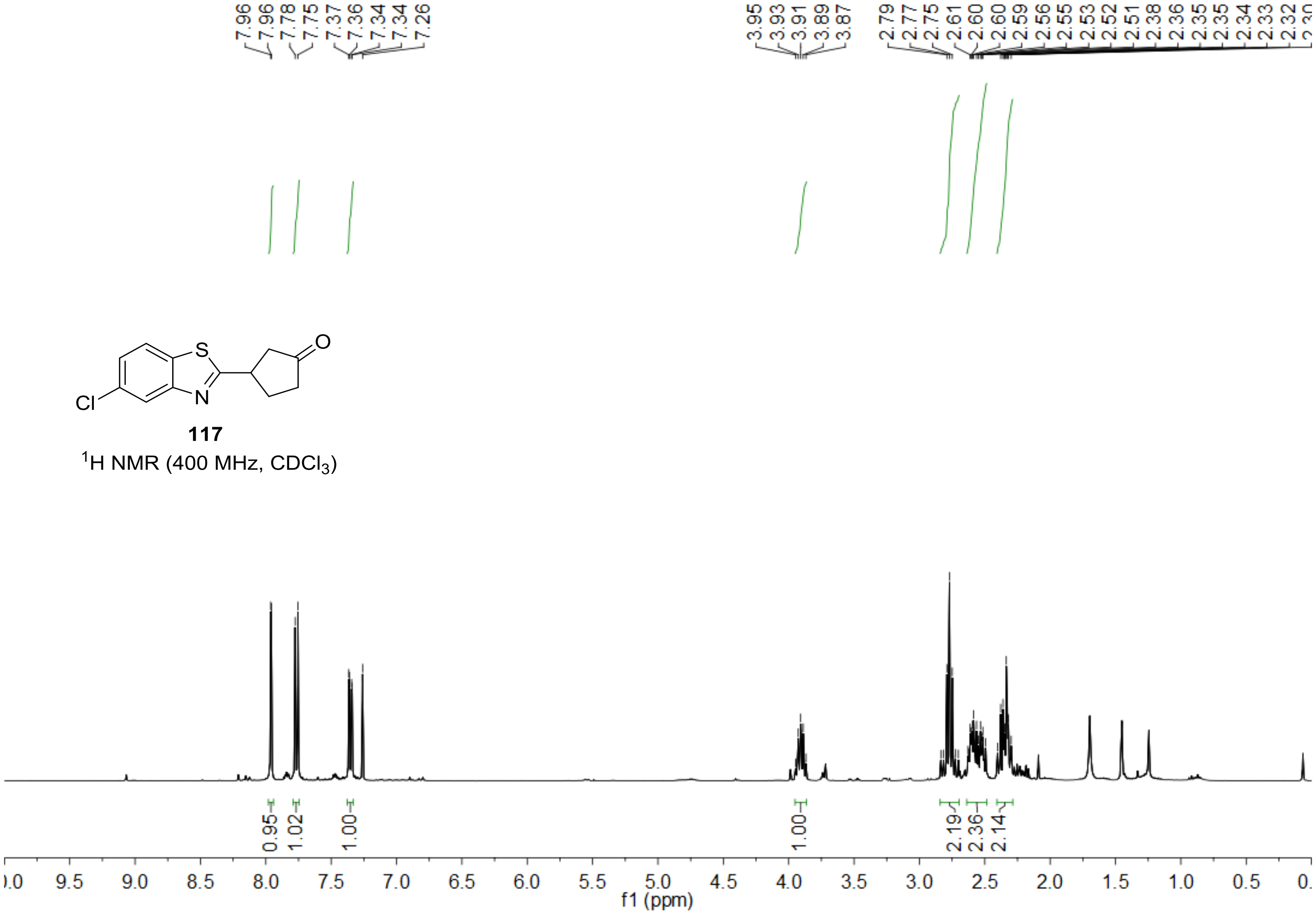

117

^{1}H NMR (400 MHz, $CDCl_3$)

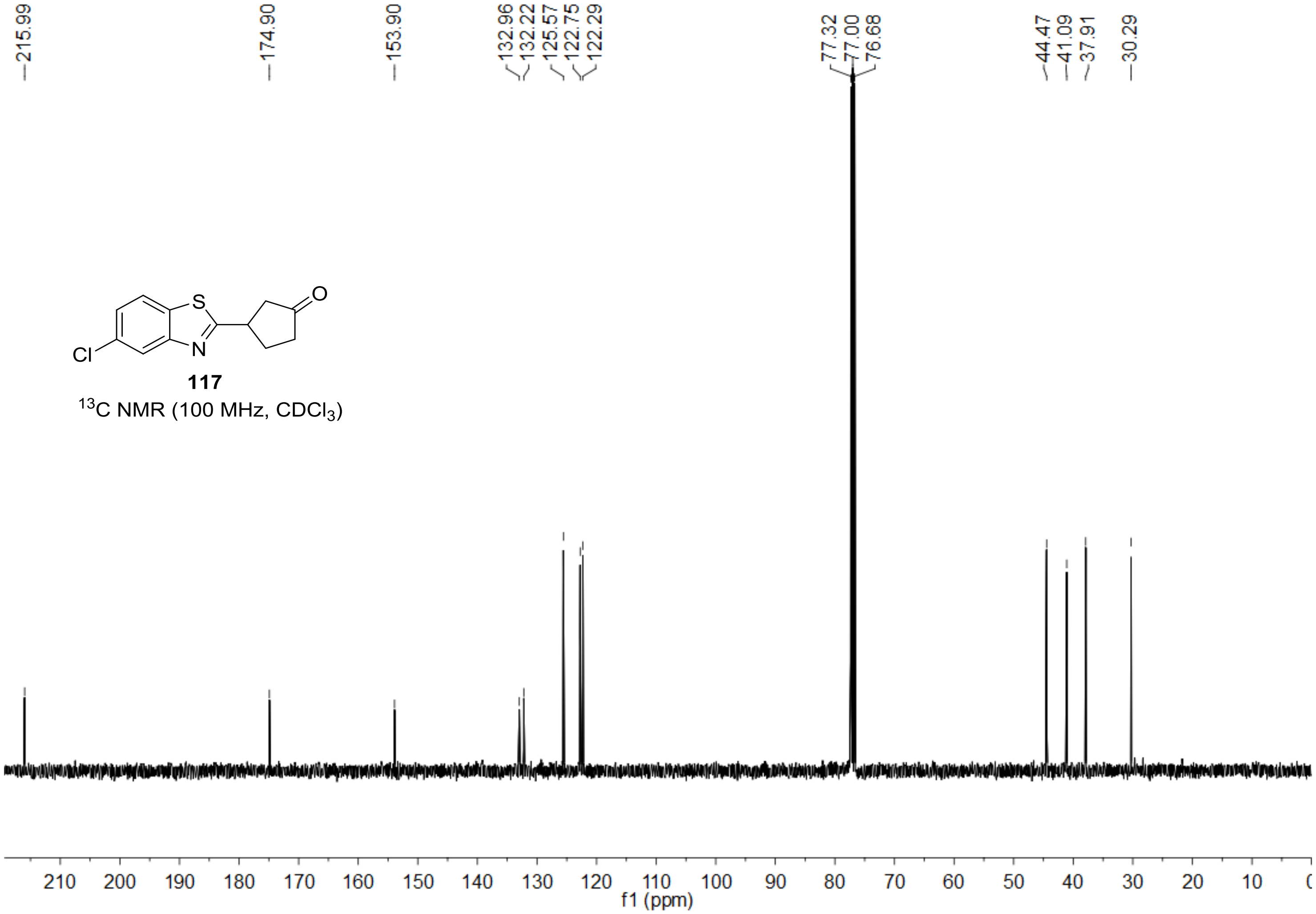
215.99
174.90
153.90
132.96
132.22
125.57
122.75
122.29
77.32
77.00
76.68
44.47
41.09
37.91
30.29
S
N
O
Cl
117
^{13}C NMR (100 MHz, $CDCl_3$)
210 200 190 180 170 160 150 140 130 120 110 100 90 80 70 60 50 40 30 20 10
f1 (ppm)

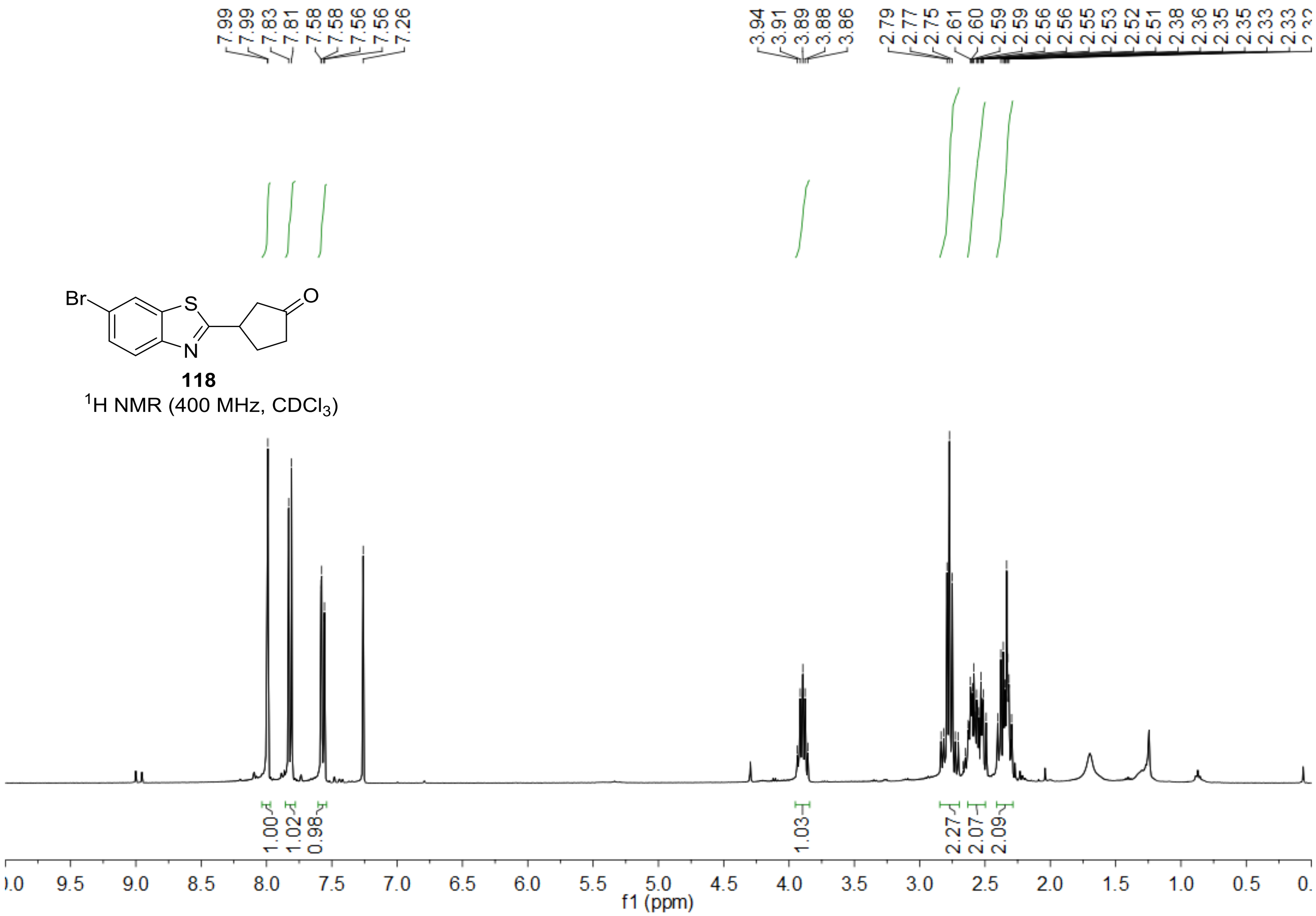

118

^{1}H NMR (400 MHz, $CDCl_3$)

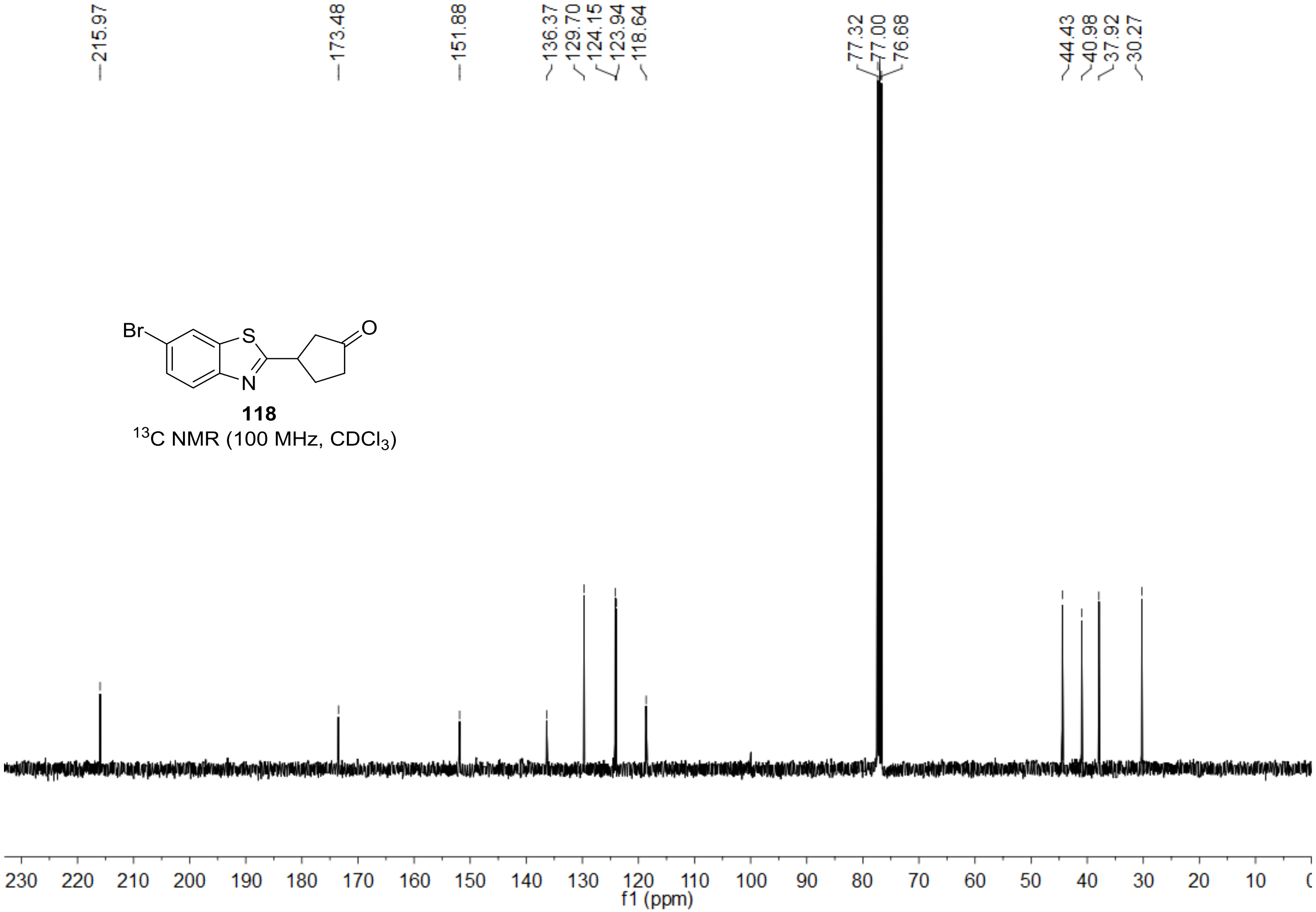

118

^{13}C NMR (100 MHz, $CDCl_3$)

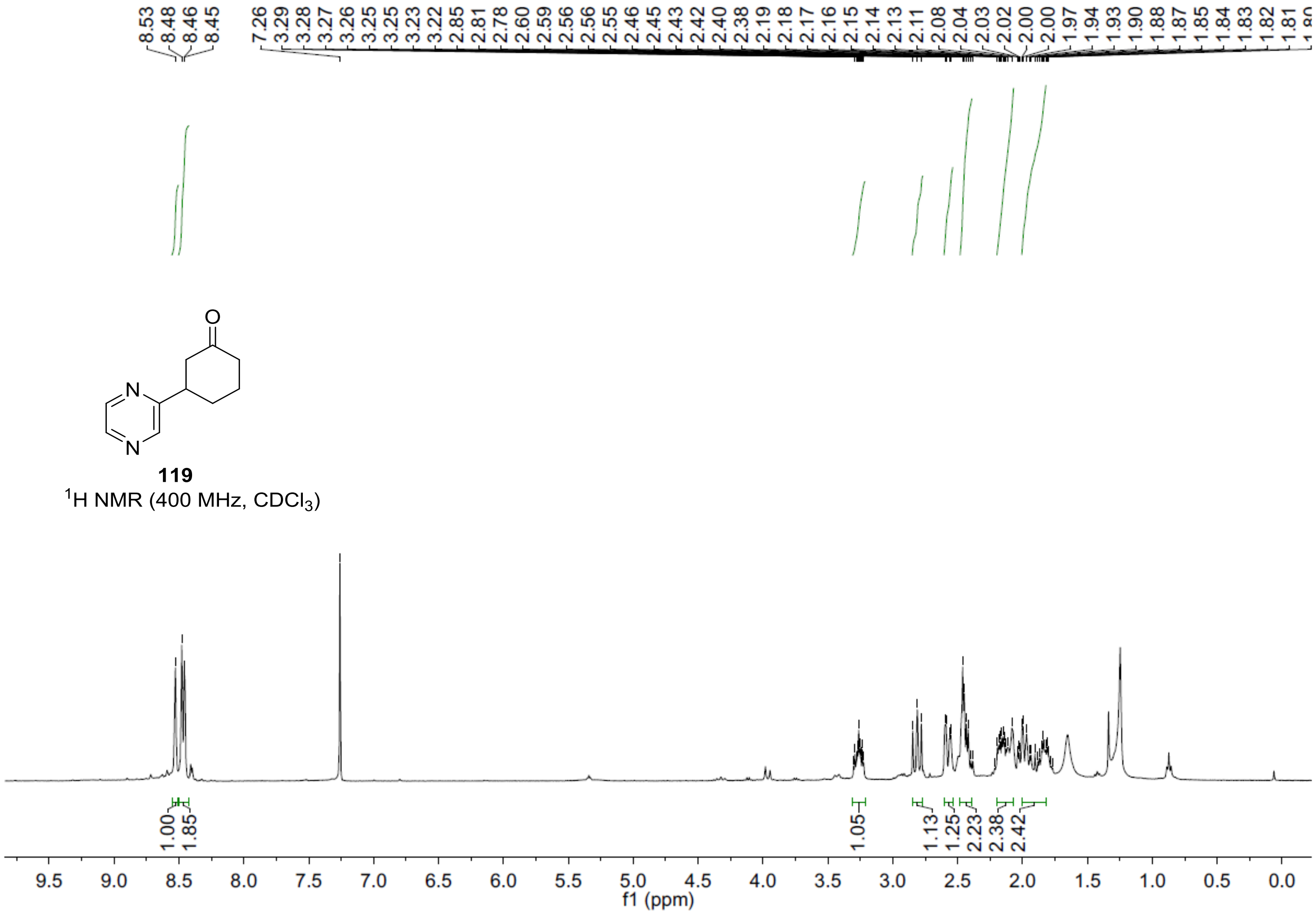

119

^{1}H NMR (400 MHz, $CDCl_3$)

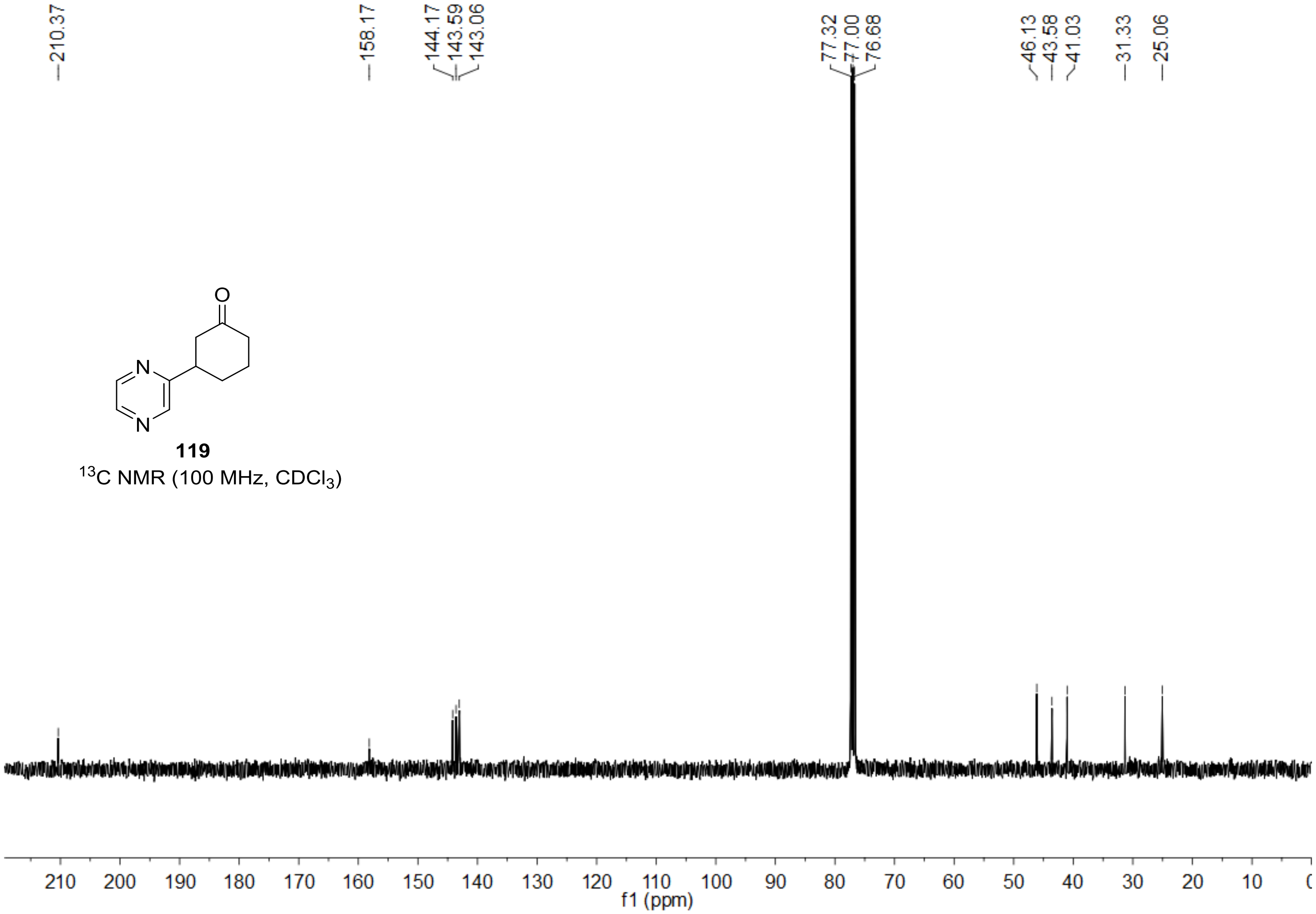
210.37
158.17
144.17
143.59
143.06
77.32
77.00
76.68
46.13
43.58
41.03
31.33
25.06
O
N
N
119
13C NMR (100 MHz, CDCl3)
210 200 190 180 170 160 150 140 130 120 110 100 90 80 70 60 50 40 30 20 10
f1 (ppm)

3 : 1

120

^{1}H NMR (400 MHz, $CDCl_3$)

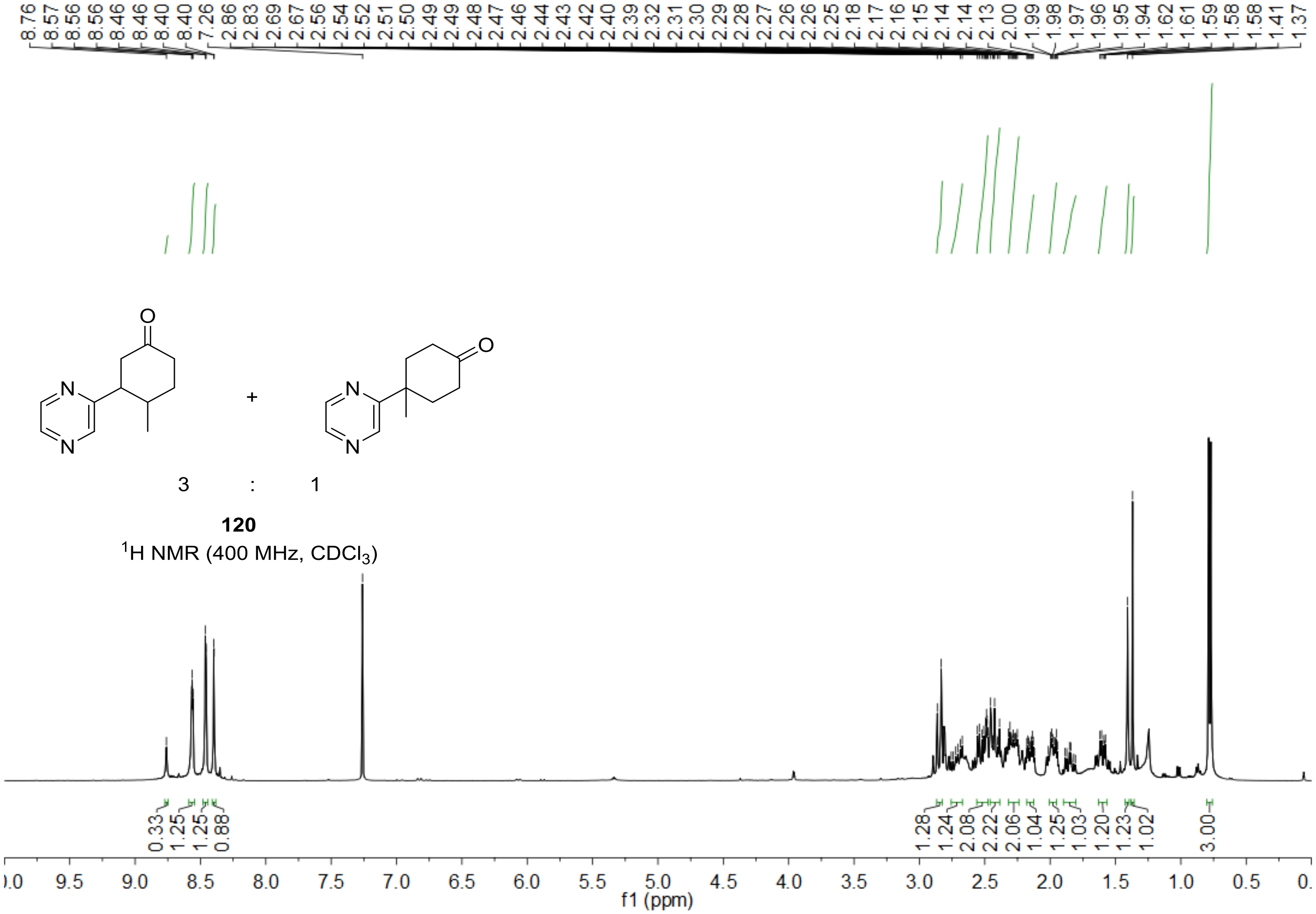

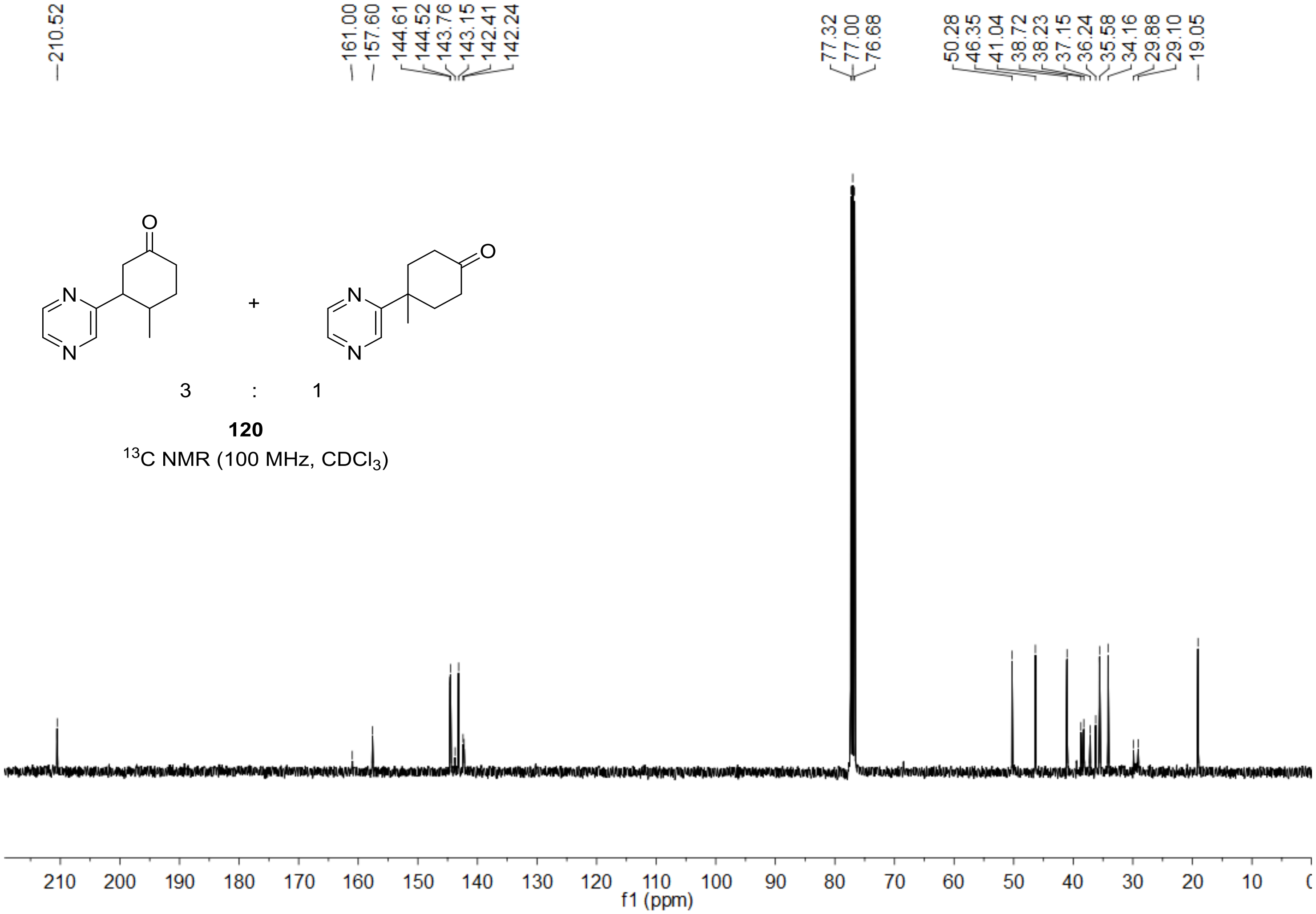

120

^{13}C NMR (100 MHz, $CDCl_3$)

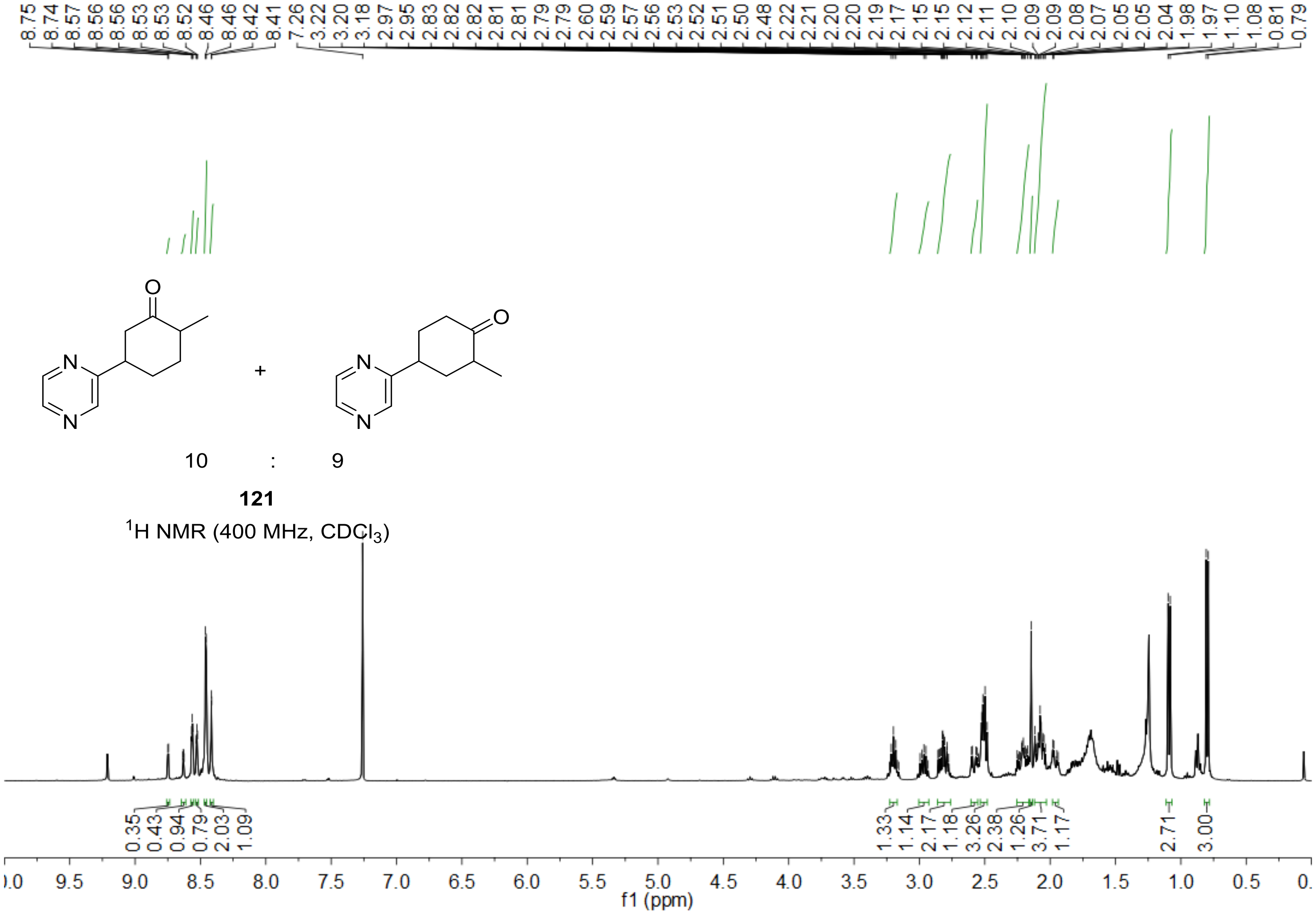
8.75 8.74 8.57 8.56 8.56 8.53 8.53 8.52 8.46 8.46 8.42 8.41 7.26 3.22 3.20 3.18 2.97 2.95 2.83 2.82 2.82 2.81 2.81 2.79 2.79 2.60 2.59 2.57 2.56 2.53 2.52 2.51 2.50 2.48 2.22 2.21 2.20 2.20 2.19 2.17 2.15 2.15 2.12 2.11 2.10 2.09 2.09 2.08 2.07 2.05 2.04 1.98 1.97 1.10 1.08 0.81 0.79
O
N
N
+
O
N
N
10 : 9
121
^{1}H NMR (400 MHz, $CDCl_3$)
0.35 0.43 0.94 0.79 2.03 1.09 1.33 1.14 2.17 1.18 3.26 2.38 1.26 3.71 1.17 2.71 3.00
9.5 9.0 8.5 8.0 7.5 7.0 6.5 6.0 5.5 5.0 4.5 4.0 3.5 3.0 2.5 2.0 1.5 1.0 0.5
f1 (ppm)

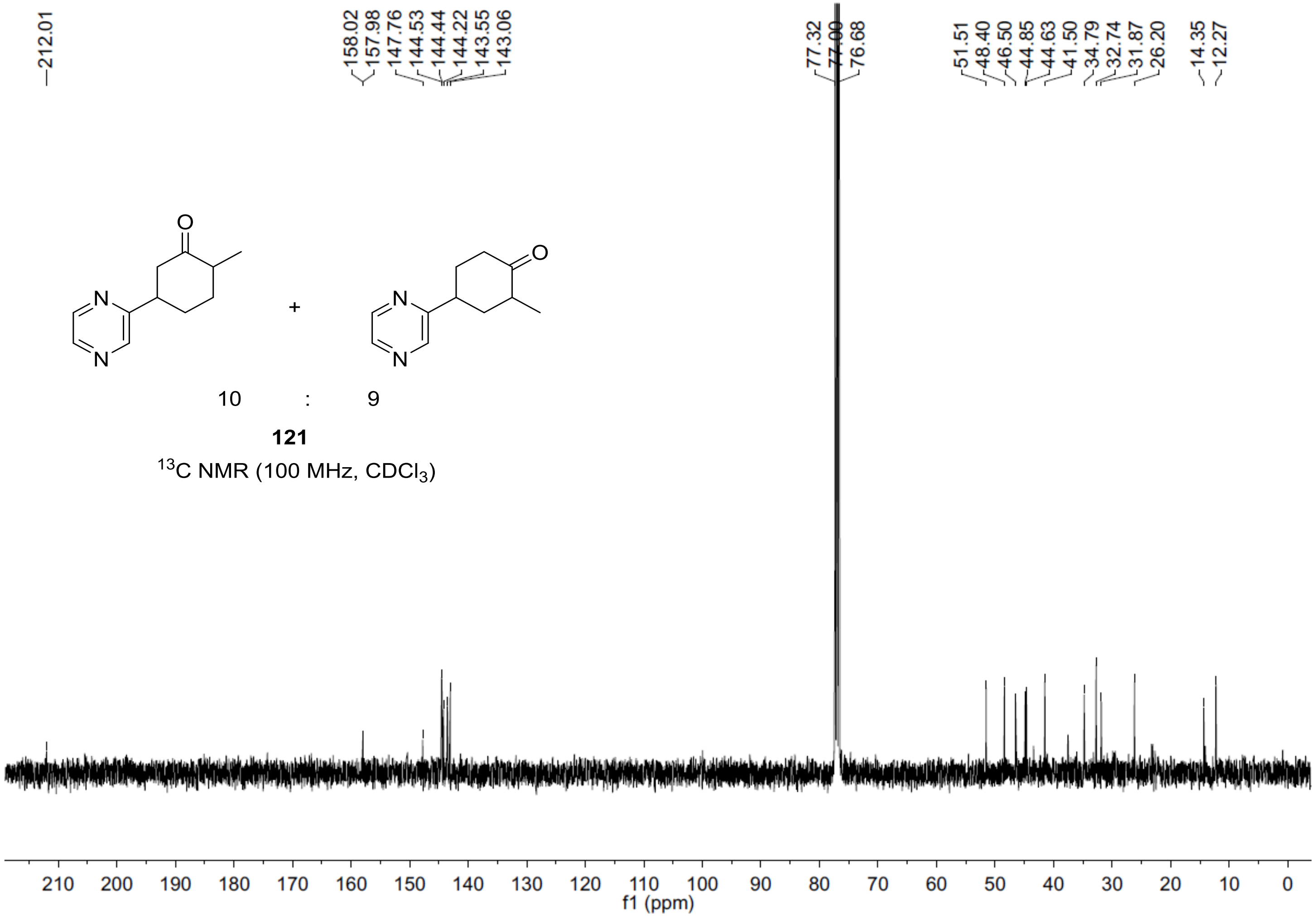
212.01
158.02
157.98
147.76
144.53
144.44
144.22
143.55
143.06
77.32
77.00
76.68
51.51
48.40
46.50
44.85
44.63
41.50
34.79
32.74
31.87
26.20
14.35
12.27
10 : 9
121
13C NMR (100 MHz, CDCl3)
210 200 190 180 170 160 150 140 130 120 110 100 90 80 70 60 50 40 30 20 10 0
f1 (ppm)

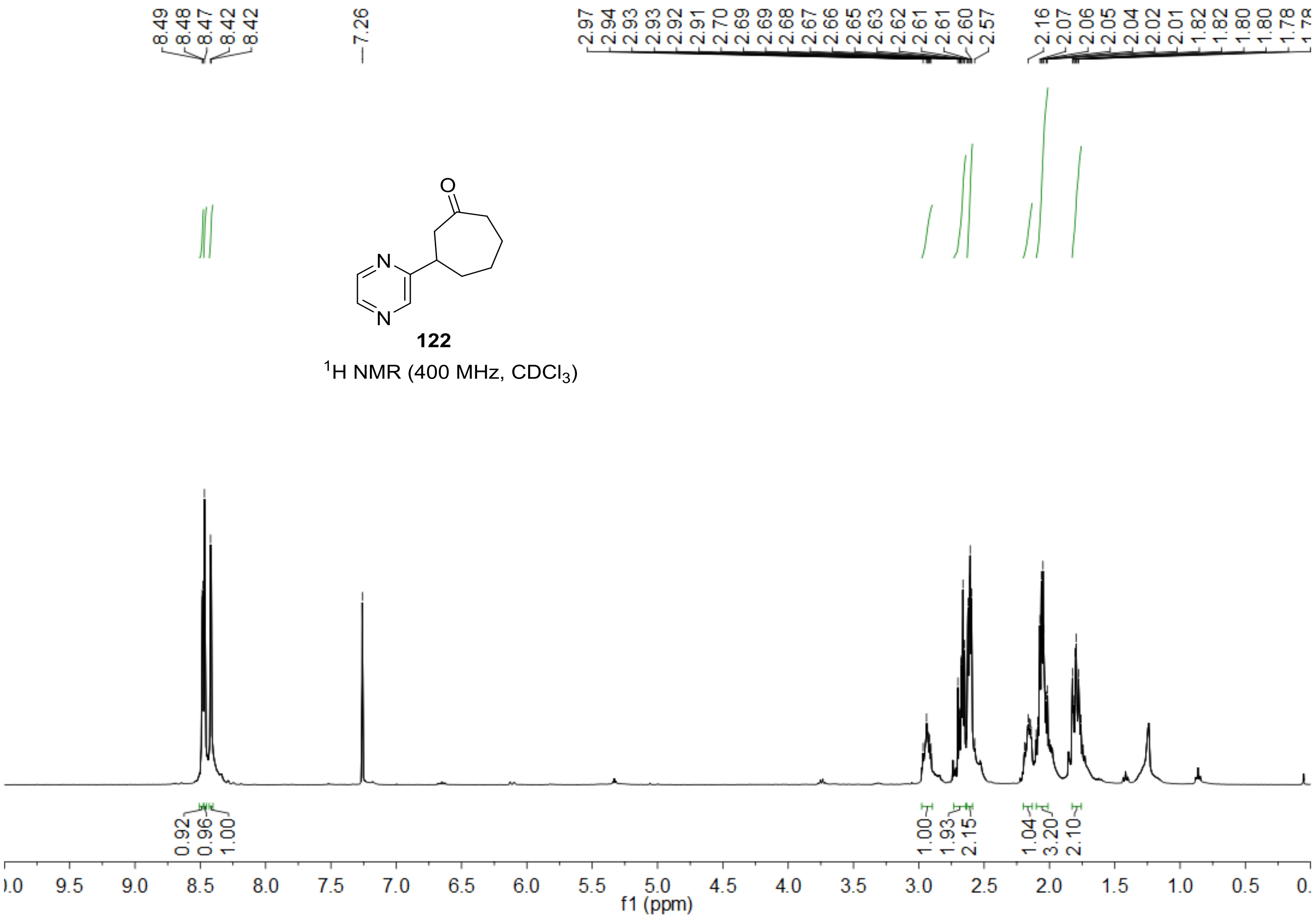
8.49
8.48
8.47
8.42
8.42
7.26
2.97
2.94
2.93
2.92
2.91
2.70
2.69
2.69
2.68
2.67
2.66
2.65
2.63
2.62
2.61
2.61
2.60
2.57
2.16
2.07
2.06
2.05
2.04
2.02
2.01
1.82
1.82
1.80
1.80
1.78
O
N
N
122
^{1}H NMR (400 MHz, CDCl$_3$)
0.92
0.96
1.00
1.00
1.93
2.15
1.04
3.20
2.10
9.5
9.0
8.5
8.0
7.5
7.0
6.5
6.0
5.5
5.0
4.5
4.0
3.5
3.0
2.5
2.0
1.5
1.0
0.5
f1 (ppm)

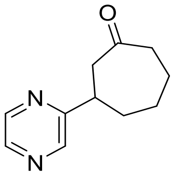

122

^{13}C NMR (100 MHz, $CDCl_3$)

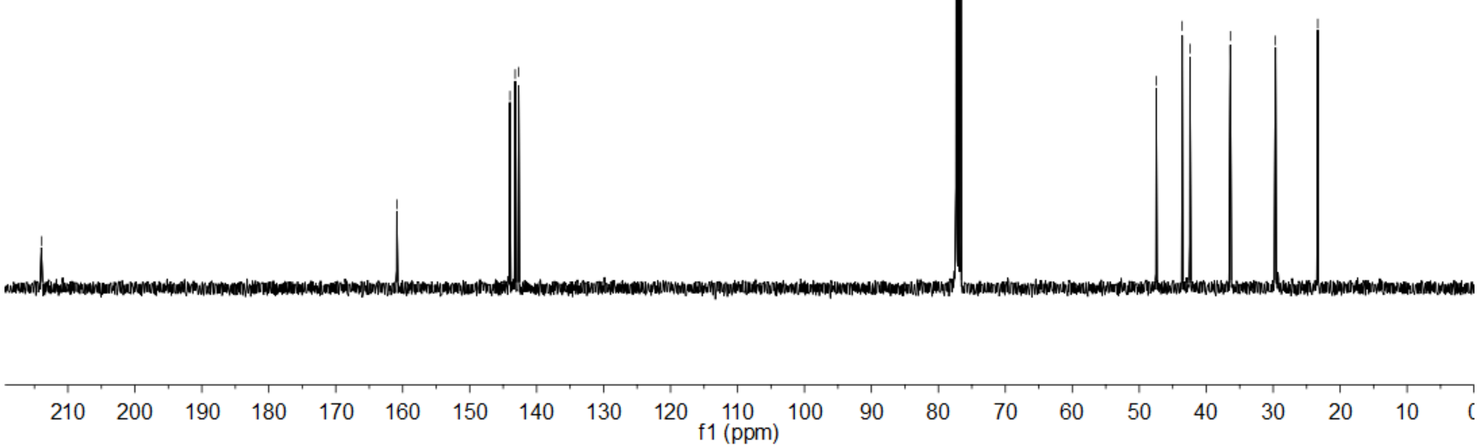

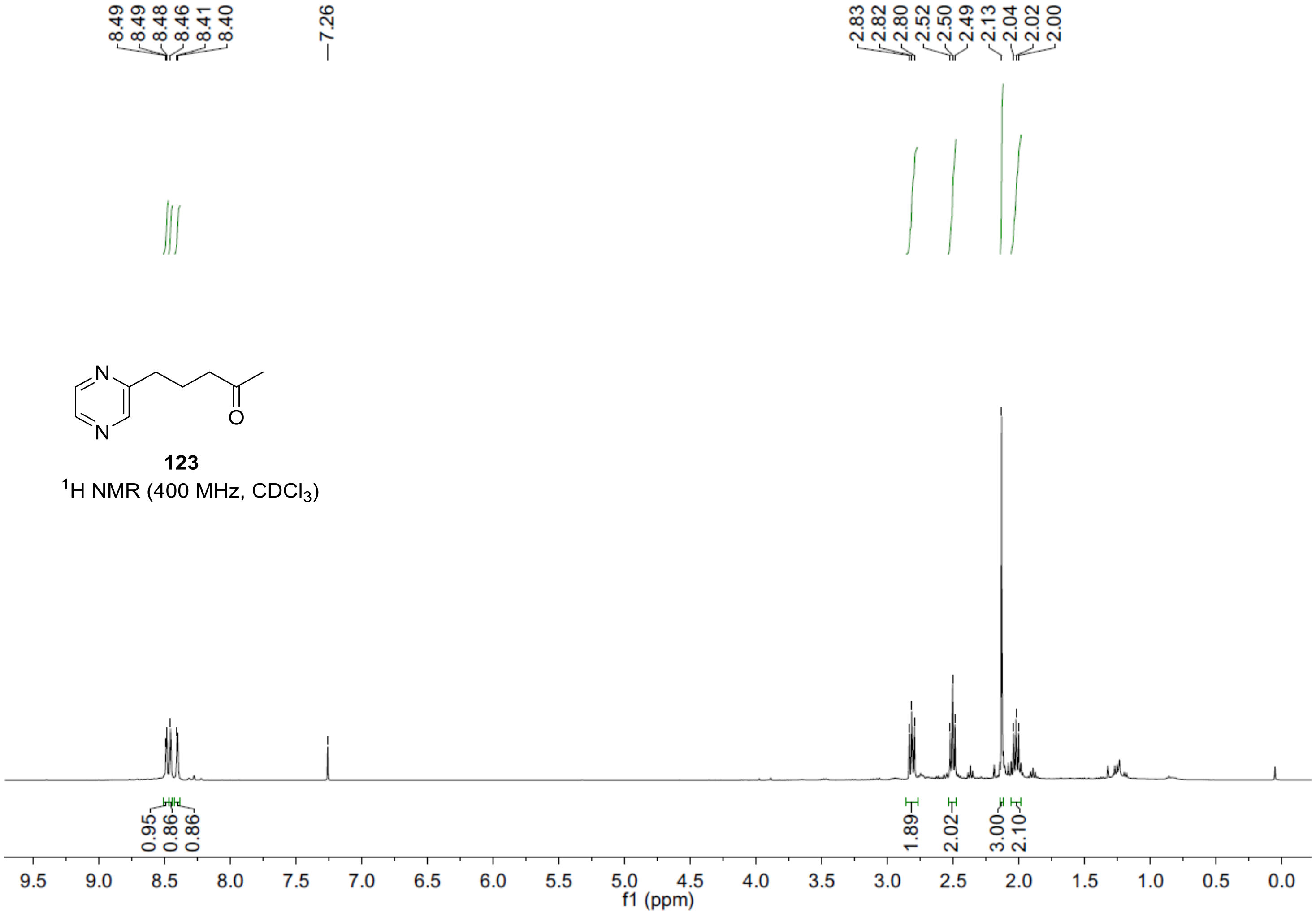

123

^{1}H NMR (400 MHz, $CDCl_3$)

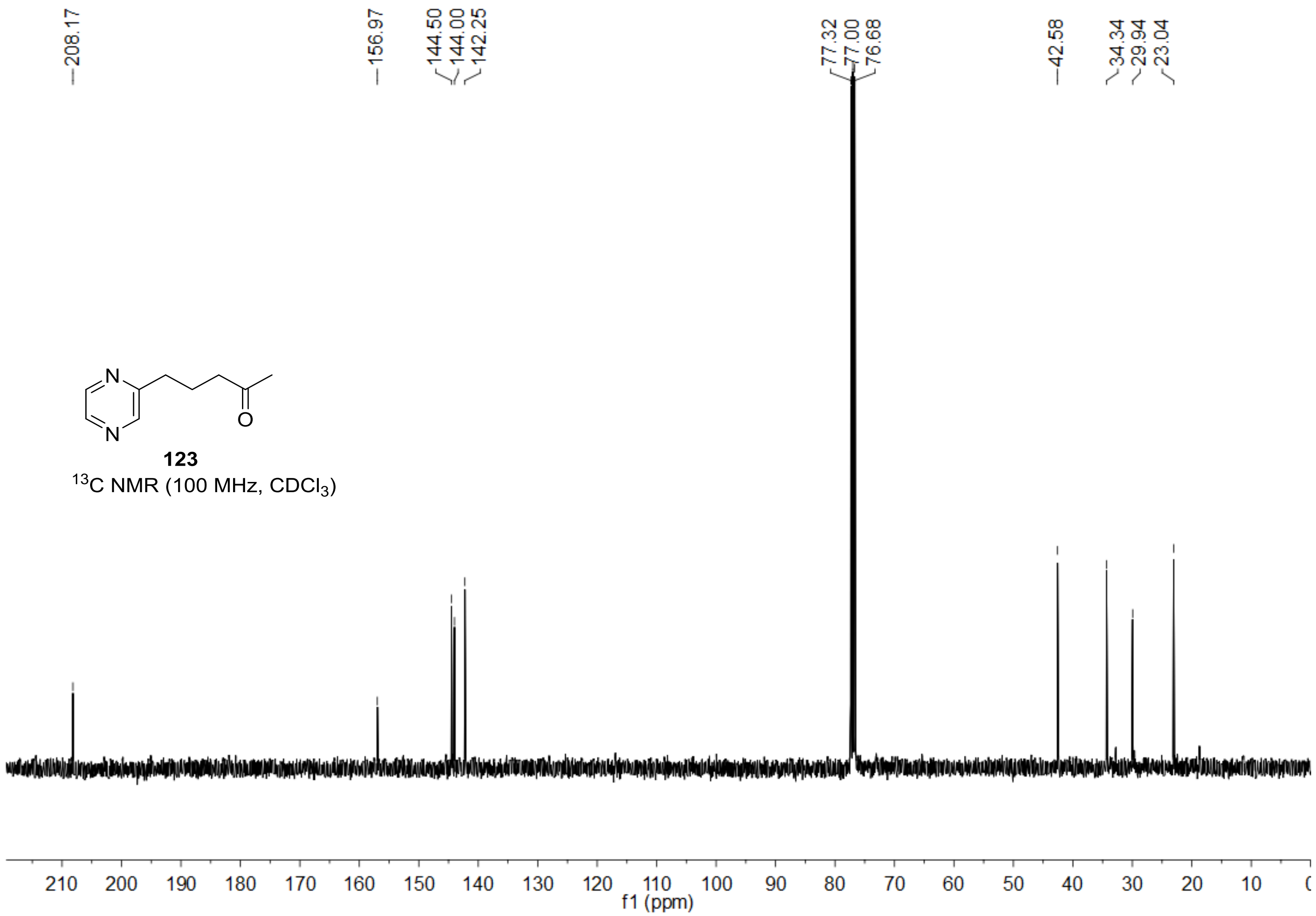

123

^{13}C NMR (100 MHz, $CDCl_3$)

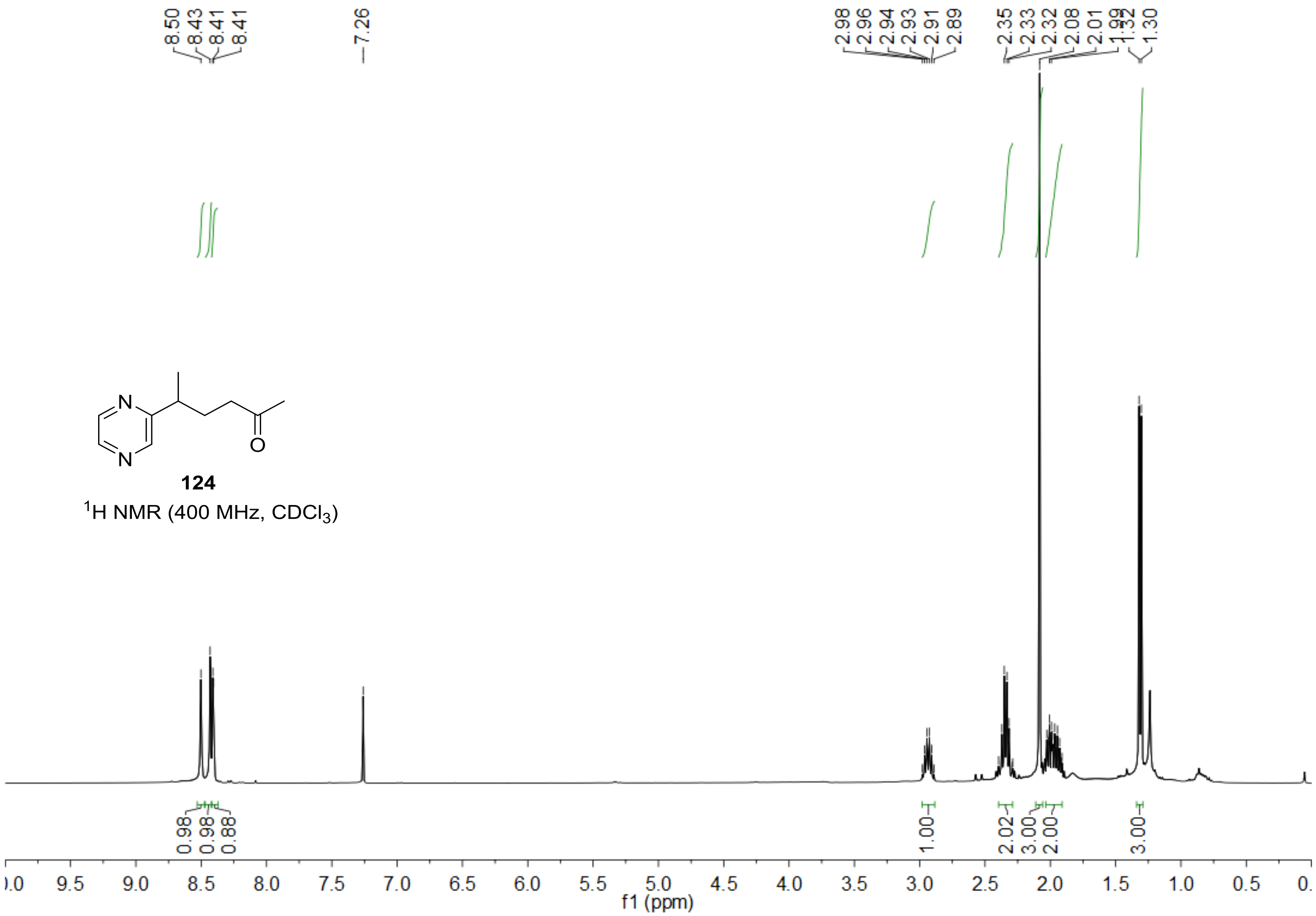

124

^{1}H NMR (400 MHz, $CDCl_3$)

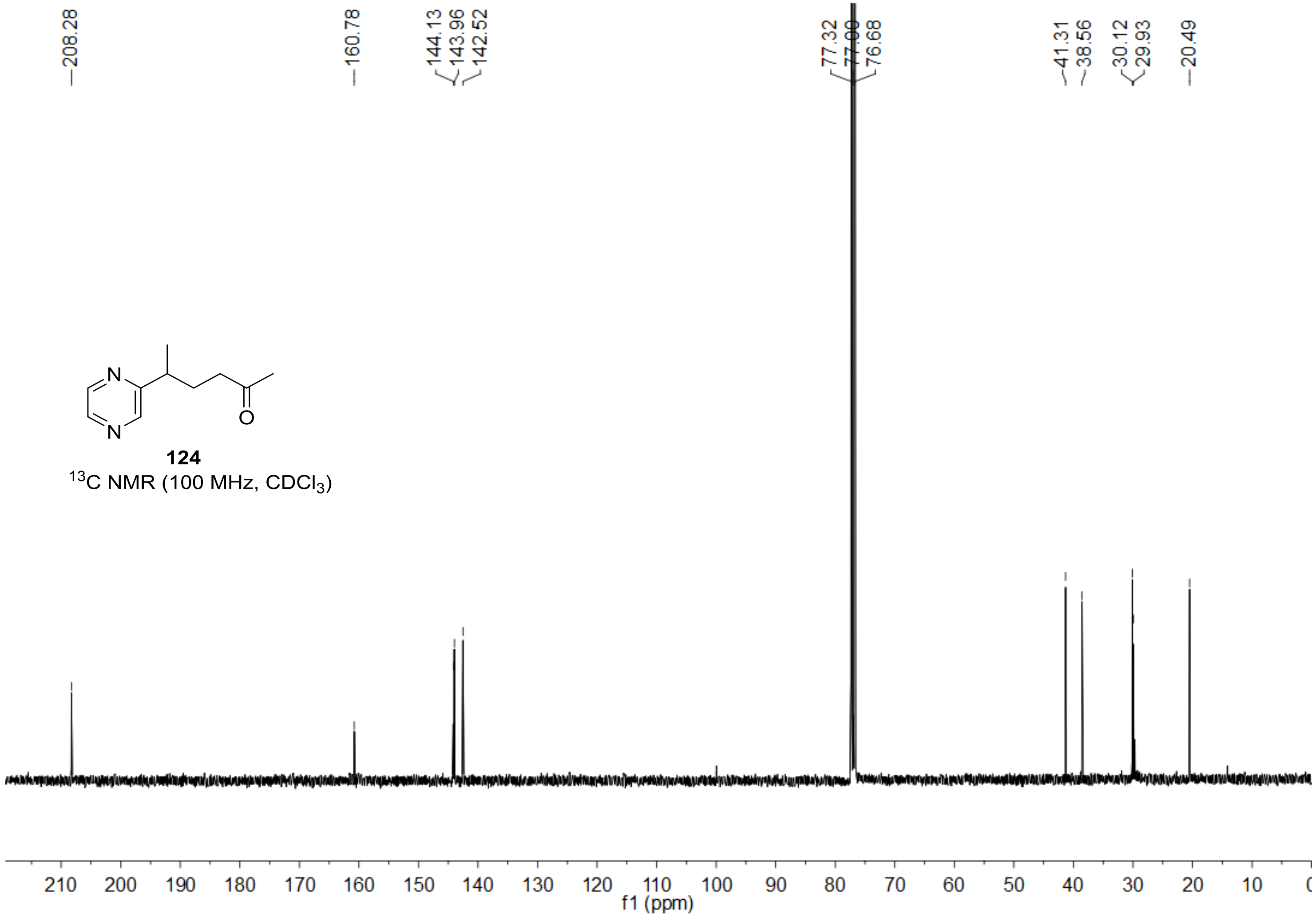

208.28
160.78
144.13
143.96
142.52
77.32
77.00
76.68
41.31
38.56
30.12
29.93
20.49
N
N
O
124
^{13}C NMR (100 MHz, $CDCl_3$)
210 200 190 180 170 160 150 140 130 120 110 100 90 80 70 60 50 40 30 20 10 0
f1 (ppm)

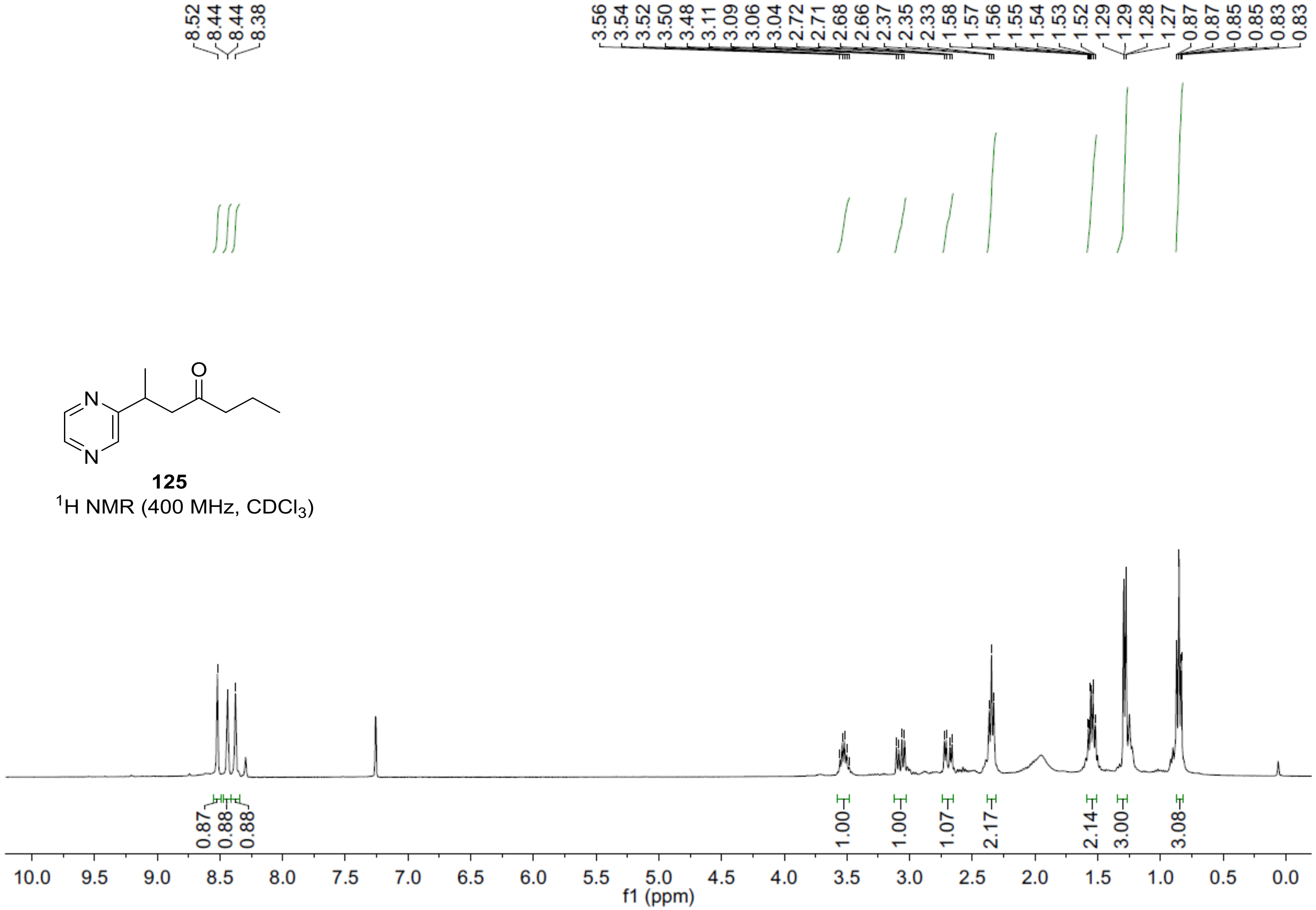

125

^1H NMR (400 MHz, $CDCl_3$)

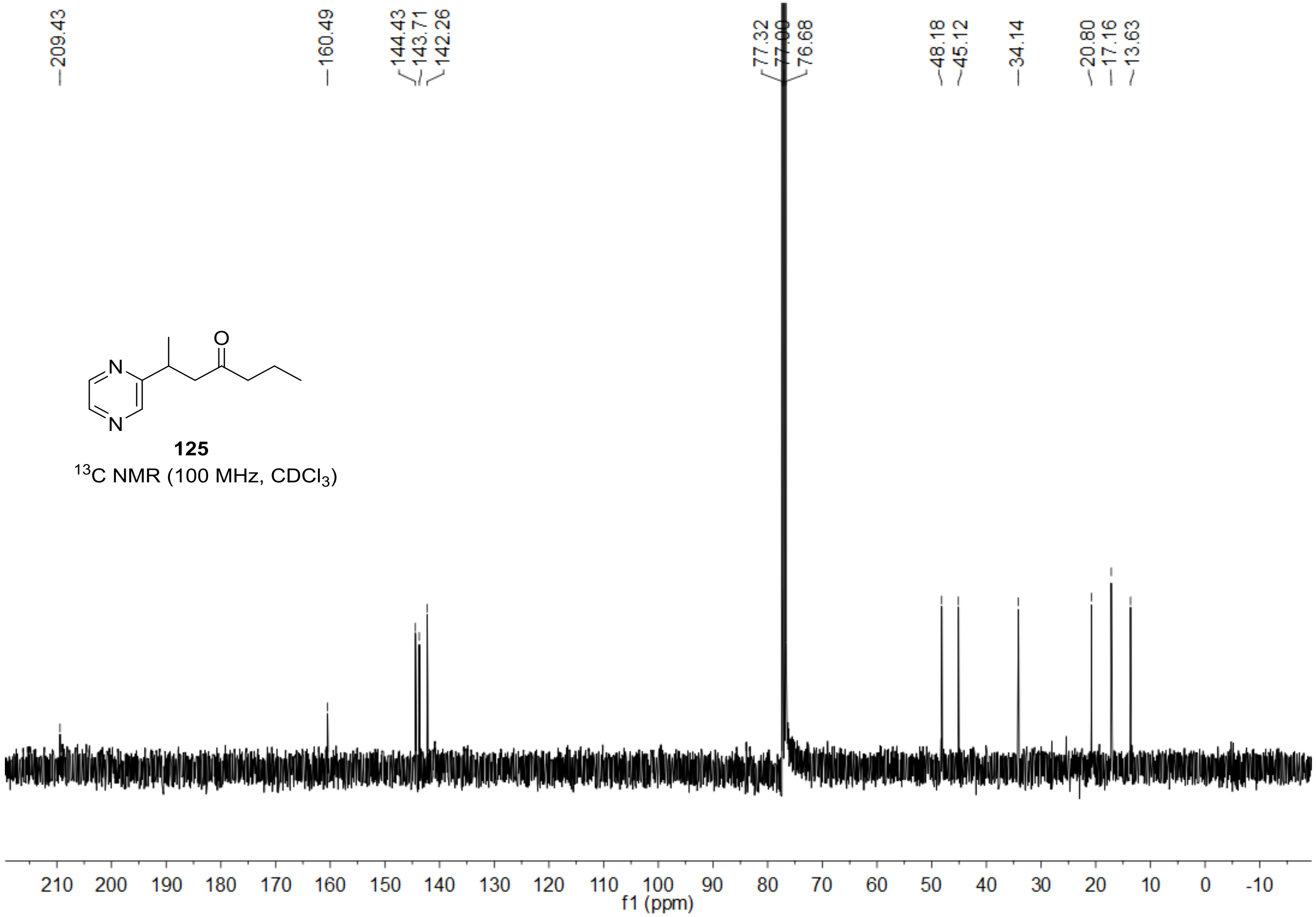

125

^{13}C NMR (100 MHz, $CDCl_3$)

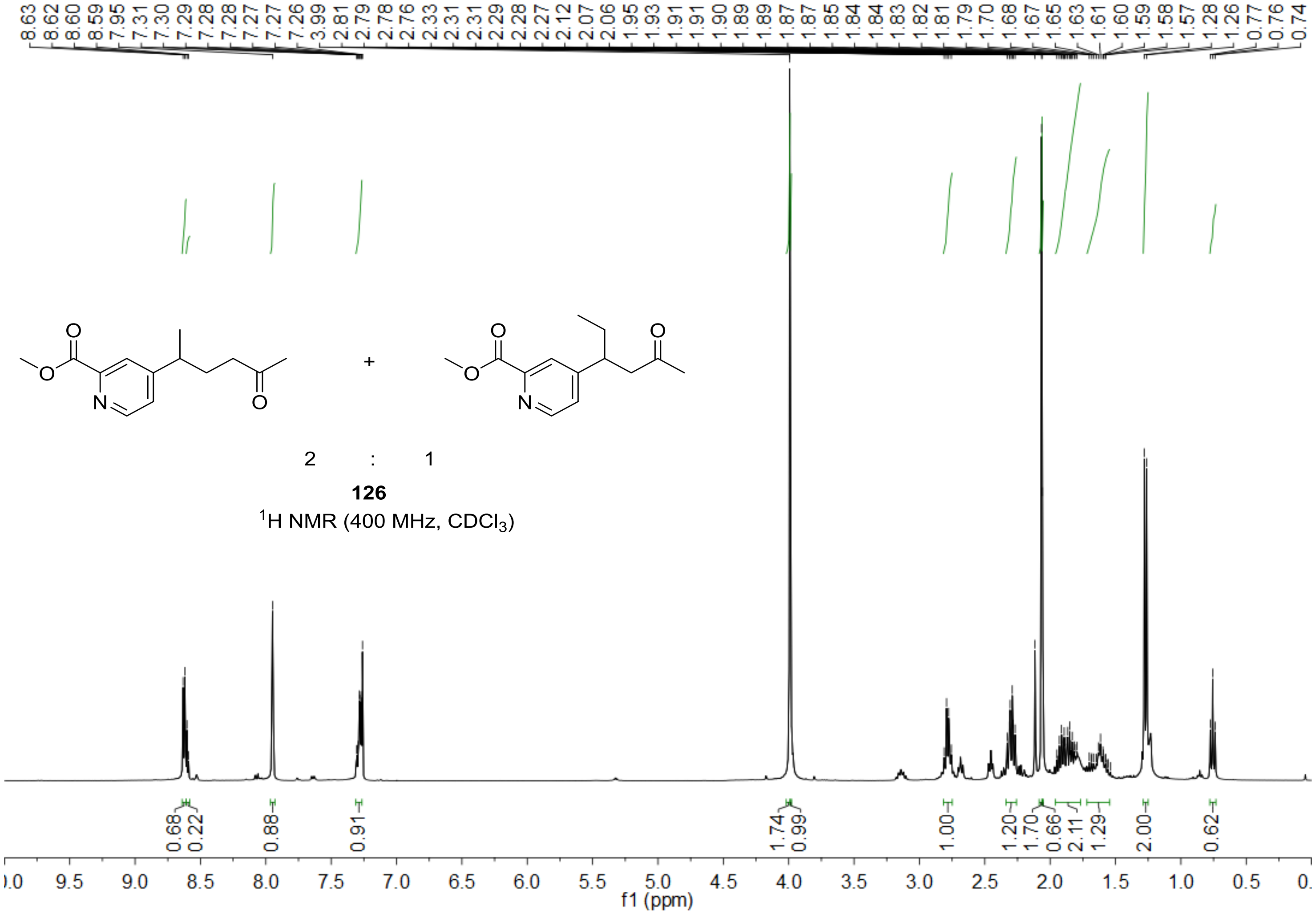

2 : 1

126

^{1}H NMR (400 MHz, $CDCl_3$)

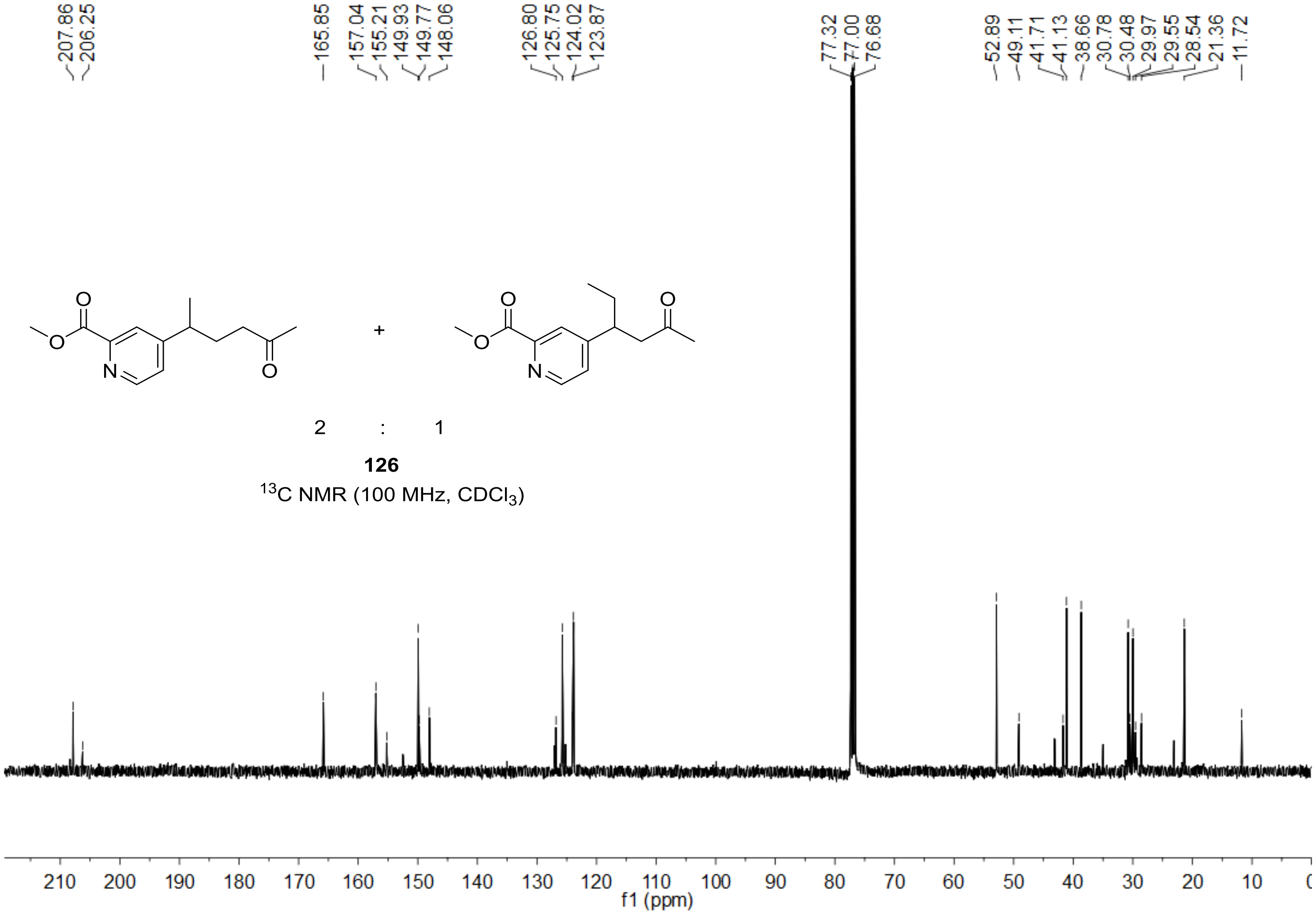

2 : 1

126

^{13}C NMR (100 MHz, $CDCl_3$)

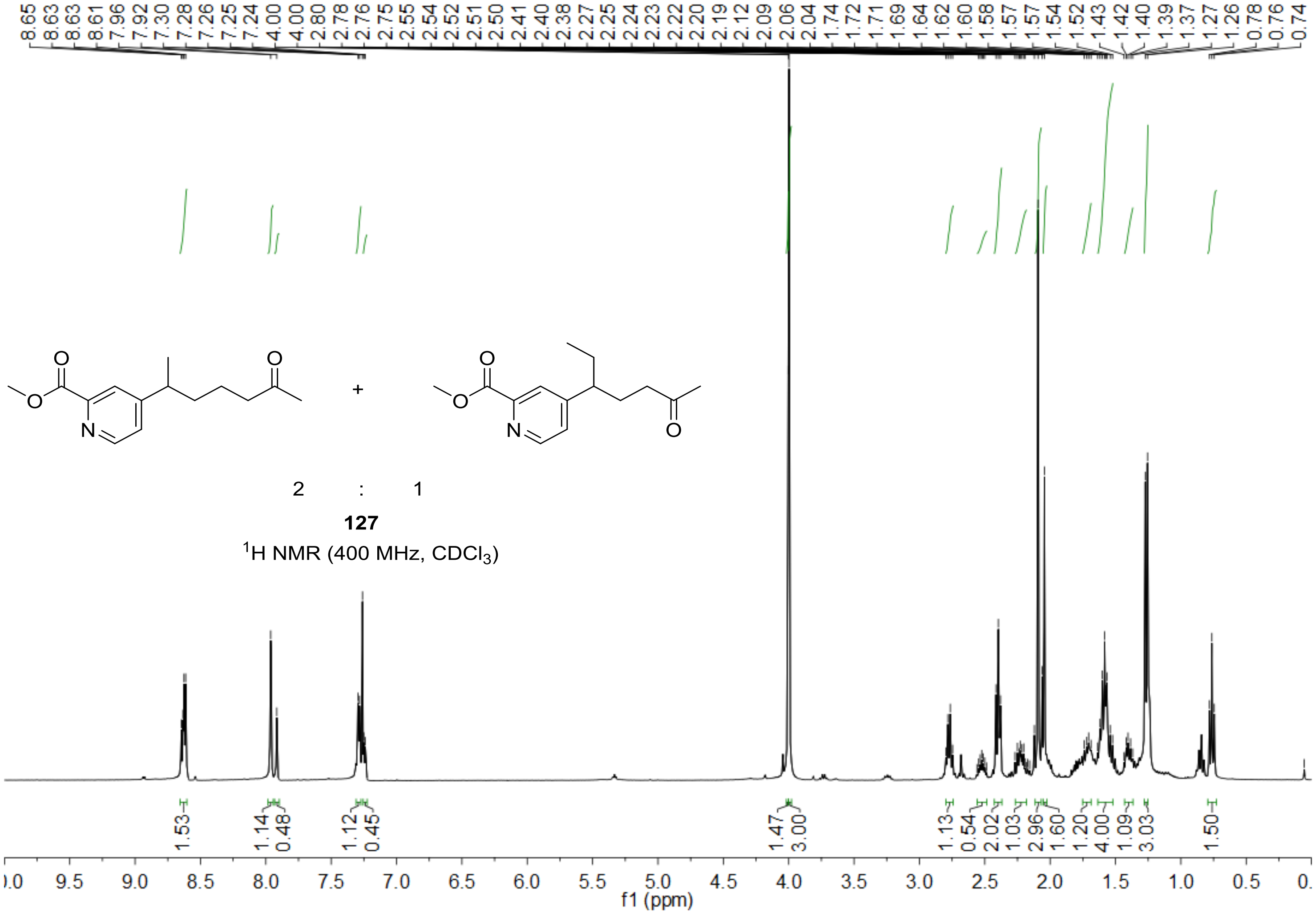
8.65 8.63 8.63 8.61 7.96 7.92 7.30 7.28 7.26 7.25 7.24 4.00 4.00 2.80 2.78 2.76 2.75 2.55 2.54 2.52 2.51 2.50 2.41 2.40 2.38 2.27 2.25 2.24 2.23 2.22 2.20 2.19 2.12 2.09 2.06 2.04 1.74 1.72 1.71 1.69 1.64 1.62 1.60 1.58 1.57 1.57 1.54 1.52 1.43 1.42 1.40 1.39 1.37 1.27 1.26 0.78 0.76 0.74
2 : 1
127
1H NMR (400 MHz, CDCl3)
1.53 1.14 0.48 1.12 0.45 1.47 3.00 1.13 0.54 2.02 1.03 2.96 1.60 1.20 4.00 1.09 3.03 1.50
f1 (ppm)

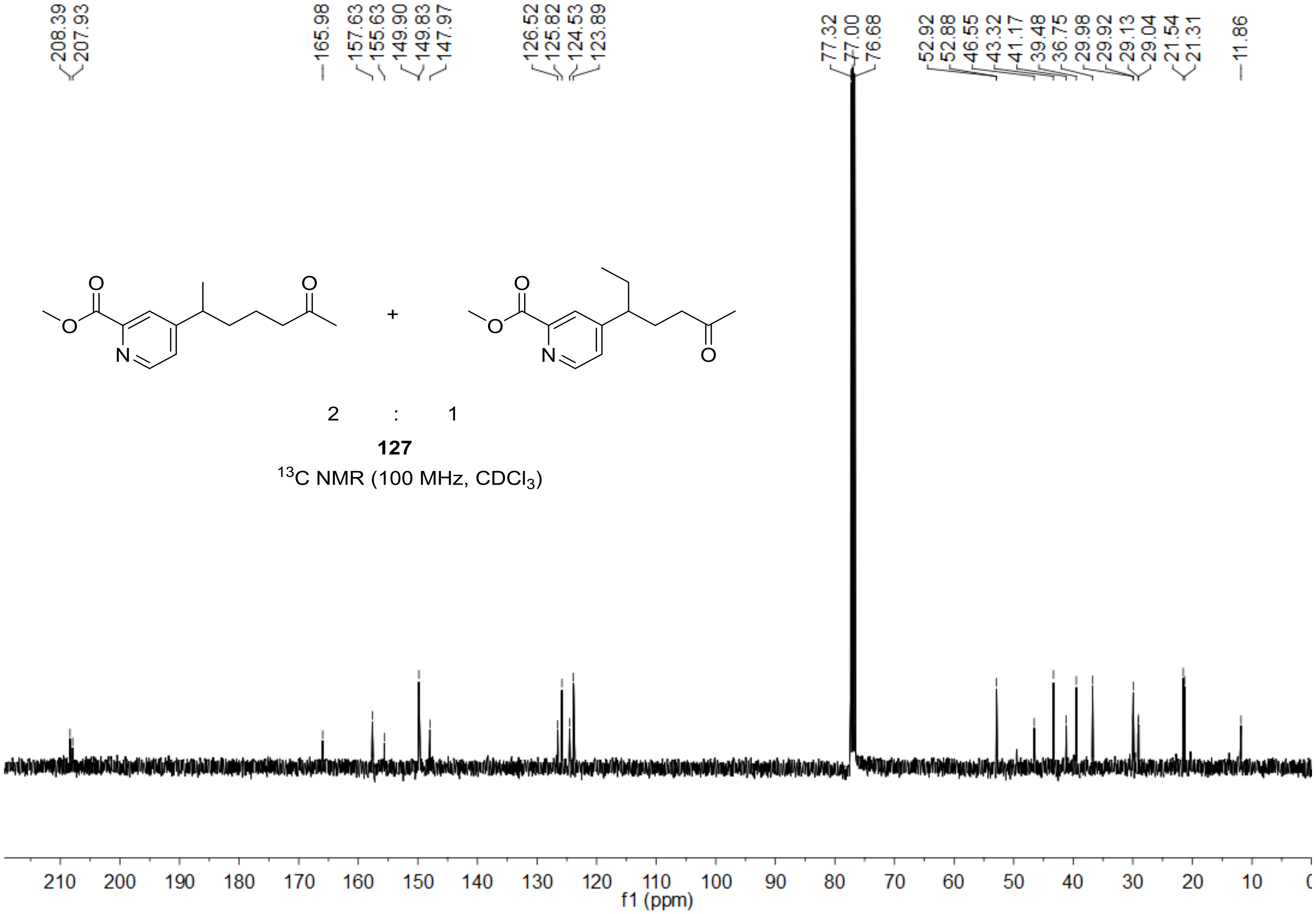

127

^{13}C NMR (100 MHz, $CDCl_3$)

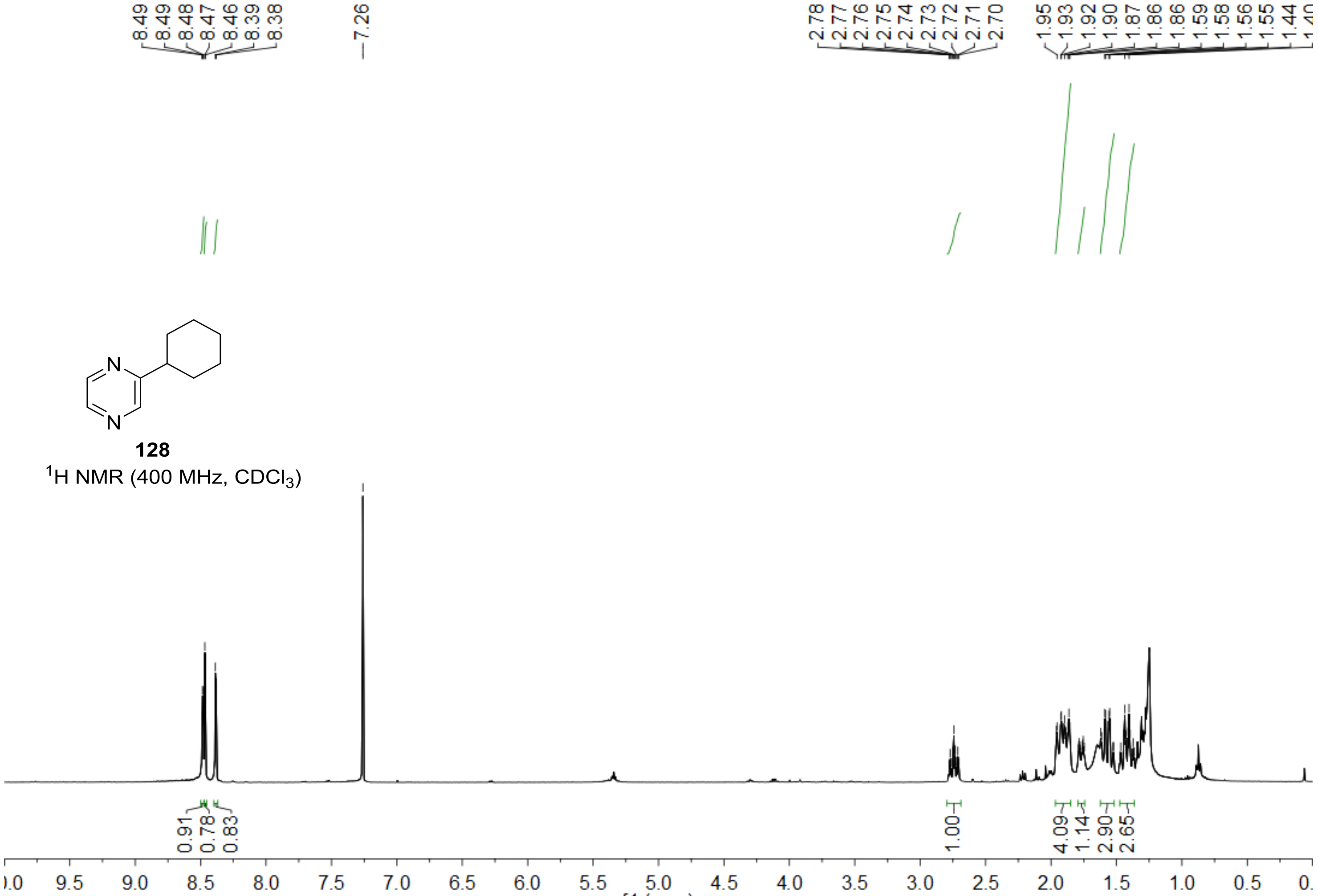

128

^1H NMR (400 MHz, $CDCl_3$)

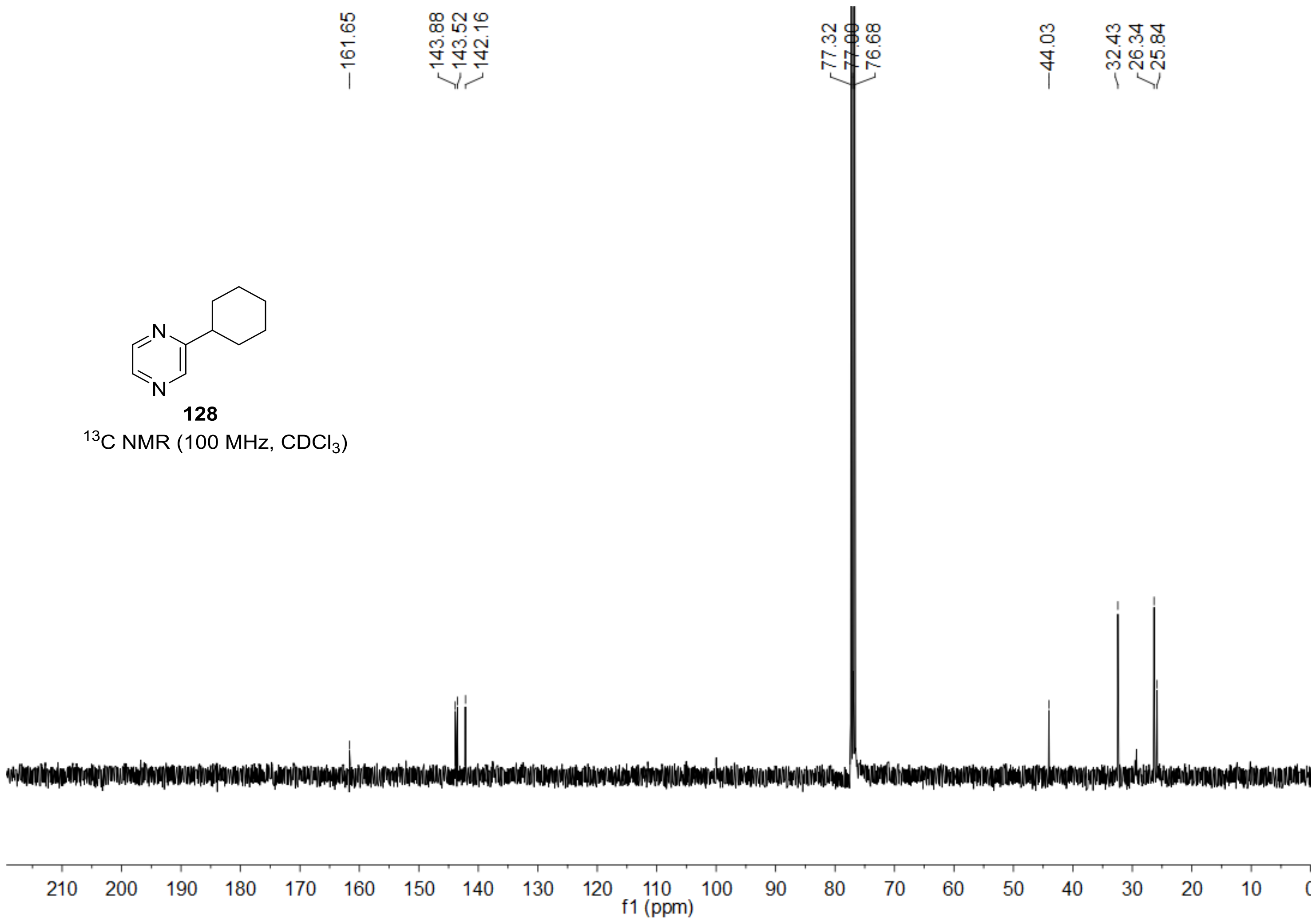
161.65
143.88
143.52
142.16
77.32
77.00
76.68
44.03
32.43
26.34
25.84
N
N
128
^{13}C NMR (100 MHz, $CDCl_3$)
210 200 190 180 170 160 150 140 130 120 110 100 90 80 70 60 50 40 30 20 10 0
f1 (ppm)

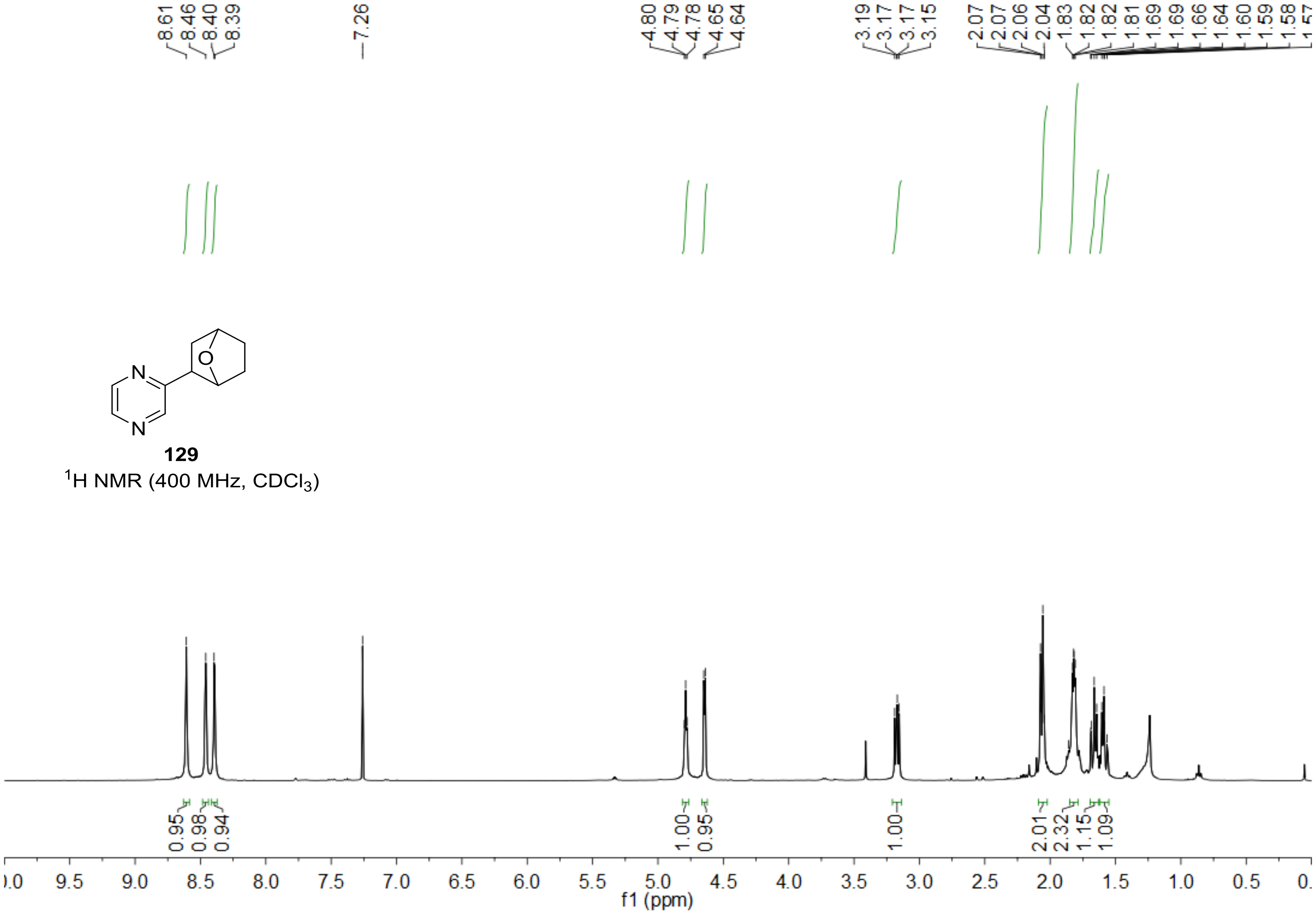

129

^{1}H NMR (400 MHz, $CDCl_3$)

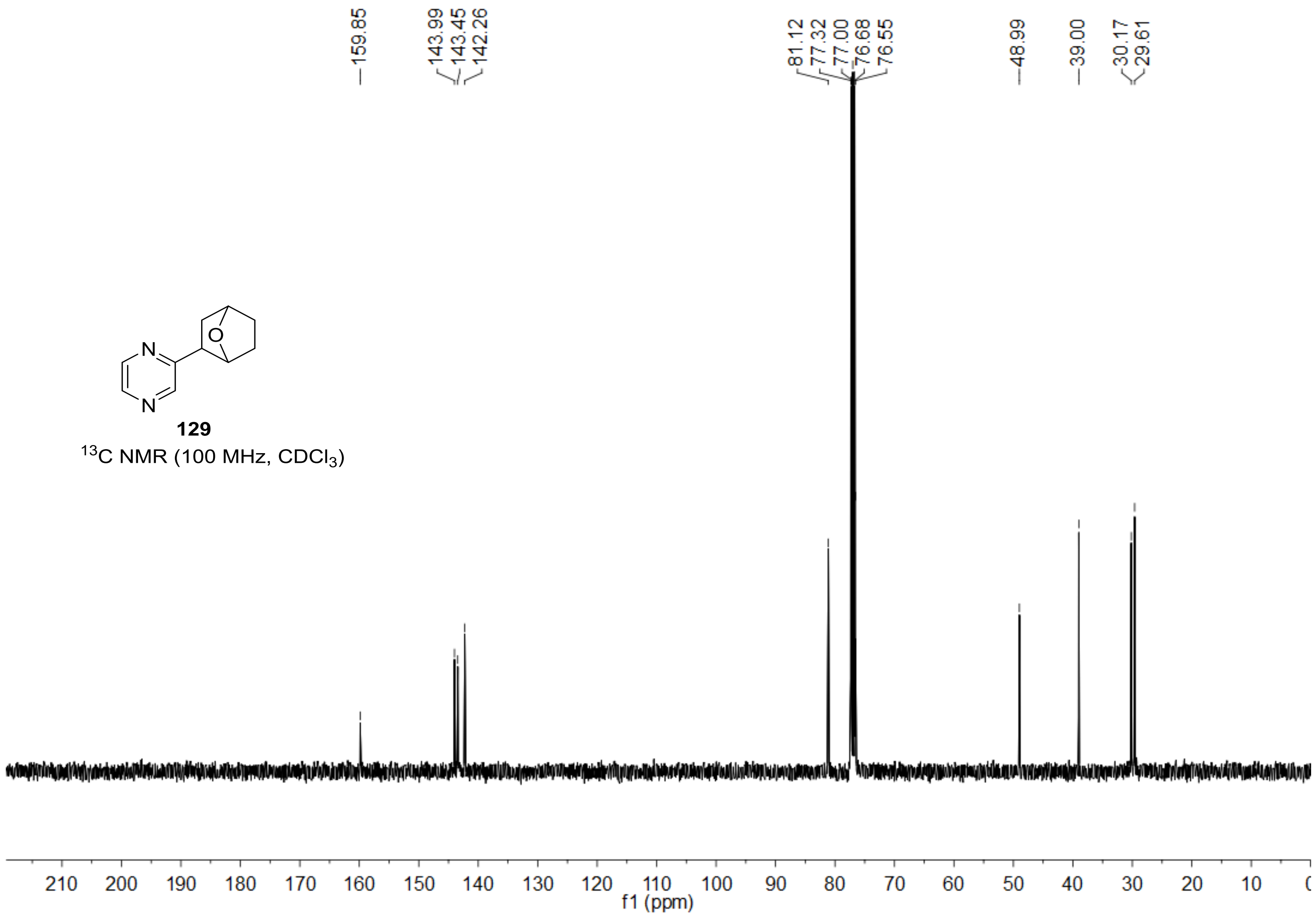
159.85
143.99
143.45
142.26
81.12
77.32
77.00
76.68
76.55
48.99
39.00
30.17
29.61
N
N
O
129
^{13}C NMR (100 MHz, $CDCl_3$)
210 200 190 180 170 160 150 140 130 120 110 100 90 80 70 60 50 40 30 20 10
f1 (ppm)

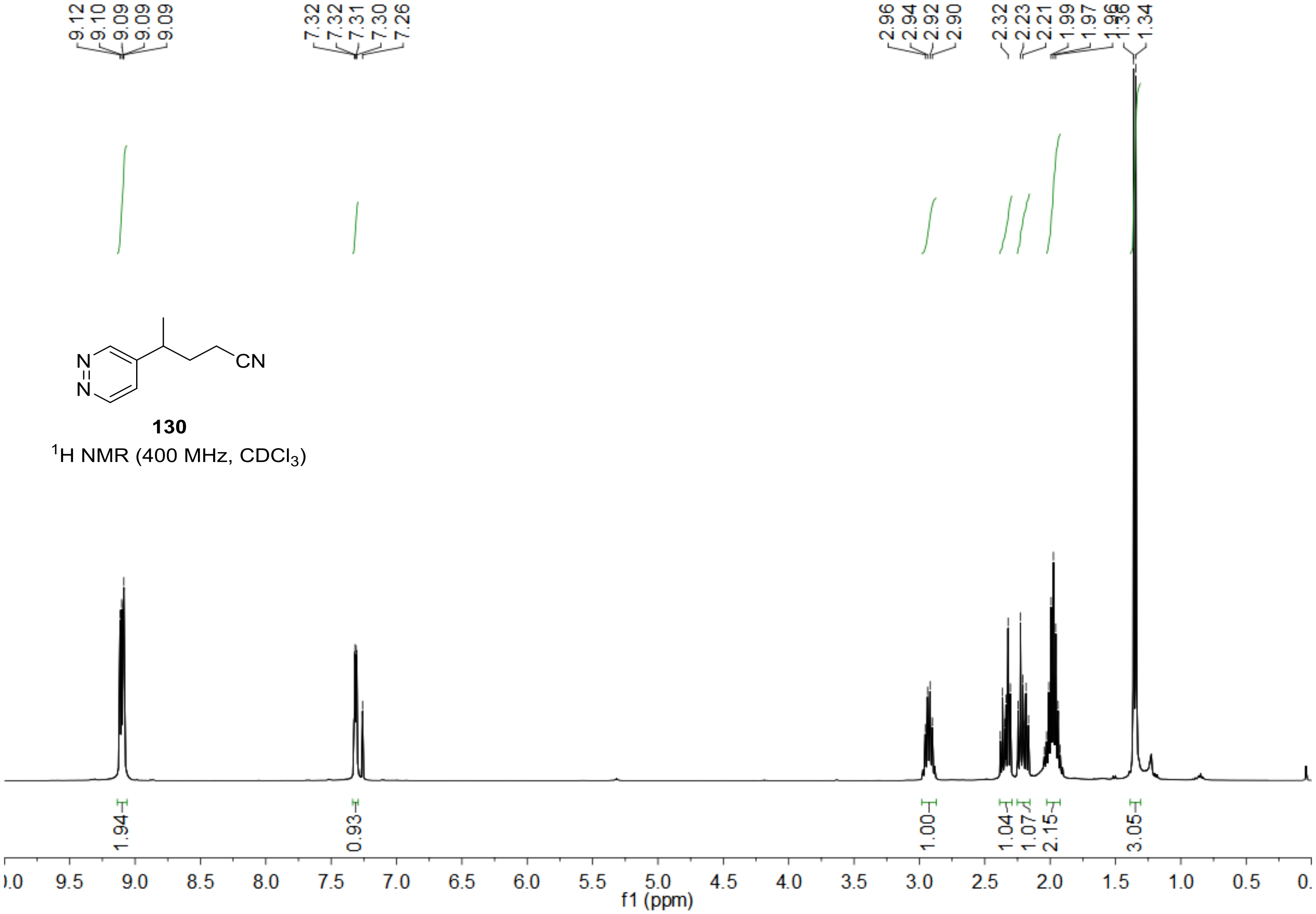

130

^{1}H NMR (400 MHz, $CDCl_3$)

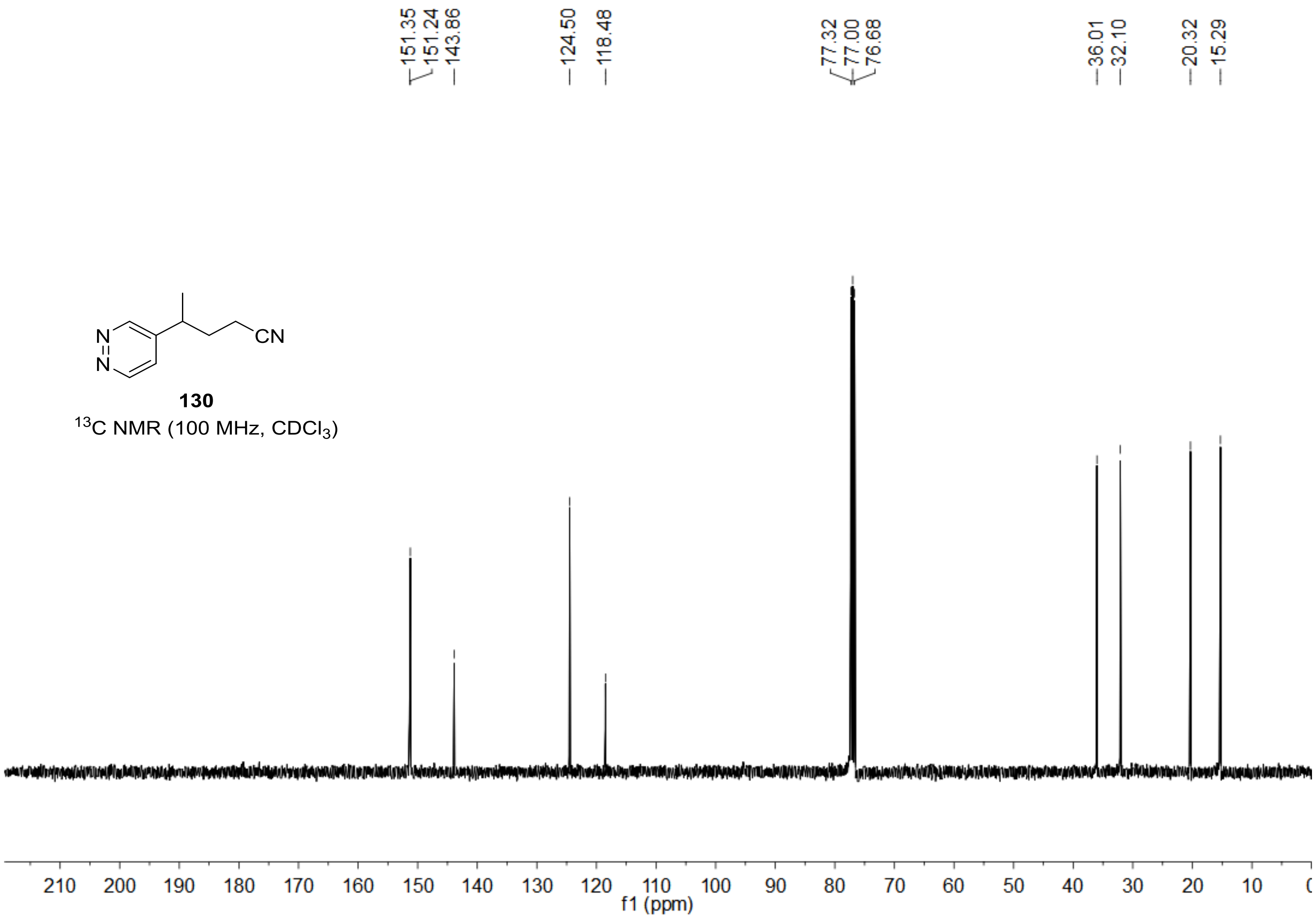

151.35
151.24
143.86
124.50
118.48
77.32
77.00
76.68
36.01
32.10
20.32
15.29
N
N
CN
130
^{13}C NMR (100 MHz, CDCl$_3$)
210 200 190 180 170 160 150 140 130 120 110 100 90 80 70 60 50 40 30 20 10
f1 (ppm)

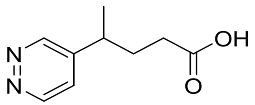

131

^{1}H NMR (400 MHz, $CDCl_3$)

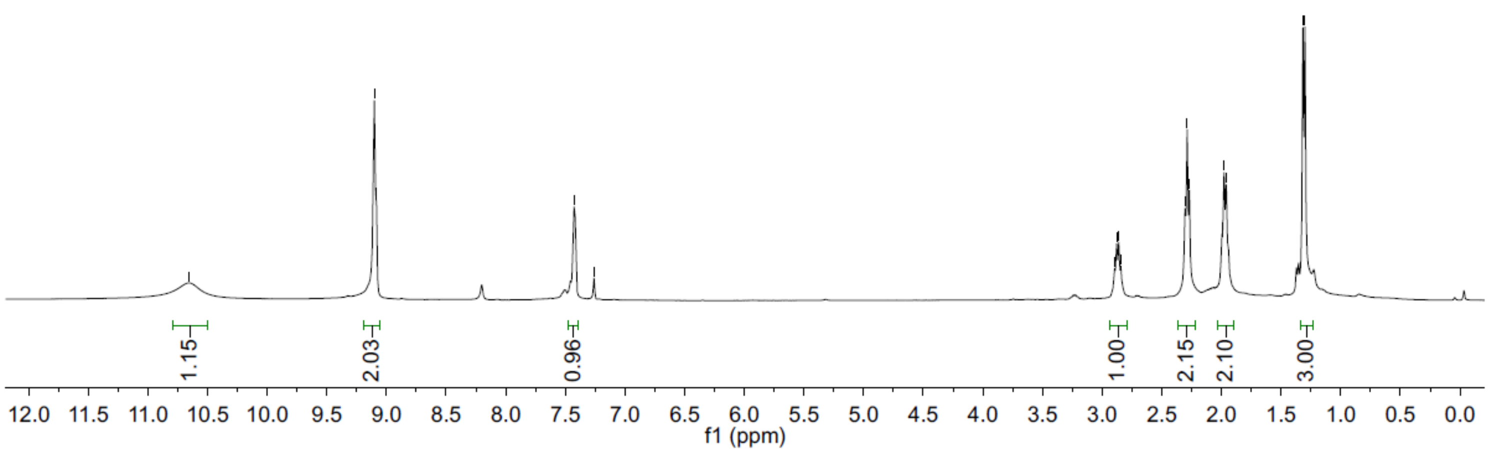

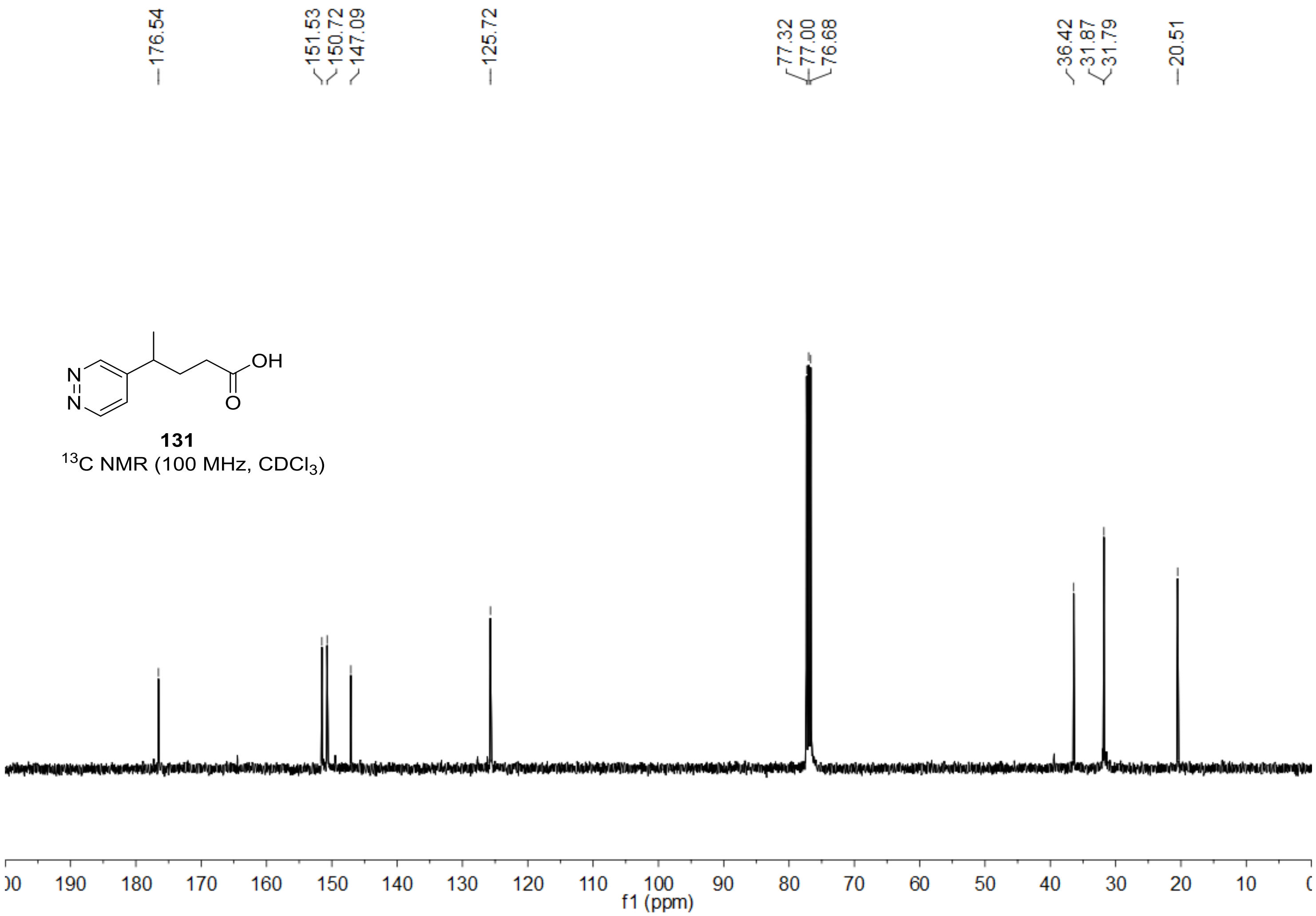
176.54
151.53
150.72
147.09
125.72
77.32
77.00
76.68
36.42
31.87
31.79
20.51
N
N
OH
O
131
^{13}C NMR (100 MHz, CDCl$_3$)
190
180
170
160
150
140
130
120
110
100
90
80
70
60
50
40
30
20
10
f1 (ppm)

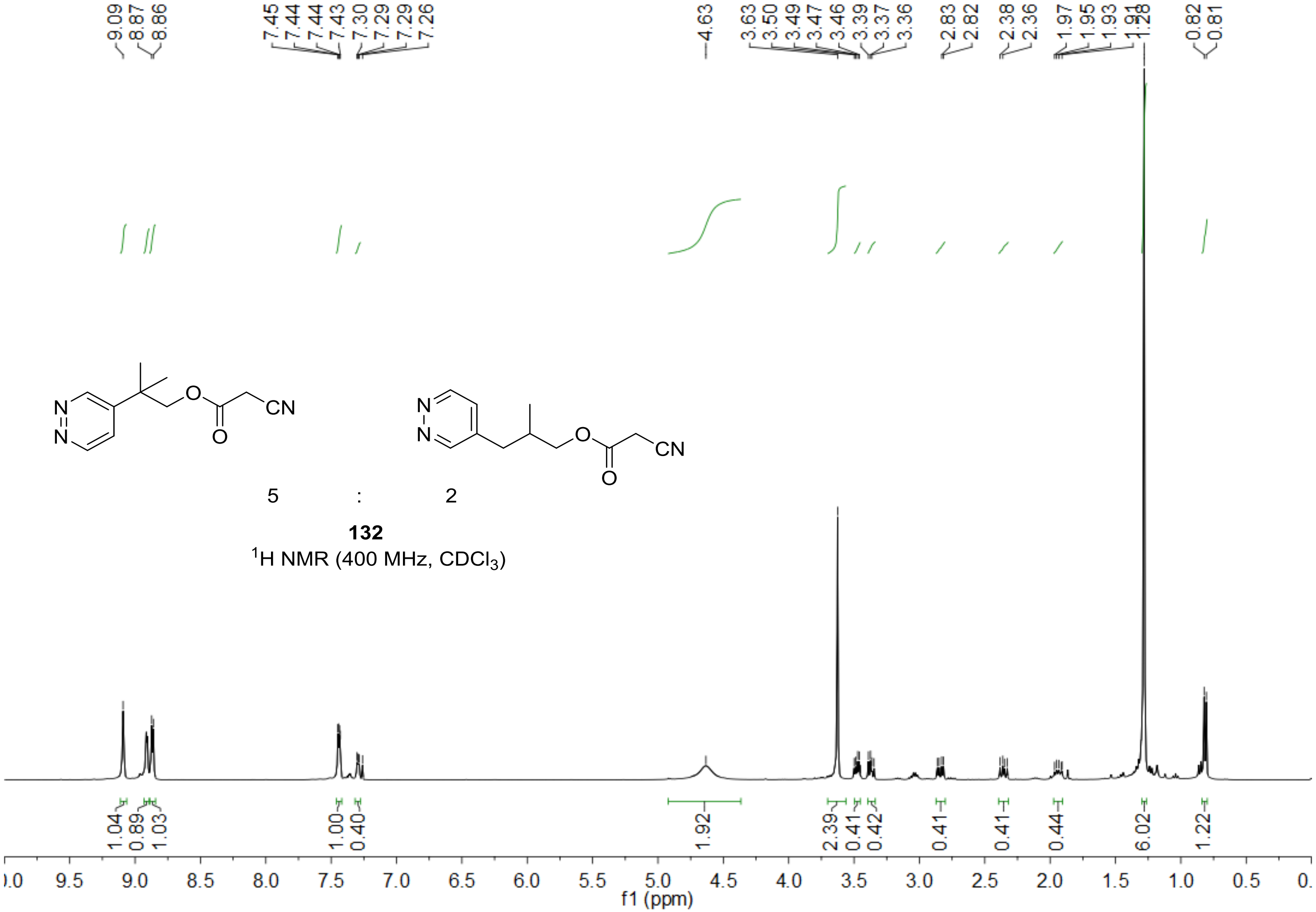

5 : 2
132
¹H NMR (400 MHz, CDCl₃)
f1 (ppm)

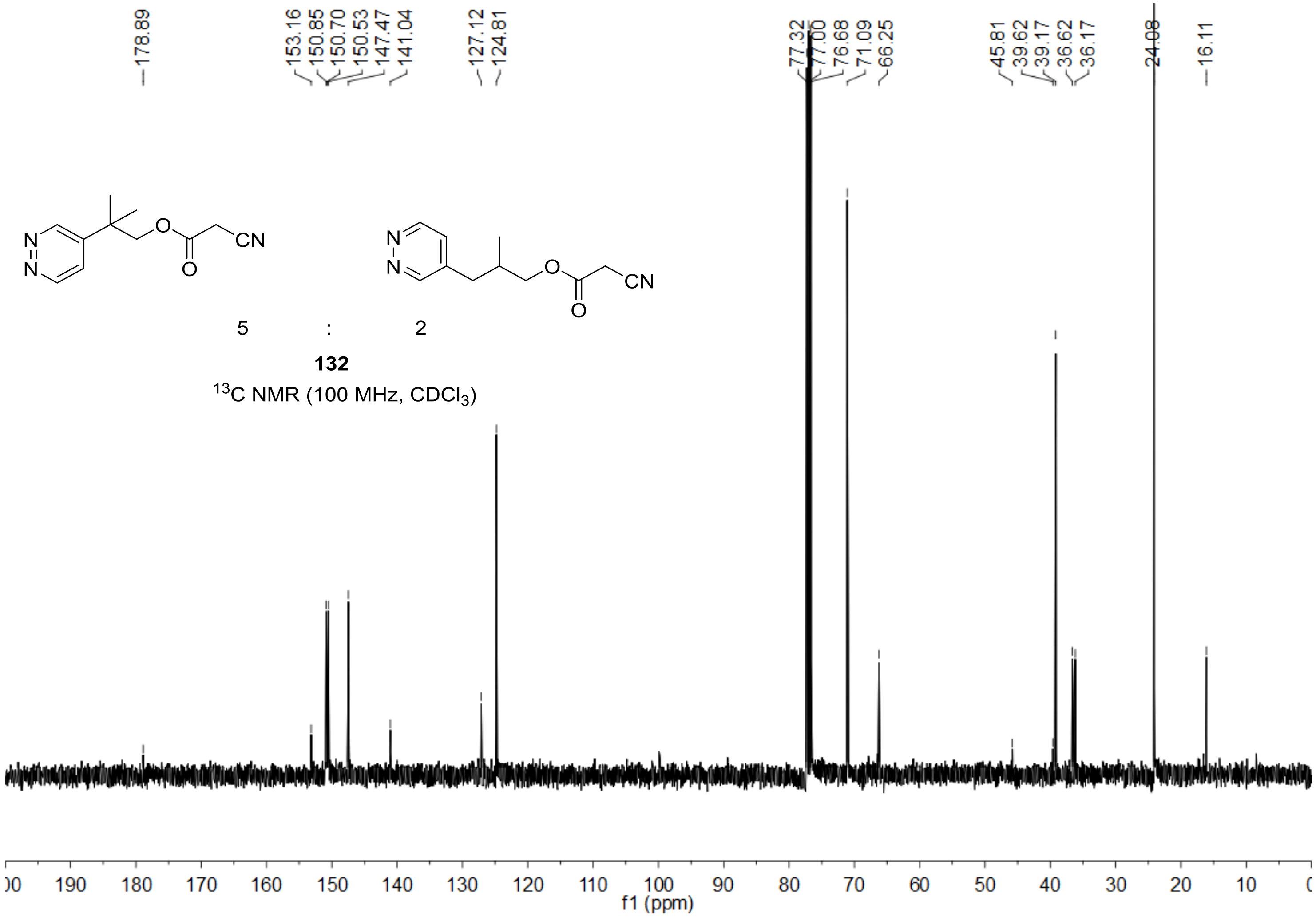
178.89
153.16
150.85
150.70
150.53
147.47
141.04
127.12
124.81
77.32
77.00
76.68
71.09
66.25
45.81
39.62
39.17
36.62
36.17
24.08
16.11
CN
O
N
5 : 2
132
^{13}C NMR (100 MHz, CDCl$_3$)
f1 (ppm)

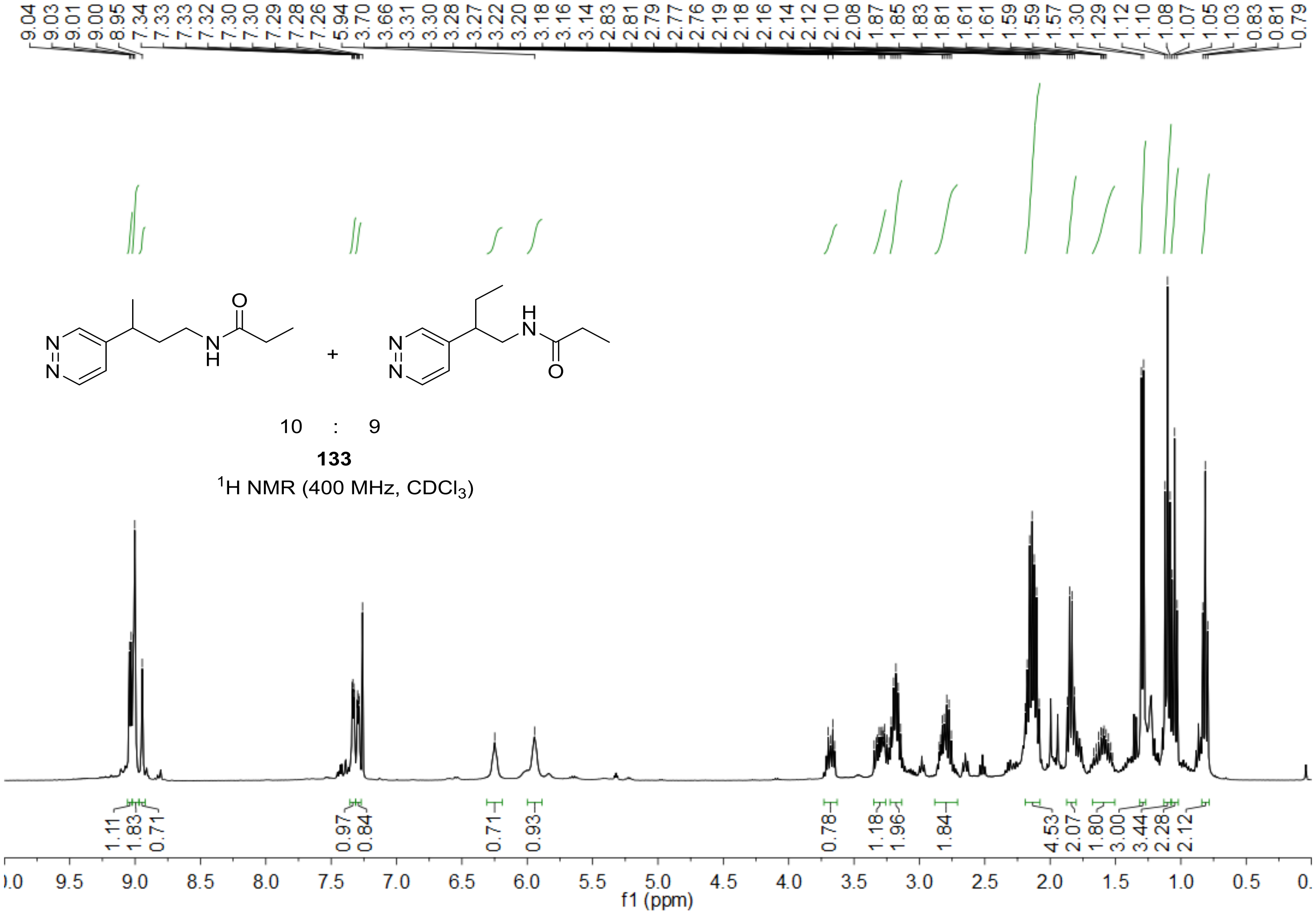
10 : 9
133
^{1}H NMR (400 MHz, $CDCl_3$)
f1 (ppm)

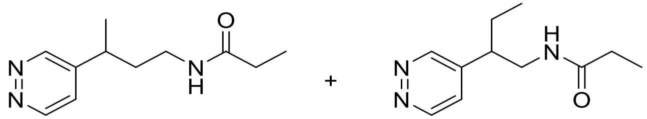

10 : 9

133

^{13}C NMR (100 MHz, $CDCl_3$)

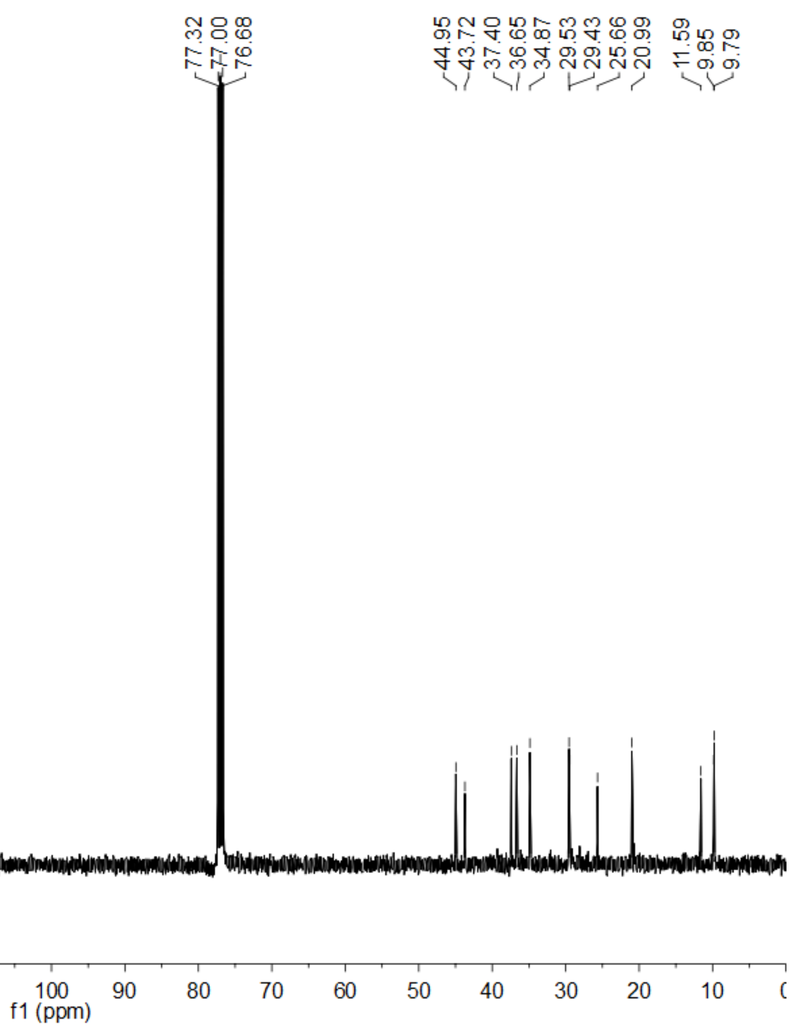

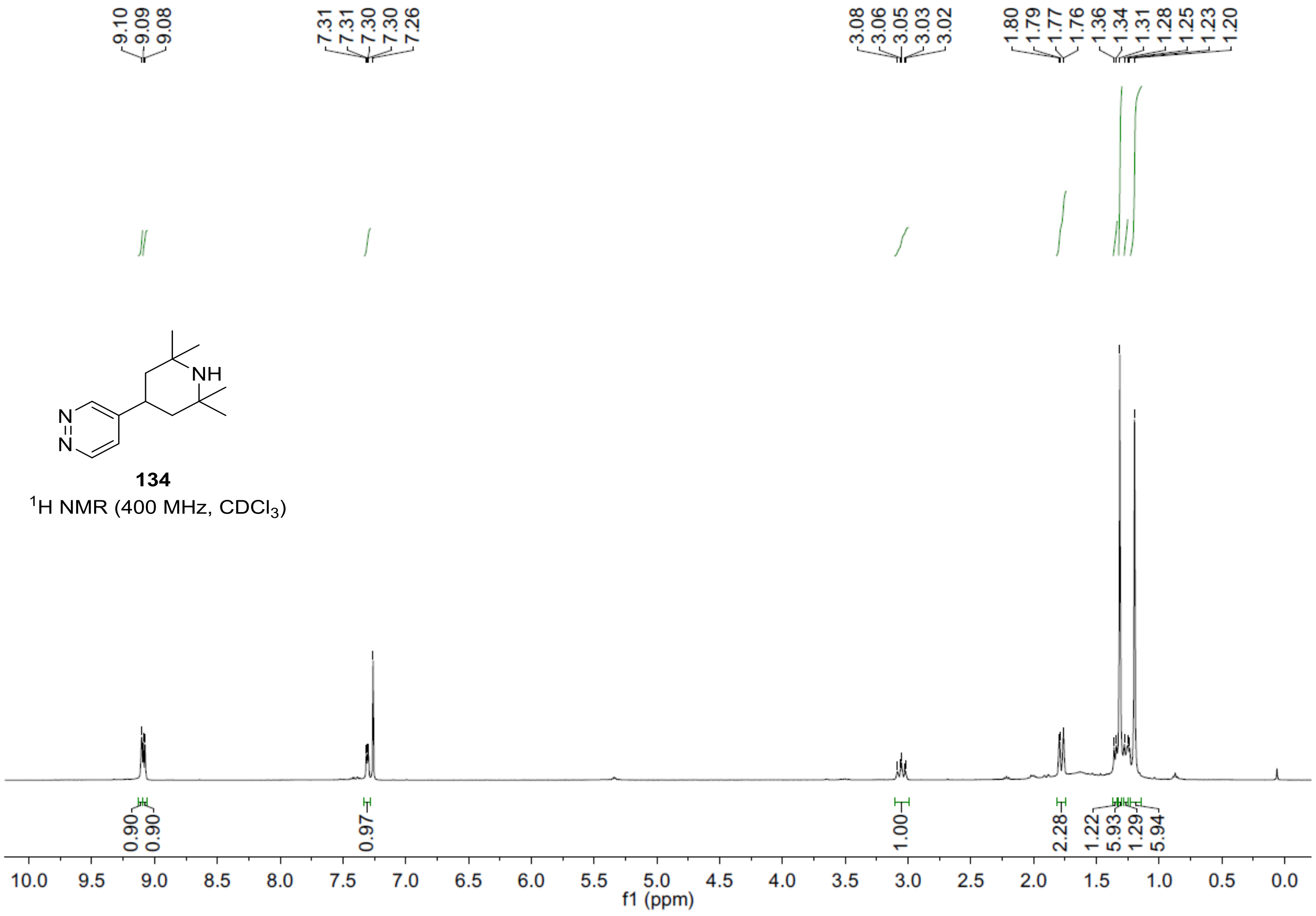

134

^{1}H NMR (400 MHz, $CDCl_3$)

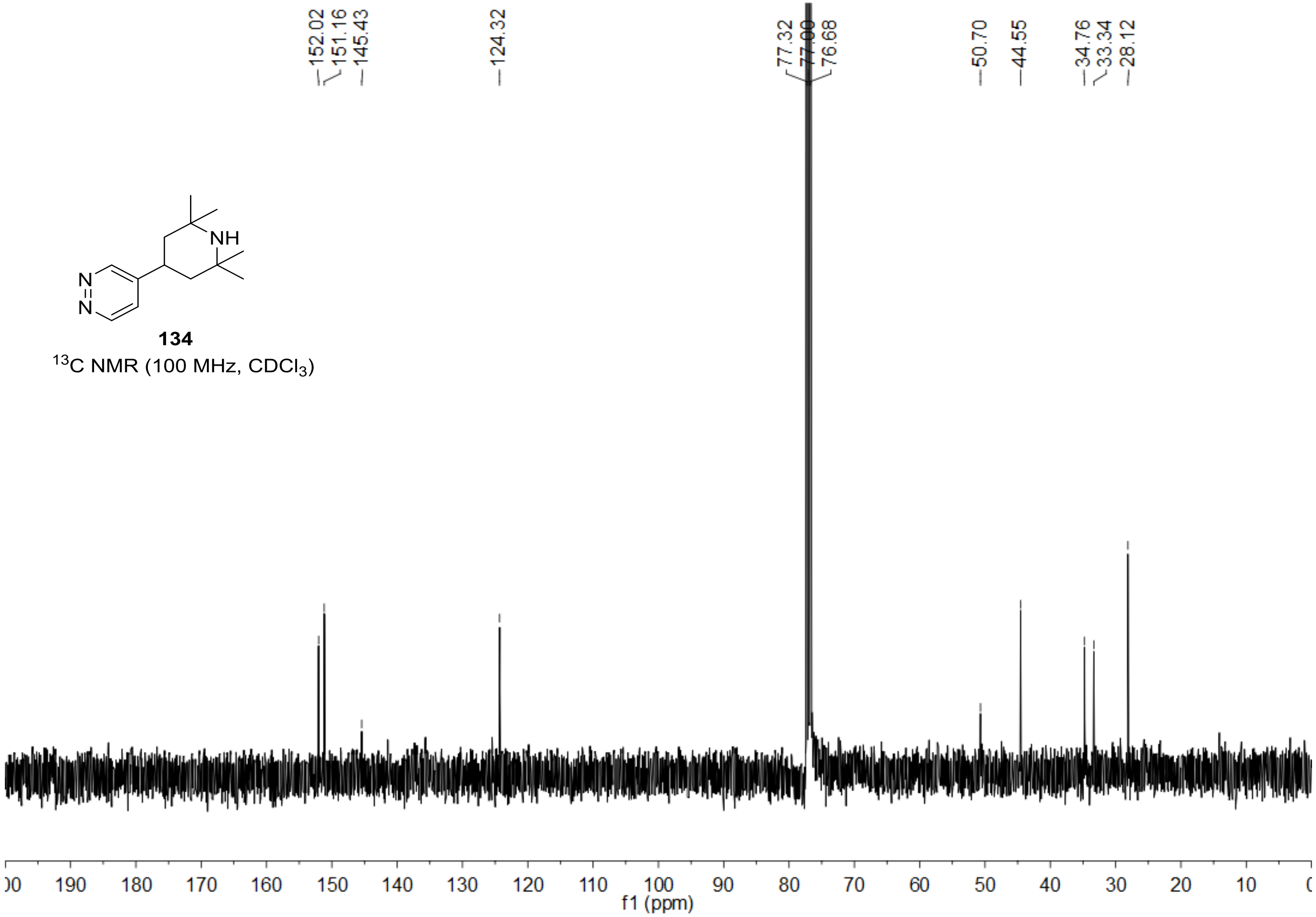
152.02
151.16
145.43
124.32
77.32
77.00
76.68
50.70
44.55
34.76
33.34
28.12
NH
N
N
134
^{13}C NMR (100 MHz, $CDCl_3$)
f1 (ppm)

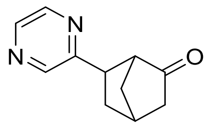

135

^{1}H NMR (400 MHz, $CDCl_3$)

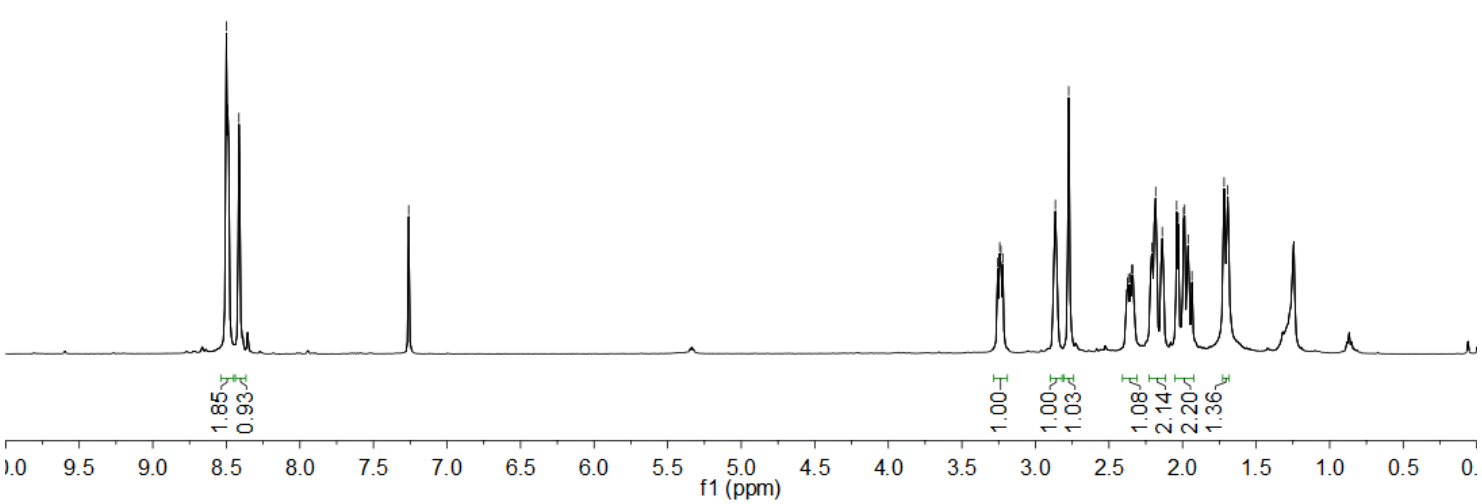

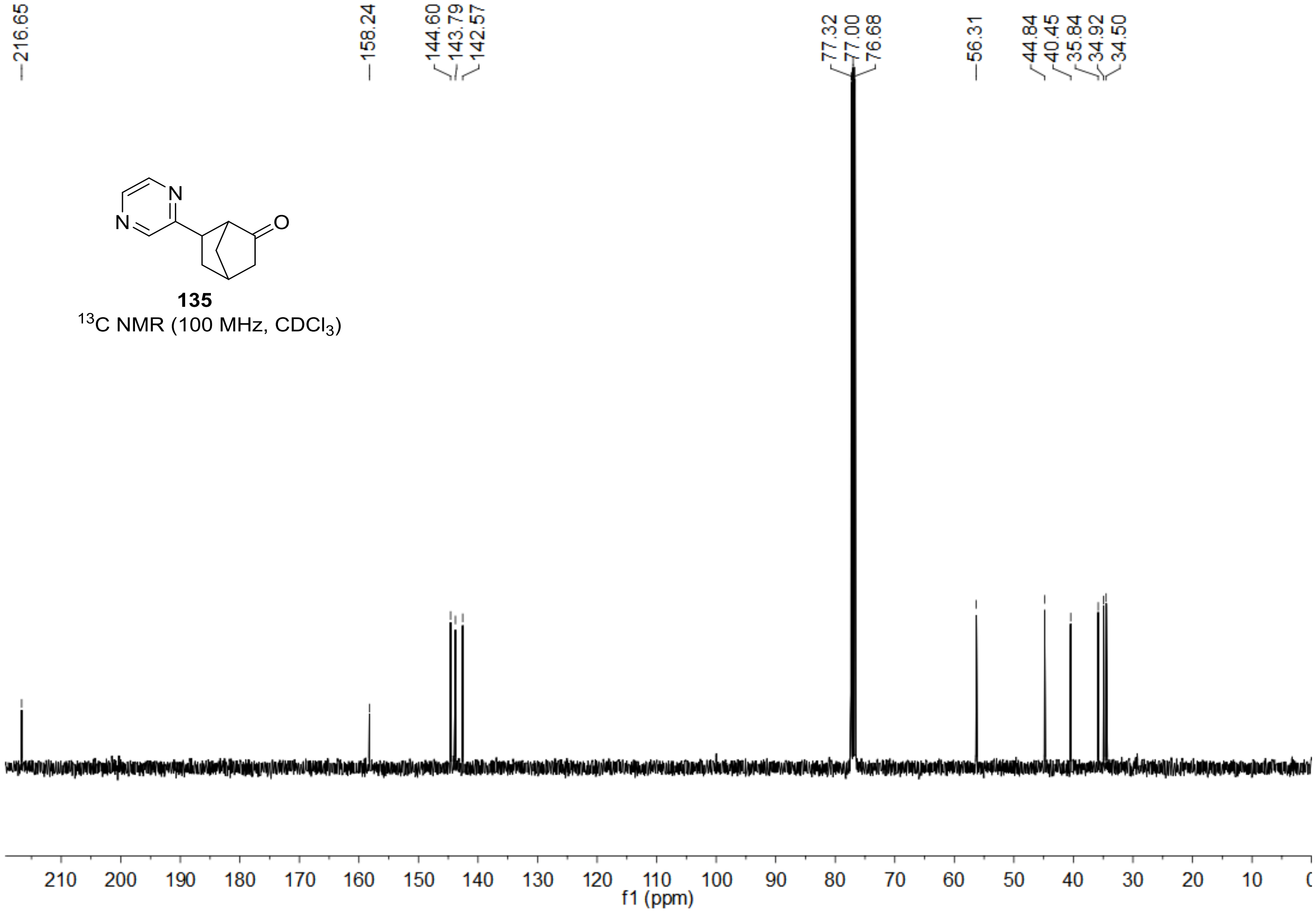

135

^{13}C NMR (100 MHz, $CDCl_3$)

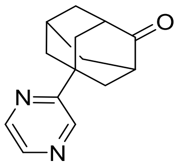

136

^{1}H NMR (400 MHz, $CDCl_3$)

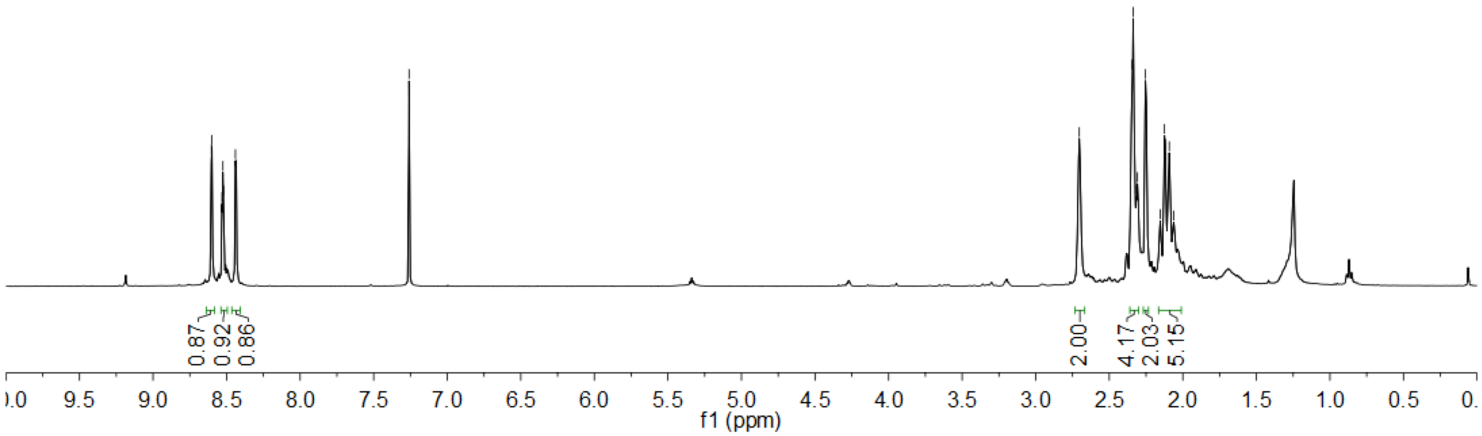

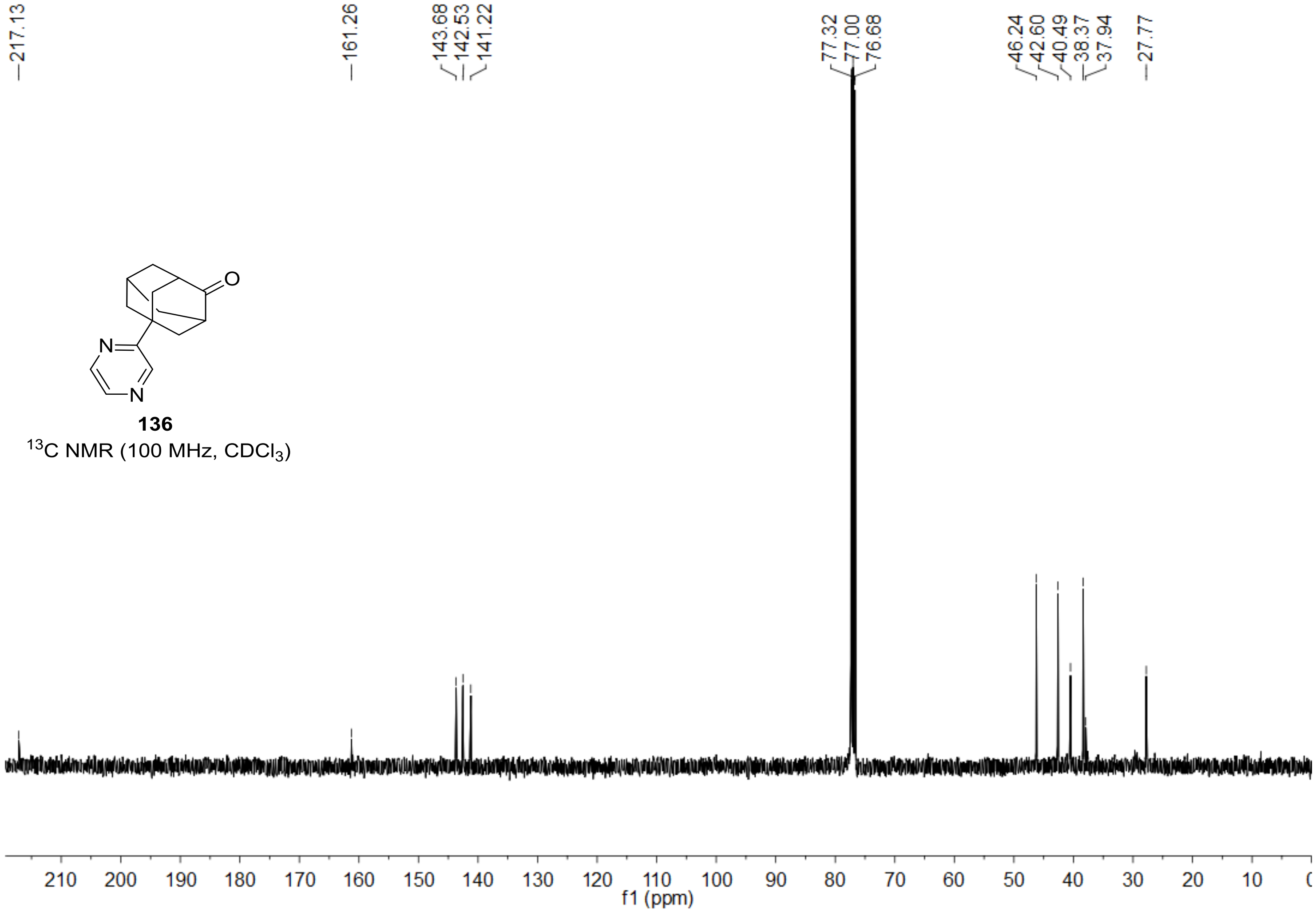

136

^{13}C NMR (100 MHz, $CDCl_3$)

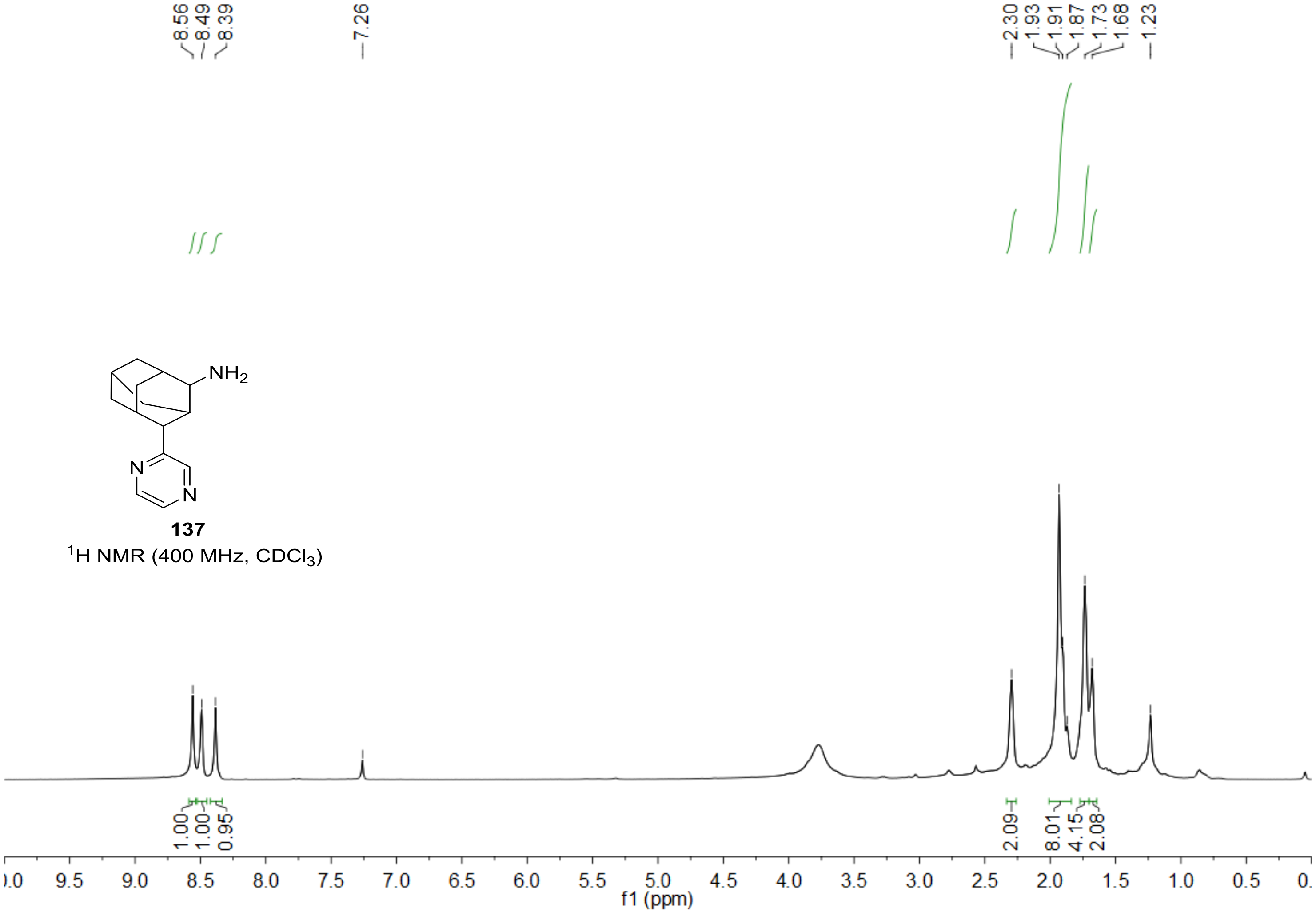

137

^{1}H NMR (400 MHz, $CDCl_3$)

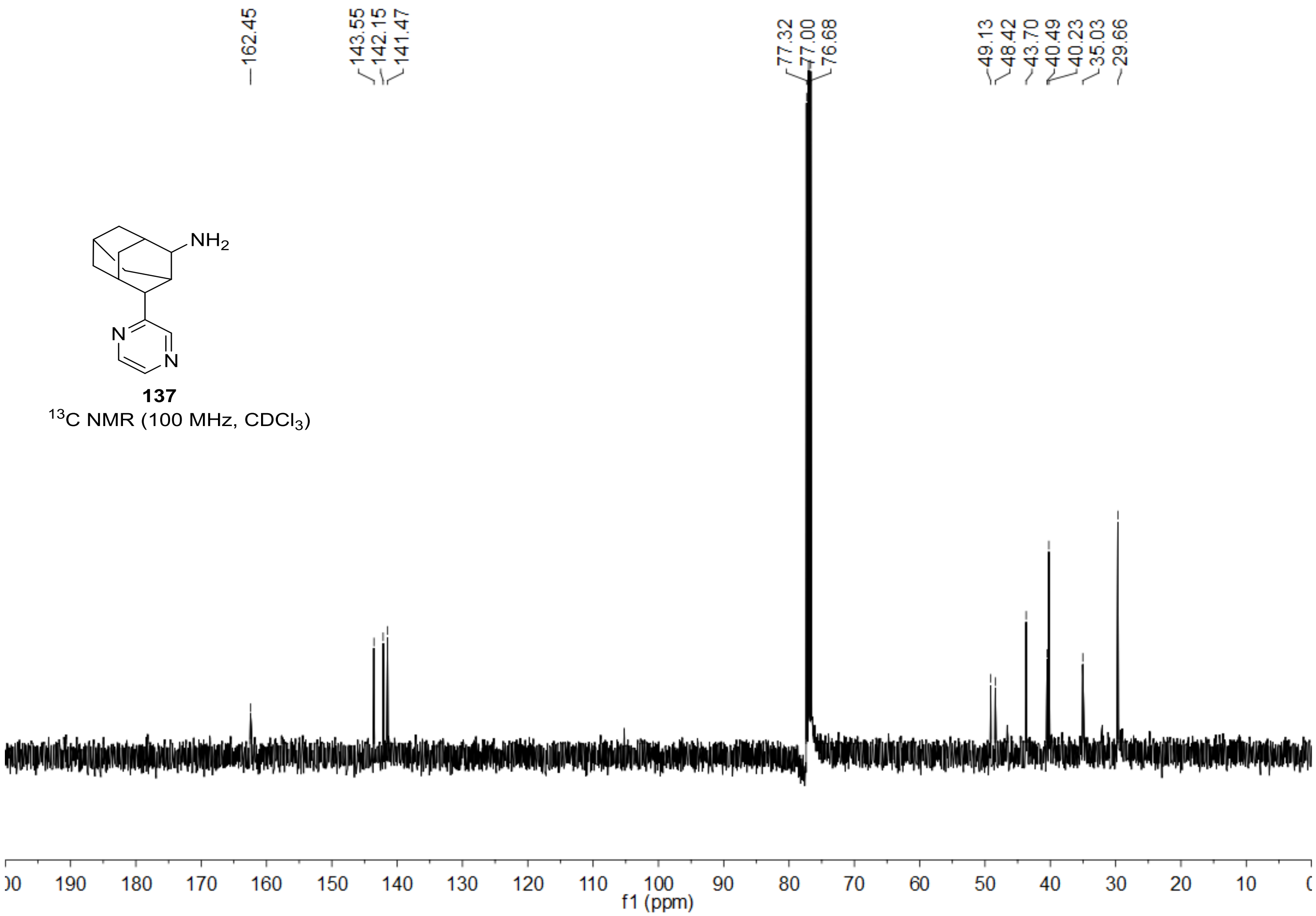
162.45
143.55
142.15
141.47
77.32
77.00
76.68
49.13
48.42
43.70
40.49
40.23
35.03
29.66
NH2
N
N
137
13C NMR (100 MHz, CDCl3)
190
180
170
160
150
140
130
120
110
100
90
80
70
60
50
40
30
20
10
f1 (ppm)

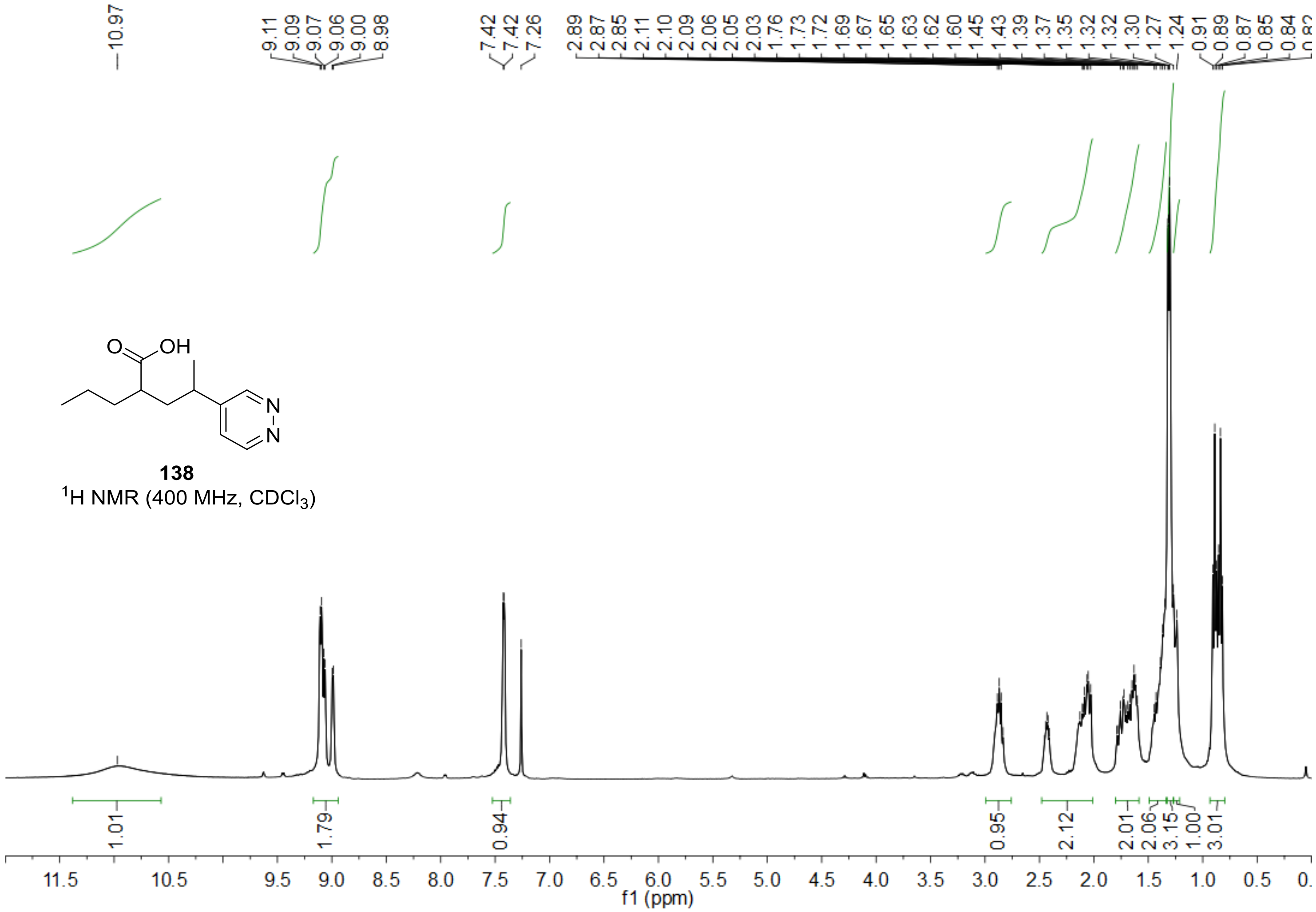
O OH
N
N
138
^{1}H NMR (400 MHz, CDCl$_3$)
f1 (ppm)

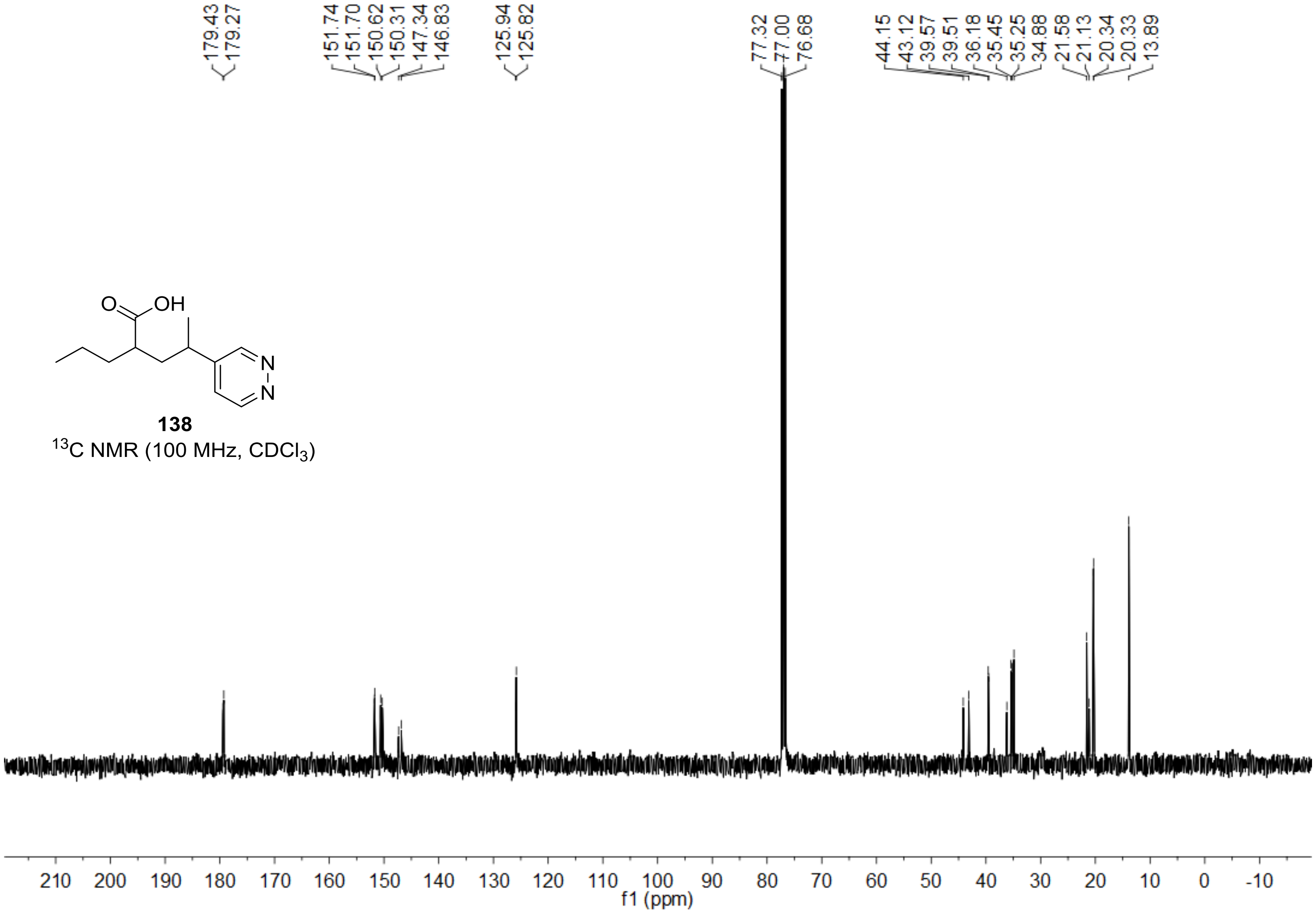

138

^{13}C NMR (100 MHz, $CDCl_3$)

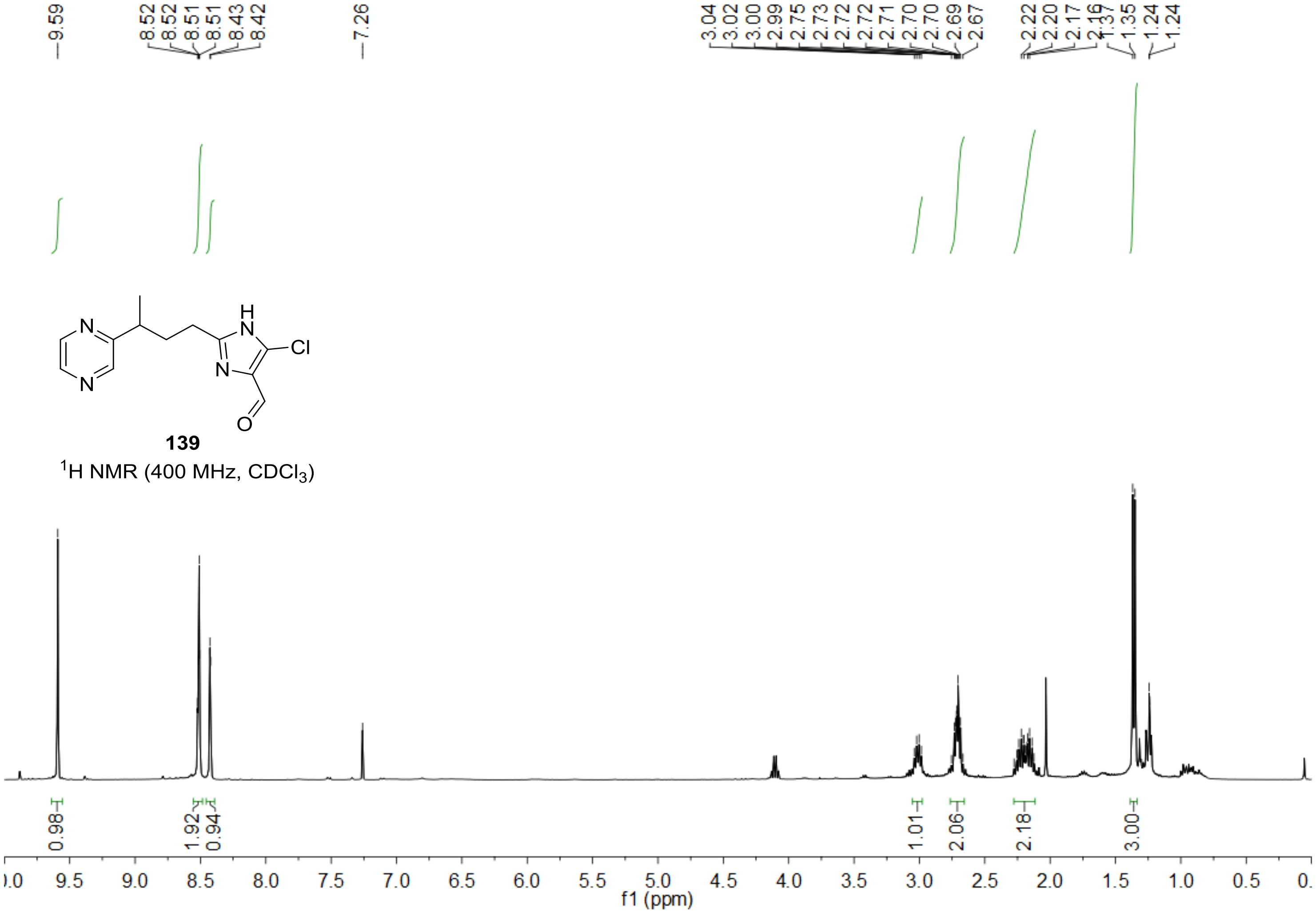

139

^{1}H NMR (400 MHz, $CDCl_3$)

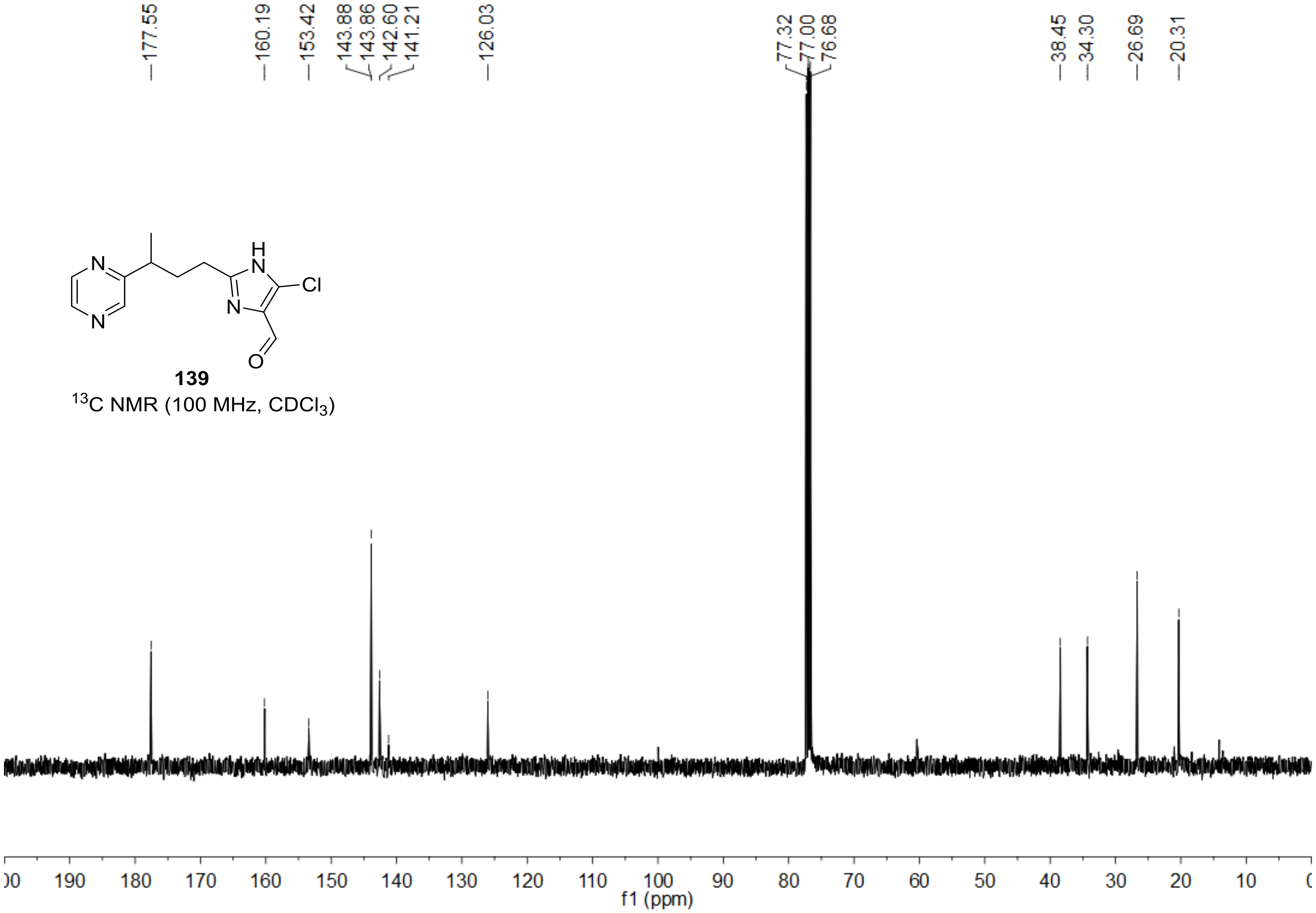

139

^{13}C NMR (100 MHz, $CDCl_3$)

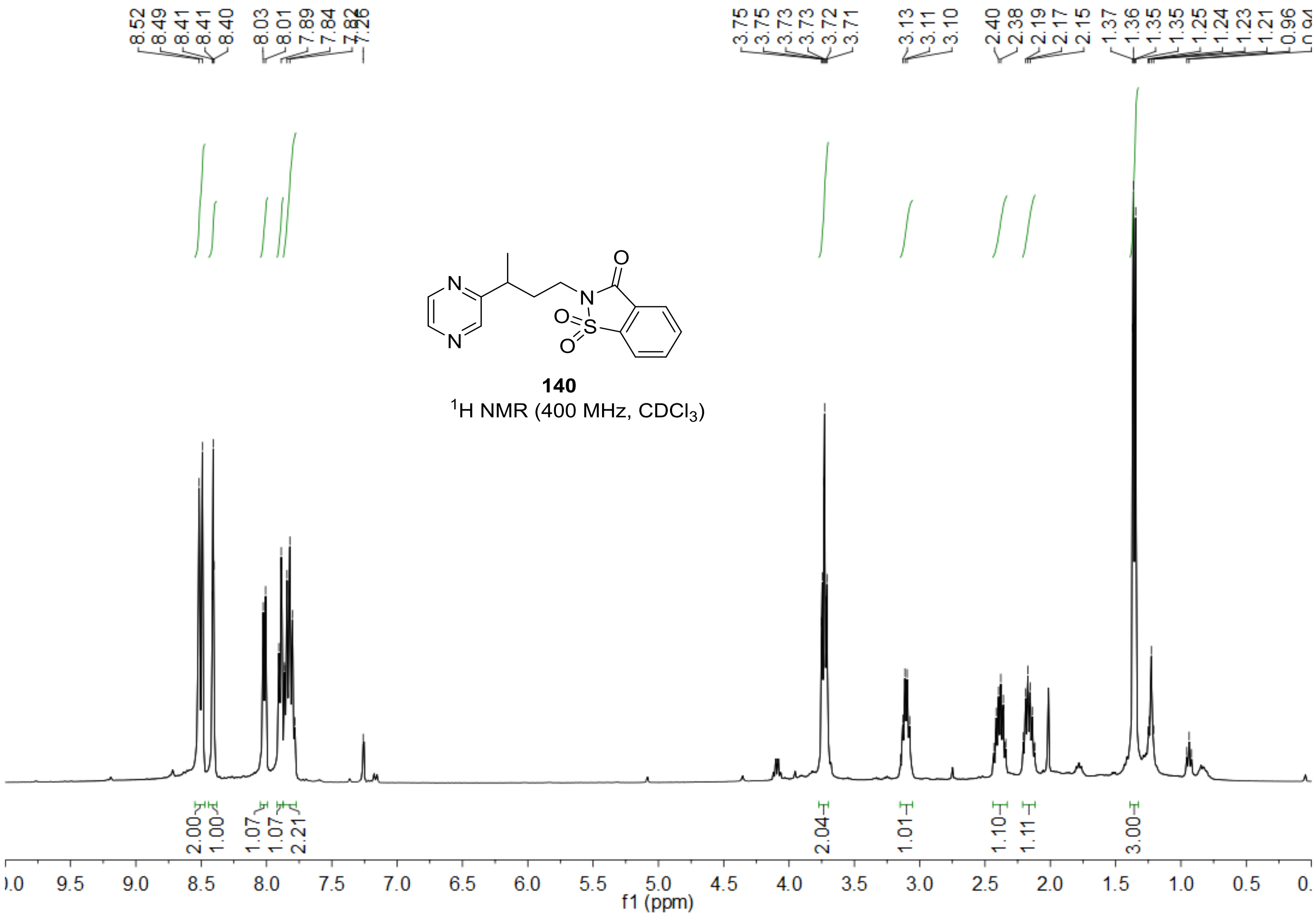

140

^{1}H NMR (400 MHz, $CDCl_3$)

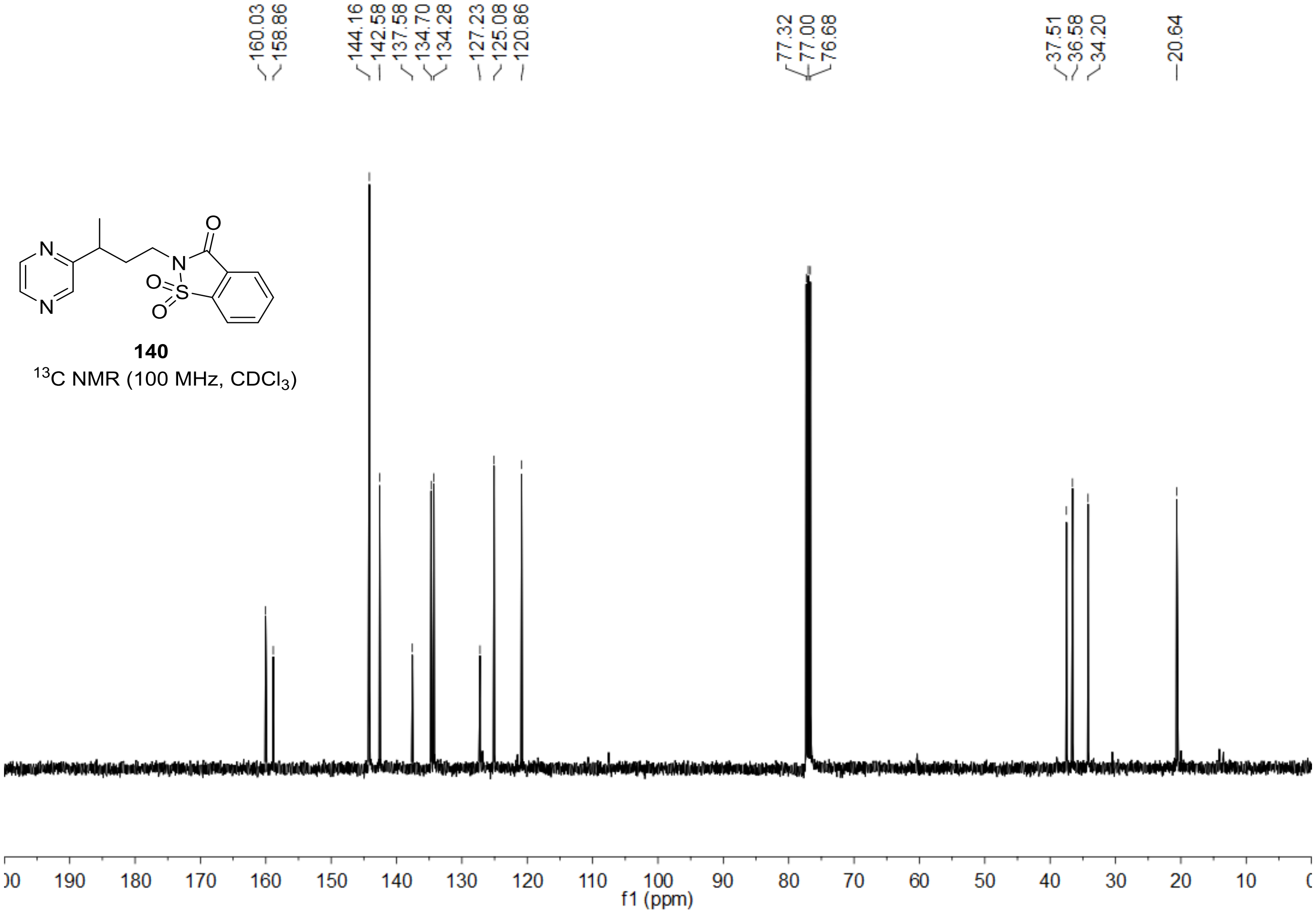

140

^{13}C NMR (100 MHz, $CDCl_3$)

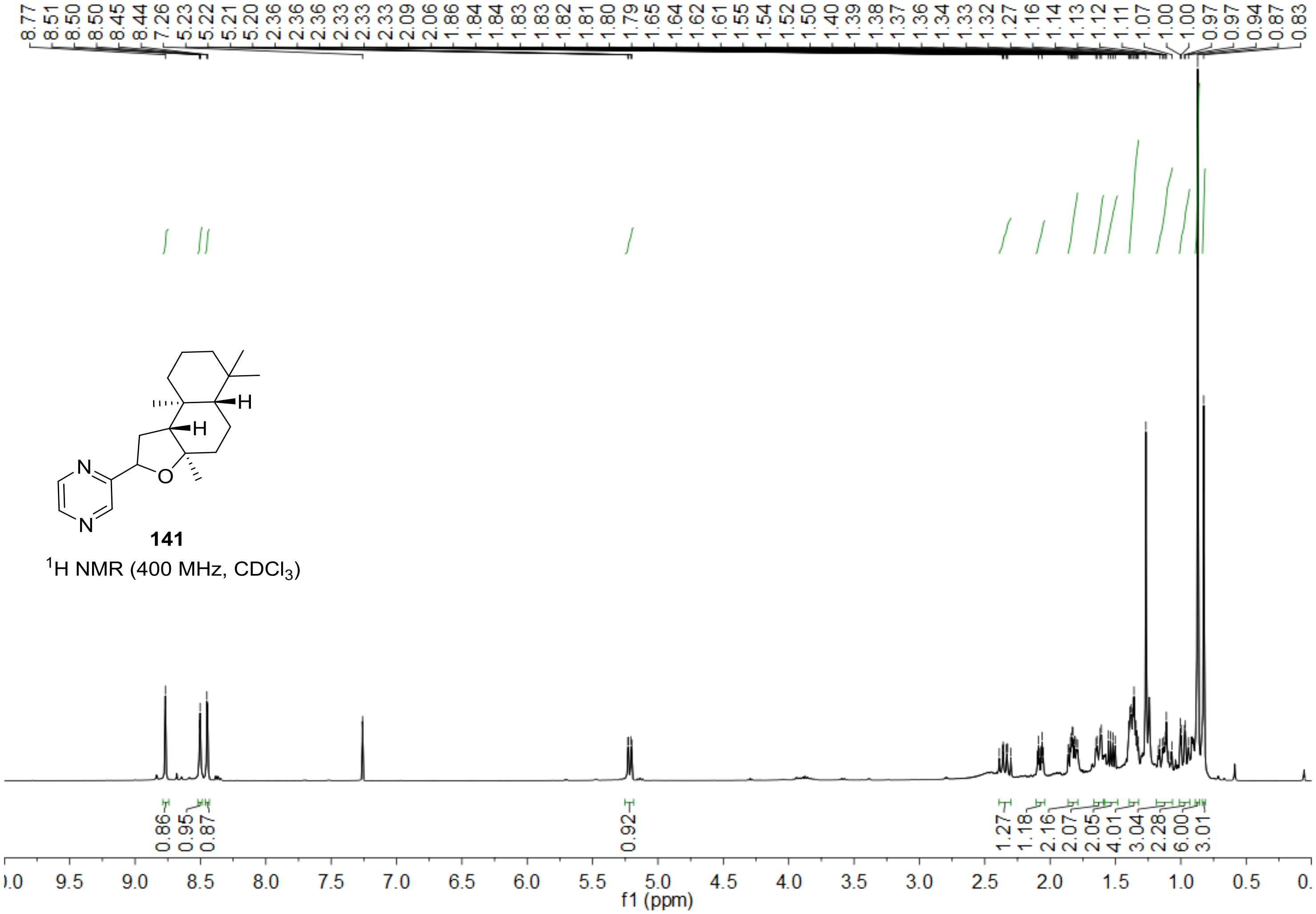
141
1H NMR (400 MHz, CDCl3)
f1 (ppm)

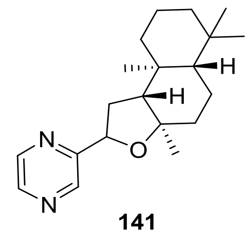

141

^{13}C NMR (100 MHz, $CDCl_3$)

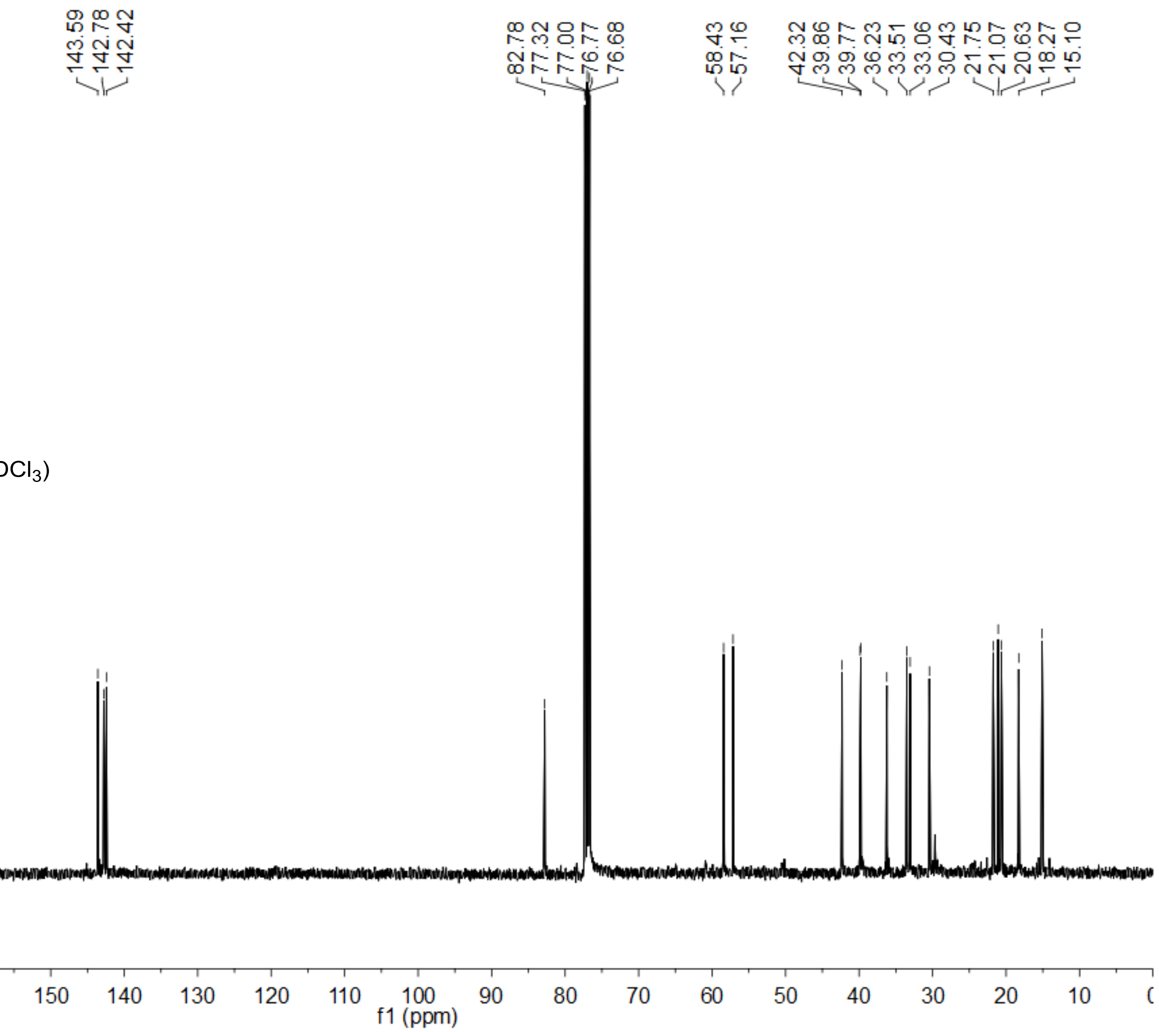

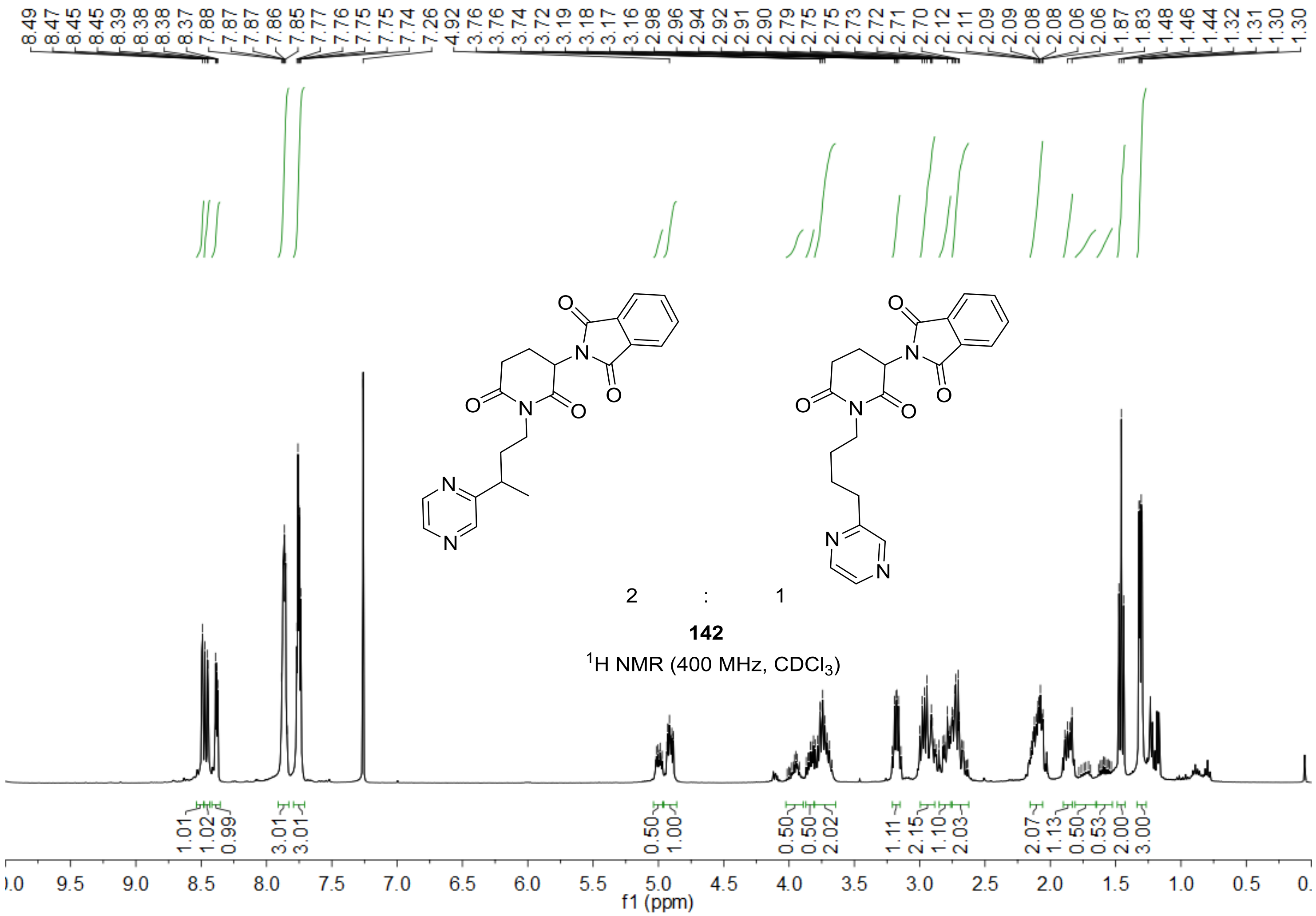
2 : 1
142
1H NMR (400 MHz, CDCl3)
f1 (ppm)

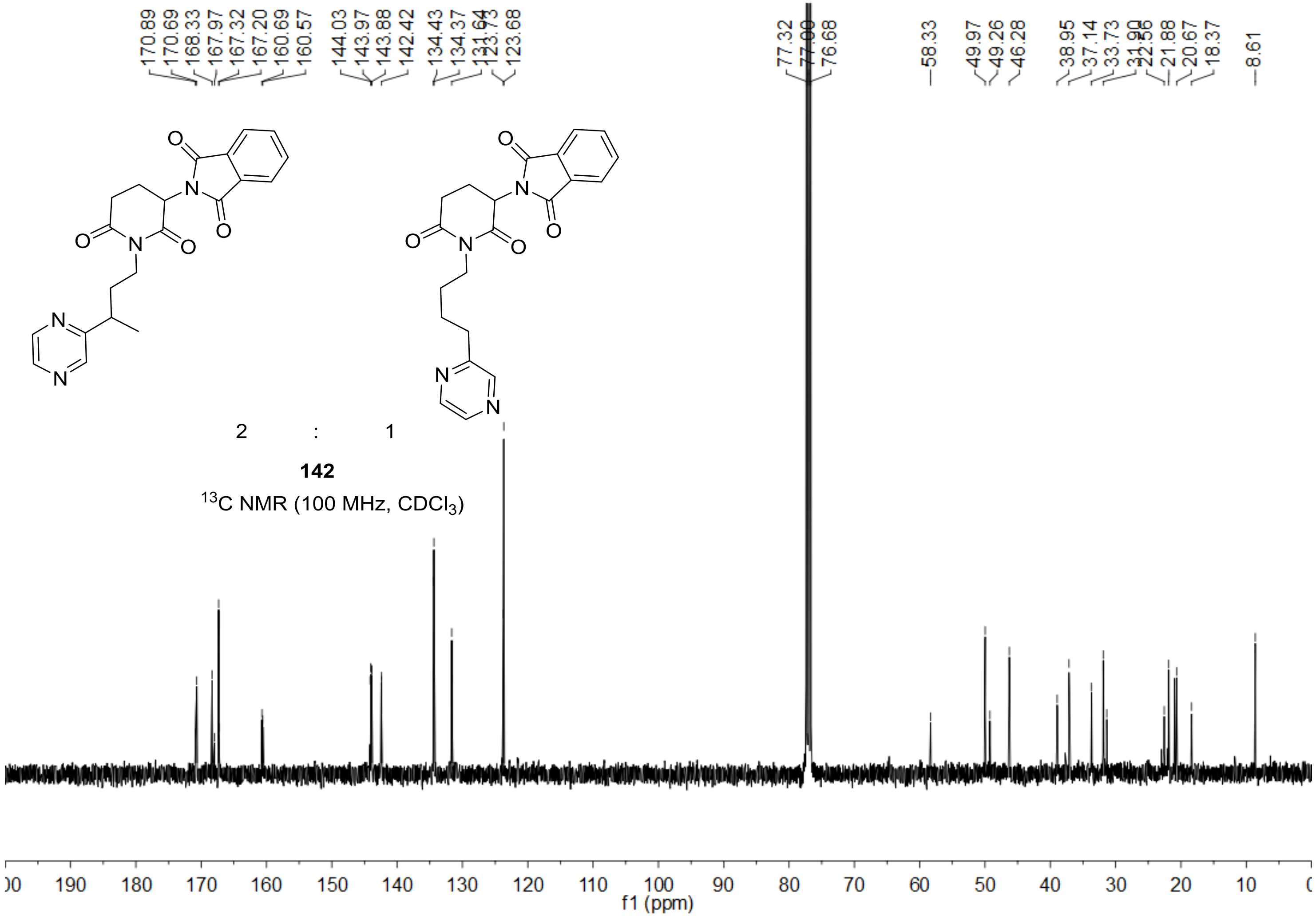
170.89
170.69
168.33
167.97
167.32
167.20
160.69
160.57
144.03
143.97
143.88
142.42
134.43
134.37
131.64
123.73
123.68
77.32
77.00
76.68
58.33
49.97
49.26
46.28
38.95
37.14
33.73
31.90
22.56
21.88
20.67
18.37
8.61
2 : 1
142
^{13}C NMR (100 MHz, CDCl$_3$)
f1 (ppm)

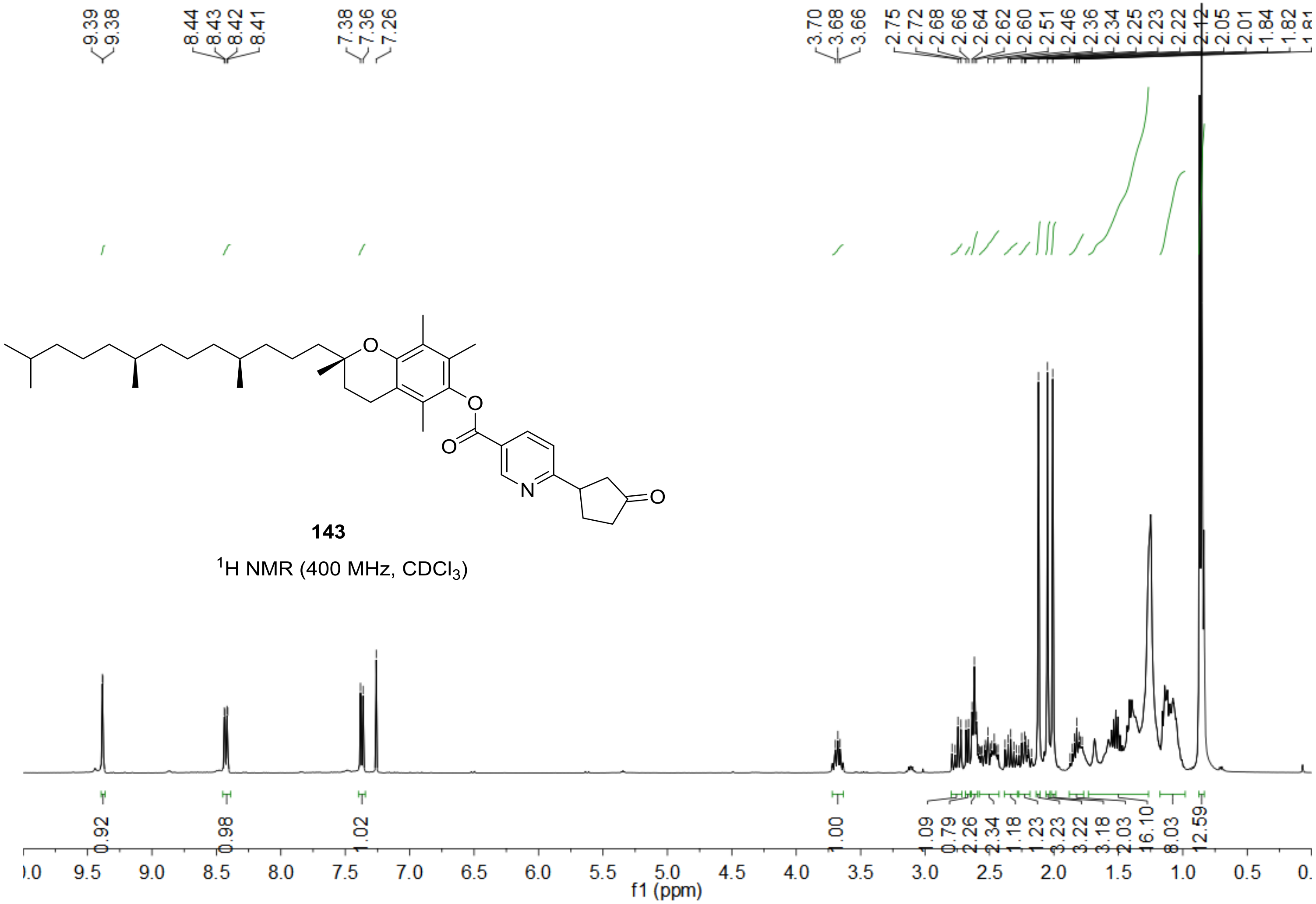

143

^{1}H NMR (400 MHz, $CDCl_3$)

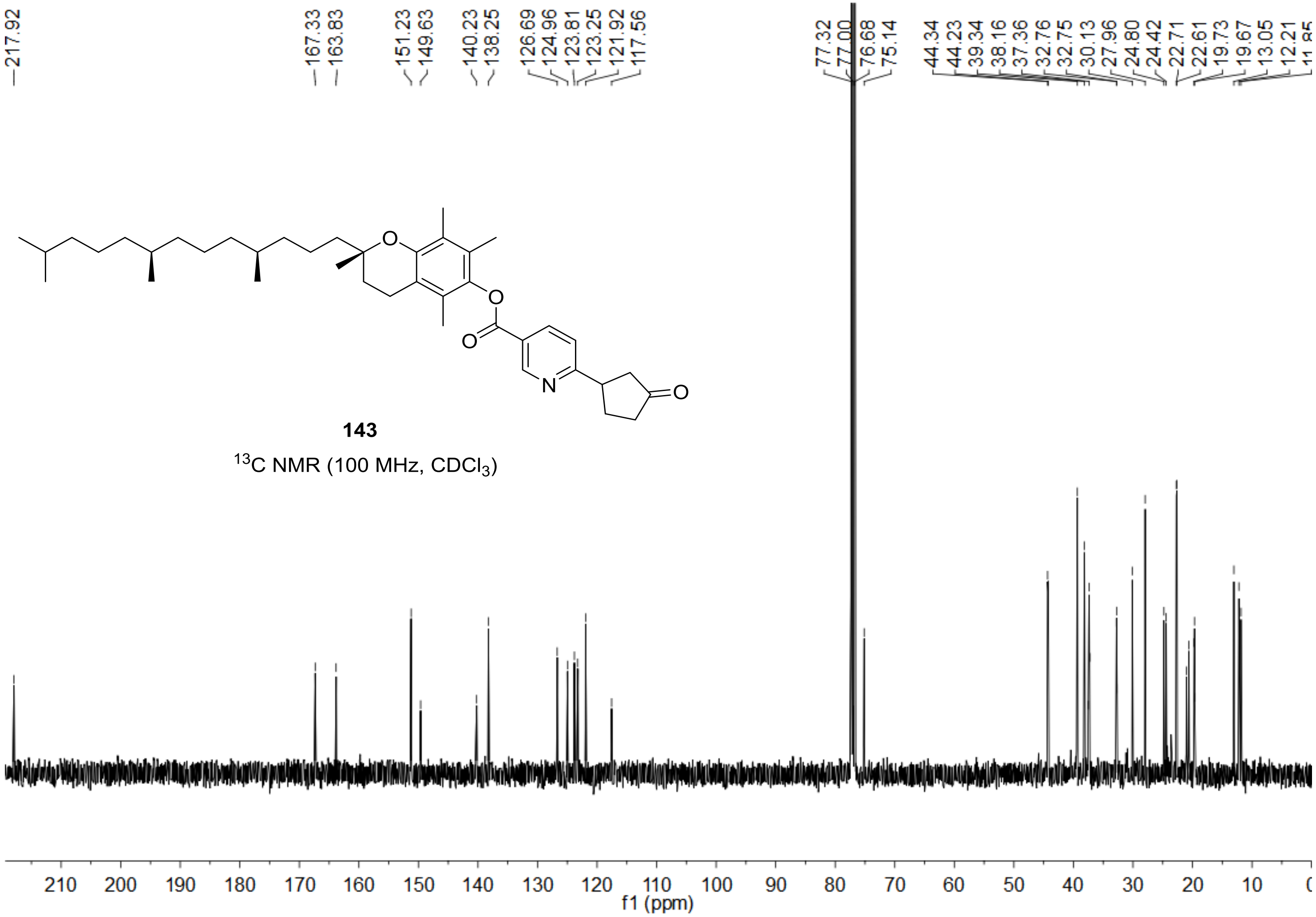

143

^{13}C NMR (100 MHz, $CDCl_3$)

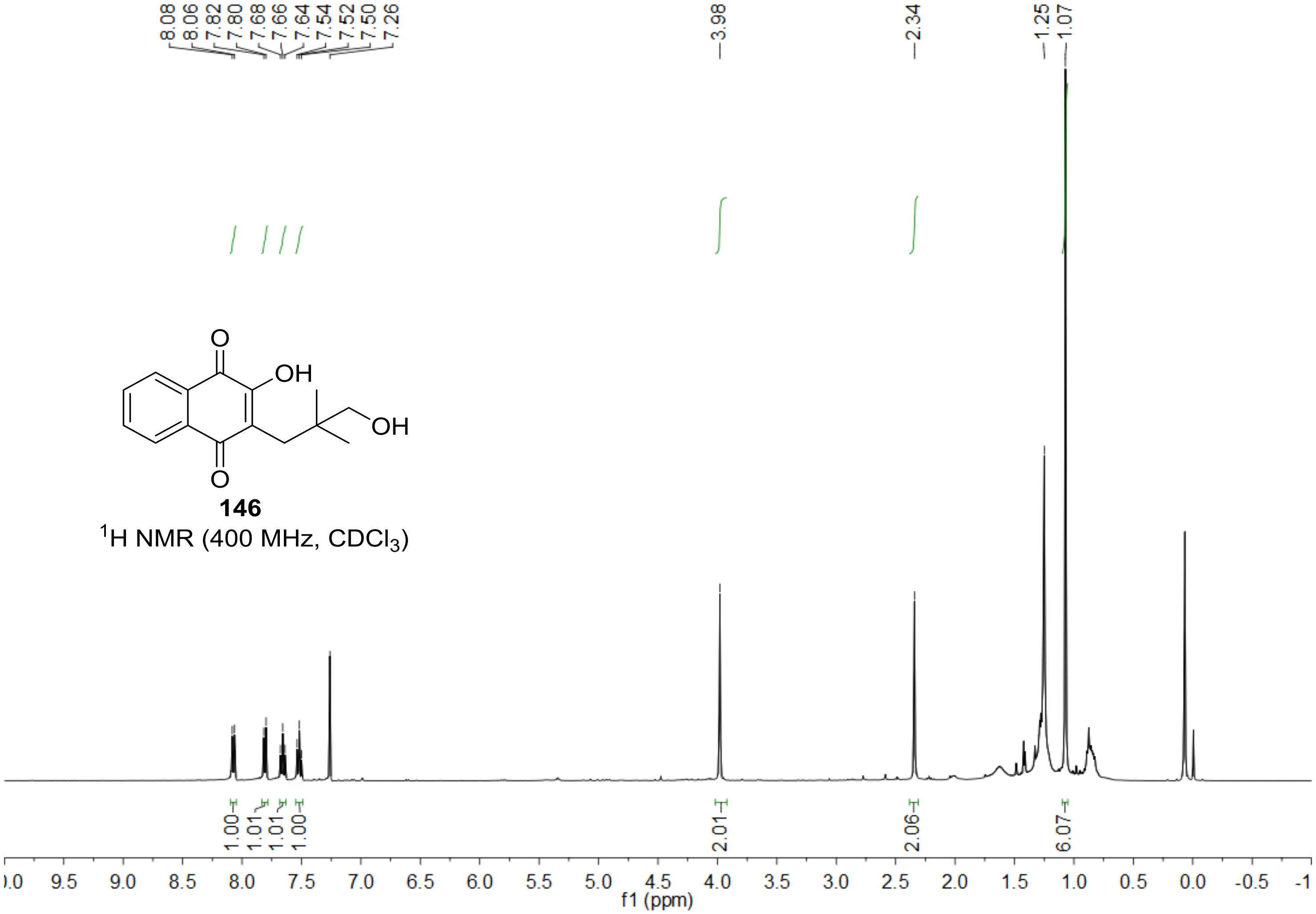

146

^{1}H NMR (400 MHz, $CDCl_3$)

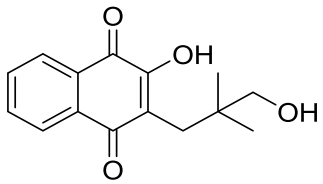

146

^{13}C NMR (100 MHz, $CDCl_3$)

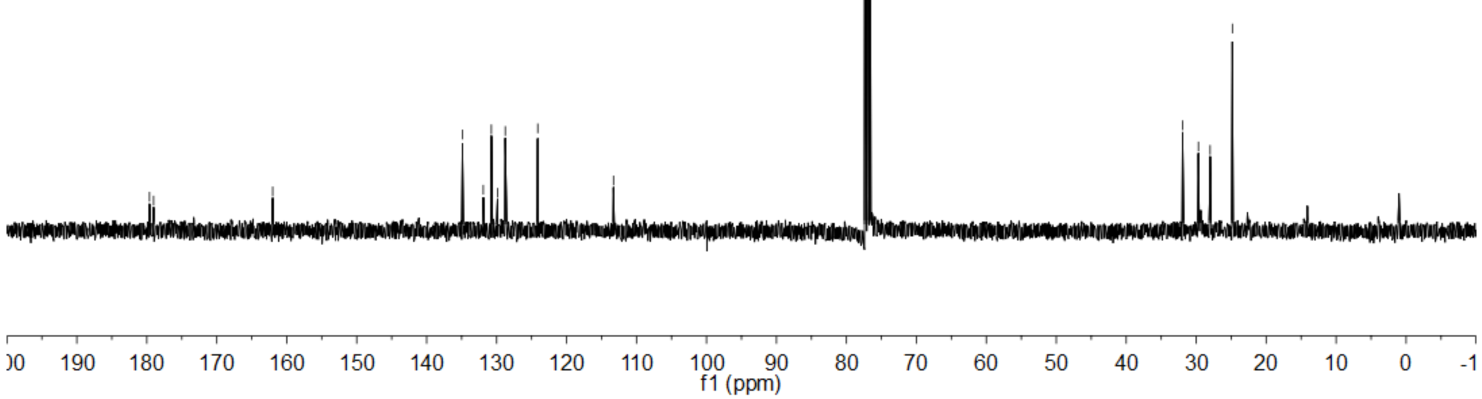

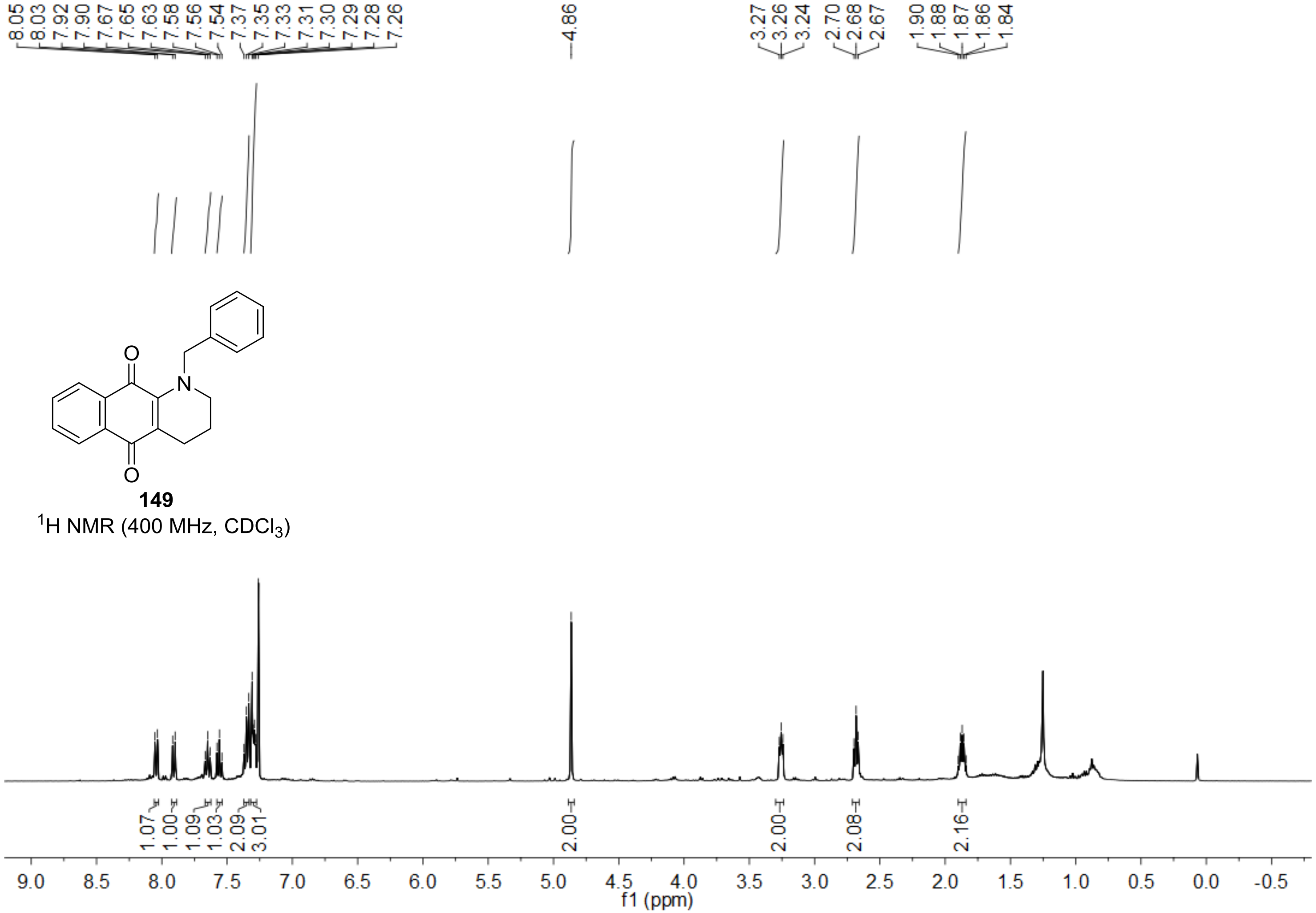

8.05
8.03
7.92
7.90
7.67
7.65
7.63
7.58
7.56
7.54
7.37
7.35
7.33
7.31
7.30
7.29
7.28
7.26
4.86
3.27
3.26
3.24
2.70
2.68
2.67
1.90
1.88
1.87
1.86
1.84
O
N
O
149
^{1}H NMR (400 MHz, $CDCl_3$)
1.07
1.00
1.09
1.03
2.09
3.01
2.00
2.00
2.08
2.16
9.0
8.5
8.0
7.5
7.0
6.5
6.0
5.5
5.0
4.5
4.0
3.5
3.0
2.5
2.0
1.5
1.0
0.5
0.0
-0.5
f1 (ppm)

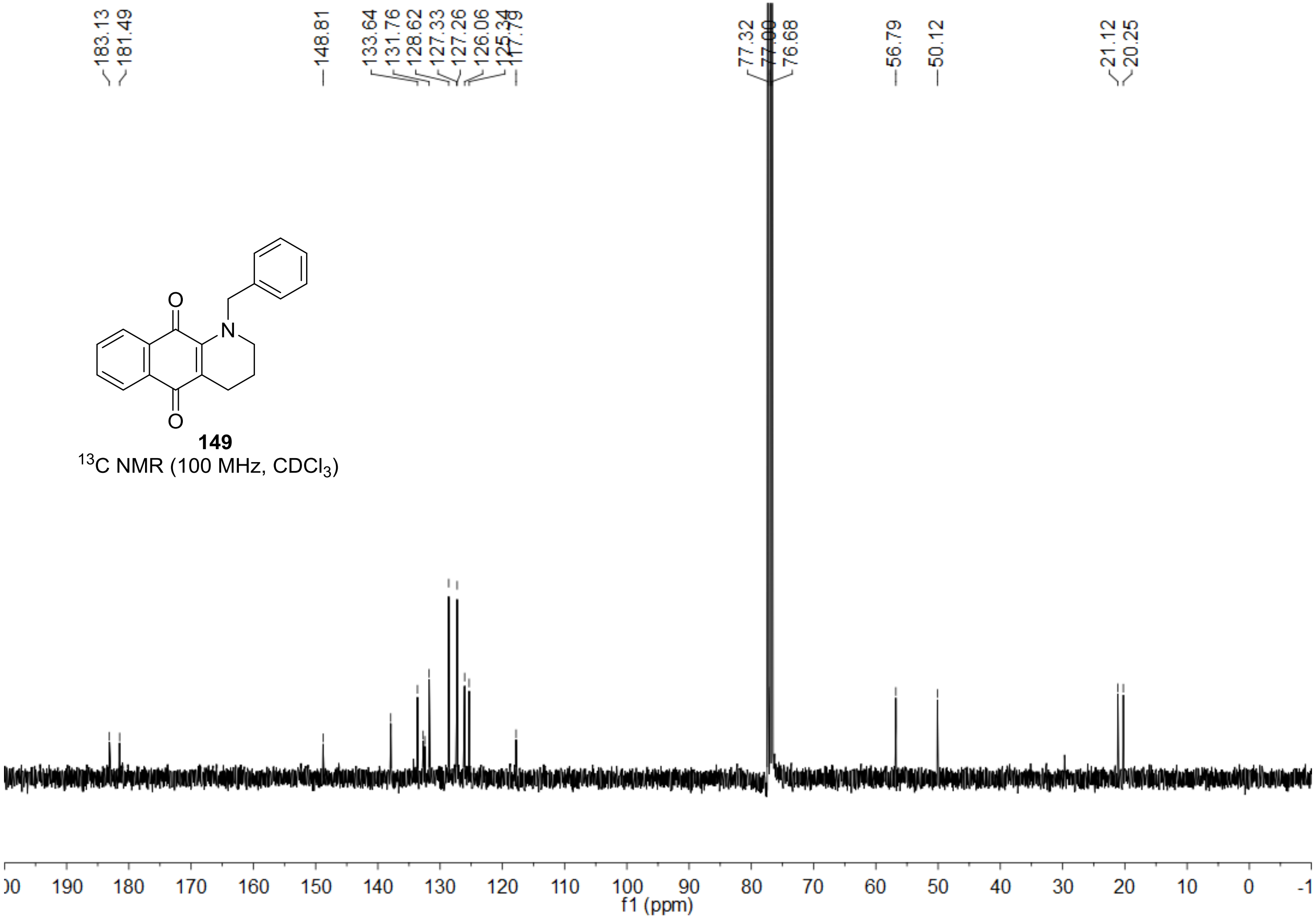
183.13
181.49
148.81
133.64
131.76
128.62
127.33
127.26
126.06
125.34
117.79
77.32
77.00
76.68
56.79
50.12
21.12
20.25
149
^{13}C NMR (100 MHz, $CDCl_3$)
190 180 170 160 150 140 130 120 110 100 90 80 70 60 50 40 30 20 10 0
f1 (ppm)

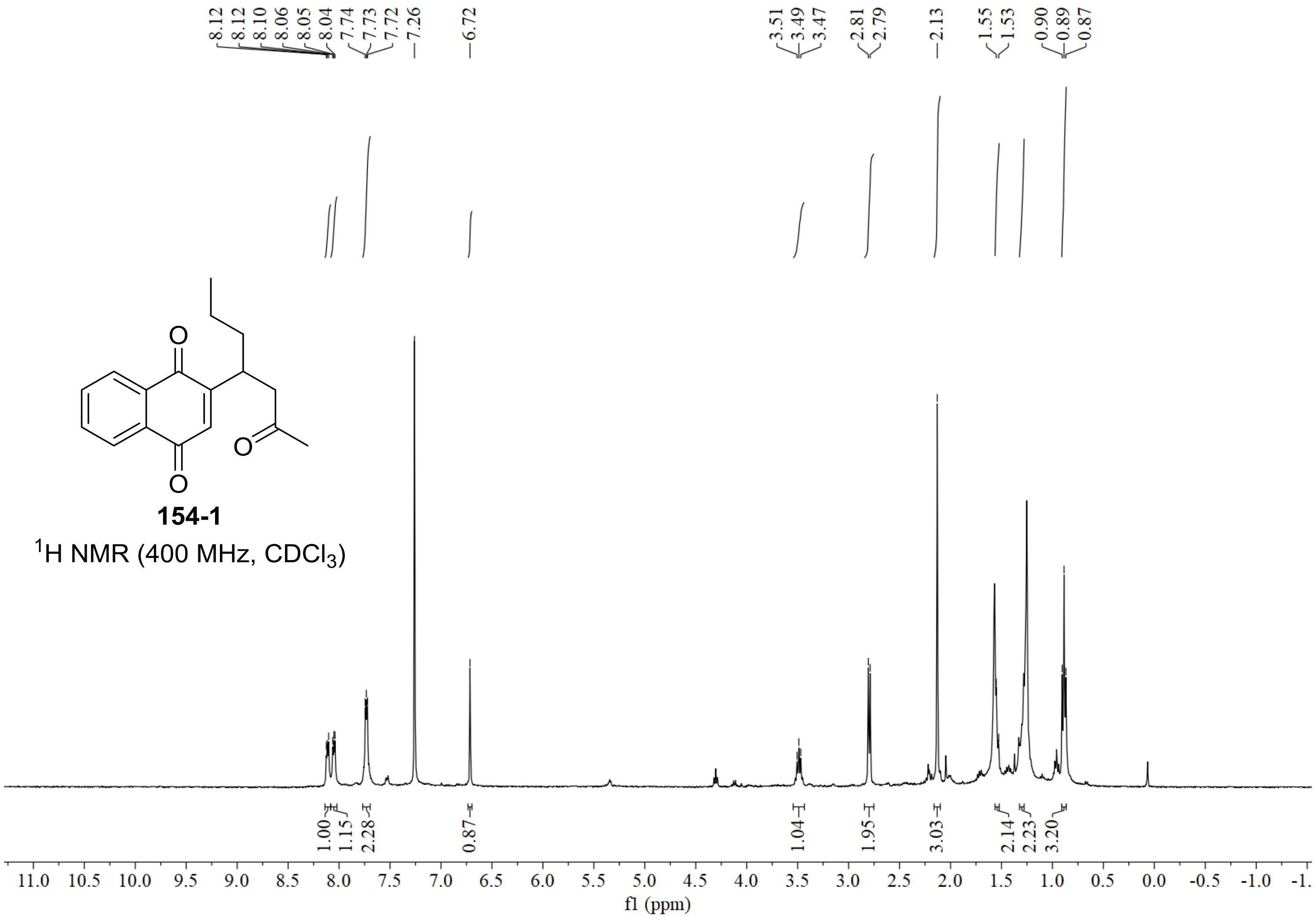

154-1

^{1}H NMR (400 MHz, $CDCl_3$)

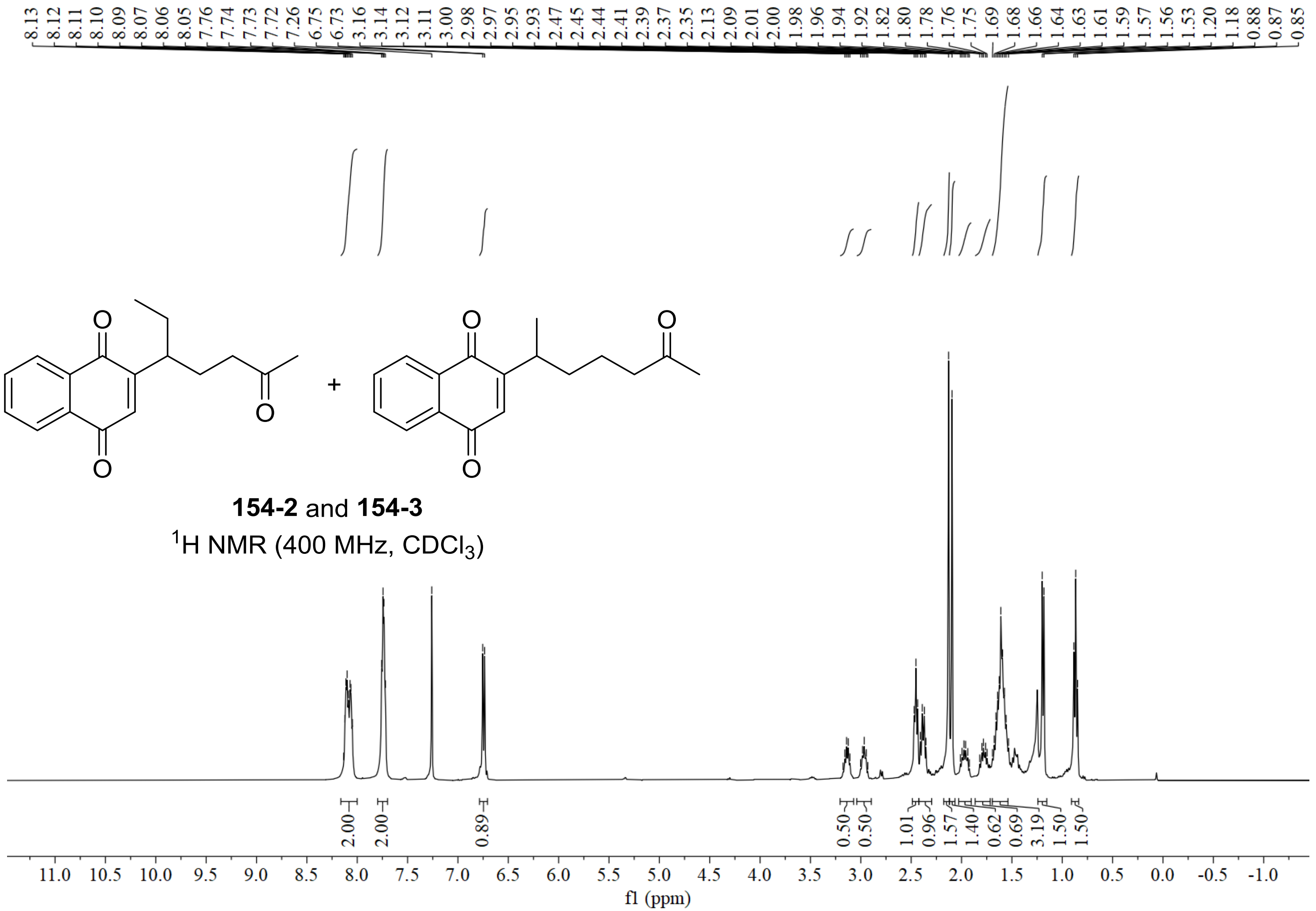
8.13 8.12 8.11 8.10 8.09 8.07 8.06 8.05 7.76 7.74 7.73 7.72 7.26 6.75 6.73 3.16 3.14 3.12 3.11 3.00 2.98 2.97 2.95 2.93 2.47 2.45 2.44 2.41 2.39 2.37 2.35 2.13 2.09 2.01 2.00 1.98 1.96 1.94 1.92 1.82 1.80 1.78 1.76 1.75 1.69 1.68 1.66 1.64 1.63 1.61 1.59 1.57 1.56 1.53 1.20 1.18 0.88 0.87 0.85
O
O
O
+
O
O
O
154-2 and **154-3**
^{1}H NMR (400 MHz, $CDCl_3$)
2.00 2.00 0.89 0.50 0.50 1.01 0.96 1.57 1.40 0.62 0.69 3.19 1.50 1.50
11.0 10.5 10.0 9.5 9.0 8.5 8.0 7.5 7.0 6.5 6.0 5.5 5.0 4.5 4.0 3.5 3.0 2.5 2.0 1.5 1.0 0.5 0.0 -0.5 -1.0
f1 (ppm)

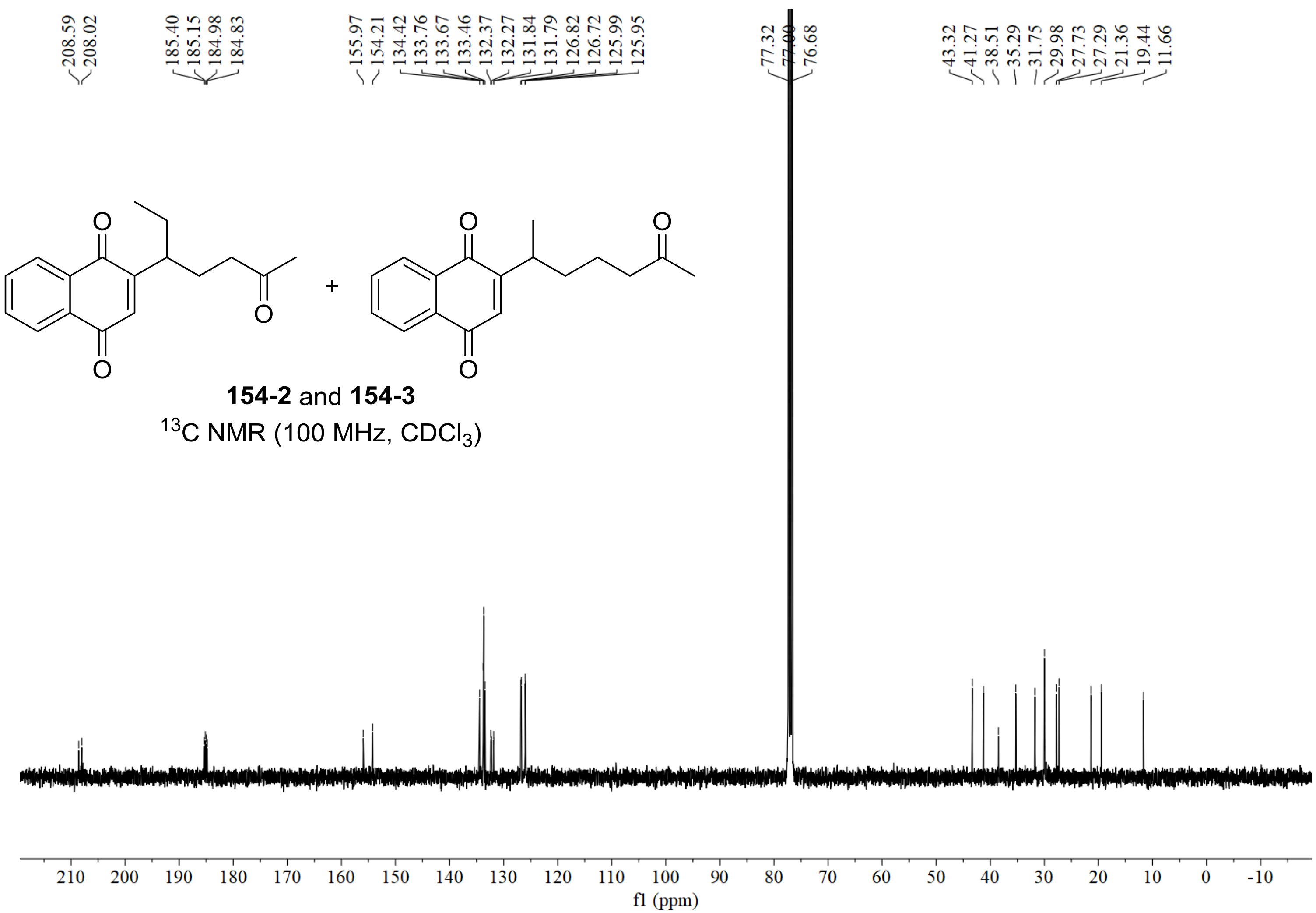

208.59
208.02
185.40
185.15
184.98
184.83
155.97
154.21
134.42
133.76
133.67
133.46
132.37
132.27
131.84
131.79
126.82
126.72
125.99
125.95
77.32
77.00
76.68
43.32
41.27
38.51
35.29
31.75
29.98
27.73
27.29
21.36
19.44
11.66
154-2 and 154-3
^{13}C NMR (100 MHz, $CDCl_3$)
210 200 190 180 170 160 150 140 130 120 110 100 90 80 70 60 50 40 30 20 10 0 -10
f1 (ppm)

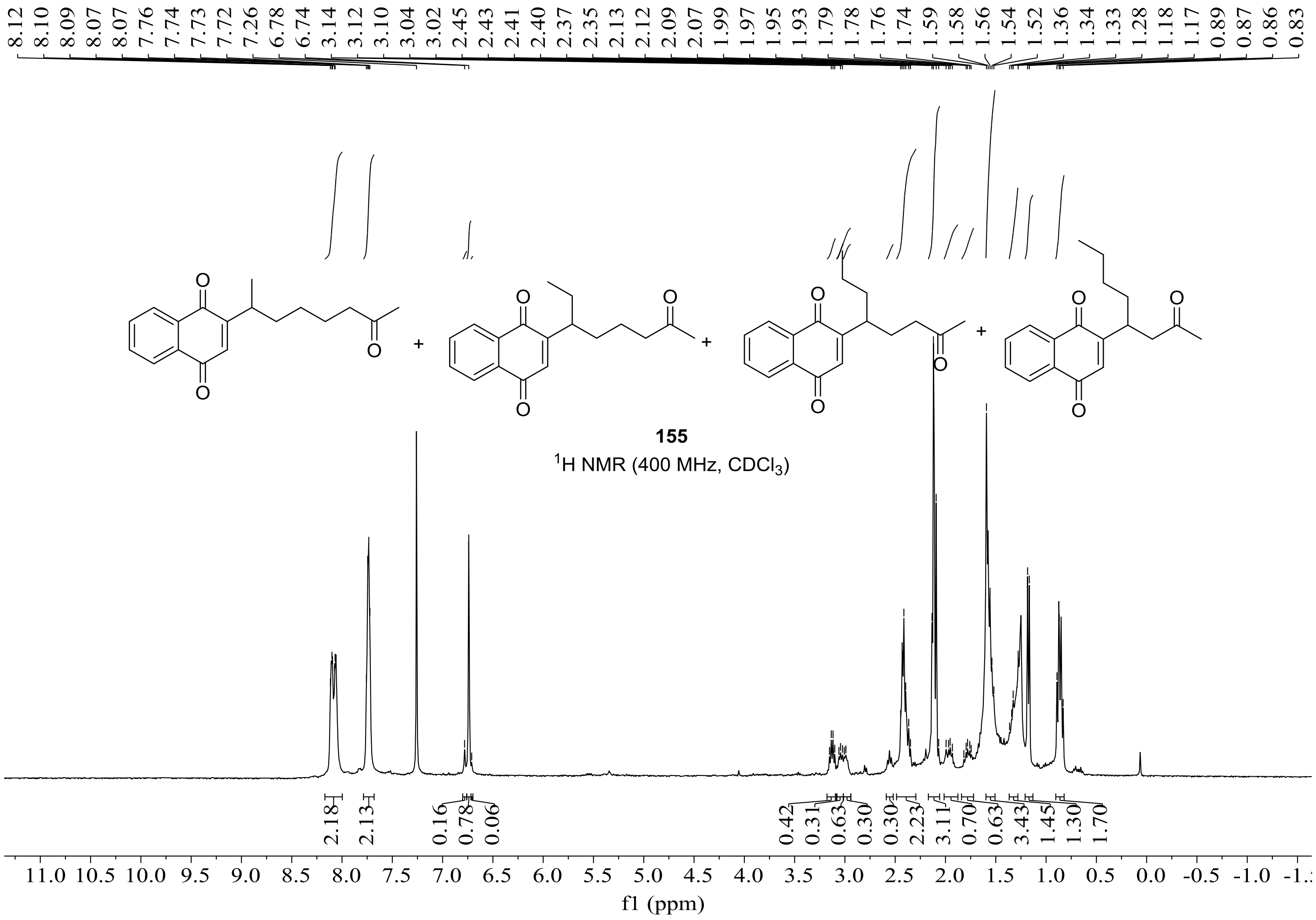
155
1H NMR (400 MHz, CDCl3)
f1 (ppm)

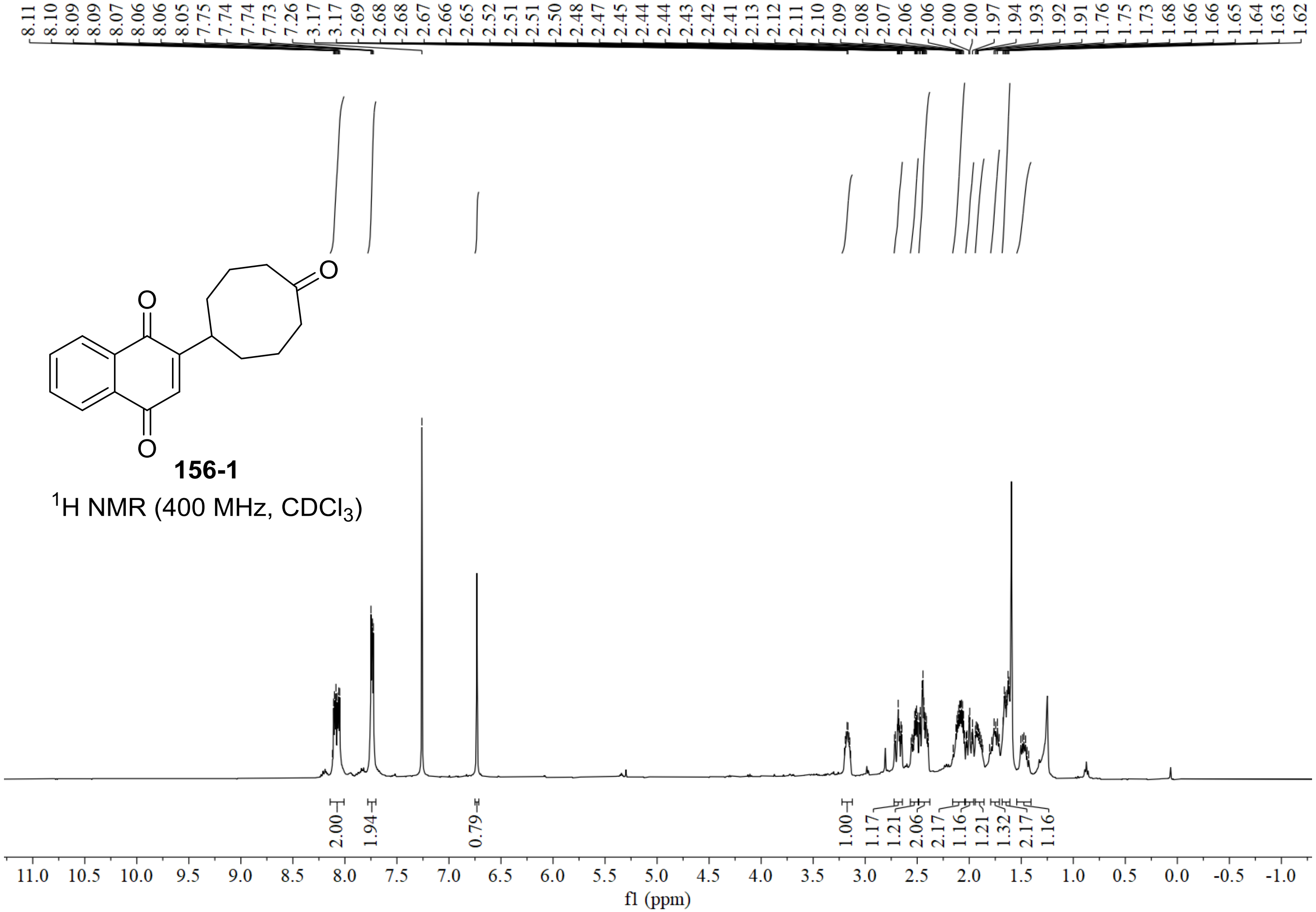

156-1

^{1}H NMR (400 MHz, $CDCl_3$)

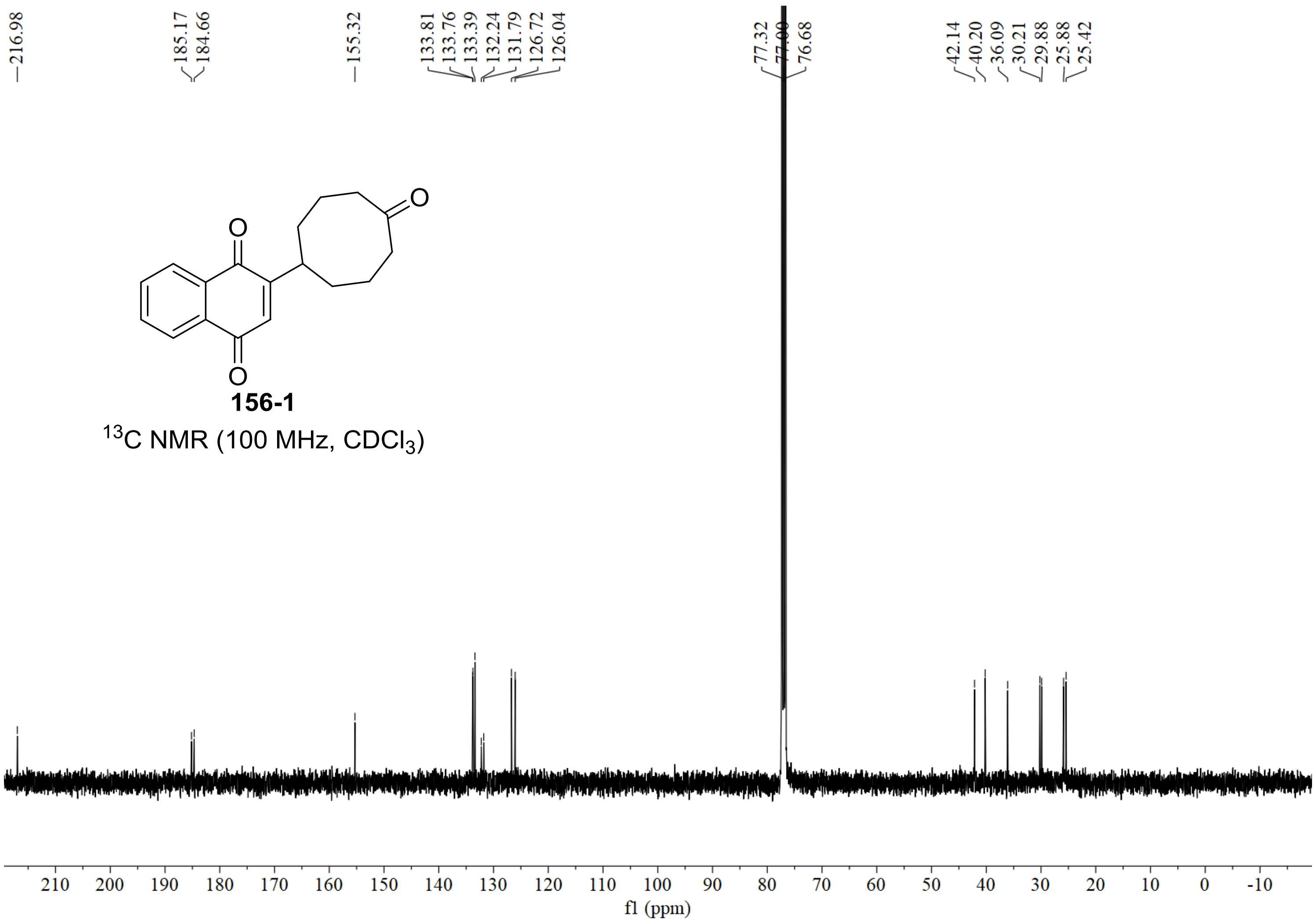

156-1

^{13}C NMR (100 MHz, $CDCl_3$)

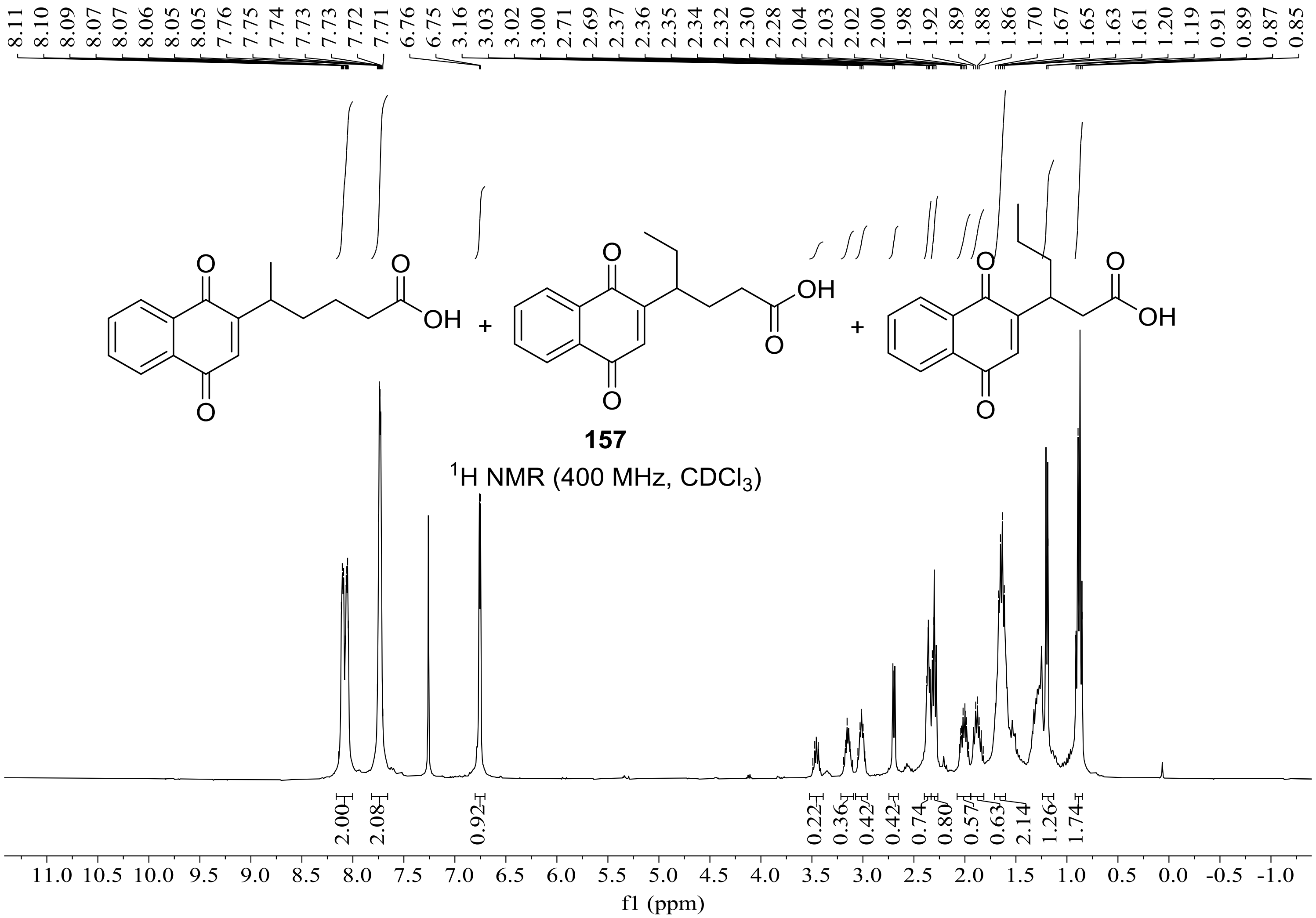
8.11 8.10 8.09 8.07 8.07 8.06 8.05 8.05 7.76 7.75 7.74 7.73 7.73 7.72 7.71 6.76 6.75 3.16 3.03 3.02 3.00 2.71 2.69 2.37 2.36 2.35 2.34 2.32 2.30 2.28 2.04 2.03 2.02 2.00 1.98 1.92 1.89 1.88 1.86 1.70 1.67 1.65 1.63 1.61 1.20 1.19 0.91 0.89 0.87 0.85
OH
+
OH
+
OH
157
1H NMR (400 MHz, CDCl3)
2.00
2.08
0.92
0.22
0.36
0.42
0.42
0.74
0.80
0.57
0.63
2.14
1.26
1.74
11.0 10.5 10.0 9.5 9.0 8.5 8.0 7.5 7.0 6.5 6.0 5.5 5.0 4.5 4.0 3.5 3.0 2.5 2.0 1.5 1.0 0.5 0.0 -0.5 -1.0
f1 (ppm)

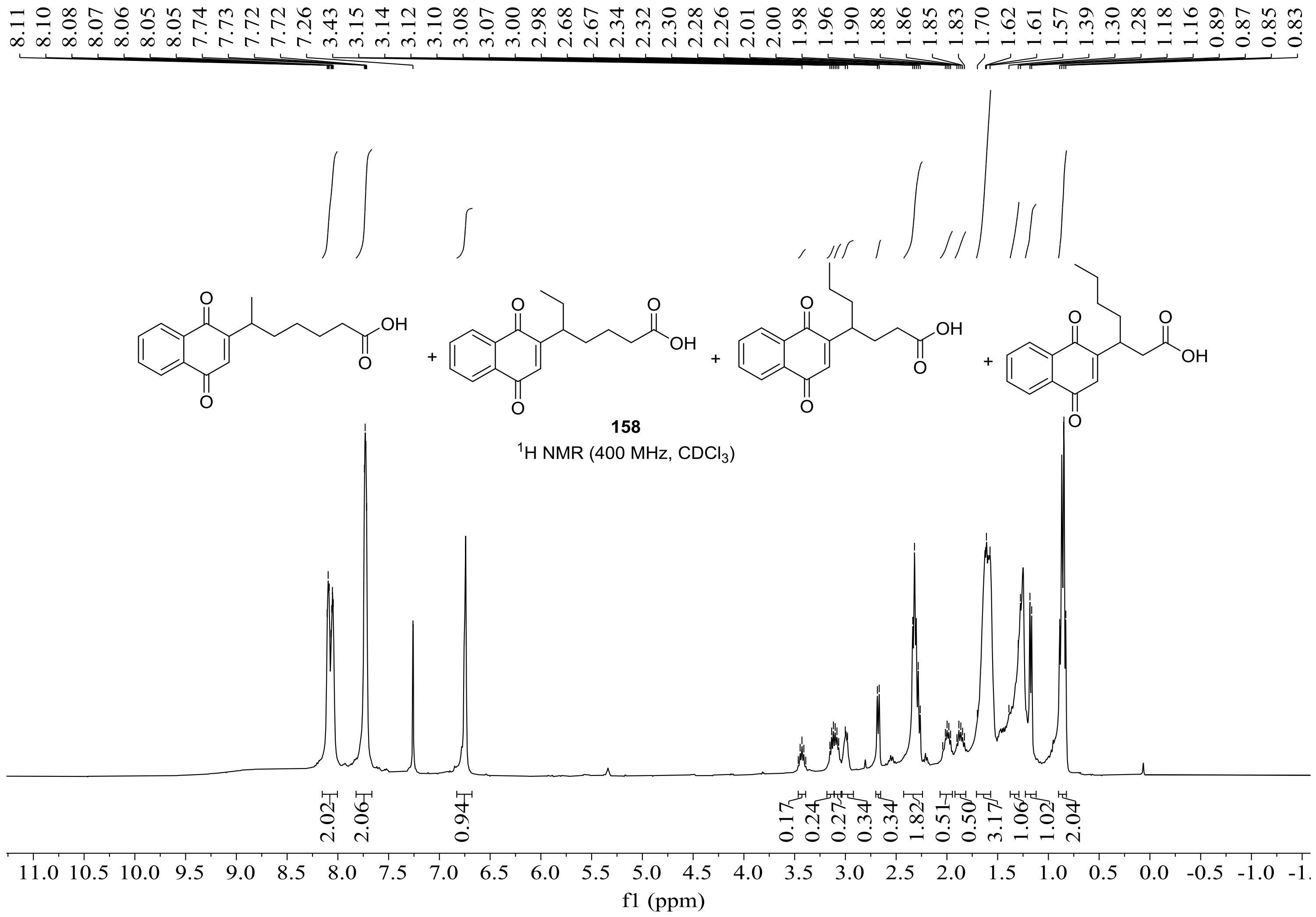
8.11
8.10
8.08
8.07
8.06
8.05
8.05
7.74
7.73
7.72
7.72
7.26
3.43
3.15
3.14
3.12
3.10
3.08
3.07
3.00
2.98
2.68
2.67
2.34
2.32
2.30
2.28
2.26
2.01
2.00
1.98
1.96
1.90
1.88
1.86
1.85
1.83
1.70
1.62
1.61
1.57
1.39
1.30
1.28
1.18
1.16
0.89
0.87
0.85
0.83
OH
O
+
158
^{1}H NMR (400 MHz, CDCl$_3$)
2.02
2.06
0.94
0.17
0.24
0.27
0.34
0.34
1.82
0.51
0.50
3.17
1.06
1.02
2.04
11.0 10.5 10.0 9.5 9.0 8.5 8.0 7.5 7.0 6.5 6.0 5.5 5.0 4.5 4.0 3.5 3.0 2.5 2.0 1.5 1.0 0.5 0.0 -0.5 -1.0
f1 (ppm)

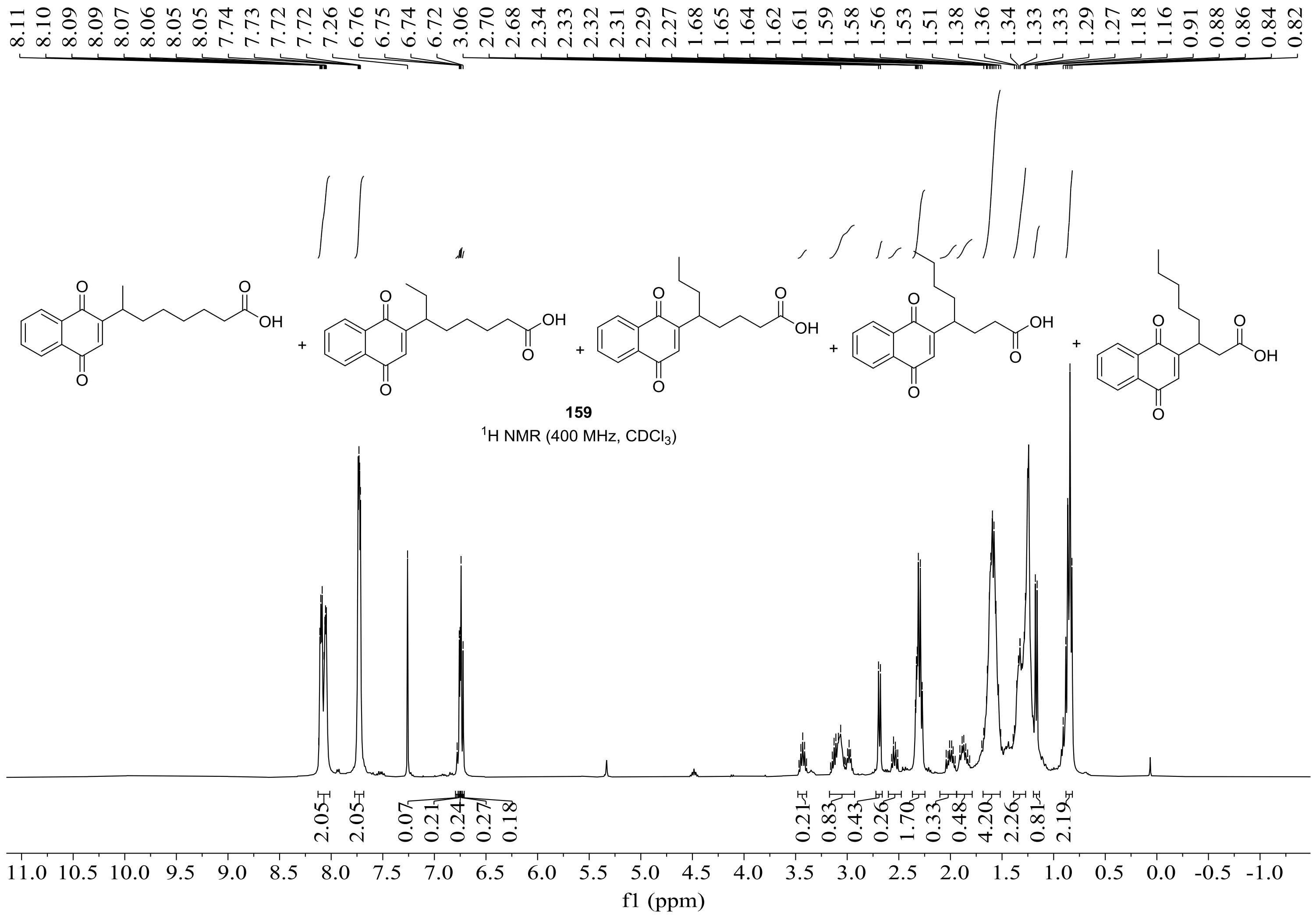
8.11 8.10 8.09 8.09 8.07 8.06 8.05 8.05 7.74 7.73 7.72 7.72 7.26 6.76 6.75 6.74 6.72 3.06 2.70 2.68 2.34 2.33 2.32 2.31 2.29 2.27 1.68 1.65 1.64 1.62 1.61 1.59 1.58 1.56 1.53 1.51 1.38 1.36 1.34 1.33 1.33 1.29 1.27 1.18 1.16 0.91 0.88 0.86 0.84 0.82
159
^{1}H NMR (400 MHz, $CDCl_3$)
2.05 2.05 0.07 0.21 0.24 0.27 0.18 0.21 0.83 0.43 0.26 1.70 0.33 0.48 4.20 2.26 0.81 2.19
11.0 10.5 10.0 9.5 9.0 8.5 8.0 7.5 7.0 6.5 6.0 5.5 5.0 4.5 4.0 3.5 3.0 2.5 2.0 1.5 1.0 0.5 0.0 -0.5 -1.0
f1 (ppm)

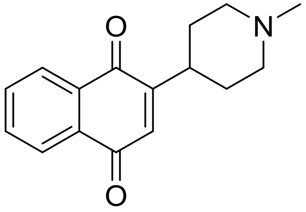

160

^{1}H NMR (400 MHz, $CDCl_3$)

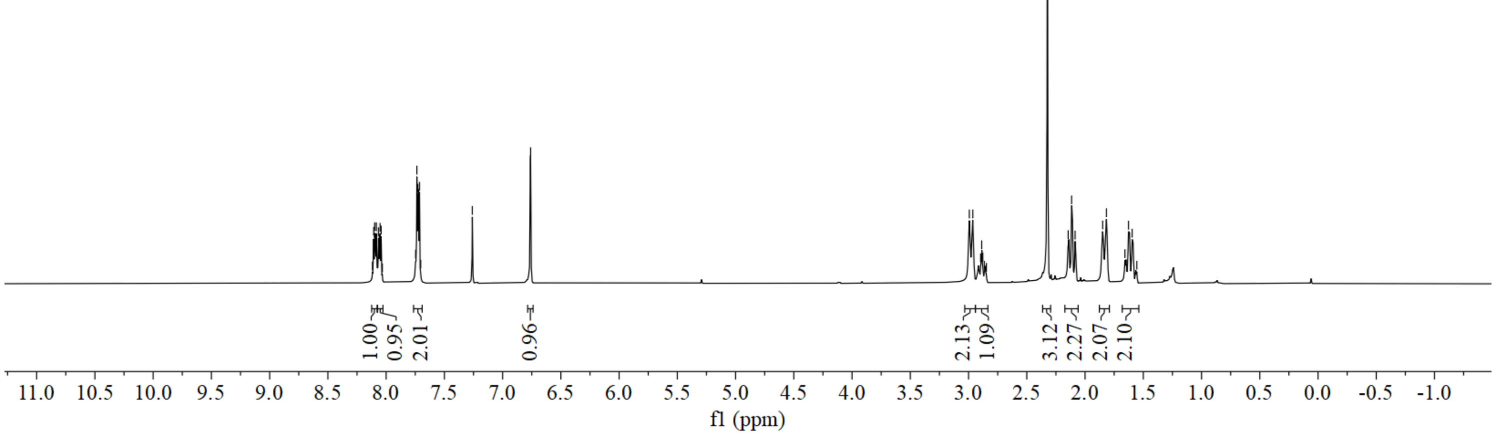

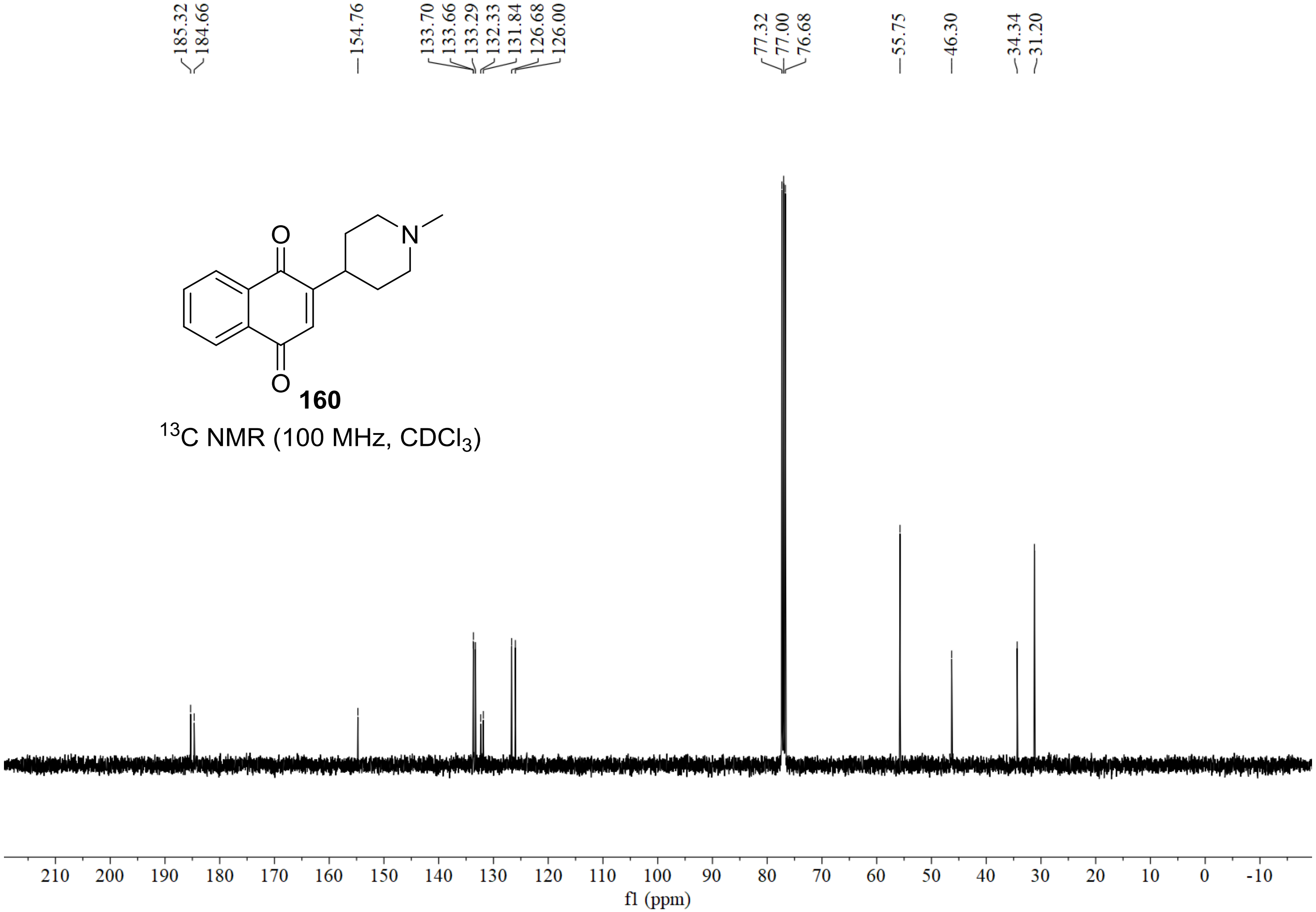
185.32
184.66
154.76
133.70
133.66
133.29
132.33
131.84
126.68
126.00
77.32
77.00
76.68
55.75
46.30
34.34
31.20
O
N
O
160
13C NMR (100 MHz, CDCl3)
210 200 190 180 170 160 150 140 130 120 110 100 90 80 70 60 50 40 30 20 10 0 -10
f1 (ppm)

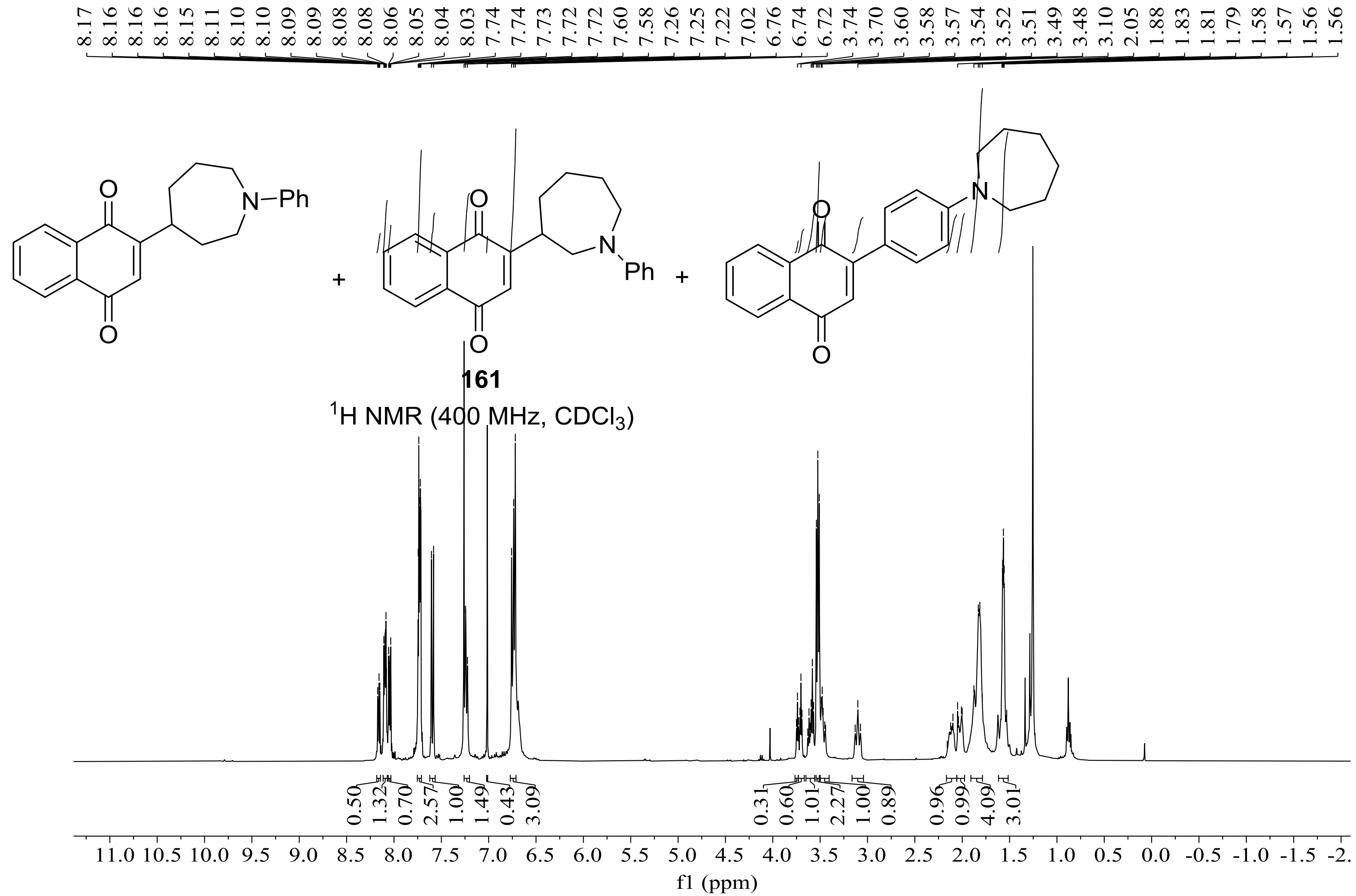

8.17 8.16 8.16 8.16 8.15 8.11 8.10 8.10 8.09 8.09 8.08 8.08 8.06 8.05 8.04 8.03 7.74 7.74 7.73 7.72 7.72 7.60 7.58 7.26 7.25 7.22 7.02 6.76 6.74 6.72 3.74 3.70 3.60 3.58 3.57 3.54 3.52 3.51 3.49 3.48 3.10 2.05 1.88 1.83 1.81 1.79 1.58 1.57 1.56 1.56
N–Ph
Ph
+
161
^{1}H NMR (400 MHz, $CDCl_3$)
0.50 1.32 0.70 2.57 1.00 1.49 0.43 3.09
0.31 0.60 1.01 2.27 1.00 0.89
0.96 0.99 4.09 3.01
11.0 10.5 10.0 9.5 9.0 8.5 8.0 7.5 7.0 6.5 6.0 5.5 5.0 4.5 4.0 3.5 3.0 2.5 2.0 1.5 1.0 0.5 0.0 -0.5 -1.0 -1.5 -2.
f1 (ppm)

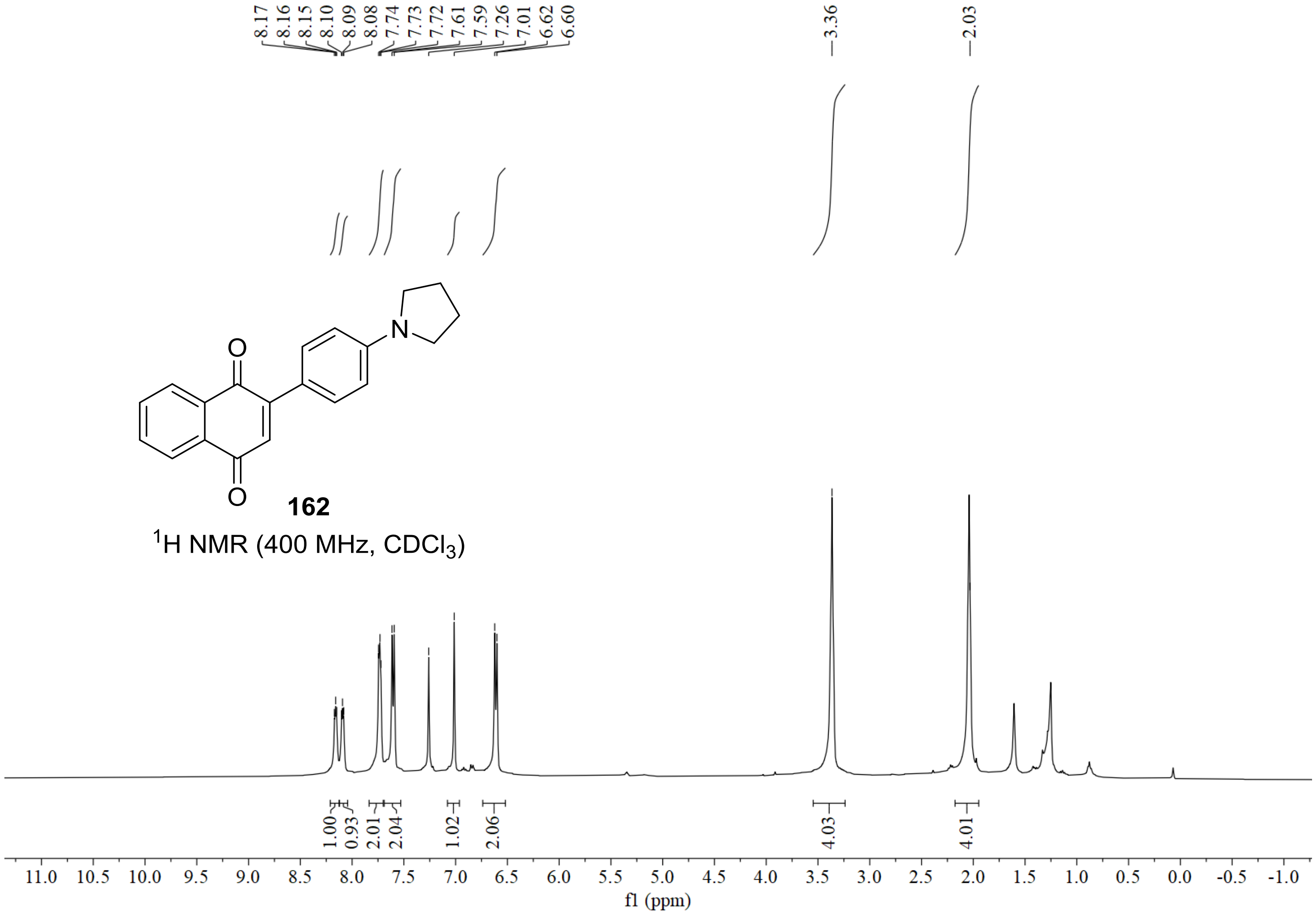

162

^1H NMR (400 MHz, $CDCl_3$)

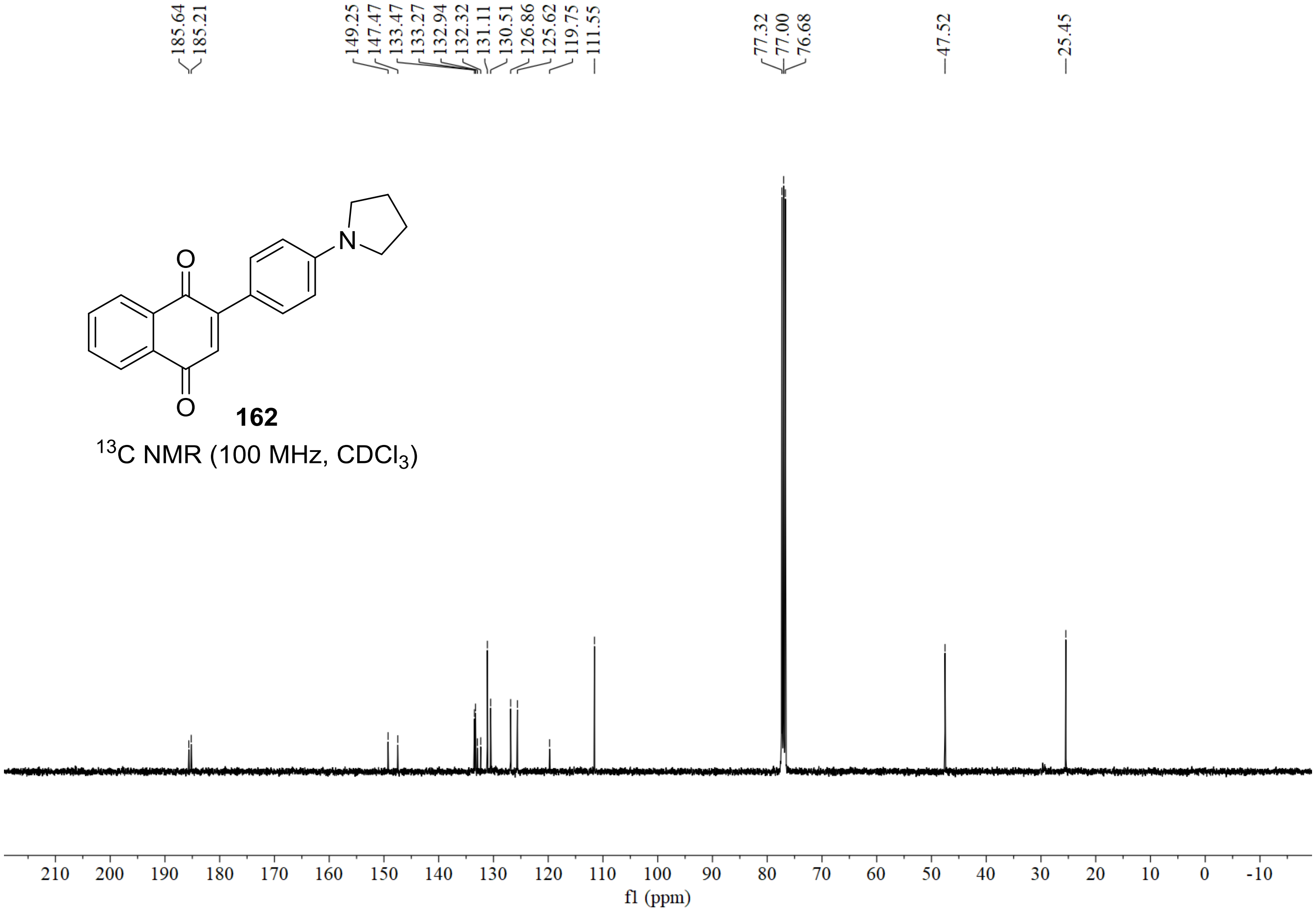

^{13}C NMR (100 MHz, $CDCl_3$)

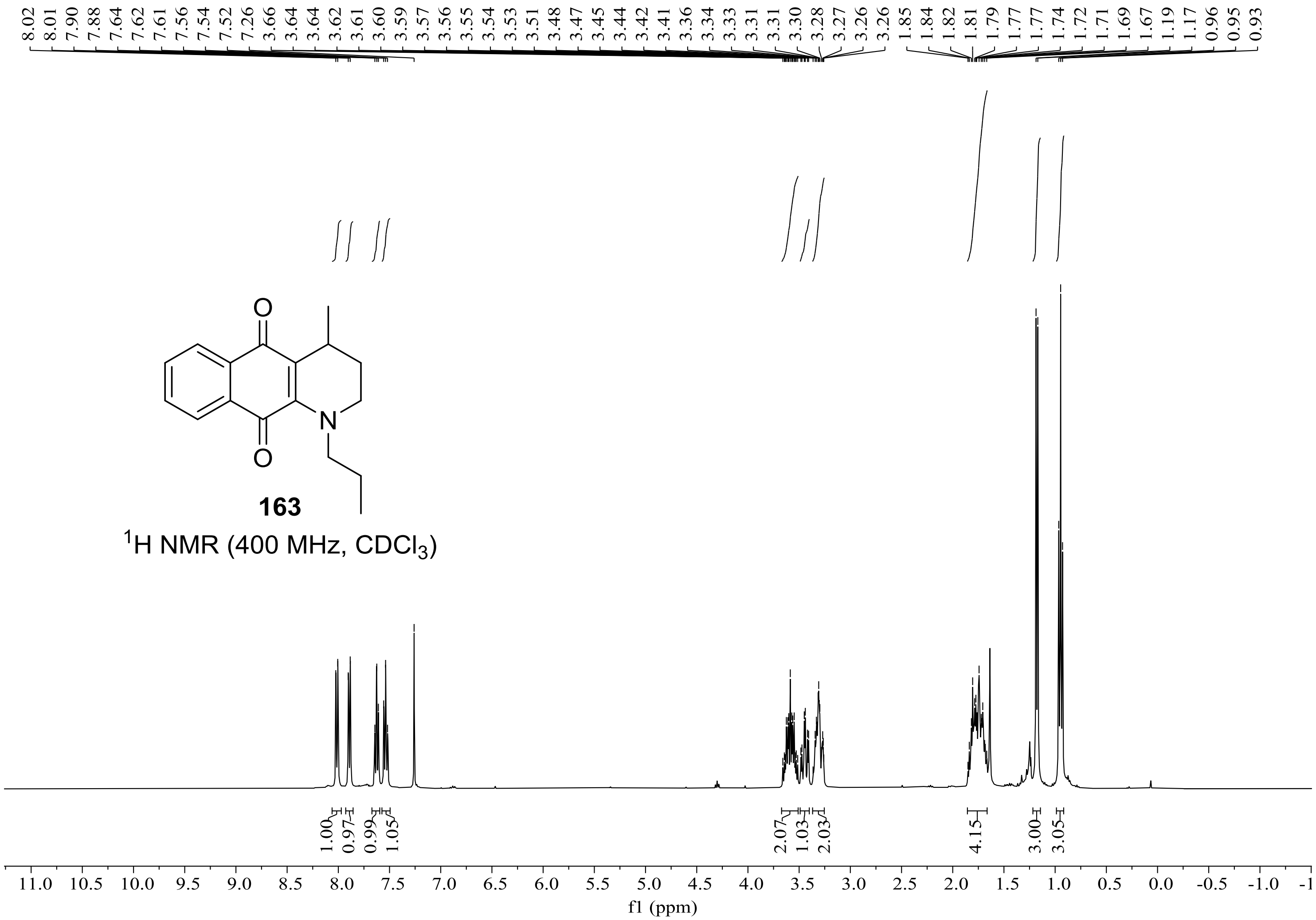

8.02
8.01
7.90
7.88
7.64
7.62
7.61
7.56
7.54
7.52
7.26
3.66
3.64
3.64
3.62
3.61
3.60
3.59
3.57
3.56
3.55
3.54
3.53
3.51
3.48
3.47
3.45
3.44
3.42
3.41
3.36
3.34
3.33
3.31
3.31
3.30
3.28
3.27
3.26
3.26
1.85
1.84
1.82
1.81
1.79
1.77
1.77
1.74
1.72
1.71
1.69
1.67
1.19
1.17
0.96
0.95
0.93
163
^{1}H NMR (400 MHz, CDCl$_3$)
1.00
0.97
0.99
1.05
2.07
1.03
2.03
4.15
3.00
3.05
11.0 10.5 10.0 9.5 9.0 8.5 8.0 7.5 7.0 6.5 6.0 5.5 5.0 4.5 4.0 3.5 3.0 2.5 2.0 1.5 1.0 0.5 0.0 -0.5 -1.0 -1
f1 (ppm)

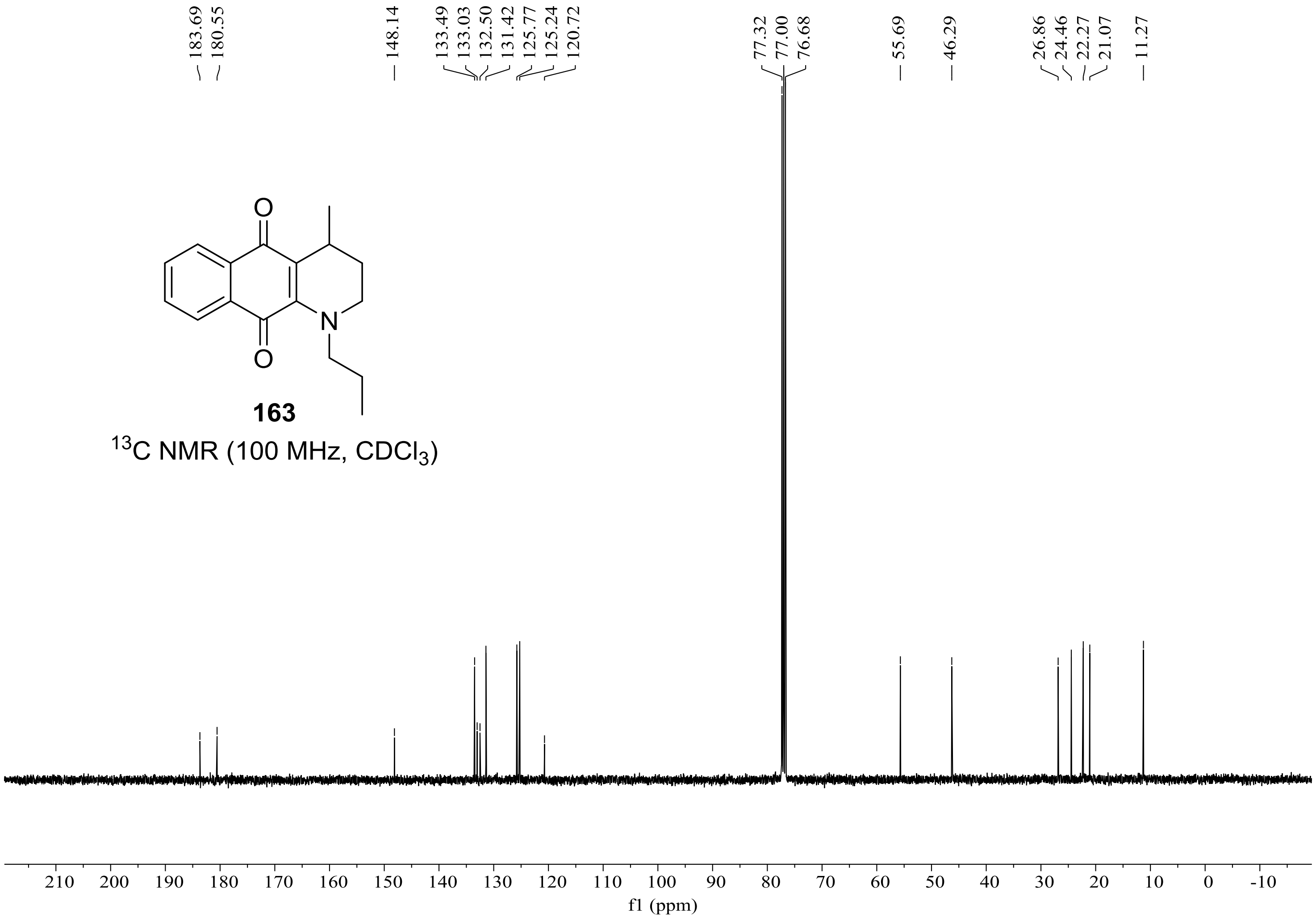
183.69
180.55
148.14
133.49
133.03
132.50
131.42
125.77
125.24
120.72
77.32
77.00
76.68
55.69
46.29
26.86
24.46
22.27
21.07
11.27
O
O
N
163
^{13}C NMR (100 MHz, $CDCl_3$)
210 200 190 180 170 160 150 140 130 120 110 100 90 80 70 60 50 40 30 20 10 0 -10
f1 (ppm)

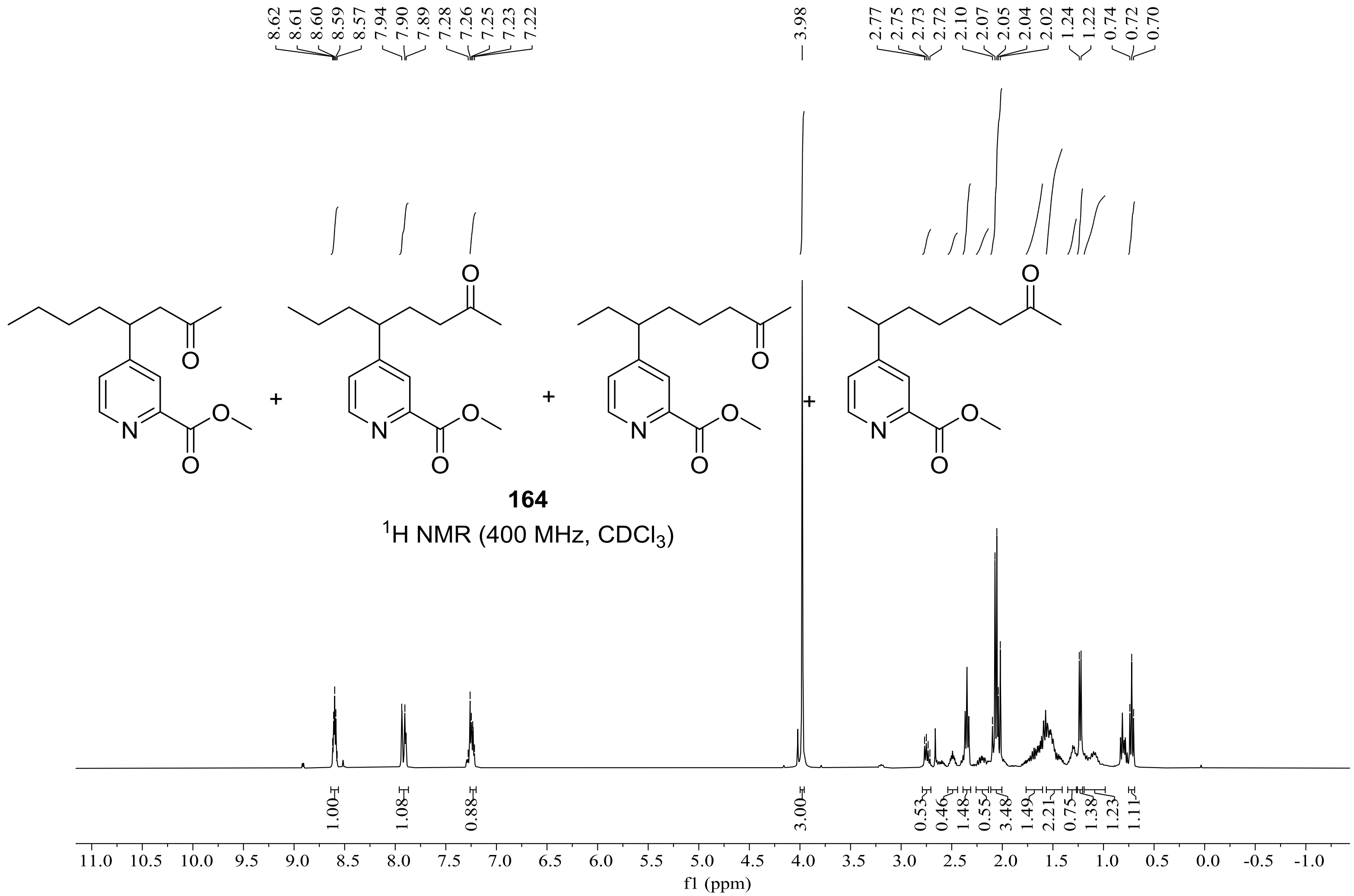
164
^{1}H NMR (400 MHz, CDCl$_3$)
f1 (ppm)

References

S1. Li, Z., Ebule, R., Kostyo, J., Hammond, G. B. & Xu, B. HBr-DMPU: The first aprotic organic solution of hydrogen bromide. *Chem. Eur. J.* **23**, 12739-12743 (2017).

S2. Cheng, L. et al. Iron-catalyzed arene C-H hydroxylation. *Science* **374**, 77-81 (2021).

S3. Goh, Y. M. & Nam, W. Significant electronic effect of porphyrin ligand on the reactivities of high-valent iron(IV) oxo porphyrin cation radical complexes. *Inorg. Chem.* **38**, 914–920 (1999).

S4. Groves, J. T., Haushalter, R. C., Nakamura, M., Nemo, T. E. & Evans, B. J. High-valent iron-porphyrin complexes related to peroxidase and cytochrome P-450. *J. Am. Chem. Soc.* **103**, 2884-2886 (1981).

S5. Baral, E. R., Kim, S. H. & Y. R. Lee, Copper-catalyzed C(sp^2)-C(sp^3) cross-dehydrogenative coupling of quinones with cyclic alkanes: One-step access to parvaquone and its analogs. *Asian J. Org. Chem.* **5**, 1134-1141 (2016).
